# A Reassessment of the Genomic Ancestry of the World's Largest Captive Baboon Colony

**DOI:** 10.1002/ajp.70096

**Published:** 2025-11-25

**Authors:** Giacomo Mercuri, Fabrizio Dallaspezia, Francesco Montinaro, Cristian Capelli

**Affiliations:** ^1^ Department of Chemistry, Life Sciences and Environmental Sustainability University of Parma Parma Italy; ^2^ Department of Biosciences, Biotechnology and Environment University of Bari Bari Italy; ^3^ Institute of Genomics, University of Tartu Tartu Estonia

**Keywords:** ancestry composition, baboons, SNPRC colony

## Abstract

The Southwest National Primate Research Center of San Antonio, Texas, hosts one of the largest captive colonies of baboons used for biomedical research. Pedigreed animals can be traced back to the second part of the last century from individuals of two *Papio* species: *P. anubis* and *P. cynocephalus*. We leveraged recently published genomic data from more than 800 baboons to investigate the ancestry profile of the colony members. By combining phylogenetic analysis of mitochondrial DNA and nuclear genomic ancestry estimations, we confirmed *P. anubis* and *P. cynocephalus* as the main sources of the colony genetic variation. Previously unreported contributions from additional *Papio* species were also detected in almost 5% of the colony samples, of which *P. hamadryas* was the most notable, while others occurred sporadically across the data set. This extensive ancestry characterisation will be of help in biomedical investigations making use of baboons from the Southwest National Primate Research Center.

## Summary

- Genomic and mitochondrial analysis of almost 900 baboons confirms primary ancestry of samples from *P. anubis* and *P. cynocephalus* in the Southwest National Primate Research Center in Texas, a major biomedical research colony
- Unexpectedly, genetic contributions from other *Papio* species, mostly *P. hamadryas*, were detected in almost 5% of the analysed individuals.
- These findings enhance understanding of the genetic diversity of this widely used colony, which have to be taken into consideration when designing future studies.

## Graphical abstract

Mitochondrial analysis revealed unexpected lineages inside the *P. anubis*/*P. cynocephalus* SNPRC colony in San Antonio, Texas. Whole‐genome ancestry reconstruction further explored these results, identifying several individuals with substantial (> 0.10) ancestry fraction coming from unexpected Papio species (here reported average ancestry estimate).

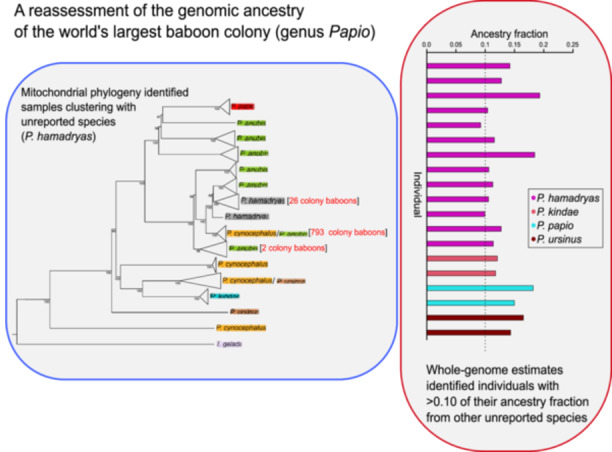

## 1 Introduction

The Southwest National Primate Research Center (SNPRC) is one of the National Primate Research Centers in the United States. Located in San Antonio, Texas, the centre is affiliated with the Texas Biomedical Research Institute and currently hosts marmosets, baboons, macaques, and chimpanzees. The baboon colony in particular is the largest captive population of baboons in North America and the world's largest colony of baboons used for biomedical research (https://snprc.org/primate-research-center).

The Texas Primate Research facility has a long history. The baboon colony was originally established at the Southwest Foundation for Research and Education (SFRE) in San Antonio in 1958 (Moore 1975). In 1972, a breeding programme was initiated including 250 females, all classified as *P. cynocephalus* following the 1966 revised taxonomy (Buettner‐Janusch 1966; Kraemer et al. 1975). Pedigrees were recorded for a subset of these individuals by pairing single males with large harems of 20–30 females (Kraemer et al. 1975; Rogers and Hixson 1997). Records indicate that approximately 600 baboons were listed at SFRE in both 1972 and 1974 (Kraemer and Vera Cruz 1972; Goodwin 1974; Moore 1975). In 1974, most baboons were identified as *P. cynocephalus*, although *P. papio* and *P. anubis* were also present, all reported to have originated directly from Africa (Moore 1975). In 1979, the SFRE established a second baboon breeding programme, bringing together 180 baboons from the existing colony and importing 247 baboons from Kenya for a total of 427 individuals (Goodwin and Coelho AM 1982). Of these, 87% were reported as *P. anubis*, while 13% were *P. cynocephalus*. The colony was maintained at around 550 individuals for most of the 1980s by providing animals to biomedical facilities (Dyke et al. 1987). The foundation underwent several name changes over the decades. In 1982, it was renamed to Southwest Foundation for Biomedical Research (SFBR), and it became the Texas Biomedical Research Institute in 2011 (https://snprc.org/). Today, the baboon colony of the SNPRC consists of almost 1000 individuals, nearly all of whom are pedigreed (Ken Sayers, personal communication; March 2025).

Animals hosted in the Texas facility have been used in a variety of investigations of complex diseases, ranging from atherosclerosis to obesity and hypertension, and have also contributed to fields as diverse as reproduction and immunology, including the study of COVID‐19 (Cox et al. 2013; Mahaney et al. 2018; King et al. 2021; Garcia‐Vilanova et al. 2024; Kendall et al. 2024; See also https://snprc.org/). In addition, the SNPRC contributes to and maintains several baboon‐related research resources, ranging from frozen tissue samples to detailed morphometric datasets, which provide valuable tools for the broader scientific community (Joganic et al. 2018; Cox et al. 2013; See also https://snprc.org/).

Given the baboons' scientific importance across a series of research areas, considerable effort has been devoted to the characterisation of the genetic variation of the colony. Early genetic investigations focused on the development of a genetic map to be used in association studies and the production of reference genome assemblies (Rogers et al. 2000; Cox et al. 2006; Wall et al. 2016). The transition to genome‐based analyses has resulted in a series of investigations defining the genomic profiles of baboons in the colony. High‐coverage whole‐genome sequences were initially obtained for 100 colony individuals, including 33 putatively purebred colony founders (Robinson et al. 2019), generating the first extensive collection of SNVs common in baboons, characterising the colony ancestry composition, and investigating the impact of inbreeding on infant mortality. Lately, more than 800 colony individuals were whole‐genome sequenced (Kendall et al. 2024). Data were used to estimate global and local ancestry, revise genetic maps, and assemble a small set of ancestry‐informative markers.

These genome‐based investigations consistently presented the genomic variation in the baboons of the colony as the result of two main contributors: olive (*P. anubis)* and yellow baboons (*P. cynocephalus*). However, some individuals appeared to deviate significantly from these ancestries. Among these, the most notable was individual 1×0812, identified as a *P. anubis* pure founder of the colony. 1×0812 was the most differentiated from the other investigated founders when its genomic data was analysed using both Principal Components Analysis (PCA) and genome‐wide Identity‐By‐State (IBS) clustering (Robinson et al. 2019). Despite this unusual behaviour, no specific analysis was further implemented to characterise this individual. It is also worth remembering that the founders investigated by Robinson et al. represent only a subset of all the founders, raising the possibility that other unsampled animals might have diverged genomically from the rest of the founders, as 1×0812. More in general, it is not known to what extent these kinds of contributions percolated across the pedigree and influenced the genomic profile of members of the colony.

The focus of this study is to characterise the ancestry composition of the baboons hosted at the SNPRC using previously released genomic data (Robinson et al. 2019; Kendall et al. 2024). We initially focused on the characterization of the mitochondrial DNA variation of the colony founders. We then proceeded to explore the mitogenomic diversity and genomic ancestry profile of all the available samples of the colony. Our main aim was to determine if other, unexpected *Papio* species might have been involved in the current makeup of the colony, without attempting to further resolve within species, population‐specific, contributions. Our results provided evidence for contributions to the SNPRC baboon colony beyond those of *P. anubis* and *P. cynocephalus* usually reported.

## 2 Materials and Methods

### 2.1 Ethics Statement

All analyses reported in this study are performed on publicly available data, hosted on the NCBI Sequence Read Archive (SRA), using samples indicated in Table S1. No new samples were generated through the course of this study. The study adhered to the American Society of Primatologists (ASP) Principles for the Ethical Treatment of Nonhuman Primates.

### 2.2 Data Collection and Data Set Assembly

We collected a series of previously published whole‐genome sequences from baboons from the SNPRC, consisting of a total of 881 samples, 854 of which are available also as whole‐genome fastq files (Kendall et al. 2024; BioProject: PRJNA433868; NIH grant R24 OD017859 (to J. Wall and L. Cox); Lin et al. 2024), also including 33 “pure” founders (8 *P. cynocephalus* and 25 *P. anubis*) investigated by Robinson et al. (2019) (which we refer to as “Robinson founders”). Variants for the 881 samples had been previously called, and genotype data for 6,209,325 SNPs are available (Kendall et al. 2024). We then assembled a set of whole genome sequences representative of the diversity of the genus *Papio* (*Papio* diversity panel, PDP; Table S1). We initially focused on the most diverse collection of whole genome sequences of *Papio* species (Sørensen et al. 2023), from which we selected 2 *P. cynocephalus* from Eastern Tanzania (from Mikumi National Park, which appeared as not admixed in Sørensen et al. 2023), 2 *P. anubis* from Ethiopia, 2 *P. kindae* from Zambia, 2 *P. papio* from Senegal, and 2 *P. ursinus* from Zambia. The *P. cynocephalus* samples from western Tanzania were excluded as reportedly the result of three‐way admixture (Sørensen et al. 2023). We finally added 1 *P. cynocephalus* from Kenya (Wall et al. 2016) and 2 *P. hamadryas* from Ethiopia (Chiou et al. 2022) for a total of 13 genomes (Table S1).

To investigate the phylogenetic placement of the mitochondrial sequences recovered from colony members, we assembled a mitogenome reference data set of 110 publicly available sequences comprising 31 mitogenomes from *P. anubis*, 20 mitogenomes from *P. cynocephalus*, 3 from *P. kindae*, 46 from *P. hamadryas*, 5 from *P. papio*, and 3 from *P. ursinus* (Table S2; Zinner et al. 2009; Finstermeier et al. 2013; Rogers et al. 2019; Roos et al. 2021; Chiou et al. 2022; Sørensen et al. 2023). This data set included the mitogenomes of all of the PDP samples (see above; Table S1). We finally added the mitogenome of *Theropithecus gelada* (NC_019802) as an outgroup (Hodgson et al. 2009) (Table S2).

### 2.3 Genome Alignment and Variant Calling

Since the available genotyped variants for the 881 SNPRC individuals were originally obtained by using the reference genome of *Papio anubis* (Panubis1; Batra et al. 2020), we aligned and called variants for the set of 13 PDP genomes by using the same reference genome. This reference was chosen as it is the same reference used in the original Kendall et al. (2024) work, to minimise possible biases and differences by calling different samples with different reference genomes. *Papio* short reads were mapped with *BWA‐MEM2* v.2.2.1 (Vasimuddin et al. 2019) to the reference assembly genome Panubis1 (Batra et al. 2020). We then marked the duplicate reads with *Picard MarkDuplicates* version 2.8.1 (http://broadinstitute.github.io/picard/) and filtered the result using the option ‐q 10, ‐F 1292, and ‐f 2 of *samtools view* (Danecek et al. 2021).

Autosomal variants were called following GATK's best practices, using GATK v. 4.2.4.1 (McKenna et al. 2010). Individual gVCF files were generated for each sample using *HaplotypeCaller*, and joint calls were made using *GenotypeGVCFs*. The obtained vcf was then filtered using hard filters for SNPs (QD < 2.0 | | MQ < 40.0 | | FS > 60.0 || MQRankSum < −12.5 || ReadPosRankSum < −8.0) with *VariantFiltration*. At this point, we converted the SNPRC vcf file taken from Kendall et al. (2024) and the newly generated *Papio* vcf file and filtered them, removing all sites from sexual chromosomes and keeping only biallelic variants through PLINK 1.9 (Chang et al. 2015). The datasets were then filtered for minor allele frequency (‐‐maf 0.05).

Datasets were converted to EIGENSOFT 8.0.0 (Patterson et al. 2006), and *geno, ind*, and *snp* files and merged using *mergeit*, creating a final data set with 4,023,297 variants, shared between both datasets (Kendall's and PDP). Variants that were not present in either data set were excluded, with a total reported genotyping rate of 0.999 *(*per sample missingness ranging from 0 to 0.0005*)*. The final data set was then converted back to PLINK 1.9 format for data handling.

### 2.4 Mitochondrial Analysis

We generated complete mitochondrial DNA sequences for each individual in the colony data set and in the PDP (if not already available), for a total of 855 mitogenomes (1 PDP, 33 founders, and 821 colony members). Given the storage and computational resources required for handling almost 1000 WGSs as well as the focus on the mitochondrial DNA, we assembled the mitogenomes by randomly subsampling reads from individuals’ sequencing runs using NCBI SRA‐Toolkit's fastq‐dump (https://trace.ncbi.nlm.nih.gov/Traces/sra/sra.cgi?view=software). We initially assembled the mitogenomes of the 33 Robinson founders, comparing different subsample sizes (5, 10, 20, and 25 million reads) to the mitogenomes assembled using all of the reads available for each individual, and evaluated median mitogenome depth as well as phylogenetic signals obtained for each subsampling. 15 million reads provided at least 50X median depth and robust phylogenetic association, similar to that obtained by a higher amount of reads (Table S3; Figure S1). We therefore proceeded and downloaded 15 million reads from each of the 855 available sequences (founder and non‐founder colony members plus DPD individuals whose mitogenome was not already available; Table S2). We then mapped the subsampled individuals to the reference sequence for *Papio anubis* (Panubis1; Batra et al. 2020), which also contains the reference mitochondrial DNA sequence for *P. anubis* (NC_20006.2; Zinner et al. 2013). This reference belongs to a sample of *P. anubis* from Ethiopia, and the associated mitochondrial DNA is phylogenetically assigned to cluster G of the *Papio* mitochondrial phylogeny (Roos et al. 2021; Sørensen et al. 2023). Reads that mapped to the mitochondrial sequence were extracted using *mpileup* from *bcftools* v.1.19 (Danecek et al. 2021), generating a consensus sequence that was then filtered and converted from fastq format to fasta through Seqtk (https://github.com/lh3/seqtk) using default settings. The obtained mitogenomes were then aligned to the ones generated in previous studies (Table S2) using MAFFT v.7.5.20 (Katoh and Standley 2013) with the –auto flag, using *Theropithecus gelada* (NC_019802) as an outgroup. TrimAl v.1.4. rev.15 (Capella‐Gutiérrez et al. 2009) with the –gappyout flag was used to remove poorly aligned regions. The d‐loop was also manually removed from the alignment. Sequences that exceeded 16% in gaps or ambiguous bases were excluded (Table S2). Phylogenetic reconstruction was performed using IQ‐TREE v.2.2.6 (Minh et al. 2020), using *ModelFinder* to detect the most fitting substitution model and performing 1000 rounds of ultrafast bootstrap to determine branch support. To test for the possibility of reference bias in the mitochondrial assembly, we reassembled the mitogenome of the 33 founders identified by Robinson et al. (2019) by using different references: JX946199.2, who belongs to *P. cynocephalus* North and is phylogenetically located within clade G, JX946198.2, who belongs to *P. anubis* from Nigeria and is part of clade F, and to JX946200.2 from a southern *P. cynocephalus* placed within clade B (Roos et al. 2021). Phylogenetic analysis of the newly assembled sets of mitochondrial genomes was performed as described above. All the mitogenomes from the SNPRC colony that showed a phylogenetic affinity different from *P. anubis*/*P. cynocephalus* were reassembled using the whole set of genomic reads, and the phylogenetic analysis of these samples was then performed as described above.

### 2.5 Ancestry Estimation via ADMIXTURE

We used the merged nuclear data set (894 colony members, including founders and non‐founders, plus the PDP samples) to perform a series of analyses on the genomic makeup of the colony, starting with exploratory analyses such as ADMIXTURE. We run ADMIXTURE 1.3, an ancestry estimator based on genotype data for autosomal SNPs (Alexander and Lange 2011), on a set of individuals comprising the PDP and all of the 33 founders from Robinson et al. (2019). We decided to use this set of individuals as a baseline for exploring the diversity inside of the colony. We note that the 881‐sample data set generated by Kendall et al. (2024) includes 18 additional samples that are reported as founders, which we analysed with the other samples from the same study (Table S1). We tested for K between 2 and 10, using the –cv flag to calculate the associated cross validation error (CV) and determine the most fitting K. We then projected the remaining colony members on the population structure detected, using the allele frequencies for *K* = 5–7 to distinguish components associated to single species (*K* = 5 for *P. anubis, P. cynocephalus, P. hamadryas*, and *P. papio*; K = 7 for *P. kindae* and *P. ursinus*).

### 2.6 PCA and Ancestry Estimation via PANE

The same set of samples used to perform the initial ADMIXTURE analysis was used to explore the colony ancestry composition through Principal Component Analysis (PCA). We initially ran PCA through EIGENSOFT's *smartpca* (Patterson et al. 2006). Similarly to ADMIXTURE analyses, we only used the PDP plus the founder samples present in Robinson et al. (2019) to calculate the eigenvectors, generating 40 PCs to be used as basis for PANE ancestry estimation (de Gennaro et al. 2025) for the remaining colony members. PANE was selected for its ability to efficiently determine sample ancestry from a given set of sources in both ancient and modern samples (de Gennaro et al. 2025). Based on our ADMIXTURE results, we selected as sources for our PANE analysis samples characterised by a single ancestry for any K between 2 and 7 (Figure S3). In doing so, we excluded the two *P. anubis* and the 3 *P. cynocephalus* from the PDP. Among the founders, a total of 3 *P. cynocephalus* and 5 *P. anubis* consistently showed a single ancestry. From these, we randomly selected two individuals from each species, in line with the number of individuals representing other *Papio* sources (2 *Papio hamadryas*, 2 *P. papio*, 2 *P. ursinus*, 2 *P. kindae*; Table S1). We used a final set of 12 samples to estimate ancestries of the 848 colony members not included in the Robinson founders (“non‐Robinson” samples) with PANE, running several analyses each considering a different number of PCs (3, 5, 6, 10, 15, 20, 25, 30, 35, and 40), weighting each PC by its explained variance (de Gennaro et al. 2025), and testing the correlation between each ancestry estimates (R^2^ and its FDR‐corrected associated p‐value; Figure S4).

### 2.7 Kinship Analysis

We explored relationships within the colony through the software KING 2.3.2 (Manichaikul et al. 2010), using available genotyped variants. Relationship inferences were performed with the *related* flag, up to the 4th degree (‐‐degree 4). Kinship analysis was performed on different subsets of our complete data set, to detect direct relationships but avoid the identification of kinship due to shared descendants. In this context, kinship was initially searched among the set of 33 founders. We then searched for related individuals among samples identified by either ADMIXTURE or PANE as deriving a fraction of 0.10 of their ancestry from a source different from *P. anubis* or *P. cynocephalus*. Finally, we searched for relatedness in a data set extending the previous set by the addition of individuals bearing *P. hamadryas* mitochondrial sequences, if not already included.

### 2.8 Validation of Ancestry Estimates

#### 2.8.1 Datasets Assembly and Variant Calling

To explore potential biases in ancestry identification related to the use of SNV identified using different pipelines and the implementation of haplotype‐based methodologies, we assembled and analysed two additional datasets, in part overlapping with the one analysed above. The first data set, labelled Set A, was specifically designed to standardize variant calling across samples and to include a more balanced representation of colony and non‐colony sources. Set A included a total of 61 individuals, including 25 non‐colony reference samples and 36 colony samples (Table S4). Specifically, we included in the 36 colony samples four high‐depth (> 10×) *P. anubis* and four high‐depth *P. cynocephalus* individuals, each with ≥ 0.99 ancestry estimates for the assigned species (Table S4). These individuals correspond to the “pure” founders described by Kendall et al. (2024), who also used them as references in their RFMix analyses. We additionally included founder 1 × 0812, which carries *P. hamadryas*‐associated mitochondrial haplotype, and all 19 samples with more than 0.1 ancestry fraction from unexpected species as identified by either PANE or ADMIXTURE analyses (both high and low depth samples; low depth < 10×; see Results; Table S4). To explore the possible impact on ancestry estimation of using low‐depth samples, we finally selected four “pure” low‐depth *P. anubis* SNPRC samples and four low‐depth *P. anubis* x *P. cynocephalus* SNPRC hybrids with high *P. cynocephalus* ancestry, chosen based on the most informative ADMIXTURE runs (see results; *K* = 5–7) (Table S4). For the 25 non‐colony samples, we included 4 samples for each of the six *Papio* species, 13 of which were included in the original complete data set (PDP samples), and two additional samples per *Papio* species were added from Sørensen et al. (2023) (Table S4). All reads were aligned following the pipeline and references used for the PDP samples. Genotypes were firstly called for high‐depth samples (Table S4) using GATK's best practices, applying HaplotypeCaller and GenotypeGVCFs, followed by variant quality filtering. Sex chromosomes were excluded, and variants were filtered in PLINK for minor allele frequency (‐‐maf 0.05), missing genotypes (‐‐geno 0.05) and linkage disequilibrium (‐‐indep‐pairwise 50 5 0.5). We obtained a total of 5,560,572 SNPs with a genotyping rate of 0.996. We used these identified variants from high‐depth samples to call low‐depth samples using ANGSD v. 0.940 (Korneliussen et al. 2014), following a routinely used approach to analyse together low and high depth samples (Ravasini et al. 2024). Briefly, we generated pseudohaploid individuals from the. bam files, calling only sites present in the high‐depth Set A variant panel. The resulting. haplo file was then converted to PLINK and merged with the high‐depth panel, keeping only biallelic variants. The final merged Set A contained 5,560,572 SNPs, with a total genotyping rate of 0.988, and was balanced between colony (*n* = 36) and non‐colony individuals (*n* = 25) (Table S4).

To evaluate any variation in ancestry inference due to the use of non‐haplotype‐based methodology, we assembled a data set focussed on high‐depth samples only, to make the implementation of RFMIX v.2.03 feasible, a haplotype‐based ancestry estimator (Maples et al. 2013). This data set, named Set B, was assembled to investigate the contribution of *P. hamadryas* ancestry, the third most common ancestry component detected in the colony. Set B was assembled by selecting 10 high‐depth colony samples: the three individuals with more than 0.1 *P. hamadryas* ancestry fraction (1 × 0812, 1 × 0356, and 1 × 2124; Table S4), plus seven high‐depth *P. anubis x P. cynocephalus* hybrids for whom no *P. hamadryas* ancestry was detected in our analysis of the whole colony, as control (Table S4). Reference haplotypes were recovered from a set of high‐depth, whole genome sequences we used as sources. This set included samples from *P. anubis*, *P. cynocephalus*, and *P. hamadryas* originally included in Set A, which was expanded to reach 10 high‐depth individuals per species using already published data (Table S4; Sørensen et al. 2023). We specifically excluded *P. anubis* from Ethiopia due to their known admixture with local *P. hamadryas* populations (Sørensen et al. 2023), to minimize conflicting signals in the reference haplotype panel. Although some of the reference samples show varying degrees of *P. anubis x P. cynocephalus* admixture (Sørensen et al. 2023), we prioritized individuals from localities with the lowest reported hybridization levels to reduce biases in ancestry estimations. However, we note that the presence of these hybrids is not expected to impact the identification of *P. hamadryas* ancestry. The final set included 40 individuals: 30 reference non‐colony samples and 10 colony samples (Table S4). Samples were aligned and called following the same pipeline as for the high‐depth Set A sequences, and variants were filtered in PLINK 1.9 for minor allele frequency and missing genotypes (‐‐maf 0.05 –geno 0.05), to allow haplotype reconstruction and local ancestry. The final data set included 13,270,964 variants with a genotyping rate of 0.997. For PCA and ADMIXTURE analyses, variants in Set B were further pruned for linkage disequilibrium (‐‐indep‐pairwise 50 5 0.5), obtaining a total of 2,512,958 variants with a genotyping rate of 0.995.

#### 2.8.2 Analyses

We repeated ADMIXTURE and PANE analyses on Set A and Set B, as described above. Briefly, for Set A, we first ran admixture for *K* = 5–7 on the selected source individuals (the 25 non‐colony samples and 9 high‐depth founder samples; Table S4) and then used the obtained frequencies to run a projected ADMIXTURE on the remaining colony members. For PANE, PCA was performed, calculating the eigenvector on the same source samples used for ADMIXTURE and projecting the remaining samples (Table S4), for which ancestry estimates were calculated using the first 6 PCs. We analysed Set B for ADMIXTURE and PANE in the same way as Set A, adjusting for the smaller number of tested ancestries and the high‐depth of all samples in the data set. We performed ADMIXTURE for *K* = 2–5 and PCA using all samples in set B. We then used the non‐colony samples as sources for the PANE analysis, targeting the 10 colony individuals. We referred to the first 3 PCs, mirroring the number of sources used, as we did for both the original complete data set and Set A (see results).

For the local‐ancestry analysis, the vcf of the Set B variants was firstly phased with Beagle v. 5.4 (Browning et al. 2021), using the updated *Panubis1* genomic map generated by Kendall et al. (2024) with default settings. Beagle was chosen due to its accuracy in human and nonhuman populations (Oget‐Ebrad et al. 2022). RFMIX v.2.03 (Maples et al. 2013) was used to determine the local ancestry of target samples based on source haplotypes, using default settings. Karyogram plots for each target sample were generated using haptools 0.5.0 (Massarat et al. 2023), following conversion of the generated RFMix output. Tractor v.1.4.0 (Atkinson et al. 2021) was used to generate per‐sample global ancestry estimates from the obtained RFMix local ancestry output.

Ancestry estimates of each set were compared for the same samples across datasets. We tested for the correlation of the obtained ancestry estimates across analyses using the corr() function in R with Pearson's correlation coefficient.

## 3 Results

### 3.1 Phylogenetic Analysis of SNPRC Mitogenomes

**Figure 1:**
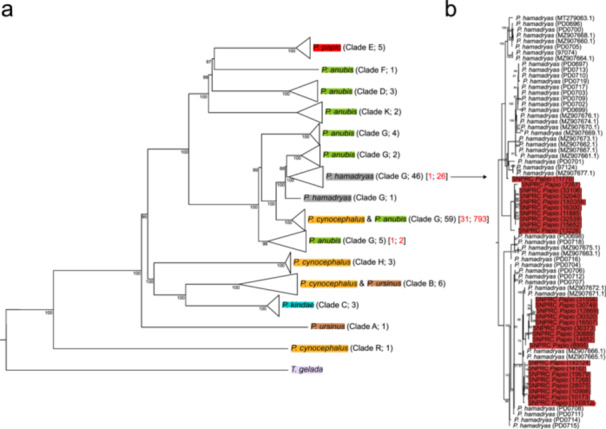
Phylogenetic analysis of SNPRC baboon mitogenomes. (a) Collapsed Papio mitochondrial DNA phylogeny with SNPRC samples. On each tip, the mitochondrial clade (Sørensen et al. 2023) and the number of individuals from the reference data set are reported in brackets. The numbers of SNPRC mitochondrial sequences of founders and non‐founders are reported between square brackets. (b) Phylogenetic analysis of SNPRC founders associated with the *P. hamadryas* group within mitochondrial clade G. SNPRC samples are highlighted in red, while the founder 1 × 0812 is indicated by a star. Ultrafast bootstrap values are reported near each node. The arrow refers to the position of the cluster in panel b within the phylogeny in panel (a).

We initially investigate the nature of the unexpected behaviour of the 1 × 0812 *P. anubis* founder by characterising the mitochondrial variation present in the 33 baboon founders investigated in Robinson et al. (2019). We generated the mitochondrial sequence of all the founders by mapping reads to the mitochondrial reference genome present in the *Papio* reference genome assembly (Panubis1; Batra et al. 2020) and extracting a consensus whole mitogenome sequence, with a minimum median alignment depth of 50X. A total of ~15,400 bp were assembled for each mitogenome once the d‐loop region was removed (~1000 bp). Gaps and ambiguous bases represented no more than 16% in each of the assembled mitogenomes. We then generated a mitogenome phylogenetic tree including samples from all major mitochondrial clades of *Papio* (Table S2; Zinner et al. 2009; Rogers et al. 2019; Roos et al. 2021; Chiou et al. 2022; Sørensen et al. 2023), adding to the analysis the mitogenomes assembled from the founders' genomes (Figure 1a). All of the founders' mitogenomes were assigned to the previously reported cluster G (Zinner et al. 2009; Roos et al. 2021; Sørensen et al. 2023). Of these, all except one (1X0812) were assigned to a subcluster comprising *P. anubis* and *P. cynocephalus* mitogenomes recovered from individuals from Eastern Africa (Kenya, Tanzania, and Ethiopia; Figure 1a; Table S2; Figures S1, S2). This was also the case for the mitogenome recovered from sample 1X4384, a *P. anubis* founder whose genomic variation placed it close to, but not overlapping with, *P. cynocephalus* founders, and 1X3576, one *P. anubis* baboon mislabeled as *P. cynocephalus* (samples 3 and 1 in Figure 2 of Robinson et al. 2019). The mitogenome of 1X0812, which in the genome‐based analysis of Robinson et al diverged from all of the other baboon founders (sample 2 in Figure 2 of Robinson et al. 2019), did not cluster with the mitogenomes of other founders and was assigned to a cluster of *P. hamadryas* mitogenomes within clade G (Figure 1a). The phylogenetic placement of the mitochondrial sequences of the Robinson founders (including 1X0812) did not show any major difference when their mitogenomes were assembled by mapping the genomic reads to other mitogenomes sampled across the *Papio* phylogeny or when assembled using the whole set of genomic reads (Figures S1, S2).

**Figure 2:**
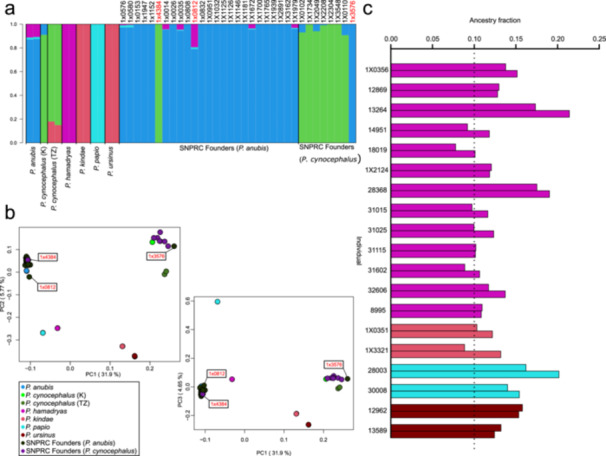
Genomic variation of the SNPRC colony. (a) ADMIXTURE results for *K* = 5 for PDP and SNPRC founders. Species/population of each sample is shown at the bottom; ID of SNPRC founders is shown on top of each corresponding bar. (b) PCA plots for the first three PCs, as PC1 vs PC2 and PC1 vs PC3 (data set as in panel (a). The variance explained by each component is reported near the relevant axis. (c) Non‐Robison colony samples with at least 0.10 ancestry fraction from *Papio* species other than *P. anubis* or *P. cynocephalus* as identified by ADMIXTURE and PANE (see main text). For each individual, the top and bottom bars report the ADMIXTURE and PANE estimates, respectively. Colours of the bars refer to the minor ancestry, as in panel b. SNPRC founders are labelled as *P. anubis* or *P. cynocephalus* according to Robinson et al. (2019). ID of the three outlier founders identified in Robinson et al. (2019) are in red. K, Kenya; TZ, Tanzania.

Considering these results, we proceeded to assemble the mitogenomes of the available colony baboons. Sequences that exceeded 16% in gaps or ambiguous bases were excluded, leaving 812 assembled mitogenomes. Each assembled mitochondrial sequence was run independently to avoid possible batch bias in phylogenetic placement. The vast majority of the colony mitogenomes were associated with the clade of cluster G that groups *P. anubis* and *P. cynocephalus* sequences (*n* = 786, 97%; Figure 1a). However, a relatively small subset of mitogenomes did not cluster with *P. cynocephalus* or *P. anubis* sequences but instead clustered with the *P. hamadryas* mitogenome clade present within cluster G (*n* = 26, 3%) (Figure 1). These sequences are grouped in different parts of the *P. hamadryas* G clade, representing at least four different clusters, indicating the presence in the colony of different lineages of *P. hamadryas* (Figure 1b).

We tested the robustness of the recovered *P. hamadryas* mitogenomes by reassembling the whole mitochondrial DNA of the samples bearing putative *P. hamadryas* mtDNA sequences using the whole set of available genomic reads (average number of reads per sample: 178 million; range 52–790). The mean of the median depth of these reassembled mitochondrial genomes was 1681x (range 218×–7901×). The phylogenetic analysis of these samples was then repeated as described above. Reassembled mitogenomes were assigned to the same *P. hamadryas* subclade within clade G as before (Figure S5).

### 3.2 SNPRC Founders and Papio Genomic Variation

Guided by the unexpected identification of *P. hamadryas* mitogenomes in the SNPRC colony, we explored how the genomic variation present in the colony compared to the diversity present within the genus *Papio*, by initially focusing on the 33 colony founders.

We first explored the genomic diversity of the 33 founders using ADMIXTURE (Alexander and Lange 2011). We analysed a set of samples assembled to represent *Papio* variation (PDP), to which we added all of the colony founders from Robinson et al. (2019). We ran the analysis for K ranging between 2 and 10, with 2 resulting in the most supported number of clusters (Figure S3). When larger numbers of K are considered, ancestries start to be specifically associated with a given species (first *P. hamadryas* and *P. anubis*, followed by *P. cynocephalus*, then *P. papio*; Figure S3). For *K* = 5 all the baboon species, except for *P. ursinus* and *P. kindae*, are characterised by a single ancestry. At this level *P. anubis* samples from Ethiopia are characterised by the presence of *P. hamadryas* ancestry, *P. cynocephalus* from Tanzania East include *P. ursinus/P. kindae* ancestry and *P. cynocephalus* from Kenya include *P. anubis* ancestry, as previously reported (Sørensen et al. 2023). Across the founders, none except one presented other ancestries than *P. cynocephalus* and *P. anubis* below 0.05: the founder 1X0812 showed a genomic fraction of *P. hamadryas* equal to 0.19, the rest being represented by *P. anubis* ancestry. For *K* = 6 *P. ursinus* and *P. kindae* are still defined by the same ancestry, *P. hamadryas* and *P. papio* samples become characterised by the same single ancestry, and three ancestries appear associated with *P. anubis*. Interestingly, one of the three *P. anubis* ancestries is the only ancestry present in samples comprising at *K* = 5 both *P. anubis* and *P. hamadryas‐P. papio* ancestries. This is the case for the founder 1X0812 and for the two *P. anubis* from Gog in Ethiopia. The other two ancestries found associated with *P. anubis* at *K* = 6 are either the only ancestry present in the sample or are paired either with the one present in 1 × 0812/*P. anubis* from Ethiopia or with each other (Figure S3; Table S5). Overall, for *K* = 6, the ancestry contributions of *P. cynocephalus* and *P. ursinus/P. kindae* is the same as that estimated for *K* = 5. This is also the case for *P. anubis*, except for those samples where *P. hamadryas‐P. papio* ancestries were detected at *K* = 5, as these ancestries “merged” with *P. anubis*, generating the new component that fully described samples such as 1×0812 and the Ethiopian *P. anubis*. The same pattern is present at *K* = 7, when the *P. ursinus/P. kindae* ancestries separate. Following these observations, we decided to focus on the ancestry assignment defined by *K* = 5 as being the one still retaining the power to detect specific, species‐level ancestry contributions (Figure 2a; Figure S3).

We then explored the ancestry composition of the colony members by Principal Component analysis. As for the ADMIXTURE analysis, we initially ran the PCA on the PDP and Robinson founders only (Figure 2b; Media S1). Northern and Southern baboons were divided into two clades along the first principal component (PC1), which explains almost a third of the overall diversity, while species variation within the two Papio clades was highlighted by PC2 (Rogers et al. 2019; Sørensen et al. 2023). The inclusion of PC3 further separated *Papio papio* (on one side) and *P. ursinus/P. kindae* (on the other) from the other *Papio* species. The Robinson founders are plotted in two clouds, one centred around *P. cynocephalus* from Kenya (including 1 × 4384), the other close to (but not overlapping with) *P. anubis* from Ethiopia. Sample 1 × 0812 is the *P. anubis* founder plotting the most away from the other founders along the PC2, placed towards *P. hamadryas* samples.

### 3.3 SNPRC Genomic Ancestry Composition

We investigated the genome assignment to different ancestries across the whole colony using ADMIXTURE and PANE, two ancestry estimation software (Alexander and Lange 2011; de Gennaro et al. 2025).

We first used the results of the ADMIXTURE analysis run on the PDP and founders for K = 5. We reconstructed the ancestry composition of all of the colony members, excluding the founders, by using the allele frequencies of the 5 identified ancestries, using ADMIXTURE projection analysis (Alexander and Lange 2011). *P. anubis and P. cynocephalus* represented on average the most common ancestries present in the colony, with mean fractions of 0.81 (SD 0.14) and 0.14 (SD 0.16) (Table S6). Together they comprised no less than 0.74 of the genome in the 848 non‐Robinson colony members. Other colony members also presented ancestries associated with *P. hamadryas*, *P. papio*, and *P. kindae/P. ursinus*, with average values of 0.03 (S.D. 0.02), 0.02 (0.01), and 0.01 (0.01). These three minority ancestries were differently distributed across samples. More than 60 samples had in their genome a fraction of *P. hamadryas* ancestry above 0.05 (range 0.05‐0.16), while it was above 0.10 in 8 samples. On the contrary, *P. kindae/P. ursinus* and *P. papio* ancestries were above 0.05 only in 8 (range 0.05–0.19; 3 samples above 0.10) and 3 samples (0.08, 0.14, and 0.16), respectively. *P. kindae* and *P. ursinus* specific contributions were evaluated by projecting the non‐Robinson colony members using the result for *K* = 7. Four of the eight samples presented *P. kindae* or *P. ursinus* genomic fractions above 0.05 (0.10, 0.09, and 0.16, 0.13, respectively), while the remaining four had ancestry values between 0.02 and 0.03 (Table S6). The sum of these two ancestries (*P. kindae* and *P. ursinus*) for *K* = 7 was similar to their combined contribution as estimated for *K* = 5.

We then explored the ancestry composition of all of the non‐Robinson members of the colony using PANE, a genome‐wide ancestry estimator based on PCA (de Gennaro et al. 2025). As for ADMIXTURE, we first analysed the PDP genomes and 33 founders together and generated 40 PCs to be used for the NNLS approach implemented in PANE, selecting 2 individuals per species to be used as sources to estimate ancestry of the 848 non‐Robinson members (Table S1). We applied PANE taking into consideration varying numbers of PCs and observed that ancestry estimates for *P. cynocephalus* and *P. anubis* were highly correlated across all runs (R^2^ > 0.99; *p* < 0.001; Figure S4). However, correlations for minor ancestries were lower, especially when 3 and 5 PCs were considered, though generally still significant (Figure S4). To further evaluate the consistency of PANE results, we compared the number of colony members with at least a fraction equal to 0.05 or 0.10 for a given ancestry across different sets of PCs (Table S7). The number of individuals identified with *P. ursinus* and *P. kindae* ancestries remained virtually identical when an increasing number of PCs was taken into consideration. For *P. papio* and *P. hamadryas*, the number of individuals with 0.10 ancestry fraction remained stable until more than 30 PCs were analysed, while the counts for > 0.05 ancestry fraction increased with increasing PCs. To avoid inflating weaker signals, we chose to focus only on individuals with > 0.10 fraction of a given ancestry, while also deeming estimates using higher PCs as being overfitted for smaller fractions (Table S7). This threshold was also chosen because the maximum ancestry assignment error encountered using PANE in simulated modern low‐missingness samples was below 0.1 (de Gennaro et al. 2025). Considering that six source species were taken into consideration, and that results were consistent when considering a larger number of PCs, we focused on PANE results generated using 6 PCs, which together explained 51% of the total variance (Table S7). Similarly to ADMIXTURE results, *P. cynocephalus* and *P. anubis* were the most common ancestries identified by PANE in the colony, together representing a genomic fraction no smaller than 0.67 in any of the individuals (mean 0.94, SD 0.03; Table S8). The mean value of the fraction of genomic ancestry being *P. anubis* or *P. cynocephalus* was 0.80 (SD 0.17) and 0.14 (SD 0.18), respectively. Only a small number of colony members were represented by a single ancestry (> 0.99): 19 for *P. anubis* and 1 for *P. cynocephalus*. Two samples had less than 0.10 of *P. anubis* ancestry, but 353 samples hosted less than 0.10 *P. cynocephalus* ancestry.

Minor ancestries accounted for a combined average of 0.06 (SD 0.03), led by *P. hamadryas* (0.03; SD 0.02) and *P. papio* (0.03; SD 0.01), while *P. kindae* and *P. ursinus* represented on average a genomic fraction smaller than 0.002 (SD 0.01). Thirteen individuals had a genomic fraction larger than 0.10 for *P. hamadryas*, and two individuals each had > 0.10 fraction for *P. papio, P. kindae*, or *P. ursinus* (largest estimated fractions: 0.21, 0.20, 0.12, 0.15, respectively). Samples with a genomic fraction larger than 0.10 for any of the minor ancestries (*P. hamadryas*, *P. papio, P. kindae*, and *P. ursinus*) as identified by either ADMIXTURE or PANE are reported in Figure 2c (Tables S5, S7). ADMIXTURE and PANE largely identified the same non‐Robinson colony samples. Differences were present for 5 samples (4 for *P. hamadryas* and 1 for *P. kindae*) due to ADMIXTURE estimates being below the 0.10 threshold (Figure 2c). We note that both the samples showing > 0.10 fraction for *P. kindae* using PANE were identified as colony founders by Kendall et al. (2024).

*P. anubis* and *P. cynocephalus* ancestry estimations by PANE and ADMIXTURE are significantly highly correlated (R^2^: 0.992 and 0.996; Media S2, S3), and they are also significantly correlated with the results of the supervised ADMIXTURE analysis presented in Kendall et al. (2024) (Table S3 In Kendall et al. (2024); Media S2, S3). Our mean ancestry estimates for *P. anubis* are similar to the mean estimated using Kendall et al. ADMIXTURE results, but *P. cynocephalus* ancestry estimates are smaller than Kendall's (0.80 for *P. anubis*; 0.21 for *P. cynocephalus*).

We finally focused on samples having a minority ancestry above 0.10 (Figure 2c), and searched for their relationships with members of the colony using the software KING (Manichaikul et al. 2010). The 6 samples with *P. kindae, P. ursinus*, and *P. papio* were either related to each other (1 × 0351‐1 × 3321, second degree) or had no relatives (≦4th degree) in the colony. On the other hand, the 13 samples with high *P. hamadryas* ancestry show a large number of relatives inside the colony (Table S9). When not considering other members of the colony, however, the only close relationship among these 13 samples is the one between individuals 14951 and 18019 (4th degree; Table S10). Four of the 26 colony members with *P. hamadryas* mitogenomes had more than 0.10 *P. hamadryas* ancestry, as estimated by both ADMIXTURE and PANE. The remaining 22 have estimates of *P. hamadryas* ancestry between 0.1 and 0.02, identified by either PANE or ADMIXTURE. KING analysis on all individuals with 0.10 *P. hamadryas* ancestry and/or *P. hamadryas* mitochondrial‐associated sequence (36 individuals) detects two kinship groups (of 5 and 7 individuals, respectively), whose members also cluster together in mitochondrial analyses (Table S10).

### 3.4 Validation of Identified Ancestries

Our initial results on the whole colony were obtained by merging the SNV data set generated by Kendall et al. (2024) with SNVs newly called by us by assembling a reference panel of Papio species. For this reason we explored if merging SNVs datasets generated using different pipelines might significantly impact ancestry detection. In doing so we assembled a new set of sources doubling up the number of individuals for each source (from 2 to 4), to which we added a selection of samples including all the samples with unexpected ancestry (Figure 2c) and several control individuals (of both high and low depth) to be used as targets to validate the robustness of the used pipeline. The SNVs of this newly assembled data set (Set A) were obtained by applying a standard pipeline to analyse together high and low depth samples. We processed the sources and the targets via ADMIXTURE and PANE using the same parameters applied for the analysis of the 881 colony individuals described above (“original complete data set”). Pairwise comparisons of ancestry estimates of the same individuals analysed as part of the original complete data set and as part of Set A highlighted a strong correlation (Table S11; Figure S6) for the results of both ADMIXTURE (*r* = 0.933, *p* < 0.0001) and PANE (*r* = 0.954, *p* < 0.0001), with average difference in individual ancestry estimates across datasets and analyses being below 0.05 (Table S11; Figure S6).

Kendall et al. (2024) originally characterised the colony ancestry composition using RFMIX, an haplotype‐based ancestry estimator. As we used non‐haplotype‐based approaches, we explored to what extent these approaches might differ in ancestry identification when applied to the same data set. An additional data set (Set B) was specifically assembled including only high‐depth genome data. We focussed on the most common unexpected ancestry (*P. hamadryas*), and included 10 individuals per species as representative of three different sources (*P. anubis*, *P. cynocephalus* and *P. hamadryas*). We then selected high‐depth genomes from the colony as targets. We included colony individuals previously identified as hosting *P. hamadryas* ancestries (*n* = 3), and, as control, we also included individuals with virtually no *P. hamadryas* ancestry (*n* = 7). We used this data set to perform ADMIXTURE and PANE, and also obtained genome‐wide ancestry estimates using RFMIX v.2.03 (Maples et al. 2013). In the ADMIXTURE analysis the most supported number of Ks for Set B was 3, which assigned each ancestry to a single source species. We therefore considered ancestry estimates for Set B considering the results obtained for *K* = 3.

Correlation between ancestry estimates were strong between samples analysed as part of both the original complete data set and Set B (ADMIXTURE *r* = 0.959, *p* < 0.0001; PANE *r* = 0.982, *p* < 0.0001), although the small number of samples analysed limits the power of the statistical inference (Table S11; Figure S6). The average difference in individual ancestry estimates was below 0.05 (Table S11; Figure S6).

Global ancestry estimates derived from RFMIX outputs were obtained for all colony samples in Set B. Two founder members (1 × 0812 and 1 × 0356) with *P. hamadryas* ancestry estimates in the original data set of 0.14–0.15 (1 × 0356) and 0.19 (1 × 0812) were confirmed with similar *P. hamadryas* ancestry proportions (0.11 for 1 × 0356 and 0.18 for 1×0812; Table S11). The third sample, 1 × 2124, which had a *P. hamadryas* ancestry of 0.12 in the original estimate, had a lower *P. hamadryas* ancestry estimate of 0.06 using RFMIX (Table S11). The rest of the colony members, chosen as control with no detected *P. hamadryas* ancestry, were all estimated by RFMIX to have on average < 0.01 *P. hamadryas* ancestry (Range 0.003–0.02; Table S11).

Finally, we used the obtained local ancestry runs by RFMIX to generate karyogram plots for the three individuals with discernible *P. hamadryas* ancestry, providing a more comprehensive description of chromosome‐level hybridization patterns (Figure S7).

## 4 Discussion

### 4.1 Complex Genomic Ancestry Profiles in SNPRC Baboons

SNPRC baboons have been recently characterised for their genomic composition. In line with previous investigations, we detected two main sources in the colony: *P. anubis* and *P. cynocephalus* (Robinson et al. 2019; Kendall et al. 2024). However, previous results hinted at the presence of some individuals that did not conform to the diversity displayed by most of the colony founders (Robinson et al. 2019). We initially pursued this evidence by performing a phylogenetic analysis of mitochondrial DNAs present in the colony. While all samples were phylogenetically placed in the North‐Eastern mitochondrial clade G, not all sequences were associated with either of the expected *Papio* sources, as a few mitogenomes were grouped with *P. hamadryas* mitochondrial lineages. The founder 1 × 0812, previously shown to deviate from the genomic profile of other colony founders (Robinson et al. 2019), was among the samples bearing a *P. hamadryas* mitochondrial DNA. The occurrence of these small fractions (3%) of unexpected mitochondrial lineages hinted at the possibility of the occurrence of *P. hamadryas* ancestry across the colony, also at the nuclear level.

We therefore explored the hypothesis of a complex ancestry profile for the members of the pedigreed SNPRC baboon colony by implementing ancestry estimation analyses that encompassed all six species of *Papio* as putative sources. *P. anubis* and *P. cynocephalus* were confirmed as the main sources of genetic variation in the colony, all the animals showing no less than 67% of any of the two ancestries. However, additional ancestries were also identified. *P. hamadryas* ancestry was detected in a relatively small set of samples (1.5%), in line with the mitochondrial results. *P. hamadryas* ancestry possibly originated from multiple individuals, as suggested by the occurrence of at least four different groups of mitochondrial sequences. Other contributions were present too, even if in a more sporadic way. *P. papio*, *P. kindae*, and *P. ursinus* ancestries were detected in a small number of individuals, but no mitochondrial DNA associated with these species was detected. The lack of mitochondrial lineages in the presence of limited nuclear ancestry contributions can be easily explained by breeding events interrupting the uniparental female transmission of the mitochondrial DNA and/or a male‐mediated initial introgression. In total, we detected 42 individuals with signals of unexpected ancestries, with at least 0.10 ancestry fractions and/or mitochondrial sequence placement, almost 5% of the analysed SNPRC individuals. We also note that some of the individuals found with unexpected ancestries are also reported to be colony founders by Kendall et al. (2024), but were not included in the original complete data set by Robinson et al. (2019). Future analysis of the variation of the Y chromosome will also clarify the role, if any, of male‐mediated introgression (Mutti et al. 2023).

We validated our initial generated ancestry estimates obtained using the original complete data set by repeating PANE and ADMIXTURE analyses with samples processed jointly and with a larger number of source individuals, as well as applying a haplotype based approach. Results were largely consistent in detecting (or not) unexpected ancestries, with some degree of variation observed in the estimated absolute amounts. Overall, ancestries detected by one approach were identified also by others, with a large degree of consistency in their relative amounts (average estimate difference below 0.05; Table S11; Figure S7). The observed consistency in the results highlights PANE's aptitude for detecting ancestry fractions in both modern and ancient samples. While not directly comparable to haplotype‐based methods, other programmes employing NNLS optimization on PCA spaces to determine ancestry components (such as RYE; Conley et al. 2023) have been proven to provide highly correlated results to RFMix and ADMIXTURE (Conley et al. 2023), and PANE has been shown to provide highly correlated results to these programmes (de Gennaro et al. 2025).

We note here that our ancestry estimations should be considered as indicative of the presence of additional ancestries and not conclusive in the amount and in the total number of individuals hosting them, as suggested by the variation in the number of individuals with unexpected ancestry fractions between 0.05 and 0.10 highlighted by analyses and validations. Moreover, the vast majority of the samples analysed by Kendall et al. (2024) were imputed low‐coverage whole‐genome sequences. The authors employed different pipelines and filtering steps to ensure optimal results, but it must be noted that the accuracy of these imputed calls has not been verified. It is important to note that Kendall et al. (2024) used pure *P. cynocephalus* and *P. anubis* samples to impute their low‐depth samples, and, nevertheless, we were able to find unexpected other ancestries in some of the individuals in this data set, which we confirmed using a different approach to analyse low‐depth samples. Further tests, including a more extensive representation of potential sources and/or a higher number of high‐depth genomes, might provide a more refined and detailed description of the ancestry profile of the colony.

### 4.2 The Origin of “Unexpected” *Papio* Ancestries

We can speculate on the origin of the observed unexpected baboon ancestries based on the history of the presence of baboons at the Texas colony, the distribution of these ancestries across the colony, and the genomic features of the founders.

Given the relatively widespread occurrence of *P. hamadryas* across the colony and the presence of multiple *P. hamadryas* mitochondrial lineages, it is possible to suggest that the origin of this ancestry is associated with multiple source individuals. Two not mutually exclusive scenarios can be proposed to explain this pattern. One assumes events of admixture involving *P. hamadryas* individuals at the time/after the foundation of the baboon colony since 1958. The other envisages the occurrence of already mixed individuals across the founders of the colony. 1 × 0812 is indeed a founder animal with mixed ancestry, characterised by the largest genome fraction of *P. hamadryas* ancestry within the colony. This animal might have been sourced in the wild, possibly from areas where the occurrence of *P. anubis* and *P. hamadryas* hybrids, for example Awash National Park in Ethiopia (Bergman, Beehner 2005), as for the occurrence of *P. hamadryas* ancestry in the Ethiopian *P. anubis* genomes sampled in the wild (Sørensen et al. 2023).

Taking all of this into consideration, most of the *P. hamadryas* ancestry present in the SNPRC data set might predate the foundation of the colony and was probably associated with the occurrence of *P. anubis/P. hamadrya*s hybrids among the founders sampled in the wild. An origin in Eastern Africa is reported for the colony baboons, often referring to Kenya, but animals might have originated from other countries too (Kraemer and Vera Cruz 1972; Moore 1975; Goodwin and Coelho AM 1982). This must have been the case for the other minor ancestries we detected, whose identification is puzzling. The occurrence of *P. kindae*, *P. ursinus*, and *P. papio* ancestries is sporadic, suggesting occasional, possibly even single events of introgression in the history of the colony. It is worth noticing here that different species of *Papio* have been hosted at the Texas facility over the years (Moore 1975; Goodwin and Coelho AM 1982; Dyke et al. 1987) and hybrids additional to *P. anubis x P. cynocephalus* have been reported (see Figure 3C in Chiou and Bergey 2018, where individual SNPRC25567 is reported as *P. anubis x P. ursinus*). This specific sample (SNPRC25567) is not present in the pedigreed data set analysed by Kendall et al. (2024), but it is not inconceivable that other hybrids were at the colony over time. However, if so, they probably had a marginal role, if any, in the making of the pedigree colony, as suggested by the lack of relatives within the colony for samples with *P. kindae*, *P. ursinus*, and *P. papio* ancestries, even if some of these individuals were reported to be colony founders (Kendall et al. 2024; Table S1). None of the less common ancestries, beside *P. hamadryas*, appears to have influenced in an extensive way the colony's mitochondrial diversity. This pattern could be explained by two different scenarios, not mutually excludable. On one side it is possible that these introgression events were sex‐biased, these ancestries solely introduced via male individuals. Pedigreed individuals were sired by pairing one male with groups of females, and additional ancestries might have originated by these males. On the other hand, it is possible that the limited introgression favoured drift‐based dynamics, usually more extreme in haploid systems as for the mitochondrial DNA (Nordborg 1998). In this case the introgression of these rarer ancestries could have involved female individuals whose mitochondrial contribution would have been lost by drift.

### 4.3 Implications for the Use of SNPRC Baboons in Biomedical Research

Hybrids have the potential for being informative models in evolutionary studies related to selection and adaptations, as well as for mapping the genomic architecture of phenotypic traits (Charpentier et al. 2012; Runemark et al. 2019; Vilgalys et al. 2022). However, these applications rely on the careful characterization of the ancestry composition of the investigated samples, so that appropriate corrections can be introduced when required (Price et al. 2006; Alexander and Lange 2011). Without such information, analyses can be influenced by underlying population structure, resulting in possibly biased findings or reduced power to detect biologically meaningful associations (Price et al. 2010; Jones et al. 2025). Our analysis provided evidence that an ancestry profile more complex than expected characterises the genomes of the SNPRC baboons. This complexity does not hamper the use of the SNPRC baboon colony in biomedical studies but highlights the importance of accounting for genetic background when designing experiments, constructing testing cohorts, or interpreting biological variation. For instance, matching ancestry compositions or including ancestry proportions as covariates (Torgerson et al. 2011; Peterson et al. 2019) can help control for confounding effects when assembling testing cohorts in immunological and metabolic studies. In addition, recombination landscapes can also be influenced by ancestry variations (Kong et al. 2010; Hinch et al. 2011), with potential consequences for the construction and interpretation of genetic maps, which in turn are essential for locating genes associated with biomedically relevant traits. Similarly, phenotypic variation, including body size, immune responses, and susceptibility to specific diseases, have been shown to correlate with genetic ancestry in both human and nonhuman primates (Charpentier et al. 2012; Zeberg and Pääbo 2020; Sibley et al. 2021; Das et al. 2022), suggesting that unrecognized hybridization could influence the outcomes of potentially impactful studies.

Our work highlighted the occurrence of additional ancestries within the colony, and this can be taken into account in the appropriate way in future investigations. We are hopeful our results will help researchers to further benefit and maximize the resources provided by the SNPRC baboon colony.

## Supporting information


**Figure S1:** Placement of mitochondrial sequence of 33 SNPRC founders inside the phylogenetic tree of Papio using different numbers of random reads. **Figure S2:** Placement of mitochondrial sequence of 33 SNPRC founders inside the phylogenetic tree of Papio using different mitochondrial reference genomes for extraction and assembly. **Figure S3:** ADMIXTURE analyses for K ranging from 2 to 10 using PDP samples plus SNPRC Founders. **Figure S4:** Heatmap of correlation analyses between PANE results for different number of PCs considered, divided by ancestry. **Figure S5:** Placement of P. hamadryas‐associated mitochondrial sequences inside P. hamadryas cluster (Clade G) using different number of reads. **Figure S6:** Comparison of ancestry estimates across analyses and datasets. **Figure S7:** Karyogram plots of local ancestry estimates.

Media_S1.

Media_S2.

Media_S3.

## References

[ajp70096-bib-0001] Alexander, D. H. , and K. Lange . 2011. “Enhancements to the ADMIXTURE Algorithm for Individual Ancestry Estimation.” BMC Bioinformatics 12: 246. 10.1186/1471-2105-12-246. 21682921 PMC3146885

[ajp70096-bib-0002] Atkinson, E. G. , A. X. Maihofer , M. Kanai , et al. 2021. “Tractor Uses Local Ancestry to Enable the Inclusion of Admixed Individuals in GWAS and to Boost Power.” Nature Genetics 53, no. 2 (February): 195–204. 10.1038/s41588-020-00766-y. 33462486 PMC7867648

[ajp70096-bib-0003] Batra, S. S. , M. Levy‐Sakin , J. Robinson , et al. 2020. “Accurate Assembly of the Olive Baboon (*Papio anubis*) Genome Using Long‐Read and Hi‐C Data.” GigaScience 9, no. 12 (December) : giaa134. 10.1093/gigascience/giaa134. 33283855 PMC7719865

[ajp70096-bib-0004] Bergman, T. J. , and J. C. Beehner . 2004. “Social System of a Hybrid Baboon Group (*Papio anubis* × *P. hamadryas*).” International Journal of Primatology 25: 1313–1330. 10.1023/B:IJOP.0000043964.01085.dc.

[ajp70096-bib-0005] Browning, B. L. , X. Tian , Y. Zhou , and S. R. Browning . 2021. “Fast Two‐Stage Phasing of Large‐Scale Sequence Data.” American Journal of Human Genetics 108, no. 10: 1880–1890. 10.1016/j.ajhg.2021.08.005. 34478634 PMC8551421

[ajp70096-bib-0006] Buettner‐Janusch, J. 1966. “A Problem in Evolutionary Systematics: Nomenclature and Classification of Baboons, Genus Papio.” Folia Primatologica 4, no. 4: 288–308. 10.1159/000155061. 4962265

[ajp70096-bib-0007] Capella‐Gutiérrez, S. , J. M. Silla‐Martínez , and T. Gabaldón . 2009. “Trimal: A Tool for Automated Alignment Trimming in Large‐Scale Phylogenetic Analyses.” Bioinformatics 25, no. 15 (August): 1972–1973. 10.1093/bioinformatics/btp348. 19505945 PMC2712344

[ajp70096-bib-0008] Chang, C. C. , C. C. Chow , L. C. Tellier , S. Vattikuti , S. M. Purcell , and J. J. Lee . 2015. “Second‐Generation PLINK: Rising to the Challenge of Larger and Richer Datasets.” GigaScience 4 (February): 7. 10.1186/s13742-015-0047-8. 25722852 PMC4342193

[ajp70096-bib-0009] Charpentier, M. J. E. , M. C. Fontaine , E. Cherel , et al. 2012. “Genetic Structure in a Dynamic Baboon Hybrid Zone Corroborates Behavioural Observations in a Hybrid Population.” Molecular Ecology 21, no. 3 (February): 715–731. 10.1111/j.1365-294X.2011.05302.x. 21988698

[ajp70096-bib-0010] Chiou, K. L. , and C. M. Bergey . 2018. “Methylation‐Based Enrichment Facilitates Low‐Cost, Noninvasive Genomic Scale Sequencing of Populations from Feces.” Scientific Reports 8, no. 1 (January): 1975. 10.1038/s41598-018-20427-9. 29386638 PMC5792461

[ajp70096-bib-0011] Chiou, K. L. , M. C. Janiak , I. A. Schneider‐Crease , et al. 2022. “Genomic Signatures of High‐Altitude Adaptation and Chromosomal Polymorphism in Geladas.” Nature Ecology & Evolution 6, no. 5 (May): 630–643. 10.1038/s41559-022-01703-4. 35332281 PMC9090980

[ajp70096-bib-0012] Conley, A. B. , L. Rishishwar , M. Ahmad , et al. 2023. “Rye: Genetic Ancestry Inference at Biobank Scale.” Nucleic Acids Research 51, no. 8 (May): e44. 10.1093/nar/gkad149. 36928108 PMC10164567

[ajp70096-bib-0013] Cox, L. A. , A. G. Comuzzie , L. M. Havill , et al. 2013. “Baboons as a Model to Study Genetics and Epigenetics of Human Disease.” ILAR Journal 54, no. 2: 106–121. 10.1093/ilar/ilt038. 24174436 PMC3924757

[ajp70096-bib-0014] Cox, L. A. , M. C. Mahaney , J. L. Vandeberg , and J. Rogers . 2006. “A Second‐Generation Genetic Linkage Map of the Baboon (*Papio hamadryas*) Genome.” Genomics 88, no. 3 (September): 274–281. 10.1016/j.ygeno.2006.03.020. 16697552

[ajp70096-bib-0015] Danecek, P. , J. K. Bonfield , J. Liddle , et al. 2021. “Twelve Years of SAMtools and BCFtools.” GigaScience 10, no. 2 (February): giab008. 10.1093/gigascience/giab008. 33590861 PMC7931819

[ajp70096-bib-0016] Das, R. , T. V. Tatarinova , E. R. Galieva , and Y. L. Orlov . 2022. “Editorial: Association Between Individuals' Genomic Ancestry and Variation in Disease Susceptibility.” Frontiers in Genetics 13 (February): 831320. 10.3389/fgene.2022.831320. 35186044 PMC8847436

[ajp70096-bib-0017] Dyke, B. , T. B. Gage , J. L. VandeBerg , et al. 1987. “Decision Making in Genetic Management of Primate Breeding Colonies.” Genetica 73, no. 1–2 (August): 137–144. 10.1007/BF00057444. 3505886

[ajp70096-bib-0018] Finstermeier, K. , D. Zinner , M. Brameier , et al. 2013. “A Mitogenomic Phylogeny of Living Primates.” PLoS One 8, no. 7 (July): e69504. 10.1371/journal.pone.0069504. 23874967 PMC3713065

[ajp70096-bib-0019] Garcia‐Vilanova, A. , A. Allué‐Guardia , N. M. Chacon , et al. 2024. “Proteomic Analysis of Lung Responses to SARS‐CoV‐2 Infection in Aged Non‐Human Primates: Clinical and Research Relevance.” GeroScience 46, no. 6 (December): 6395–6417. 10.1007/s11357-024-01264-3. 38969861 PMC11493886

[ajp70096-bib-0020] de Gennaro, L. , L. Molinaro , A. Raveane , et al. 2025. “PANE: Fast and Reliable Ancestral Reconstruction on Ancient Genotype Data with Non‐Negative Least Square and Principal Component Analysis.” Genome Biology 26, no. 1 (February): 29. 10.1186/s13059-025-03491-z. 39934833 PMC11818073

[ajp70096-bib-0021] Goodwin, W. J. 1974. “Primate resources—current status and future needs.” In Primate utilization and conservation, edited by G. Bermant and D. G. Lindburg , 4–15. New York: Wiley.

[ajp70096-bib-0022] Goodwin, W. J. , and Jr Coelho AM, Jr. . 1982 Dec. “Development of a Large Scale Baboon Breeding Program.” Laboratory Animal Science 32, no. 6: 672–676. 7162132

[ajp70096-bib-0023] Hinch, A. G. , A. Tandon , N. Patterson , et al. 2011 Jul 20. “The Landscape of Recombination in African Americans.” Nature 476, no. 7359: 170–175. 10.1038/nature10336. 21775986 PMC3154982

[ajp70096-bib-0024] Hodgson, J. A. , K. N. Sterner , L. J. Matthews , et al. 2009 Apr 7. “Successive Radiations, Not Stasis, in the South American Primate Fauna.” Proceedings of the National Academy of Sciences 106, no. 14: 5534–5539. 10.1073/pnas.0810346106. PMC2667060 19321426

[ajp70096-bib-0025] Joganic, J. L. , K. E. Willmore , J. T. Richtsmeier , et al. 2018. “Additive Genetic Variation in the Craniofacial Skeleton of Baboons (Genus Papio) and Its Relationship to Body and Cranial Size.” American Journal of Physical Anthropology 165, no. 2 (February): 269–285. 10.1002/ajpa.23349. 29154459 PMC5966830

[ajp70096-bib-0026] Jones, S. C. , K. M. Cardone , Y. Bradford , S. A. Tishkoff , and M. D. Ritchie . 2025. “The Impact of Ancestry on Genome‐Wide Association Studies.” Pacific Symposium on Biocomputing. Pacific Symposium on Biocomputing 30: 251–267. 39670375 10.1142/9789819807024_0019 PMC11694900

[ajp70096-bib-0027] Katoh, K. , and D. M. Standley . 2013 Apr. “MAFFT Multiple Sequence Alignment Software Version 7: Improvements in Performance and Usability.” Molecular Biology and Evolution 30, no. 4: 772–780. 10.1093/molbev/mst010. 23329690 PMC3603318

[ajp70096-bib-0028] Kendall, C. , J. Robinson , G. Debortoli , et al. 2024 Jul 3. “Global and Local Ancestry Estimation in a Captive Baboon Colony.” PLoS One 19, no. 7: e0305157. 10.1371/journal.pone.0305157. 38959276 PMC11221750

[ajp70096-bib-0029] King, H. A. D. , M. G. Joyce , I. Lakhal‐Naouar , et al. 2021 Sep 21. “Efficacy and Breadth of Adjuvanted SARS‐CoV‐2 Receptor‐Binding Domain Nanoparticle Vaccine in Macaques.” Proceedings of the National Academy of Sciences 118, no. 38: e2106433118. 10.1073/pnas.2106433118. PMC8463842 34470866

[ajp70096-bib-0030] Kong, A. , G. Thorleifsson , D. F. Gudbjartsson , et al. 2010 Oct 28. “Fine‐Scale Recombination Rate Differences Between Sexes, Populations and Individuals.” Nature 467, no. 7319: 1099–1103. 10.1038/nature09525. 20981099

[ajp70096-bib-0031] Korneliussen, T. S. , A. Albrechtsen , and R. Nielsen . 2014. “ANGSD: Analysis of Next Generation Sequencing Data.” BMC Bioinformatics 15: 356. 10.1186/s12859-014-0356-4. 25420514 PMC4248462

[ajp70096-bib-0032] Kraemer, D. C. , S. S. Kalter , and G. T. Moore . 1975. “The Establishment of Non‐Human Primate Breeding Colonies at the Southwest Foundation for Research and Education.” In Breeding Simians for Developmental Biology: Proceedings of the International Association of Biological Standardization symposium, edited by F. T. Perkins and P. N. O'Donoghue , 41–47. (Laboratory animal handbooks; no. 6. London: Laboratory Animals Ltd.

[ajp70096-bib-0033] Kraemer, D. C. , and N. C. Vera Cruz . 1972. “Breeding Baboons for Laboratory Use.” In Breeding Primates: Proceedings of the International Symposium on Breeding Non‐Human Primates for Laboratory Use, Berne, June 1971, edited by W. I. B. Beveridge . Basel: S. Karger.

[ajp70096-bib-0034] Lin, W. , J. D. Wall , G. Li , et al. 2024 Mar 13. “Genetic Regulatory Effects in Response to a High‐Cholesterol, High‐Fat Diet in Baboons.” Cell genomics 4, no. 3: 100509. 10.1016/j.xgen.2024.100509. 38430910 PMC10943580

[ajp70096-bib-0035] Mahaney, M. C. , G. M. Karere , D. L. Rainwater , et al. 2018. “Diet‐Induced Early‐Stage Atherosclerosis in Baboons: Lipoproteins, Atherogenesis, and Arterial Compliance.” Journal of Medical Primatology 47, no. 1 (February): 3–17. 10.1111/jmp.12283. 28620920 PMC5839476

[ajp70096-bib-0036] Manichaikul, A. , J. C. Mychaleckyj , S. S. Rich , K. Daly , M. Sale , and W. M. Chen . 2010. “Robust Relationship Inference in Genome‐Wide Association Studies.” Bioinformatics 26, no. 22: 2867–2873. 20926424 10.1093/bioinformatics/btq559 PMC3025716

[ajp70096-bib-0037] Maples, B. K. , S. Gravel , E. E. Kenny , and C. D. Bustamante . 2013 Aug 8. “RFMix: A Discriminative Modeling Approach for Rapid and Robust Local‐Ancestry Inference.” American Journal of Human Genetics 93, no. 2: 278–288. 10.1016/j.ajhg.2013.06.020. 23910464 PMC3738819

[ajp70096-bib-0038] Massarat, A. R. , M. Lamkin , C. Reeve , A. L. Williams , M. D'Antonio , and M. Gymrek . 2023. “Haptools: A Toolkit for Admixture and Haplotype Analysis.” Bioinformatics 39, no. 3: btad104. 10.1093/bioinformatics/btad104. 36847450 PMC9991497

[ajp70096-bib-0039] McKenna, A. , M. Hanna , E. Banks , et al. 2010 Sep. “The Genome Analysis Toolkit: A Mapreduce Framework for Analyzing Next‐Generation DNA Sequencing Data.” Genome Research 20, no. 9: 1297–1303. 10.1101/gr.107524. 20644199 PMC2928508

[ajp70096-bib-0040] Minh, B. Q. , H. A. Schmidt , O. Chernomor , et al. 2020 May 1. “IQ‐TREE 2: New Models and Efficient Methods for Phylogenetic Inference in the Genomic Era.” Molecular Biology and Evolution 37, no. 5: 1530–1534. Erratum in: Mol Biol Evol. 2020 Aug 1;37(8):2461. 10.1093/molbev/msaa015. 32011700 PMC7182206

[ajp70096-bib-0041] Moore, G. T. 1975 Dec. “The Breeding and Utilization of Baboons for Biomedical Research.” Laboratory Animal Science 25, no. 6: 798–801. 1207052

[ajp70096-bib-0063] Mutti, G. , G. Oteo‐Garcia , M. Caldon , et al. 2023. “Assessing the Recovery of Y Chromosome Microsatellites With Population Genomic Data Using Papio and Theropithecus Genomes.” Scientific Reports 13: 13839. 10.1038/s41598-023-40931-x. 37620368 PMC10449864

[ajp70096-bib-0042] Nordborg, M. 1998. “On the Probability of Neanderthal Ancestry.” American Journal of Human Genetics 63, no. 4: 1237–1240. 10.1086/302052. 9758610 PMC1377484

[ajp70096-bib-0043] Torgerson, D. G. , E. J. Ampleford , G. Y. Chiu , et al. 2011 Jul 31. “Meta‐Analysis of Genome‐Wide Association Studies of Asthma in Ethnically Diverse North American Populations.” Nature Genetics 43, no. 9: 887–892. 10.1038/ng.888. 21804549 PMC3445408

[ajp70096-bib-0044] Oget‐Ebrad, C. , N. K. Kadri , G. C. M. Moreira , et al. 2022. “Benchmarking Phasing Software With a Whole‐Genome Sequenced Cattle Pedigree.” BMC Genomics 23, no. 1 (February): 130. 10.1186/s12864-022-08354-6. 35164677 PMC8845340

[ajp70096-bib-0045] Patterson, N. , A. L. Price , and D. Reich . 2006. “Population Structure and Eigenanalysis.” PLoS Genetics 2, no. 12: e190. 10.1371/journal.pgen.0020190. 17194218 PMC1713260

[ajp70096-bib-0046] Peterson, R. E. , K. Kuchenbaecker , R. K. Walters , et al. 2019 Oct 17. “Genome‐Wide Association Studies in Ancestrally Diverse Populations: Opportunities, Methods, Pitfalls, and Recommendations.” Cell 179, no. 3: 589–603. 10.1016/j.cell.2019.08.051. 31607513 PMC6939869

[ajp70096-bib-0047] Price, A. L. , N. J. Patterson , R. M. Plenge , M. E. Weinblatt , N. A. Shadick , and D. Reich . 2006 Aug. “Principal Components Analysis Corrects for Stratification in Genome‐Wide Association Studies.” Nature Genetics 38, no. 8: 904–909. 10.1038/ng1847. 16862161

[ajp70096-bib-0048] Price, A. L. , N. A. Zaitlen , D. Reich , and N. Patterson . 2010 Jul. “New Approaches to Population Stratification in Genome‐Wide Association Studies.” Nature Reviews Genetics 11, no. 7: 459–463. 10.1038/nrg2813. PMC2975875 20548291

[ajp70096-bib-0049] Ravasini , F. Kabral , H. Solnik , et al. 2024. “The Genomic Portrait of the Picene Culture Provides New Insights Into the Italic Iron Age and the Legacy of the Roman Empire in Central Italy.” Genome Biology 25, no. 1: 1–27. 10.1186/S13059-024-03430-4. 39567978 PMC11580440

[ajp70096-bib-0050] Robinson, J. A. , S. Belsare , S. Birnbaum , et al. 2019 May. “Analysis of 100 High‐Coverage Genomes From a Pedigreed Captive Baboon Colony.” Genome Research 29, no. 5: 848–856. 10.1101/gr.247122.118. 30926611 PMC6499309

[ajp70096-bib-0051] Rogers, J. , and J. E. Hixson . 1997 Sep. “Baboons as an Animal Model for Genetic Studies of Common Human Disease.” American Journal of Human Genetics 61, no. 3: 489–493. 10.1086/515527. 9326312 PMC1715968

[ajp70096-bib-0052] Rogers, J. , M. C. Mahaney , S. M. Witte , et al. 2000 Aug 1. “A Genetic Linkage Map of the Baboon (*Papio hamadryas*) Genome Based on Human Microsatellite Polymorphisms.” Genomics 67, no. 3: 237–247. 10.1006/geno.2000.6245. 10936045

[ajp70096-bib-0053] Rogers, J. , M. Raveendran , R. A. Harris , et al. 2019 Jan 30. “The Comparative Genomics and Complex Population History of *Papio* Baboons.” Science Advances 5, no. 1: eaau6947. 10.1126/sciadv.aau6947. 30854422 PMC6401983

[ajp70096-bib-0054] Roos, C. , S. Knauf , I. S. Chuma , et al. 2021 Mar. “New Mitogenomic Lineages in Papio Baboons and Their Phylogeographic Implications.” American Journal of Physical Anthropology 174, no. 3: 407–417. 10.1002/ajpa.24186. 33244782

[ajp70096-bib-0065] Runemark, A. , M. Vallejo‐Marin , and J. I. Meier . 2019. “Eukaryote Hybrid Genomes.” PLOS Genetics 15, no. 11: e1008404. 10.1371/journal.pgen.1008404. 31774811 PMC6880984

[ajp70096-bib-0055] Sibley, L. , O. Daykin‐Pont , C. Sarfas , J. Pascoe , A. D. White , and S. Sharpe . 2021 Apr 23. “Differences in Host Immune Populations Between Rhesus Macaques and Cynomolgus Macaque Subspecies In Relation to Susceptibility to Mycobacterium Tuberculosis Infection.” Scientific Reports 11, no. 1: 8810. 10.1038/s41598-021-87872-x. 33893359 PMC8065127

[ajp70096-bib-0056] Sørensen, E. F. , R. A. Harris , L. Zhang , et al. 2023 Jun 2. “Genome‐Wide Coancestry Reveals Details of Ancient and Recent Male‐Driven Reticulation in Baboons.” Science 380, no. 6648: eabn8153. 10.1126/science.abn8153. 37262153

[ajp70096-bib-0064] Vasimuddin, M. , S. Misra , H. Li , and S. Aluru . 2019. “Efficient Architecture-Aware Acceleration of BWA-MEM for Multicore Systems.” IEEE Parallel and Distributed Processing Symposium (IPDPS). 10.1109/IPDPS.2019.00041.

[ajp70096-bib-0057] Vilgalys, T. P. , A. S. Fogel , J. A. Anderson , et al. 2022 Aug 5. “Selection Against Admixture and Gene Regulatory Divergence in a Long‐Term Primate Field Study.” Science 377, no. 6606: 635–641. 10.1126/science.abm4917. 35926022 PMC9682493

[ajp70096-bib-0058] Wall, J. D. , S. A. Schlebusch , S. C. Alberts , et al. 2016 Jul. “Genomewide Ancestry and Divergence Patterns From Low‐Coverage Sequencing Data Reveal a Complex History of Admixture in Wild Baboons.” Molecular Ecology 25, no. 14: 3469–3483. Epub 2016 Jun 15. 10.1111/mec.13684. 27145036 PMC5306399

[ajp70096-bib-0062] Zeberg, H. , and S. Pääbo . 2020. “The Major Genetic Risk Factor for Severe COVID‐19 Is Inherited From Neanderthals.” Nature 587, no. 7835: 610–612. 10.1038/s41586-020-2818-3. 32998156

[ajp70096-bib-0059] Zinner, D. , L. F. Groeneveld , C. Keller , and C. Roos . 2009 Apr 23. “Mitochondrial Phylogeography of Baboons (Papio spp.): Indication for Introgressive Hybridization?” In BMC Evol Biol (9, 83. Erratum in: BMC Evol Biol. 2019 Nov 4;19(1):198. doi: 10. 1186/s12862‐019‐1537‐6. 10.1186/1471-2148-9-83. 19389236 PMC2681462

[ajp70096-bib-0060] Zinner, D. , J. Wertheimer , R. Liedigk , L. F. Groeneveld , and C. Roos . 2013 Jan. “Baboon Phylogeny as Inferred From Complete Mitochondrial Genomes.” Am J Phys Anthropol 150, no. 1: 133–140. Epub 2012 Nov 26. Erratum in: Am J Phys Anthropol. 2016 Jun;160(2):364. doi: 10. 1002/ajpa.22973. 10.1002/ajpa.22185. 23180628 PMC3572579

