# Supplementary material for: A Reassessment of the Genomic Ancestry of the World's Largest Captive Baboon Colony
Source: Am J Primatol. 2025 Nov 25;87(11):e70096. doi: 10.1002/ajp.70096 (PMC12646039; doi:10.1002/ajp.70096)
Supplement: Supplementary file 1 — Figure S1: Placement of mitochondrial sequence of 33 SNPRC founders inside the phylogenetic tree of Papio using different numbers of random reads. Figure S2: Placement of mitochondrial sequence of 33 SNPRC founders inside the phylogenetic tree of Papio using different mitochondrial reference genomes for extraction and assembly. Figure S3: ADMIXTURE analyses for K ranging from 2 to 10 using PDP samples plus SNPRC Founders. Figure S4: Heatmap of correlation analyses between PANE results for different number of PCs considered, divided by ancestry. Figure S5: Placement of P. hamadryas‐associated mitochondrial sequences inside P. hamadryas cluster (Clade G) using different number of reads. Figure S6: Comparison of ancestry estimates across analyses and datasets. Figure S7: Karyogram plots of local ancestry estimates. [file AJP-87-e70096-s003.pdf]

Supplementary Info for:

A reassessment of the genomic ancestry of the world's largest captive baboon colony

Giacomo Mercuri[1], Fabrizio Dallaspezia[1], Francesco Montinaro[2,3], Cristian Capelli[1]

1. Department of Chemistry, Life Sciences and Environmental Sustainability, University of Parma, Parma, Italy
2. Department of Biosciences, Biotechnology and Environment, University of Bari, Bari, Italy.
3. Institute of Genomics, University of Tartu, Tartu, Estonia.

This PDF includes:

Supplementary Figure 1……………………………………………………………p.1

Supplementary Figure 2………………………………………………………………p.7

Supplementary Figure 3………………………………………………………………p.8

Supplementary Figure 4………………………………………………………………p.9

Supplementary Figure 5………………………………………………………………p.10

Supplementary Figure 6………………………………………………………………p.11

Supplementary Figure 7………………………………………………………………p.12

Supplementary Table 1………………………………………………………………p.13

Supplementary Table 2………………………………………………………………p.25

Supplementary Table 3………………………………………………………………p.37

Supplementary Table 4………………………………………………………………p.38

Supplementary Table 5………………………………………………………………p.39

Supplementary Table 6………………………………………………………………p.40

Supplementary Table 7………………………………………………………………p.46

Supplementary Table 8………………………………………………………………p.47

Supplementary Table 9……………………………………..……………………p.61

Supplementary Table 10……………………………...…………………….p.104

Supplementary Table 11……………………………...…………………….p.105

Supplementary References……………………………………….………..p.106

a

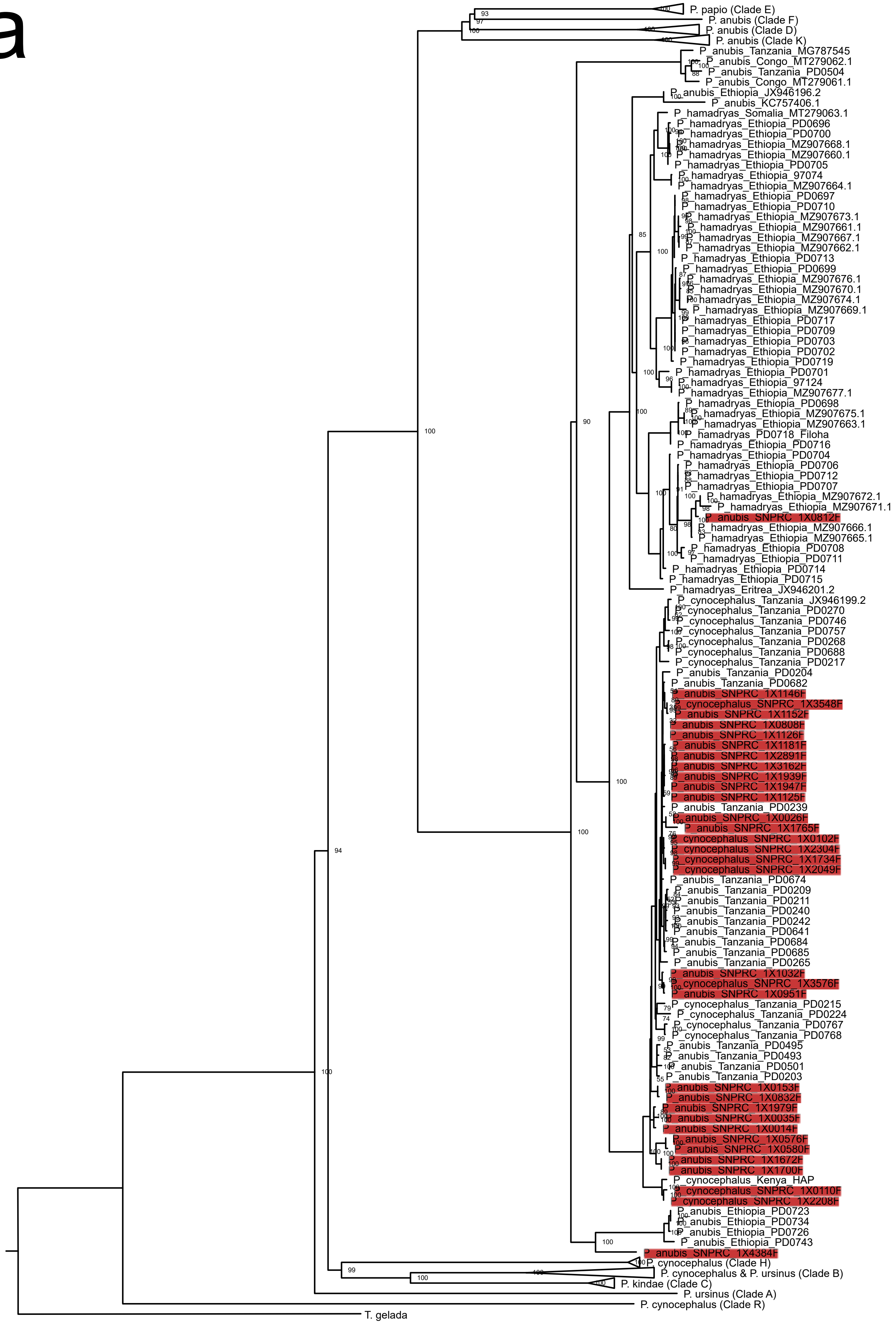

Figure S1. Placement of mitochondrial sequence of 33 SNPRC founders inside the phylogenetic tree of *Papio* using different numbers of random reads. a) 5 million reads. b) 10 million reads. c) 15 million reads. d) 20 million reads. e) 25 million reads. f) All reads available for the sample. Founders' sequences highlighted in red.

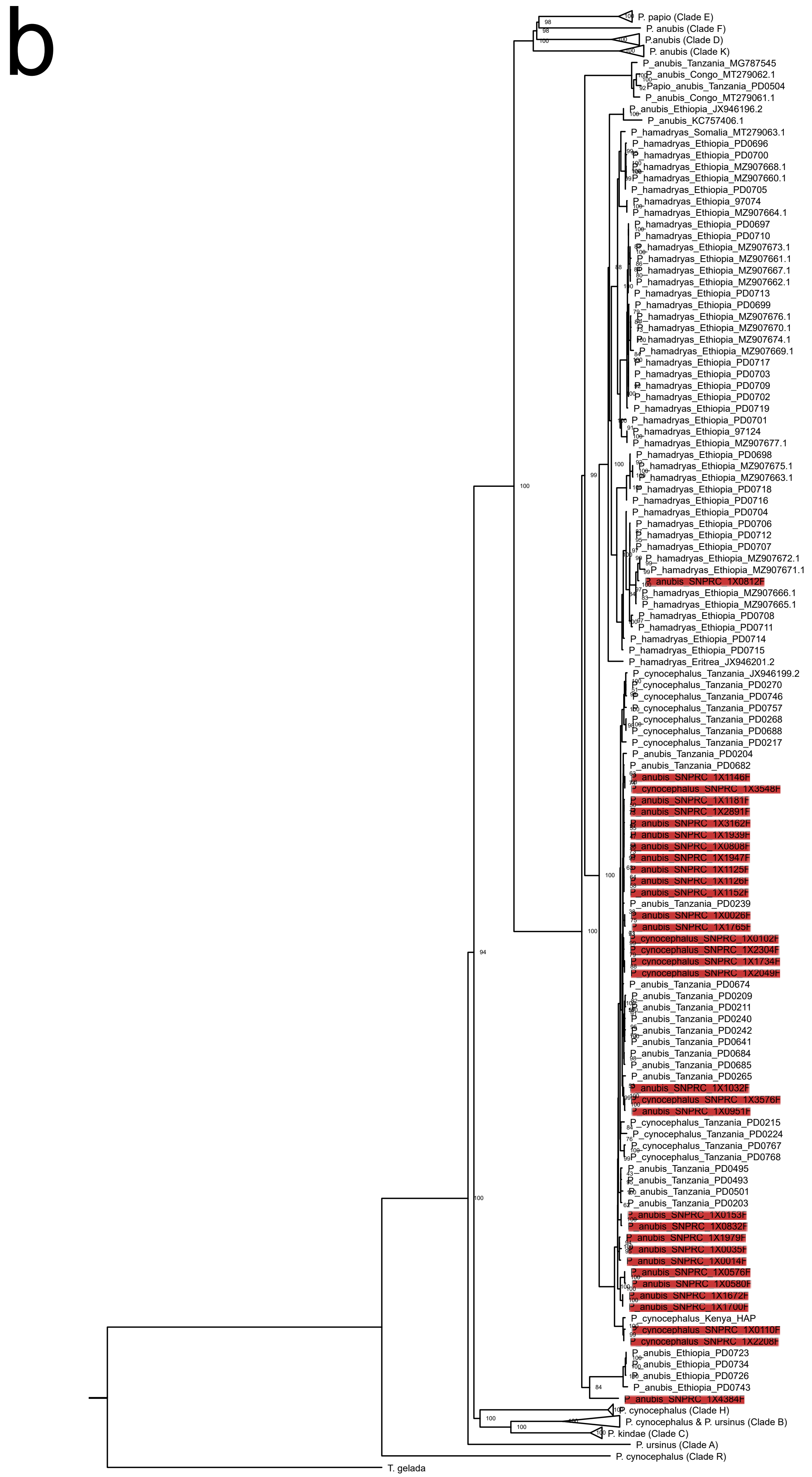

Figure S1. Placement of mitochondrial sequence of 33 SNPRC founders inside the phylogenetic tree of *Papio* using different numbers of random reads. a) 5 million reads. b) 10 million reads. c) 15 million reads. d) 20 million reads. e) 25 million reads. f) All reads available for the sample. Founders' sequences highlighted in red.

C

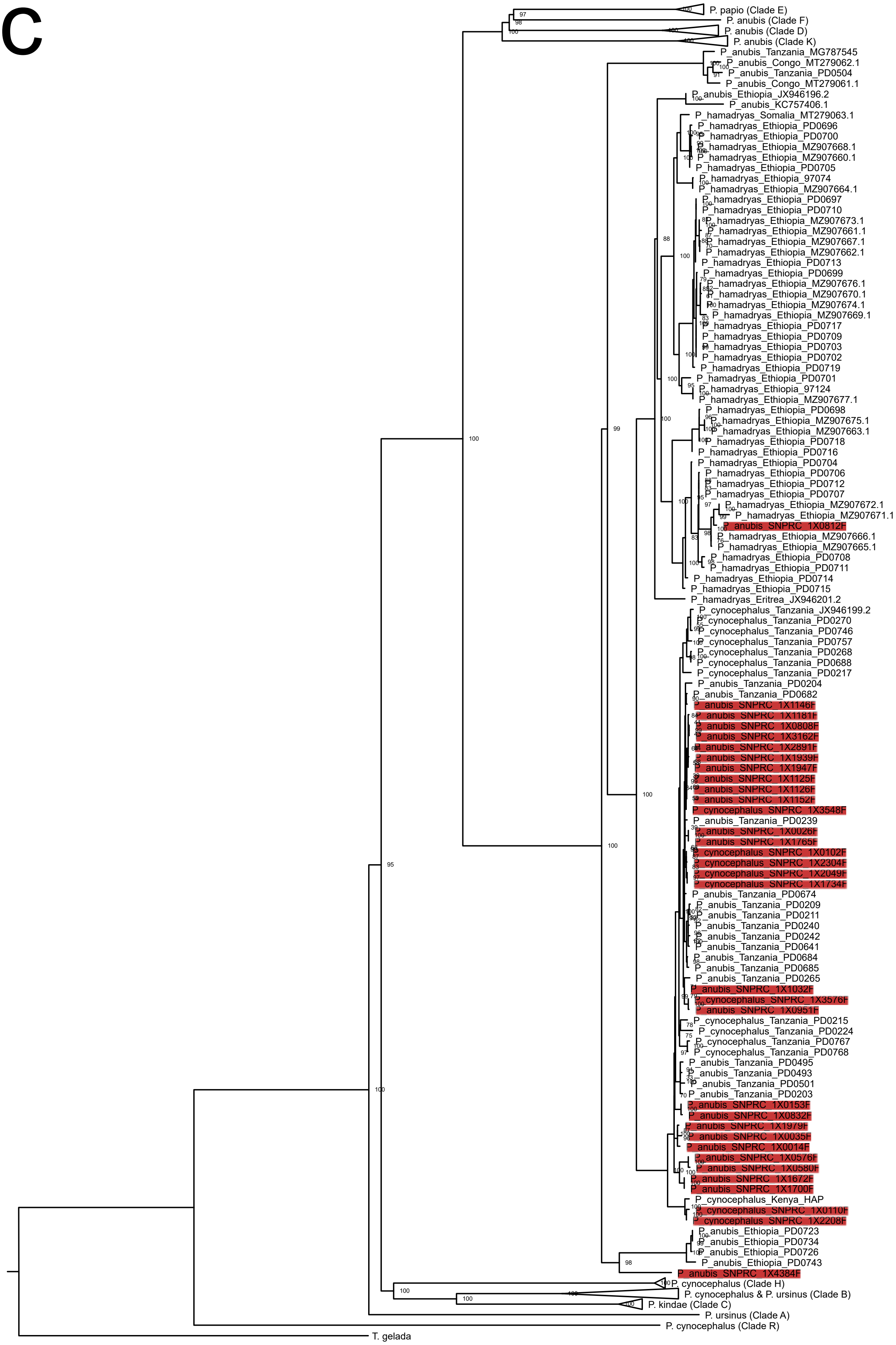

Figure S1. Placement of mitochondrial sequence of 33 SNPRC founders inside the phylogenetic tree of *Papio* using different numbers of random reads. a) 5 million reads. b) 10 million reads. c) 15 million reads. d) 20 million reads. e) 25 million reads. f) All reads available for the sample. Founders' sequences highlighted in red.

d

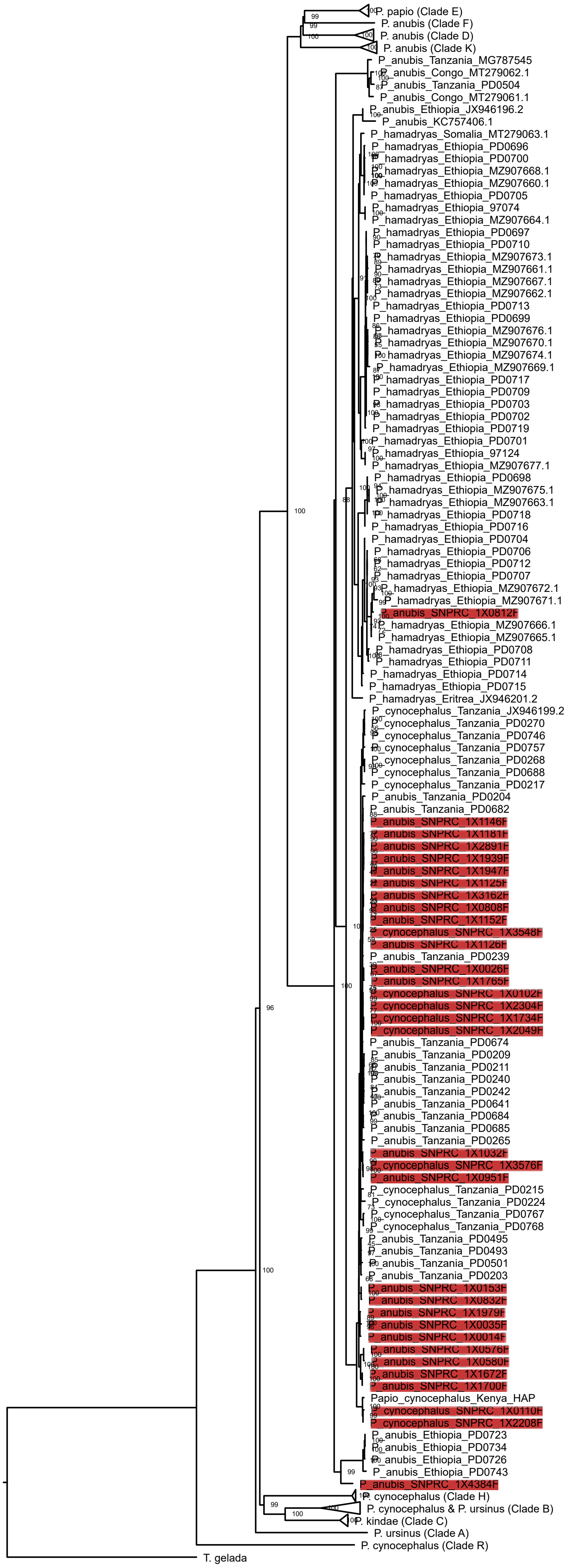

Figure S1. Placement of mitochondrial sequence of 33 SNPRC founders inside the phylogenetic tree of *Papio* using different numbers of random reads. a) 5 million reads. b) 10 million reads. c) 15 million reads. d) 20 million reads. e) 25 million reads. f) All reads available for the sample. Founders' sequences highlighted in red.

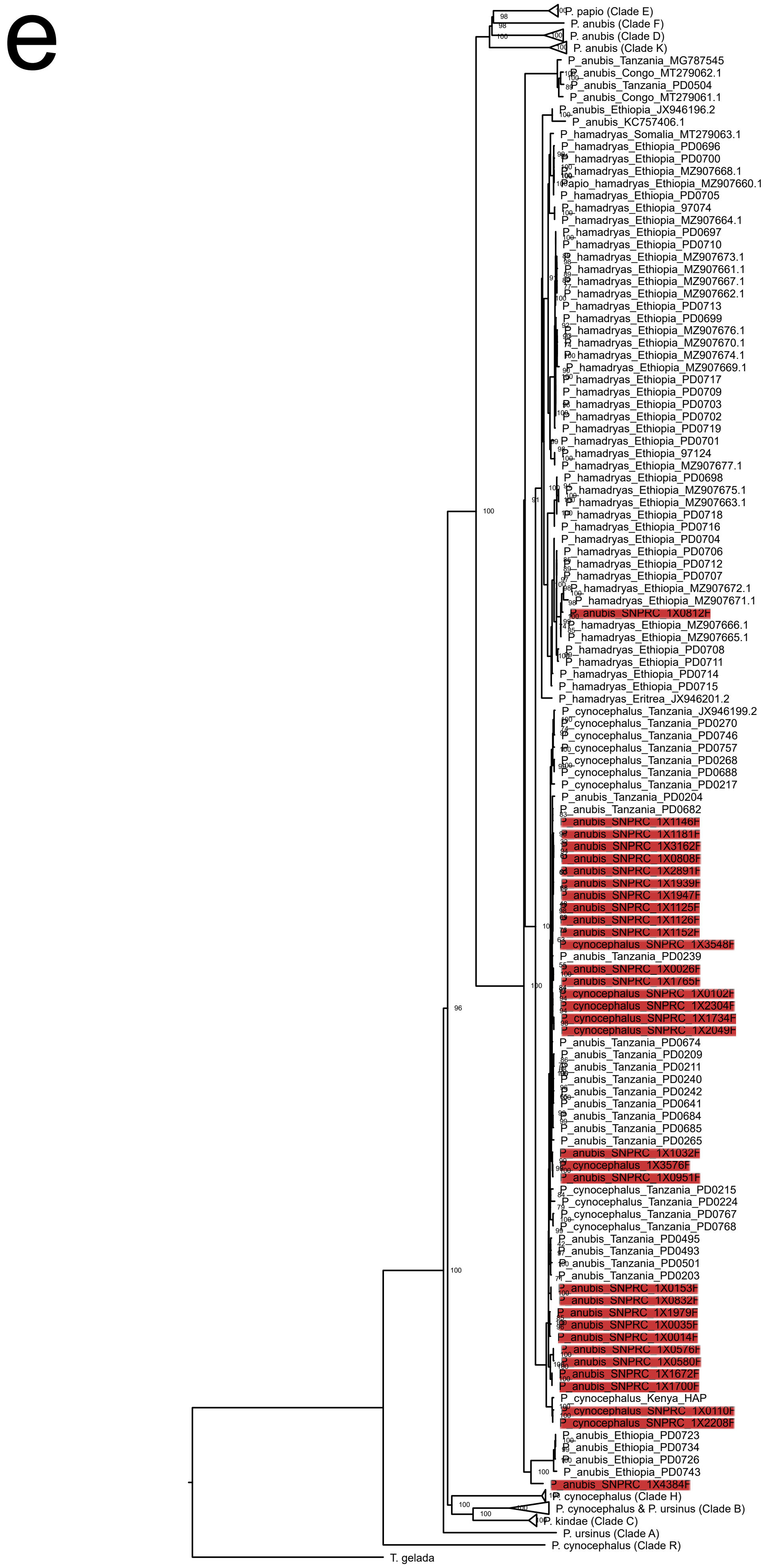

Figure S1. Placement of mitochondrial sequence of 33 SNPRC founders inside the phylogenetic tree of *Papio* using different numbers of random reads. a) 5 million reads. b) 10 million reads. c) 15 million reads. d) 20 million reads. e) 25 million reads. f) All reads available for the sample. Founders' sequences highlighted in red.

f

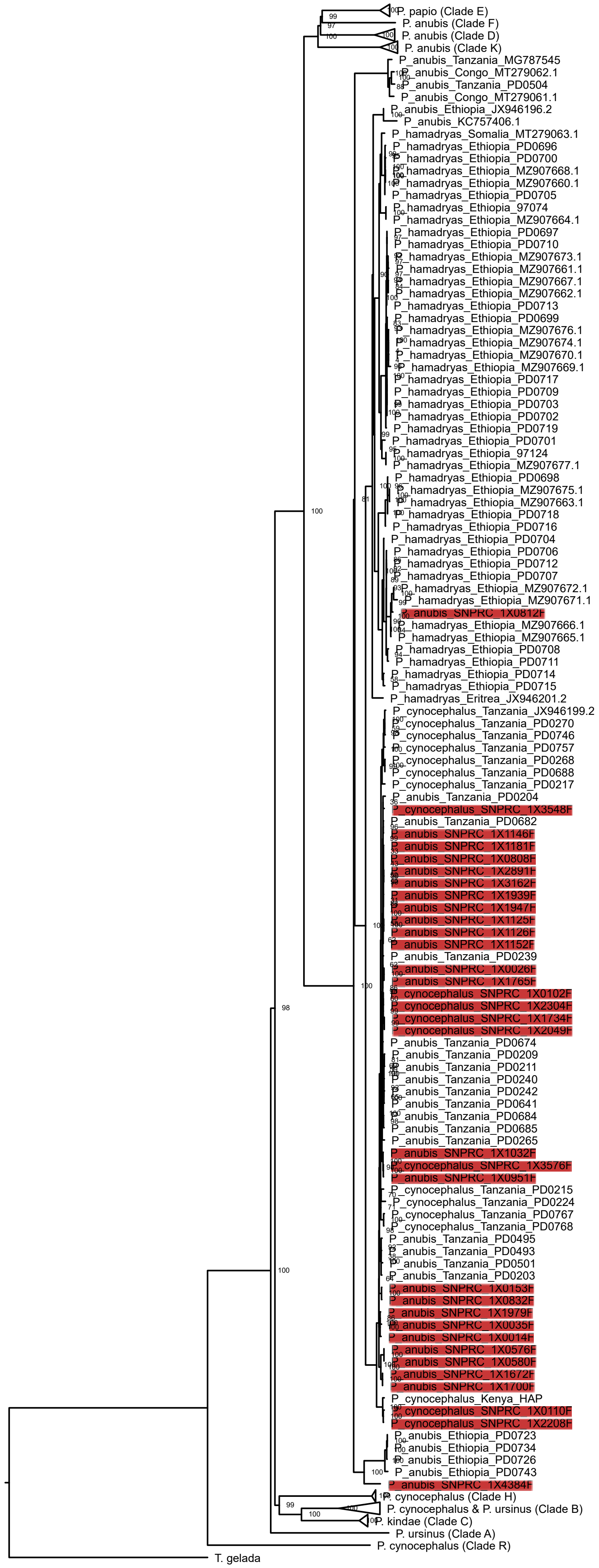

Figure S1. Placement of mitochondrial sequence of 33 SNPRC founders inside the phylogenetic tree of *Papio* using different numbers of random reads. a) 5 million reads. b) 10 million reads. c) 15 million reads. d) 20 million reads. e) 25 million reads. f) All reads available for the sample. Founders' sequences highlighted in red.

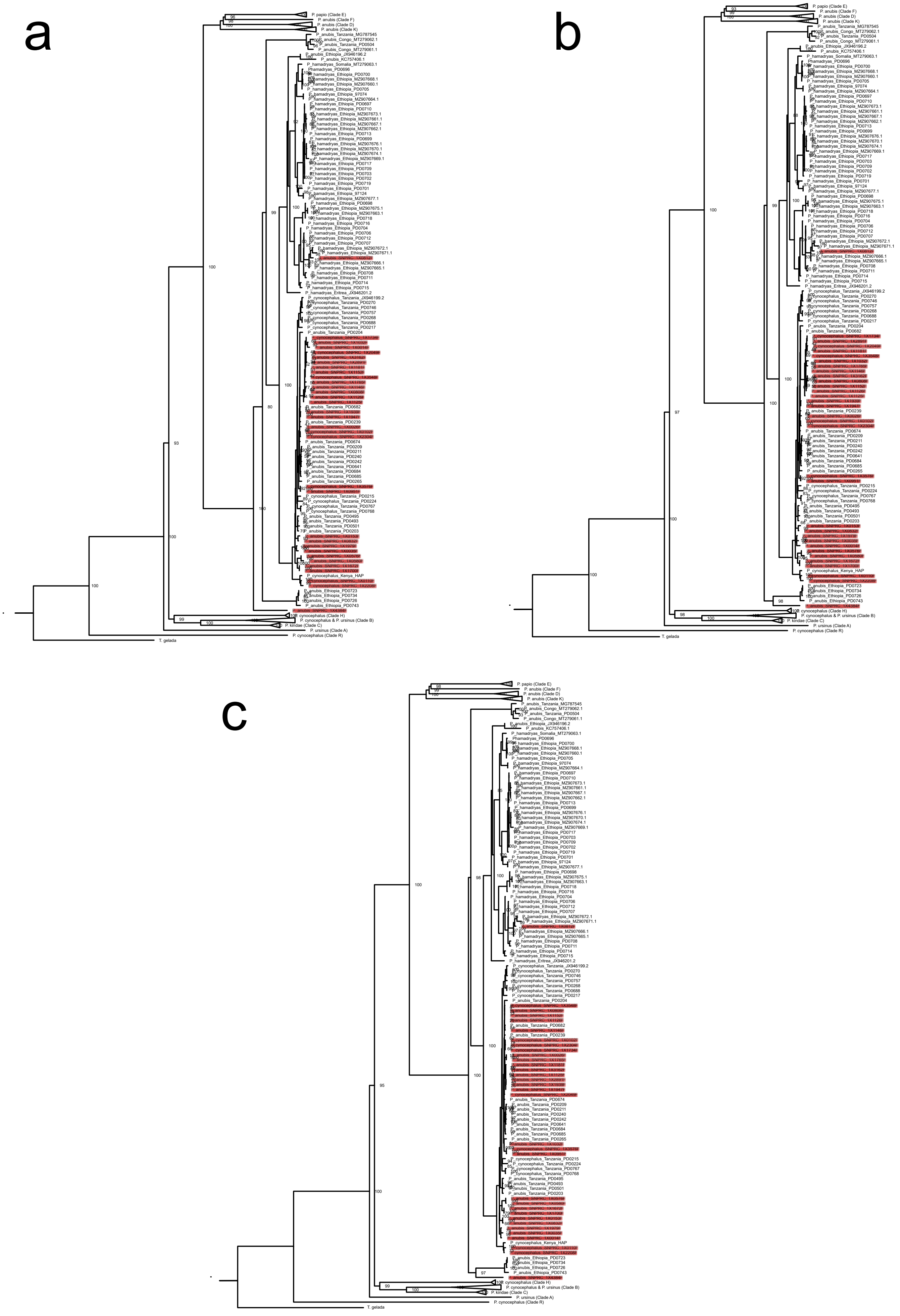

Figure S2. Placement of mitochondrial sequence of 33 SNPRC founders inside the phylogenetic tree of *Papio* using different mitochondrial reference genomes for extraction and assembly. a) *P. anubis* from Nigeria (Clade F; JX946198.2). b) *P. cynocephalus* from Tanzania (Clade B2; JX946200.2). c) *P. cynocephalus* from Tanzania (Clade G; JX946199.2). Founder's sequences highlighted in red.

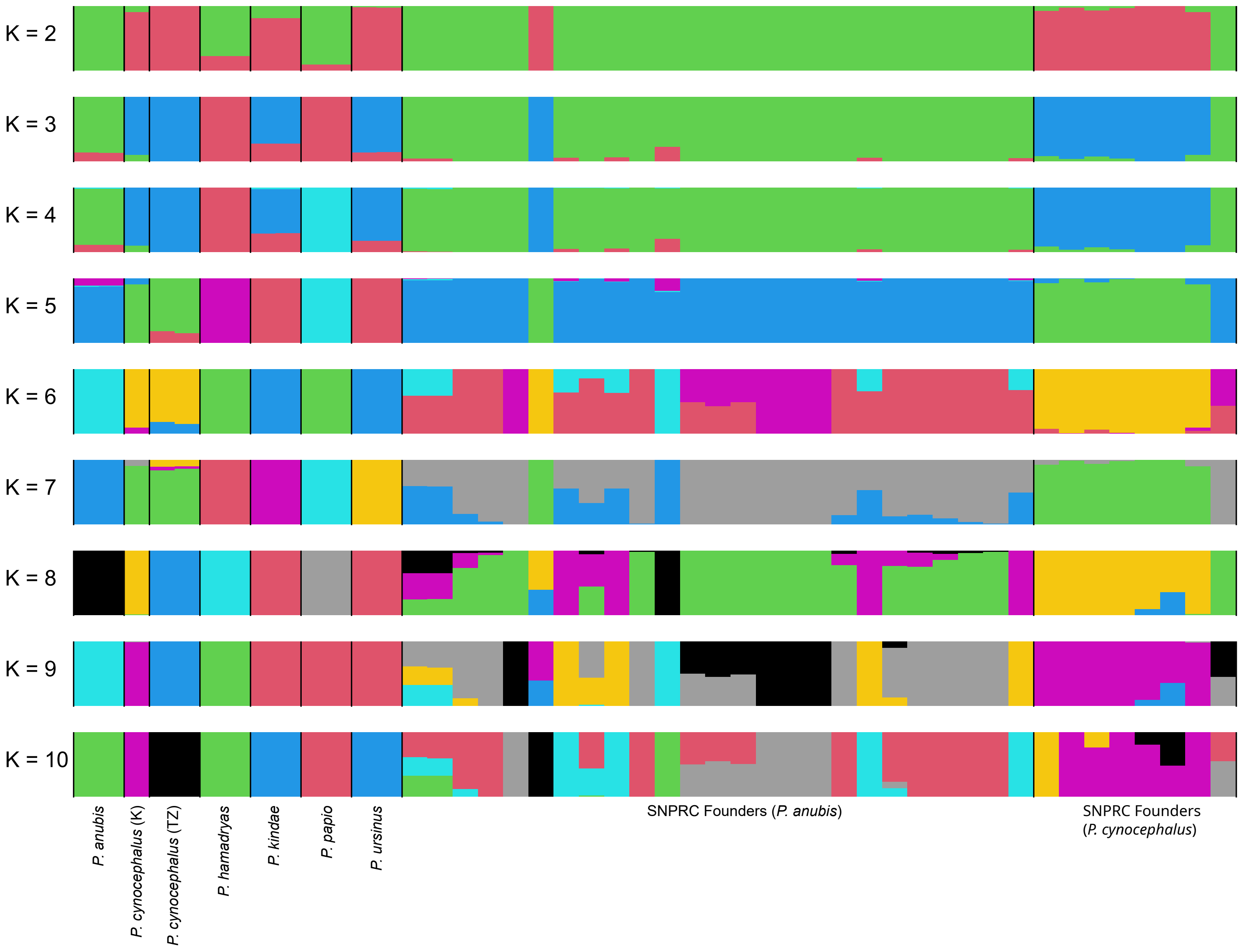

Figure S3. ADMIXTURE analyses for K ranging from 2 to 10 using PDP samples plus SNPRC Founders. Species/population of each sample are shown at the bottom. Samples ordered as Figure 2a. TZ: Tanzania; K: Kenya

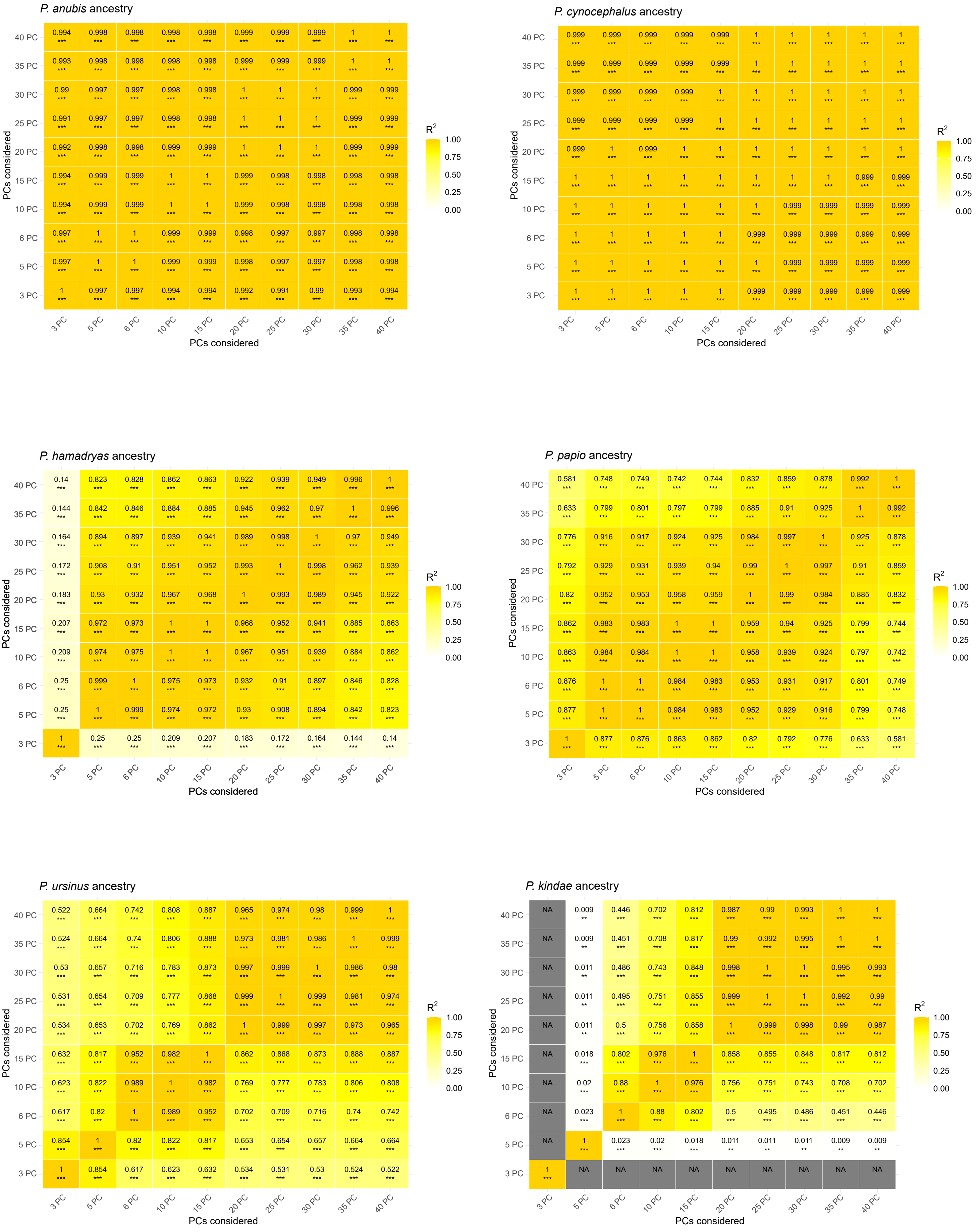

Figure S4.Heatmap of correlation analyses between PANE results for different number of PCs considered, divided by ancestry. Correlations coloured based on R^2 values, while relative p-values are reported as following: * : <0.05; **: <0.01; *** : < 0.001. FDR correction for multiple p-values was applied.

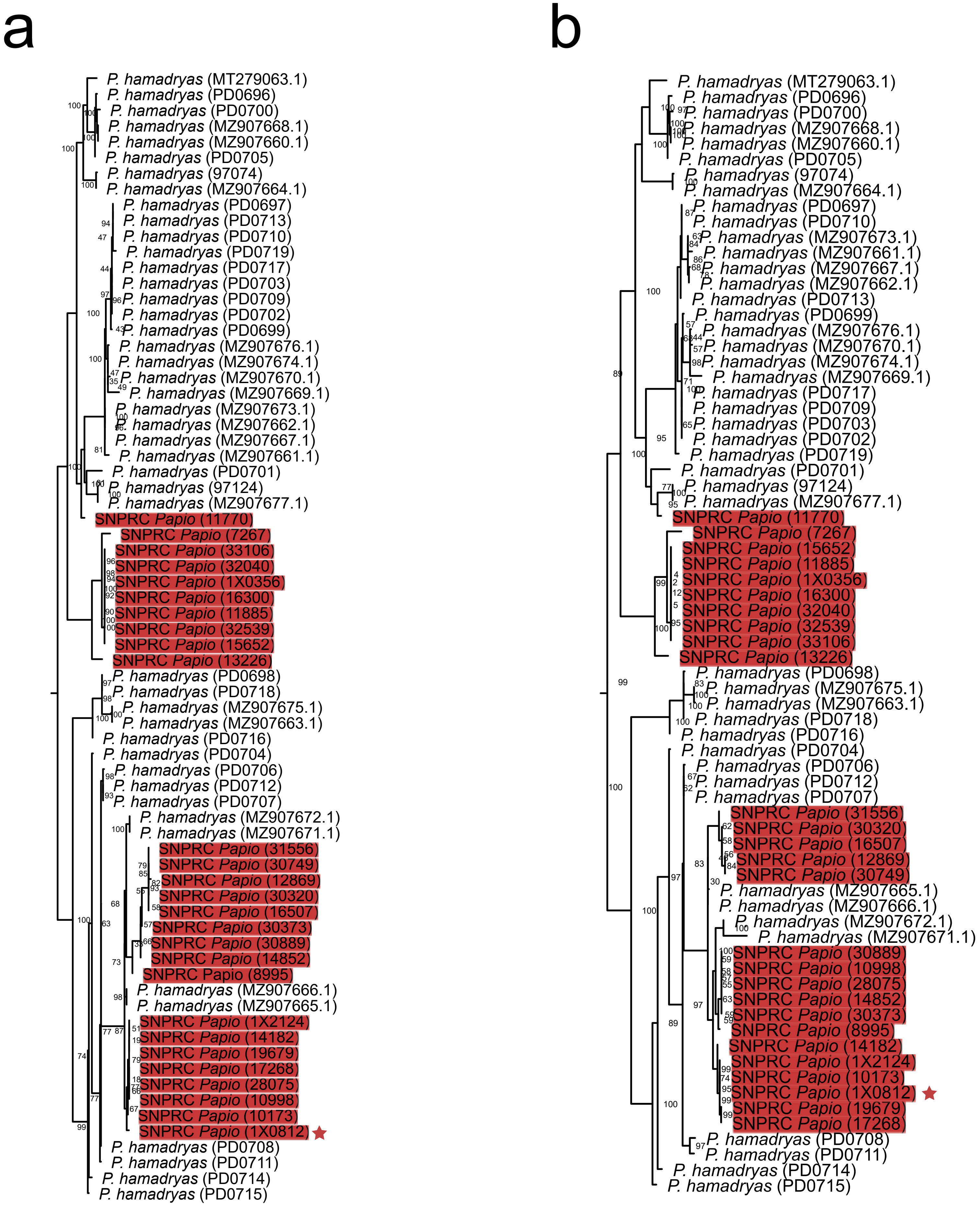

Figure S5. Placement of *P. hamadryas*-associated mitochondrial sequences inside *P. hamadryas* cluster (Clade G) using different number of reads. a) 15 million reads. b) All reads available for the sample. SNPRC sequences highlighted in red, SNPRC founder indicated by star.

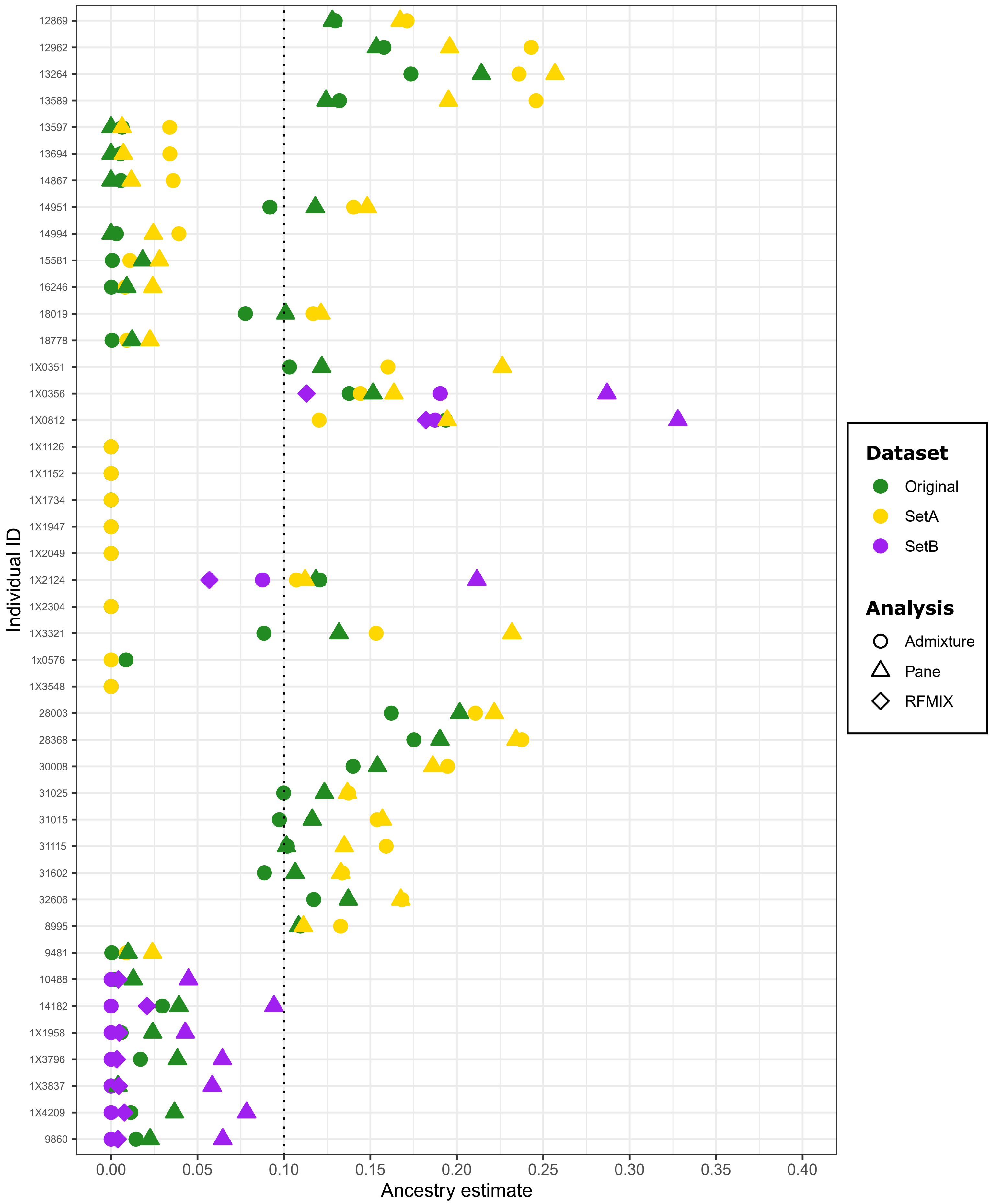

Figure S6. Comparison of ancestry estimates across analyses and datasets. On the Y axis, individuals present in Set A and/or Set B. On the X axis, ancestry estimates for the same individuals across datasets and analyses, following the ancestries reported in Table S11. Dot color based on dataset utilized, dot shape based on analysis performed.

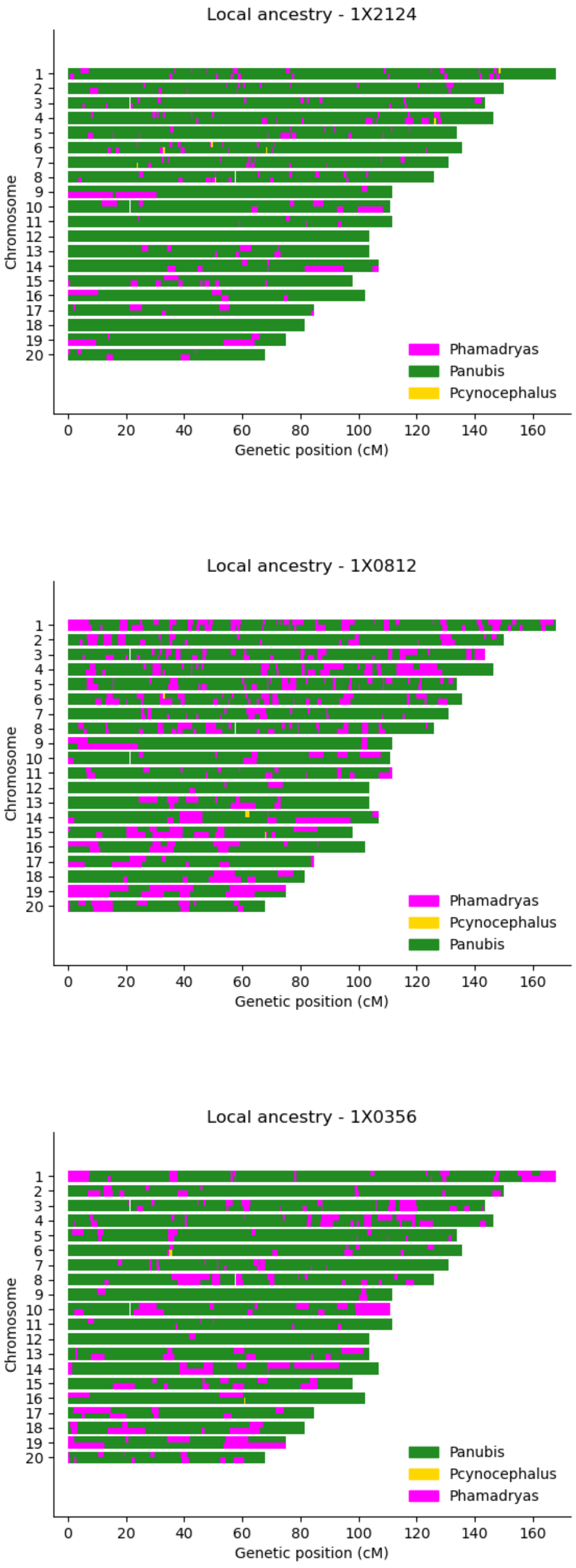

Figure S7. Karyogram plots of local ancestry estimates. From top to bottom, samples 1X2124, 1X0812, and 1X0356. Local ancestry was performed after phasing samples using Beagle v.5.4 and local ancestry was reconstructed through RFMIX v.2.03. Karyograms generated using haptools v0.5.0. Green represents *P. anubis* ancestry, yellow represents *P. cynocephalus* ancestry, and purple represents *P. hamadryas* ancestry.

Sample	Genus	Species	Country	Locality	Source	Dataset	Note
PD_0720	*Papio*	*anubis*	Ethiopia	Gog	Sørensen et al., 2023	PDP	
PD_0727	*Papio*	*anubis*	Ethiopia	Gog	Sørensen et al., 2023	PDP	
PD_0746	*Papio*	*cynocephalus*	Tanzania	Mikumi National	Sørensen et al., 2023	PDP	
PD_0770	*Papio*	*cynocephalus*	Tanzania	Mikumi National	Sørensen et al., 2023	PDP	
HAP	*Papio*	*cynocephalus*	Kenya	Amboseli Nationa	Wall et al., 2016	PDP	
SAMN20949827	*Papio*	*hamadryas*	Ethiopia	Filoha	Chiou et al., 2022	PDP	Selected for PANE
SAMN20949837	*Papio*	*hamadryas*	Ethiopia	Filoha	Chiou et al., 2022	PDP	Selected for PANE
PD_0749	*Papio*	*kindae*	Zambia	Chunga	Sørensen et al., 2023	PDP	Selected for PANE
PD_0751	*Papio*	*kindae*	Zambia	Chunga	Sørensen et al., 2023	PDP	Selected for PANE
PD_0400	*Papio*	*papio*	Senegal	Niokolo-Koba	Sørensen et al., 2023	PDP	Selected for PANE
PD_0401	*Papio*	*papio*	Senegal	Niokolo-Koba	Sørensen et al., 2023	PDP	Selected for PANE
PD_0692	*Papio*	*ursinus*	Zambia	Dendro Park	Sørensen et al., 2023	PDP	Selected for PANE
PD_0693	*Papio*	*ursinus*	Zambia	Dendro Park	Sørensen et al., 2023	PDP	Selected for PANE
1X0576	*Papio*	*anubis*	United States of /	SNPRC	Kendall et al., 2024	SNPRC FOUNDER	
1X0580	*Papio*	*anubis*	United States of /	SNPRC	Kendall et al., 2024	SNPRC FOUNDER	
1X2054	*Papio*	*anubis*	United States of /	SNPRC	Kendall et al., 2024	SNPRC	Reported as FOUNDER by Kendall et al., (2024)
1X1960	*Papio*	*anubis*	United States of /	SNPRC	Kendall et al., 2024	SNPRC	Reported as FOUNDER by Kendall et al., (2024)
14712	*Papio*	*anubis*	United States of /	SNPRC	Kendall et al., 2024	SNPRC	Reported as FOUNDER by Kendall et al., (2024)
1X0153	*Papio*	*anubis*	United States of /	SNPRC	Kendall et al., 2024	SNPRC FOUNDER	
1X0291	*Papio*	*anubis*	United States of /	SNPRC	Kendall et al., 2024	SNPRC	Reported as FOUNDER by Kendall et al., (2024)
1X1947	*Papio*	*anubis*	United States of /	SNPRC	Kendall et al., 2024	SNPRC FOUNDER	
1X1152	*Papio*	*anubis*	United States of /	SNPRC	Kendall et al., 2024	SNPRC FOUNDER	Selected for PANE
1X0102	*Papio*	*cynocephalus*	United States of /	SNPRC	Kendall et al., 2024	SNPRC FOUNDER	
1X1487	*Papio*	*cynocephalus*	United States of /	SNPRC	Kendall et al., 2024	SNPRC	Reported as FOUNDER by Kendall et al., (2024)
1X1734	*Papio*	*cynocephalus*	United States of /	SNPRC	Kendall et al., 2024	SNPRC FOUNDER	
1X2049	*Papio*	*cynocephalus*	United States of /	SNPRC	Kendall et al., 2024	SNPRC FOUNDER	
1X2208	*Papio*	*cynocephalus*	United States of /	SNPRC	Kendall et al., 2024	SNPRC FOUNDER	
1X2304	*Papio*	*cynocephalus*	United States of /	SNPRC	Kendall et al., 2024	SNPRC FOUNDER	Selected for PANE
1X4384	*Papio*	*anubis*	United States of /	SNPRC	Kendall et al., 2024	SNPRC FOUNDER	
1X4811	*Papio*	*cynocephalus*	United States of /	SNPRC	Kendall et al., 2024	SNPRC	Reported as FOUNDER by Kendall et al., (2024)
1X3548	*Papio*	*cynocephalus*	United States of /	SNPRC	Kendall et al., 2024	SNPRC FOUNDER	Selected for PANE
1X0014	*Papio*	*anubis*	United States of /	SNPRC	Kendall et al., 2024	SNPRC FOUNDER	
1X0026	*Papio*	*anubis*	United States of /	SNPRC	Kendall et al., 2024	SNPRC FOUNDER	
1X0035	*Papio*	*anubis*	United States of /	SNPRC	Kendall et al., 2024	SNPRC FOUNDER	
1X0110	*Papio*	*cynocephalus*	United States of /	SNPRC	Kendall et al., 2024	SNPRC FOUNDER	
1X0351	*Papio*	*anubis*	United States of /	SNPRC	Kendall et al., 2024	SNPRC	Reported as FOUNDER by Kendall et al., (2024)
1X0354	*Papio*	*hybrid*	United States of /	SNPRC	Kendall et al., 2024	SNPRC	Reported as FOUNDER by Kendall et al., (2024)
1X0356	*Papio*	*hybrid*	United States of /	SNPRC	Kendall et al., 2024	SNPRC	Reported as FOUNDER by Kendall et al., (2024)
1X0808	*Papio*	*anubis*	United States of /	SNPRC	Kendall et al., 2024	SNPRC FOUNDER	
1X0812	*Papio*	*anubis*	United States of /	SNPRC	Kendall et al., 2024	SNPRC FOUNDER	
1X0832	*Papio*	*anubis*	United States of /	SNPRC	Kendall et al., 2024	SNPRC FOUNDER	
1X0843	*Papio*	*hybrid*	United States of /	SNPRC	Kendall et al., 2024	SNPRC	Reported as FOUNDER by Kendall et al., (2024)
1X0951	*Papio*	*anubis*	United States of /	SNPRC	Kendall et al., 2024	SNPRC FOUNDER	
1X1032	*Papio*	*anubis*	United States of /	SNPRC	Kendall et al., 2024	SNPRC FOUNDER	
1X1125	*Papio*	*anubis*	United States of /	SNPRC	Kendall et al., 2024	SNPRC FOUNDER	
1X1126	*Papio*	*anubis*	United States of /	SNPRC	Kendall et al., 2024	SNPRC FOUNDER	Selected for PANE
1X1146	*Papio*	*anubis*	United States of /	SNPRC	Kendall et al., 2024	SNPRC FOUNDER	
1X1181	*Papio*	*anubis*	United States of /	SNPRC	Kendall et al., 2024	SNPRC FOUNDER	
1X1230	*Papio*	*hybrid*	United States of /	SNPRC	Kendall et al., 2024	SNPRC	Reported as FOUNDER by Kendall et al., (2024)
1X1237	*Papio*	*hybrid*	United States of /	SNPRC	Kendall et al., 2024	SNPRC	Reported as FOUNDER by Kendall et al., (2024)
1X1392	*Papio*	*hybrid*	United States of /	SNPRC	Kendall et al., 2024	SNPRC	Reported as FOUNDER by Kendall et al., (2024)
1X1672	*Papio*	*anubis*	United States of /	SNPRC	Kendall et al., 2024	SNPRC FOUNDER	
1X1700	*Papio*	*anubis*	United States of /	SNPRC	Kendall et al., 2024	SNPRC FOUNDER	
1X1765	*Papio*	*anubis*	United States of /	SNPRC	Kendall et al., 2024	SNPRC FOUNDER	
1X1939	*Papio*	*anubis*	United States of /	SNPRC	Kendall et al., 2024	SNPRC FOUNDER	
1X1958	*Papio*	*hybrid*	United States of /	SNPRC	Kendall et al., 2024	SNPRC	Reported as FOUNDER by Kendall et al., (2024)
1X2055	*Papio*	*hybrid*	United States of /	SNPRC	Kendall et al., 2024	SNPRC	Reported as FOUNDER by Kendall et al., (2024)
1X2664	*Papio*	*hybrid*	United States of /	SNPRC	Kendall et al., 2024	SNPRC	Reported as FOUNDER by Kendall et al., (2024)
1X2891	*Papio*	*anubis*	United States of /	SNPRC	Kendall et al., 2024	SNPRC FOUNDER	
1X3162	*Papio*	*anubis*	United States of /	SNPRC	Kendall et al., 2024	SNPRC FOUNDER	
1X3321	*Papio*	*hybrid*	United States of /	SNPRC	Kendall et al., 2024	SNPRC	Reported as FOUNDER by Kendall et al., (2024)
1X3576	*Papio*	*cynocephalus*	United States of /	SNPRC	Kendall et al., 2024	SNPRC FOUNDER	
1X4519	*Papio*	*hybrid*	United States of /	SNPRC	Kendall et al., 2024	SNPRC	Reported as FOUNDER by Kendall et al., (2024)
1X1979	*Papio*	*anubis*	United States of /	SNPRC	Kendall et al., 2024	SNPRC FOUNDER	
10046	*Papio*	*hybrid*	United States of /	SNPRC	Kendall et al., 2024	SNPRC	
10099	*Papio*	*hybrid*	United States of /	SNPRC	Kendall et al., 2024	SNPRC	
10164	*Papio*	*hybrid*	United States of /	SNPRC	Kendall et al., 2024	SNPRC	
10173	*Papio*	*hybrid*	United States of /	SNPRC	Kendall et al., 2024	SNPRC	
10192	*Papio*	*hybrid*	United States of /	SNPRC	Kendall et al., 2024	SNPRC	
10316	*Papio*	*hybrid*	United States of /	SNPRC	Kendall et al., 2024	SNPRC	
10349	*Papio*	*hybrid*	United States of /	SNPRC	Kendall et al., 2024	SNPRC	
10418	*Papio*	*hybrid*	United States of /	SNPRC	Kendall et al., 2024	SNPRC	
10482	*Papio*	*hybrid*	United States of /	SNPRC	Kendall et al., 2024	SNPRC	
10488	*Papio*	*hybrid*	United States of /	SNPRC	Kendall et al., 2024	SNPRC	
10489	*Papio*	*hybrid*	United States of /	SNPRC	Kendall et al., 2024	SNPRC	

Sample	Genus	Species	Country	Locality	Source	Dataset	Note		
10842	*Papio*	*hybrid*	United States of /	SNPRC	Kendall et al., 2024	SNPRC			
10987	*Papio*	*hybrid*	United States of /	SNPRC	Kendall et al., 2024	SNPRC			
10998	*Papio*	*hybrid*	United States of /	SNPRC	Kendall et al., 2024	SNPRC			
11004	*Papio*	*hybrid*	United States of /	SNPRC	Kendall et al., 2024	SNPRC			
11158	*Papio*	*hybrid*	United States of /	SNPRC	Kendall et al., 2024	SNPRC			
11562	*Papio*	*hybrid*	United States of /	SNPRC	Kendall et al., 2024	SNPRC			
11608	*Papio*	*hybrid*	United States of /	SNPRC	Kendall et al., 2024	SNPRC			
11693	*Papio*	*hybrid*	United States of /	SNPRC	Kendall et al., 2024	SNPRC			
11752	*Papio*	*hybrid*	United States of /	SNPRC	Kendall et al., 2024	SNPRC			
11769	*Papio*	*hybrid*	United States of /	SNPRC	Kendall et al., 2024	SNPRC			
11770	*Papio*	*hybrid*	United States of /	SNPRC	Kendall et al., 2024	SNPRC			
11885	*Papio*	*hybrid*	United States of /	SNPRC	Kendall et al., 2024	SNPRC			
11887	*Papio*	*hybrid*	United States of /	SNPRC	Kendall et al., 2024	SNPRC			
11959	*Papio*	*hybrid*	United States of /	SNPRC	Kendall et al., 2024	SNPRC			
11967	*Papio*	*hybrid*	United States of /	SNPRC	Kendall et al., 2024	SNPRC			
11981	*Papio*	*hybrid*	United States of /	SNPRC	Kendall et al., 2024	SNPRC			
12138	*Papio*	*hybrid*	United States of /	SNPRC	Kendall et al., 2024	SNPRC			
12141	*Papio*	*hybrid*	United States of /	SNPRC	Kendall et al., 2024	SNPRC			
12152	*Papio*	*hybrid*	United States of /	SNPRC	Kendall et al., 2024	SNPRC			
12156	*Papio*	*hybrid*	United States of /	SNPRC	Kendall et al., 2024	SNPRC			
12175	*Papio*	*hybrid*	United States of /	SNPRC	Kendall et al., 2024	SNPRC			
12218	*Papio*	*hybrid*	United States of /	SNPRC	Kendall et al., 2024	SNPRC			
12226	*Papio*	*hybrid*	United States of /	SNPRC	Kendall et al., 2024	SNPRC			
12242	*Papio*	*hybrid*	United States of /	SNPRC	Kendall et al., 2024	SNPRC			
12282	*Papio*	*hybrid*	United States of /	SNPRC	Kendall et al., 2024	SNPRC			
12423	*Papio*	*hybrid*	United States of /	SNPRC	Kendall et al., 2024	SNPRC			
12427	*Papio*	*hybrid*	United States of /	SNPRC	Kendall et al., 2024	SNPRC			
12457	*Papio*	*hybrid*	United States of /	SNPRC	Kendall et al., 2024	SNPRC			
12473	*Papio*	*hybrid*	United States of /	SNPRC	Kendall et al., 2024	SNPRC			
12481	*Papio*	*hybrid*	United States of /	SNPRC	Kendall et al., 2024	SNPRC			
12491	*Papio*	*hybrid*	United States of /	SNPRC	Kendall et al., 2024	SNPRC			
12552	*Papio*	*hybrid*	United States of /	SNPRC	Kendall et al., 2024	SNPRC			
12656	*Papio*	*hybrid*	United States of /	SNPRC	Kendall et al., 2024	SNPRC			
12681	*Papio*	*hybrid*	United States of /	SNPRC	Kendall et al., 2024	SNPRC			
12717	*Papio*	*hybrid*	United States of /	SNPRC	Kendall et al., 2024	SNPRC			
12719	*Papio*	*hybrid*	United States of /	SNPRC	Kendall et al., 2024	SNPRC			
12720	*Papio*	*hybrid*	United States of /	SNPRC	Kendall et al., 2024	SNPRC			
12869	*Papio*	*hybrid*	United States of /	SNPRC	Kendall et al., 2024	SNPRC			
12962	*Papio*	*hybrid*	United States of /	SNPRC	Kendall et al., 2024	SNPRC			
12963	*Papio*	*hybrid*	United States of /	SNPRC	Kendall et al., 2024	SNPRC			
13068	*Papio*	*hybrid*	United States of /	SNPRC	Kendall et al., 2024	SNPRC			
13103	*Papio*	*hybrid*	United States of /	SNPRC	Kendall et al., 2024	SNPRC			
13110	*Papio*	*hybrid*	United States of /	SNPRC	Kendall et al., 2024	SNPRC			
13152	*Papio*	*hybrid*	United States of /	SNPRC	Kendall et al., 2024	SNPRC			
13169	*Papio*	*hybrid*	United States of /	SNPRC	Kendall et al., 2024	SNPRC			
13225	*Papio*	*hybrid*	United States of /	SNPRC	Kendall et al., 2024	SNPRC			
13226	*Papio*	*hybrid*	United States of /	SNPRC	Kendall et al., 2024	SNPRC			
13228	*Papio*	*hybrid*	United States of /	SNPRC	Kendall et al., 2024	SNPRC			
13245	*Papio*	*hybrid*	United States of /	SNPRC	Kendall et al., 2024	SNPRC			
13264	*Papio*	*hybrid*	United States of /	SNPRC	Kendall et al., 2024	SNPRC			
13387	*Papio*	*hybrid*	United States of /	SNPRC	Kendall et al., 2024	SNPRC			
13463	*Papio*	*hybrid*	United States of /	SNPRC	Kendall et al., 2024	SNPRC			
13570	*Papio*	*hybrid*	United States of /	SNPRC	Kendall et al., 2024	SNPRC			
13575	*Papio*	*hybrid*	United States of /	SNPRC	Kendall et al., 2024	SNPRC			
13589	*Papio*	*hybrid*	United States of /	SNPRC	Kendall et al., 2024	SNPRC			
13597	*Papio*	*hybrid*	United States of /	SNPRC	Kendall et al., 2024	SNPRC			
13644	*Papio*	*hybrid*	United States of /	SNPRC	Kendall et al., 2024	SNPRC			
13645	*Papio*	*hybrid*	United States of /	SNPRC	Kendall et al., 2024	SNPRC			
13673	*Papio*	*hybrid*	United States of /	SNPRC	Kendall et al., 2024	SNPRC			
13694	*Papio*	*hybrid*	United States of /	SNPRC	Kendall et al., 2024	SNPRC			
13698	*Papio*	*hybrid*	United States of /	SNPRC	Kendall et al., 2024	SNPRC			
13739	*Papio*	*hybrid*	United States of /	SNPRC	Kendall et al., 2024	SNPRC			
13859	*Papio*	*hybrid*	United States of /	SNPRC	Kendall et al., 2024	SNPRC			
13914	*Papio*	*hybrid*	United States of /	SNPRC	Kendall et al., 2024	SNPRC			
13942	*Papio*	*hybrid*	United States of /	SNPRC	Kendall et al., 2024	SNPRC			
13951	*Papio*	*hybrid*	United States of /	SNPRC	Kendall et al., 2024	SNPRC			
13988	*Papio*	*hybrid*	United States of /	SNPRC	Kendall et al., 2024	SNPRC			
14012	*Papio*	*hybrid*	United States of /	SNPRC	Kendall et al., 2024	SNPRC			
14013	*Papio*	*hybrid*	United States of /	SNPRC	Kendall et al., 2024	SNPRC			
14016	*Papio*	*hybrid*	United States of /	SNPRC	Kendall et al., 2024	SNPRC			
14022	*Papio*	*hybrid*	United States of /	SNPRC	Kendall et al., 2024	SNPRC			
14058	*Papio*	*hybrid*	United States of /	SNPRC	Kendall et al., 2024	SNPRC			
14062	*Papio*	*hybrid*	United States of /	SNPRC	Kendall et al., 2024	SNPRC			
14066	*Papio*	*hybrid*	United States of /	SNPRC	Kendall et al., 2024	SNPRC			
14068	*Papio*	*hybrid*	United States of /	SNPRC	Kendall et al., 2024	SNPRC			

Sample	Genus	Species	Country	Locality	Source	Dataset	Note		
14076	*Papio*	*hybrid*	United States of /	SNPRC	Kendall et al., 2024	SNPRC			
14077	*Papio*	*hybrid*	United States of /	SNPRC	Kendall et al., 2024	SNPRC			
14154	*Papio*	*hybrid*	United States of /	SNPRC	Kendall et al., 2024	SNPRC			
14158	*Papio*	*hybrid*	United States of /	SNPRC	Kendall et al., 2024	SNPRC			
14167	*Papio*	*hybrid*	United States of /	SNPRC	Kendall et al., 2024	SNPRC			
14172	*Papio*	*hybrid*	United States of /	SNPRC	Kendall et al., 2024	SNPRC			
14181	*Papio*	*hybrid*	United States of /	SNPRC	Kendall et al., 2024	SNPRC			
14182	*Papio*	*hybrid*	United States of /	SNPRC	Kendall et al., 2024	SNPRC			
14191	*Papio*	*hybrid*	United States of /	SNPRC	Kendall et al., 2024	SNPRC			
14204	*Papio*	*hybrid*	United States of /	SNPRC	Kendall et al., 2024	SNPRC			
14250	*Papio*	*hybrid*	United States of /	SNPRC	Kendall et al., 2024	SNPRC			
14273	*Papio*	*hybrid*	United States of /	SNPRC	Kendall et al., 2024	SNPRC			
14276	*Papio*	*hybrid*	United States of /	SNPRC	Kendall et al., 2024	SNPRC			
14282	*Papio*	*hybrid*	United States of /	SNPRC	Kendall et al., 2024	SNPRC			
14290	*Papio*	*hybrid*	United States of /	SNPRC	Kendall et al., 2024	SNPRC			
14324	*Papio*	*hybrid*	United States of /	SNPRC	Kendall et al., 2024	SNPRC			
14330	*Papio*	*hybrid*	United States of /	SNPRC	Kendall et al., 2024	SNPRC			
14342	*Papio*	*hybrid*	United States of /	SNPRC	Kendall et al., 2024	SNPRC			
14343	*Papio*	*hybrid*	United States of /	SNPRC	Kendall et al., 2024	SNPRC			
14350	*Papio*	*hybrid*	United States of /	SNPRC	Kendall et al., 2024	SNPRC			
14351	*Papio*	*hybrid*	United States of /	SNPRC	Kendall et al., 2024	SNPRC			
14355	*Papio*	*hybrid*	United States of /	SNPRC	Kendall et al., 2024	SNPRC			
14369	*Papio*	*hybrid*	United States of /	SNPRC	Kendall et al., 2024	SNPRC			
14379	*Papio*	*hybrid*	United States of /	SNPRC	Kendall et al., 2024	SNPRC			
14427	*Papio*	*hybrid*	United States of /	SNPRC	Kendall et al., 2024	SNPRC			
14435	*Papio*	*hybrid*	United States of /	SNPRC	Kendall et al., 2024	SNPRC			
14437	*Papio*	*hybrid*	United States of /	SNPRC	Kendall et al., 2024	SNPRC			
14460	*Papio*	*hybrid*	United States of /	SNPRC	Kendall et al., 2024	SNPRC			
14473	*Papio*	*hybrid*	United States of /	SNPRC	Kendall et al., 2024	SNPRC			
14498	*Papio*	*hybrid*	United States of /	SNPRC	Kendall et al., 2024	SNPRC			
14526	*Papio*	*hybrid*	United States of /	SNPRC	Kendall et al., 2024	SNPRC			
14642	*Papio*	*hybrid*	United States of /	SNPRC	Kendall et al., 2024	SNPRC			
14652	*Papio*	*hybrid*	United States of /	SNPRC	Kendall et al., 2024	SNPRC			
14668	*Papio*	*hybrid*	United States of /	SNPRC	Kendall et al., 2024	SNPRC			
14690	*Papio*	*hybrid*	United States of /	SNPRC	Kendall et al., 2024	SNPRC			
14695	*Papio*	*hybrid*	United States of /	SNPRC	Kendall et al., 2024	SNPRC			
14696	*Papio*	*hybrid*	United States of /	SNPRC	Kendall et al., 2024	SNPRC			
14740	*Papio*	*hybrid*	United States of /	SNPRC	Kendall et al., 2024	SNPRC			
14753	*Papio*	*hybrid*	United States of /	SNPRC	Kendall et al., 2024	SNPRC			
14756	*Papio*	*hybrid*	United States of /	SNPRC	Kendall et al., 2024	SNPRC			
14795	*Papio*	*hybrid*	United States of /	SNPRC	Kendall et al., 2024	SNPRC			
14800	*Papio*	*hybrid*	United States of /	SNPRC	Kendall et al., 2024	SNPRC			
14833	*Papio*	*hybrid*	United States of /	SNPRC	Kendall et al., 2024	SNPRC			
14847	*Papio*	*hybrid*	United States of /	SNPRC	Kendall et al., 2024	SNPRC			
14850	*Papio*	*hybrid*	United States of /	SNPRC	Kendall et al., 2024	SNPRC			
14852	*Papio*	*hybrid*	United States of /	SNPRC	Kendall et al., 2024	SNPRC			
14860	*Papio*	*hybrid*	United States of /	SNPRC	Kendall et al., 2024	SNPRC			
14863	*Papio*	*hybrid*	United States of /	SNPRC	Kendall et al., 2024	SNPRC			
14865	*Papio*	*hybrid*	United States of /	SNPRC	Kendall et al., 2024	SNPRC			
14867	*Papio*	*hybrid*	United States of /	SNPRC	Kendall et al., 2024	SNPRC			
14886	*Papio*	*hybrid*	United States of /	SNPRC	Kendall et al., 2024	SNPRC			
14908	*Papio*	*hybrid*	United States of /	SNPRC	Kendall et al., 2024	SNPRC			
14909	*Papio*	*hybrid*	United States of /	SNPRC	Kendall et al., 2024	SNPRC			
14916	*Papio*	*hybrid*	United States of /	SNPRC	Kendall et al., 2024	SNPRC			
14922	*Papio*	*hybrid*	United States of /	SNPRC	Kendall et al., 2024	SNPRC			
14924	*Papio*	*hybrid*	United States of /	SNPRC	Kendall et al., 2024	SNPRC			
14925	*Papio*	*hybrid*	United States of /	SNPRC	Kendall et al., 2024	SNPRC			
14930	*Papio*	*hybrid*	United States of /	SNPRC	Kendall et al., 2024	SNPRC			
14943	*Papio*	*hybrid*	United States of /	SNPRC	Kendall et al., 2024	SNPRC			
14944	*Papio*	*hybrid*	United States of /	SNPRC	Kendall et al., 2024	SNPRC			
14948	*Papio*	*hybrid*	United States of /	SNPRC	Kendall et al., 2024	SNPRC			
14951	*Papio*	*hybrid*	United States of /	SNPRC	Kendall et al., 2024	SNPRC			
14959	*Papio*	*hybrid*	United States of /	SNPRC	Kendall et al., 2024	SNPRC			
14989	*Papio*	*hybrid*	United States of /	SNPRC	Kendall et al., 2024	SNPRC			
14994	*Papio*	*hybrid*	United States of /	SNPRC	Kendall et al., 2024	SNPRC			
15009	*Papio*	*hybrid*	United States of /	SNPRC	Kendall et al., 2024	SNPRC			
15107	*Papio*	*hybrid*	United States of /	SNPRC	Kendall et al., 2024	SNPRC			
15112	*Papio*	*hybrid*	United States of /	SNPRC	Kendall et al., 2024	SNPRC			
15113	*Papio*	*hybrid*	United States of /	SNPRC	Kendall et al., 2024	SNPRC			
15122	*Papio*	*hybrid*	United States of /	SNPRC	Kendall et al., 2024	SNPRC			
15149	*Papio*	*hybrid*	United States of /	SNPRC	Kendall et al., 2024	SNPRC			
15150	*Papio*	*hybrid*	United States of /	SNPRC	Kendall et al., 2024	SNPRC			
15156	*Papio*	*hybrid*	United States of /	SNPRC	Kendall et al., 2024	SNPRC			
15157	*Papio*	*hybrid*	United States of /	SNPRC	Kendall et al., 2024	SNPRC			
15163	*Papio*	*hybrid*	United States of /	SNPRC	Kendall et al., 2024	SNPRC			

Sample	Genus	Species	Country	Locality	Source	Dataset	Note		
15175	*Papio*	*hybrid*	United States of /	SNPRC	Kendall et al., 2024	SNPRC			
15178	*Papio*	*hybrid*	United States of /	SNPRC	Kendall et al., 2024	SNPRC			
15190	*Papio*	*hybrid*	United States of /	SNPRC	Kendall et al., 2024	SNPRC			
15197	*Papio*	*hybrid*	United States of /	SNPRC	Kendall et al., 2024	SNPRC			
15211	*Papio*	*hybrid*	United States of /	SNPRC	Kendall et al., 2024	SNPRC			
15212	*Papio*	*hybrid*	United States of /	SNPRC	Kendall et al., 2024	SNPRC			
15217	*Papio*	*hybrid*	United States of /	SNPRC	Kendall et al., 2024	SNPRC			
15225	*Papio*	*hybrid*	United States of /	SNPRC	Kendall et al., 2024	SNPRC			
15232	*Papio*	*hybrid*	United States of /	SNPRC	Kendall et al., 2024	SNPRC			
15244	*Papio*	*hybrid*	United States of /	SNPRC	Kendall et al., 2024	SNPRC			
15267	*Papio*	*hybrid*	United States of /	SNPRC	Kendall et al., 2024	SNPRC			
15274	*Papio*	*hybrid*	United States of /	SNPRC	Kendall et al., 2024	SNPRC			
15279	*Papio*	*hybrid*	United States of /	SNPRC	Kendall et al., 2024	SNPRC			
15286	*Papio*	*hybrid*	United States of /	SNPRC	Kendall et al., 2024	SNPRC			
15290	*Papio*	*hybrid*	United States of /	SNPRC	Kendall et al., 2024	SNPRC			
15292	*Papio*	*hybrid*	United States of /	SNPRC	Kendall et al., 2024	SNPRC			
15308	*Papio*	*hybrid*	United States of /	SNPRC	Kendall et al., 2024	SNPRC			
15414	*Papio*	*hybrid*	United States of /	SNPRC	Kendall et al., 2024	SNPRC			
15419	*Papio*	*hybrid*	United States of /	SNPRC	Kendall et al., 2024	SNPRC			
15421	*Papio*	*hybrid*	United States of /	SNPRC	Kendall et al., 2024	SNPRC			
15442	*Papio*	*hybrid*	United States of /	SNPRC	Kendall et al., 2024	SNPRC			
15444	*Papio*	*hybrid*	United States of /	SNPRC	Kendall et al., 2024	SNPRC			
15458	*Papio*	*hybrid*	United States of /	SNPRC	Kendall et al., 2024	SNPRC			
15467	*Papio*	*hybrid*	United States of /	SNPRC	Kendall et al., 2024	SNPRC			
15475	*Papio*	*hybrid*	United States of /	SNPRC	Kendall et al., 2024	SNPRC			
15486	*Papio*	*hybrid*	United States of /	SNPRC	Kendall et al., 2024	SNPRC			
15494	*Papio*	*hybrid*	United States of /	SNPRC	Kendall et al., 2024	SNPRC			
15509	*Papio*	*hybrid*	United States of /	SNPRC	Kendall et al., 2024	SNPRC			
15515	*Papio*	*hybrid*	United States of /	SNPRC	Kendall et al., 2024	SNPRC			
15523	*Papio*	*hybrid*	United States of /	SNPRC	Kendall et al., 2024	SNPRC			
15545	*Papio*	*hybrid*	United States of /	SNPRC	Kendall et al., 2024	SNPRC			
15553	*Papio*	*hybrid*	United States of /	SNPRC	Kendall et al., 2024	SNPRC			
15560	*Papio*	*hybrid*	United States of /	SNPRC	Kendall et al., 2024	SNPRC			
15562	*Papio*	*hybrid*	United States of /	SNPRC	Kendall et al., 2024	SNPRC			
15566	*Papio*	*hybrid*	United States of /	SNPRC	Kendall et al., 2024	SNPRC			
15568	*Papio*	*hybrid*	United States of /	SNPRC	Kendall et al., 2024	SNPRC			
15571	*Papio*	*hybrid*	United States of /	SNPRC	Kendall et al., 2024	SNPRC			
15575	*Papio*	*hybrid*	United States of /	SNPRC	Kendall et al., 2024	SNPRC			
15579	*Papio*	*hybrid*	United States of /	SNPRC	Kendall et al., 2024	SNPRC			
15581	*Papio*	*hybrid*	United States of /	SNPRC	Kendall et al., 2024	SNPRC			
15584	*Papio*	*hybrid*	United States of /	SNPRC	Kendall et al., 2024	SNPRC			
15599	*Papio*	*hybrid*	United States of /	SNPRC	Kendall et al., 2024	SNPRC			
15600	*Papio*	*hybrid*	United States of /	SNPRC	Kendall et al., 2024	SNPRC			
15626	*Papio*	*hybrid*	United States of /	SNPRC	Kendall et al., 2024	SNPRC			
15628	*Papio*	*hybrid*	United States of /	SNPRC	Kendall et al., 2024	SNPRC			
15633	*Papio*	*hybrid*	United States of /	SNPRC	Kendall et al., 2024	SNPRC			
15652	*Papio*	*hybrid*	United States of /	SNPRC	Kendall et al., 2024	SNPRC			
15653	*Papio*	*hybrid*	United States of /	SNPRC	Kendall et al., 2024	SNPRC			
15659	*Papio*	*hybrid*	United States of /	SNPRC	Kendall et al., 2024	SNPRC			
15664	*Papio*	*hybrid*	United States of /	SNPRC	Kendall et al., 2024	SNPRC			
15733	*Papio*	*hybrid*	United States of /	SNPRC	Kendall et al., 2024	SNPRC			
15736	*Papio*	*hybrid*	United States of /	SNPRC	Kendall et al., 2024	SNPRC			
15820	*Papio*	*hybrid*	United States of /	SNPRC	Kendall et al., 2024	SNPRC			
15824	*Papio*	*hybrid*	United States of /	SNPRC	Kendall et al., 2024	SNPRC			
15845	*Papio*	*hybrid*	United States of /	SNPRC	Kendall et al., 2024	SNPRC			
15846	*Papio*	*hybrid*	United States of /	SNPRC	Kendall et al., 2024	SNPRC			
15849	*Papio*	*hybrid*	United States of /	SNPRC	Kendall et al., 2024	SNPRC			
15870	*Papio*	*hybrid*	United States of /	SNPRC	Kendall et al., 2024	SNPRC			
15874	*Papio*	*hybrid*	United States of /	SNPRC	Kendall et al., 2024	SNPRC			
15889	*Papio*	*hybrid*	United States of /	SNPRC	Kendall et al., 2024	SNPRC			
15938	*Papio*	*hybrid*	United States of /	SNPRC	Kendall et al., 2024	SNPRC			
15940	*Papio*	*hybrid*	United States of /	SNPRC	Kendall et al., 2024	SNPRC			
15944	*Papio*	*hybrid*	United States of /	SNPRC	Kendall et al., 2024	SNPRC			
15975	*Papio*	*hybrid*	United States of /	SNPRC	Kendall et al., 2024	SNPRC			
15976	*Papio*	*hybrid*	United States of /	SNPRC	Kendall et al., 2024	SNPRC			
15979	*Papio*	*hybrid*	United States of /	SNPRC	Kendall et al., 2024	SNPRC			
15996	*Papio*	*hybrid*	United States of /	SNPRC	Kendall et al., 2024	SNPRC			
16000	*Papio*	*hybrid*	United States of /	SNPRC	Kendall et al., 2024	SNPRC			
16006	*Papio*	*hybrid*	United States of /	SNPRC	Kendall et al., 2024	SNPRC			
16017	*Papio*	*hybrid*	United States of /	SNPRC	Kendall et al., 2024	SNPRC			
16019	*Papio*	*hybrid*	United States of /	SNPRC	Kendall et al., 2024	SNPRC			
16056	*Papio*	*hybrid*	United States of /	SNPRC	Kendall et al., 2024	SNPRC			
16058	*Papio*	*hybrid*	United States of /	SNPRC	Kendall et al., 2024	SNPRC			
16059	*Papio*	*hybrid*	United States of /	SNPRC	Kendall et al., 2024	SNPRC			
16065	*Papio*	*hybrid*	United States of /	SNPRC	Kendall et al., 2024	SNPRC			

Sample	Genus	Species	Country	Locality	Source	Dataset	Note		
16067	*Papio*	*hybrid*	United States of /	SNPRC	Kendall et al., 2024	SNPRC			
16072	*Papio*	*hybrid*	United States of /	SNPRC	Kendall et al., 2024	SNPRC			
16083	*Papio*	*hybrid*	United States of /	SNPRC	Kendall et al., 2024	SNPRC			
16092	*Papio*	*hybrid*	United States of /	SNPRC	Kendall et al., 2024	SNPRC			
16094	*Papio*	*hybrid*	United States of /	SNPRC	Kendall et al., 2024	SNPRC			
16101	*Papio*	*hybrid*	United States of /	SNPRC	Kendall et al., 2024	SNPRC			
16104	*Papio*	*hybrid*	United States of /	SNPRC	Kendall et al., 2024	SNPRC			
16112	*Papio*	*hybrid*	United States of /	SNPRC	Kendall et al., 2024	SNPRC			
16115	*Papio*	*hybrid*	United States of /	SNPRC	Kendall et al., 2024	SNPRC			
16121	*Papio*	*hybrid*	United States of /	SNPRC	Kendall et al., 2024	SNPRC			
16122	*Papio*	*hybrid*	United States of /	SNPRC	Kendall et al., 2024	SNPRC			
16243	*Papio*	*hybrid*	United States of /	SNPRC	Kendall et al., 2024	SNPRC			
16244	*Papio*	*hybrid*	United States of /	SNPRC	Kendall et al., 2024	SNPRC			
16246	*Papio*	*hybrid*	United States of /	SNPRC	Kendall et al., 2024	SNPRC			
16254	*Papio*	*hybrid*	United States of /	SNPRC	Kendall et al., 2024	SNPRC			
16261	*Papio*	*hybrid*	United States of /	SNPRC	Kendall et al., 2024	SNPRC			
16263	*Papio*	*hybrid*	United States of /	SNPRC	Kendall et al., 2024	SNPRC			
16288	*Papio*	*hybrid*	United States of /	SNPRC	Kendall et al., 2024	SNPRC			
16289	*Papio*	*hybrid*	United States of /	SNPRC	Kendall et al., 2024	SNPRC			
16300	*Papio*	*hybrid*	United States of /	SNPRC	Kendall et al., 2024	SNPRC			
16304	*Papio*	*hybrid*	United States of /	SNPRC	Kendall et al., 2024	SNPRC			
16316	*Papio*	*hybrid*	United States of /	SNPRC	Kendall et al., 2024	SNPRC			
16321	*Papio*	*hybrid*	United States of /	SNPRC	Kendall et al., 2024	SNPRC			
16329	*Papio*	*hybrid*	United States of /	SNPRC	Kendall et al., 2024	SNPRC			
16338	*Papio*	*hybrid*	United States of /	SNPRC	Kendall et al., 2024	SNPRC			
16363	*Papio*	*hybrid*	United States of /	SNPRC	Kendall et al., 2024	SNPRC			
16369	*Papio*	*hybrid*	United States of /	SNPRC	Kendall et al., 2024	SNPRC			
16371	*Papio*	*hybrid*	United States of /	SNPRC	Kendall et al., 2024	SNPRC			
16372	*Papio*	*hybrid*	United States of /	SNPRC	Kendall et al., 2024	SNPRC			
16383	*Papio*	*hybrid*	United States of /	SNPRC	Kendall et al., 2024	SNPRC			
16385	*Papio*	*hybrid*	United States of /	SNPRC	Kendall et al., 2024	SNPRC			
16386	*Papio*	*hybrid*	United States of /	SNPRC	Kendall et al., 2024	SNPRC			
16391	*Papio*	*hybrid*	United States of /	SNPRC	Kendall et al., 2024	SNPRC			
16400	*Papio*	*hybrid*	United States of /	SNPRC	Kendall et al., 2024	SNPRC			
16408	*Papio*	*hybrid*	United States of /	SNPRC	Kendall et al., 2024	SNPRC			
16409	*Papio*	*hybrid*	United States of /	SNPRC	Kendall et al., 2024	SNPRC			
16413	*Papio*	*hybrid*	United States of /	SNPRC	Kendall et al., 2024	SNPRC			
16417	*Papio*	*hybrid*	United States of /	SNPRC	Kendall et al., 2024	SNPRC			
16420	*Papio*	*hybrid*	United States of /	SNPRC	Kendall et al., 2024	SNPRC			
16424	*Papio*	*hybrid*	United States of /	SNPRC	Kendall et al., 2024	SNPRC			
16436	*Papio*	*hybrid*	United States of /	SNPRC	Kendall et al., 2024	SNPRC			
16486	*Papio*	*hybrid*	United States of /	SNPRC	Kendall et al., 2024	SNPRC			
16499	*Papio*	*hybrid*	United States of /	SNPRC	Kendall et al., 2024	SNPRC			
16507	*Papio*	*hybrid*	United States of /	SNPRC	Kendall et al., 2024	SNPRC			
16510	*Papio*	*hybrid*	United States of /	SNPRC	Kendall et al., 2024	SNPRC			
16517	*Papio*	*hybrid*	United States of /	SNPRC	Kendall et al., 2024	SNPRC			
16524	*Papio*	*hybrid*	United States of /	SNPRC	Kendall et al., 2024	SNPRC			
16527	*Papio*	*hybrid*	United States of /	SNPRC	Kendall et al., 2024	SNPRC			
16538	*Papio*	*hybrid*	United States of /	SNPRC	Kendall et al., 2024	SNPRC			
16548	*Papio*	*hybrid*	United States of /	SNPRC	Kendall et al., 2024	SNPRC			
16550	*Papio*	*hybrid*	United States of /	SNPRC	Kendall et al., 2024	SNPRC			
16562	*Papio*	*hybrid*	United States of /	SNPRC	Kendall et al., 2024	SNPRC			
16569	*Papio*	*hybrid*	United States of /	SNPRC	Kendall et al., 2024	SNPRC			
16671	*Papio*	*hybrid*	United States of /	SNPRC	Kendall et al., 2024	SNPRC			
16678	*Papio*	*hybrid*	United States of /	SNPRC	Kendall et al., 2024	SNPRC			
16692	*Papio*	*hybrid*	United States of /	SNPRC	Kendall et al., 2024	SNPRC			
16702	*Papio*	*hybrid*	United States of /	SNPRC	Kendall et al., 2024	SNPRC			
16708	*Papio*	*hybrid*	United States of /	SNPRC	Kendall et al., 2024	SNPRC			
16719	*Papio*	*hybrid*	United States of /	SNPRC	Kendall et al., 2024	SNPRC			
16731	*Papio*	*hybrid*	United States of /	SNPRC	Kendall et al., 2024	SNPRC			
16741	*Papio*	*hybrid*	United States of /	SNPRC	Kendall et al., 2024	SNPRC			
16743	*Papio*	*hybrid*	United States of /	SNPRC	Kendall et al., 2024	SNPRC			
16744	*Papio*	*hybrid*	United States of /	SNPRC	Kendall et al., 2024	SNPRC			
16748	*Papio*	*hybrid*	United States of /	SNPRC	Kendall et al., 2024	SNPRC			
16761	*Papio*	*hybrid*	United States of /	SNPRC	Kendall et al., 2024	SNPRC			
16775	*Papio*	*hybrid*	United States of /	SNPRC	Kendall et al., 2024	SNPRC			
16780	*Papio*	*hybrid*	United States of /	SNPRC	Kendall et al., 2024	SNPRC			
16787	*Papio*	*hybrid*	United States of /	SNPRC	Kendall et al., 2024	SNPRC			
16798	*Papio*	*hybrid*	United States of /	SNPRC	Kendall et al., 2024	SNPRC			
16808	*Papio*	*hybrid*	United States of /	SNPRC	Kendall et al., 2024	SNPRC			
16815	*Papio*	*hybrid*	United States of /	SNPRC	Kendall et al., 2024	SNPRC			
16816	*Papio*	*hybrid*	United States of /	SNPRC	Kendall et al., 2024	SNPRC			
16823	*Papio*	*hybrid*	United States of /	SNPRC	Kendall et al., 2024	SNPRC			
16827	*Papio*	*hybrid*	United States of /	SNPRC	Kendall et al., 2024	SNPRC			
16829	*Papio*	*hybrid*	United States of /	SNPRC	Kendall et al., 2024	SNPRC			

Sample	Genus	Species	Country	Locality	Source	Dataset	Note		
16847	*Papio*	*hybrid*	United States of /	SNPRC	Kendall et al., 2024	SNPRC			
16853	*Papio*	*hybrid*	United States of /	SNPRC	Kendall et al., 2024	SNPRC			
16856	*Papio*	*hybrid*	United States of /	SNPRC	Kendall et al., 2024	SNPRC			
16861	*Papio*	*hybrid*	United States of /	SNPRC	Kendall et al., 2024	SNPRC			
16862	*Papio*	*hybrid*	United States of /	SNPRC	Kendall et al., 2024	SNPRC			
16866	*Papio*	*hybrid*	United States of /	SNPRC	Kendall et al., 2024	SNPRC			
16873	*Papio*	*hybrid*	United States of /	SNPRC	Kendall et al., 2024	SNPRC			
16879	*Papio*	*hybrid*	United States of /	SNPRC	Kendall et al., 2024	SNPRC			
16880	*Papio*	*hybrid*	United States of /	SNPRC	Kendall et al., 2024	SNPRC			
16889	*Papio*	*hybrid*	United States of /	SNPRC	Kendall et al., 2024	SNPRC			
16890	*Papio*	*hybrid*	United States of /	SNPRC	Kendall et al., 2024	SNPRC			
16986	*Papio*	*hybrid*	United States of /	SNPRC	Kendall et al., 2024	SNPRC			
16999	*Papio*	*hybrid*	United States of /	SNPRC	Kendall et al., 2024	SNPRC			
17006	*Papio*	*hybrid*	United States of /	SNPRC	Kendall et al., 2024	SNPRC			
17024	*Papio*	*hybrid*	United States of /	SNPRC	Kendall et al., 2024	SNPRC			
17111	*Papio*	*hybrid*	United States of /	SNPRC	Kendall et al., 2024	SNPRC			
17112	*Papio*	*hybrid*	United States of /	SNPRC	Kendall et al., 2024	SNPRC			
17139	*Papio*	*hybrid*	United States of /	SNPRC	Kendall et al., 2024	SNPRC			
17141	*Papio*	*hybrid*	United States of /	SNPRC	Kendall et al., 2024	SNPRC			
17153	*Papio*	*hybrid*	United States of /	SNPRC	Kendall et al., 2024	SNPRC			
17158	*Papio*	*hybrid*	United States of /	SNPRC	Kendall et al., 2024	SNPRC			
17159	*Papio*	*hybrid*	United States of /	SNPRC	Kendall et al., 2024	SNPRC			
17165	*Papio*	*hybrid*	United States of /	SNPRC	Kendall et al., 2024	SNPRC			
17174	*Papio*	*hybrid*	United States of /	SNPRC	Kendall et al., 2024	SNPRC			
17184	*Papio*	*hybrid*	United States of /	SNPRC	Kendall et al., 2024	SNPRC			
17190	*Papio*	*hybrid*	United States of /	SNPRC	Kendall et al., 2024	SNPRC			
17199	*Papio*	*hybrid*	United States of /	SNPRC	Kendall et al., 2024	SNPRC			
17222	*Papio*	*hybrid*	United States of /	SNPRC	Kendall et al., 2024	SNPRC			
17228	*Papio*	*hybrid*	United States of /	SNPRC	Kendall et al., 2024	SNPRC			
17232	*Papio*	*hybrid*	United States of /	SNPRC	Kendall et al., 2024	SNPRC			
17233	*Papio*	*hybrid*	United States of /	SNPRC	Kendall et al., 2024	SNPRC			
17254	*Papio*	*hybrid*	United States of /	SNPRC	Kendall et al., 2024	SNPRC			
17255	*Papio*	*hybrid*	United States of /	SNPRC	Kendall et al., 2024	SNPRC			
17268	*Papio*	*hybrid*	United States of /	SNPRC	Kendall et al., 2024	SNPRC			
17294	*Papio*	*hybrid*	United States of /	SNPRC	Kendall et al., 2024	SNPRC			
17295	*Papio*	*hybrid*	United States of /	SNPRC	Kendall et al., 2024	SNPRC			
17325	*Papio*	*hybrid*	United States of /	SNPRC	Kendall et al., 2024	SNPRC			
17328	*Papio*	*hybrid*	United States of /	SNPRC	Kendall et al., 2024	SNPRC			
17340	*Papio*	*hybrid*	United States of /	SNPRC	Kendall et al., 2024	SNPRC			
17776	*Papio*	*hybrid*	United States of /	SNPRC	Kendall et al., 2024	SNPRC			
17792	*Papio*	*hybrid*	United States of /	SNPRC	Kendall et al., 2024	SNPRC			
17795	*Papio*	*hybrid*	United States of /	SNPRC	Kendall et al., 2024	SNPRC			
17826	*Papio*	*hybrid*	United States of /	SNPRC	Kendall et al., 2024	SNPRC			
17829	*Papio*	*hybrid*	United States of /	SNPRC	Kendall et al., 2024	SNPRC			
17832	*Papio*	*hybrid*	United States of /	SNPRC	Kendall et al., 2024	SNPRC			
17833	*Papio*	*hybrid*	United States of /	SNPRC	Kendall et al., 2024	SNPRC			
17840	*Papio*	*hybrid*	United States of /	SNPRC	Kendall et al., 2024	SNPRC			
17851	*Papio*	*hybrid*	United States of /	SNPRC	Kendall et al., 2024	SNPRC			
17852	*Papio*	*hybrid*	United States of /	SNPRC	Kendall et al., 2024	SNPRC			
17871	*Papio*	*hybrid*	United States of /	SNPRC	Kendall et al., 2024	SNPRC			
17873	*Papio*	*hybrid*	United States of /	SNPRC	Kendall et al., 2024	SNPRC			
17879	*Papio*	*hybrid*	United States of /	SNPRC	Kendall et al., 2024	SNPRC			
17891	*Papio*	*hybrid*	United States of /	SNPRC	Kendall et al., 2024	SNPRC			
17898	*Papio*	*hybrid*	United States of /	SNPRC	Kendall et al., 2024	SNPRC			
17903	*Papio*	*hybrid*	United States of /	SNPRC	Kendall et al., 2024	SNPRC			
17916	*Papio*	*hybrid*	United States of /	SNPRC	Kendall et al., 2024	SNPRC			
17929	*Papio*	*hybrid*	United States of /	SNPRC	Kendall et al., 2024	SNPRC			
17969	*Papio*	*hybrid*	United States of /	SNPRC	Kendall et al., 2024	SNPRC			
17970	*Papio*	*hybrid*	United States of /	SNPRC	Kendall et al., 2024	SNPRC			
17971	*Papio*	*hybrid*	United States of /	SNPRC	Kendall et al., 2024	SNPRC			
17981	*Papio*	*hybrid*	United States of /	SNPRC	Kendall et al., 2024	SNPRC			
17997	*Papio*	*hybrid*	United States of /	SNPRC	Kendall et al., 2024	SNPRC			
17998	*Papio*	*hybrid*	United States of /	SNPRC	Kendall et al., 2024	SNPRC			
17999	*Papio*	*hybrid*	United States of /	SNPRC	Kendall et al., 2024	SNPRC			
18004	*Papio*	*hybrid*	United States of /	SNPRC	Kendall et al., 2024	SNPRC			
18015	*Papio*	*hybrid*	United States of /	SNPRC	Kendall et al., 2024	SNPRC			
18019	*Papio*	*hybrid*	United States of /	SNPRC	Kendall et al., 2024	SNPRC			
18022	*Papio*	*hybrid*	United States of /	SNPRC	Kendall et al., 2024	SNPRC			
18024	*Papio*	*hybrid*	United States of /	SNPRC	Kendall et al., 2024	SNPRC			
18141	*Papio*	*hybrid*	United States of /	SNPRC	Kendall et al., 2024	SNPRC			
18144	*Papio*	*hybrid*	United States of /	SNPRC	Kendall et al., 2024	SNPRC			
18168	*Papio*	*hybrid*	United States of /	SNPRC	Kendall et al., 2024	SNPRC			
18341	*Papio*	*hybrid*	United States of /	SNPRC	Kendall et al., 2024	SNPRC			
18385	*Papio*	*hybrid*	United States of /	SNPRC	Kendall et al., 2024	SNPRC			
18395	*Papio*	*hybrid*	United States of /	SNPRC	Kendall et al., 2024	SNPRC			

Sample	Genus	Species	Country	Locality	Source	Dataset	Note
18431	*Papio*	*hybrid*	United States of	SNPRC	Kendall et al., 2024	SNPRC	
18469	*Papio*	*hybrid*	United States of	SNPRC	Kendall et al., 2024	SNPRC	
18523	*Papio*	*hybrid*	United States of	SNPRC	Kendall et al., 2024	SNPRC	
18528	*Papio*	*hybrid*	United States of	SNPRC	Kendall et al., 2024	SNPRC	
18529	*Papio*	*hybrid*	United States of	SNPRC	Kendall et al., 2024	SNPRC	
18557	*Papio*	*hybrid*	United States of	SNPRC	Kendall et al., 2024	SNPRC	
18586	*Papio*	*hybrid*	United States of	SNPRC	Kendall et al., 2024	SNPRC	
18599	*Papio*	*hybrid*	United States of	SNPRC	Kendall et al., 2024	SNPRC	
18672	*Papio*	*hybrid*	United States of	SNPRC	Kendall et al., 2024	SNPRC	
18713	*Papio*	*hybrid*	United States of	SNPRC	Kendall et al., 2024	SNPRC	
18715	*Papio*	*hybrid*	United States of	SNPRC	Kendall et al., 2024	SNPRC	
18769	*Papio*	*hybrid*	United States of	SNPRC	Kendall et al., 2024	SNPRC	
18778	*Papio*	*hybrid*	United States of	SNPRC	Kendall et al., 2024	SNPRC	
18789	*Papio*	*hybrid*	United States of	SNPRC	Kendall et al., 2024	SNPRC	
18818	*Papio*	*hybrid*	United States of	SNPRC	Kendall et al., 2024	SNPRC	
18840	*Papio*	*hybrid*	United States of	SNPRC	Kendall et al., 2024	SNPRC	
18841	*Papio*	*hybrid*	United States of	SNPRC	Kendall et al., 2024	SNPRC	
18866	*Papio*	*hybrid*	United States of	SNPRC	Kendall et al., 2024	SNPRC	
18892	*Papio*	*hybrid*	United States of	SNPRC	Kendall et al., 2024	SNPRC	
18917	*Papio*	*hybrid*	United States of	SNPRC	Kendall et al., 2024	SNPRC	
18929	*Papio*	*hybrid*	United States of	SNPRC	Kendall et al., 2024	SNPRC	
18938	*Papio*	*hybrid*	United States of	SNPRC	Kendall et al., 2024	SNPRC	
18972	*Papio*	*hybrid*	United States of	SNPRC	Kendall et al., 2024	SNPRC	
18974	*Papio*	*hybrid*	United States of	SNPRC	Kendall et al., 2024	SNPRC	
19012	*Papio*	*hybrid*	United States of	SNPRC	Kendall et al., 2024	SNPRC	
19144	*Papio*	*hybrid*	United States of	SNPRC	Kendall et al., 2024	SNPRC	
19154	*Papio*	*hybrid*	United States of	SNPRC	Kendall et al., 2024	SNPRC	
19165	*Papio*	*hybrid*	United States of	SNPRC	Kendall et al., 2024	SNPRC	
19173	*Papio*	*hybrid*	United States of	SNPRC	Kendall et al., 2024	SNPRC	
19181	*Papio*	*hybrid*	United States of	SNPRC	Kendall et al., 2024	SNPRC	
19207	*Papio*	*hybrid*	United States of	SNPRC	Kendall et al., 2024	SNPRC	
19215	*Papio*	*hybrid*	United States of	SNPRC	Kendall et al., 2024	SNPRC	
19237	*Papio*	*hybrid*	United States of	SNPRC	Kendall et al., 2024	SNPRC	
19348	*Papio*	*hybrid*	United States of	SNPRC	Kendall et al., 2024	SNPRC	
19359	*Papio*	*hybrid*	United States of	SNPRC	Kendall et al., 2024	SNPRC	
19365	*Papio*	*hybrid*	United States of	SNPRC	Kendall et al., 2024	SNPRC	
19371	*Papio*	*hybrid*	United States of	SNPRC	Kendall et al., 2024	SNPRC	
19376	*Papio*	*hybrid*	United States of	SNPRC	Kendall et al., 2024	SNPRC	
19378	*Papio*	*hybrid*	United States of	SNPRC	Kendall et al., 2024	SNPRC	
19381	*Papio*	*hybrid*	United States of	SNPRC	Kendall et al., 2024	SNPRC	
19658	*Papio*	*hybrid*	United States of	SNPRC	Kendall et al., 2024	SNPRC	
19674	*Papio*	*hybrid*	United States of	SNPRC	Kendall et al., 2024	SNPRC	
19679	*Papio*	*hybrid*	United States of	SNPRC	Kendall et al., 2024	SNPRC	
19750	*Papio*	*hybrid*	United States of	SNPRC	Kendall et al., 2024	SNPRC	
1X0830	*Papio*	*hybrid*	United States of	SNPRC	Kendall et al., 2024	SNPRC	
1X1155	*Papio*	*hybrid*	United States of	SNPRC	Kendall et al., 2024	SNPRC	
1X1693	*Papio*	*hybrid*	United States of	SNPRC	Kendall et al., 2024	SNPRC	
1X2124	*Papio*	*hybrid*	United States of	SNPRC	Kendall et al., 2024	SNPRC	
1X2231	*Papio*	*hybrid*	United States of	SNPRC	Kendall et al., 2024	SNPRC	
1X2816	*Papio*	*hybrid*	United States of	SNPRC	Kendall et al., 2024	SNPRC	
1X2892	*Papio*	*hybrid*	United States of	SNPRC	Kendall et al., 2024	SNPRC	
1X3656	*Papio*	*hybrid*	United States of	SNPRC	Kendall et al., 2024	SNPRC	
1X3697	*Papio*	*hybrid*	United States of	SNPRC	Kendall et al., 2024	SNPRC	
1X3796	*Papio*	*hybrid*	United States of	SNPRC	Kendall et al., 2024	SNPRC	
1X3822	*Papio*	*hybrid*	United States of	SNPRC	Kendall et al., 2024	SNPRC	
1X3837	*Papio*	*hybrid*	United States of	SNPRC	Kendall et al., 2024	SNPRC	
1X4080	*Papio*	*hybrid*	United States of	SNPRC	Kendall et al., 2024	SNPRC	
1X4179	*Papio*	*hybrid*	United States of	SNPRC	Kendall et al., 2024	SNPRC	
1X4209	*Papio*	*hybrid*	United States of	SNPRC	Kendall et al., 2024	SNPRC	
1X4696	*Papio*	*hybrid*	United States of	SNPRC	Kendall et al., 2024	SNPRC	
1X4739	*Papio*	*hybrid*	United States of	SNPRC	Kendall et al., 2024	SNPRC	
1X4777	*Papio*	*hybrid*	United States of	SNPRC	Kendall et al., 2024	SNPRC	
1X4859	*Papio*	*hybrid*	United States of	SNPRC	Kendall et al., 2024	SNPRC	
20117	*Papio*	*hybrid*	United States of	SNPRC	Kendall et al., 2024	SNPRC	
25347	*Papio*	*hybrid*	United States of	SNPRC	Kendall et al., 2024	SNPRC	
25354	*Papio*	*hybrid*	United States of	SNPRC	Kendall et al., 2024	SNPRC	
25355	*Papio*	*hybrid*	United States of	SNPRC	Kendall et al., 2024	SNPRC	
25409	*Papio*	*hybrid*	United States of	SNPRC	Kendall et al., 2024	SNPRC	
25593	*Papio*	*hybrid*	United States of	SNPRC	Kendall et al., 2024	SNPRC	
26101	*Papio*	*hybrid*	United States of	SNPRC	Kendall et al., 2024	SNPRC	
26138	*Papio*	*hybrid*	United States of	SNPRC	Kendall et al., 2024	SNPRC	
26196	*Papio*	*hybrid*	United States of	SNPRC	Kendall et al., 2024	SNPRC	
26198	*Papio*	*hybrid*	United States of	SNPRC	Kendall et al., 2024	SNPRC	
26310	*Papio*	*hybrid*	United States of	SNPRC	Kendall et al., 2024	SNPRC	
26316	*Papio*	*hybrid*	United States of	SNPRC	Kendall et al., 2024	SNPRC	

Sample	Genus	Species	Country	Locality	Source	Dataset	Note
26318	*Papio*	*hybrid*	United States of /	SNPRC	Kendall et al., 2024	SNPRC	
26324	*Papio*	*hybrid*	United States of /	SNPRC	Kendall et al., 2024	SNPRC	
26355	*Papio*	*hybrid*	United States of /	SNPRC	Kendall et al., 2024	SNPRC	
26498	*Papio*	*hybrid*	United States of /	SNPRC	Kendall et al., 2024	SNPRC	
26800	*Papio*	*hybrid*	United States of /	SNPRC	Kendall et al., 2024	SNPRC	
26980	*Papio*	*hybrid*	United States of /	SNPRC	Kendall et al., 2024	SNPRC	
26988	*Papio*	*hybrid*	United States of /	SNPRC	Kendall et al., 2024	SNPRC	
27033	*Papio*	*hybrid*	United States of /	SNPRC	Kendall et al., 2024	SNPRC	
27036	*Papio*	*hybrid*	United States of /	SNPRC	Kendall et al., 2024	SNPRC	
27166	*Papio*	*hybrid*	United States of /	SNPRC	Kendall et al., 2024	SNPRC	
27181	*Papio*	*hybrid*	United States of /	SNPRC	Kendall et al., 2024	SNPRC	
27190	*Papio*	*hybrid*	United States of /	SNPRC	Kendall et al., 2024	SNPRC	
27193	*Papio*	*hybrid*	United States of /	SNPRC	Kendall et al., 2024	SNPRC	
27199	*Papio*	*hybrid*	United States of /	SNPRC	Kendall et al., 2024	SNPRC	
27230	*Papio*	*hybrid*	United States of /	SNPRC	Kendall et al., 2024	SNPRC	
27237	*Papio*	*hybrid*	United States of /	SNPRC	Kendall et al., 2024	SNPRC	
27267	*Papio*	*hybrid*	United States of /	SNPRC	Kendall et al., 2024	SNPRC	
27306	*Papio*	*hybrid*	United States of /	SNPRC	Kendall et al., 2024	SNPRC	
27351	*Papio*	*hybrid*	United States of /	SNPRC	Kendall et al., 2024	SNPRC	
27358	*Papio*	*hybrid*	United States of /	SNPRC	Kendall et al., 2024	SNPRC	
27433	*Papio*	*hybrid*	United States of /	SNPRC	Kendall et al., 2024	SNPRC	
27472	*Papio*	*hybrid*	United States of /	SNPRC	Kendall et al., 2024	SNPRC	
27503	*Papio*	*hybrid*	United States of /	SNPRC	Kendall et al., 2024	SNPRC	
27525	*Papio*	*hybrid*	United States of /	SNPRC	Kendall et al., 2024	SNPRC	
27592	*Papio*	*hybrid*	United States of /	SNPRC	Kendall et al., 2024	SNPRC	
27594	*Papio*	*hybrid*	United States of /	SNPRC	Kendall et al., 2024	SNPRC	
27622	*Papio*	*hybrid*	United States of /	SNPRC	Kendall et al., 2024	SNPRC	
27643	*Papio*	*hybrid*	United States of /	SNPRC	Kendall et al., 2024	SNPRC	
27666	*Papio*	*hybrid*	United States of /	SNPRC	Kendall et al., 2024	SNPRC	
27668	*Papio*	*hybrid*	United States of /	SNPRC	Kendall et al., 2024	SNPRC	
27686	*Papio*	*hybrid*	United States of /	SNPRC	Kendall et al., 2024	SNPRC	
27742	*Papio*	*hybrid*	United States of /	SNPRC	Kendall et al., 2024	SNPRC	
27811	*Papio*	*hybrid*	United States of /	SNPRC	Kendall et al., 2024	SNPRC	
27814	*Papio*	*hybrid*	United States of /	SNPRC	Kendall et al., 2024	SNPRC	
27820	*Papio*	*hybrid*	United States of /	SNPRC	Kendall et al., 2024	SNPRC	
27827	*Papio*	*hybrid*	United States of /	SNPRC	Kendall et al., 2024	SNPRC	
27841	*Papio*	*hybrid*	United States of /	SNPRC	Kendall et al., 2024	SNPRC	
27844	*Papio*	*hybrid*	United States of /	SNPRC	Kendall et al., 2024	SNPRC	
27874	*Papio*	*hybrid*	United States of /	SNPRC	Kendall et al., 2024	SNPRC	
27885	*Papio*	*hybrid*	United States of /	SNPRC	Kendall et al., 2024	SNPRC	
27889	*Papio*	*hybrid*	United States of /	SNPRC	Kendall et al., 2024	SNPRC	
27908	*Papio*	*hybrid*	United States of /	SNPRC	Kendall et al., 2024	SNPRC	
27985	*Papio*	*hybrid*	United States of /	SNPRC	Kendall et al., 2024	SNPRC	
27989	*Papio*	*hybrid*	United States of /	SNPRC	Kendall et al., 2024	SNPRC	
27999	*Papio*	*hybrid*	United States of /	SNPRC	Kendall et al., 2024	SNPRC	
28003	*Papio*	*hybrid*	United States of /	SNPRC	Kendall et al., 2024	SNPRC	
28021	*Papio*	*hybrid*	United States of /	SNPRC	Kendall et al., 2024	SNPRC	
28037	*Papio*	*hybrid*	United States of /	SNPRC	Kendall et al., 2024	SNPRC	
28074	*Papio*	*hybrid*	United States of /	SNPRC	Kendall et al., 2024	SNPRC	
28075	*Papio*	*hybrid*	United States of /	SNPRC	Kendall et al., 2024	SNPRC	
28076	*Papio*	*hybrid*	United States of /	SNPRC	Kendall et al., 2024	SNPRC	
28102	*Papio*	*hybrid*	United States of /	SNPRC	Kendall et al., 2024	SNPRC	
28183	*Papio*	*hybrid*	United States of /	SNPRC	Kendall et al., 2024	SNPRC	
28212	*Papio*	*hybrid*	United States of /	SNPRC	Kendall et al., 2024	SNPRC	
28246	*Papio*	*hybrid*	United States of /	SNPRC	Kendall et al., 2024	SNPRC	
28259	*Papio*	*hybrid*	United States of /	SNPRC	Kendall et al., 2024	SNPRC	
28269	*Papio*	*hybrid*	United States of /	SNPRC	Kendall et al., 2024	SNPRC	
28274	*Papio*	*hybrid*	United States of /	SNPRC	Kendall et al., 2024	SNPRC	
28279	*Papio*	*hybrid*	United States of /	SNPRC	Kendall et al., 2024	SNPRC	
28281	*Papio*	*hybrid*	United States of /	SNPRC	Kendall et al., 2024	SNPRC	
28285	*Papio*	*hybrid*	United States of /	SNPRC	Kendall et al., 2024	SNPRC	
28304	*Papio*	*hybrid*	United States of /	SNPRC	Kendall et al., 2024	SNPRC	
28328	*Papio*	*hybrid*	United States of /	SNPRC	Kendall et al., 2024	SNPRC	
28368	*Papio*	*hybrid*	United States of /	SNPRC	Kendall et al., 2024	SNPRC	
28375	*Papio*	*hybrid*	United States of /	SNPRC	Kendall et al., 2024	SNPRC	
28376	*Papio*	*hybrid*	United States of /	SNPRC	Kendall et al., 2024	SNPRC	
28385	*Papio*	*hybrid*	United States of /	SNPRC	Kendall et al., 2024	SNPRC	
28386	*Papio*	*hybrid*	United States of /	SNPRC	Kendall et al., 2024	SNPRC	
28400	*Papio*	*hybrid*	United States of /	SNPRC	Kendall et al., 2024	SNPRC	
28416	*Papio*	*hybrid*	United States of /	SNPRC	Kendall et al., 2024	SNPRC	
28417	*Papio*	*hybrid*	United States of /	SNPRC	Kendall et al., 2024	SNPRC	
28428	*Papio*	*hybrid*	United States of /	SNPRC	Kendall et al., 2024	SNPRC	
28431	*Papio*	*hybrid*	United States of /	SNPRC	Kendall et al., 2024	SNPRC	
28436	*Papio*	*hybrid*	United States of /	SNPRC	Kendall et al., 2024	SNPRC	
28438	*Papio*	*hybrid*	United States of /	SNPRC	Kendall et al., 2024	SNPRC	

Sample	Genus	Species	Country	Locality	Source	Dataset	Note		
28478	*Papio*	*hybrid*	United States of /	SNPRC	Kendall et al., 2024	SNPRC			
28495	*Papio*	*hybrid*	United States of /	SNPRC	Kendall et al., 2024	SNPRC			
28497	*Papio*	*hybrid*	United States of /	SNPRC	Kendall et al., 2024	SNPRC			
28576	*Papio*	*hybrid*	United States of /	SNPRC	Kendall et al., 2024	SNPRC			
28591	*Papio*	*hybrid*	United States of /	SNPRC	Kendall et al., 2024	SNPRC			
28609	*Papio*	*hybrid*	United States of /	SNPRC	Kendall et al., 2024	SNPRC			
28618	*Papio*	*hybrid*	United States of /	SNPRC	Kendall et al., 2024	SNPRC			
28635	*Papio*	*hybrid*	United States of /	SNPRC	Kendall et al., 2024	SNPRC			
28729	*Papio*	*hybrid*	United States of /	SNPRC	Kendall et al., 2024	SNPRC			
28740	*Papio*	*hybrid*	United States of /	SNPRC	Kendall et al., 2024	SNPRC			
28742	*Papio*	*hybrid*	United States of /	SNPRC	Kendall et al., 2024	SNPRC			
28779	*Papio*	*hybrid*	United States of /	SNPRC	Kendall et al., 2024	SNPRC			
28799	*Papio*	*hybrid*	United States of /	SNPRC	Kendall et al., 2024	SNPRC			
28801	*Papio*	*hybrid*	United States of /	SNPRC	Kendall et al., 2024	SNPRC			
28802	*Papio*	*hybrid*	United States of /	SNPRC	Kendall et al., 2024	SNPRC			
28806	*Papio*	*hybrid*	United States of /	SNPRC	Kendall et al., 2024	SNPRC			
28830	*Papio*	*hybrid*	United States of /	SNPRC	Kendall et al., 2024	SNPRC			
28925	*Papio*	*hybrid*	United States of /	SNPRC	Kendall et al., 2024	SNPRC			
28949	*Papio*	*hybrid*	United States of /	SNPRC	Kendall et al., 2024	SNPRC			
29156	*Papio*	*hybrid*	United States of /	SNPRC	Kendall et al., 2024	SNPRC			
29333	*Papio*	*hybrid*	United States of /	SNPRC	Kendall et al., 2024	SNPRC			
29368	*Papio*	*hybrid*	United States of /	SNPRC	Kendall et al., 2024	SNPRC			
29565	*Papio*	*hybrid*	United States of /	SNPRC	Kendall et al., 2024	SNPRC			
29758	*Papio*	*hybrid*	United States of /	SNPRC	Kendall et al., 2024	SNPRC			
30008	*Papio*	*hybrid*	United States of /	SNPRC	Kendall et al., 2024	SNPRC			
30033	*Papio*	*hybrid*	United States of /	SNPRC	Kendall et al., 2024	SNPRC			
30104	*Papio*	*hybrid*	United States of /	SNPRC	Kendall et al., 2024	SNPRC			
30320	*Papio*	*hybrid*	United States of /	SNPRC	Kendall et al., 2024	SNPRC			
30366	*Papio*	*hybrid*	United States of /	SNPRC	Kendall et al., 2024	SNPRC			
30373	*Papio*	*hybrid*	United States of /	SNPRC	Kendall et al., 2024	SNPRC			
30424	*Papio*	*hybrid*	United States of /	SNPRC	Kendall et al., 2024	SNPRC			
30508	*Papio*	*hybrid*	United States of /	SNPRC	Kendall et al., 2024	SNPRC			
30564	*Papio*	*hybrid*	United States of /	SNPRC	Kendall et al., 2024	SNPRC			
30609	*Papio*	*hybrid*	United States of /	SNPRC	Kendall et al., 2024	SNPRC			
30611	*Papio*	*hybrid*	United States of /	SNPRC	Kendall et al., 2024	SNPRC			
30614	*Papio*	*hybrid*	United States of /	SNPRC	Kendall et al., 2024	SNPRC			
30636	*Papio*	*hybrid*	United States of /	SNPRC	Kendall et al., 2024	SNPRC			
30650	*Papio*	*hybrid*	United States of /	SNPRC	Kendall et al., 2024	SNPRC			
30660	*Papio*	*hybrid*	United States of /	SNPRC	Kendall et al., 2024	SNPRC			
30662	*Papio*	*hybrid*	United States of /	SNPRC	Kendall et al., 2024	SNPRC			
30673	*Papio*	*hybrid*	United States of /	SNPRC	Kendall et al., 2024	SNPRC			
30699	*Papio*	*hybrid*	United States of /	SNPRC	Kendall et al., 2024	SNPRC			
30749	*Papio*	*hybrid*	United States of /	SNPRC	Kendall et al., 2024	SNPRC			
30751	*Papio*	*hybrid*	United States of /	SNPRC	Kendall et al., 2024	SNPRC			
30752	*Papio*	*hybrid*	United States of /	SNPRC	Kendall et al., 2024	SNPRC			
30772	*Papio*	*hybrid*	United States of /	SNPRC	Kendall et al., 2024	SNPRC			
30871	*Papio*	*hybrid*	United States of /	SNPRC	Kendall et al., 2024	SNPRC			
30878	*Papio*	*hybrid*	United States of /	SNPRC	Kendall et al., 2024	SNPRC			
30886	*Papio*	*hybrid*	United States of /	SNPRC	Kendall et al., 2024	SNPRC			
30889	*Papio*	*hybrid*	United States of /	SNPRC	Kendall et al., 2024	SNPRC			
30907	*Papio*	*hybrid*	United States of /	SNPRC	Kendall et al., 2024	SNPRC			
30917	*Papio*	*hybrid*	United States of /	SNPRC	Kendall et al., 2024	SNPRC			
30920	*Papio*	*hybrid*	United States of /	SNPRC	Kendall et al., 2024	SNPRC			
30927	*Papio*	*hybrid*	United States of /	SNPRC	Kendall et al., 2024	SNPRC			
30933	*Papio*	*hybrid*	United States of /	SNPRC	Kendall et al., 2024	SNPRC			
30934	*Papio*	*hybrid*	United States of /	SNPRC	Kendall et al., 2024	SNPRC			
30961	*Papio*	*hybrid*	United States of /	SNPRC	Kendall et al., 2024	SNPRC			
30973	*Papio*	*hybrid*	United States of /	SNPRC	Kendall et al., 2024	SNPRC			
30974	*Papio*	*hybrid*	United States of /	SNPRC	Kendall et al., 2024	SNPRC			
31011	*Papio*	*hybrid*	United States of /	SNPRC	Kendall et al., 2024	SNPRC			
31015	*Papio*	*hybrid*	United States of /	SNPRC	Kendall et al., 2024	SNPRC			
31016	*Papio*	*hybrid*	United States of /	SNPRC	Kendall et al., 2024	SNPRC			
31021	*Papio*	*hybrid*	United States of /	SNPRC	Kendall et al., 2024	SNPRC			
31025	*Papio*	*hybrid*	United States of /	SNPRC	Kendall et al., 2024	SNPRC			
31026	*Papio*	*hybrid*	United States of /	SNPRC	Kendall et al., 2024	SNPRC			
31028	*Papio*	*hybrid*	United States of /	SNPRC	Kendall et al., 2024	SNPRC			
31035	*Papio*	*hybrid*	United States of /	SNPRC	Kendall et al., 2024	SNPRC			
31064	*Papio*	*hybrid*	United States of /	SNPRC	Kendall et al., 2024	SNPRC			
31065	*Papio*	*hybrid*	United States of /	SNPRC	Kendall et al., 2024	SNPRC			
31072	*Papio*	*hybrid*	United States of /	SNPRC	Kendall et al., 2024	SNPRC			
31086	*Papio*	*hybrid*	United States of /	SNPRC	Kendall et al., 2024	SNPRC			
31110	*Papio*	*hybrid*	United States of /	SNPRC	Kendall et al., 2024	SNPRC			
31115	*Papio*	*hybrid*	United States of /	SNPRC	Kendall et al., 2024	SNPRC			
31120	*Papio*	*hybrid*	United States of /	SNPRC	Kendall et al., 2024	SNPRC			
31124	*Papio*	*hybrid*	United States of /	SNPRC	Kendall et al., 2024	SNPRC			

Sample	Genus	Species	Country	Locality	Source	Dataset	Note		
31130	*Papio*	*hybrid*	United States of /	SNPRC	Kendall et al., 2024	SNPRC			
31131	*Papio*	*hybrid*	United States of /	SNPRC	Kendall et al., 2024	SNPRC			
31134	*Papio*	*hybrid*	United States of /	SNPRC	Kendall et al., 2024	SNPRC			
31145	*Papio*	*hybrid*	United States of /	SNPRC	Kendall et al., 2024	SNPRC			
31152	*Papio*	*hybrid*	United States of /	SNPRC	Kendall et al., 2024	SNPRC			
31177	*Papio*	*hybrid*	United States of /	SNPRC	Kendall et al., 2024	SNPRC			
31178	*Papio*	*hybrid*	United States of /	SNPRC	Kendall et al., 2024	SNPRC			
31234	*Papio*	*hybrid*	United States of /	SNPRC	Kendall et al., 2024	SNPRC			
31235	*Papio*	*hybrid*	United States of /	SNPRC	Kendall et al., 2024	SNPRC			
31255	*Papio*	*hybrid*	United States of /	SNPRC	Kendall et al., 2024	SNPRC			
31275	*Papio*	*hybrid*	United States of /	SNPRC	Kendall et al., 2024	SNPRC			
31284	*Papio*	*hybrid*	United States of /	SNPRC	Kendall et al., 2024	SNPRC			
31288	*Papio*	*hybrid*	United States of /	SNPRC	Kendall et al., 2024	SNPRC			
31297	*Papio*	*hybrid*	United States of /	SNPRC	Kendall et al., 2024	SNPRC			
31299	*Papio*	*hybrid*	United States of /	SNPRC	Kendall et al., 2024	SNPRC			
31300	*Papio*	*hybrid*	United States of /	SNPRC	Kendall et al., 2024	SNPRC			
31311	*Papio*	*hybrid*	United States of /	SNPRC	Kendall et al., 2024	SNPRC			
31327	*Papio*	*hybrid*	United States of /	SNPRC	Kendall et al., 2024	SNPRC			
31329	*Papio*	*hybrid*	United States of /	SNPRC	Kendall et al., 2024	SNPRC			
31331	*Papio*	*hybrid*	United States of /	SNPRC	Kendall et al., 2024	SNPRC			
31333	*Papio*	*hybrid*	United States of /	SNPRC	Kendall et al., 2024	SNPRC			
31337	*Papio*	*hybrid*	United States of /	SNPRC	Kendall et al., 2024	SNPRC			
31358	*Papio*	*hybrid*	United States of /	SNPRC	Kendall et al., 2024	SNPRC			
31364	*Papio*	*hybrid*	United States of /	SNPRC	Kendall et al., 2024	SNPRC			
31392	*Papio*	*hybrid*	United States of /	SNPRC	Kendall et al., 2024	SNPRC			
31398	*Papio*	*hybrid*	United States of /	SNPRC	Kendall et al., 2024	SNPRC			
31401	*Papio*	*hybrid*	United States of /	SNPRC	Kendall et al., 2024	SNPRC			
31407	*Papio*	*hybrid*	United States of /	SNPRC	Kendall et al., 2024	SNPRC			
31408	*Papio*	*hybrid*	United States of /	SNPRC	Kendall et al., 2024	SNPRC			
31409	*Papio*	*hybrid*	United States of /	SNPRC	Kendall et al., 2024	SNPRC			
31410	*Papio*	*hybrid*	United States of /	SNPRC	Kendall et al., 2024	SNPRC			
31415	*Papio*	*hybrid*	United States of /	SNPRC	Kendall et al., 2024	SNPRC			
31417	*Papio*	*hybrid*	United States of /	SNPRC	Kendall et al., 2024	SNPRC			
31440	*Papio*	*hybrid*	United States of /	SNPRC	Kendall et al., 2024	SNPRC			
31457	*Papio*	*hybrid*	United States of /	SNPRC	Kendall et al., 2024	SNPRC			
31462	*Papio*	*hybrid*	United States of /	SNPRC	Kendall et al., 2024	SNPRC			
31469	*Papio*	*hybrid*	United States of /	SNPRC	Kendall et al., 2024	SNPRC			
31471	*Papio*	*hybrid*	United States of /	SNPRC	Kendall et al., 2024	SNPRC			
31475	*Papio*	*hybrid*	United States of /	SNPRC	Kendall et al., 2024	SNPRC			
31477	*Papio*	*hybrid*	United States of /	SNPRC	Kendall et al., 2024	SNPRC			
31482	*Papio*	*hybrid*	United States of /	SNPRC	Kendall et al., 2024	SNPRC			
31484	*Papio*	*hybrid*	United States of /	SNPRC	Kendall et al., 2024	SNPRC			
31490	*Papio*	*hybrid*	United States of /	SNPRC	Kendall et al., 2024	SNPRC			
31497	*Papio*	*hybrid*	United States of /	SNPRC	Kendall et al., 2024	SNPRC			
31498	*Papio*	*hybrid*	United States of /	SNPRC	Kendall et al., 2024	SNPRC			
31499	*Papio*	*hybrid*	United States of /	SNPRC	Kendall et al., 2024	SNPRC			
31500	*Papio*	*hybrid*	United States of /	SNPRC	Kendall et al., 2024	SNPRC			
31506	*Papio*	*hybrid*	United States of /	SNPRC	Kendall et al., 2024	SNPRC			
31507	*Papio*	*hybrid*	United States of /	SNPRC	Kendall et al., 2024	SNPRC			
31511	*Papio*	*hybrid*	United States of /	SNPRC	Kendall et al., 2024	SNPRC			
31515	*Papio*	*hybrid*	United States of /	SNPRC	Kendall et al., 2024	SNPRC			
31518	*Papio*	*hybrid*	United States of /	SNPRC	Kendall et al., 2024	SNPRC			
31525	*Papio*	*hybrid*	United States of /	SNPRC	Kendall et al., 2024	SNPRC			
31526	*Papio*	*hybrid*	United States of /	SNPRC	Kendall et al., 2024	SNPRC			
31528	*Papio*	*hybrid*	United States of /	SNPRC	Kendall et al., 2024	SNPRC			
31540	*Papio*	*hybrid*	United States of /	SNPRC	Kendall et al., 2024	SNPRC			
31542	*Papio*	*hybrid*	United States of /	SNPRC	Kendall et al., 2024	SNPRC			
31543	*Papio*	*hybrid*	United States of /	SNPRC	Kendall et al., 2024	SNPRC			
31552	*Papio*	*hybrid*	United States of /	SNPRC	Kendall et al., 2024	SNPRC			
31553	*Papio*	*hybrid*	United States of /	SNPRC	Kendall et al., 2024	SNPRC			
31556	*Papio*	*hybrid*	United States of /	SNPRC	Kendall et al., 2024	SNPRC			
31559	*Papio*	*hybrid*	United States of /	SNPRC	Kendall et al., 2024	SNPRC			
31560	*Papio*	*hybrid*	United States of /	SNPRC	Kendall et al., 2024	SNPRC			
31573	*Papio*	*hybrid*	United States of /	SNPRC	Kendall et al., 2024	SNPRC			
31576	*Papio*	*hybrid*	United States of /	SNPRC	Kendall et al., 2024	SNPRC			
31578	*Papio*	*hybrid*	United States of /	SNPRC	Kendall et al., 2024	SNPRC			
31602	*Papio*	*hybrid*	United States of /	SNPRC	Kendall et al., 2024	SNPRC			
31605	*Papio*	*hybrid*	United States of /	SNPRC	Kendall et al., 2024	SNPRC			
31624	*Papio*	*hybrid*	United States of /	SNPRC	Kendall et al., 2024	SNPRC			
31630	*Papio*	*hybrid*	United States of /	SNPRC	Kendall et al., 2024	SNPRC			
31642	*Papio*	*hybrid*	United States of /	SNPRC	Kendall et al., 2024	SNPRC			
31650	*Papio*	*hybrid*	United States of /	SNPRC	Kendall et al., 2024	SNPRC			
31656	*Papio*	*hybrid*	United States of /	SNPRC	Kendall et al., 2024	SNPRC			
31659	*Papio*	*hybrid*	United States of /	SNPRC	Kendall et al., 2024	SNPRC			
31666	*Papio*	*hybrid*	United States of /	SNPRC	Kendall et al., 2024	SNPRC			

Sample	Genus	Species	Country	Locality	Source	Dataset	Note		
31693	*Papio*	*hybrid*	United States of /	SNPRC	Kendall et al., 2024	SNPRC			
31716	*Papio*	*hybrid*	United States of /	SNPRC	Kendall et al., 2024	SNPRC			
31720	*Papio*	*hybrid*	United States of /	SNPRC	Kendall et al., 2024	SNPRC			
31729	*Papio*	*hybrid*	United States of /	SNPRC	Kendall et al., 2024	SNPRC			
31731	*Papio*	*hybrid*	United States of /	SNPRC	Kendall et al., 2024	SNPRC			
31738	*Papio*	*hybrid*	United States of /	SNPRC	Kendall et al., 2024	SNPRC			
31764	*Papio*	*hybrid*	United States of /	SNPRC	Kendall et al., 2024	SNPRC			
31778	*Papio*	*hybrid*	United States of /	SNPRC	Kendall et al., 2024	SNPRC			
31786	*Papio*	*hybrid*	United States of /	SNPRC	Kendall et al., 2024	SNPRC			
31789	*Papio*	*hybrid*	United States of /	SNPRC	Kendall et al., 2024	SNPRC			
31800	*Papio*	*hybrid*	United States of /	SNPRC	Kendall et al., 2024	SNPRC			
31804	*Papio*	*hybrid*	United States of /	SNPRC	Kendall et al., 2024	SNPRC			
31805	*Papio*	*hybrid*	United States of /	SNPRC	Kendall et al., 2024	SNPRC			
31836	*Papio*	*hybrid*	United States of /	SNPRC	Kendall et al., 2024	SNPRC			
31846	*Papio*	*hybrid*	United States of /	SNPRC	Kendall et al., 2024	SNPRC			
31849	*Papio*	*hybrid*	United States of /	SNPRC	Kendall et al., 2024	SNPRC			
31852	*Papio*	*hybrid*	United States of /	SNPRC	Kendall et al., 2024	SNPRC			
31880	*Papio*	*hybrid*	United States of /	SNPRC	Kendall et al., 2024	SNPRC			
31882	*Papio*	*hybrid*	United States of /	SNPRC	Kendall et al., 2024	SNPRC			
31902	*Papio*	*hybrid*	United States of /	SNPRC	Kendall et al., 2024	SNPRC			
31903	*Papio*	*hybrid*	United States of /	SNPRC	Kendall et al., 2024	SNPRC			
31929	*Papio*	*hybrid*	United States of /	SNPRC	Kendall et al., 2024	SNPRC			
31931	*Papio*	*hybrid*	United States of /	SNPRC	Kendall et al., 2024	SNPRC			
31937	*Papio*	*hybrid*	United States of /	SNPRC	Kendall et al., 2024	SNPRC			
31938	*Papio*	*hybrid*	United States of /	SNPRC	Kendall et al., 2024	SNPRC			
31952	*Papio*	*hybrid*	United States of /	SNPRC	Kendall et al., 2024	SNPRC			
31958	*Papio*	*hybrid*	United States of /	SNPRC	Kendall et al., 2024	SNPRC			
31960	*Papio*	*hybrid*	United States of /	SNPRC	Kendall et al., 2024	SNPRC			
31966	*Papio*	*hybrid*	United States of /	SNPRC	Kendall et al., 2024	SNPRC			
31970	*Papio*	*hybrid*	United States of /	SNPRC	Kendall et al., 2024	SNPRC			
31980	*Papio*	*hybrid*	United States of /	SNPRC	Kendall et al., 2024	SNPRC			
31984	*Papio*	*hybrid*	United States of /	SNPRC	Kendall et al., 2024	SNPRC			
31993	*Papio*	*hybrid*	United States of /	SNPRC	Kendall et al., 2024	SNPRC			
31994	*Papio*	*hybrid*	United States of /	SNPRC	Kendall et al., 2024	SNPRC			
31997	*Papio*	*hybrid*	United States of /	SNPRC	Kendall et al., 2024	SNPRC			
32025	*Papio*	*hybrid*	United States of /	SNPRC	Kendall et al., 2024	SNPRC			
32039	*Papio*	*hybrid*	United States of /	SNPRC	Kendall et al., 2024	SNPRC			
32040	*Papio*	*hybrid*	United States of /	SNPRC	Kendall et al., 2024	SNPRC			
32043	*Papio*	*hybrid*	United States of /	SNPRC	Kendall et al., 2024	SNPRC			
32053	*Papio*	*hybrid*	United States of /	SNPRC	Kendall et al., 2024	SNPRC			
32089	*Papio*	*hybrid*	United States of /	SNPRC	Kendall et al., 2024	SNPRC			
32103	*Papio*	*hybrid*	United States of /	SNPRC	Kendall et al., 2024	SNPRC			
32130	*Papio*	*hybrid*	United States of /	SNPRC	Kendall et al., 2024	SNPRC			
32132	*Papio*	*hybrid*	United States of /	SNPRC	Kendall et al., 2024	SNPRC			
32187	*Papio*	*hybrid*	United States of /	SNPRC	Kendall et al., 2024	SNPRC			
32207	*Papio*	*hybrid*	United States of /	SNPRC	Kendall et al., 2024	SNPRC			
32209	*Papio*	*hybrid*	United States of /	SNPRC	Kendall et al., 2024	SNPRC			
32215	*Papio*	*hybrid*	United States of /	SNPRC	Kendall et al., 2024	SNPRC			
32224	*Papio*	*hybrid*	United States of /	SNPRC	Kendall et al., 2024	SNPRC			
32227	*Papio*	*hybrid*	United States of /	SNPRC	Kendall et al., 2024	SNPRC			
32298	*Papio*	*hybrid*	United States of /	SNPRC	Kendall et al., 2024	SNPRC			
32299	*Papio*	*hybrid*	United States of /	SNPRC	Kendall et al., 2024	SNPRC			
32311	*Papio*	*hybrid*	United States of /	SNPRC	Kendall et al., 2024	SNPRC			
32356	*Papio*	*hybrid*	United States of /	SNPRC	Kendall et al., 2024	SNPRC			
32358	*Papio*	*hybrid*	United States of /	SNPRC	Kendall et al., 2024	SNPRC			
32363	*Papio*	*hybrid*	United States of /	SNPRC	Kendall et al., 2024	SNPRC			
32372	*Papio*	*hybrid*	United States of /	SNPRC	Kendall et al., 2024	SNPRC			
32451	*Papio*	*hybrid*	United States of /	SNPRC	Kendall et al., 2024	SNPRC			
32467	*Papio*	*hybrid*	United States of /	SNPRC	Kendall et al., 2024	SNPRC			
32469	*Papio*	*hybrid*	United States of /	SNPRC	Kendall et al., 2024	SNPRC			
32472	*Papio*	*hybrid*	United States of /	SNPRC	Kendall et al., 2024	SNPRC			
32539	*Papio*	*hybrid*	United States of /	SNPRC	Kendall et al., 2024	SNPRC			
32540	*Papio*	*hybrid*	United States of /	SNPRC	Kendall et al., 2024	SNPRC			
32552	*Papio*	*hybrid*	United States of /	SNPRC	Kendall et al., 2024	SNPRC			
32553	*Papio*	*hybrid*	United States of /	SNPRC	Kendall et al., 2024	SNPRC			
32557	*Papio*	*hybrid*	United States of /	SNPRC	Kendall et al., 2024	SNPRC			
32560	*Papio*	*hybrid*	United States of /	SNPRC	Kendall et al., 2024	SNPRC			
32588	*Papio*	*hybrid*	United States of /	SNPRC	Kendall et al., 2024	SNPRC			
32591	*Papio*	*hybrid*	United States of /	SNPRC	Kendall et al., 2024	SNPRC			
32606	*Papio*	*hybrid*	United States of /	SNPRC	Kendall et al., 2024	SNPRC			
32629	*Papio*	*hybrid*	United States of /	SNPRC	Kendall et al., 2024	SNPRC			
32651	*Papio*	*hybrid*	United States of /	SNPRC	Kendall et al., 2024	SNPRC			
32736	*Papio*	*hybrid*	United States of /	SNPRC	Kendall et al., 2024	SNPRC			
32772	*Papio*	*hybrid*	United States of /	SNPRC	Kendall et al., 2024	SNPRC			
32773	*Papio*	*hybrid*	United States of /	SNPRC	Kendall et al., 2024	SNPRC			

Sample	Genus	Species	Country	Locality	Source	Dataset	Note		
32785	*Papio*	*hybrid*	United States of /	SNPRC	Kendall et al., 2024	SNPRC			
32794	*Papio*	*hybrid*	United States of /	SNPRC	Kendall et al., 2024	SNPRC			
32797	*Papio*	*hybrid*	United States of /	SNPRC	Kendall et al., 2024	SNPRC			
32801	*Papio*	*hybrid*	United States of /	SNPRC	Kendall et al., 2024	SNPRC			
32849	*Papio*	*hybrid*	United States of /	SNPRC	Kendall et al., 2024	SNPRC			
32860	*Papio*	*hybrid*	United States of /	SNPRC	Kendall et al., 2024	SNPRC			
32863	*Papio*	*hybrid*	United States of /	SNPRC	Kendall et al., 2024	SNPRC			
32876	*Papio*	*hybrid*	United States of /	SNPRC	Kendall et al., 2024	SNPRC			
32906	*Papio*	*hybrid*	United States of /	SNPRC	Kendall et al., 2024	SNPRC			
32909	*Papio*	*hybrid*	United States of /	SNPRC	Kendall et al., 2024	SNPRC			
32988	*Papio*	*hybrid*	United States of /	SNPRC	Kendall et al., 2024	SNPRC			
32993	*Papio*	*hybrid*	United States of /	SNPRC	Kendall et al., 2024	SNPRC			
32995	*Papio*	*hybrid*	United States of /	SNPRC	Kendall et al., 2024	SNPRC			
32996	*Papio*	*hybrid*	United States of /	SNPRC	Kendall et al., 2024	SNPRC			
33082	*Papio*	*hybrid*	United States of /	SNPRC	Kendall et al., 2024	SNPRC			
33083	*Papio*	*hybrid*	United States of /	SNPRC	Kendall et al., 2024	SNPRC			
33097	*Papio*	*hybrid*	United States of /	SNPRC	Kendall et al., 2024	SNPRC			
33106	*Papio*	*hybrid*	United States of /	SNPRC	Kendall et al., 2024	SNPRC			
33112	*Papio*	*hybrid*	United States of /	SNPRC	Kendall et al., 2024	SNPRC			
33115	*Papio*	*hybrid*	United States of /	SNPRC	Kendall et al., 2024	SNPRC			
33163	*Papio*	*hybrid*	United States of /	SNPRC	Kendall et al., 2024	SNPRC			
33524	*Papio*	*hybrid*	United States of /	SNPRC	Kendall et al., 2024	SNPRC			
33545	*Papio*	*hybrid*	United States of /	SNPRC	Kendall et al., 2024	SNPRC			
33599	*Papio*	*hybrid*	United States of /	SNPRC	Kendall et al., 2024	SNPRC			
33605	*Papio*	*hybrid*	United States of /	SNPRC	Kendall et al., 2024	SNPRC			
33631	*Papio*	*hybrid*	United States of /	SNPRC	Kendall et al., 2024	SNPRC			
33863	*Papio*	*hybrid*	United States of /	SNPRC	Kendall et al., 2024	SNPRC			
33874	*Papio*	*hybrid*	United States of /	SNPRC	Kendall et al., 2024	SNPRC			
33889	*Papio*	*hybrid*	United States of /	SNPRC	Kendall et al., 2024	SNPRC			
33908	*Papio*	*hybrid*	United States of /	SNPRC	Kendall et al., 2024	SNPRC			
34568	*Papio*	*hybrid*	United States of /	SNPRC	Kendall et al., 2024	SNPRC			
34857	*Papio*	*hybrid*	United States of /	SNPRC	Kendall et al., 2024	SNPRC			
34859	*Papio*	*hybrid*	United States of /	SNPRC	Kendall et al., 2024	SNPRC			
34897	*Papio*	*hybrid*	United States of /	SNPRC	Kendall et al., 2024	SNPRC			
6265	*Papio*	*hybrid*	United States of /	SNPRC	Kendall et al., 2024	SNPRC			
6382	*Papio*	*hybrid*	United States of /	SNPRC	Kendall et al., 2024	SNPRC			
6716	*Papio*	*hybrid*	United States of /	SNPRC	Kendall et al., 2024	SNPRC			
6955	*Papio*	*hybrid*	United States of /	SNPRC	Kendall et al., 2024	SNPRC			
7091	*Papio*	*hybrid*	United States of /	SNPRC	Kendall et al., 2024	SNPRC			
7158	*Papio*	*hybrid*	United States of /	SNPRC	Kendall et al., 2024	SNPRC			
7267	*Papio*	*hybrid*	United States of /	SNPRC	Kendall et al., 2024	SNPRC			
7311	*Papio*	*hybrid*	United States of /	SNPRC	Kendall et al., 2024	SNPRC			
7478	*Papio*	*hybrid*	United States of /	SNPRC	Kendall et al., 2024	SNPRC			
7625	*Papio*	*hybrid*	United States of /	SNPRC	Kendall et al., 2024	SNPRC			
7777	*Papio*	*hybrid*	United States of /	SNPRC	Kendall et al., 2024	SNPRC			
7790	*Papio*	*hybrid*	United States of /	SNPRC	Kendall et al., 2024	SNPRC			
7937	*Papio*	*hybrid*	United States of /	SNPRC	Kendall et al., 2024	SNPRC			
8134	*Papio*	*hybrid*	United States of /	SNPRC	Kendall et al., 2024	SNPRC			
8170	*Papio*	*hybrid*	United States of /	SNPRC	Kendall et al., 2024	SNPRC			
8307	*Papio*	*hybrid*	United States of /	SNPRC	Kendall et al., 2024	SNPRC			
8344	*Papio*	*hybrid*	United States of /	SNPRC	Kendall et al., 2024	SNPRC			
8395	*Papio*	*hybrid*	United States of /	SNPRC	Kendall et al., 2024	SNPRC			
8465	*Papio*	*hybrid*	United States of /	SNPRC	Kendall et al., 2024	SNPRC			
8581	*Papio*	*hybrid*	United States of /	SNPRC	Kendall et al., 2024	SNPRC			
8596	*Papio*	*hybrid*	United States of /	SNPRC	Kendall et al., 2024	SNPRC			
8635	*Papio*	*hybrid*	United States of /	SNPRC	Kendall et al., 2024	SNPRC			
8653	*Papio*	*hybrid*	United States of /	SNPRC	Kendall et al., 2024	SNPRC			
8780	*Papio*	*hybrid*	United States of /	SNPRC	Kendall et al., 2024	SNPRC			
8995	*Papio*	*hybrid*	United States of /	SNPRC	Kendall et al., 2024	SNPRC			
9045	*Papio*	*hybrid*	United States of /	SNPRC	Kendall et al., 2024	SNPRC			
9086	*Papio*	*hybrid*	United States of /	SNPRC	Kendall et al., 2024	SNPRC			
9128	*Papio*	*hybrid*	United States of /	SNPRC	Kendall et al., 2024	SNPRC			
9481	*Papio*	*hybrid*	United States of /	SNPRC	Kendall et al., 2024	SNPRC			
9514	*Papio*	*hybrid*	United States of /	SNPRC	Kendall et al., 2024	SNPRC			
9562	*Papio*	*hybrid*	United States of /	SNPRC	Kendall et al., 2024	SNPRC			
9656	*Papio*	*hybrid*	United States of /	SNPRC	Kendall et al., 2024	SNPRC			
9841	*Papio*	*hybrid*	United States of /	SNPRC	Kendall et al., 2024	SNPRC			
9860	*Papio*	*hybrid*	United States of /	SNPRC	Kendall et al., 2024	SNPRC			
9878	*Papio*	*hybrid*	United States of /	SNPRC	Kendall et al., 2024	SNPRC			

Sample ID/ Acces	Genus	Species	Country	Locality	Mitochondrial Cla	Source	notes	Mitogenome newly assembled
MT279068	*Papio*	*anubis*	Central African Rep	Bamingi R [Baming	K	Roos et al., 2021		NO
JX946198	*Papio*	*anubis*	Nigeria	Gashaka-Gumti Na	F	Zinner et al., 2013		NO
MT279066	*Papio*	*anubis*	Niger	Mt Todera [Mt Todr	D	Roos et al., 2021		NO
MT279065	*Papio*	*anubis*	Niger	Taranré Rn[Tararat	D	Roos et al., 2021		NO
JX946197	*Papio*	*anubis*	Cote d'Ivoire	Komoé National Pa	D	Zinner et al., 2013		NO
MG787545	*Papio*	*anubis*	Tanzania	Gombe National Pa	G	Roos et al., 2018		NO
MT279062	*Papio*	*anubis*	Democratic Repub	Biruwe, E of Oso R	G	Roos et al., 2021		NO
MT279061	*Papio*	*anubis*	Democratic Repub	Near Labutu [?Lub	G	Roos et al., 2021		NO
KC757406	*Papio*	*anubis*	NA	NA	G	Finstermeier et al., 2013		NO
JX946196	*Papio*	*anubis*	Ethiopia	Managasha Nation	G	Zinner et al., 2013		NO
MT279067	*Papio*	*anubis*	Chad	Enneri Debassar, I	K	Roos et al., 2021		NO
PD_0203	*Papio*	*anubis*	Tanzania	Serengeti National	G	Sørensen et al., 2023		NO
PD_0204	*Papio*	*anubis*	Tanzania	Arusha	G	Sørensen et al., 2023		NO
PD_0209	*Papio*	*anubis*	Tanzania	Ngorongoro	G	Sørensen et al., 2023		NO
PD_0211	*Papio*	*anubis*	Tanzania	Ngorongoro	G	Sørensen et al., 2023		NO
PD_0239	*Papio*	*anubis*	Tanzania	Lake Manyara	G	Sørensen et al., 2023		NO
PD_0240	*Papio*	*anubis*	Tanzania	Lake Manyara	G	Sørensen et al., 2023		NO
PD_0242	*Papio*	*anubis*	Tanzania	Lake Manyara	G	Sørensen et al., 2023		NO
PD_0265	*Papio*	*anubis*	Tanzania	Tarangire	G	Sørensen et al., 2023		NO
PD_0493	*Papio*	*anubis*	Tanzania	Serengeti National	G	Sørensen et al., 2023		NO
PD_0495	*Papio*	*anubis*	Tanzania	Serengeti National	G	Sørensen et al., 2023		NO
PD_0501	*Papio*	*anubis*	Tanzania	Serengeti National	G	Sørensen et al., 2023		NO
PD_0504	*Papio*	*anubis*	Tanzania	Gombe National Pa	G	Sørensen et al., 2023		NO
PD_0641	*Papio*	*anubis*	Tanzania	Tarangire	G	Sørensen et al., 2023		NO
PD_0674	*Papio*	*anubis*	Tanzania	Lake Manyara	G	Sørensen et al., 2023		NO
PD_0682	*Papio*	*anubis*	Tanzania	Lake Manyara	G	Sørensen et al., 2023		NO
PD_0684	*Papio*	*anubis*	Tanzania	Lake Manyara	G	Sørensen et al., 2023		NO
PD_0685	*Papio*	*anubis*	Tanzania	Lake Manyara	G	Sørensen et al., 20	Same MTDNA as P	NO
PD_0723	*Papio*	*anubis*	Ethiopia	Gog	G	Sørensen et al., 2023		NO
PD_0726	*Papio*	*anubis*	Ethiopia	Gog	G	Sørensen et al., 20	Same MTDNA as P	NO
PD_0734	*Papio*	*anubis*	Ethiopia	Gog	G	Sørensen et al., 2023		NO
PD_0743	*Papio*	*anubis*	Ethiopia	Gog	G	Sørensen et al., 2023		NO
MT279060	*Papio*	*cynocephalus*	Tanzania	Udzungwa Mounta	G	Roos et al., 2021		NO
JX946199	*Papio*	*cynocephalus*	Tanzania	Mikumi National Pa	G	Zinner et al., 2013		NO
MT279069	*Papio*	*cynocephalus*	Tanzania	Mahale Mountains	H	Roos et al., 2021		NO
JX946200	*Papio*	*cynocephalus*	Tanzania	Amani (south of Tu	B	Zinner et al., 2013		NO
PD_0215	*Papio*	*cynocephalus*	Tanzania	Ruaha	G	Sørensen et al., 2023		NO
PD_0217	*Papio*	*cynocephalus*	Tanzania	Ruaha	G	Sørensen et al., 2023		NO
PD_0224	*Papio*	*cynocephalus*	Tanzania	Udzungwa Mounta	G	Sørensen et al., 2023		NO
PD_0228	*Papio*	*cynocephalus*	Tanzania	Mahale Mountains	H	Sørensen et al., 2023		NO
PD_0235	*Papio*	cynocephalus	Tanzania	Selous	B	Sørensen et al., 2023		NO
PD_0236	*Papio*	cynocephalus	Tanzania	Selous	B	Sørensen et al., 2023		NO
PD_0237	*Papio*	cynocephalus	Tanzania	Selous	B	Sørensen et al., 2023		NO
PD_0268	*Papio*	cynocephalus	Tanzania	Mikumi National Pa	G	Sørensen et al., 2023		NO
PD_0270	*Papio*	cynocephalus	Tanzania	Mikumi National Pa	G	Sørensen et al., 20	Same MTDNA as P	NO
PD_0662	*Papio*	cynocephalus	Tanzania	Issa-Valley	H	Sørensen et al., 2023		NO
PD_0668	*Papio*	cynocephalus	Tanzania	Mikumi National Pa	G	Sørensen et al., 2023		NO
PD_0746	*Papio*	cynocephalus	Tanzania	Mikumi National Pa	G	Sørensen et al., 20	PDP	NO
PD_0757	*Papio*	cynocephalus	Tanzania	Mikumi National Pa	G	Sørensen et al., 2023		NO
PD_0767	*Papio*	cynocephalus	Tanzania	Mikumi National Pa	G	Sørensen et al., 2023		NO
PD_0768	*Papio*	cynocephalus	Tanzania	Mikumi National Pa	G	Sørensen et al., 2023		NO
HAP	*Papio*	*cynocephalus*	Kenya	Amboseli National	G	Wall et al., 2016	PDP	YES
JX946201	*Papio*	*hamadryas*	Eritrea	Furrus	G	Zinner et al., 2013		NO
MT279063	*Papio*	*hamadryas*	Somalia	[Upper Sheikh]	G	Roos et al., 2021		NO
PD_0696	*Papio*	*hamadryas*	Ethiopia	Filoha	G	Sørensen et al., 2023		NO
PD_0700	*Papio*	*hamadryas*	Ethiopia	Filoha	G	Sørensen et al., 2023		NO
PD_0705	*Papio*	*hamadryas*	Ethiopia	Filoha	G	Sørensen et al., 2023		NO
97074	*Papio*	*hamadryas*	Ethiopia	Awash	G	Rogers et al., 2019		NO
97124	*Papio*	*hamadryas*	Ethiopia	Awash	G	Rogers et al., 2019		NO
PD_0697	*Papio*	*hamadryas*	Ethiopia	Filoha	G	Sørensen et al., 2023		NO
PD_0710	*Papio*	*hamadryas*	Ethiopia	Filoha	G	Sørensen et al., 2023		NO
PD_0699	*Papio*	*hamadryas*	Ethiopia	Filoha	G	Sørensen et al., 2023		NO
PD_0717	*Papio*	*hamadryas*	Ethiopia	Filoha	G	Sørensen et al., 2023		NO
PD_0702	*Papio*	*hamadryas*	Ethiopia	Filoha	G	Sørensen et al., 2023		NO
PD_0703	*Papio*	*hamadryas*	Ethiopia	Filoha	G	Sørensen et al., 2023		NO
PD_0709	*Papio*	*hamadryas*	Ethiopia	Filoha	G	Sørensen et al., 2023		NO
PD_0713	*Papio*	*hamadryas*	Ethiopia	Filoha	G	Sørensen et al., 2023		NO
PD_0719	*Papio*	*hamadryas*	Ethiopia	Filoha	G	Sørensen et al., 2023		NO
PD_0701	*Papio*	*hamadryas*	Ethiopia	Filoha	G	Sørensen et al., 2023		NO
PD_0698	*Papio*	*hamadryas*	Ethiopia	Filoha	G	Sørensen et al., 2023		NO
PD_0718	*Papio*	*hamadryas*	Ethiopia	Filoha	G	Sørensen et al., 2023		NO
PD_0716	*Papio*	*hamadryas*	Ethiopia	Filoha	G	Sørensen et al., 2023		NO
PD_0704	*Papio*	*hamadryas*	Ethiopia	Filoha	G	Sørensen et al., 2023		NO
PD_0706	*Papio*	*hamadryas*	Ethiopia	Filoha	G	Sørensen et al., 2023		NO
PD_0712	*Papio*	*hamadryas*	Ethiopia	Filoha	G	Sørensen et al., 2023		NO
PD_0707	*Papio*	*hamadryas*	Ethiopia	Filoha	G	Sørensen et al., 2023		NO
PD_0708	*Papio*	*hamadryas*	Ethiopia	Filoha	G	Sørensen et al., 2023		NO
PD_0711	*Papio*	*hamadryas*	Ethiopia	Filoha	G	Sørensen et al., 2023		NO
PD_0714	*Papio*	*hamadryas*	Ethiopia	Filoha	G	Sørensen et al., 2023		NO
PD_0715	*Papio*	*hamadryas*	Ethiopia	Filoha	G	Sørensen et al., 2023		NO
MZ907660	*Papio*	*hamadryas*	Ethiopia	Filoha	G	Chiou et al., 2022	SAMN20949826	NO
MZ907661	*Papio*	*hamadryas*	Ethiopia	Filoha	G	Chiou et al., 2022	SAMN20949827 (F	NO
MZ907662	*Papio*	*hamadryas*	Ethiopia	Filoha	G	Chiou et al., 2022	SAMN20949828	NO
MZ907663	*Papio*	*hamadryas*	Ethiopia	Filoha	G	Chiou et al., 2022	SAMN20949829	NO

Sample ID/ Acces	Genus	Species	Country	Locality	Mitochondrial Cla	Source	notes	Mitogenome newly assembled
MZ907664	*Papio*	*hamadryas*	Ethiopia	Filoha	G	Chiou et al., 2022	SAMN20949830	NO
MZ907665	*Papio*	*hamadryas*	Ethiopia	Filoha	G	Chiou et al., 2022	SAMN20949831	NO
MZ907666	*Papio*	*hamadryas*	Ethiopia	Filoha	G	Chiou et al., 2022	SAMN20949832	NO
MZ907667	*Papio*	*hamadryas*	Ethiopia	Filoha	G	Chiou et al., 2022	SAMN20949833	NO
MZ907668	*Papio*	*hamadryas*	Ethiopia	Filoha	G	Chiou et al., 2022	SAMN20949834	NO
MZ907669	*Papio*	*hamadryas*	Ethiopia	Filoha	G	Chiou et al., 2022	SAMN20949835	NO
MZ907670	*Papio*	*hamadryas*	Ethiopia	Filoha	G	Chiou et al., 2022	SAMN20949836	NO
MZ907671	*Papio*	*hamadryas*	Ethiopia	Filoha	G	Chiou et al., 2022	SAMN20949837 (F	NO
MZ907672	*Papio*	*hamadryas*	Ethiopia	Filoha	G	Chiou et al., 2022	SAMN20949838	NO
MZ907673	*Papio*	*hamadryas*	Ethiopia	Filoha	G	Chiou et al., 2022	SAMN20949839	NO
MZ907674	*Papio*	*hamadryas*	Ethiopia	Filoha	G	Chiou et al., 2022	SAMN20949840	NO
MZ907675	*Papio*	*hamadryas*	Ethiopia	Filoha	G	Chiou et al., 2022	SAMN20949841	NO
MZ907676	*Papio*	*hamadryas*	Ethiopia	Filoha	G	Chiou et al., 2022	SAMN20949842	NO
MZ907677	*Papio*	*hamadryas*	Ethiopia	Filoha	G	Chiou et al., 2022	SAMN20949843	NO
JX946202	*Papio*	*kindae*	Zambia	Kasanka National I	C	Zinner et al., 2013		NO
PD_0751	*Papio*	*kindae*	Zambia	Chunga	C	Sørensen et al., 20	PDP	NO
PD_0749	*Papio*	*kindae*	Zambia	Chunga	C	Sørensen et al., 20	PDP	NO
JX946203	*Papio*	*papio*	Senegal	Niokolo Koba Natic	F	Zinner et al 2013		NO
MT279064	*Papio*	*papio*	Sierra Leone	Makontande, N of I	F	Roos et al., 2021		NO
PD_0390	*Papio*	*papio*	Senegal	Niokolo Koba Natic	F	Sørensen et al., 2023		NO
PD_0393	*Papio*	*papio*	Senegal	Niokolo Koba Natic	F	Sørensen et al., 2023		NO
PD_0394	*Papio*	*papio*	Senegal	Niokolo Koba Natic	F	Sørensen et al., 20	Same MTDNA as F	NO
JX946204	*Papio*	*ursinus*	South Africa	DeHoop NR	A	Zinner et al., 2013		NO
JX946205	*Papio*	*ursinus*	South Africa	Blyde River	B	Zinner et al., 2013		NO
PD_0692	*Papio*	*ursinus*	Zambia	Dendro Park	B	Sørensen et al., 20	Same MTDNA as F	NO
NC_019802	*Theropithecus*	*gelada*	-	-	-	Hodgson et al., 2009		NO
1X4811	*Papio*	*hybrid*	United States of Ar	SNPRC		Kendall et al., 2024	SNPRC	YES
1X4777	*Papio*	*hybrid*	United States of Ar	SNPRC		Kendall et al., 2024	SNPRC	YES
1X4519	*Papio*	*hybrid*	United States of Ar	SNPRC		Kendall et al., 2024	SNPRC	YES
1X4384	*Papio*	*anubis*	United States of Ar	SNPRC		Kendall et al., 2024	SNPRC FOUNDEF	YES
1X4209	*Papio*	*hybrid*	United States of Ar	SNPRC		Kendall et al., 2024	SNPRC	YES
1X4179	*Papio*	*hybrid*	United States of Ar	SNPRC		Kendall et al., 2024	SNPRC	YES
1X4080	*Papio*	*hybrid*	United States of Ar	SNPRC		Kendall et al., 2024	SNPRC	YES
1X3837	*Papio*	*hybrid*	United States of Ar	SNPRC		Kendall et al., 2024	SNPRC	YES
1X3822	*Papio*	*hybrid*	United States of Ar	SNPRC		Kendall et al., 2024	SNPRC	YES
1X3796	*Papio*	*hybrid*	United States of Ar	SNPRC		Kendall et al., 2024	SNPRC	YES
1X3697	*Papio*	*hybrid*	United States of Ar	SNPRC		Kendall et al., 2024	SNPRC	YES
1X3656	*Papio*	*hybrid*	United States of Ar	SNPRC		Kendall et al., 2024	SNPRC	YES
1X3576	*Papio*	*cynocephalus*	United States of Ar	SNPRC		Kendall et al., 2024	SNPRC FOUNDEF	YES
1X3321	*Papio*	*hybrid*	United States of Ar	SNPRC		Kendall et al., 2024	SNPRC	YES but deleted due to high missing/ambigous bases
1X3162	*Papio*	*anubis*	United States of Ar	SNPRC		Kendall et al., 2024	SNPRC FOUNDEF	YES
1X2892	*Papio*	*hybrid*	United States of Ar	SNPRC		Kendall et al., 2024	SNPRC	YES but deleted due to high missing/ambigous bases
1X2891	*Papio*	*anubis*	United States of Ar	SNPRC		Kendall et al., 2024	SNPRC FOUNDEF	YES
1X2816	*Papio*	*hybrid*	United States of Ar	SNPRC		Kendall et al., 2024	SNPRC	YES
1X2664	*Papio*	*hybrid*	United States of Ar	SNPRC		Kendall et al., 2024	SNPRC	YES
1X2304	*Papio*	*cynocephalus*	United States of Ar	SNPRC		Kendall et al., 2024	SNPRC FOUNDEF	YES
1X2231	*Papio*	*hybrid*	United States of Ar	SNPRC		Kendall et al., 2024	SNPRC	YES
1X2208	*Papio*	*cynocephalus*	United States of Ar	SNPRC		Kendall et al., 2024	SNPRC FOUNDEF	YES
1X2124	*Papio*	*hybrid*	United States of Ar	SNPRC		Kendall et al., 2024	SNPRC	YES
1X2055	*Papio*	*hybrid*	United States of Ar	SNPRC		Kendall et al., 2024	SNPRC	YES
1X2054	*Papio*	*hybrid*	United States of Ar	SNPRC		Kendall et al., 2024	SNPRC	YES
1X2049	*Papio*	*cynocephalus*	United States of Ar	SNPRC		Kendall et al., 2024	SNPRC FOUNDEF	YES
1X1960	*Papio*	*hybrid*	United States of Ar	SNPRC		Kendall et al., 2024	SNPRC	YES
1X1958	*Papio*	*hybrid*	United States of Ar	SNPRC		Kendall et al., 2024	SNPRC	YES
1X1947	*Papio*	*anubis*	United States of Ar	SNPRC		Kendall et al., 2024	SNPRC FOUNDEF	YES
1X1939	*Papio*	*anubis*	United States of Ar	SNPRC		Kendall et al., 2024	SNPRC FOUNDEF	YES
1X1765	*Papio*	*anubis*	United States of Ar	SNPRC		Kendall et al., 2024	SNPRC FOUNDEF	YES
1X1734	*Papio*	*cynocephalus*	United States of Ar	SNPRC		Kendall et al., 2024	SNPRC FOUNDEF	YES
1X1700	*Papio*	*anubis*	United States of Ar	SNPRC		Kendall et al., 2024	SNPRC FOUNDEF	YES
1X1693	*Papio*	*hybrid*	United States of Ar	SNPRC		Kendall et al., 2024	SNPRC	YES but deleted due to high missing/ambigous bases
1X1672	*Papio*	*anubis*	United States of Ar	SNPRC		Kendall et al., 2024	SNPRC FOUNDEF	YES
1X1487	*Papio*	*hybrid*	United States of Ar	SNPRC		Kendall et al., 2024	SNPRC	YES
1X1392	*Papio*	*hybrid*	United States of Ar	SNPRC		Kendall et al., 2024	SNPRC	YES
1X1237	*Papio*	*hybrid*	United States of Ar	SNPRC		Kendall et al., 2024	SNPRC	YES
1X1230	*Papio*	*hybrid*	United States of Ar	SNPRC		Kendall et al., 2024	SNPRC	YES
1X1181	*Papio*	*anubis*	United States of Ar	SNPRC		Kendall et al., 2024	SNPRC FOUNDEF	YES
1X1152	*Papio*	*anubis*	United States of Ar	SNPRC		Kendall et al., 2024	SNPRC FOUNDEF	YES
1X1146	*Papio*	*anubis*	United States of Ar	SNPRC		Kendall et al., 2024	SNPRC FOUNDEF	YES
1X1126	*Papio*	*anubis*	United States of Ar	SNPRC		Kendall et al., 2024	SNPRC FOUNDEF	YES
1X1125	*Papio*	*anubis*	United States of Ar	SNPRC		Kendall et al., 2024	SNPRC FOUNDEF	YES
1X1032	*Papio*	*anubis*	United States of Ar	SNPRC		Kendall et al., 2024	SNPRC FOUNDEF	YES
1X0951	*Papio*	*anubis*	United States of Ar	SNPRC		Kendall et al., 2024	SNPRC FOUNDEF	YES
1X0843	*Papio*	*hybrid*	United States of Ar	SNPRC		Kendall et al., 2024	SNPRC	YES
1X0832	*Papio*	*anubis*	United States of Ar	SNPRC		Kendall et al., 2024	SNPRC FOUNDEF	YES
1X0830	*Papio*	*hybrid*	United States of Ar	SNPRC		Kendall et al., 2024	SNPRC	YES but deleted due to high missing/ambigous bases
1X0812	*Papio*	*anubis*	United States of Ar	SNPRC		Kendall et al., 2024	SNPRC FOUNDEF	YES
1X0808	*Papio*	*anubis*	United States of Ar	SNPRC		Kendall et al., 2024	SNPRC FOUNDEF	YES
1X0580	*Papio*	*anubis*	United States of Ar	SNPRC		Kendall et al., 2024	SNPRC FOUNDEF	YES
1X0576	*Papio*	*anubis*	United States of Ar	SNPRC		Kendall et al., 2024	SNPRC FOUNDEF	YES
1X0356	*Papio*	*hybrid*	United States of Ar	SNPRC		Kendall et al., 2024	SNPRC	YES
1X0354	*Papio*	*hybrid*	United States of Ar	SNPRC		Kendall et al., 2024	SNPRC	YES
1X0351	*Papio*	*hybrid*	United States of Ar	SNPRC		Kendall et al., 2024	SNPRC	YES but deleted due to high missing/ambigous bases
1X0153	*Papio*	*anubis*	United States of Ar	SNPRC		Kendall et al., 2024	SNPRC FOUNDEF	YES
1X0110	*Papio*	*cynocephalus*	United States of Ar	SNPRC		Kendall et al., 2024	SNPRC FOUNDEF	YES

Sample ID/ Acces	Genus	Species	Country	Locality	Mitochondrial Cla	Source	notes	Mitogenome newly assembled
1X0102	*Papio*	*cynocephalus*	United States of Ar	SNPRC		Kendall et al., 2024	SNPRC FOUNDEF	YES
1X0035	*Papio*	*anubis*	United States of Ar	SNPRC		Kendall et al., 2024	SNPRC FOUNDEF	YES
1X0026	*Papio*	*anubis*	United States of Ar	SNPRC		Kendall et al., 2024	SNPRC FOUNDEF	YES
1X0014	*Papio*	*anubis*	United States of Ar	SNPRC		Kendall et al., 2024	SNPRC FOUNDEF	YES
1X3548	*Papio*	*cynocephalus*	United States of Ar	SNPRC		Kendall et al., 2024	SNPRC FOUNDEF	YES
1X1979	*Papio*	*anubis*	United States of Ar	SNPRC		Kendall et al., 2024	SNPRC FOUNDEF	YES
1X0291	*Papio*	*hybrid*	United States of Ar	SNPRC		Kendall et al., 2024	SNPRC	YES
34897	*Papio*	*hybrid*	United States of Ar	SNPRC		Kendall et al., 2024	SNPRC	YES
34859	*Papio*	*hybrid*	United States of Ar	SNPRC		Kendall et al., 2024	SNPRC	YES
34857	*Papio*	*hybrid*	United States of Ar	SNPRC		Kendall et al., 2024	SNPRC	YES
34568	*Papio*	*hybrid*	United States of Ar	SNPRC		Kendall et al., 2024	SNPRC	YES
33908	*Papio*	*hybrid*	United States of Ar	SNPRC		Kendall et al., 2024	SNPRC	YES
33889	*Papio*	*hybrid*	United States of Ar	SNPRC		Kendall et al., 2024	SNPRC	YES
33874	*Papio*	*hybrid*	United States of Ar	SNPRC		Kendall et al., 2024	SNPRC	YES
33863	*Papio*	*hybrid*	United States of Ar	SNPRC		Kendall et al., 2024	SNPRC	YES
33631	*Papio*	*hybrid*	United States of Ar	SNPRC		Kendall et al., 2024	SNPRC	YES
33605	*Papio*	*hybrid*	United States of Ar	SNPRC		Kendall et al., 2024	SNPRC	YES
33599	*Papio*	*hybrid*	United States of Ar	SNPRC		Kendall et al., 2024	SNPRC	YES
33545	*Papio*	*hybrid*	United States of Ar	SNPRC		Kendall et al., 2024	SNPRC	YES
33524	*Papio*	*hybrid*	United States of Ar	SNPRC		Kendall et al., 2024	SNPRC	YES
33163	*Papio*	*hybrid*	United States of Ar	SNPRC		Kendall et al., 2024	SNPRC	YES
33115	*Papio*	*hybrid*	United States of Ar	SNPRC		Kendall et al., 2024	SNPRC	YES
33112	*Papio*	*hybrid*	United States of Ar	SNPRC		Kendall et al., 2024	SNPRC	YES
33106	*Papio*	*hybrid*	United States of Ar	SNPRC		Kendall et al., 2024	SNPRC	YES
33097	*Papio*	*hybrid*	United States of Ar	SNPRC		Kendall et al., 2024	SNPRC	YES
33083	*Papio*	*hybrid*	United States of Ar	SNPRC		Kendall et al., 2024	SNPRC	YES
33082	*Papio*	*hybrid*	United States of Ar	SNPRC		Kendall et al., 2024	SNPRC	YES
32996	*Papio*	*hybrid*	United States of Ar	SNPRC		Kendall et al., 2024	SNPRC	YES
32995	*Papio*	*hybrid*	United States of Ar	SNPRC		Kendall et al., 2024	SNPRC	YES
32993	*Papio*	*hybrid*	United States of Ar	SNPRC		Kendall et al., 2024	SNPRC	YES
32988	*Papio*	*hybrid*	United States of Ar	SNPRC		Kendall et al., 2024	SNPRC	YES
32909	*Papio*	*hybrid*	United States of Ar	SNPRC		Kendall et al., 2024	SNPRC	YES
32906	*Papio*	*hybrid*	United States of Ar	SNPRC		Kendall et al., 2024	SNPRC	YES
32876	*Papio*	*hybrid*	United States of Ar	SNPRC		Kendall et al., 2024	SNPRC	YES
32863	*Papio*	*hybrid*	United States of Ar	SNPRC		Kendall et al., 2024	SNPRC	YES
32860	*Papio*	*hybrid*	United States of Ar	SNPRC		Kendall et al., 2024	SNPRC	YES
32849	*Papio*	*hybrid*	United States of Ar	SNPRC		Kendall et al., 2024	SNPRC	YES
32801	*Papio*	*hybrid*	United States of Ar	SNPRC		Kendall et al., 2024	SNPRC	YES
32797	*Papio*	*hybrid*	United States of Ar	SNPRC		Kendall et al., 2024	SNPRC	YES
32794	*Papio*	*hybrid*	United States of Ar	SNPRC		Kendall et al., 2024	SNPRC	YES
32785	*Papio*	*hybrid*	United States of Ar	SNPRC		Kendall et al., 2024	SNPRC	YES
32773	*Papio*	*hybrid*	United States of Ar	SNPRC		Kendall et al., 2024	SNPRC	YES
32772	*Papio*	*hybrid*	United States of Ar	SNPRC		Kendall et al., 2024	SNPRC	YES
32736	*Papio*	*hybrid*	United States of Ar	SNPRC		Kendall et al., 2024	SNPRC	YES
32651	*Papio*	*hybrid*	United States of Ar	SNPRC		Kendall et al., 2024	SNPRC	YES
32629	*Papio*	*hybrid*	United States of Ar	SNPRC		Kendall et al., 2024	SNPRC	YES
32606	*Papio*	*hybrid*	United States of Ar	SNPRC		Kendall et al., 2024	SNPRC	YES
32591	*Papio*	*hybrid*	United States of Ar	SNPRC		Kendall et al., 2024	SNPRC	YES
32588	*Papio*	*hybrid*	United States of Ar	SNPRC		Kendall et al., 2024	SNPRC	YES
32560	*Papio*	*hybrid*	United States of Ar	SNPRC		Kendall et al., 2024	SNPRC	YES
32557	*Papio*	*hybrid*	United States of Ar	SNPRC		Kendall et al., 2024	SNPRC	YES
32553	*Papio*	*hybrid*	United States of Ar	SNPRC		Kendall et al., 2024	SNPRC	YES
32552	*Papio*	*hybrid*	United States of Ar	SNPRC		Kendall et al., 2024	SNPRC	YES
32540	*Papio*	*hybrid*	United States of Ar	SNPRC		Kendall et al., 2024	SNPRC	YES
32539	*Papio*	*hybrid*	United States of Ar	SNPRC		Kendall et al., 2024	SNPRC	YES
32472	*Papio*	*hybrid*	United States of Ar	SNPRC		Kendall et al., 2024	SNPRC	YES
32469	*Papio*	*hybrid*	United States of Ar	SNPRC		Kendall et al., 2024	SNPRC	YES
32467	*Papio*	*hybrid*	United States of Ar	SNPRC		Kendall et al., 2024	SNPRC	YES
32451	*Papio*	*hybrid*	United States of Ar	SNPRC		Kendall et al., 2024	SNPRC	YES
32372	*Papio*	*hybrid*	United States of Ar	SNPRC		Kendall et al., 2024	SNPRC	YES
32363	*Papio*	*hybrid*	United States of Ar	SNPRC		Kendall et al., 2024	SNPRC	YES
32358	*Papio*	*hybrid*	United States of Ar	SNPRC		Kendall et al., 2024	SNPRC	YES
32356	*Papio*	*hybrid*	United States of Ar	SNPRC		Kendall et al., 2024	SNPRC	YES
32311	*Papio*	*hybrid*	United States of Ar	SNPRC		Kendall et al., 2024	SNPRC	YES
32299	*Papio*	*hybrid*	United States of Ar	SNPRC		Kendall et al., 2024	SNPRC	YES
32298	*Papio*	*hybrid*	United States of Ar	SNPRC		Kendall et al., 2024	SNPRC	YES
32227	*Papio*	*hybrid*	United States of Ar	SNPRC		Kendall et al., 2024	SNPRC	YES
32224	*Papio*	*hybrid*	United States of Ar	SNPRC		Kendall et al., 2024	SNPRC	YES
32215	*Papio*	*hybrid*	United States of Ar	SNPRC		Kendall et al., 2024	SNPRC	YES
32209	*Papio*	*hybrid*	United States of Ar	SNPRC		Kendall et al., 2024	SNPRC	YES
32207	*Papio*	*hybrid*	United States of Ar	SNPRC		Kendall et al., 2024	SNPRC	YES
32187	*Papio*	*hybrid*	United States of Ar	SNPRC		Kendall et al., 2024	SNPRC	YES
32132	*Papio*	*hybrid*	United States of Ar	SNPRC		Kendall et al., 2024	SNPRC	YES
32130	*Papio*	*hybrid*	United States of Ar	SNPRC		Kendall et al., 2024	SNPRC	YES
32103	*Papio*	*hybrid*	United States of Ar	SNPRC		Kendall et al., 2024	SNPRC	YES
32089	*Papio*	*hybrid*	United States of Ar	SNPRC		Kendall et al., 2024	SNPRC	YES
32053	*Papio*	*hybrid*	United States of Ar	SNPRC		Kendall et al., 2024	SNPRC	YES
32043	*Papio*	*hybrid*	United States of Ar	SNPRC		Kendall et al., 2024	SNPRC	YES
32040	*Papio*	*hybrid*	United States of Ar	SNPRC		Kendall et al., 2024	SNPRC	YES
32039	*Papio*	*hybrid*	United States of Ar	SNPRC		Kendall et al., 2024	SNPRC	YES
32025	*Papio*	*hybrid*	United States of Ar	SNPRC		Kendall et al., 2024	SNPRC	YES
31997	*Papio*	*hybrid*	United States of Ar	SNPRC		Kendall et al., 2024	SNPRC	YES
31994	*Papio*	*hybrid*	United States of Ar	SNPRC		Kendall et al., 2024	SNPRC	YES
31993	*Papio*	*hybrid*	United States of Ar	SNPRC		Kendall et al., 2024	SNPRC	YES

Sample ID/ Acces	Genus	Species	Country	Locality	Mitochondrial Cla	Source	notes	Mitogenome newly assembled			
31984	*Papio*	*hybrid*	United States of Ar	SNPRC		Kendall et al., 2024	SNPRC	YES			
31980	*Papio*	*hybrid*	United States of Ar	SNPRC		Kendall et al., 2024	SNPRC	YES			
31970	*Papio*	*hybrid*	United States of Ar	SNPRC		Kendall et al., 2024	SNPRC	YES			
31966	*Papio*	*hybrid*	United States of Ar	SNPRC		Kendall et al., 2024	SNPRC	YES			
31960	*Papio*	*hybrid*	United States of Ar	SNPRC		Kendall et al., 2024	SNPRC	YES			
31958	*Papio*	*hybrid*	United States of Ar	SNPRC		Kendall et al., 2024	SNPRC	YES			
31952	*Papio*	*hybrid*	United States of Ar	SNPRC		Kendall et al., 2024	SNPRC	YES			
31938	*Papio*	*hybrid*	United States of Ar	SNPRC		Kendall et al., 2024	SNPRC	YES			
31937	*Papio*	*hybrid*	United States of Ar	SNPRC		Kendall et al., 2024	SNPRC	YES			
31931	*Papio*	*hybrid*	United States of Ar	SNPRC		Kendall et al., 2024	SNPRC	YES			
31929	*Papio*	*hybrid*	United States of Ar	SNPRC		Kendall et al., 2024	SNPRC	YES			
31903	*Papio*	*hybrid*	United States of Ar	SNPRC		Kendall et al., 2024	SNPRC	YES			
31902	*Papio*	*hybrid*	United States of Ar	SNPRC		Kendall et al., 2024	SNPRC	YES			
31882	*Papio*	*hybrid*	United States of Ar	SNPRC		Kendall et al., 2024	SNPRC	YES			
31880	*Papio*	*hybrid*	United States of Ar	SNPRC		Kendall et al., 2024	SNPRC	YES			
31852	*Papio*	*hybrid*	United States of Ar	SNPRC		Kendall et al., 2024	SNPRC	YES			
31849	*Papio*	*hybrid*	United States of Ar	SNPRC		Kendall et al., 2024	SNPRC	YES			
31846	*Papio*	*hybrid*	United States of Ar	SNPRC		Kendall et al., 2024	SNPRC	YES			
31836	*Papio*	*hybrid*	United States of Ar	SNPRC		Kendall et al., 2024	SNPRC	YES			
31805	*Papio*	*hybrid*	United States of Ar	SNPRC		Kendall et al., 2024	SNPRC	YES			
31804	*Papio*	*hybrid*	United States of Ar	SNPRC		Kendall et al., 2024	SNPRC	YES			
31800	*Papio*	*hybrid*	United States of Ar	SNPRC		Kendall et al., 2024	SNPRC	YES			
31789	*Papio*	*hybrid*	United States of Ar	SNPRC		Kendall et al., 2024	SNPRC	YES			
31786	*Papio*	*hybrid*	United States of Ar	SNPRC		Kendall et al., 2024	SNPRC	YES			
31778	*Papio*	*hybrid*	United States of Ar	SNPRC		Kendall et al., 2024	SNPRC	YES			
31764	*Papio*	*hybrid*	United States of Ar	SNPRC		Kendall et al., 2024	SNPRC	YES			
31738	*Papio*	*hybrid*	United States of Ar	SNPRC		Kendall et al., 2024	SNPRC	YES			
31731	*Papio*	*hybrid*	United States of Ar	SNPRC		Kendall et al., 2024	SNPRC	YES			
31729	*Papio*	*hybrid*	United States of Ar	SNPRC		Kendall et al., 2024	SNPRC	YES			
31720	*Papio*	*hybrid*	United States of Ar	SNPRC		Kendall et al., 2024	SNPRC	YES			
31716	*Papio*	*hybrid*	United States of Ar	SNPRC		Kendall et al., 2024	SNPRC	YES			
31693	*Papio*	*hybrid*	United States of Ar	SNPRC		Kendall et al., 2024	SNPRC	YES			
31666	*Papio*	*hybrid*	United States of Ar	SNPRC		Kendall et al., 2024	SNPRC	YES			
31659	*Papio*	*hybrid*	United States of Ar	SNPRC		Kendall et al., 2024	SNPRC	YES			
31656	*Papio*	*hybrid*	United States of Ar	SNPRC		Kendall et al., 2024	SNPRC	YES			
31650	*Papio*	*hybrid*	United States of Ar	SNPRC		Kendall et al., 2024	SNPRC	YES			
31642	*Papio*	*hybrid*	United States of Ar	SNPRC		Kendall et al., 2024	SNPRC	YES			
31630	*Papio*	*hybrid*	United States of Ar	SNPRC		Kendall et al., 2024	SNPRC	YES			
31624	*Papio*	*hybrid*	United States of Ar	SNPRC		Kendall et al., 2024	SNPRC	YES			
31605	*Papio*	*hybrid*	United States of Ar	SNPRC		Kendall et al., 2024	SNPRC	YES			
31602	*Papio*	*hybrid*	United States of Ar	SNPRC		Kendall et al., 2024	SNPRC	YES			
31578	*Papio*	*hybrid*	United States of Ar	SNPRC		Kendall et al., 2024	SNPRC	YES			
31576	*Papio*	*hybrid*	United States of Ar	SNPRC		Kendall et al., 2024	SNPRC	YES			
31573	*Papio*	*hybrid*	United States of Ar	SNPRC		Kendall et al., 2024	SNPRC	YES			
31560	*Papio*	*hybrid*	United States of Ar	SNPRC		Kendall et al., 2024	SNPRC	YES			
31559	*Papio*	*hybrid*	United States of Ar	SNPRC		Kendall et al., 2024	SNPRC	YES			
31556	*Papio*	*hybrid*	United States of Ar	SNPRC		Kendall et al., 2024	SNPRC	YES			
31553	*Papio*	*hybrid*	United States of Ar	SNPRC		Kendall et al., 2024	SNPRC	YES			
31552	*Papio*	*hybrid*	United States of Ar	SNPRC		Kendall et al., 2024	SNPRC	YES			
31543	*Papio*	*hybrid*	United States of Ar	SNPRC		Kendall et al., 2024	SNPRC	YES			
31542	*Papio*	*hybrid*	United States of Ar	SNPRC		Kendall et al., 2024	SNPRC	YES			
31540	*Papio*	*hybrid*	United States of Ar	SNPRC		Kendall et al., 2024	SNPRC	YES			
31528	*Papio*	*hybrid*	United States of Ar	SNPRC		Kendall et al., 2024	SNPRC	YES			
31526	*Papio*	*hybrid*	United States of Ar	SNPRC		Kendall et al., 2024	SNPRC	YES			
31525	*Papio*	*hybrid*	United States of Ar	SNPRC		Kendall et al., 2024	SNPRC	YES			
31518	*Papio*	*hybrid*	United States of Ar	SNPRC		Kendall et al., 2024	SNPRC	YES			
31515	*Papio*	*hybrid*	United States of Ar	SNPRC		Kendall et al., 2024	SNPRC	YES			
31511	*Papio*	*hybrid*	United States of Ar	SNPRC		Kendall et al., 2024	SNPRC	YES			
31507	*Papio*	*hybrid*	United States of Ar	SNPRC		Kendall et al., 2024	SNPRC	YES			
31506	*Papio*	*hybrid*	United States of Ar	SNPRC		Kendall et al., 2024	SNPRC	YES			
31500	*Papio*	*hybrid*	United States of Ar	SNPRC		Kendall et al., 2024	SNPRC	YES			
31499	*Papio*	*hybrid*	United States of Ar	SNPRC		Kendall et al., 2024	SNPRC	YES			
31498	*Papio*	*hybrid*	United States of Ar	SNPRC		Kendall et al., 2024	SNPRC	YES			
31497	*Papio*	*hybrid*	United States of Ar	SNPRC		Kendall et al., 2024	SNPRC	YES			
31490	*Papio*	*hybrid*	United States of Ar	SNPRC		Kendall et al., 2024	SNPRC	YES			
31484	*Papio*	*hybrid*	United States of Ar	SNPRC		Kendall et al., 2024	SNPRC	YES			
31482	*Papio*	*hybrid*	United States of Ar	SNPRC		Kendall et al., 2024	SNPRC	YES			
31477	*Papio*	*hybrid*	United States of Ar	SNPRC		Kendall et al., 2024	SNPRC	YES			
31475	*Papio*	*hybrid*	United States of Ar	SNPRC		Kendall et al., 2024	SNPRC	YES			
31471	*Papio*	*hybrid*	United States of Ar	SNPRC		Kendall et al., 2024	SNPRC	YES			
31469	*Papio*	*hybrid*	United States of Ar	SNPRC		Kendall et al., 2024	SNPRC	YES			
31462	*Papio*	*hybrid*	United States of Ar	SNPRC		Kendall et al., 2024	SNPRC	YES			
31457	*Papio*	*hybrid*	United States of Ar	SNPRC		Kendall et al., 2024	SNPRC	YES			
31440	*Papio*	*hybrid*	United States of Ar	SNPRC		Kendall et al., 2024	SNPRC	YES			
31417	*Papio*	*hybrid*	United States of Ar	SNPRC		Kendall et al., 2024	SNPRC	YES			
31415	*Papio*	*hybrid*	United States of Ar	SNPRC		Kendall et al., 2024	SNPRC	YES			
31410	*Papio*	*hybrid*	United States of Ar	SNPRC		Kendall et al., 2024	SNPRC	YES			
31409	*Papio*	*hybrid*	United States of Ar	SNPRC		Kendall et al., 2024	SNPRC	YES			
31408	*Papio*	*hybrid*	United States of Ar	SNPRC		Kendall et al., 2024	SNPRC	YES			
31407	*Papio*	*hybrid*	United States of Ar	SNPRC		Kendall et al., 2024	SNPRC	YES			
31401	*Papio*	*hybrid*	United States of Ar	SNPRC		Kendall et al., 2024	SNPRC	YES			
31398	*Papio*	*hybrid*	United States of Ar	SNPRC		Kendall et al., 2024	SNPRC	YES			
31392	*Papio*	*hybrid*	United States of Ar	SNPRC		Kendall et al., 2024	SNPRC	YES			
31364	*Papio*	*hybrid*	United States of Ar	SNPRC		Kendall et al., 2024	SNPRC	YES			

Sample ID/ Acces	Genus	Species	Country	Locality	Mitochondrial Cla	Source	notes	Mitogenome newly assembled
31358	*Papio*	*hybrid*	United States of Ar	SNPRC		Kendall et al., 2024	SNPRC	YES
31337	*Papio*	*hybrid*	United States of Ar	SNPRC		Kendall et al., 2024	SNPRC	YES
31333	*Papio*	*hybrid*	United States of Ar	SNPRC		Kendall et al., 2024	SNPRC	YES
31331	*Papio*	*hybrid*	United States of Ar	SNPRC		Kendall et al., 2024	SNPRC	YES
31329	*Papio*	*hybrid*	United States of Ar	SNPRC		Kendall et al., 2024	SNPRC	YES
31327	*Papio*	*hybrid*	United States of Ar	SNPRC		Kendall et al., 2024	SNPRC	YES
31311	*Papio*	*hybrid*	United States of Ar	SNPRC		Kendall et al., 2024	SNPRC	YES
31300	*Papio*	*hybrid*	United States of Ar	SNPRC		Kendall et al., 2024	SNPRC	YES
31299	*Papio*	*hybrid*	United States of Ar	SNPRC		Kendall et al., 2024	SNPRC	YES
31297	*Papio*	*hybrid*	United States of Ar	SNPRC		Kendall et al., 2024	SNPRC	YES
31288	*Papio*	*hybrid*	United States of Ar	SNPRC		Kendall et al., 2024	SNPRC	YES
31284	*Papio*	*hybrid*	United States of Ar	SNPRC		Kendall et al., 2024	SNPRC	YES
31275	*Papio*	*hybrid*	United States of Ar	SNPRC		Kendall et al., 2024	SNPRC	YES
31255	*Papio*	*hybrid*	United States of Ar	SNPRC		Kendall et al., 2024	SNPRC	YES
31235	*Papio*	*hybrid*	United States of Ar	SNPRC		Kendall et al., 2024	SNPRC	YES
31234	*Papio*	*hybrid*	United States of Ar	SNPRC		Kendall et al., 2024	SNPRC	YES
31178	*Papio*	*hybrid*	United States of Ar	SNPRC		Kendall et al., 2024	SNPRC	YES
31177	*Papio*	*hybrid*	United States of Ar	SNPRC		Kendall et al., 2024	SNPRC	YES
31152	*Papio*	*hybrid*	United States of Ar	SNPRC		Kendall et al., 2024	SNPRC	YES
31145	*Papio*	*hybrid*	United States of Ar	SNPRC		Kendall et al., 2024	SNPRC	YES
31134	*Papio*	*hybrid*	United States of Ar	SNPRC		Kendall et al., 2024	SNPRC	YES
31131	*Papio*	*hybrid*	United States of Ar	SNPRC		Kendall et al., 2024	SNPRC	YES
31130	*Papio*	*hybrid*	United States of Ar	SNPRC		Kendall et al., 2024	SNPRC	YES
31124	*Papio*	*hybrid*	United States of Ar	SNPRC		Kendall et al., 2024	SNPRC	YES
31120	*Papio*	*hybrid*	United States of Ar	SNPRC		Kendall et al., 2024	SNPRC	YES
31115	*Papio*	*hybrid*	United States of Ar	SNPRC		Kendall et al., 2024	SNPRC	YES
31110	*Papio*	*hybrid*	United States of Ar	SNPRC		Kendall et al., 2024	SNPRC	YES
31086	*Papio*	*hybrid*	United States of Ar	SNPRC		Kendall et al., 2024	SNPRC	YES
31072	*Papio*	*hybrid*	United States of Ar	SNPRC		Kendall et al., 2024	SNPRC	YES
31065	*Papio*	*hybrid*	United States of Ar	SNPRC		Kendall et al., 2024	SNPRC	YES
31064	*Papio*	*hybrid*	United States of Ar	SNPRC		Kendall et al., 2024	SNPRC	YES
31035	*Papio*	*hybrid*	United States of Ar	SNPRC		Kendall et al., 2024	SNPRC	YES
31028	*Papio*	*hybrid*	United States of Ar	SNPRC		Kendall et al., 2024	SNPRC	YES
31026	*Papio*	*hybrid*	United States of Ar	SNPRC		Kendall et al., 2024	SNPRC	YES
31025	*Papio*	*hybrid*	United States of Ar	SNPRC		Kendall et al., 2024	SNPRC	YES
31021	*Papio*	*hybrid*	United States of Ar	SNPRC		Kendall et al., 2024	SNPRC	YES
31016	*Papio*	*hybrid*	United States of Ar	SNPRC		Kendall et al., 2024	SNPRC	YES
31015	*Papio*	*hybrid*	United States of Ar	SNPRC		Kendall et al., 2024	SNPRC	YES
31011	*Papio*	*hybrid*	United States of Ar	SNPRC		Kendall et al., 2024	SNPRC	YES
30974	*Papio*	*hybrid*	United States of Ar	SNPRC		Kendall et al., 2024	SNPRC	YES
30973	*Papio*	*hybrid*	United States of Ar	SNPRC		Kendall et al., 2024	SNPRC	YES
30961	*Papio*	*hybrid*	United States of Ar	SNPRC		Kendall et al., 2024	SNPRC	YES
30934	*Papio*	*hybrid*	United States of Ar	SNPRC		Kendall et al., 2024	SNPRC	YES
30933	*Papio*	*hybrid*	United States of Ar	SNPRC		Kendall et al., 2024	SNPRC	YES
30927	*Papio*	*hybrid*	United States of Ar	SNPRC		Kendall et al., 2024	SNPRC	YES
30920	*Papio*	*hybrid*	United States of Ar	SNPRC		Kendall et al., 2024	SNPRC	YES
30917	*Papio*	*hybrid*	United States of Ar	SNPRC		Kendall et al., 2024	SNPRC	YES
30907	*Papio*	*hybrid*	United States of Ar	SNPRC		Kendall et al., 2024	SNPRC	YES
30889	*Papio*	*hybrid*	United States of Ar	SNPRC		Kendall et al., 2024	SNPRC	YES
30886	*Papio*	*hybrid*	United States of Ar	SNPRC		Kendall et al., 2024	SNPRC	YES
30878	*Papio*	*hybrid*	United States of Ar	SNPRC		Kendall et al., 2024	SNPRC	YES
30871	*Papio*	*hybrid*	United States of Ar	SNPRC		Kendall et al., 2024	SNPRC	YES
30772	*Papio*	*hybrid*	United States of Ar	SNPRC		Kendall et al., 2024	SNPRC	YES
30752	*Papio*	*hybrid*	United States of Ar	SNPRC		Kendall et al., 2024	SNPRC	YES
30751	*Papio*	*hybrid*	United States of Ar	SNPRC		Kendall et al., 2024	SNPRC	YES
30749	*Papio*	*hybrid*	United States of Ar	SNPRC		Kendall et al., 2024	SNPRC	YES
30699	*Papio*	*hybrid*	United States of Ar	SNPRC		Kendall et al., 2024	SNPRC	YES
30673	*Papio*	*hybrid*	United States of Ar	SNPRC		Kendall et al., 2024	SNPRC	YES
30662	*Papio*	*hybrid*	United States of Ar	SNPRC		Kendall et al., 2024	SNPRC	YES
30660	*Papio*	*hybrid*	United States of Ar	SNPRC		Kendall et al., 2024	SNPRC	YES
30650	*Papio*	*hybrid*	United States of Ar	SNPRC		Kendall et al., 2024	SNPRC	YES
30636	*Papio*	*hybrid*	United States of Ar	SNPRC		Kendall et al., 2024	SNPRC	YES
30614	*Papio*	*hybrid*	United States of Ar	SNPRC		Kendall et al., 2024	SNPRC	YES
30611	*Papio*	*hybrid*	United States of Ar	SNPRC		Kendall et al., 2024	SNPRC	YES
30609	*Papio*	*hybrid*	United States of Ar	SNPRC		Kendall et al., 2024	SNPRC	YES
30564	*Papio*	*hybrid*	United States of Ar	SNPRC		Kendall et al., 2024	SNPRC	YES
30508	*Papio*	*hybrid*	United States of Ar	SNPRC		Kendall et al., 2024	SNPRC	YES
30424	*Papio*	*hybrid*	United States of Ar	SNPRC		Kendall et al., 2024	SNPRC	YES
30373	*Papio*	*hybrid*	United States of Ar	SNPRC		Kendall et al., 2024	SNPRC	YES
30366	*Papio*	*hybrid*	United States of Ar	SNPRC		Kendall et al., 2024	SNPRC	YES
30320	*Papio*	*hybrid*	United States of Ar	SNPRC		Kendall et al., 2024	SNPRC	YES
30104	*Papio*	*hybrid*	United States of Ar	SNPRC		Kendall et al., 2024	SNPRC	YES
30033	*Papio*	*hybrid*	United States of Ar	SNPRC		Kendall et al., 2024	SNPRC	YES
30008	*Papio*	*hybrid*	United States of Ar	SNPRC		Kendall et al., 2024	SNPRC	YES
29758	*Papio*	*hybrid*	United States of Ar	SNPRC		Kendall et al., 2024	SNPRC	YES
29565	*Papio*	*hybrid*	United States of Ar	SNPRC		Kendall et al., 2024	SNPRC	YES
29368	*Papio*	*hybrid*	United States of Ar	SNPRC		Kendall et al., 2024	SNPRC	YES
29333	*Papio*	*hybrid*	United States of Ar	SNPRC		Kendall et al., 2024	SNPRC	YES
29156	*Papio*	*hybrid*	United States of Ar	SNPRC		Kendall et al., 2024	SNPRC	YES
28949	*Papio*	*hybrid*	United States of Ar	SNPRC		Kendall et al., 2024	SNPRC	YES
28925	*Papio*	*hybrid*	United States of Ar	SNPRC		Kendall et al., 2024	SNPRC	YES
28830	*Papio*	*hybrid*	United States of Ar	SNPRC		Kendall et al., 2024	SNPRC	YES
28806	*Papio*	*hybrid*	United States of Ar	SNPRC		Kendall et al., 2024	SNPRC	YES
28802	*Papio*	*hybrid*	United States of Ar	SNPRC		Kendall et al., 2024	SNPRC	YES

Sample ID/ Acces	Genus	Species	Country	Locality	Mitochondrial Cla	Source	notes	Mitogenome newly assembled
28801	*Papio*	*hybrid*	United States of Ar	SNPRC		Kendall et al., 2024	SNPRC	YES
28799	*Papio*	*hybrid*	United States of Ar	SNPRC		Kendall et al., 2024	SNPRC	YES
28779	*Papio*	*hybrid*	United States of Ar	SNPRC		Kendall et al., 2024	SNPRC	YES
28742	*Papio*	*hybrid*	United States of Ar	SNPRC		Kendall et al., 2024	SNPRC	YES
28740	*Papio*	*hybrid*	United States of Ar	SNPRC		Kendall et al., 2024	SNPRC	YES
28729	*Papio*	*hybrid*	United States of Ar	SNPRC		Kendall et al., 2024	SNPRC	YES
28635	*Papio*	*hybrid*	United States of Ar	SNPRC		Kendall et al., 2024	SNPRC	YES
28618	*Papio*	*hybrid*	United States of Ar	SNPRC		Kendall et al., 2024	SNPRC	YES
28609	*Papio*	*hybrid*	United States of Ar	SNPRC		Kendall et al., 2024	SNPRC	YES
28591	*Papio*	*hybrid*	United States of Ar	SNPRC		Kendall et al., 2024	SNPRC	YES
28576	*Papio*	*hybrid*	United States of Ar	SNPRC		Kendall et al., 2024	SNPRC	YES
28497	*Papio*	*hybrid*	United States of Ar	SNPRC		Kendall et al., 2024	SNPRC	YES
28495	*Papio*	*hybrid*	United States of Ar	SNPRC		Kendall et al., 2024	SNPRC	YES
28478	*Papio*	*hybrid*	United States of Ar	SNPRC		Kendall et al., 2024	SNPRC	YES
28438	*Papio*	*hybrid*	United States of Ar	SNPRC		Kendall et al., 2024	SNPRC	YES
28436	*Papio*	*hybrid*	United States of Ar	SNPRC		Kendall et al., 2024	SNPRC	YES
28431	*Papio*	*hybrid*	United States of Ar	SNPRC		Kendall et al., 2024	SNPRC	YES
28428	*Papio*	*hybrid*	United States of Ar	SNPRC		Kendall et al., 2024	SNPRC	YES
28417	*Papio*	*hybrid*	United States of Ar	SNPRC		Kendall et al., 2024	SNPRC	YES
28416	*Papio*	*hybrid*	United States of Ar	SNPRC		Kendall et al., 2024	SNPRC	YES
28400	*Papio*	*hybrid*	United States of Ar	SNPRC		Kendall et al., 2024	SNPRC	YES
28386	*Papio*	*hybrid*	United States of Ar	SNPRC		Kendall et al., 2024	SNPRC	YES
28385	*Papio*	*hybrid*	United States of Ar	SNPRC		Kendall et al., 2024	SNPRC	YES
28376	*Papio*	*hybrid*	United States of Ar	SNPRC		Kendall et al., 2024	SNPRC	YES
28375	*Papio*	*hybrid*	United States of Ar	SNPRC		Kendall et al., 2024	SNPRC	YES
28368	*Papio*	*hybrid*	United States of Ar	SNPRC		Kendall et al., 2024	SNPRC	YES
28328	*Papio*	*hybrid*	United States of Ar	SNPRC		Kendall et al., 2024	SNPRC	YES
28304	*Papio*	*hybrid*	United States of Ar	SNPRC		Kendall et al., 2024	SNPRC	YES
28285	*Papio*	*hybrid*	United States of Ar	SNPRC		Kendall et al., 2024	SNPRC	YES
28281	*Papio*	*hybrid*	United States of Ar	SNPRC		Kendall et al., 2024	SNPRC	YES
28279	*Papio*	*hybrid*	United States of Ar	SNPRC		Kendall et al., 2024	SNPRC	YES
28274	*Papio*	*hybrid*	United States of Ar	SNPRC		Kendall et al., 2024	SNPRC	YES
28269	*Papio*	*hybrid*	United States of Ar	SNPRC		Kendall et al., 2024	SNPRC	YES
28259	*Papio*	*hybrid*	United States of Ar	SNPRC		Kendall et al., 2024	SNPRC	YES
28246	*Papio*	*hybrid*	United States of Ar	SNPRC		Kendall et al., 2024	SNPRC	YES
28212	*Papio*	*hybrid*	United States of Ar	SNPRC		Kendall et al., 2024	SNPRC	YES
28183	*Papio*	*hybrid*	United States of Ar	SNPRC		Kendall et al., 2024	SNPRC	YES
28102	*Papio*	*hybrid*	United States of Ar	SNPRC		Kendall et al., 2024	SNPRC	YES
28076	*Papio*	*hybrid*	United States of Ar	SNPRC		Kendall et al., 2024	SNPRC	YES
28075	*Papio*	*hybrid*	United States of Ar	SNPRC		Kendall et al., 2024	SNPRC	YES
28074	*Papio*	*hybrid*	United States of Ar	SNPRC		Kendall et al., 2024	SNPRC	YES
28037	*Papio*	*hybrid*	United States of Ar	SNPRC		Kendall et al., 2024	SNPRC	YES
28021	*Papio*	*hybrid*	United States of Ar	SNPRC		Kendall et al., 2024	SNPRC	YES
28003	*Papio*	*hybrid*	United States of Ar	SNPRC		Kendall et al., 2024	SNPRC	YES
27999	*Papio*	*hybrid*	United States of Ar	SNPRC		Kendall et al., 2024	SNPRC	YES
27989	*Papio*	*hybrid*	United States of Ar	SNPRC		Kendall et al., 2024	SNPRC	YES
27985	*Papio*	*hybrid*	United States of Ar	SNPRC		Kendall et al., 2024	SNPRC	YES
27908	*Papio*	*hybrid*	United States of Ar	SNPRC		Kendall et al., 2024	SNPRC	YES
27889	*Papio*	*hybrid*	United States of Ar	SNPRC		Kendall et al., 2024	SNPRC	YES
27885	*Papio*	*hybrid*	United States of Ar	SNPRC		Kendall et al., 2024	SNPRC	YES
27874	*Papio*	*hybrid*	United States of Ar	SNPRC		Kendall et al., 2024	SNPRC	YES
27844	*Papio*	*hybrid*	United States of Ar	SNPRC		Kendall et al., 2024	SNPRC	YES
27841	*Papio*	*hybrid*	United States of Ar	SNPRC		Kendall et al., 2024	SNPRC	YES
27827	*Papio*	*hybrid*	United States of Ar	SNPRC		Kendall et al., 2024	SNPRC	YES
27820	*Papio*	*hybrid*	United States of Ar	SNPRC		Kendall et al., 2024	SNPRC	YES
27814	*Papio*	*hybrid*	United States of Ar	SNPRC		Kendall et al., 2024	SNPRC	YES
27811	*Papio*	*hybrid*	United States of Ar	SNPRC		Kendall et al., 2024	SNPRC	YES
27742	*Papio*	*hybrid*	United States of Ar	SNPRC		Kendall et al., 2024	SNPRC	YES
27686	*Papio*	*hybrid*	United States of Ar	SNPRC		Kendall et al., 2024	SNPRC	YES
27668	*Papio*	*hybrid*	United States of Ar	SNPRC		Kendall et al., 2024	SNPRC	YES
27666	*Papio*	*hybrid*	United States of Ar	SNPRC		Kendall et al., 2024	SNPRC	YES
27643	*Papio*	*hybrid*	United States of Ar	SNPRC		Kendall et al., 2024	SNPRC	YES
27622	*Papio*	*hybrid*	United States of Ar	SNPRC		Kendall et al., 2024	SNPRC	YES
27594	*Papio*	*hybrid*	United States of Ar	SNPRC		Kendall et al., 2024	SNPRC	YES
27592	*Papio*	*hybrid*	United States of Ar	SNPRC		Kendall et al., 2024	SNPRC	YES
27525	*Papio*	*hybrid*	United States of Ar	SNPRC		Kendall et al., 2024	SNPRC	YES
27503	*Papio*	*hybrid*	United States of Ar	SNPRC		Kendall et al., 2024	SNPRC	YES
27472	*Papio*	*hybrid*	United States of Ar	SNPRC		Kendall et al., 2024	SNPRC	YES
27433	*Papio*	*hybrid*	United States of Ar	SNPRC		Kendall et al., 2024	SNPRC	YES
27358	*Papio*	*hybrid*	United States of Ar	SNPRC		Kendall et al., 2024	SNPRC	YES
27351	*Papio*	*hybrid*	United States of Ar	SNPRC		Kendall et al., 2024	SNPRC	YES
27306	*Papio*	*hybrid*	United States of Ar	SNPRC		Kendall et al., 2024	SNPRC	YES
27267	*Papio*	*hybrid*	United States of Ar	SNPRC		Kendall et al., 2024	SNPRC	YES
27237	*Papio*	*hybrid*	United States of Ar	SNPRC		Kendall et al., 2024	SNPRC	YES
27230	*Papio*	*hybrid*	United States of Ar	SNPRC		Kendall et al., 2024	SNPRC	YES
27199	*Papio*	*hybrid*	United States of Ar	SNPRC		Kendall et al., 2024	SNPRC	YES
27193	*Papio*	*hybrid*	United States of Ar	SNPRC		Kendall et al., 2024	SNPRC	YES
27190	*Papio*	*hybrid*	United States of Ar	SNPRC		Kendall et al., 2024	SNPRC	YES
27181	*Papio*	*hybrid*	United States of Ar	SNPRC		Kendall et al., 2024	SNPRC	YES
27166	*Papio*	*hybrid*	United States of Ar	SNPRC		Kendall et al., 2024	SNPRC	YES
27036	*Papio*	*hybrid*	United States of Ar	SNPRC		Kendall et al., 2024	SNPRC	YES
27033	*Papio*	*hybrid*	United States of Ar	SNPRC		Kendall et al., 2024	SNPRC	YES
26988	*Papio*	*hybrid*	United States of Ar	SNPRC		Kendall et al., 2024	SNPRC	YES
26980	*Papio*	*hybrid*	United States of Ar	SNPRC		Kendall et al., 2024	SNPRC	YES

Sample ID/ Acces	Genus	Species	Country	Locality	Mitochondrial Cla	Source	notes	Mitogenome newly assembled
26800	*Papio*	*hybrid*	United States of Ar	SNPRC		Kendall et al., 2024	SNPRC	YES
26498	*Papio*	*hybrid*	United States of Ar	SNPRC		Kendall et al., 2024	SNPRC	YES
26355	*Papio*	*hybrid*	United States of Ar	SNPRC		Kendall et al., 2024	SNPRC	YES
26324	*Papio*	*hybrid*	United States of Ar	SNPRC		Kendall et al., 2024	SNPRC	YES
26318	*Papio*	*hybrid*	United States of Ar	SNPRC		Kendall et al., 2024	SNPRC	YES
26316	*Papio*	*hybrid*	United States of Ar	SNPRC		Kendall et al., 2024	SNPRC	YES
26310	*Papio*	*hybrid*	United States of Ar	SNPRC		Kendall et al., 2024	SNPRC	YES
26198	*Papio*	*hybrid*	United States of Ar	SNPRC		Kendall et al., 2024	SNPRC	YES
26196	*Papio*	*hybrid*	United States of Ar	SNPRC		Kendall et al., 2024	SNPRC	YES
26138	*Papio*	*hybrid*	United States of Ar	SNPRC		Kendall et al., 2024	SNPRC	YES
26101	*Papio*	*hybrid*	United States of Ar	SNPRC		Kendall et al., 2024	SNPRC	YES
25593	*Papio*	*hybrid*	United States of Ar	SNPRC		Kendall et al., 2024	SNPRC	YES
25409	*Papio*	*hybrid*	United States of Ar	SNPRC		Kendall et al., 2024	SNPRC	YES
25355	*Papio*	*hybrid*	United States of Ar	SNPRC		Kendall et al., 2024	SNPRC	YES
25354	*Papio*	*hybrid*	United States of Ar	SNPRC		Kendall et al., 2024	SNPRC	YES
25347	*Papio*	*hybrid*	United States of Ar	SNPRC		Kendall et al., 2024	SNPRC	YES
20117	*Papio*	*hybrid*	United States of Ar	SNPRC		Kendall et al., 2024	SNPRC	YES
19750	*Papio*	*hybrid*	United States of Ar	SNPRC		Kendall et al., 2024	SNPRC	YES
19679	*Papio*	*hybrid*	United States of Ar	SNPRC		Kendall et al., 2024	SNPRC	YES
19674	*Papio*	*hybrid*	United States of Ar	SNPRC		Kendall et al., 2024	SNPRC	YES
19658	*Papio*	*hybrid*	United States of Ar	SNPRC		Kendall et al., 2024	SNPRC	YES
19381	*Papio*	*hybrid*	United States of Ar	SNPRC		Kendall et al., 2024	SNPRC	YES
19378	*Papio*	*hybrid*	United States of Ar	SNPRC		Kendall et al., 2024	SNPRC	YES
19376	*Papio*	*hybrid*	United States of Ar	SNPRC		Kendall et al., 2024	SNPRC	YES
19371	*Papio*	*hybrid*	United States of Ar	SNPRC		Kendall et al., 2024	SNPRC	YES
19365	*Papio*	*hybrid*	United States of Ar	SNPRC		Kendall et al., 2024	SNPRC	YES
19359	*Papio*	*hybrid*	United States of Ar	SNPRC		Kendall et al., 2024	SNPRC	YES
19348	*Papio*	*hybrid*	United States of Ar	SNPRC		Kendall et al., 2024	SNPRC	YES
19237	*Papio*	*hybrid*	United States of Ar	SNPRC		Kendall et al., 2024	SNPRC	YES
19215	*Papio*	*hybrid*	United States of Ar	SNPRC		Kendall et al., 2024	SNPRC	YES
19207	*Papio*	*hybrid*	United States of Ar	SNPRC		Kendall et al., 2024	SNPRC	YES
19181	*Papio*	*hybrid*	United States of Ar	SNPRC		Kendall et al., 2024	SNPRC	YES
19173	*Papio*	*hybrid*	United States of Ar	SNPRC		Kendall et al., 2024	SNPRC	YES
19165	*Papio*	*hybrid*	United States of Ar	SNPRC		Kendall et al., 2024	SNPRC	YES
19154	*Papio*	*hybrid*	United States of Ar	SNPRC		Kendall et al., 2024	SNPRC	YES
19144	*Papio*	*hybrid*	United States of Ar	SNPRC		Kendall et al., 2024	SNPRC	YES
19012	*Papio*	*hybrid*	United States of Ar	SNPRC		Kendall et al., 2024	SNPRC	YES
18974	*Papio*	*hybrid*	United States of Ar	SNPRC		Kendall et al., 2024	SNPRC	YES
18972	*Papio*	*hybrid*	United States of Ar	SNPRC		Kendall et al., 2024	SNPRC	YES
18938	*Papio*	*hybrid*	United States of Ar	SNPRC		Kendall et al., 2024	SNPRC	YES
18929	*Papio*	*hybrid*	United States of Ar	SNPRC		Kendall et al., 2024	SNPRC	YES
18917	*Papio*	*hybrid*	United States of Ar	SNPRC		Kendall et al., 2024	SNPRC	YES
18892	*Papio*	*hybrid*	United States of Ar	SNPRC		Kendall et al., 2024	SNPRC	YES
18866	*Papio*	*hybrid*	United States of Ar	SNPRC		Kendall et al., 2024	SNPRC	YES
18841	*Papio*	*hybrid*	United States of Ar	SNPRC		Kendall et al., 2024	SNPRC	YES
18840	*Papio*	*hybrid*	United States of Ar	SNPRC		Kendall et al., 2024	SNPRC	YES
18818	*Papio*	*hybrid*	United States of Ar	SNPRC		Kendall et al., 2024	SNPRC	YES
18789	*Papio*	*hybrid*	United States of Ar	SNPRC		Kendall et al., 2024	SNPRC	YES
18778	*Papio*	*hybrid*	United States of Ar	SNPRC		Kendall et al., 2024	SNPRC	YES
18715	*Papio*	*hybrid*	United States of Ar	SNPRC		Kendall et al., 2024	SNPRC	YES
18713	*Papio*	*hybrid*	United States of Ar	SNPRC		Kendall et al., 2024	SNPRC	YES
18672	*Papio*	*hybrid*	United States of Ar	SNPRC		Kendall et al., 2024	SNPRC	YES
18599	*Papio*	*hybrid*	United States of Ar	SNPRC		Kendall et al., 2024	SNPRC	YES
18586	*Papio*	*hybrid*	United States of Ar	SNPRC		Kendall et al., 2024	SNPRC	YES
18529	*Papio*	*hybrid*	United States of Ar	SNPRC		Kendall et al., 2024	SNPRC	YES
18528	*Papio*	*hybrid*	United States of Ar	SNPRC		Kendall et al., 2024	SNPRC	YES
18523	*Papio*	*hybrid*	United States of Ar	SNPRC		Kendall et al., 2024	SNPRC	YES
18469	*Papio*	*hybrid*	United States of Ar	SNPRC		Kendall et al., 2024	SNPRC	YES
18431	*Papio*	*hybrid*	United States of Ar	SNPRC		Kendall et al., 2024	SNPRC	YES
18395	*Papio*	*hybrid*	United States of Ar	SNPRC		Kendall et al., 2024	SNPRC	YES
18385	*Papio*	*hybrid*	United States of Ar	SNPRC		Kendall et al., 2024	SNPRC	YES
18168	*Papio*	*hybrid*	United States of Ar	SNPRC		Kendall et al., 2024	SNPRC	YES
18144	*Papio*	*hybrid*	United States of Ar	SNPRC		Kendall et al., 2024	SNPRC	YES
18141	*Papio*	*hybrid*	United States of Ar	SNPRC		Kendall et al., 2024	SNPRC	YES
18024	*Papio*	*hybrid*	United States of Ar	SNPRC		Kendall et al., 2024	SNPRC	YES
18022	*Papio*	*hybrid*	United States of Ar	SNPRC		Kendall et al., 2024	SNPRC	YES
18019	*Papio*	*hybrid*	United States of Ar	SNPRC		Kendall et al., 2024	SNPRC	YES
18015	*Papio*	*hybrid*	United States of Ar	SNPRC		Kendall et al., 2024	SNPRC	YES
18004	*Papio*	*hybrid*	United States of Ar	SNPRC		Kendall et al., 2024	SNPRC	YES
17999	*Papio*	*hybrid*	United States of Ar	SNPRC		Kendall et al., 2024	SNPRC	YES
17998	*Papio*	*hybrid*	United States of Ar	SNPRC		Kendall et al., 2024	SNPRC	YES
17997	*Papio*	*hybrid*	United States of Ar	SNPRC		Kendall et al., 2024	SNPRC	YES
17981	*Papio*	*hybrid*	United States of Ar	SNPRC		Kendall et al., 2024	SNPRC	YES
17971	*Papio*	*hybrid*	United States of Ar	SNPRC		Kendall et al., 2024	SNPRC	YES
17970	*Papio*	*hybrid*	United States of Ar	SNPRC		Kendall et al., 2024	SNPRC	YES
17969	*Papio*	*hybrid*	United States of Ar	SNPRC		Kendall et al., 2024	SNPRC	YES
17929	*Papio*	*hybrid*	United States of Ar	SNPRC		Kendall et al., 2024	SNPRC	YES
17916	*Papio*	*hybrid*	United States of Ar	SNPRC		Kendall et al., 2024	SNPRC	YES
17903	*Papio*	*hybrid*	United States of Ar	SNPRC		Kendall et al., 2024	SNPRC	YES
17898	*Papio*	*hybrid*	United States of Ar	SNPRC		Kendall et al., 2024	SNPRC	YES
17891	*Papio*	*hybrid*	United States of Ar	SNPRC		Kendall et al., 2024	SNPRC	YES
17879	*Papio*	*hybrid*	United States of Ar	SNPRC		Kendall et al., 2024	SNPRC	YES
17873	*Papio*	*hybrid*	United States of Ar	SNPRC		Kendall et al., 2024	SNPRC	YES
17871	*Papio*	*hybrid*	United States of Ar	SNPRC		Kendall et al., 2024	SNPRC	YES

Sample ID/ Acces	Genus	Species	Country	Locality	Mitochondrial Cla	Source	notes	Mitogenome newly assembled
17852	*Papio*	*hybrid*	United States of Ar	SNPRC		Kendall et al., 2024	SNPRC	YES
17851	*Papio*	*hybrid*	United States of Ar	SNPRC		Kendall et al., 2024	SNPRC	YES
17840	*Papio*	*hybrid*	United States of Ar	SNPRC		Kendall et al., 2024	SNPRC	YES
17833	*Papio*	*hybrid*	United States of Ar	SNPRC		Kendall et al., 2024	SNPRC	YES
17832	*Papio*	*hybrid*	United States of Ar	SNPRC		Kendall et al., 2024	SNPRC	YES
17829	*Papio*	*hybrid*	United States of Ar	SNPRC		Kendall et al., 2024	SNPRC	YES
17826	*Papio*	*hybrid*	United States of Ar	SNPRC		Kendall et al., 2024	SNPRC	YES
17795	*Papio*	*hybrid*	United States of Ar	SNPRC		Kendall et al., 2024	SNPRC	YES
17792	*Papio*	*hybrid*	United States of Ar	SNPRC		Kendall et al., 2024	SNPRC	YES
17776	*Papio*	*hybrid*	United States of Ar	SNPRC		Kendall et al., 2024	SNPRC	YES
17340	*Papio*	*hybrid*	United States of Ar	SNPRC		Kendall et al., 2024	SNPRC	YES
17328	*Papio*	*hybrid*	United States of Ar	SNPRC		Kendall et al., 2024	SNPRC	YES
17325	*Papio*	*hybrid*	United States of Ar	SNPRC		Kendall et al., 2024	SNPRC	YES
17295	*Papio*	*hybrid*	United States of Ar	SNPRC		Kendall et al., 2024	SNPRC	YES
17294	*Papio*	*hybrid*	United States of Ar	SNPRC		Kendall et al., 2024	SNPRC	YES
17268	*Papio*	*hybrid*	United States of Ar	SNPRC		Kendall et al., 2024	SNPRC	YES
17255	*Papio*	*hybrid*	United States of Ar	SNPRC		Kendall et al., 2024	SNPRC	YES
17254	*Papio*	*hybrid*	United States of Ar	SNPRC		Kendall et al., 2024	SNPRC	YES
17233	*Papio*	*hybrid*	United States of Ar	SNPRC		Kendall et al., 2024	SNPRC	YES
17232	*Papio*	*hybrid*	United States of Ar	SNPRC		Kendall et al., 2024	SNPRC	YES
17228	*Papio*	*hybrid*	United States of Ar	SNPRC		Kendall et al., 2024	SNPRC	YES
17222	*Papio*	*hybrid*	United States of Ar	SNPRC		Kendall et al., 2024	SNPRC	YES
17199	*Papio*	*hybrid*	United States of Ar	SNPRC		Kendall et al., 2024	SNPRC	YES
17190	*Papio*	*hybrid*	United States of Ar	SNPRC		Kendall et al., 2024	SNPRC	YES
17184	*Papio*	*hybrid*	United States of Ar	SNPRC		Kendall et al., 2024	SNPRC	YES
17174	*Papio*	*hybrid*	United States of Ar	SNPRC		Kendall et al., 2024	SNPRC	YES
17165	*Papio*	*hybrid*	United States of Ar	SNPRC		Kendall et al., 2024	SNPRC	YES
17159	*Papio*	*hybrid*	United States of Ar	SNPRC		Kendall et al., 2024	SNPRC	YES
17158	*Papio*	*hybrid*	United States of Ar	SNPRC		Kendall et al., 2024	SNPRC	YES
17153	*Papio*	*hybrid*	United States of Ar	SNPRC		Kendall et al., 2024	SNPRC	YES
17141	*Papio*	*hybrid*	United States of Ar	SNPRC		Kendall et al., 2024	SNPRC	YES
17139	*Papio*	*hybrid*	United States of Ar	SNPRC		Kendall et al., 2024	SNPRC	YES
17112	*Papio*	*hybrid*	United States of Ar	SNPRC		Kendall et al., 2024	SNPRC	YES
17111	*Papio*	*hybrid*	United States of Ar	SNPRC		Kendall et al., 2024	SNPRC	YES
17024	*Papio*	*hybrid*	United States of Ar	SNPRC		Kendall et al., 2024	SNPRC	YES but deleted due to high missing/ambigous bases
17006	*Papio*	*hybrid*	United States of Ar	SNPRC		Kendall et al., 2024	SNPRC	YES
16999	*Papio*	*hybrid*	United States of Ar	SNPRC		Kendall et al., 2024	SNPRC	YES
16986	*Papio*	*hybrid*	United States of Ar	SNPRC		Kendall et al., 2024	SNPRC	YES
16890	*Papio*	*hybrid*	United States of Ar	SNPRC		Kendall et al., 2024	SNPRC	YES
16889	*Papio*	*hybrid*	United States of Ar	SNPRC		Kendall et al., 2024	SNPRC	YES
16880	*Papio*	*hybrid*	United States of Ar	SNPRC		Kendall et al., 2024	SNPRC	YES
16879	*Papio*	*hybrid*	United States of Ar	SNPRC		Kendall et al., 2024	SNPRC	YES
16873	*Papio*	*hybrid*	United States of Ar	SNPRC		Kendall et al., 2024	SNPRC	YES
16866	*Papio*	*hybrid*	United States of Ar	SNPRC		Kendall et al., 2024	SNPRC	YES
16862	*Papio*	*hybrid*	United States of Ar	SNPRC		Kendall et al., 2024	SNPRC	YES
16861	*Papio*	*hybrid*	United States of Ar	SNPRC		Kendall et al., 2024	SNPRC	YES
16856	*Papio*	*hybrid*	United States of Ar	SNPRC		Kendall et al., 2024	SNPRC	YES
16853	*Papio*	*hybrid*	United States of Ar	SNPRC		Kendall et al., 2024	SNPRC	YES
16847	*Papio*	*hybrid*	United States of Ar	SNPRC		Kendall et al., 2024	SNPRC	YES
16829	*Papio*	*hybrid*	United States of Ar	SNPRC		Kendall et al., 2024	SNPRC	YES
16827	*Papio*	*hybrid*	United States of Ar	SNPRC		Kendall et al., 2024	SNPRC	YES
16823	*Papio*	*hybrid*	United States of Ar	SNPRC		Kendall et al., 2024	SNPRC	YES
16816	*Papio*	*hybrid*	United States of Ar	SNPRC		Kendall et al., 2024	SNPRC	YES
16815	*Papio*	*hybrid*	United States of Ar	SNPRC		Kendall et al., 2024	SNPRC	YES
16808	*Papio*	*hybrid*	United States of Ar	SNPRC		Kendall et al., 2024	SNPRC	YES
16798	*Papio*	*hybrid*	United States of Ar	SNPRC		Kendall et al., 2024	SNPRC	YES
16787	*Papio*	*hybrid*	United States of Ar	SNPRC		Kendall et al., 2024	SNPRC	YES
16780	*Papio*	*hybrid*	United States of Ar	SNPRC		Kendall et al., 2024	SNPRC	YES
16775	*Papio*	*hybrid*	United States of Ar	SNPRC		Kendall et al., 2024	SNPRC	YES
16761	*Papio*	*hybrid*	United States of Ar	SNPRC		Kendall et al., 2024	SNPRC	YES
16748	*Papio*	*hybrid*	United States of Ar	SNPRC		Kendall et al., 2024	SNPRC	YES
16744	*Papio*	*hybrid*	United States of Ar	SNPRC		Kendall et al., 2024	SNPRC	YES
16743	*Papio*	*hybrid*	United States of Ar	SNPRC		Kendall et al., 2024	SNPRC	YES
16741	*Papio*	*hybrid*	United States of Ar	SNPRC		Kendall et al., 2024	SNPRC	YES
16731	*Papio*	*hybrid*	United States of Ar	SNPRC		Kendall et al., 2024	SNPRC	YES
16719	*Papio*	*hybrid*	United States of Ar	SNPRC		Kendall et al., 2024	SNPRC	YES
16708	*Papio*	*hybrid*	United States of Ar	SNPRC		Kendall et al., 2024	SNPRC	YES
16702	*Papio*	*hybrid*	United States of Ar	SNPRC		Kendall et al., 2024	SNPRC	YES
16692	*Papio*	*hybrid*	United States of Ar	SNPRC		Kendall et al., 2024	SNPRC	YES
16678	*Papio*	*hybrid*	United States of Ar	SNPRC		Kendall et al., 2024	SNPRC	YES
16671	*Papio*	*hybrid*	United States of Ar	SNPRC		Kendall et al., 2024	SNPRC	YES
16569	*Papio*	*hybrid*	United States of Ar	SNPRC		Kendall et al., 2024	SNPRC	YES
16562	*Papio*	*hybrid*	United States of Ar	SNPRC		Kendall et al., 2024	SNPRC	YES
16550	*Papio*	*hybrid*	United States of Ar	SNPRC		Kendall et al., 2024	SNPRC	YES
16548	*Papio*	*hybrid*	United States of Ar	SNPRC		Kendall et al., 2024	SNPRC	YES
16538	*Papio*	*hybrid*	United States of Ar	SNPRC		Kendall et al., 2024	SNPRC	YES
16527	*Papio*	*hybrid*	United States of Ar	SNPRC		Kendall et al., 2024	SNPRC	YES
16524	*Papio*	*hybrid*	United States of Ar	SNPRC		Kendall et al., 2024	SNPRC	YES
16517	*Papio*	*hybrid*	United States of Ar	SNPRC		Kendall et al., 2024	SNPRC	YES
16510	*Papio*	*hybrid*	United States of Ar	SNPRC		Kendall et al., 2024	SNPRC	YES
16507	*Papio*	*hybrid*	United States of Ar	SNPRC		Kendall et al., 2024	SNPRC	YES
16499	*Papio*	*hybrid*	United States of Ar	SNPRC		Kendall et al., 2024	SNPRC	YES
16486	*Papio*	*hybrid*	United States of Ar	SNPRC		Kendall et al., 2024	SNPRC	YES
16436	*Papio*	*hybrid*	United States of Ar	SNPRC		Kendall et al., 2024	SNPRC	YES

Sample ID/ Acces	Genus	Species	Country	Locality	Mitochondrial Cla	Source	notes	Mitogenome newly assembled
16424	*Papio*	*hybrid*	United States of Ar	SNPRC		Kendall et al., 2024	SNPRC	YES
16420	*Papio*	*hybrid*	United States of Ar	SNPRC		Kendall et al., 2024	SNPRC	YES
16417	*Papio*	*hybrid*	United States of Ar	SNPRC		Kendall et al., 2024	SNPRC	YES
16413	*Papio*	*hybrid*	United States of Ar	SNPRC		Kendall et al., 2024	SNPRC	YES
16409	*Papio*	*hybrid*	United States of Ar	SNPRC		Kendall et al., 2024	SNPRC	YES
16408	*Papio*	*hybrid*	United States of Ar	SNPRC		Kendall et al., 2024	SNPRC	YES
16400	*Papio*	*hybrid*	United States of Ar	SNPRC		Kendall et al., 2024	SNPRC	YES
16391	*Papio*	*hybrid*	United States of Ar	SNPRC		Kendall et al., 2024	SNPRC	YES
16385	*Papio*	*hybrid*	United States of Ar	SNPRC		Kendall et al., 2024	SNPRC	YES
16383	*Papio*	*hybrid*	United States of Ar	SNPRC		Kendall et al., 2024	SNPRC	YES
16372	*Papio*	*hybrid*	United States of Ar	SNPRC		Kendall et al., 2024	SNPRC	YES
16371	*Papio*	*hybrid*	United States of Ar	SNPRC		Kendall et al., 2024	SNPRC	YES
16369	*Papio*	*hybrid*	United States of Ar	SNPRC		Kendall et al., 2024	SNPRC	YES
16363	*Papio*	*hybrid*	United States of Ar	SNPRC		Kendall et al., 2024	SNPRC	YES
16338	*Papio*	*hybrid*	United States of Ar	SNPRC		Kendall et al., 2024	SNPRC	YES
16329	*Papio*	*hybrid*	United States of Ar	SNPRC		Kendall et al., 2024	SNPRC	YES
16321	*Papio*	*hybrid*	United States of Ar	SNPRC		Kendall et al., 2024	SNPRC	YES
16316	*Papio*	*hybrid*	United States of Ar	SNPRC		Kendall et al., 2024	SNPRC	YES
16304	*Papio*	*hybrid*	United States of Ar	SNPRC		Kendall et al., 2024	SNPRC	YES
16300	*Papio*	*hybrid*	United States of Ar	SNPRC		Kendall et al., 2024	SNPRC	YES
16289	*Papio*	*hybrid*	United States of Ar	SNPRC		Kendall et al., 2024	SNPRC	YES
16288	*Papio*	*hybrid*	United States of Ar	SNPRC		Kendall et al., 2024	SNPRC	YES
16263	*Papio*	*hybrid*	United States of Ar	SNPRC		Kendall et al., 2024	SNPRC	YES
16261	*Papio*	*hybrid*	United States of Ar	SNPRC		Kendall et al., 2024	SNPRC	YES
16254	*Papio*	*hybrid*	United States of Ar	SNPRC		Kendall et al., 2024	SNPRC	YES
16246	*Papio*	*hybrid*	United States of Ar	SNPRC		Kendall et al., 2024	SNPRC	YES
16244	*Papio*	*hybrid*	United States of Ar	SNPRC		Kendall et al., 2024	SNPRC	YES
16243	*Papio*	*hybrid*	United States of Ar	SNPRC		Kendall et al., 2024	SNPRC	YES
16122	*Papio*	*hybrid*	United States of Ar	SNPRC		Kendall et al., 2024	SNPRC	YES
16121	*Papio*	*hybrid*	United States of Ar	SNPRC		Kendall et al., 2024	SNPRC	YES
16115	*Papio*	*hybrid*	United States of Ar	SNPRC		Kendall et al., 2024	SNPRC	YES
16112	*Papio*	*hybrid*	United States of Ar	SNPRC		Kendall et al., 2024	SNPRC	YES
16104	*Papio*	*hybrid*	United States of Ar	SNPRC		Kendall et al., 2024	SNPRC	YES
16101	*Papio*	*hybrid*	United States of Ar	SNPRC		Kendall et al., 2024	SNPRC	YES
16094	*Papio*	*hybrid*	United States of Ar	SNPRC		Kendall et al., 2024	SNPRC	YES
16092	*Papio*	*hybrid*	United States of Ar	SNPRC		Kendall et al., 2024	SNPRC	YES
16083	*Papio*	*hybrid*	United States of Ar	SNPRC		Kendall et al., 2024	SNPRC	YES
16072	*Papio*	*hybrid*	United States of Ar	SNPRC		Kendall et al., 2024	SNPRC	YES
16067	*Papio*	*hybrid*	United States of Ar	SNPRC		Kendall et al., 2024	SNPRC	YES
16065	*Papio*	*hybrid*	United States of Ar	SNPRC		Kendall et al., 2024	SNPRC	YES
16059	*Papio*	*hybrid*	United States of Ar	SNPRC		Kendall et al., 2024	SNPRC	YES
16058	*Papio*	*hybrid*	United States of Ar	SNPRC		Kendall et al., 2024	SNPRC	YES
16056	*Papio*	*hybrid*	United States of Ar	SNPRC		Kendall et al., 2024	SNPRC	YES
16019	*Papio*	*hybrid*	United States of Ar	SNPRC		Kendall et al., 2024	SNPRC	YES
16017	*Papio*	*hybrid*	United States of Ar	SNPRC		Kendall et al., 2024	SNPRC	YES
16006	*Papio*	*hybrid*	United States of Ar	SNPRC		Kendall et al., 2024	SNPRC	YES
16000	*Papio*	*hybrid*	United States of Ar	SNPRC		Kendall et al., 2024	SNPRC	YES
15996	*Papio*	*hybrid*	United States of Ar	SNPRC		Kendall et al., 2024	SNPRC	YES
15979	*Papio*	*hybrid*	United States of Ar	SNPRC		Kendall et al., 2024	SNPRC	YES
15975	*Papio*	*hybrid*	United States of Ar	SNPRC		Kendall et al., 2024	SNPRC	YES
15944	*Papio*	*hybrid*	United States of Ar	SNPRC		Kendall et al., 2024	SNPRC	YES
15940	*Papio*	*hybrid*	United States of Ar	SNPRC		Kendall et al., 2024	SNPRC	YES
15938	*Papio*	*hybrid*	United States of Ar	SNPRC		Kendall et al., 2024	SNPRC	YES
15889	*Papio*	*hybrid*	United States of Ar	SNPRC		Kendall et al., 2024	SNPRC	YES
15874	*Papio*	*hybrid*	United States of Ar	SNPRC		Kendall et al., 2024	SNPRC	YES
15870	*Papio*	*hybrid*	United States of Ar	SNPRC		Kendall et al., 2024	SNPRC	YES
15849	*Papio*	*hybrid*	United States of Ar	SNPRC		Kendall et al., 2024	SNPRC	YES
15846	*Papio*	*hybrid*	United States of Ar	SNPRC		Kendall et al., 2024	SNPRC	YES
15845	*Papio*	*hybrid*	United States of Ar	SNPRC		Kendall et al., 2024	SNPRC	YES
15824	*Papio*	*hybrid*	United States of Ar	SNPRC		Kendall et al., 2024	SNPRC	YES
15820	*Papio*	*hybrid*	United States of Ar	SNPRC		Kendall et al., 2024	SNPRC	YES
15736	*Papio*	*hybrid*	United States of Ar	SNPRC		Kendall et al., 2024	SNPRC	YES
15733	*Papio*	*hybrid*	United States of Ar	SNPRC		Kendall et al., 2024	SNPRC	YES
15664	*Papio*	*hybrid*	United States of Ar	SNPRC		Kendall et al., 2024	SNPRC	YES
15659	*Papio*	*hybrid*	United States of Ar	SNPRC		Kendall et al., 2024	SNPRC	YES
15653	*Papio*	*hybrid*	United States of Ar	SNPRC		Kendall et al., 2024	SNPRC	YES
15652	*Papio*	*hybrid*	United States of Ar	SNPRC		Kendall et al., 2024	SNPRC	YES
15633	*Papio*	*hybrid*	United States of Ar	SNPRC		Kendall et al., 2024	SNPRC	YES
15628	*Papio*	*hybrid*	United States of Ar	SNPRC		Kendall et al., 2024	SNPRC	YES
15626	*Papio*	*hybrid*	United States of Ar	SNPRC		Kendall et al., 2024	SNPRC	YES
15600	*Papio*	*hybrid*	United States of Ar	SNPRC		Kendall et al., 2024	SNPRC	YES
15599	*Papio*	*hybrid*	United States of Ar	SNPRC		Kendall et al., 2024	SNPRC	YES
15581	*Papio*	*hybrid*	United States of Ar	SNPRC		Kendall et al., 2024	SNPRC	YES
15575	*Papio*	*hybrid*	United States of Ar	SNPRC		Kendall et al., 2024	SNPRC	YES
15571	*Papio*	*hybrid*	United States of Ar	SNPRC		Kendall et al., 2024	SNPRC	YES
15568	*Papio*	*hybrid*	United States of Ar	SNPRC		Kendall et al., 2024	SNPRC	YES
15566	*Papio*	*hybrid*	United States of Ar	SNPRC		Kendall et al., 2024	SNPRC	YES
15562	*Papio*	*hybrid*	United States of Ar	SNPRC		Kendall et al., 2024	SNPRC	YES
15560	*Papio*	*hybrid*	United States of Ar	SNPRC		Kendall et al., 2024	SNPRC	YES
15553	*Papio*	*hybrid*	United States of Ar	SNPRC		Kendall et al., 2024	SNPRC	YES
15545	*Papio*	*hybrid*	United States of Ar	SNPRC		Kendall et al., 2024	SNPRC	YES
15523	*Papio*	*hybrid*	United States of Ar	SNPRC		Kendall et al., 2024	SNPRC	YES
15509	*Papio*	*hybrid*	United States of Ar	SNPRC		Kendall et al., 2024	SNPRC	YES
15494	*Papio*	*hybrid*	United States of Ar	SNPRC		Kendall et al., 2024	SNPRC	YES

Sample ID/ Acces	Genus	Species	Country	Locality	Mitochondrial Cla	Source	notes	Mitogenome newly assembled			
15486	*Papio*	*hybrid*	United States of Ar	SNPRC		Kendall et al., 2024	SNPRC	YES			
15475	*Papio*	*hybrid*	United States of Ar	SNPRC		Kendall et al., 2024	SNPRC	YES			
15467	*Papio*	*hybrid*	United States of Ar	SNPRC		Kendall et al., 2024	SNPRC	YES			
15458	*Papio*	*hybrid*	United States of Ar	SNPRC		Kendall et al., 2024	SNPRC	YES			
15444	*Papio*	*hybrid*	United States of Ar	SNPRC		Kendall et al., 2024	SNPRC	YES			
15442	*Papio*	*hybrid*	United States of Ar	SNPRC		Kendall et al., 2024	SNPRC	YES			
15421	*Papio*	*hybrid*	United States of Ar	SNPRC		Kendall et al., 2024	SNPRC	YES			
15419	*Papio*	*hybrid*	United States of Ar	SNPRC		Kendall et al., 2024	SNPRC	YES			
15414	*Papio*	*hybrid*	United States of Ar	SNPRC		Kendall et al., 2024	SNPRC	YES			
15308	*Papio*	*hybrid*	United States of Ar	SNPRC		Kendall et al., 2024	SNPRC	YES			
15292	*Papio*	*hybrid*	United States of Ar	SNPRC		Kendall et al., 2024	SNPRC	YES			
15290	*Papio*	*hybrid*	United States of Ar	SNPRC		Kendall et al., 2024	SNPRC	YES			
15286	*Papio*	*hybrid*	United States of Ar	SNPRC		Kendall et al., 2024	SNPRC	YES			
15279	*Papio*	*hybrid*	United States of Ar	SNPRC		Kendall et al., 2024	SNPRC	YES			
15274	*Papio*	*hybrid*	United States of Ar	SNPRC		Kendall et al., 2024	SNPRC	YES			
15267	*Papio*	*hybrid*	United States of Ar	SNPRC		Kendall et al., 2024	SNPRC	YES			
15244	*Papio*	*hybrid*	United States of Ar	SNPRC		Kendall et al., 2024	SNPRC	YES			
15232	*Papio*	*hybrid*	United States of Ar	SNPRC		Kendall et al., 2024	SNPRC	YES			
15225	*Papio*	*hybrid*	United States of Ar	SNPRC		Kendall et al., 2024	SNPRC	YES			
15212	*Papio*	*hybrid*	United States of Ar	SNPRC		Kendall et al., 2024	SNPRC	YES			
15211	*Papio*	*hybrid*	United States of Ar	SNPRC		Kendall et al., 2024	SNPRC	YES			
15197	*Papio*	*hybrid*	United States of Ar	SNPRC		Kendall et al., 2024	SNPRC	YES			
15190	*Papio*	*hybrid*	United States of Ar	SNPRC		Kendall et al., 2024	SNPRC	YES			
15178	*Papio*	*hybrid*	United States of Ar	SNPRC		Kendall et al., 2024	SNPRC	YES			
15175	*Papio*	*hybrid*	United States of Ar	SNPRC		Kendall et al., 2024	SNPRC	YES			
15163	*Papio*	*hybrid*	United States of Ar	SNPRC		Kendall et al., 2024	SNPRC	YES			
15157	*Papio*	*hybrid*	United States of Ar	SNPRC		Kendall et al., 2024	SNPRC	YES			
15156	*Papio*	*hybrid*	United States of Ar	SNPRC		Kendall et al., 2024	SNPRC	YES			
15150	*Papio*	*hybrid*	United States of Ar	SNPRC		Kendall et al., 2024	SNPRC	YES			
15149	*Papio*	*hybrid*	United States of Ar	SNPRC		Kendall et al., 2024	SNPRC	YES			
15122	*Papio*	*hybrid*	United States of Ar	SNPRC		Kendall et al., 2024	SNPRC	YES			
15113	*Papio*	*hybrid*	United States of Ar	SNPRC		Kendall et al., 2024	SNPRC	YES			
15112	*Papio*	*hybrid*	United States of Ar	SNPRC		Kendall et al., 2024	SNPRC	YES			
15107	*Papio*	*hybrid*	United States of Ar	SNPRC		Kendall et al., 2024	SNPRC	YES			
15009	*Papio*	*hybrid*	United States of Ar	SNPRC		Kendall et al., 2024	SNPRC	YES			
14994	*Papio*	*hybrid*	United States of Ar	SNPRC		Kendall et al., 2024	SNPRC	YES			
14989	*Papio*	*hybrid*	United States of Ar	SNPRC		Kendall et al., 2024	SNPRC	YES			
14959	*Papio*	*hybrid*	United States of Ar	SNPRC		Kendall et al., 2024	SNPRC	YES			
14951	*Papio*	*hybrid*	United States of Ar	SNPRC		Kendall et al., 2024	SNPRC	YES			
14948	*Papio*	*hybrid*	United States of Ar	SNPRC		Kendall et al., 2024	SNPRC	YES			
14944	*Papio*	*hybrid*	United States of Ar	SNPRC		Kendall et al., 2024	SNPRC	YES			
14943	*Papio*	*hybrid*	United States of Ar	SNPRC		Kendall et al., 2024	SNPRC	YES			
14930	*Papio*	*hybrid*	United States of Ar	SNPRC		Kendall et al., 2024	SNPRC	YES			
14925	*Papio*	*hybrid*	United States of Ar	SNPRC		Kendall et al., 2024	SNPRC	YES			
14924	*Papio*	*hybrid*	United States of Ar	SNPRC		Kendall et al., 2024	SNPRC	YES			
14922	*Papio*	*hybrid*	United States of Ar	SNPRC		Kendall et al., 2024	SNPRC	YES			
14916	*Papio*	*hybrid*	United States of Ar	SNPRC		Kendall et al., 2024	SNPRC	YES			
14909	*Papio*	*hybrid*	United States of Ar	SNPRC		Kendall et al., 2024	SNPRC	YES			
14908	*Papio*	*hybrid*	United States of Ar	SNPRC		Kendall et al., 2024	SNPRC	YES			
14886	*Papio*	*hybrid*	United States of Ar	SNPRC		Kendall et al., 2024	SNPRC	YES			
14867	*Papio*	*hybrid*	United States of Ar	SNPRC		Kendall et al., 2024	SNPRC	YES			
14865	*Papio*	*hybrid*	United States of Ar	SNPRC		Kendall et al., 2024	SNPRC	YES			
14863	*Papio*	*hybrid*	United States of Ar	SNPRC		Kendall et al., 2024	SNPRC	YES			
14860	*Papio*	*hybrid*	United States of Ar	SNPRC		Kendall et al., 2024	SNPRC	YES			
14852	*Papio*	*hybrid*	United States of Ar	SNPRC		Kendall et al., 2024	SNPRC	YES			
14850	*Papio*	*hybrid*	United States of Ar	SNPRC		Kendall et al., 2024	SNPRC	YES			
14847	*Papio*	*hybrid*	United States of Ar	SNPRC		Kendall et al., 2024	SNPRC	YES			
14833	*Papio*	*hybrid*	United States of Ar	SNPRC		Kendall et al., 2024	SNPRC	YES			
14800	*Papio*	*hybrid*	United States of Ar	SNPRC		Kendall et al., 2024	SNPRC	YES			
14795	*Papio*	*hybrid*	United States of Ar	SNPRC		Kendall et al., 2024	SNPRC	YES			
14756	*Papio*	*hybrid*	United States of Ar	SNPRC		Kendall et al., 2024	SNPRC	YES			
14753	*Papio*	*hybrid*	United States of Ar	SNPRC		Kendall et al., 2024	SNPRC	YES			
14740	*Papio*	*hybrid*	United States of Ar	SNPRC		Kendall et al., 2024	SNPRC	YES			
14712	*Papio*	*hybrid*	United States of Ar	SNPRC		Kendall et al., 2024	SNPRC	YES			
14690	*Papio*	*hybrid*	United States of Ar	SNPRC		Kendall et al., 2024	SNPRC	YES			
14668	*Papio*	*hybrid*	United States of Ar	SNPRC		Kendall et al., 2024	SNPRC	YES			
14652	*Papio*	*hybrid*	United States of Ar	SNPRC		Kendall et al., 2024	SNPRC	YES			
14642	*Papio*	*hybrid*	United States of Ar	SNPRC		Kendall et al., 2024	SNPRC	YES			
14526	*Papio*	*hybrid*	United States of Ar	SNPRC		Kendall et al., 2024	SNPRC	YES			
14498	*Papio*	*hybrid*	United States of Ar	SNPRC		Kendall et al., 2024	SNPRC	YES			
14473	*Papio*	*hybrid*	United States of Ar	SNPRC		Kendall et al., 2024	SNPRC	YES			
14460	*Papio*	*hybrid*	United States of Ar	SNPRC		Kendall et al., 2024	SNPRC	YES			
14437	*Papio*	*hybrid*	United States of Ar	SNPRC		Kendall et al., 2024	SNPRC	YES			
14435	*Papio*	*hybrid*	United States of Ar	SNPRC		Kendall et al., 2024	SNPRC	YES			
14427	*Papio*	*hybrid*	United States of Ar	SNPRC		Kendall et al., 2024	SNPRC	YES			
14379	*Papio*	*hybrid*	United States of Ar	SNPRC		Kendall et al., 2024	SNPRC	YES			
14369	*Papio*	*hybrid*	United States of Ar	SNPRC		Kendall et al., 2024	SNPRC	YES			
14355	*Papio*	*hybrid*	United States of Ar	SNPRC		Kendall et al., 2024	SNPRC	YES			
14351	*Papio*	*hybrid*	United States of Ar	SNPRC		Kendall et al., 2024	SNPRC	YES			
14350	*Papio*	*hybrid*	United States of Ar	SNPRC		Kendall et al., 2024	SNPRC	YES			
14343	*Papio*	*hybrid*	United States of Ar	SNPRC		Kendall et al., 2024	SNPRC	YES			
14342	*Papio*	*hybrid*	United States of Ar	SNPRC		Kendall et al., 2024	SNPRC	YES			
14330	*Papio*	*hybrid*	United States of Ar	SNPRC		Kendall et al., 2024	SNPRC	YES			
14324	*Papio*	*hybrid*	United States of Ar	SNPRC		Kendall et al., 2024	SNPRC	YES			

Sample ID/ Acces	Genus	Species	Country	Locality	Mitochondrial Cla	Source	notes	Mitogenome newly assembled
14290	*Papio*	*hybrid*	United States of Ar	SNPRC		Kendall et al., 2024	SNPRC	YES
14282	*Papio*	*hybrid*	United States of Ar	SNPRC		Kendall et al., 2024	SNPRC	YES
14276	*Papio*	*hybrid*	United States of Ar	SNPRC		Kendall et al., 2024	SNPRC	YES
14273	*Papio*	*hybrid*	United States of Ar	SNPRC		Kendall et al., 2024	SNPRC	YES
14250	*Papio*	*hybrid*	United States of Ar	SNPRC		Kendall et al., 2024	SNPRC	YES
14204	*Papio*	*hybrid*	United States of Ar	SNPRC		Kendall et al., 2024	SNPRC	YES
14191	*Papio*	*hybrid*	United States of Ar	SNPRC		Kendall et al., 2024	SNPRC	YES
14182	*Papio*	*hybrid*	United States of Ar	SNPRC		Kendall et al., 2024	SNPRC	YES
14181	*Papio*	*hybrid*	United States of Ar	SNPRC		Kendall et al., 2024	SNPRC	YES
14172	*Papio*	*hybrid*	United States of Ar	SNPRC		Kendall et al., 2024	SNPRC	YES
14167	*Papio*	*hybrid*	United States of Ar	SNPRC		Kendall et al., 2024	SNPRC	YES
14158	*Papio*	*hybrid*	United States of Ar	SNPRC		Kendall et al., 2024	SNPRC	YES
14154	*Papio*	*hybrid*	United States of Ar	SNPRC		Kendall et al., 2024	SNPRC	YES
14077	*Papio*	*hybrid*	United States of Ar	SNPRC		Kendall et al., 2024	SNPRC	YES
14076	*Papio*	*hybrid*	United States of Ar	SNPRC		Kendall et al., 2024	SNPRC	YES
14068	*Papio*	*hybrid*	United States of Ar	SNPRC		Kendall et al., 2024	SNPRC	YES but deleted due to high missing/ambigous bases
14066	*Papio*	*hybrid*	United States of Ar	SNPRC		Kendall et al., 2024	SNPRC	YES but deleted due to high missing/ambigous bases
14062	*Papio*	*hybrid*	United States of Ar	SNPRC		Kendall et al., 2024	SNPRC	YES
14058	*Papio*	*hybrid*	United States of Ar	SNPRC		Kendall et al., 2024	SNPRC	YES
14022	*Papio*	*hybrid*	United States of Ar	SNPRC		Kendall et al., 2024	SNPRC	YES
14016	*Papio*	*hybrid*	United States of Ar	SNPRC		Kendall et al., 2024	SNPRC	YES
14013	*Papio*	*hybrid*	United States of Ar	SNPRC		Kendall et al., 2024	SNPRC	YES
14012	*Papio*	*hybrid*	United States of Ar	SNPRC		Kendall et al., 2024	SNPRC	YES
13988	*Papio*	*hybrid*	United States of Ar	SNPRC		Kendall et al., 2024	SNPRC	YES
13951	*Papio*	*hybrid*	United States of Ar	SNPRC		Kendall et al., 2024	SNPRC	YES
13942	*Papio*	*hybrid*	United States of Ar	SNPRC		Kendall et al., 2024	SNPRC	YES
13914	*Papio*	*hybrid*	United States of Ar	SNPRC		Kendall et al., 2024	SNPRC	YES
13698	*Papio*	*hybrid*	United States of Ar	SNPRC		Kendall et al., 2024	SNPRC	YES
13694	*Papio*	*hybrid*	United States of Ar	SNPRC		Kendall et al., 2024	SNPRC	YES
13644	*Papio*	*hybrid*	United States of Ar	SNPRC		Kendall et al., 2024	SNPRC	YES
13597	*Papio*	*hybrid*	United States of Ar	SNPRC		Kendall et al., 2024	SNPRC	YES
13589	*Papio*	*hybrid*	United States of Ar	SNPRC		Kendall et al., 2024	SNPRC	YES
13575	*Papio*	*hybrid*	United States of Ar	SNPRC		Kendall et al., 2024	SNPRC	YES
13570	*Papio*	*hybrid*	United States of Ar	SNPRC		Kendall et al., 2024	SNPRC	YES
13463	*Papio*	*hybrid*	United States of Ar	SNPRC		Kendall et al., 2024	SNPRC	YES
13387	*Papio*	*hybrid*	United States of Ar	SNPRC		Kendall et al., 2024	SNPRC	YES
13264	*Papio*	*hybrid*	United States of Ar	SNPRC		Kendall et al., 2024	SNPRC	YES
13245	*Papio*	*hybrid*	United States of Ar	SNPRC		Kendall et al., 2024	SNPRC	YES
13228	*Papio*	*hybrid*	United States of Ar	SNPRC		Kendall et al., 2024	SNPRC	YES
13226	*Papio*	*hybrid*	United States of Ar	SNPRC		Kendall et al., 2024	SNPRC	YES
13225	*Papio*	*hybrid*	United States of Ar	SNPRC		Kendall et al., 2024	SNPRC	YES
13169	*Papio*	*hybrid*	United States of Ar	SNPRC		Kendall et al., 2024	SNPRC	YES
13152	*Papio*	*hybrid*	United States of Ar	SNPRC		Kendall et al., 2024	SNPRC	YES
13110	*Papio*	*hybrid*	United States of Ar	SNPRC		Kendall et al., 2024	SNPRC	YES
13103	*Papio*	*hybrid*	United States of Ar	SNPRC		Kendall et al., 2024	SNPRC	YES
13068	*Papio*	*hybrid*	United States of Ar	SNPRC		Kendall et al., 2024	SNPRC	YES
12963	*Papio*	*hybrid*	United States of Ar	SNPRC		Kendall et al., 2024	SNPRC	YES
12962	*Papio*	*hybrid*	United States of Ar	SNPRC		Kendall et al., 2024	SNPRC	YES
12869	*Papio*	*hybrid*	United States of Ar	SNPRC		Kendall et al., 2024	SNPRC	YES
12720	*Papio*	*hybrid*	United States of Ar	SNPRC		Kendall et al., 2024	SNPRC	YES
12719	*Papio*	*hybrid*	United States of Ar	SNPRC		Kendall et al., 2024	SNPRC	YES
12717	*Papio*	*hybrid*	United States of Ar	SNPRC		Kendall et al., 2024	SNPRC	YES
12681	*Papio*	*hybrid*	United States of Ar	SNPRC		Kendall et al., 2024	SNPRC	YES
12656	*Papio*	*hybrid*	United States of Ar	SNPRC		Kendall et al., 2024	SNPRC	YES but deleted due to high missing/ambigous bases
12552	*Papio*	*hybrid*	United States of Ar	SNPRC		Kendall et al., 2024	SNPRC	YES
12491	*Papio*	*hybrid*	United States of Ar	SNPRC		Kendall et al., 2024	SNPRC	YES
12481	*Papio*	*hybrid*	United States of Ar	SNPRC		Kendall et al., 2024	SNPRC	YES
12473	*Papio*	*hybrid*	United States of Ar	SNPRC		Kendall et al., 2024	SNPRC	YES
12457	*Papio*	*hybrid*	United States of Ar	SNPRC		Kendall et al., 2024	SNPRC	YES
12427	*Papio*	*hybrid*	United States of Ar	SNPRC		Kendall et al., 2024	SNPRC	YES
12423	*Papio*	*hybrid*	United States of Ar	SNPRC		Kendall et al., 2024	SNPRC	YES
12282	*Papio*	*hybrid*	United States of Ar	SNPRC		Kendall et al., 2024	SNPRC	YES
12242	*Papio*	*hybrid*	United States of Ar	SNPRC		Kendall et al., 2024	SNPRC	YES
12226	*Papio*	*hybrid*	United States of Ar	SNPRC		Kendall et al., 2024	SNPRC	YES
12218	*Papio*	*hybrid*	United States of Ar	SNPRC		Kendall et al., 2024	SNPRC	YES
12175	*Papio*	*hybrid*	United States of Ar	SNPRC		Kendall et al., 2024	SNPRC	YES
12156	*Papio*	*hybrid*	United States of Ar	SNPRC		Kendall et al., 2024	SNPRC	YES
12152	*Papio*	*hybrid*	United States of Ar	SNPRC		Kendall et al., 2024	SNPRC	YES
12141	*Papio*	*hybrid*	United States of Ar	SNPRC		Kendall et al., 2024	SNPRC	YES
12138	*Papio*	*hybrid*	United States of Ar	SNPRC		Kendall et al., 2024	SNPRC	YES
11981	*Papio*	*hybrid*	United States of Ar	SNPRC		Kendall et al., 2024	SNPRC	YES
11967	*Papio*	*hybrid*	United States of Ar	SNPRC		Kendall et al., 2024	SNPRC	YES
11887	*Papio*	*hybrid*	United States of Ar	SNPRC		Kendall et al., 2024	SNPRC	YES
11885	*Papio*	*hybrid*	United States of Ar	SNPRC		Kendall et al., 2024	SNPRC	YES
11770	*Papio*	*hybrid*	United States of Ar	SNPRC		Kendall et al., 2024	SNPRC	YES
11769	*Papio*	*hybrid*	United States of Ar	SNPRC		Kendall et al., 2024	SNPRC	YES
11752	*Papio*	*hybrid*	United States of Ar	SNPRC		Kendall et al., 2024	SNPRC	YES
11693	*Papio*	*hybrid*	United States of Ar	SNPRC		Kendall et al., 2024	SNPRC	YES
11608	*Papio*	*hybrid*	United States of Ar	SNPRC		Kendall et al., 2024	SNPRC	YES
11562	*Papio*	*hybrid*	United States of Ar	SNPRC		Kendall et al., 2024	SNPRC	YES
11158	*Papio*	*hybrid*	United States of Ar	SNPRC		Kendall et al., 2024	SNPRC	YES
11004	*Papio*	*hybrid*	United States of Ar	SNPRC		Kendall et al., 2024	SNPRC	YES
10998	*Papio*	*hybrid*	United States of Ar	SNPRC		Kendall et al., 2024	SNPRC	YES
10987	*Papio*	*hybrid*	United States of Ar	SNPRC		Kendall et al., 2024	SNPRC	YES

Sample ID/ Acces	Genus	Species	Country	Locality	Mitochondrial Cla	Source	notes	Mitogenome newly assembled			
10842	*Papio*	*hybrid*	United States of Ar	SNPRC		Kendall et al., 2024	SNPRC	YES			
10489	*Papio*	*hybrid*	United States of Ar	SNPRC		Kendall et al., 2024	SNPRC	YES			
10488	*Papio*	*hybrid*	United States of Ar	SNPRC		Kendall et al., 2024	SNPRC	YES			
10482	*Papio*	*hybrid*	United States of Ar	SNPRC		Kendall et al., 2024	SNPRC	YES			
10418	*Papio*	*hybrid*	United States of Ar	SNPRC		Kendall et al., 2024	SNPRC	YES			
10349	*Papio*	*hybrid*	United States of Ar	SNPRC		Kendall et al., 2024	SNPRC	YES			
10316	*Papio*	*hybrid*	United States of Ar	SNPRC		Kendall et al., 2024	SNPRC	YES			
10192	*Papio*	*hybrid*	United States of Ar	SNPRC		Kendall et al., 2024	SNPRC	YES			
10173	*Papio*	*hybrid*	United States of Ar	SNPRC		Kendall et al., 2024	SNPRC	YES			
10046	*Papio*	*hybrid*	United States of Ar	SNPRC		Kendall et al., 2024	SNPRC	YES			
9878	*Papio*	*hybrid*	United States of Ar	SNPRC		Kendall et al., 2024	SNPRC	YES			
9860	*Papio*	*hybrid*	United States of Ar	SNPRC		Kendall et al., 2024	SNPRC	YES			
9841	*Papio*	*hybrid*	United States of Ar	SNPRC		Kendall et al., 2024	SNPRC	YES			
9656	*Papio*	*hybrid*	United States of Ar	SNPRC		Kendall et al., 2024	SNPRC	YES			
9562	*Papio*	*hybrid*	United States of Ar	SNPRC		Kendall et al., 2024	SNPRC	YES			
9481	*Papio*	*hybrid*	United States of Ar	SNPRC		Kendall et al., 2024	SNPRC	YES			
9128	*Papio*	*hybrid*	United States of Ar	SNPRC		Kendall et al., 2024	SNPRC	YES			
9086	*Papio*	*hybrid*	United States of Ar	SNPRC		Kendall et al., 2024	SNPRC	YES			
9045	*Papio*	*hybrid*	United States of Ar	SNPRC		Kendall et al., 2024	SNPRC	YES			
8995	*Papio*	*hybrid*	United States of Ar	SNPRC		Kendall et al., 2024	SNPRC	YES			
8780	*Papio*	*hybrid*	United States of Ar	SNPRC		Kendall et al., 2024	SNPRC	YES			
8653	*Papio*	*hybrid*	United States of Ar	SNPRC		Kendall et al., 2024	SNPRC	YES			
8596	*Papio*	*hybrid*	United States of Ar	SNPRC		Kendall et al., 2024	SNPRC	YES			
8581	*Papio*	*hybrid*	United States of Ar	SNPRC		Kendall et al., 2024	SNPRC	YES			
8465	*Papio*	*hybrid*	United States of Ar	SNPRC		Kendall et al., 2024	SNPRC	YES			
8395	*Papio*	*hybrid*	United States of Ar	SNPRC		Kendall et al., 2024	SNPRC	YES			
8344	*Papio*	*hybrid*	United States of Ar	SNPRC		Kendall et al., 2024	SNPRC	YES			
8307	*Papio*	*hybrid*	United States of Ar	SNPRC		Kendall et al., 2024	SNPRC	YES			
8170	*Papio*	*hybrid*	United States of Ar	SNPRC		Kendall et al., 2024	SNPRC	YES			
7937	*Papio*	*hybrid*	United States of Ar	SNPRC		Kendall et al., 2024	SNPRC	YES			
7625	*Papio*	*hybrid*	United States of Ar	SNPRC		Kendall et al., 2024	SNPRC	YES			
7478	*Papio*	*hybrid*	United States of Ar	SNPRC		Kendall et al., 2024	SNPRC	YES			
7311	*Papio*	*hybrid*	United States of Ar	SNPRC		Kendall et al., 2024	SNPRC	YES			
7267	*Papio*	*hybrid*	United States of Ar	SNPRC		Kendall et al., 2024	SNPRC	YES			
7158	*Papio*	*hybrid*	United States of Ar	SNPRC		Kendall et al., 2024	SNPRC	YES			
7091	*Papio*	*hybrid*	United States of Ar	SNPRC		Kendall et al., 2024	SNPRC	YES			
6955	*Papio*	*hybrid*	United States of Ar	SNPRC		Kendall et al., 2024	SNPRC	YES			
6716	*Papio*	*hybrid*	United States of Ar	SNPRC		Kendall et al., 2024	SNPRC	YES			
6382	*Papio*	*hybrid*	United States of Ar	SNPRC		Kendall et al., 2024	SNPRC	YES			
6265	*Papio*	*hybrid*	United States of Ar	SNPRC		Kendall et al., 2024	SNPRC	YES			

ID	5 million reads	10 million reads	15 million reads	20 million reads	25 million reads	Whole Genome
1X0102	396.59	790.59	1,186.35	1,589.68	1,979.47	32,524.50
1X0110	388.15	779.08	1,167.29	1,551.20	1,938.91	40,191.99
1X1734	27.96	57.06	85.88	115.94	144.17	2,537.24
1X2049	35.37	71.13	106.56	143.28	180.27	3,345.08
1X2208	30.53	63.33	94.45	125.98	159.07	3,270.47
1X2304	487.01	972.87	1,464.78	1,954.13	2,440.59	45,041.67
1X0026	456.81	925.14	1,395.64	1,864.36	2,337.73	42,908.87
1X0576	846.11	1,705.84	2,567.74	3,431.47	4,290.46	63,809.44
1X0580	44.18	88.56	131.77	175.57	219.22	4,313.70
1X0812	76.82	153.43	229.92	306.50	380.73	7,901.34
1X1032	39.83	79.77	119.86	160.81	200.51	3,551.29
1X1146	32.44	66.60	101.98	135.77	169.96	3,195.59
1X1181	44.33	87.22	129.73	173.36	218.86	4,454.23
1X2891	31.42	64.09	95.78	128.62	161.34	2,102.64
1X3162	27.17	54.78	81.54	108.87	135.98	2,608.17
1X4384	47.93	96.27	145.26	191.00	239.46	4,373.88
1X3548	42.59	86.10	128.53	172.98	218.66	4,357.44
1X3576	498.42	1,002.65	1,505.56	2,011.58	2,519.58	46,094.02
1X1979	417.97	854.47	1,291.89	1,727.85	2,163.61	37,847.34
1X0014	36.83	75.00	114.12	152.55	190.46	3,659.26
1X0035	451.10	910.30	1,377.82	1,847.81	2,311.86	31,796.97
1X0153	40.03	80.66	122.91	163.13	205.05	3,013.53
1X0808	27.61	55.22	83.26	111.43	139.59	2,291.40
1X0832	287.39	579.33	876.15	1,174.93	1,466.27	29,120.72
1X0951	363.85	734.72	1,104.41	1,475.10	1,843.68	31,191.44
1X1125	36.71	76.97	116.20	156.59	196.01	3,752.15
1X1126	32.64	65.82	98.89	131.59	165.49	3,287.55
1X1152	22.34	44.98	68.03	90.48	112.70	2,166.89
1X1672	423.58	852.00	1,274.10	1,701.53	2,139.68	40,133.00
1X1700	463.45	926.82	1,397.71	1,864.84	2,332.01	46,139.14
1X1765	26.44	50.43	77.25	101.54	128.91	2,469.80
1X1939	479.45	965.50	1,455.15	1,942.07	2,426.84	48,903.02
1X1947	410.56	820.35	1,221.25	1,613.79	2,015.26	35,678.27

Sample	Genus	Species	Country	Locality	Source	Depth	Dataset	Set A	Set B	Note
PA_0208	*Papio*	*anubis*	Tanzania	Ngorongoro	Sørensen et al., 2023	35.7	Set B		Reference	
PA_0209	*Papio*	*anubis*	Tanzania	Ngorongoro	Sørensen et al., 2023	34.5	Set B		Reference	
PA_0210	*Papio*	*anubis*	Tanzania	Ngorongoro	Sørensen et al., 2023	33.6	Set B		Reference	
PA_0211	*Papio*	*anubis*	Tanzania	Ngorongoro	Sørensen et al., 2023	33.1	Set B; Set A	Reference	Reference	
PA_0212	*Papio*	*anubis*	Tanzania	Ngorongoro	Sørensen et al., 2023	34	Set B		Reference	
PA_0238	*Papio*	*anubis*	Tanzania	Lake Manyara	Sørensen et al., 2023	31.4	Set B; Set A	Reference	Reference	
PA_0240	*Papio*	*anubis*	Tanzania	Lake Manyara	Sørensen et al., 2023	31.3	Set B		Reference	
PA_0242	*Papio*	*anubis*	Tanzania	Lake Manyara	Sørensen et al., 2023	29.8	Set B		Reference	
PA_0675	*Papio*	*anubis*	Tanzania	Lake Manyara	Sørensen et al., 2023	28.6	Set B		Reference	
PA_0682	*Papio*	*anubis*	Tanzania	Lake Manyara	Sørensen et al., 2023	30.4	Set B		Reference	
PA_0720	*Papio*	*anubis*	Ethiopia	Gog	Sørensen et al., 2023	35.7	Set A	Reference	Reference	
PA_0727	*Papio*	*anubis*	Ethiopia	Gog	Sørensen et al., 2023	35.2	Set A	Reference	Reference	
PC_0745	*Papio*	*cynocephalus*	Tanzania	Mikumi National	Sørensen et al., 2023	30.8	Set B		Reference	
PC_0746	*Papio*	*cynocephalus*	Tanzania	Mikumi National	Sørensen et al., 2023	54	Set B; Set A	Reference	Reference	
PC_0748	*Papio*	*cynocephalus*	Tanzania	Mikumi National	Sørensen et al., 2023	29.5	Set B		Reference	
PC_0755	*Papio*	*cynocephalus*	Tanzania	Mikumi National	Sørensen et al., 2023	26.3	Set B; Set A	Reference	Reference	
PC_0756	*Papio*	*cynocephalus*	Tanzania	Mikumi National	Sørensen et al., 2023	35.1	Set B		Reference	
PC_0761	*Papio*	*cynocephalus*	Tanzania	Mikumi National	Sørensen et al., 2023	35.9	Set B		Reference	
PC_0763	*Papio*	*cynocephalus*	Tanzania	Mikumi National	Sørensen et al., 2023	34.4	Set B		Reference	
PC_0765	*Papio*	*cynocephalus*	Tanzania	Mikumi National	Sørensen et al., 2023	27.9	Set B; Set A	Reference	Reference	
PC_0768	*Papio*	*cynocephalus*	Tanzania	Mikumi National	Sørensen et al., 2023	28.2	Set B		Reference	
PC_0770	*Papio*	*cynocephalus*	Tanzania	Mikumi National	Sørensen et al., 2023	38.1	Set B; Set A	Reference	Reference	
PC_HAP	*Papio*	*cynocephalus*	Kenya	Amboseli Nationa	Wall et al., 2016	19.6	Set B; Set A	Reference	Reference	
PH_0697	*Papio*	*hamadryas*	Ethiopia	Filoha	Sørensen et al., 2023	39.3	Set B; Set A	Reference/PANE Source	Reference	
PH_0699	*Papio*	*hamadryas*	Ethiopia	Filoha	Sørensen et al., 2023	36.7	Set B		Reference	
PH_0700	*Papio*	*hamadryas*	Ethiopia	Filoha	Sørensen et al., 2023	36.5	Set B		Reference	
PH_0703	*Papio*	*hamadryas*	Ethiopia	Filoha	Sørensen et al., 2023	32.9	Set B		Reference	
PH_0705	*Papio*	*hamadryas*	Ethiopia	Filoha	Sørensen et al., 2023	33.9	Set B; Set A	Reference/PANE Source	Reference	
PH_0709	*Papio*	*hamadryas*	Ethiopia	Filoha	Sørensen et al., 2023	62.1	Set B		Reference	
PH_0711	*Papio*	*hamadryas*	Ethiopia	Filoha	Sørensen et al., 2023	24.5	Set B		Reference	
PH_0715	*Papio*	*hamadryas*	Ethiopia	Filoha	Sørensen et al., 2023	43.5	Set B		Reference	
PH_SAMN20949	*Papio*	*hamadryas*	Ethiopia	Filoha	Chiou et al., 2022	14.6	Set B; Set A	Reference/PANE Source	Reference	
PH_SAMN20949	*Papio*	*hamadryas*	Ethiopia	Filoha	Chiou et al., 2022	11.8	Set B; Set A	Reference/PANE Source	Reference	
PK_0749	*Papio*	*kindae*	Zambia	Chunga	Sørensen et al., 2023	44.9	Set A	Reference/PANE Source		
PK_0751	*Papio*	*kindae*	Zambia	Chunga	Sørensen et al., 2023	41.5	Set A	Reference/PANE Source		
PK_0758	*Papio*	*kindae*	Zambia	Chunga	Sørensen et al., 2023	44.4	Set A	Reference/PANE Source		
PK_0760	*Papio*	*kindae*	Zambia	Chunga	Sørensen et al., 2023	37.6	Set A	Reference/PANE Source		
PP_0394	*Papio*	*papio*	Senegal	Niokolo-Koba	Sørensen et al., 2023	31.9	Set A	Reference/PANE Source		
PP_0398	*Papio*	*papio*	Senegal	Niokolo-Koba	Sørensen et al., 2023	31.4	Set A	Reference/PANE Source		
PP_0400	*Papio*	*papio*	Senegal	Niokolo-Koba	Sørensen et al., 2023	37.6	Set A	Reference/PANE Source		
PP_0401	*Papio*	*papio*	Senegal	Niokolo-Koba	Sørensen et al., 2023	32.6	Set A	Reference/PANE Source		
PU_0692	*Papio*	*ursinus*	Zambia	Dendro Park	Sørensen et al., 2023	33	Set A	Reference/PANE Source		
PU_0693	*Papio*	*ursinus*	Zambia	Dendro Park	Sørensen et al., 2023	33	Set A	Reference/PANE Source		
PU_0694	*Papio*	*ursinus*	Zambia	Dendro Park	Sørensen et al., 2023	33.2	Set A	Reference/PANE Source		
PU_0695	*Papio*	*ursinus*	Zambia	Dendro Park	Sørensen et al., 2023	34.2	Set A	Reference/PANE Source		
1X0351	*Papio*	*anubis*	United States of	SNPRC	Kendall et al., 2024	35.3	Set A	Target		
1X0356	*Papio*	*hybrid*	United States of	SNPRC	Kendall et al., 2024	53	Set B; Set A	Target	Target	Reported as FOUNDER by Kendall et al., (2024)
1X0812	*Papio*	*anubis*	United States of	SNPRC	Kendall et al., 2024	36.67	Set B; Set A	Founder/Reference	Target	
1X1126	*Papio*	*anubis*	United States of	SNPRC	Kendall et al., 2024	60	Set A	Founder/Reference/PANE Source		
1X1152	*Papio*	*anubis*	United States of	SNPRC	Kendall et al., 2024	35	Set A	Founder/Reference/PANE Source		
1X1734	*Papio*	*cynocephalus*	United States of	SNPRC	Kendall et al., 2024	32.5	Set A	Founder/Reference/PANE Source		
1X1947	*Papio*	*anubis*	United States of	SNPRC	Kendall et al., 2024	35.4	Set A	Founder/Reference/PANE Source		
1X1958	*Papio*	*hybrid*	United States of	SNPRC	Kendall et al., 2024	30.4	Set B		Target	Reported as FOUNDER by Kendall et al., (2024)
1X2049	*Papio*	*cynocephalus*	United States of	SNPRC	Kendall et al., 2024	35.3	Set A	Founder/Reference/PANE Source		
1X2124	*Papio*	*hybrid*	United States of	SNPRC	Kendall et al., 2024	54.9	Set B; Set A	Target	Target	
1X2304	*Papio*	*cynocephalus*	United States of	SNPRC	Kendall et al., 2024	31.7	Set A	Founder/Reference/PANE Source		
1X3321	*Papio*	*hybrid*	United States of	SNPRC	Kendall et al., 2024	36.1	Set A	Target		
1X3548	*Papio*	*cynocephalus*	United States of	SNPRC	Kendall et al., 2024	36.9	Set A	Founder/Reference/PANE Source		
1X3796	*Papio*	*hybrid*	United States of	SNPRC	Kendall et al., 2024	26.8	Set B		Target	
1X3837	*Papio*	*hybrid*	United States of	SNPRC	Kendall et al., 2024	35.1	Set B		Target	
1X4209	*Papio*	*hybrid*	United States of	SNPRC	Kendall et al., 2024	32.9	Set B		Target	
1x0576	*Papio*	*anubis*	United States of	SNPRC	Kendall et al., 2024	34.7	Set A	Founder/Reference/PANE Source		
10488	*Papio*	*hybrid*	United States of	SNPRC	Kendall et al., 2024	33.48	Set B		Target	
12869	*Papio*	*hybrid*	United States of	SNPRC	Kendall et al., 2024	6	Set A	Target		
12962	*Papio*	*hybrid*	United States of	SNPRC	Kendall et al., 2024	6.5	Set A	Target		
13264	*Papio*	*hybrid*	United States of	SNPRC	Kendall et al., 2024	5.3	Set A	Target		
13589	*Papio*	*hybrid*	United States of	SNPRC	Kendall et al., 2024	3.6	Set A	Target		
13597	*Papio*	*hybrid*	United States of	SNPRC	Kendall et al., 2024	5.41	Set A	Target		
13694	*Papio*	*hybrid*	United States of	SNPRC	Kendall et al., 2024	5.7	Set A	Target		
14182	*Papio*	*hybrid*	United States of	SNPRC	Kendall et al., 2024	30.1	Set B		Target	
14867	*Papio*	*hybrid*	United States of	SNPRC	Kendall et al., 2024	4.5	Set A	Target		
14951	*Papio*	*hybrid*	United States of	SNPRC	Kendall et al., 2024	11	Set A	Target		
14994	*Papio*	*hybrid*	United States of	SNPRC	Kendall et al., 2024	2.5	Set A	Target		
15581	*Papio*	*hybrid*	United States of	SNPRC	Kendall et al., 2024	5.7	Set A	Target		
16246	*Papio*	*hybrid*	United States of	SNPRC	Kendall et al., 2024	4.6	Set A	Target		
18019	*Papio*	*hybrid*	United States of	SNPRC	Kendall et al., 2024	7.9	Set A	Target		
18778	*Papio*	*hybrid*	United States of	SNPRC	Kendall et al., 2024	6.1	Set A	Target		
28003	*Papio*	*hybrid*	United States of	SNPRC	Kendall et al., 2024	5.3	Set A	Target		
28368	*Papio*	*hybrid*	United States of	SNPRC	Kendall et al., 2024	5.3	Set A	Target		
30008	*Papio*	*hybrid*	United States of	SNPRC	Kendall et al., 2024	4.5	Set A	Target		
31015	*Papio*	*hybrid*	United States of	SNPRC	Kendall et al., 2024	5.9	Set A	Target		
31025	*Papio*	*hybrid*	United States of	SNPRC	Kendall et al., 2024	4.1	Set A	Target		
31115	*Papio*	*hybrid*	United States of	SNPRC	Kendall et al., 2024	4.2	Set A	Target		
31602	*Papio*	*hybrid*	United States of	SNPRC	Kendall et al., 2024	4.7	Set A	Target		
32606	*Papio*	*hybrid*	United States of	SNPRC	Kendall et al., 2024	4.7	Set A	Target		
8995	*Papio*	*hybrid*	United States of	SNPRC	Kendall et al., 2024	4.9	Set A	Target		
9481	*Papio*	*hybrid*	United States of	SNPRC	Kendall et al., 2024	5.9	Set A	Target		
9860	*Papio*	*hybrid*	United States of	SNPRC	Kendall et al., 2024	33.6	Set B	Target	Target	

TableS5_AdmixtureResults

ID	Pop	K=2 Southern *Papio*	K=2 Northern *Papio*	K=3 *P. hamadryas/P. pa*	K=3 *P. anubis*	K=3 Southern *Papio*	K=4 *P. hamadryas*	K=4 *P. anubis*	K=4 Southern *Papio*	K=4 *P. papio*
PD_0720	*P. anubis*	0.00001	0.99999	0.138653	0.861337	0.00001	0.115535	0.868711	0.00001	0.015744
PD_0727	*P. anubis*	0.00001	0.99999	0.1326	0.86739	0.00001	0.110615	0.873868	0.00001	0.015507
PD_0746	*P. cynocephalus* (Tanzania)	0.99999	0.00001	0.00001	0.00001	0.99998	0.00001	0.00001	0.99997	0.00001
PD_0770	*P. cynocephalus* (Tanzania)	0.99999	0.00001	0.00001	0.00001	0.99998	0.00001	0.00001	0.99997	0.00001
HAP	*P. cynocephalus* (Kenya)	0.911169	0.088831	0.00001	0.098411	0.901579	0.00001	0.099372	0.900608	0.00001
SAMN20949827	*P. hamadryas*	0.225446	0.774554	0.999979	0.000011	0.00001	0.99997	0.00001	0.00001	0.00001
SAMN20949837	*P. hamadryas*	0.224074	0.775926	0.99998	0.00001	0.00001	0.99997	0.00001	0.00001	0.00001
PD_0749	*P. kindae*	0.816662	0.183338	0.271957	0.00001	0.728033	0.288763	0.00001	0.689315	0.021911
PD_0751	*P. kindae*	0.815314	0.184686	0.273184	0.00001	0.726806	0.292168	0.00001	0.687982	0.01984
PD_0400	*P. papio*	0.091285	0.908715	0.99998	0.00001	0.00001	0.00001	0.00001	0.00001	0.99997
PD_0401	*P. papio*	0.091331	0.908669	0.99998	0.00001	0.00001	0.00001	0.00001	0.00001	0.99997
PD_0892	*P. ursinus*	0.983105	0.016895	0.139266	0.00001	0.860724	0.17238	0.00001	0.8276	0.00001
PD_0893	*P. ursinus*	0.978825	0.021175	0.140192	0.00001	0.859798	0.173421	0.00001	0.826559	0.00001
1X0576	SNPRC Founder (*P. anubis*)	0.00001	0.99999	0.040346	0.959644	0.00001	0.010832	0.970595	0.00001	0.018563
1X0580	SNPRC Founder (*P. anubis*)	0.00001	0.99999	0.039798	0.960192	0.00001	0.006826	0.972082	0.00001	0.021082
1X0153	SNPRC Founder (*P. anubis*)	0.00001	0.99999	0.00001	0.99998	0.00001	0.00001	0.99997	0.00001	0.00001
1X1947	SNPRC Founder (*P. anubis*)	0.00001	0.99999	0.00001	0.99998	0.00001	0.00001	0.99997	0.00001	0.00001
1X1152	SNPRC Founder (*P. anubis*)	0.00001	0.99999	0.00001	0.99998	0.00001	0.00001	0.99997	0.00001	0.00001
1X0102	SNPRC Founder (*P. cynocepha*	0.926625	0.073375	0.00001	0.08282	0.91717	0.00001	0.083723	0.916257	0.00001
1X1734	SNPRC Founder (*P. cynocepha*	0.976573	0.023427	0.00001	0.038235	0.961755	0.00001	0.038872	0.961108	0.00001
1X2049	SNPRC Founder (*P. cynocepha*	0.934806	0.065194	0.00001	0.074705	0.925285	0.00001	0.075527	0.924453	0.00001
1X2208	SNPRC Founder (*P. cynocepha*	0.97118	0.02882	0.00001	0.042606	0.957384	0.00001	0.043275	0.956705	0.00001
1X2304	SNPRC Founder (*P. cynocepha*	0.99999	0.00001	0.00001	0.00001	0.99998	0.00001	0.00001	0.99997	0.00001
1X4384	SNPRC Founder (*P. anubis*)	0.99999	0.00001	0.00001	0.00001	0.99998	0.00001	0.00001	0.99997	0.00001
1Y3548	SNPRC Founder (*P. cynocepha*	0.99999	0.00001	0.00001	0.00001	0.99998	0.00001	0.00001	0.99997	0.00001
1X0014	SNPRC Founder (*P. anubis*)	0.00001	0.99999	0.057095	0.942895	0.00001	0.049275	0.948793	0.00001	0.001922
1X0026	SNPRC Founder (*P. anubis*)	0.00001	0.99999	0.000097	0.999893	0.00001	0.00001	0.999295	0.00001	0.000685
1X0035	SNPRC Founder (*P. anubis*)	0.00001	0.99999	0.063926	0.936064	0.00001	0.05203	0.940823	0.00001	0.007137
1X0110	SNPRC Founder (*P. cynocepha*	0.907726	0.092274	0.00001	0.101539	0.898451	0.00001	0.102584	0.897396	0.00001
1X0606	SNPRC Founder (*P. anubis*)	0.00001	0.99999	0.00001	0.99998	0.00001	0.00001	0.99997	0.00001	0.00001
1X0612	SNPRC Founder (*P. anubis*)	0.00001	0.99999	0.225319	0.774671	0.00001	0.203341	0.784765	0.00001	0.011884
1X0632	SNPRC Founder (*P. anubis*)	0.00001	0.99999	0.00001	0.99998	0.00001	0.00001	0.99997	0.00001	0.00001
1X0951	SNPRC Founder (*P. anubis*)	0.00001	0.99999	0.00001	0.99998	0.00001	0.00001	0.99997	0.00001	0.00001
1X1032	SNPRC Founder (*P. anubis*)	0.00001	0.99999	0.00001	0.99998	0.00001	0.00001	0.99997	0.00001	0.00001
1X1125	SNPRC Founder (*P. anubis*)	0.00001	0.99999	0.00001	0.99998	0.00001	0.00001	0.99997	0.00001	0.00001
1X1126	SNPRC Founder (*P. anubis*)	0.00001	0.99999	0.00001	0.99998	0.00001	0.00001	0.99997	0.00001	0.00001
1X1146	SNPRC Founder (*P. anubis*)	0.00001	0.99999	0.00001	0.99998	0.00001	0.00001	0.99997	0.00001	0.00001
1X1181	SNPRC Founder (*P. anubis*)	0.00001	0.99999	0.00001	0.99998	0.00001	0.00001	0.99997	0.00001	0.00001
1X1672	SNPRC Founder (*P. anubis*)	0.00001	0.99999	0.056386	0.943604	0.00001	0.042628	0.94901	0.00001	0.008352
1X1700	SNPRC Founder (*P. anubis*)	0.00001	0.99999	0.00001	0.99998	0.00001	0.00001	0.99997	0.00001	0.00001
1X1765	SNPRC Founder (*P. anubis*)	0.00001	0.99999	0.00001	0.99998	0.00001	0.00001	0.99997	0.00001	0.00001
1X1939	SNPRC Founder (*P. anubis*)	0.00001	0.99999	0.00001	0.99998	0.00001	0.00001	0.99997	0.00001	0.00001
1X2891	SNPRC Founder (*P. anubis*)	0.00001	0.99999	0.00001	0.99998	0.00001	0.00001	0.99997	0.00001	0.00001
1X3162	SNPRC Founder (*P. anubis*)	0.00001	0.99999	0.00001	0.99998	0.00001	0.00001	0.99997	0.00001	0.00001
1X3576	SNPRC Founder (*P. cynocepha*	0.00001	0.99999	0.00001	0.99998	0.00001	0.00001	0.99997	0.00001	0.00001
1X1979	SNPRC Founder (*P. anubis*)	0.00001	0.99999	0.048171	0.951819	0.00001	0.034788	0.957861	0.00001	0.007341

ID	K=5 *P. kindae/P. ursin*	K=5 *P. cynocephalus*	K=5 *P. anubis*	K=5 *P. papio*	K=5 *P. hamadryas*	K=6 SNPRC *P. anubis*	K=6 *P. hamadryas/P. pa*	K=6 *P. kindae/P. ursin*	K=6 *P. anubis*	K=6 SNPRC *P. anubis*	K=6 *P. cynocephalus*
PD_0720	0.00001	0.00001	0.870475	0.017466	0.11204	0.00001	0.00001	0.00001	0.99995	0.00001	0.00001
PD_0727	0.00001	0.00001	0.87485	0.017165	0.107964	0.00001	0.00001	0.00001	0.99995	0.00001	0.00001
PD_0746	0.179983	0.819987	0.00001	0.00001	0.00001	0.00001	0.00001	0.179973	0.00001	0.00001	0.819987
PD_0770	0.150176	0.849794	0.00001	0.00001	0.00001	0.00001	0.000011	0.150148	0.00001	0.00001	0.849811
HAP	0.00001	0.910393	0.089577	0.00001	0.00001	0.00001	0.00001	0.00001	0.00001	0.090163	0.909797
SAMN20949827	0.00001	0.00001	0.00001	0.00001	0.99996	0.00001	0.99995	0.00001	0.00001	0.00001	0.00001
SAMN20949837	0.00001	0.00001	0.00001	0.00001	0.99996	0.00001	0.99995	0.00001	0.00001	0.00001	0.00001
PD_0749	0.999959	0.000011	0.00001	0.00001	0.00001	0.00001	0.00001	0.99995	0.00001	0.00001	0.00001
PD_0751	0.99996	0.00001	0.00001	0.00001	0.00001	0.00001	0.00001	0.99995	0.00001	0.00001	0.00001
PD_0400	0.00001	0.00001	0.00001	0.99996	0.00001	0.00001	0.99995	0.00001	0.00001	0.00001	0.00001
PD_0401	0.00001	0.00001	0.000011	0.999959	0.00001	0.00001	0.999945	0.00001	0.000015	0.00001	0.00001
PD_0892	0.99996	0.00001	0.00001	0.00001	0.00001	0.00001	0.00001	0.99995	0.00001	0.00001	0.00001
PD_0893	0.99996	0.00001	0.00001	0.00001	0.00001	0.00001	0.00001	0.99995	0.00001	0.00001	0.00001
1X0576	0.00001	0.00001	0.972647	0.018695	0.008638	0.587144	0.00001	0.00001	0.412816	0.00001	0.00001
1X0580	0.00001	0.00001	0.973604	0.021043	0.005333	0.590779	0.00001	0.00001	0.409181	0.00001	0.00001
1X0153	0.00001	0.00001	0.99996	0.00001	0.00001	0.99995	0.00001	0.00001	0.00001	0.00001	0.00001
1X1947	0.00001	0.00001	0.99996	0.00001	0.00001	0.99995	0.00001	0.00001	0.00001	0.00001	0.00001
1X1152	0.00001	0.00001	0.99996	0.00001	0.00001	0.00001	0.00001	0.00001	0.00001	0.99995	0.00001
1X0102	0.00001	0.930448	0.069522	0.00001	0.00001	0.071136	0.00001	0.00001	0.00001	0.00001	0.928824
1X1734	0.00001	0.997177	0.002793	0.00001	0.00001	0.00001	0.00001	0.00001	0.00001	0.007289	0.992671
1X2049	0.00001	0.94071	0.05926	0.00001	0.00001	0.062297	0.00001	0.00001	0.00001	0.00001	0.937663
1X2208	0.00001	0.991206	0.008764	0.00001	0.00001	0.00001	0.00001	0.00001	0.00001	0.013635	0.986325
1X2304	0.00001	0.99996	0.00001	0.00001	0.00001	0.00001	0.00001	0.00001	0.00001	0.00001	0.99995
1X4384	0.00001	0.99996	0.00001	0.00001	0.00001	0.00001	0.00001	0.00001	0.00001	0.00001	0.99995
1Y3548	0.000013	0.999957	0.00001	0.00001	0.00001	0.00001	0.00001	0.00001	0.00001	0.00001	0.99995
1X0014	0.00001	0.00001	0.953582	0.003034	0.043363	0.637584	0.00001	0.00001	0.362376	0.00001	0.00001
1X0026	0.00001	0.00001	0.999556	0.000414	0.00001	0.859431	0.00001	0.00001	0.140529	0.00001	0.00001
1X0035	0.00001	0.00001	0.946016	0.008294	0.04567	0.63187	0.00001	0.00001	0.36809	0.00001	0.00001
1X0110	0.00001	0.907377	0.092593	0.00001	0.00001	0.040655	0.00001	0.00001	0.00001	0.054109	0.905206
1X0606	0.00001	0.00001	0.99996	0.00001	0.00001	0.99995	0.00001	0.00001	0.00001	0.00001	0.00001
1X0612	0.00001	0.00001	0.7908	0.015548	0.193632	0.00001	0.00001	0.00001	0.99995	0.00001	0.00001
1X0632	0.00001	0.00001	0.99996	0.00001	0.00001	0.488534	0.00001	0.00001	0.000019	0.511417	0.00001
1X0951	0.00001	0.00001	0.99996	0.00001	0.00001	0.428216	0.000011	0.00001	0.00001	0.571743	0.00001
1X1032	0.00001	0.00001	0.99996	0.00001	0.00001	0.490049	0.00001	0.00001	0.00001	0.509911	0.00001
1X1125	0.00001	0.00001	0.99996	0.00001	0.00001	0.00001	0.00001	0.00001	0.00001	0.99995	0.00001
1X1126	0.00001	0.00001	0.99996	0.00001	0.00001	0.00001	0.00001	0.00001	0.00001	0.99995	0.00001
1X1146	0.00001	0.00001	0.99996	0.00001	0.00001	0.00001	0.00001	0.00001	0.00001	0.99995	0.00001
1X1181	0.00001	0.00001	0.99996	0.00001	0.00001	0.99995	0.00001	0.00001	0.00001	0.00001	0.00001
1X1672	0.00001	0.00001	0.954546	0.009525	0.035909	0.65839	0.00001	0.00001	0.34157	0.00001	0.00001
1X1700	0.00001	0.00001	0.99996	0.00001	0.00001	0.99995	0.00001	0.00001	0.00001	0.00001	0.00001
1X1765	0.00001	0.00001	0.99996	0.00001	0.00001	0.99995	0.00001	0.00001	0.00001	0.00001	0.00001
1X1939	0.00001	0.00001	0.99996	0.00001	0.00001	0.99995	0.00001	0.00001	0.00001	0.00001	0.00001
1X2891	0.00001	0.00001	0.99996	0.00001	0.00001	0.99995	0.00001	0.00001	0.00001	0.00001	0.00001
1X3162	0.00001	0.00001	0.99996	0.00001	0.00001	0.99995	0.00001	0.00001	0.00001	0.00001	0.00001
1X3576	0.00001	0.00001	0.99996	0.00001	0.00001	0.433398	0.00001	0.00001	0.00001	0.566562	0.00001
1X1979	0.00001	0.00001	0.96082	0.008048	0.051113	0.678622	0.00001	0.00001	0.321338	0.00001	0.00001

ID	K=7 *P. hamadryas*	K=7 *P. cynocephalus*	K=7 *P. anubis*	K=7 *P. papio*	K=7 *P. kindae*	K=7 *P. ursinus*	K=7 SNPRC *P. anubis*
PD_0720	0.00001	0.00001	0.99994	0.00001	0.00001	0.00001	0.00001
PD_0727	0.00001	0.00001	0.99994	0.00001	0.00001	0.00001	0.00001
PD_0746	0.00001	0.836875	0.00001	0.00001	0.056403	0.106681	0.00001
PD_0770	0.00001	0.867419	0.00001	0.00001	0.035938	0.096603	0.00001
HAP	0.00001	0.908166	0.00001	0.00001	0.00001	0.00001	0.091784
SAMN20949827	0.999936	0.00001	0.00001	0.000014	0.00001	0.00001	0.00001
SAMN20949837	0.99994	0.00001	0.00001	0.00001	0.00001	0.00001	0.00001
PD_0749	0.00001	0.00001	0.00001	0.00001	0.99994	0.00001	0.00001
PD_0751	0.000014	0.00001	0.00001	0.00001	0.999932	0.000014	0.00001
PD_0400	0.000014	0.00001	0.00001	0.999934	0.000012	0.00001	0.00001
PD_0401	0.00001	0.00001	0.00001	0.999939	0.000011	0.00001	0.00001
PD_0892	0.00001	0.00001	0.00001	0.000014	0.00001	0.999936	0.00001
PD_0893	0.00001	0.00001	0.00001	0.00001	0.00001	0.99994	0.00001
1X0576	0.00001	0.00001	0.596216	0.00001	0.00001	0.00001	0.403734
1X0580	0.00001	0.00001	0.591107	0.00001	0.00001	0.00001	0.408843
1X0153	0.00001	0.00001	0.159196	0.00001	0.00001	0.00001	0.840754
1X1947	0.00001	0.00001	0.043323	0.00001	0.00001	0.00001	0.956627
1X1152	0.00001	0.00001	0.00001	0.00001	0.00001	0.00001	0.99994
1X0102	0.00001	0.928214	0.00001	0.00001	0.00001	0.00001	0.071736
1X1734	0.00001	0.992148	0.00001	0.00001	0.00001	0.00001	0.007802
1X2049	0.00001	0.93752	0.00001	0.00001	0.00001	0.00001	0.06243
1X2208	0.00001	0.986591	0.00001	0.00001	0.00001	0.00001	0.013359
1X2304	0.00001	0.99994	0.00001	0.00001	0.00001	0.00001	0.00001
1X4384	0.00001	0.99994	0.00001	0.00001	0.00001	0.00001	0.00001
1Y3548	0.00001	0.99994	0.00001	0.00001	0.00001	0.00001	0.00001
1X0014	0.00001	0.00001	0.558279	0.00001	0.00001	0.00001	0.441671
1X0026	0.00001	0.00001	0.329886	0.00001	0.00001	0.00001	0.670064
1X0035	0.00001	0.00001	0.556785	0.00001	0.00001	0.00001	0.443165
1X0110	0.00001	0.904993	0.00001	0.00001	0.00001	0.00001	0.094957
1X0606	0.00001	0.00001	0.007782	0.00001	0.00001	0.00001	0.992168
1X0612	0.00001	0.00001	0.99994	0.00001	0.00001	0.00001	0.00001
1X0632	0.00001	0.00001	0.00001	0.00001	0.00001	0.00001	0.99994
1X0951	0.00001	0.00001	0.00001	0.00001	0.00001	0.00001	0.99994
1X1032	0.00001	0.00001	0.00001	0.00001	0.00001	0.00001	0.99994
1X1125	0.00001	0.00001	0.00001	0.00001	0.00001	0.00001	0.99994
1X1126	0.000012	0.00001	0.00001	0.00001	0.00001	0.00001	0.999938
1X1146	0.00001	0.00001	0.00001	0.00001	0.00001	0.00001	0.99994
1X1181	0.00001	0.00001	0.142104	0.00001	0.00001	0.00001	0.857846
1X1672	0.00001	0.00001	0.531882	0.00001	0.00001	0.00001	0.468068
1X1700	0.00001	0.00001	0.125636	0.00001	0.00001	0.00001	0.874314
1X1765	0.00001	0.00001	0.147451	0.000019	0.00001	0.00001	0.85249
1X1939	0.00001	0.00001	0.091492	0.000014	0.00001	0.00001	0.908453
1X2891	0.00001	0.00001	0.037555	0.000018	0.00001	0.00001	0.962387
1X3162	0.00001	0.00001	0.01086	0.00001	0.00001	0.00001	0.98909
1X3576	0.00001	0.00001	0.00001	0.00001	0.00001	0.00001	0.99994
1X1979	0.00001	0.00001	0.495376	0.00001	0.00001	0.00001	0.504574

ID	K=8 *P. kindae/P. ursin*	K=8 SNPRC *P. anubis*	K=8 *P. cynocephalus* (Tan	K=8 *P. hamadryas*	K=8 SNPRC *P. anubis*	K=8 *P. cynocephalus* (Ke	K=8 *P. papio*	K=8 *P. anubis*
PD_0720	0.00001	0.00001	0.00001	0.00001	0.00001	0.00001	0.00001	0.99993
PD_0727	0.00001	0.00001	0.00001	0.00001	0.00001	0.00001	0.00001	0.99993
PD_0746	0.00001	0.00001	0.99993	0.00001	0.00001	0.00001	0.00001	0.00001
PD_0770	0.00001	0.00001	0.99993	0.00001	0.00001	0.00001	0.00001	0.00001
HAP	0.00001	0.00976	0.00001	0.00001	0.00001	0.99018	0.00001	0.00001
SAMN20949827	0.00001	0.00001	0.00001	0.99993	0.00001	0.00001	0.00001	0.00001
SAMN20949837	0.00001	0.000018	0.00001	0.999922	0.00001	0.00001	0.00001	0.00001
PD_0749	0.99993	0.00001	0.00001	0.00001	0.00001	0.00001	0.00001	0.00001
PD_0751	0.99993	0.00001	0.00001	0.00001	0.00001	0.00001	0.00001	0.00001
PD_0400	0.00001	0.00001	0.00001	0.00001	0.00001	0.00001	0.99993	0.00001
PD_0401	0.00001	0.00001	0.00001	0.00001	0.00001	0.00001	0.99993	0.00001
PD_0892	0.99993	0.00001	0.00001	0.00001	0.00001	0.00001	0.00001	0.00001
PD_0893	0.99993	0.00001	0.00001	0.00001	0.00001	0.00001	0.00001	0.00001
1X0576	0.00001	0.242618	0.00001	0.00001	0.411884	0.00001	0.00001	0.345447
1X0580	0.00001	0.25417	0.00001	0.00001	0.397546	0.00001	0.00001	0.348234
1X0153	0.00001	0.733118	0.00001	0.00001	0.233834	0.00001	0.00001	0.032999
1X1947	0.00001	0.934248	0.00001	0.00001	0.036785	0.00001	0.00001	0.028918
1X1152	0.00001	0.99993	0.00001	0.00001	0.00001	0.00001	0.00001	0.00001
1X0102	0.00001	0.00001	0.00001	0.00001	0.00001	0.99993	0.00001	0.00001
1X1734	0.00001	0.00001	0.00001	0.00001	0.00001	0.99993	0.00001	0.00001
1X2049	0.00001	0.00001	0.00001	0.00001	0.00001	0.99993	0.00001	0.00001
1X2208	0.00001	0.00001	0.00001	0.00001	0.00001	0.99993	0.00001	0.00001
1X2304	0.00001	0.000018	0.094951	0.000014	0.000019	0.904962	0.000016	0.00001
1X4384	0.00001	0.00001	0.396594	0.00001	0.00001	0.603346	0.00001	0.00001
1Y3548	0.00001	0.00001	0.35535	0.00001	0.00001	0.64459	0.00001	0.00001
1X0014	0.00001	0.00001	0.00001	0.00001	0.99993	0.00001	0.00001	0.00001
1X0026	0.00001	0.446177	0.00001	0.00001	0.499821	0.00001	0.00001	0.053952
1X0035	0.00001	0.00001	0.00001	0.00001	0.99993	0.00001	0.00001	0.00001
1X0110	0.00001	0.014798	0.00001	0.00001	0.00001	0.985142	0.00001	0.00001
1X0606	0.00001	0.987127	0.00001	0.00001	0.00001	0.00001	0.00001	0.012813
1X0612	0.00001	0.00001	0.00001	0.00001	0.00001	0.00001	0.00001	0.99993
1X0632	0.00001	0.99993	0.00001	0.00001	0.00001	0.00001	0.00001	0.00001
1X0951	0.00001	0.99993	0.00001	0.00001	0.00001	0.00001	0.00001	0.00001
1X1032	0.00001	0.99993	0.00001	0.00001	0.00001	0.00001	0.00001	0.00001
1X1125	0.00001	0.99993	0.00001	0.00001	0.00001	0.00001	0.00001	0.00001
1X1126	0.00001	0.99993	0.00001	0.00001	0.00001	0.00001	0.00001	0.00001
1X1146	0.00001	0.99993	0.00001	0.00001	0.00001	0.00001	0.00001	0.00001
1X1181	0.00001	0.77724	0.00001	0.00001	0.173347	0.00001	0.00001	0.049363
1X1672	0.00001	0.00001	0.00001	0.00001	0.99993	0.00001	0.00001	0.00001
1X1700	0.00001	0.761793	0.00001	0.00001	0.238147	0.00001	0.00001	0.00001
1X1765	0.00001	0.75377	0.000011	0.00001	0.218734	0.00001	0.00001	0.027445
1X1939	0.00001	0.858413	0.00001	0.00001	0.094751	0.00001	0.00001	0.046786
1X2891	0.00001	0.967348	0.00001	0.00001	0.00001	0.00001	0.00001	0.032592
1X3162	0.00001	0.983254	0.00001	0.00001	0.00001	0.00001	0.00001	0.016686
1X3576	0.00001	0.99993	0.00001	0.00001	0.00001	0.00001	0.00001	0.00001
1X1979	0.00001	0.00001	0.00001	0.00001	0.99993	0.00001	0.00001	0.00001

ID	K=9 *P. papio*	K=9 *P. hamadryas*	K=9 *P. cynocephalus* (Tan	K=9 *P. anubis*	K=9 *P. cynocephalus* (Ke	K=9 SNPRC *P. anubis*	K=9 SNPRC *P. anubis*	K=9 SNPRC *P. anubis*	K=9 *P. kindae/P. ursinus*
PD_0720	0.00001	0.00001	0.00001	0.99992	0.00001	0.00001	0.00001	0.00001	0.00001
PD_0727	0.00001	0.00001	0.00001	0.99992	0.00001	0.00001	0.00001	0.00001	0.00001
PD_0746	0.00001	0.00001	0.99992	0.00001	0.00001	0.00001	0.00001	0.00001	0.00001
PD_0770	0.00001	0.00001	0.99992	0.00001	0.00001	0.00001	0.00001	0.00001	0.00001
HAP	0.00001	0.00001	0.00001	0.00001	0.990387	0.00001	0.009543	0.00001	0.00001
SAMN20949827	0.00001	0.99992	0.00001	0.00001	0.00001	0.00001	0.00001	0.00001	0.00001
SAMN20949837	0.00001	0.999909	0.00001	0.00001	0.00001	0.000019	0.00001	0.000013	0.00001
PD_0749	0.00001	0.00001	0.00001	0.00001	0.00001	0.00001	0.00001	0.00001	0.99992
PD_0751	0.00001	0.00001	0.00001	0.00001	0.000012	0.00001	0.00001	0.00001	0.999918
PD_0400	0.99992	0.00001	0.00001	0.00001	0.00001	0.00001	0.00001	0.00001	0.00001
PD_0401	0.99992	0.00001	0.00001	0.00001	0.00001	0.00001	0.00001	0.00001	0.00001
PD_0892	0.00001	0.00001	0.00001	0.00001	0.00001	0.00001	0.00001	0.00001	0.99992
PD_0893	0.00001	0.00001	0.00001	0.00001	0.00001	0.00001	0.00001	0.00001	0.99992
1X0576	0.00001	0.00001	0.00001	0.3272	0.00001	0.286822	0.385917	0.00001	0.00001
1X0580	0.00001	0.00001	0.00001	0.328368	0.00001	0.267527	0.404045	0.00001	0.00001
1X0153	0.00001	0.00001	0.00001	0.00001	0.00001	0.115639	0.884291	0.00001	0.00001
1X1947	0.00001	0.00001	0.00001	0.00001	0.00001	0.00001	0.99992	0.00001	0.00001
1X1152	0.00001	0.00001	0.00001	0.00001	0.00001	0.00001	0.000011	0.999919	0.00001
1X0102	0.00001	0.00001	0.00001	0.00001	0.99992	0.00001	0.00001	0.00001	0.00001
1X1734	0.00001	0.00001	0.00001	0.00001	0.99992	0.00001	0.00001	0.00001	0.00001
1X2049	0.00001	0.00001	0.00001	0.00001	0.99992	0.00001	0.00001	0.00001	0.00001
1X2208	0.00001	0.00001	0.00001	0.00001	0.99992	0.00001	0.00001	0.00001	0.00001
1X2304	0.00001	0.00001	0.095059	0.00001	0.904871	0.00001	0.00001	0.00001	0.00001
1X4384	0.00001	0.00001	0.396711	0.00001	0.603219	0.00001	0.00001	0.00001	0.00001
1Y3548	0.00001	0.00001	0.355402	0.00001	0.644528	0.00001	0.00001	0.00001	0.00001
1X0014	0.00001	0.00001	0.00001	0.00001	0.00001	0.99992	0.00001	0.00001	0.00001
1X0026	0.00001	0.00001	0.00001	0.020958	0.00001	0.414753	0.564229	0.00001	0.00001
1X0035	0.00001	0.00001	0.00001	0.00001	0.00001	0.99992	0.00001	0.00001	0.00001
1X0110	0.00001	0.00001	0.00001	0.00001	0.985075	0.00001	0.014855	0.00001	0.00001
1X0606	0.00001	0.00001	0.00001	0.00001	0.00001	0.00001	0.99992	0.00001	0.00001
1X0612	0.00001	0.00001	0.00001	0.99992	0.00001	0.00001	0.00001	0.00001	0.00001
1X0632	0.00001	0.00001	0.00001	0.00001	0.00001	0.00001	0.500032	0.499898	0.00001
1X0951	0.00001	0.00001	0.00001	0.00001	0.00001	0.00001	0.45138	0.54855	0.00001
1X1032	0.00001	0.00001	0.00001	0.00001	0.00001	0.00001	0.49016	0.50977	0.00001
1X1125	0.00001	0.00001	0.00001	0.00001	0.00001	0.00001	0.00001	0.99992	0.00001
1X1126	0.00001	0.00001	0.00001	0.00001	0.00001	0.00001	0.00001	0.99992	0.00001
1X1146	0.00001	0.00001	0.00001	0.00001	0.00001	0.00001	0.00001	0.99992	0.00001
1X1181	0.00001	0.00001	0.00001	0.00001	0.00001	0.00001	0.99992	0.00001	0.00001
1X1672	0.00001	0.00001	0.00001	0.00001	0.00001	0.99992	0.00001	0.00001	0.00001
1X1700	0.00001	0.00001	0.00001	0.00001	0.00001	0.135117	0.768745	0.096077	0.00001
1X1765	0.00001	0.00001	0.00001	0.00001	0.00001	0.00001	0.99992	0.00001	0.00001
1X1939	0.00001	0.00001	0.00001	0.00001	0.00001	0.000012	0.999918	0.00001	0.00001
1X2891	0.00001	0.00001	0.00001	0.00001	0.00001	0.00001	0.99992	0.00001	0.00001
1X3162	0.00001	0.00001	0.00001	0.00001	0.00001	0.00001	0.99992	0.00001	0.00001
1X3576	0.00001	0.00001	0.00001	0.00001	0.00001	0.00001	0.450745	0.549185	0.00001
1X1979	0.00001	0.00001	0.00001	0.00001	0.00001	0.99992	0.00001	0.00001	0.00001

ID	K=10 *P. papio*	K=10 *P. anubis*	K=10 *P. kindae/P. ursin*	K=10 SNPRC *P. anubis*	K=10 *P. cynocephalus* (Ke	K=10 SNPRC *P. cynoceph*	K=10 SNPRC *P. anubis*	K=10 SNPRC *P. anubis*	K=10 SNPRC *P. anubis*	K=10 *P. hamadryas*
PD_0720	0.00001	0.99991	0.00001	0.00001	0.00001	0.00001	0.00001	0.00001	0.00001	0.00001
PD_0727	0.00001	0.999906	0.00001	0.00001	0.00001	0.00001	0.000014	0.00001	0.00001	0.00001
PD_0746	0.00001	0.00001	0.00001	0.00001	0.00001	0.00001	0.00001	0.99991	0.00001	0.00001
PD_0770	0.00001	0.00001	0.00001	0.00001	0.00001	0.00001	0.00001	0.99991	0.00001	0.00001
HAP	0.00001	0.00001	0.00001	0.00001	0.99991	0.00001	0.00001	0.00001	0.00001	0.00001
SAMN20949827	0.00001	0.00001	0.00001	0.000012	0.00001	0.00001	0.00001	0.00001	0.00001	0.999908
SAMN20949837	0.00001	0.00001	0.00001	0.00001	0.00001	0.00001	0.00001	0.00001	0.00001	0.99991
PD_0749	0.00001	0.00001	0.99991	0.00001	0.00001	0.00001	0.00001	0.00001	0.00001	0.00001
PD_0751	0.00001	0.00001	0.999902	0.00001	0.000014	0.00001	0.00001	0.000014	0.00001	0.00001
PD_0400	0.999904	0.00001	0.00001	0.00001	0.000016	0.00001	0.00001	0.00001	0.00001	0.00001
PD_0401	0.99991	0.00001	0.00001	0.00001	0.00001	0.00001	0.00001	0.00001	0.00001	0.00001
PD_0892	0.00001	0.00001	0.99991	0.00001	0.00001	0.00001	0.00001	0.00001	0.00001	0.00001
PD_0893	0.00001	0.00001	0.999902	0.00001	0.00001	0.00001	0.00001	0.000018	0.00001	0.00001
1X0576	0.00001	0.327178	0.00001	0.286773	0.00001	0.00001	0.000015	0.00001	0.385974	0.00001
1X0580	0.00001	0.328357	0.00001	0.267476	0.00001	0.00001	0.00001	0.00001	0.404097	0.00001
1X0153	0.00001	0.00001	0.00001	0.115295	0.00001	0.00001	0.00001	0.00001	0.884625	0.00001
1X1947	0.00001	0.00001	0.00001	0.00001	0.00001	0.00001	0.00001	0.00001	0.99991	0.00001
1X1152	0.00001	0.00001	0.00001	0.00001	0.00001	0.00001	0.999904	0.00001	0.000016	0.00001
1X0102	0.00001	0.00001	0.00001	0.00001	0.00001	0.99991	0.00001	0.00001	0.00001	0.00001
1X1734	0.00001	0.00001	0.00001	0.00001	0.99991	0.00001	0.00001	0.00001	0.00001	0.00001
1X2049	0.00001	0.00001	0.00001	0.00001	0.766826	0.233094	0.00001	0.00001	0.00001	0.00001
1X2208	0.00001	0.00001	0.00001	0.00001	0.999909	0.00001	0.00001	0.000011	0.00001	0.00001
1X2304	0.00001	0.00001	0.00001	0.00001	0.809081	0.00001	0.00001	0.190839	0.00001	0.00001
1X4384	0.00001	0.00001	0.00001	0.00001	0.00001	0.00001	0.00001	0.99991	0.00001	0.00001
1Y3548	0.00001	0.00001	0.00001	0.00001	0.482101	0.00001	0.00001	0.517819	0.00001	0.00001
1X0014	0.00001	0.00001	0.00001	0.99991	0.00001	0.00001	0.00001	0.00001	0.00001	0.00001
1X0026	0.00001	0.020918	0.00001	0.41466	0.00001	0.00001	0.00001	0.00001	0.564352	0.00001
1X0035	0.000016	0.00001	0.00001	0.999901	0.00001	0.00001	0.000014	0.00001	0.00001	0.00001
1X0110	0.00001	0.00001	0.00001	0.00001	0.99991	0.00001	0.00001	0.00001	0.00001	0.00001
1X0606	0.00001	0.00001	0.00001	0.00001	0.00001	0.00001	0.00001	0.00001	0.99991	0.00001
1X0612	0.00001	0.999909	0.00001	0.00001	0.00001	0.00001	0.000011	0.00001	0.00001	0.00001
1X0632	0.00001	0.00001	0.00001	0.00001	0.00001	0.00001	0.499809	0.00001	0.500111	0.00001
1X0951	0.00001	0.00001	0.00001	0.000014	0.00001	0.00001	0.548468	0.00001	0.451449	0.00001
1X1032	0.00001	0.00001	0.00001	0.00001	0.00001	0.00001	0.509757	0.00001	0.490163	0.00001
1X1125	0.00001	0.00001	0.00001	0.00001	0.00001	0.00001	0.99991	0.00001	0.00001	0.00001
1X1126	0.00001	0.00001	0.00001	0.00001	0.00001	0.00001	0.999905	0.00001	0.000015	0.00001
1X1146	0.00001	0.00001	0.00001	0.00001	0.00001	0.00001	0.99991	0.00001	0.00001	0.00001
1X1181	0.00001	0.00001	0.00001	0.00001	0.00001	0.00001	0.00001	0.00001	0.99991	0.00001
1X1672	0.00001	0.00001	0.00001	0.99991	0.00001	0.00001	0.00001	0.00001	0.00001	0.00001
1X1700	0.00001	0.00001	0.00001	0.134858	0.00001	0.00001	0.095802	0.00001	0.769269	0.00001
1X1765	0.00001	0.00001	0.00001	0.000075	0.00001	0.00001	0.00001	0.00001	0.999845	0.00001
1X1939	0.00001	0.00001	0.00001	0.00001	0.00001	0.00001	0.00001	0.00001	0.99991	0.00001
1X2891	0.00001	0.00001	0.00001	0.00001	0.00001	0.00001	0.00001	0.00001	0.99991	0.00001
1X3162	0.00001	0.00001	0.00001	0.00001	0.00001	0.00001	0.00001	0.00001	0.99991	0.00001
1X3576	0.00001	0.00001	0.00001	0.00001	0.00001	0.00001	0.549068	0.00001	0.450852	0.00001
1X1979	0.00001	0.00001	0.00001	0.99991	0.00001	0.00001	0.00001	0.00001	0.00001	0.00001

	K=5					K=6						K=7						
ID	kindae/P. ursin	P. cynocephalus	P. anubis	P. papio	P. hamadryas	SNPRC P. anubis	amadryas/P. pa	kindae/P. ursin	P. anubis	NPRC P. anubis	P. cynocephalus	P. hamadryas	P. cynocephalus	P. anubis	P. papio	P. kindae	P. ursinus	NPRC P. anubis
1X2054	0.008783	0.003682	0.929999	0.028092	0.029443	0.652016	0.046303	0.008544	0.10037	0.189083	0.003684	0.020116	0.003653	0.170035	0.026259	0.006047	0.002681	0.771209
1X1960	0.012612	0.004103	0.931836	0.027516	0.023933	0.64398	0.041254	0.011924	0.097614	0.201109	0.00412	0.015299	0.004099	0.1677	0.025793	0.008236	0.004135	0.774738
14712	0.007789	0.00516	0.912905	0.032326	0.04182	0.631474	0.050472	0.008135	0.184111	0.120604	0.005204	0.021302	0.005171	0.281453	0.027726	0.005649	0.002457	0.656243
1Y0291	0.008727	0.003342	0.929961	0.0321	0.02587	0.644767	0.04281	0.008377	0.126773	0.173921	0.003352	0.013005	0.003322	0.207277	0.02932	0.005894	0.002908	0.738273
1X1487	0.007777	0.801093	0.191055	0.00001	0.000065	0.120925	0.00001	0.008988	0.004114	0.067231	0.798732	0.00001	0.798625	0.013242	0.00001	0.000088	0.009199	0.178825
1X4811	0.008288	0.811311	0.18033	0.00001	0.00006	0.112512	0.00001	0.008916	0.005943	0.06326	0.809359	0.00001	0.809265	0.014888	0.00001	0.000318	0.008863	0.166646
1X0351	0.158441	0.161061	0.583116	0.031926	0.065456	0.363246	0.077249	0.160157	0.130389	0.10713	0.161828	0.048521	0.162057	0.187414	0.028419	0.103252	0.057637	0.412699
1X0354	0.008323	0.009119	0.918266	0.029906	0.034386	0.645433	0.047932	0.008318	0.136403	0.152756	0.009158	0.019555	0.009054	0.227486	0.026655	0.006775	0.001891	0.708585
1X0356	0.007215	0.002458	0.811172	0.041401	0.137754	0.327432	0.103971	0.009275	0.497201	0.059633	0.002489	0.07502	0.002471	0.558275	0.031092	0.006239	0.002249	0.324653
1X0843	0.012106	0.00616	0.929509	0.021493	0.030732	0.493475	0.046833	0.010993	0.077928	0.364825	0.005945	0.025339	0.006071	0.124394	0.021171	0.008409	0.003486	0.81113
1X1230	0.012264	0.026211	0.872357	0.02862	0.060548	0.57221	0.057849	0.013265	0.229348	0.100914	0.026415	0.033323	0.026041	0.334101	0.02252	0.007133	0.005488	0.571393
1X1237	0.009155	0.018993	0.881946	0.028923	0.060984	0.568008	0.053576	0.010143	0.261998	0.087558	0.018717	0.029166	0.018211	0.376484	0.021903	0.005655	0.003754	0.544827
1X1392	0.010639	0.019519	0.882524	0.02746	0.059858	0.580011	0.053553	0.011822	0.242878	0.09237	0.019365	0.030559	0.019022	0.352306	0.020847	0.006605	0.004277	0.566385
1X1958	0.02171	0.452968	0.518278	0.001213	0.005832	0.335267	0.000351	0.021445	0.063353	0.127493	0.452091	0.000062	0.453094	0.105941	0.000229	0.008566	0.011518	0.420591
1X2055	0.012724	0.006027	0.927185	0.02945	0.024614	0.6426	0.044076	0.012038	0.098108	0.197167	0.006012	0.016021	0.005959	0.167198	0.027808	0.009934	0.002917	0.770163
1X2664	0.009255	0.010486	0.889643	0.032772	0.057844	0.593547	0.059045	0.010055	0.231899	0.094835	0.010617	0.030288	0.010379	0.339573	0.026502	0.006379	0.00323	0.583649
1X3321	0.145892	0.502996	0.318267	0.006147	0.026698	0.181542	0.024295	0.146988	0.049108	0.095358	0.502708	0.020045	0.504491	0.071507	0.005081	0.088425	0.057142	0.253309
1X4519	0.013378	0.028467	0.857307	0.030021	0.070827	0.55138	0.068066	0.01487	0.230397	0.106557	0.02873	0.043083	0.028383	0.328302	0.023822	0.009784	0.004262	0.562364
10046	0.002855	0.004129	0.95081	0.018933	0.023274	0.402623	0.03503	0.001969	0.101096	0.455375	0.003907	0.016672	0.00405	0.142425	0.018202	0.001456	0.001191	0.816003
10099	0.005006	0.001813	0.917397	0.028249	0.047535	0.618442	0.037261	0.004753	0.270275	0.067543	0.001726	0.012432	0.001609	0.406246	0.020202	0.003071	0.000967	0.555473
10164	0.002637	0.223706	0.729579	0.013253	0.030824	0.515661	0.011911	0.002502	0.212393	0.031528	0.226006	0.000765	0.225911	0.328746	0.005885	0.00001	0.001659	0.437024
10173	0.006352	0.001835	0.895917	0.021854	0.074042	0.575459	0.052453	0.007275	0.224795	0.138222	0.001797	0.037021	0.001829	0.298451	0.017047	0.004473	0.002457	0.638723
10192	0.008361	0.004621	0.928273	0.031683	0.027061	0.65062	0.043197	0.00805	0.130921	0.162565	0.004647	0.01393	0.004612	0.212999	0.028697	0.005716	0.002725	0.731321
10316	0.002378	0.002456	0.957297	0.023869	0.014	0.784232	0.028297	0.002112	0.079833	0.103077	0.002449	0.006728	0.002421	0.166978	0.022276	0.001423	0.000829	0.799344
10349	0.000413	0.448861	0.546786	0.002245	0.001695	0.380572	0.000804	0.000382	0.051733	0.119337	0.447172	0.000191	0.447049	0.088891	0.001366	0.000259	0.000128	0.462116
10418	0.017658	0.405259	0.570061	0.000333	0.00669	0.442098	0.00054	0.017605	0.066098	0.069134	0.404526	0.000352	0.405394	0.127847	0.00013	0.005492	0.010121	0.450664
10482	0.005285	0.003886	0.942521	0.027662	0.020645	0.6723	0.036144	0.00493	0.107171	0.175581	0.003874	0.010794	0.00386	0.180588	0.025675	0.002948	0.002159	0.773977
10488	0.024203	0.452077	0.518673	0.003514	0.001532	0.359564	0.000177	0.023242	0.062994	0.102887	0.451136	0.00001	0.452012	0.104956	0.001538	0.008539	0.013038	0.419906
10489	0.015521	0.01677	0.888668	0.027388	0.051653	0.509221	0.061103	0.015908	0.14739	0.249492	0.016886	0.036416	0.016868	0.210818	0.024302	0.010571	0.005264	0.69576
10842	0.002448	0.002335	0.959972	0.018775	0.01647	0.414909	0.031324	0.001738	0.078337	0.47147	0.002222	0.012409	0.002292	0.11708	0.018945	0.001592	0.000697	0.846984
10987	0.016978	0.015888	0.887531	0.027324	0.052277	0.514994	0.060888	0.017376	0.150813	0.239935	0.015994	0.036209	0.01594	0.215685	0.024158	0.0123	0.00519	0.690518
10998	0.002442	0.001728	0.919934	0.019987	0.055909	0.620716	0.041692	0.002587	0.189455	0.143863	0.001688	0.026176	0.001701	0.269257	0.015984	0.001656	0.000872	0.684354
11004	0.011897	0.234041	0.736778	0.00753	0.009754	0.504218	0.012591	0.011163	0.055455	0.18298	0.233592	0.005781	0.233765	0.102244	0.007168	0.004919	0.005788	0.640335
11158	0.001431	0.232076	0.72488	0.014006	0.027607	0.497815	0.019585	0.001749	0.158912	0.089154	0.232784	0.007899	0.232136	0.241268	0.009487	0.00001	0.002034	0.507167
11562	0.012295	0.272302	0.68226	0.012348	0.020795	0.481578	0.015435	0.012717	0.13558	0.082199	0.272492	0.004484	0.272856	0.21417	0.008811	0.003961	0.007769	0.487949
11608	0.00233	0.002073	0.947034	0.023275	0.025288	0.761688	0.03825	0.002297	0.091281	0.104456	0.002028	0.015674	0.002052	0.195219	0.021067	0.001243	0.001002	0.763742
11693	0.008884	0.17447	0.77694	0.017138	0.022569	0.512869	0.023604	0.008843	0.128357	0.151731	0.174595	0.008033	0.174591	0.202057	0.013984	0.002881	0.005692	0.592761
11752	0.004523	0.004768	0.940897	0.024638	0.025174	0.681365	0.038464	0.004286	0.101803	0.169329	0.004753	0.015918	0.004709	0.171019	0.022893	0.003106	0.001319	0.781035
11769	0.001867	0.247577	0.716424	0.01493	0.019203	0.50597	0.016551	0.00195	0.135778	0.091929	0.247823	0.003297	0.247715	0.216613	0.01132	0.000343	0.001488	0.519223
11770	0.000038	0.301711	0.621233	0.012457	0.064562	0.32147	0.037278	0.00005	0.248755	0.087094	0.305353	0.033596	0.303824	0.29636	0.006296	0.00003	0.000014	0.35988
11885	0.01214	0.004016	0.899209	0.026857	0.057777	0.441993	0.0635	0.01158	0.16052	0.318538	0.00387	0.040268	0.00394	0.196175	0.024191	0.009208	0.00322	0.722997
11887	0.011166	0.011904	0.919785	0.021921	0.035223	0.540727	0.046749	0.010853	0.103355	0.286488	0.011828	0.025894	0.011831	0.166386	0.020341	0.007657	0.003502	0.764389
11959	0.006737	0.414526	0.555684	0.007858	0.015195	0.35791	0.002659	0.006705	0.157726	0.059872	0.415128	0.000077	0.415227	0.226369	0.002495	0.000192	0.005648	0.349993
11967	0.000482	0.188649	0.768109	0.015399	0.027361	0.485815	0.022477	0.000607	0.158792	0.143291	0.189018	0.008633	0.188679	0.241818	0.011257	0.000055	0.000864	0.548694
11981	0.023362	0.227163	0.709823	0.015596	0.024057	0.433742	0.029038	0.023094	0.096853	0.190502	0.226772	0.014492	0.227314	0.148639	0.013862	0.014361	0.008428	0.572904
12138	0.004816	0.003878	0.941341	0.020384	0.029581	0.664584	0.03169	0.005229	0.146132	0.148339	0.004026	0.013179	0.003925	0.243414	0.016616	0.002943	0.001993	0.71793
12141	0.014023	0.168669	0.768765	0.020216	0.028326	0.526217	0.031124	0.014185	0.138909	0.120597	0.168968	0.01307	0.168953	0.218859	0.016829	0.004392	0.008949	0.568947
12152	0.01583	0.213549	0.75216	0.005852	0.012609	0.260593	0.014837	0.013915	0.065826	0.432286	0.212543	0.009371	0.213259	0.089225	0.005975	0.006508	0.008093	0.66757
12156	0.005613	0.344338	0.624835	0.010915	0.014299	0.414052	0.004984	0.005736	0.158704	0.072027	0.344497	0.000101	0.344894	0.232922	0.005699	0.00001	0.004292	0.412082
12175	0.000555	0.415763	0.581258	0.000345	0.002079	0.45486	0.000691	0.000533	0.035138	0.094345	0.414433	0.00034	0.414057	0.088381	0.000208	0.000022	0.000494	0.496498
12218	0.001284	0.253295	0.704755	0.014852	0.025814	0.483183	0.0194	0.001414	0.156051	0.085958	0.253996	0.006575	0.253834	0.237066	0.010477	0.00038	0.000872	0.490796
12226	0.020288	0.223202	0.713802	0.014318	0.02839	0.451073	0.027431	0.020625	0.125033	0.152599	0.22324	0.014767	0.223407	0.190073	0.011496	0.01116	0.008874	0.540223
12242	0.005764	0.002425	0.933537	0.027078	0.031196	0.756859	0.047344	0.005498	0.093869	0.094108	0.002322	0.02088	0.002391	0.200912	0.024707	0.003693	0.00195	0.745467
12282	0.018911	0.195198	0.749252	0.016597	0.020042	0.457963	0.027838	0.018307	0.089245	0.21193	0.194717	0.011924	0.19507	0.139599	0.015322	0.008016	0.009973	0.620095
12423	0.00027	0.384608	0.601915	0.008822	0.004385	0.403786	0.001149	0.000249	0.122331	0.08894	0.383545	0.00017	0.38284	0.183908	0.003992	0.000015	0.000208	0.428866
12427	0.005231	0.080083	0.874047	0.015516	0.025123	0.395965	0.031323	0.004475	0.107409	0.381345	0.079484	0.016094	0.080004	0.158385	0.013959	0.004043	0.001014	0.726502
12457	0.015701	0.191688	0.771866	0.005989	0.014757	0.273308	0.016737	0.013921	0.071255	0.43402	0.190758	0.011408	0.191295	0.098517	0.005955	0.005124	0.009331	0.67837
12473	0.000497	0.395681	0.59522	0.0038	0.004802	0.434674	0.002226	0.000487	0.065046	0.102822	0.394745	0.000156	0.394343	0.122728	0.002141	0.00001	0.000602	0.48002
12481	0.003501	0.002403	0.960878	0.017285	0.015933	0.357242	0.031487	0.002169	0.06784	0.539079	0.002183	0.014296	0.002314	0.091699	0.018043	0.002132	0.000911	0.870604
12491	0.000608	0.168624	0.800985	0.011809	0.017975	0.473732	0.014401	0.000461	0.137096	0.205988	0.168321	0.002675	0.168507	0.223657	0.008241	0.000947	0.00001	0.595962
12552	0.000187	0.000462	0.981807	0.013687	0.003857	0.538375	0.017448	0.000068	0.034429	0.409251	0.00043	0.003804	0.000438	0.089606	0.015211	0.00001	0.0001	0.890831
12656	0.056502	0.151385	0.739391	0.0224	0.030322	0.473413	0.039503	0.056336	0.111921	0.167449	0.151378	0.018537	0.151787	0.170942	0.020263	0.033529	0.022822	0.58212
12681	0.009451	0.441163	0.537677	0.001859	0.009849	0.358595	0.000398	0.008721	0.137685	0.053772	0.440829	0.000199	0.44124	0.210043	0.000063	0.000028	0.007014	0.341412
12717	0.003182	0.002397	0.959599	0.016763	0.018059	0.385996	0.031278	0.002175	0.075423	0.50284	0.002287	0.014702	0.002362	0.106604	0.017293	0.002171	0.000759	0.856109
12719	0.000979	0.196499	0.780649	0.006203	0.01567	0.281247	0.015355	0.000481	0.084333	0.4237	0.194884	0.01	0.195845	0.113655	0.00578	0.00001	0.000691	0.674021
12720	0.010598	0.254268	0.708829	0.012457	0.013848	0.352888	0.018095	0.009559	0.096556	0.269628	0.253274	0.006274	0.253977	0.139338	0.010824	0.003868	0.005868	0.579852
12869	0.00662	0.004161	0.82856	0.031025	0.129634	0.467651	0.119913	0.00932	0.283525	0.115316	0.004275	0.096491	0.004181	0.348293	0.024238	0.00469	0.002628	0.519478
12962	0.192074	0.027107	0.764515	0.007248	0.009057	0.274382	0.015046	0.191051	0.053496	0.440479	0.025546	0.010221	0.027224	0.075229	0.009088	0.024316	0.157818	0.696104
12963	0.005011	0.00757	0.929571	0.027983	0.029866	0.642054	0.039565	0.004984	0.14809	0.157632	0.007675	0.013991	0.007557	0.236831	0.024201	0.004118	0.001156	0.712147
13068	0.002155	0.001897	0.962126	0.018666	0.015156	0.711834	0.026589	0.002005	0.074533	0.183172	0.001868	0.009097	0.001843	0.147992	0.017712	0.001435	0.000629	0.821291
13103	0.003309	0.087385	0.869602	0.016937	0.022768	0.462228	0.026307	0.002642	0.129791	0.291892	0.08714	0.010152	0.087335	0.196771	0.014253	0.002702	0.000674	0.688112
13110	0.000209	0.000348	0.981454	0.012619	0.00537	0.528745	0.019516	0.000123	0.030276	0.421027	0.000314	0.006228	0.000334	0.085996	0.01406	0.00002	0.000109	0.893254
13152	0.017519	0.145654	0.79266	0.017221	0.026946	0.582786	0.029487	0.017802	0.114346	0.109528	0.146051	0.014568	0.145822	0.193013	0.014555	0.006732	0.009976	0.615334
13169	0.015849	0.157225	0.797363	0.009848	0.019715	0.295043	0.022814	0.014227	0.091375	0.420213	0.156328	0.013572	0.157046	0.125118	0.009209	0.005865	0.008884	0.680306
13225	0.016185	0.08839	0.847837	0.020643	0.026945	0.524368	0.030494	0.016049	0.144848	0.195696	0.088545	0.011163	0.088597	0.225065	0.017272	0.007789	0.008204	0.641909
13226	0.003681	0.00638	0.867688	0.033827	0.088423	0.492208	0.071743	0.004606	0.345691	0.079308	0.006444	0.045865	0.006392	0.433612	0.025552	0.002866	0.001192	0.484521
13228	0.014658	0.064701	0.871941	0.025001	0.023698	0.577185	0.037853	0.014031	0.105139	0.201131	0.064661	0.014532	0.064545	0.168879	0.023129	0.007457	0.006835	0.714624
13245	0.009677	0.009182	0.913232	0.027712	0.040195	0.579784	0.049205	0.00993	0.152122	0.199671	0.009288	0.023856	0.00919	0.231092	0.024161	0.006496	0.003457	0.701748
13264	0.004247	0.256623	0.554977	0.010703	0.17345	0.369948	0.165408	0.012376	0.0765	0.116248	0.25952	0.167844	0.256156	0.110927	0.009515	0.001907	0.002081	0.45157
13387	0.016331	0.189851	0.771583	0.007901	0.014334	0.264528	0.019744	0.014207	0.066846	0.445856	0.18882	0.011671	0.189317	0.089441	0.008363	0.007888	0.007451	0.685868
13463	0.006987	0.004261	0.926161	0.029861	0.03273	0.707798	0.048	0.007022	0.117959	0.115038	0.004184	0.019653	0.004238	0.215676	0.026954	0.004292	0.002715	0.726472
13570	0.020151	0.315008	0.642057	0.006815	0.01597	0.431138	0.014066	0.020173	0.080637	0.139486	0.3145	0.007802	0.315055	0.139162	0.005206	0.009781	0.009433	0.513561
13575	0.014968	0.199101	0.741114	0.018248	0.026569	0.50577	0.026196	0.015103	0.1415	0.112023	0.199409	0.010144	0.199497	0.216941	0.014749	0.005137	0.009304	0.544228
13589	0.162372	0.022082	0.795946	0.008817	0.010783	0.300589	0.0173	0.160812	0.061363	0.438965	0.020971	0.011031	0.022283	0.085107	0.010191	0.021921	0.132118	0.717348
13597	0.000016	0.000521	0.986214	0.006876	0.006373	0.154577	0.022119	0.00001	0.034966	0.787977	0.000351	0.011524	0.000442	0.019636	0.01089	0.00001	0.00001	0.957488
13644	0.020933	0.178669	0.789969	0.003081	0.007347	0.231194	0.014308	0.017669	0.020458	0.538369	0.178	0.01087	0.178653	0.021438	0.004877	0.009008	0.009784	0.765369
13645	0.02295	0.120672	0.814162	0.017903	0.024313	0.459582	0.033516	0.022075	0.094063	0.270258	0.120505	0.016433	0.120876	0.139534	0.01666	0.012447	0.009696	0.684354
13673	0.024399	0.098733	0.831749	0.018664	0.026455	0.466309	0.038432	0.023226	0.081763	0.2916	0.098671	0.020615	0.099027	0.124469	0.017728	0.013496	0.009807	0.714859
13694	0.000115	0.000444	0.984584	0.009393	0.005465	0.256994	0.021456	0.00001	0.030512	0.690686	0.000342	0.009683	0.000402	0.025032	0.013275	0.000029	0.00001	0.951569
13698	0.008697	0.002848	0.937442	0.021443	0.02957	0.523667	0.042229	0.008053	0.100017	0.323119	0.002914	0.021671	0.002917	0.150536	0.020108	0.006428	0.002369	0.795971
13739	0.029469	0.105307	0.823177	0.018638	0.023409	0.450969	0.036915	0.02821	0.07187	0.306934	0.105102	0.018782	0.105659	0.108879	0.018117	0.016674	0.011627	0.720262
13859	0.031682	0.129118	0.798924	0.015177	0.025099	0.452777	0.036429	0.030716	0.05628	0.295118	0.12868	0.021981	0.129137	0.091055	0.015184	0.016786	0.013603	0.712255
13914	0.018534	0.204052	0.747585	0.013625	0.016205	0.446188	0.024589	0.017316	0.064944	0.24329	0.203673	0.011238	0.204102	0.106434	0.013046	0.01026	0.00713	0.647789
13942	0.027792	0.10844	0.818679	0.018283	0.026806	0.459543	0.037386	0.026734	0.088159	0.279775	0.108403	0.019914	0.108698	0.133626	0.017149	0.015142	0.011711	0.693758
13951	0.010942	0.004074	0.9324	0.02338	0.029204	0.49094	0.049273	0.009128	0.066985	0.379745	0.003929	0.026371	0.003995	0.102026	0.023313	0.008012	0.002254	0.834028
13988	0.004147	0.004454	0.937818	0.020422	0.033159	0.512771	0.038601	0.003879	0.144042	0.296196	0.00451	0.019605	0.004574	0.205952	0.017765	0.003141	0.001241	0.747723
14012	0.016861	0.009051	0.907129	0.024552	0.042407	0.504357	0.054299	0.016456	0.11835	0.297528	0.00901	0.031414	0.009071	0.171335	0.022699	0.012215	0.004927	0.748339
14013	0.005374	0.005637	0.910765	0.031283	0.046941	0.622762	0.04304	0.005573	0.256372	0.066794	0.00546	0.015583	0.005275	0.381273	0.023875	0.00293	0.002052	0.569013
14016	0.000174	0.000502	0.984373	0.008616	0.006335	0.154437	0.022298	0.00001	0.042896	0.779999	0.00036	0.010185	0.000442	0.028276	0.012227	0.000083	0.00001	0.948776
14022	0.024617	0.082065	0.839944	0.020139	0.033235	0.459393	0.042737	0.02418	0.109891	0.281972	0.081826	0.023535	0.082108	0.160564	0.018452	0.013378	0.010822	0.691141
14058	0.005692	0.00628	0.906647	0.029788	0.051593	0.622405	0.04503	0.00607	0.258751	0.061665	0.006079	0.018871	0.005856	0.385567	0.02258	0.003094	0.002158	0.561875
14062	0.00279	0.00403	0.917189	0.02695	0.049041	0.568722	0.037468	0.002585	0.274148	0.113345	0.003732	0.013648	0.003561	0.403785	0.019107	0.001647	0.000566	0.557686
14066	0.010676	0.005085	0.928883	0.024883	0.030474	0.539372	0.048418	0.009685	0.082334	0.315161	0.005031	0.024111	0.005036	0.130906	0.024211	0.007539	0.002849	0.805347
14068	0.016372	0.006959	0.90107	0.025594	0.050006	0.442918	0.058527	0.015587	0.143718	0.332546	0.006703	0.036026	0.006849	0.176049	0.023545	0.010835	0.005426	0.741271
14076	0.000042	0.389583	0.60859	0.001556	0.000229	0.458713	0.000237	0.000044	0.060874	0.092185	0.387946	0.00001	0.387287	0.122573	0.000626	0.000057	0.00001	0.489438
14077	0.004482	0.002426	0.951493	0.021682	0.019917	0.58691	0.035995	0.003903	0.073429	0.297378	0.002386	0.014983	0.002385	0.125941	0.021241	0.002942	0.001259	0.831251
14154	0.004146	0.002389	0.954627	0.023113	0.015726	0.630033	0.033307	0.003609	0.067004	0.263707	0.002339	0.010939	0.00232	0.12348	0.022736	0.003132	0.000818	0.836576
14158	0.010095	0.157893	0.795956	0.018421	0.017635	0.587573	0.019788	0.009923	0.142191	0.082364	0.15816	0.003956	0.158074	0.222639	0.014711	0.002935	0.006416	0.59127
14167	0.011419	0.295128	0.663595	0.012941	0.016917	0.458406	0.015146	0.011381	0.114074	0.105907	0.295086	0.004025	0.295473	0.179473	0.010157	0.002661	0.007901	0.500311
14172	0.013449	0.502602	0.481807	0.001127	0.001015	0.326724	0.000114	0.012043	0.062509	0.097098	0.501512	0.000026	0.502349	0.103043	0.000135	0.003029	0.007666	0.383751
14181	0.00021	0.438716	0.559886	0.000198	0.000989	0.331504	0.000519	0.000207	0.040446	0.190732	0.436592	0.000305	0.436887	0.069598	0.000092	0.000052	0.000169	0.492897
14182	0.000256	0.346436	0.616248	0.007335	0.029725	0.348254	0.008484	0.000283	0.176269	0.118868	0.347842	0.006181	0.347742	0.2213	0.002721	0.000065	0.000199	0.421793
14191	0.009711	0.201785	0.753623	0.018259	0.016621	0.456865	0.021317	0.008997	0.131306	0.180157	0.201358	0.004374	0.201794	0.195965	0.015101	0.005413	0.003889	0.573465
14204	0.0132	0.004415	0.931336	0.023141	0.027908	0.501777	0.047393	0.011452	0.066603	0.368553	0.004221	0.024408	0.004322	0.105173	0.023211	0.008328	0.004125	0.830434
14250	0.000217	0.000509	0.938498	0.022415	0.038361	0.47584	0.028632	0.000138	0.232153	0.262755	0.000482	0.008725	0.000468	0.335424	0.015776	0.000201	0.00001	0.639396
14273	0.00931	0.494522	0.494915	0.000465	0.000788	0.340381	0.000133	0.008022	0.055968	0.102163	0.493334	0.000033	0.493396	0.097202	0.000148	0.00009	0.007464	0.401667
14276	0.014133	0.345	0.631364	0.00114	0.008363	0.342814	0.003964	0.013537	0.062682	0.232803	0.344199	0.002741	0.344718	0.097131	0.000702	0.008051	0.005411	0.541246
14282	0.004891	0.002639	0.953212	0.020197	0.019061	0.608825	0.035229	0.004253	0.062314	0.286815	0.002563	0.015599	0.002583	0.114286	0.02028	0.002093	0.002097	0.843062
14290	0.010477	0.270008	0.699708	0.009722	0.010086	0.454433	0.0131	0.009953	0.069041	0.184249	0.269223	0.003975	0.269625	0.117549	0.008767	0.003605	0.005968	0.590511
14324	0.016825	0.07788	0.850486	0.022311	0.032498	0.48257	0.042847	0.016458	0.11737	0.262953	0.077803	0.021617	0.077967	0.174254	0.020334	0.00804	0.00826	0.689527
14330	0.006281	0.003205	0.950391	0.020456	0.019666	0.563601	0.036659	0.005193	0.062241	0.329214	0.003092	0.016339	0.003099	0.109022	0.020523	0.004645	0.001205	0.845166
14342	0.002922	0.003412	0.962685	0.010974	0.020007	0.247657	0.029311	0.001312	0.082248	0.636236	0.003236	0.017555	0.003578	0.096256	0.011659	0.001492	0.001019	0.868441
14343	0.011648	0.071962	0.868577	0.024227	0.023585	0.647028	0.031986	0.011376	0.127708	0.109463	0.072438	0.010641	0.071979	0.201105	0.021317	0.007462	0.004124	0.683372
14350	0.003015	0.002292	0.927236	0.023796	0.043661	0.53466	0.035049	0.002891	0.236505	0.188664	0.002231	0.014006	0.002208	0.346426	0.017213	0.001554	0.001114	0.617479
14351	0.000505	0.000854	0.936467	0.023409	0.038764	0.512542	0.029553	0.000394	0.239177	0.217516	0.000818	0.008185	0.000809	0.351816	0.016676	0.000223	0.000146	0.622145
14355	0.000865	0.393369	0.594654	0.004036	0.007076	0.417897	0.002966	0.000888	0.079818	0.106051	0.392381	0.001061	0.392107	0.129851	0.001966	0.000077	0.000902	0.474036
14369	0.002141	0.34621	0.631557	0.006508	0.013585	0.44217	0.008173	0.002281	0.09458	0.106934	0.345861	0.003138	0.345516	0.149817	0.004406	0.00024	0.002255	0.494628
14379	0.020383	0.070189	0.858992	0.019684	0.030752	0.501144	0.038998	0.020083	0.114653	0.254985	0.070136	0.020438	0.070213	0.172895	0.017647	0.013482	0.006897	0.698429
14427	0.006579	0.308938	0.662738	0.003816	0.017928	0.4251	0.004039	0.00724	0.136278	0.118237	0.309106	0.001027	0.309614	0.213912	0.000493	0.001662	0.004409	0.468884
14435	0.015466	0.01056	0.906581	0.024235	0.043159	0.506127	0.054699	0.01513	0.116956	0.296549	0.010539	0.032181	0.01058	0.168266	0.022409	0.011433	0.004239	0.750893
14437	0.001476	0.004013	0.945918	0.023752	0.024842	0.538962	0.036904	0.001225	0.121221	0.297721	0.003968	0.014609	0.004017	0.180002	0.021668	0.00073	0.000655	0.778318
14460	0.003267	0.245095	0.709563	0.012555	0.029521	0.424118	0.017626	0.003695	0.172775	0.135952	0.245834	0.006556	0.246259	0.26277	0.007117	0.002511	0.000754	0.474034
14473	0.000015	0.000379	0.974214	0.014602	0.01079	0.263334	0.017174	0.00001	0.130819	0.588365	0.000297	0.006035	0.000364	0.156905	0.012642	0.000011	0.00001	0.824033
14498	0.001319	0.157608	0.795712	0.014698	0.030663	0.474903	0.019555	0.00143	0.192567	0.153159	0.158386	0.006384	0.158438	0.292616	0.009035	0.00001	0.001219	0.532298
14526	0.004847	0.006615	0.913154	0.02854	0.046844	0.637205	0.040414	0.005305	0.254391	0.05618	0.006504	0.015615	0.006351	0.383815	0.02111	0.002715	0.001772	0.568622
14642	0.008321	0.00753	0.928846	0.028019	0.027285	0.585141	0.042731	0.007928	0.116453	0.240174	0.007573	0.016346	0.007576	0.184914	0.025753	0.005486	0.002764	0.757161
14652	0.009176	0.003344	0.937255	0.022055	0.028169	0.531493	0.042008	0.008262	0.09581	0.318967	0.00346	0.020817	0.003439	0.143377	0.02086	0.007227	0.002067	0.802214
14668	0.000311	0.000324	0.969967	0.015809	0.013589	0.266907	0.021921	0.000098	0.13003	0.580771	0.000273	0.009209	0.000316	0.15722	0.013863	0.000137	0.000128	0.819126
14690	0.005457	0.00426	0.9365	0.02219	0.031593	0.55458	0.042268	0.005299	0.114672	0.278798	0.004383	0.021381	0.004338	0.176513	0.020342	0.003399	0.002013	0.772013
14695	0.023176	0.115854	0.82396	0.017276	0.019734	0.482662	0.034274	0.021797	0.050138	0.295554	0.115574	0.017016	0.115912	0.088432	0.01734	0.014089	0.008055	0.739156
14696	0.022899	0.100301	0.83083	0.019181	0.02679	0.46854	0.038032	0.021871	0.088006	0.283346	0.100204	0.019628	0.100517	0.132839	0.018234	0.013342	0.008747	0.706693
14740	0.004843	0.004926	0.915763	0.030707	0.043762	0.629695	0.040481	0.005059	0.248648	0.07121	0.004908	0.013633	0.004728	0.371187	0.02366	0.003111	0.0015	0.582181
14753	0.013402	0.051015	0.898426	0.021037	0.01612	0.683948	0.030756	0.012485	0.066068	0.155416	0.051327	0.010786	0.050961	0.130049	0.020356	0.007044	0.005456	0.775348
14756	0.015339	0.006736	0.916459	0.024128	0.037338	0.492779	0.053508	0.014364	0.092092	0.340628	0.006629	0.030469	0.006704	0.132442	0.023159	0.010605	0.004543	0.792079
14795	0.00473	0.002148	0.926528	0.026127	0.040466	0.585827	0.035816	0.004893	0.229839	0.141515	0.002109	0.012825	0.002058	0.345694	0.019463	0.003112	0.001555	0.615292
14800	0.007573	0.363922	0.612687	0.006792	0.009026	0.41032	0.00045	0.007335	0.132786	0.085339	0.363771	0.000034	0.363335	0.199927	0.002121	0.000047	0.00661	0.427927
14833	0.00484	0.00354	0.937238	0.022664	0.031718	0.532172	0.043974	0.004474	0.111734	0.304026	0.00362	0.022586	0.003603	0.164829	0.021011	0.003435	0.001477	0.78306
14847	0.009756	0.29329	0.680485	0.00501	0.011459	0.464587	0.005955	0.009894	0.089823	0.136877	0.292864	0.001485	0.293309	0.155239	0.003095	0.002598	0.006327	0.537947
14850	0.004448	0.002329	0.926977	0.021472	0.044774	0.689277	0.043882	0.004501	0.132577	0.127525	0.002238	0.024699	0.002286	0.22099	0.018917	0.002737	0.001645	0.728726
14852	0.002266	0.001628	0.927159	0.012596	0.056352	0.171915	0.057554	0.001471	0.146914	0.620645	0.001502	0.045413	0.001596	0.144956	0.011508	0.001805	0.000424	0.794298
14860	0.010287	0.006802	0.915068	0.032099	0.035744	0.640343	0.051041	0.010295	0.14108	0.150383	0.006858	0.02117	0.006796	0.225758	0.028788	0.008024	0.002716	0.706748
14863	0.003643	0.002958	0.944674	0.026085	0.02264	0.600098	0.036414	0.003448	0.114375	0.242653	0.003013	0.012156	0.00298	0.18405	0.023915	0.002243	0.001356	0.773301
14865	0.006069	0.00424	0.938714	0.024161	0.026816	0.494787	0.044927	0.005216	0.083849	0.367037	0.004184	0.021629	0.004226	0.124862	0.023752	0.004435	0.001494	0.819601
14867	0.000299	0.00065	0.985542	0.007796	0.005714	0.263713	0.018055	0.000074	0.035896	0.681744	0.000517	0.009188	0.000605	0.032582	0.011151	0.00001	0.000119	0.946345
14886	0.002652	0.002448	0.966673	0.012311	0.015916	0.280234	0.02709	0.001182	0.074677	0.614681	0.002136	0.014029	0.002358	0.091242	0.013399	0.001086	0.001073	0.876814

	K=5					K=6						K=7						
ID	kindae/P. ursin	?. cynocephalus	P. anubis	P. papio	P. hamadryas	SNPRC P. anubis	amadryas/P. pa	kindae/P. ursin	P. anubis	NPRC P. anubis	?. cynocephalus	P. hamadryas	?. cynocephalus	P. anubis	P. papio	P. kindae	P. ursinus	NPRC P. anubis
14908	0.014612	0.552904	0.430618	0.000017	0.00185	0.288468	0.000083	0.013452	0.05848	0.087749	0.551767	0.000087	0.552479	0.096821	0.00001	0.001279	0.010791	0.338534
14909	0.000348	0.000511	0.983721	0.00846	0.00696	0.149466	0.024189	0.000013	0.036156	0.789761	0.000416	0.011738	0.000482	0.020638	0.01243	0.000071	0.00008	0.95456
14916	0.004864	0.004949	0.925659	0.023544	0.040984	0.543762	0.041907	0.005132	0.186676	0.217503	0.00502	0.020892	0.004934	0.270328	0.01902	0.003578	0.00152	0.679727
14922	0.003262	0.003471	0.914111	0.024184	0.054971	0.662735	0.045526	0.003633	0.196383	0.088314	0.003409	0.025503	0.003447	0.291676	0.019604	0.002063	0.001311	0.656395
14924	0.002178	0.003209	0.924716	0.024946	0.044951	0.561084	0.033317	0.001931	0.263087	0.137508	0.003073	0.011753	0.002985	0.387379	0.017239	0.001267	0.00045	0.578928
14925	0.000382	0.000034	0.975413	0.012037	0.012133	0.256033	0.02945	0.000034	0.024937	0.689519	0.000026	0.017515	0.000031	0.018295	0.015472	0.000072	0.000039	0.948575
14930	0.005085	0.001193	0.929917	0.018326	0.045478	0.707065	0.038264	0.005021	0.137614	0.110907	0.001129	0.023523	0.001169	0.216081	0.015787	0.002927	0.001929	0.738583
14943	0.006822	0.268186	0.700746	0.01166	0.012586	0.481204	0.016151	0.006376	0.076479	0.152237	0.267554	0.004987	0.267732	0.13378	0.010318	0.002577	0.003695	0.576911
14944	0.018683	0.32492	0.634909	0.007062	0.014426	0.445125	0.007672	0.018919	0.106417	0.097091	0.324776	0.002542	0.325272	0.169936	0.004452	0.007095	0.010644	0.480058
14948	0.011842	0.215956	0.765666	0.000239	0.006298	0.138124	0.007774	0.00749	0.020894	0.608492	0.217225	0.009909	0.215764	0.017516	0.002059	0.005093	0.004877	0.744781
14951	0.002896	0.345967	0.553197	0.006054	0.091886	0.351457	0.085202	0.005572	0.066538	0.143402	0.347829	0.086697	0.345053	0.095596	0.005176	0.000297	0.002426	0.464757
14959	0.006213	0.00497	0.932642	0.023439	0.032737	0.522458	0.042984	0.005911	0.131226	0.292367	0.005053	0.020831	0.005044	0.189918	0.02108	0.004743	0.00177	0.756614
14989	0.006448	0.00311	0.943002	0.024847	0.022593	0.64674	0.039704	0.005927	0.079177	0.225359	0.003093	0.015869	0.003076	0.144522	0.023796	0.004584	0.001635	0.806518
14994	0.000079	0.000848	0.988401	0.007592	0.003079	0.293698	0.013819	0.00001	0.039317	0.652459	0.000696	0.005395	0.000788	0.040896	0.010984	0.00001	0.000014	0.941913
15009	0.010633	0.007164	0.898034	0.030881	0.053288	0.616788	0.048258	0.011127	0.254233	0.062725	0.00687	0.021451	0.00658	0.378409	0.023489	0.006355	0.003956	0.55976
15107	0.006815	0.001899	0.94666	0.020357	0.02427	0.608494	0.041187	0.006069	0.058148	0.28423	0.001872	0.021399	0.00187	0.108699	0.020262	0.004419	0.001823	0.841529
15112	0.012567	0.211979	0.741849	0.016604	0.017001	0.461612	0.020438	0.011961	0.122858	0.171469	0.211663	0.005285	0.212097	0.184905	0.013802	0.00512	0.006671	0.57212
15113	0.00856	0.305496	0.665831	0.008595	0.011518	0.414349	0.013847	0.008049	0.064461	0.1947	0.304593	0.005728	0.305053	0.107384	0.007754	0.003755	0.004034	0.566293
15122	0.012204	0.309801	0.660904	0.004792	0.0123	0.417932	0.007137	0.012298	0.088759	0.16457	0.309305	0.002427	0.309755	0.14885	0.003127	0.004133	0.007386	0.524321
15149	0.00997	0.220706	0.740217	0.015613	0.013494	0.460645	0.021045	0.009019	0.081183	0.207957	0.22015	0.005648	0.220424	0.133345	0.014392	0.00533	0.004183	0.616678
15150	0.005033	0.00193	0.911713	0.028316	0.053008	0.607699	0.0434	0.005232	0.262336	0.079508	0.001826	0.018898	0.00176	0.392334	0.020382	0.002817	0.001574	0.562236
15156	0.006585	0.005122	0.924247	0.02691	0.037136	0.549197	0.046362	0.006545	0.151895	0.24077	0.005232	0.021533	0.005185	0.222675	0.023664	0.004458	0.002276	0.720208
15157	0.019291	0.1756	0.769133	0.016023	0.019953	0.459526	0.028125	0.01865	0.08727	0.231398	0.175031	0.01263	0.175415	0.136304	0.014959	0.007636	0.010621	0.642434
15163	0.000873	0.000525	0.940141	0.021774	0.036686	0.454517	0.028414	0.00068	0.222052	0.29384	0.000496	0.008847	0.000519	0.318535	0.015652	0.0002	0.000463	0.655783
15175	0.016831	0.159973	0.813145	0.003525	0.006526	0.265702	0.012365	0.013902	0.035184	0.513788	0.159059	0.008649	0.159832	0.040804	0.005164	0.007241	0.007868	0.770442
15178	0.001396	0.0015	0.971327	0.014771	0.011006	0.291034	0.026536	0.000545	0.057553	0.622989	0.001344	0.011242	0.001447	0.071369	0.016423	0.000713	0.000368	0.898438
15190	0.000049	0.00001	0.971536	0.013927	0.014477	0.251877	0.000087	0.000025	0.127402	0.620598	0.00001	0.000016	0.00001	0.141497	0.015096	0.000024	0.000029	0.843328
15197	0.013346	0.31245	0.648979	0.00474	0.020485	0.387531	0.011055	0.013964	0.113282	0.161929	0.312239	0.007093	0.312746	0.175118	0.002309	0.00435	0.008412	0.489972
15211	0.004901	0.002671	0.90976	0.025423	0.057245	0.636267	0.049169	0.005237	0.193171	0.113513	0.002643	0.027869	0.00265	0.284538	0.020979	0.002684	0.002299	0.658981
15212	0.003952	0.002983	0.951078	0.022931	0.019056	0.691013	0.034783	0.003812	0.076835	0.190587	0.00297	0.012699	0.002925	0.145508	0.022055	0.003313	0.00062	0.81288
15217	0.02834	0.122199	0.813406	0.015755	0.0203	0.452369	0.035097	0.026671	0.046048	0.318076	0.121739	0.01969	0.122185	0.076748	0.016133	0.01398	0.012552	0.738711
15225	0.011542	0.532553	0.455369	0.00007	0.000466	0.319192	0.00001	0.010241	0.048523	0.090639	0.531394	0.000012	0.531647	0.085941	0.00001	0.000151	0.009349	0.372889
15232	0.006823	0.645882	0.346927	0.000041	0.000327	0.255417	0.00001	0.006663	0.02328	0.070512	0.644118	0.00001	0.644425	0.052883	0.00001	0.000439	0.005812	0.296421
15244	0.027178	0.097313	0.828804	0.019548	0.027156	0.447771	0.038194	0.026163	0.09559	0.295332	0.096949	0.01966	0.097513	0.140114	0.018282	0.015889	0.01048	0.698061
15267	0.000127	0.000293	0.983041	0.009052	0.007488	0.145188	0.025002	0.00001	0.036665	0.792934	0.000201	0.012405	0.000261	0.021155	0.012527	0.00001	0.000029	0.953613
15274	0.015758	0.073204	0.86473	0.017028	0.02928	0.478398	0.037264	0.01524	0.102749	0.29329	0.073058	0.021327	0.073238	0.152802	0.015619	0.007724	0.007368	0.721921
15279	0.000483	0.000872	0.981252	0.008414	0.008979	0.16103	0.025489	0.00001	0.03603	0.77674	0.000702	0.013525	0.000841	0.023512	0.012247	0.000043	0.000121	0.949712
15286	0.00001	0.00071	0.978123	0.01488	0.006276	0.261731	0.015557	0.00001	0.113438	0.608712	0.000552	0.003249	0.000675	0.132706	0.014018	0.000011	0.00001	0.849332
15290	0.016669	0.398604	0.562834	0.00608	0.015812	0.380931	0.007636	0.017084	0.107389	0.088531	0.398429	0.003108	0.399031	0.1666	0.003459	0.004694	0.011113	0.411994
15292	0.008499	0.00318	0.920359	0.027304	0.040657	0.581139	0.04153	0.008809	0.204411	0.16093	0.003182	0.01682	0.003051	0.307457	0.021665	0.00612	0.002723	0.642164
15308	0.014872	0.243266	0.716447	0.01129	0.014126	0.36464	0.016921	0.01397	0.096967	0.265156	0.242346	0.006645	0.24301	0.140631	0.00972	0.003906	0.009825	0.586264
15414	0.005268	0.003889	0.949623	0.02132	0.019899	0.592068	0.037247	0.004653	0.063077	0.299152	0.003803	0.016446	0.003818	0.113077	0.021345	0.00324	0.001575	0.840499
15419	0.004107	0.003673	0.941362	0.020131	0.030727	0.49261	0.041699	0.003466	0.110789	0.347835	0.003602	0.022168	0.003705	0.161434	0.018825	0.002637	0.001406	0.789825
15421	0.007531	0.003354	0.946075	0.021696	0.021345	0.645837	0.038156	0.006867	0.061743	0.244094	0.003303	0.017247	0.003221	0.116459	0.021585	0.00557	0.001671	0.834246
15442	0.000397	0.322779	0.592172	0.006564	0.078088	0.355039	0.074803	0.000602	0.062756	0.181527	0.325273	0.074122	0.321743	0.091606	0.005847	0.000067	0.000302	0.506313
15444	0.007519	0.002159	0.921655	0.021694	0.046974	0.704026	0.048462	0.007522	0.122875	0.115066	0.002049	0.028394	0.002128	0.219514	0.01909	0.005064	0.002471	0.723339
15458	0.007794	0.002617	0.912896	0.024136	0.052557	0.630401	0.047905	0.008073	0.164351	0.146743	0.002527	0.027373	0.002573	0.243409	0.020772	0.005607	0.002441	0.697824
15467	0.007028	0.002691	0.932306	0.022707	0.035268	0.515053	0.046918	0.006572	0.118186	0.3106	0.002672	0.025178	0.002711	0.172042	0.02084	0.005938	0.001515	0.771777
15475	0.005902	0.003171	0.909249	0.02404	0.057637	0.596047	0.048034	0.006246	0.189081	0.157461	0.00313	0.02844	0.003133	0.262204	0.020227	0.004405	0.001812	0.679778
15486	0.018499	0.165563	0.784909	0.014538	0.01649	0.451844	0.02805	0.017346	0.055244	0.282625	0.164892	0.013852	0.165367	0.092558	0.014392	0.010064	0.007411	0.696356
15494	0.004494	0.227582	0.722675	0.015323	0.029926	0.489897	0.018593	0.004923	0.184342	0.073513	0.228732	0.005066	0.228834	0.283745	0.009548	0.001854	0.002374	0.46858
15509	0.006441	0.002744	0.92591	0.025399	0.039506	0.575371	0.040201	0.006817	0.193094	0.181759	0.002758	0.017374	0.002702	0.293173	0.020226	0.004194	0.002445	0.659885
15515	0.028403	0.233465	0.70852	0.009835	0.019778	0.443732	0.023075	0.027767	0.070444	0.201685	0.233297	0.013783	0.233684	0.121601	0.008885	0.013173	0.013787	0.595088
15523	0.021457	0.251043	0.702142	0.006378	0.018981	0.428604	0.019358	0.021038	0.065105	0.215218	0.250677	0.013341	0.251074	0.112366	0.005795	0.011426	0.008877	0.597121
15545	0.003435	0.287947	0.680264	0.010692	0.017662	0.472128	0.013725	0.003629	0.113451	0.10916	0.287907	0.004842	0.288019	0.176903	0.008068	0.000836	0.002278	0.519054
15553	0.018145	0.103984	0.834258	0.01765	0.025963	0.481115	0.039384	0.016974	0.062452	0.296322	0.103754	0.022344	0.104007	0.105602	0.017385	0.011834	0.005337	0.733493
15560	0.003845	0.002485	0.966604	0.012264	0.014801	0.2713	0.03134	0.001865	0.049299	0.644017	0.002179	0.017121	0.002377	0.059148	0.014201	0.001755	0.001235	0.904163
15562	0.020809	0.13823	0.796906	0.015226	0.028829	0.435258	0.033526	0.020438	0.107465	0.265313	0.137999	0.019455	0.138316	0.156522	0.013481	0.011921	0.008403	0.651903
15566	0.010036	0.523202	0.465066	0.000161	0.001536	0.341781	0.000077	0.009123	0.055115	0.072061	0.521843	0.000063	0.522513	0.101339	0.00001	0.000265	0.007573	0.368239
15568	0.008773	0.325186	0.637978	0.011555	0.016508	0.422623	0.00563	0.008675	0.177758	0.0595	0.325816	0.000023	0.326184	0.259374	0.005589	0.000864	0.006307	0.401658
15571	0.001894	0.000764	0.935281	0.022521	0.039539	0.470508	0.032716	0.001538	0.222175	0.272332	0.00073	0.012489	0.000734	0.31778	0.016689	0.001302	0.00051	0.650495
15575	0.004742	0.002798	0.933115	0.022477	0.036869	0.48809	0.03362	0.004476	0.2017	0.269373	0.002741	0.013184	0.002778	0.295003	0.017189	0.003092	0.001628	0.667126
15579	0.027641	0.230576	0.71214	0.00988	0.019762	0.436149	0.023205	0.027052	0.066398	0.216869	0.230326	0.013945	0.230693	0.114252	0.009007	0.014067	0.012407	0.60563
15581	0.008768	0.619821	0.370666	0.000038	0.000707	0.248616	0.000044	0.007491	0.048486	0.076713	0.618649	0.000015	0.61864	0.080571	0.000026	0.000113	0.007306	0.29333
15584	0.021595	0.129873	0.80573	0.016643	0.026159	0.481603	0.034645	0.021144	0.087299	0.24581	0.129498	0.01898	0.129705	0.1377	0.015465	0.013787	0.007401	0.676962
15599	0.010987	0.341575	0.628094	0.007526	0.011817	0.443696	0.006803	0.011039	0.099185	0.097961	0.341315	0.00146	0.341792	0.160461	0.005032	0.001431	0.00829	0.481534
15600	0.017814	0.147043	0.798235	0.014205	0.022703	0.51225	0.028357	0.017401	0.086509	0.208453	0.147031	0.01522	0.147191	0.144884	0.012779	0.009244	0.007636	0.663047
15626	0.013004	0.008487	0.915268	0.023795	0.039445	0.485554	0.049033	0.012618	0.130087	0.314303	0.008404	0.026693	0.008492	0.186649	0.021544	0.009422	0.003868	0.743332
15628	0.008866	0.006777	0.897152	0.032087	0.055118	0.593255	0.047932	0.00899	0.277324	0.066042	0.006456	0.01984	0.00611	0.408617	0.024166	0.005002	0.003066	0.533199
15633	0.007671	0.002701	0.943285	0.022767	0.023576	0.559639	0.042933	0.006511	0.061744	0.326541	0.002633	0.020324	0.002646	0.107866	0.022873	0.00472	0.002235	0.839336
15652	0.023491	0.090166	0.815817	0.022525	0.048	0.430145	0.051051	0.023675	0.152553	0.252423	0.090154	0.032451	0.090338	0.192958	0.019789	0.015636	0.007999	0.640828
15653	0.00008	0.385655	0.614167	0.000061	0.000037	0.49586	0.00001	0.00006	0.074903	0.045315	0.383854	0.00001	0.382796	0.157655	0.000014	0.00001	0.000083	0.459432
15659	0.00605	0.005237	0.935657	0.024831	0.028225	0.48988	0.038463	0.005546	0.139919	0.320994	0.005198	0.015523	0.005269	0.204418	0.022092	0.003904	0.002223	0.746571
15664	0.002444	0.001538	0.954182	0.023999	0.017836	0.560328	0.028148	0.002132	0.142778	0.265082	0.001532	0.007308	0.00154	0.213891	0.020564	0.00163	0.000761	0.754306
15733	0.031266	0.051921	0.855289	0.017187	0.044337	0.495685	0.055022	0.031268	0.072102	0.293833	0.052091	0.039232	0.052038	0.121444	0.016636	0.019882	0.011115	0.739652
15736	0.011935	0.191519	0.766345	0.0132	0.017	0.496242	0.02218	0.011291	0.080409	0.198766	0.191113	0.010022	0.191269	0.134087	0.012012	0.00433	0.006659	0.64162
15820	0.01124	0.347645	0.610378	0.012258	0.018479	0.413879	0.015871	0.011426	0.113957	0.097382	0.347485	0.005084	0.348133	0.17753	0.009213	0.00525	0.005408	0.449381
15824	0.002512	0.001979	0.970174	0.011945	0.01339	0.346136	0.025676	0.001299	0.054397	0.57067	0.001822	0.01361	0.001937	0.070842	0.013492	0.001183	0.000797	0.89814
15845	0.004485	0.003881	0.907153	0.024454	0.060027	0.644114	0.050374	0.005089	0.194874	0.101753	0.003797	0.030167	0.003858	0.286847	0.019832	0.002669	0.002007	0.654621
15846	0.008194	0.525941	0.464383	0.000283	0.0012	0.317802	0.000271	0.007248	0.043514	0.106461	0.524703	0.000142	0.524277	0.075923	0.000095	0.000228	0.007435	0.391899
15849	0.000413	0.528121	0.468665	0.000237	0.002564	0.324055	0.000036	0.000431	0.062799	0.086369	0.52631	0.000036	0.526233	0.104888	0.000012	0.00001	0.000397	0.368424
15870	0.003287	0.002812	0.963968	0.015207	0.014727	0.334831	0.029602	0.001881	0.065214	0.565914	0.002559	0.013641	0.002738	0.086639	0.016388	0.001556	0.001073	0.877965
15874	0.005337	0.005996	0.908848	0.029988	0.049831	0.628048	0.043616	0.005488	0.263454	0.053544	0.00585	0.01753	0.005591	0.393699	0.022202	0.003162	0.00174	0.556076
15889	0.003605	0.004182	0.959509	0.01544	0.017263	0.371585	0.030936	0.002418	0.070631	0.520449	0.003981	0.01474	0.004161	0.100845	0.016295	0.001775	0.00137	0.860814
15938	0.002572	0.00297	0.961335	0.016571	0.016552	0.37052	0.031978	0.001662	0.068855	0.524229	0.002755	0.014841	0.002877	0.098798	0.017237	0.001836	0.000448	0.863962
15940	0.001642	0.001833	0.966837	0.015538	0.01415	0.372419	0.029422	0.000846	0.057332	0.538326	0.001655	0.013664	0.001777	0.078586	0.016805	0.00085	0.000448	0.887868
15944	0.008074	0.004269	0.926673	0.02557	0.035414	0.512268	0.049994	0.007589	0.11777	0.308114	0.004265	0.025596	0.004316	0.169872	0.023862	0.005438	0.002735	0.768182
15975	0.008828	0.268743	0.686442	0.020567	0.01542	0.454944	0.015107	0.008204	0.176035	0.076819	0.268891	0.000293	0.269242	0.258065	0.014927	0.002846	0.004538	0.450089
15976	0.010482	0.006117	0.922118	0.027251	0.034031	0.493211	0.049547	0.009735	0.118478	0.323004	0.006025	0.023267	0.006138	0.171978	0.02551	0.007419	0.003114	0.762573
15979	0.004636	0.002756	0.949351	0.022039	0.021218	0.655342	0.035497	0.004273	0.079256	0.222897	0.002735	0.014613	0.002725	0.145266	0.021088	0.003094	0.001274	0.811941
15996	0.020134	0.236227	0.717191	0.010323	0.016124	0.444947	0.022277	0.019119	0.054425	0.223417	0.235816	0.01246	0.23625	0.097252	0.010052	0.008387	0.01006	0.625539
16000	0.004846	0.003068	0.960027	0.014763	0.017296	0.374723	0.030924	0.003602	0.065381	0.52247	0.0029	0.015212	0.002997	0.095591	0.015665	0.002514	0.001929	0.866092
16006	0.014885	0.144826	0.802513	0.017239	0.020537	0.491649	0.027629	0.014259	0.102376	0.219578	0.144508	0.01146	0.144724	0.159574	0.015562	0.004895	0.00903	0.654755
16017	0.006182	0.007884	0.926929	0.023103	0.035902	0.550668	0.043235	0.006118	0.141397	0.25056	0.008022	0.021809	0.007991	0.212831	0.020239	0.003209	0.002808	0.731115
16019	0.007235	0.002834	0.916655	0.027154	0.046122	0.621691	0.041346	0.007742	0.232774	0.093637	0.00281	0.017456	0.00271	0.353582	0.020334	0.004935	0.002316	0.598667
16056	0.002652	0.002194	0.941653	0.021663	0.031839	0.496537	0.044154	0.002202	0.107799	0.347106	0.002202	0.023584	0.002245	0.155539	0.020435	0.001375	0.001198	0.795624
16058	0.003377	0.274957	0.687027	0.011464	0.023175	0.477177	0.010045	0.003904	0.169973	0.063037	0.275864	0.001061	0.275987	0.263306	0.005963	0.00001	0.003019	0.450653
16059	0.005133	0.409382	0.571349	0.003374	0.010762	0.389198	0.001647	0.005399	0.104225	0.090619	0.408912	0.000292	0.409368	0.166044	0.000884	0.00001	0.004227	0.419175
16065	0.000218	0.37633	0.620666	0.000949	0.001837	0.301027	0.000477	0.000173	0.039712	0.284292	0.374319	0.00004	0.375065	0.065082	0.000669	0.000092	0.000054	0.558998
16067	0.006928	0.002327	0.94746	0.021516	0.021769	0.604892	0.038779	0.006111	0.065509	0.282402	0.002307	0.017654	0.002279	0.119056	0.021303	0.004843	0.001592	0.833273
16072	0.008209	0.233602	0.73515	0.00942	0.013619	0.402693	0.016305	0.007464	0.080687	0.260001	0.23285	0.006737	0.2333	0.1246	0.008534	0.002414	0.00504	0.619375
16083	0.004731	0.00353	0.936714	0.023468	0.031557	0.509719	0.044107	0.004174	0.117949	0.320535	0.003516	0.02157	0.00356	0.173208	0.021656	0.003605	0.001256	0.775144
16092	0.006779	0.005974	0.932817	0.024263	0.030166	0.487979	0.043224	0.006038	0.117749	0.339101	0.005909	0.020128	0.006001	0.168337	0.022591	0.004873	0.001893	0.776177
16094	0.008778	0.188913	0.769575	0.016892	0.015842	0.567611	0.018006	0.008539	0.128453	0.08844	0.188952	0.003457	0.188909	0.202713	0.013589	0.003063	0.005121	0.583148
16101	0.002481	0.002158	0.925009	0.027516	0.042836	0.630425	0.035434	0.002753	0.251246	0.078002	0.00214	0.011412	0.002063	0.3826	0.019753	0.001207	0.000976	0.581988
16104	0.006024	0.007442	0.927308	0.022261	0.036966	0.517922	0.045597	0.005757	0.12999	0.293245	0.007489	0.024958	0.007578	0.188616	0.019923	0.003034	0.002802	0.753088
16112	0.001143	0.002295	0.970952	0.016002	0.009608	0.416375	0.02007	0.000821	0.08035	0.480225	0.002159	0.005576	0.002229	0.11766	0.016177	0.000691	0.00038	0.857286
16115	0.009636	0.558724	0.43079	0.000053	0.000797	0.321092	0.000062	0.008968	0.042163	0.07046	0.557256	0.000041	0.557447	0.084411	0.000027	0.000106	0.008211	0.349758
16121	0.023481	0.217859	0.728286	0.009325	0.021049	0.424692	0.023316	0.022879	0.074717	0.236785	0.217611	0.0147	0.21796	0.120813	0.00834	0.013461	0.009112	0.615614
16122	0.008683	0.005723	0.928506	0.024926	0.032162	0.492118	0.046486	0.007955	0.111004	0.33677	0.005666	0.022761	0.005725	0.160813	0.023377	0.006038	0.002622	0.778664
16243	0.012021	0.312534	0.654796	0.005227	0.015422	0.422933	0.006993	0.012404	0.108965	0.136399	0.312305	0.001991	0.312932	0.178584	0.002789	0.004213	0.007106	0.492386
16244	0.004092	0.002782	0.927703	0.027803	0.037619	0.617778	0.037698	0.004379	0.210368	0.126964	0.002813	0.013282	0.002714	0.317714	0.021847	0.002902	0.00129	0.640251
16246	0.005749	0.630387	0.363639	0.000034	0.000192	0.269481	0.00001	0.005679	0.022708	0.073531	0.62859	0.000015	0.628647	0.054155	0.00001	0.000213	0.00541	0.31155
16254	0.014822	0.123705	0.818599	0.017411	0.025463	0.478154	0.032713	0.014419	0.106185	0.245044	0.123485	0.01612	0.123714	0.161983	0.015614	0.008336	0.00602	0.668213
16261	0.003436	0.002032	0.933708	0.023339	0.037485	0.488853	0.032761	0.003144	0.212056	0.261202	0.001984	0.011769	0.001999	0.308713	0.017582	0.002102	0.001223	0.656611
16263	0.023276	0.103197	0.824459	0.021818	0.02725	0.45677	0.039637	0.022439	0.101208	0.27714	0.102806	0.018746	0.103219	0.149308	0.020376	0.014126	0.008671	0.685554
16288	0.003463	0.002832	0.951497	0.023237	0.018971	0.6309	0.036596	0.003113	0.068598	0.258029	0.002765	0.014085	0.002745	0.12682	0.02285	0.00232	0.000944	0.830235
16289	0.008741	0.28698	0.684439	0.005136	0.014705	0.438039	0.009211	0.008908	0.095199	0.162004	0.286639	0.004213	0.287079	0.161204	0.003282	0.002564	0.00535	0.536308
16300	0.015558	0.098621	0.81684	0.022113	0.046868	0.446366	0.048368	0.015846	0.160834	0.229888	0.098698	0.030346	0.098784	0.207346	0.01908	0.009936	0.005656	0.628851
16304	0.015378	0.273214	0.686802	0.009384	0.015222	0.443679	0.020202	0.014732	0.051188	0.19759	0.272609	0.011099	0.273171	0.095557	0.008966	0.010339	0.003968	0.5969
16316	0.001689	0.001863	0.942491	0.022766	0.03119	0.509482	0.027276	0.001525	0.205024	0.254861	0.001833	0.007426	0.001836	0.299266	0.017206	0.001055	0.000598	0.672612
16321	0.012618	0.200373	0.77497	0.005127	0.006912	0.229237	0.012996	0.009704	0.036249	0.511942	0.199871	0.007935	0.200088	0.046221	0.006557	0.004694	0.006367	0.728139
16329	0.013063	0.5116	0.4688	0.000179	0.006358	0.313612	0.00001	0.012413	0.083135	0.080005	0.510825	0.00001	0.511845	0.129483	0.00001	0.001581	0.009003	0.348068
16338	0.005068	0.002524	0.920877	0.028568	0.042962	0.623123	0.038752	0.005369	0.237522	0.092757	0.002477	0.013875	0.002396	0.357897	0.021667	0.002213	0.002447	0.599506
16363	0.000883	0.002288	0.973791	0.012134	0.010904	0.289916	0.02388	0.000356	0.060485	0.623255	0.002107	0.010505	0.002247	0.077376	0.0137	0.000523	0.00016	0.895489
16369	0.020699	0.175789	0.768107	0.014176	0.021229	0.4588	0.031314	0.019696	0.055876	0.259026	0.175289	0.017758	0.175638	0.09941	0.013841	0.010831	0.00857	0.673952
16371	0.006361	0.195935	0.749046	0.015476	0.033183	0.506876	0.019488	0.006834	0.20169	0.067847	0.197265	0.006215	0.197315	0.308644	0.009231	0.002447	0.003374	0.472775
16372	0.00456	0.001291	0.962571	0.015244	0.016334	0.753286	0.029607	0.004001	0.038696	0.173125	0.001286	0.014919	0.001241	0.090969	0.016134	0.002672	0.001119	0.872947
16383	0.012387	0.564349	0.422419	0.000149	0.000696	0.284351	0.00001	0.011164	0.048824	0.092528	0.563123	0.000013	0.563971	0.08486	0.00001	0.000439	0.009303	0.341404
16385	0.016733	0.145574	0.801916	0.012435	0.023342	0.468863	0.025032	0.016491	0.106081	0.238097	0.145436	0.012845	0.145558	0.166476	0.010688	0.010416	0.006216	0.647802
16386	0.022977	0.195312	0.74764	0.012254	0.021817	0.43616	0.028766	0.022106	0.064923	0.252882	0.195162	0.0169	0.195475	0.107589	0.011658	0.01482	0.007273	0.646285
16391	0.021501	0.280097	0.674924	0.006646	0.016832	0.41638	0.017384	0.021141	0.064137	0.20137	0.279588	0.011391	0.279975	0.109423	0.005868	0.011436	0.009072	0.572836
16400	0.000341	0.300343	0.663821	0.006942	0.028553	0.364267	0.006494	0.000374	0.18344	0.14386	0.301564	0.004581	0.301536	0.230153	0.002349	0.000106	0.000274	0.461003
16408	0.004451	0.00214	0.929832	0.023483	0.040093	0.701567	0.042227	0.004342	0.129327	0.120436	0.0021	0.020911	0.00209	0.21963	0.02081	0.002985	0.001466	0.732108
16409	0.003227	0.002899	0.959527	0.017892	0.016456	0.388775	0.030708	0.002268	0.076288	0.499297	0.002663	0.012621	0.00282	0.110902	0.018507	0.00169	0.001209	0.852251
16413	0.006618	0.002075	0.902516	0.025367	0.063424	0.612121	0.055003	0.007188	0.188242	0.135393	0.002053	0.033918	0.002067	0.268884	0.02126	0.005327	0.001762	0.666782
16417	0.006436	0.404951	0.571622	0.005097	0.011894	0.392432	0.003579	0.006526	0.103922	0.088756	0.404785	0.000273	0.405171	0.163832	0.002425	0.000607	0.005021	0.422671
16420	0.009734	0.004881	0.934869	0.021726	0.028789	0.48523	0.043723	0.008663	0.088584	0.368957	0.004844	0.022801	0.004931	0.130406	0.021105	0.006091	0.003313	0.811352
16424	0.011376	0.492562	0.494177	0.001036	0.000848	0.336573	0.000132	0.010101	0.057413	0.10436	0.491422	0.000035	0.491888	0.097653	0.00016	0.000313	0.008703	0.401248
16436	0.015083	0.188084	0.786952	0.004621	0.00526	0.345277	0.009513	0.012999	0.046521	0.398583	0.187105	0.004508	0.187869	0.069484	0.005365	0.005484	0.00797	0.719321
16486	0.020679	0.127537	0.8117	0.019655	0.020429	0.505337	0.033793	0.019556	0.073157	0.240826	0.12733	0.014622	0.127593	0.124697	0.018868	0.011397	0.008308	0.694515
16499	0.000367	0.349276	0.635788	0.007278	0.007291	0.445058	0.005255	0.000328	0.077582	0.123211	0.348566	0.000739	0.348292	0.12645	0.005446	0.000067	0.000286	0.518719
16507	0.004272	0.003318	0.907771	0.02681	0.05783	0.586142	0.062443	0.004694	0.164383	0.179008	0.00333	0.039702	0.003291	0.222986	0.023597	0.002859	0.001492	0.706074
16510	0.008488	0.004262	0.934425	0.027589	0.025237	0.698405	0.041973	0.008125	0.09926	0.14793	0.004307	0.016271	0.004217	0.175092	0.025663	0.006023	0.00253	0.770205
16517	0.006521	0.002565	0.914661	0.024334	0.051918	0.643496	0.047282	0.006805	0.171313	0.128589	0.002515	0.026497	0.002539	0.2569	0.02059	0.004124	0.002536	0.686816
16524	0.008089	0.005425	0.907108	0.029435	0.049943	0.62024	0.042885	0.008635	0.258887	0.064143	0.00521	0.017661	0.005009	0.384856	0.021885	0.005224	0.002709	0.562656
16527	0.005001	0.003327	0.951917	0.017677	0.022078	0.387485	0.03526	0.003791	0.086911	0.483413	0.00314	0.017252	0.003261	0.12308	0.017456	0.003047	0.001732	0.834173
16538	0.002239	0.001134	0.930976	0.024214	0.041437	0.506469	0.03377	0.001986	0.237175	0.219528	0.001072	0.012469	0.001056	0.342913	0.017622	0.00119	0.00079	0.623959
16548	0.006958	0.002801	0.922017	0.024942	0.043282	0.655344	0.043184	0.006903	0.150559	0.141304	0.002705	0.020881	0.002747	0.23383	0.022029	0.004394	0.002651	0.713468
16550	0.012474	0.007457	0.92041	0.022397	0.037262	0.478276	0.048666	0.011744	0.115567	0.338359	0.007388	0.027542	0.007463	0.165122	0.020677	0.008901	0.003732	0.766563
16562	0.006671	0.002981	0.926949	0.026739	0.03666	0.57763	0.039852	0.006846	0.187712	0.184935	0.003025	0.015867	0.002967	0.283801	0.021632	0.004848	0.002136	0.668749
16569	0.00108	0.002038	0.975883	0.013018	0.007982	0.413246	0.016914	0.000723	0.077395	0.489818	0.001905	0.005204	0.001986	0.111989	0.013131	0.000148	0.000762	0.866781
16671	0.020685	0.162903	0.775584	0.01648	0.024348	0.485887	0.033315	0.020103	0.077459	0.220447	0.162788	0.017409	0.163022	0.132238	0.015426	0.011253	0.00855	0.652102
16678	0.010181	0.106766	0.84511	0.016218	0.021725	0.498784	0.030059	0.009664	0.092782	0.262452	0.106259	0.014286	0.10653	0.146121	0.01501	0.005861	0.003786	0.708405
16692	0.010108	0.004274	0.9328	0.022824	0.029994	0.480824	0.0474	0.008729	0.081724	0.377247	0.004076	0.025142	0.004178	0.118552	0.022215	0.007623	0.002299	0.819992

	K=5					K=6						K=7						
ID	kindae/P. ursin	. cynocephalus	P. anubis	P. papio	P. hamadryas	SNPRC P. anubis	amadryas/P. pa	kindae/P. ursin	P. anubis	NPRC P. anubis	. cynocephalus	P. hamadryas	. cynocephalus	P. anubis	P. papio	P. kindae	P. ursinus	NPRC P. anubis
16702	0.010463	0.007231	0.923457	0.025174	0.033675	0.480154	0.048729	0.009408	0.111118	0.343495	0.007096	0.024424	0.007258	0.158791	0.023629	0.007684	0.002733	0.77548
16708	0.005203	0.002682	0.922117	0.0284	0.041598	0.622287	0.038061	0.005621	0.233186	0.098238	0.002606	0.013128	0.002553	0.352396	0.021277	0.003533	0.001707	0.605406
16719	0.021569	0.158839	0.777626	0.016523	0.025443	0.485415	0.033785	0.02111	0.082993	0.218075	0.158622	0.018365	0.158849	0.136793	0.015195	0.012214	0.008612	0.649972
16731	0.021466	0.235641	0.709741	0.010893	0.022258	0.424554	0.02549	0.020869	0.077222	0.216422	0.235443	0.015196	0.235829	0.126365	0.009763	0.011943	0.008451	0.592453
16741	0.006238	0.006157	0.90651	0.02881	0.052285	0.611564	0.042481	0.006305	0.272238	0.061485	0.005927	0.017421	0.005635	0.404414	0.021134	0.003708	0.001771	0.545917
16743	0.012497	0.557106	0.428553	0.000352	0.001491	0.285631	0.000363	0.011175	0.056483	0.090386	0.555962	0.000209	0.556724	0.093257	0.000115	0.000934	0.008998	0.339763
16744	0.01282	0.183948	0.772917	0.011442	0.018872	0.459121	0.021891	0.012393	0.088235	0.234749	0.18361	0.010553	0.183815	0.145994	0.010038	0.006773	0.005563	0.637264
16748	0.007103	0.179503	0.767695	0.015604	0.030095	0.516695	0.020744	0.007416	0.182057	0.092646	0.180442	0.006817	0.18048	0.282012	0.010352	0.004725	0.002114	0.5135
16761	0.000248	0.333782	0.658708	0.004088	0.003174	0.492721	0.000113	0.000243	0.101896	0.072346	0.33268	0.000029	0.331723	0.18454	0.000449	0.000195	0.000025	0.483038
16775	0.004712	0.002953	0.932646	0.02228	0.03741	0.521818	0.043175	0.004661	0.151547	0.27578	0.003018	0.022832	0.003056	0.217862	0.01915	0.003285	0.001597	0.732217
16780	0.006767	0.288762	0.669597	0.01353	0.021344	0.46334	0.018928	0.007061	0.121107	0.100835	0.288729	0.007059	0.289016	0.189429	0.01046	0.002934	0.003568	0.497533
16787	0.013906	0.283972	0.684318	0.005733	0.012072	0.438362	0.009272	0.013827	0.078211	0.176965	0.283362	0.003602	0.283793	0.133801	0.0045	0.005774	0.007459	0.56107
16798	0.008585	0.290561	0.676356	0.011689	0.012809	0.472318	0.00586	0.008473	0.151961	0.070658	0.290729	0.00001	0.291051	0.233689	0.006699	0.00001	0.00671	0.461831
16808	0.00253	0.003322	0.908789	0.024503	0.060856	0.639436	0.047811	0.002761	0.213794	0.0929	0.003297	0.027967	0.003285	0.305536	0.019318	0.00192	0.000837	0.641137
16815	0.017196	0.220469	0.73071	0.013291	0.018335	0.431381	0.024036	0.01659	0.081597	0.226533	0.219863	0.011434	0.220272	0.128214	0.012119	0.007155	0.009102	0.611704
16816	0.013179	0.396984	0.565946	0.008867	0.015024	0.374895	0.011179	0.013344	0.100615	0.10339	0.396576	0.003678	0.397265	0.157603	0.006309	0.003713	0.008597	0.422835
16823	0.004495	0.004457	0.948399	0.020056	0.022593	0.700709	0.030611	0.004516	0.108143	0.151544	0.004477	0.012373	0.004449	0.192945	0.017738	0.002762	0.001607	0.768126
16827	0.00556	0.003046	0.925853	0.027487	0.038054	0.599421	0.037391	0.005852	0.211901	0.142355	0.003081	0.013285	0.002977	0.318327	0.021361	0.004005	0.001736	0.638309
16829	0.002479	0.002201	0.932911	0.022535	0.039874	0.518619	0.03064	0.002265	0.237207	0.209158	0.002111	0.011025	0.002091	0.346117	0.015919	0.000831	0.001232	0.622786
16847	0.000185	0.490129	0.509316	0.000136	0.000234	0.316941	0.000195	0.000186	0.031933	0.162729	0.488016	0.0001	0.488246	0.05873	0.000093	0.000075	0.000115	0.452642
16853	0.006058	0.417029	0.555998	0.007229	0.013686	0.354539	0.000905	0.006003	0.166285	0.054872	0.417397	0.000053	0.41787	0.236501	0.001372	0.000715	0.003864	0.339625
16856	0.006099	0.003346	0.950205	0.022288	0.018063	0.708	0.032388	0.005623	0.080257	0.170376	0.003355	0.011256	0.003287	0.150111	0.021293	0.004393	0.001536	0.808124
16861	0.012719	0.01327	0.920888	0.0204	0.032724	0.606835	0.046584	0.01237	0.074441	0.246393	0.013377	0.027203	0.013209	0.13172	0.019872	0.008488	0.00394	0.795568
16862	0.015074	0.560674	0.418833	0.000163	0.005256	0.272909	0.000162	0.014185	0.076754	0.076082	0.559908	0.000103	0.561065	0.117861	0.000048	0.002549	0.009735	0.30864
16866	0.007928	0.490028	0.49947	0.000406	0.002168	0.342488	0.000721	0.006884	0.048879	0.112093	0.488934	0.000466	0.488317	0.083973	0.000165	0.000216	0.007296	0.419567
16873	0.005359	0.003994	0.942704	0.021295	0.026648	0.635657	0.035163	0.005356	0.11602	0.203683	0.00412	0.015734	0.004032	0.188839	0.019009	0.003307	0.002071	0.767009
16879	0.02503	0.194215	0.743206	0.014096	0.023453	0.431706	0.031003	0.024336	0.071614	0.24756	0.19378	0.017473	0.194204	0.114926	0.013391	0.014463	0.009633	0.635909
16880	0.003724	0.003096	0.961189	0.016762	0.015229	0.379535	0.029104	0.002703	0.073171	0.512554	0.002934	0.012164	0.003059	0.102748	0.017394	0.002462	0.001017	0.861156
16889	0.01159	0.547466	0.440061	0.000022	0.000861	0.293963	0.00001	0.010155	0.058847	0.090735	0.54629	0.00001	0.546262	0.096654	0.00001	0.00001	0.009817	0.347236
16890	0.002524	0.378571	0.594506	0.010876	0.013524	0.376163	0.003966	0.002485	0.168357	0.070317	0.378712	0.000215	0.378349	0.239915	0.0049	0.00003	0.002197	0.374394
16986	0.007071	0.003774	0.938529	0.026654	0.023972	0.711207	0.039046	0.006796	0.102966	0.136207	0.003778	0.014736	0.003743	0.179531	0.024652	0.004445	0.002514	0.770379
16999	0.007889	0.004915	0.939982	0.022936	0.024278	0.66295	0.035637	0.007647	0.10493	0.183849	0.004987	0.014292	0.004848	0.176556	0.020918	0.006606	0.001489	0.775292
17006	0.014301	0.392965	0.583732	0.002787	0.006215	0.389813	0.001858	0.013933	0.074262	0.128014	0.392121	0.00055	0.392648	0.123342	0.001478	0.00258	0.010122	0.469279
17024	0.007671	0.342766	0.637638	0.006212	0.005712	0.484317	0.001097	0.007392	0.099869	0.064954	0.34237	0.000084	0.342356	0.174752	0.002635	0.00001	0.005785	0.474378
17111	0.00001	0.412899	0.580729	0.00209	0.004272	0.381711	0.00025	0.00001	0.078226	0.128311	0.411492	0.000094	0.411372	0.125832	0.000059	0.00001	0.00001	0.462622
17112	0.005293	0.003908	0.941681	0.020182	0.028936	0.541499	0.040281	0.004767	0.099593	0.309883	0.003976	0.021002	0.004002	0.151538	0.019054	0.003669	0.001476	0.799259
17139	0.00421	0.30202	0.625811	0.008805	0.059154	0.408533	0.056323	0.006204	0.076804	0.149736	0.3024	0.05224	0.301443	0.116176	0.007608	0.001154	0.00266	0.518718
17141	0.003644	0.416604	0.573759	0.002837	0.003157	0.404564	0.000897	0.003167	0.062529	0.11332	0.415523	0.000262	0.415437	0.10771	0.00119	0.000444	0.002519	0.472438
17153	0.023702	0.095663	0.8342	0.018768	0.027667	0.489273	0.041565	0.022755	0.064949	0.28608	0.095378	0.023732	0.095719	0.109328	0.018247	0.014252	0.008467	0.730255
17158	0.001355	0.002363	0.942958	0.023008	0.030316	0.557663	0.036537	0.001334	0.154075	0.247997	0.002394	0.015275	0.002386	0.228019	0.019813	0.000761	0.000604	0.733142
17159	0.022936	0.173115	0.766278	0.016089	0.021583	0.47209	0.03273	0.021852	0.060661	0.239806	0.172861	0.017015	0.173188	0.108363	0.015548	0.011425	0.010212	0.664248
17165	0.000128	0.448505	0.54965	0.000255	0.001462	0.3263	0.000567	0.000134	0.042179	0.184327	0.446493	0.000351	0.446787	0.070539	0.000101	0.000013	0.000129	0.48208
17174	0.012295	0.165598	0.792779	0.012881	0.016447	0.611745	0.018037	0.012015	0.093311	0.098897	0.165995	0.006392	0.165633	0.176092	0.010737	0.005569	0.006005	0.629571
17184	0.025154	0.11199	0.814446	0.02091	0.0275	0.446761	0.040078	0.024184	0.091424	0.285886	0.111666	0.020018	0.112131	0.134206	0.019733	0.015221	0.009314	0.689376
17190	0.016694	0.292834	0.658433	0.013655	0.018384	0.444417	0.01564	0.016669	0.129641	0.100767	0.292866	0.004111	0.293424	0.198673	0.010083	0.007448	0.008419	0.477841
17199	0.007474	0.001811	0.914299	0.023954	0.052461	0.660736	0.052157	0.007803	0.14429	0.13327	0.001744	0.030616	0.001774	0.232167	0.02095	0.005264	0.002436	0.706793
17222	0.02172	0.075555	0.853756	0.021917	0.027051	0.484808	0.042289	0.020668	0.081253	0.295794	0.075188	0.020728	0.075467	0.132282	0.021094	0.012351	0.008698	0.72938
17228	0.006074	0.054577	0.89563	0.022278	0.02144	0.584203	0.034384	0.005464	0.096492	0.22478	0.054678	0.0131	0.054377	0.160154	0.020684	0.003889	0.002018	0.745778
17232	0.019213	0.265608	0.687636	0.007559	0.019983	0.432914	0.022326	0.018747	0.058553	0.202128	0.265332	0.015323	0.265644	0.106093	0.006928	0.010147	0.007809	0.588056
17233	0.005628	0.002706	0.923542	0.025986	0.042137	0.612926	0.038171	0.006046	0.220561	0.119597	0.002699	0.01568	0.002628	0.332497	0.019829	0.002915	0.002596	0.623855
17254	0.006479	0.599995	0.392944	0.000058	0.000524	0.286708	0.000064	0.005833	0.031766	0.077196	0.598433	0.000071	0.598801	0.066644	0.000016	0.000209	0.005026	0.329233
17255	0.010593	0.368651	0.597646	0.009318	0.013792	0.382584	0.001482	0.010289	0.177852	0.058572	0.369221	0.000114	0.369184	0.256379	0.002733	0.000066	0.008682	0.362842
17268	0.00001	0.362737	0.609623	0.003083	0.024548	0.327414	0.000557	0.00001	0.189701	0.118784	0.363534	0.000548	0.363708	0.230868	0.000097	0.00001	0.00001	0.404758
17294	0.003782	0.003069	0.9385	0.021902	0.032746	0.521128	0.042029	0.003474	0.12917	0.301093	0.003106	0.021352	0.003132	0.187988	0.019646	0.002802	0.001091	0.763989
17295	0.012872	0.320569	0.649879	0.008014	0.008667	0.410644	0.011321	0.012224	0.058104	0.188078	0.319629	0.003511	0.320226	0.100664	0.007432	0.004136	0.007604	0.556427
17325	0.003964	0.00325	0.939851	0.024542	0.028394	0.536553	0.042515	0.003593	0.112878	0.301182	0.00328	0.018706	0.003279	0.169893	0.022937	0.002679	0.001352	0.781155
17328	0.00502	0.005726	0.925797	0.025476	0.03798	0.574709	0.03958	0.005199	0.186613	0.1881	0.0058	0.016664	0.005731	0.281574	0.020588	0.00296	0.002106	0.670377
17340	0.003158	0.00175	0.931123	0.024052	0.039917	0.695733	0.042834	0.003089	0.130193	0.126445	0.001706	0.020839	0.00172	0.21975	0.021354	0.002308	0.000878	0.733152
17776	0.002876	0.005346	0.940327	0.023134	0.028317	0.559706	0.036465	0.002742	0.139975	0.255697	0.005414	0.015015	0.005403	0.212698	0.02026	0.001302	0.001464	0.743858
17792	0.00005	0.381142	0.608284	0.004414	0.00611	0.398083	0.001963	0.000048	0.079374	0.140376	0.380156	0.000382	0.380008	0.127148	0.002357	0.00001	0.000013	0.490081
17795	0.001881	0.335531	0.642842	0.011306	0.00844	0.43283	0.012236	0.001687	0.071457	0.147367	0.334423	0.002382	0.334152	0.119298	0.009865	0.00016	0.002141	0.532004
17826	0.00386	0.397193	0.587709	0.00357	0.007668	0.405424	0.003346	0.003992	0.068092	0.122887	0.396259	0.000893	0.396368	0.115524	0.002241	0.00001	0.003659	0.481305
17829	0.000054	0.405477	0.587974	0.002944	0.00355	0.392721	0.000742	0.000094	0.075972	0.126518	0.403953	0.000188	0.403784	0.124395	0.000956	0.000011	0.00005	0.470615
17832	0.002549	0.002024	0.93263	0.019172	0.043625	0.694288	0.038091	0.002529	0.140943	0.122202	0.001947	0.022319	0.001955	0.216886	0.016538	0.001894	0.000672	0.739736
17833	0.012816	0.543511	0.442687	0.000347	0.00064	0.299787	0.000073	0.011602	0.05229	0.094019	0.542229	0.00001	0.54316	0.087764	0.000113	0.001601	0.008509	0.358841
17840	0.010888	0.331249	0.624847	0.012906	0.02011	0.420772	0.016363	0.011037	0.123876	0.096617	0.331334	0.005202	0.331775	0.189451	0.009725	0.002306	0.00788	0.45366
17851	0.014654	0.204286	0.730837	0.021196	0.029027	0.501237	0.028401	0.014785	0.164919	0.085895	0.204763	0.009816	0.204925	0.2494	0.016592	0.004788	0.009208	0.505271
17852	0.021633	0.108119	0.822132	0.018991	0.029125	0.483689	0.040838	0.020915	0.083096	0.263673	0.107789	0.022776	0.108066	0.13019	0.018076	0.012815	0.00804	0.700036
17871	0.004519	0.002239	0.919615	0.028838	0.044789	0.613813	0.040655	0.004724	0.240626	0.097976	0.002206	0.014983	0.002136	0.362233	0.021937	0.002671	0.001575	0.594466
17873	0.000944	0.001215	0.967782	0.016886	0.013173	0.323197	0.022751	0.000558	0.118444	0.533976	0.001074	0.008174	0.001177	0.14989	0.015371	0.000655	0.000246	0.824487
17879	0.020564	0.270733	0.684271	0.007851	0.016581	0.427204	0.02006	0.019745	0.050874	0.211853	0.270263	0.013007	0.270719	0.0919	0.007482	0.0096	0.009331	0.59796
17891	0.010379	0.013036	0.905735	0.026106	0.044745	0.573249	0.047664	0.010885	0.181726	0.173197	0.013279	0.02416	0.013044	0.276518	0.021359	0.007407	0.003283	0.65423
17898	0.00016	0.366994	0.624775	0.003356	0.004714	0.404858	0.000644	0.000155	0.090023	0.138628	0.365692	0.00015	0.365436	0.147458	0.000719	0.00001	0.000134	0.486093
17903	0.008438	0.002695	0.911796	0.025859	0.051212	0.66982	0.050392	0.008612	0.154002	0.11458	0.002594	0.027478	0.002658	0.24506	0.022528	0.005226	0.003314	0.693736
17916	0.00001	0.00065	0.971543	0.017551	0.010245	0.364156	0.018894	0.00001	0.122816	0.493582	0.000543	0.005361	0.000623	0.158677	0.015394	0.00001	0.00001	0.819924
17929	0.018738	0.173626	0.770433	0.017112	0.020092	0.469716	0.027821	0.018141	0.095451	0.215663	0.173207	0.011582	0.173625	0.14932	0.015621	0.006619	0.010953	0.632282
17969	0.004792	0.217069	0.743071	0.016387	0.01868	0.520939	0.022501	0.004586	0.106425	0.128658	0.216892	0.007355	0.216888	0.175588	0.014038	0.002029	0.00247	0.581632
17970	0.006873	0.215295	0.744835	0.016031	0.016965	0.522156	0.022487	0.006394	0.09157	0.142278	0.215115	0.007799	0.214949	0.15077	0.014302	0.000872	0.005346	0.605963
17971	0.00001	0.000582	0.963606	0.021738	0.014063	0.376799	0.016048	0.00001	0.204642	0.40199	0.000511	0.001615	0.000579	0.270128	0.014885	0.00001	0.00001	0.712773
17981	0.013542	0.021891	0.919238	0.024074	0.021254	0.663723	0.038582	0.012543	0.068892	0.193872	0.022389	0.015595	0.021965	0.131037	0.023313	0.009259	0.003743	0.795088
17997	0.001544	0.001727	0.960868	0.018762	0.017099	0.376897	0.02567	0.00106	0.129966	0.464735	0.001672	0.009424	0.001723	0.175483	0.016515	0.000683	0.000755	0.795417
17998	0.005649	0.002722	0.939423	0.021145	0.031061	0.691861	0.030968	0.006184	0.159031	0.109115	0.002841	0.012964	0.002748	0.249245	0.017034	0.003864	0.002048	0.712097
17999	0.001005	0.001355	0.963015	0.017793	0.016833	0.342675	0.026052	0.000584	0.12826	0.501219	0.00121	0.010346	0.001326	0.1683	0.015679	0.000474	0.000439	0.803437
18004	0.018236	0.294304	0.670882	0.005831	0.010747	0.421279	0.014523	0.01742	0.035192	0.21801	0.293575	0.008861	0.294022	0.072636	0.005874	0.008554	0.008305	0.601749
18015	0.016435	0.540435	0.441658	0.000194	0.001278	0.301422	0.000107	0.015124	0.055201	0.088812	0.539334	0.000056	0.540146	0.093468	0.000045	0.002371	0.011281	0.352632
18019	0.007567	0.312228	0.592702	0.009794	0.077709	0.381049	0.074313	0.010524	0.079201	0.141887	0.313026	0.07084	0.311779	0.117411	0.008442	0.002128	0.004883	0.484517
18022	0.004842	0.002067	0.953942	0.020017	0.019131	0.594645	0.035432	0.004186	0.063377	0.300331	0.002028	0.016004	0.002024	0.114741	0.019926	0.002923	0.001502	0.84288
18024	0.006129	0.023906	0.932453	0.018496	0.019016	0.426527	0.027463	0.004974	0.135332	0.382387	0.023318	0.010425	0.023815	0.190434	0.015795	0.002873	0.002855	0.753802
18141	0.001849	0.002195	0.961652	0.017096	0.017208	0.349462	0.026323	0.001208	0.126657	0.494269	0.002079	0.011116	0.002191	0.167861	0.014874	0.000721	0.000916	0.80232
18144	0.011102	0.020897	0.904628	0.027134	0.036239	0.594039	0.049088	0.01106	0.123878	0.200895	0.021041	0.023939	0.020893	0.19909	0.02456	0.006472	0.004559	0.720487
18168	0.00001	0.2991	0.691863	0.006438	0.002589	0.457569	0.004158	0.00001	0.060534	0.180018	0.297711	0.000418	0.297469	0.110369	0.005409	0.00001	0.00001	0.586315
18341	0.017192	0.198935	0.720314	0.016185	0.047374	0.381946	0.036825	0.018332	0.184903	0.178665	0.199329	0.026059	0.199458	0.229339	0.01222	0.010992	0.006681	0.515251
18385	0.007576	0.002154	0.917403	0.025676	0.04719	0.686687	0.049603	0.007426	0.135643	0.118549	0.002092	0.026226	0.002126	0.228043	0.022811	0.004676	0.002909	0.713209
18395	0.00981	0.259788	0.708618	0.008543	0.013242	0.416635	0.016033	0.009275	0.067443	0.231609	0.259005	0.007394	0.259502	0.115053	0.007948	0.003834	0.005065	0.601204
18431	0.002114	0.004588	0.94383	0.023378	0.02609	0.580077	0.037823	0.001911	0.115193	0.260333	0.004662	0.01564	0.004614	0.180258	0.021458	0.001563	0.000536	0.775931
18469	0.020806	0.274027	0.679502	0.00733	0.018336	0.422069	0.019198	0.020442	0.065702	0.198938	0.273651	0.012262	0.274002	0.116222	0.006517	0.008604	0.01098	0.571414
18523	0.001991	0.001812	0.933157	0.021554	0.041486	0.703021	0.038894	0.00191	0.136499	0.117933	0.001745	0.020235	0.00178	0.217017	0.019111	0.001446	0.000483	0.739929
18528	0.015649	0.317389	0.653481	0.004079	0.009402	0.422283	0.00746	0.015304	0.059005	0.179401	0.316548	0.004148	0.31712	0.10076	0.003504	0.004918	0.009435	0.560115
18529	0.000653	0.351412	0.634216	0.006718	0.007001	0.437737	0.006694	0.000611	0.066673	0.137951	0.350334	0.001522	0.350275	0.11372	0.005412	0.000064	0.00055	0.528458
18557	0.020554	0.11921	0.817212	0.015689	0.027335	0.458934	0.038825	0.019504	0.059274	0.304685	0.118778	0.02407	0.119186	0.097725	0.015483	0.011887	0.007514	0.724134
18586	0.024788	0.250744	0.695865	0.011832	0.016771	0.435324	0.024603	0.023694	0.050883	0.215098	0.250399	0.013203	0.250769	0.091832	0.01157	0.012102	0.011183	0.609341
18599	0.005627	0.003392	0.918984	0.024351	0.047646	0.668706	0.045906	0.005863	0.156973	0.119236	0.003316	0.024662	0.00335	0.247005	0.020772	0.003696	0.002048	0.698467
18672	0.004649	0.005586	0.891954	0.033239	0.064572	0.547977	0.059371	0.005184	0.270687	0.111177	0.005605	0.032008	0.005572	0.359241	0.026618	0.003761	0.001247	0.571552
18713	0.009762	0.324875	0.6471	0.006915	0.011348	0.392945	0.007055	0.009483	0.099408	0.166696	0.324412	0.001285	0.32479	0.153497	0.004738	0.003327	0.005796	0.506568
18715	0.004223	0.003111	0.91638	0.026087	0.050198	0.571895	0.042652	0.00429	0.199815	0.178284	0.003064	0.021241	0.003074	0.273133	0.021799	0.002813	0.001525	0.676415
18769	0.025635	0.284743	0.666987	0.007658	0.014978	0.393351	0.019562	0.024532	0.04211	0.236366	0.28408	0.012428	0.284574	0.076082	0.007505	0.013692	0.010503	0.595217
18778	0.003052	0.605246	0.3911	0.000044	0.000559	0.279584	0.000037	0.00268	0.034104	0.080029	0.603566	0.000027	0.603469	0.068395	0.000014	0.00001	0.002825	0.32526
18789	0.015824	0.115618	0.817643	0.022282	0.028634	0.488291	0.035792	0.015493	0.14031	0.204604	0.115511	0.015323	0.1157	0.204222	0.019186	0.009723	0.005897	0.62995
18818	0.02045	0.178159	0.765167	0.015361	0.020863	0.480704	0.028914	0.019636	0.076059	0.216635	0.178053	0.013874	0.178303	0.131775	0.014299	0.011722	0.007839	0.642189
18840	0.0056	0.003393	0.91915	0.025792	0.046065	0.566658	0.044975	0.005648	0.167682	0.211691	0.003346	0.023236	0.00335	0.231406	0.022514	0.003083	0.002498	0.713913
18841	0.00195	0.003162	0.943974	0.020963	0.029951	0.518257	0.038152	0.00177	0.127328	0.311334	0.003159	0.018583	0.003217	0.186785	0.018919	0.00103	0.000887	0.77058
18866	0.022979	0.240592	0.707095	0.009515	0.019819	0.410203	0.021486	0.022604	0.08125	0.224504	0.239954	0.013003	0.240408	0.124285	0.008481	0.010269	0.011625	0.591929
18892	0.019212	0.123273	0.810458	0.018053	0.029004	0.496898	0.038089	0.018888	0.089572	0.23343	0.123123	0.021309	0.12323	0.142354	0.016741	0.010202	0.008344	0.67782
18917	0.024645	0.125474	0.809869	0.016676	0.023336	0.466977	0.03605	0.023324	0.058071	0.290404	0.125174	0.02014	0.125507	0.09636	0.016321	0.014592	0.008871	0.718208
18929	0.014925	0.176204	0.75323	0.021191	0.03445	0.473121	0.03673	0.01524	0.144437	0.154146	0.176326	0.018443	0.176493	0.207285	0.017975	0.008761	0.006107	0.564937
18938	0.01021	0.102093	0.85178	0.015559	0.020358	0.332755	0.023825	0.008367	0.148354	0.385445	0.101254	0.009297	0.10203	0.200558	0.012472	0.005669	0.004013	0.665962
18972	0.006102	0.318832	0.650135	0.010154	0.014777	0.45715	0.012487	0.006142	0.101884	0.103827	0.318509	0.003749	0.318673	0.162985	0.007807	0.000394	0.005215	0.501178
18974	0.018999	0.580988	0.399292	0.000122	0.000599	0.272876	0.000148	0.018054	0.041715	0.087561	0.579647	0.000118	0.580875	0.075094	0.00001	0.004094	0.012133	0.327675
19012	0.02593	0.223444	0.719838	0.008915	0.021874	0.438951	0.022821	0.025605	0.077796	0.211549	0.223278	0.01447	0.223579	0.131066	0.007835	0.01207	0.012742	0.598239
19144	0.00001	0.362842	0.631462	0.003223	0.002462	0.397149	0.002673	0.00001	0.047491	0.191548	0.361129	0.000663	0.361207	0.087399	0.002452	0.000023	0.00001	0.548245
19154	0.013532	0.246455	0.693826	0.015993	0.030195	0.450257	0.028881	0.014107	0.133321	0.126868	0.246565	0.014788	0.246974	0.204883	0.012671	0.005874	0.007081	0.507728
19165	0.00001	0.000594	0.96152	0.023981	0.013895	0.390921	0.016286	0.00001	0.214285	0.377982	0.000516	0.000764	0.000601	0.286051	0.01635	0.00001	0.00001	0.696213
19173	0.001237	0.001986	0.96229	0.017147	0.01734	0.341261	0.02531	0.000724	0.131031	0.499844	0.00183	0.010848	0.00192	0.170615	0.01482	0.001132	0.000196	0.800469
19181	0.005918	0.00275	0.915829	0.024173	0.051331	0.663579	0.047695	0.006205	0.161176	0.118672	0.002672	0.026909	0.002718	0.250753	0.020504	0.004303	0.001809	0.693006
19207	0.015019	0.025959	0.885019	0.025055	0.048948	0.555659	0.056212	0.01559	0.14483	0.201508	0.0262	0.033746	0.026023	0.221006	0.021874	0.010468	0.004841	0.682042
19215	0.002009	0.001489	0.960937	0.020349	0.015217	0.379703	0.023911	0.001485	0.138689	0.454801	0.00141	0.00656	0.001473	0.185799	0.017586	0.001451	0.000556	0.786576
19237	0.000251	0.405818	0.587542	0.002094	0.004295	0.39239	0.000692	0.000246	0.08256	0.119753	0.404358	0.000246	0.404151	0.140362	0.000344	0.000208	0.000021	0.454668
19348	0.006425	0.001847	0.919163	0.02442	0.048146	0.688779	0.048449	0.00659	0.141557	0.112864	0.001761	0.026568	0.001812	0.234804	0.021287	0.004068	0.002407	0.709054
19359	0.007209	0.014114	0.946039	0.016941	0.015697	0.389168	0.0235	0.005652	0.133411	0.434901	0.013368	0.007869	0.014043	0.17999	0.014605	0.004081	0.002831	0.776581
19365	0.003704	0.002768	0.924042	0.021985	0.0475	0.663742	0.044369	0.003736	0.146612	0.138842	0.002698	0.025287	0.0027	0.229551	0.019076	0.002752	0.001072	0.719562
19371	0.000219	0.000558	0.974099	0.015444	0.00968	0.432905	0.020017	0.000094	0.09743	0.449036	0.000518	0.006287	0.000544	0.146779	0.014153	0.000025	0.000144	0.832069
19376	0.016573	0.324386	0.626358	0.010871	0.021812	0.392753	0.016797	0.017039	0.128496	0.120735	0.32418	0.007489	0.324818	0.192527	0.007539	0.007786	0.008386	0.451456
19378	0.018956	0.164031	0.768167	0.017293	0.031554	0.442707	0.031872	0.019097	0.147006	0.195302	0.164016	0.016668	0.164264	0.212216	0.013872	0.010252	0.008478	0.57425
19381	0.009028	0.489049	0.49623	0.00012	0.005572	0.327644	0.000067	0.008105	0.090481	0.085552	0.48815	0.000047	0.488902	0.138629	0.00001	0.000565	0.006074	0.365772
19658	0.020187	0.162344	0.775481	0.016347	0.02564	0.48262	0.034136	0.019785	0.081395	0.220051	0.162013	0.018465	0.162347	0.135384	0.015192	0.01342	0.006203	0.648989
19674	0.003301	0.002239	0.934574	0.021608	0.038278	0.528088	0.04317	0.00332	0.152812	0.270351	0.002259	0.023044	0.002258	0.223855	0.018482	0.002272	0.001223	0.728867
19679	0.004672	0.001821	0.904853	0.020607	0.068046	0.575939	0.050099	0.005263	0.208943	0.157968	0.001788	0.035272	0.001779	0.279595	0.016309	0.003219	0.001797	0.662029
19750	0.007882	0.007375	0.919579	0.027003	0.038162	0.628141	0.044758	0.008216	0.160156	0.151236	0.007493	0.020264	0.007341	0.248808	0.023182	0.006463	0.001882	0.69206
1X0830	0.055841	0.147851	0.740796	0.024076	0.031435	0.471824	0.042219	0.055629	0.113208	0.169267	0.147853	0.01953	0.148287	0.17253	0.021897	0.032944	0.022713	0.582099
1X1155	0.010583	0.005581	0.934441	0.019872	0.029524	0.418111	0.046703	0.00888	0.070953	0.450021	0.005333	0.026325	0.005482	0.103471	0.020027	0.007736	0.002647	0.834312
1X1693	0.057505	0.146817	0.742087	0.023037	0.030553	0.476823	0.040609	0.057312	0.110357	0.168077	0.146821	0.018874	0.147228	0.169741	0.020916	0.034625	0.022792	0.585825
1X2124	0.004421	0.002284	0.847818	0.024878	0.120599	0.300017	0.059645	0.005594	0.436843	0.19563	0.002272	0.050163	0.002296	0.469778	0.015773	0.003815	0.001816	0.456358
1X2231	0.000044	0.00001	0.999189	0.000682	0.000075	0.146503	0.000018	0.000026	0.030744	0.822699	0.00001	0.000747	0.00001	0.000242	0.014149	0.000084	0.000027	0.984741
1X2816	0.013159	0.004045	0.931203	0.022531	0.029063	0.49608	0.049266	0.011111	0.059438	0.380216	0.00389	0.02682	0.003953	0.089872	0.022916	0.009272	0.003067	0.844099
1X2892	0.054323	0.149951	0.740989	0.023966	0.030772	0.476117	0.041869	0.054096	0.11138	0.166619	0.149919	0.019186	0.150327	0.17168	0.02178	0.032285	0.021903	0.582839
1X3656	0.007775	0.004102	0.919999	0.031182	0.036943	0.642625	0.051167	0.007951	0.138906	0.155221	0.004129	0.022317	0.004086	0.222983	0.027877	0.005359	0.002585	0.714793
1X3697	0.009729	0.002542	0.91236	0.030825	0.044544	0.635458	0.044617	0.010392	0.223878	0.083125	0.002531	0.017394	0.002436	0.340042	0.024293	0.006433	0.003454	0.605949
1X3796	0.016183	0.412147	0.546785	0.007877	0.017008	0.360676	0.01068	0.016579	0.107329	0.092834	0.411902	0.004425	0.412673	0.163616	0.005051	0.005762	0.009625	0.398849
1X3822	0.009237	0.003475	0.919899	0.023509	0.04388	0.525957	0.040857	0.009497	0.203321	0.216924	0.003445	0.019896	0.003385	0.299208	0.018108	0.006936	0.002652	0.649816
1X3837	0.00001	0.428372	0.571574	0.000033	0.00001	0.458365	0.000013	0.00001	0.074835	0.040352	0.426424	0.000018	0.425583	0.152149	0.000017	0.00001	0.00001	0.422214
1X4080	0.00849	0.008172	0.926261	0.026056	0.031021	0.525205	0.047285	0.007726	0.102598	0.309083	0.008104	0.02226	0.00811	0.163723	0.024399	0.005611	0.002679	0.773217
1X4179	0.009949	0.00645	0.917228	0.032049	0.034323	0.637281	0.048477	0.010005	0.145062	0.15266	0.006515	0.018976	0.006439	0.229553	0.028542	0.007027	0.00331	0.706152
1X4209	0.00748	0.409135	0.561192	0.01076	0.011432	0.348947	0.000949	0.00713	0.180063	0.053388	0.409524	0.000095	0.409746	0.252355	0.003498	0.000172	0.00521	0.328924
1X4696	0.005513	0.003601	0.919928	0.027738	0.043219	0.60537	0.035559	0.005542	0.252936	0.097148	0.003444	0.010953	0.003273	0.378992	0.020744	0.002939	0.001915	0.581184
1X4739	0.000422	0.000394	0.933287	0.023369	0.042529	0.51899	0.029126	0.00028	0.255543	0.195695	0.000366	0.008493	0.000356	0.37395	0.015519	0.000198	0.000051	0.601434

K=5

ID	kindae/P. ursin	P. cynocephalus	P. anubis	P. papio	P. hamadryas
1X4777	0.005274	0.003137	0.954165	0.018082	0.019343
1X4859	0.00208	0.000866	0.91733	0.027091	0.052633
20117	0.014249	0.023097	0.916373	0.016196	0.030085
25347	0.019244	0.057812	0.862849	0.018422	0.041674
25354	0.007818	0.003389	0.915079	0.022543	0.051171
25355	0.006639	0.587812	0.404947	0.000054	0.000547
25409	0.017216	0.296546	0.654595	0.00913	0.022514
25593	0.021524	0.198647	0.743035	0.019383	0.017411
26101	0.00558	0.007396	0.920227	0.030221	0.036575
26138	0.020244	0.274434	0.679999	0.009406	0.015917
26196	0.01692	0.085522	0.820712	0.025408	0.051437
26198	0.001428	0.001611	0.936475	0.017351	0.043135
26310	0.012438	0.022137	0.919407	0.017288	0.028731
26316	0.015096	0.022751	0.910901	0.021544	0.029707
26318	0.014016	0.022367	0.912513	0.018872	0.032231
26324	0.016535	0.208438	0.740301	0.01734	0.017386
26355	0.016548	0.207207	0.747048	0.009401	0.019796
26498	0.019778	0.189781	0.75517	0.013017	0.022254
26800	0.001932	0.001666	0.967608	0.012339	0.016456
26980	0.012678	0.085101	0.879654	0.009116	0.01345
26988	0.008039	0.002861	0.911567	0.024015	0.053517
27033	0.00959	0.340261	0.630012	0.008353	0.011783
27036	0.004608	0.004275	0.934691	0.021766	0.034661
27166	0.007212	0.004122	0.945832	0.019964	0.02287
27181	0.005468	0.006254	0.925737	0.026211	0.03633
27190	0.009811	0.337666	0.640478	0.000728	0.011317
27193	0.013237	0.332677	0.634205	0.005017	0.014864
27199	0.003144	0.002394	0.923276	0.020352	0.050834
27230	0.008543	0.06806	0.878184	0.023115	0.022097
27237	0.012513	0.252617	0.72062	0.008238	0.006012
27267	0.006678	0.309396	0.683436	0.000129	0.000362
27306	0.004789	0.004176	0.911546	0.027708	0.051781
27351	0.01914	0.115106	0.844893	0.007952	0.012909
27358	0.015003	0.112637	0.856555	0.011152	0.004654
27433	0.022053	0.299441	0.65257	0.007907	0.018028
27472	0.007864	0.005004	0.930235	0.02568	0.031217
27503	0.004966	0.001162	0.928988	0.020115	0.04477
27525	0.014235	0.045855	0.884446	0.022943	0.032521
27592	0.018749	0.050365	0.878535	0.023253	0.029098
27594	0.013595	0.089448	0.874246	0.008936	0.013776
27622	0.019234	0.176277	0.762185	0.018076	0.024227
27643	0.019498	0.294182	0.657634	0.007699	0.020987
27666	0.017037	0.169816	0.798619	0.00572	0.008807
27668	0.011942	0.451943	0.526171	0.002262	0.007682
27686	0.014386	0.18816	0.768325	0.008727	0.020402
27742	0.012468	0.493113	0.489202	0.000593	0.004625
27811	0.012518	0.169335	0.799936	0.006379	0.011832
27814	0.015001	0.269853	0.688341	0.0081	0.018706
27820	0.025147	0.116563	0.818773	0.015314	0.024203
27827	0.012516	0.017949	0.924067	0.01749	0.027978
27841	0.012283	0.540093	0.446271	0.000304	0.001049
27844	0.002847	0.002351	0.959175	0.017137	0.01849
27874	0.003974	0.002335	0.954394	0.018836	0.020462
27885	0.005154	0.493449	0.497165	0.000491	0.003741
27889	0.005867	0.002512	0.9475	0.022065	0.022057
27908	0.004685	0.500011	0.490921	0.000558	0.003826
27985	0.009847	0.344601	0.626511	0.002497	0.016545
27989	0.005339	0.002819	0.921157	0.026547	0.044138
27999	0.004126	0.00232	0.924337	0.025431	0.043787
28003	0.009981	0.077742	0.733204	0.162093	0.01698
28021	0.022465	0.109303	0.829338	0.02043	0.018463
28037	0.000043	0.411686	0.580787	0.001304	0.00618
28074	0.011145	0.218961	0.736516	0.01412	0.019258
28075	0.000059	0.31958	0.635592	0.007754	0.037015
28076	0.016996	0.095229	0.840441	0.019467	0.027867
28102	0.004683	0.00421	0.937772	0.022487	0.030848
28183	0.008485	0.005562	0.902464	0.029111	0.054378
28212	0.000647	0.000889	0.975767	0.011467	0.01123
28246	0.006718	0.00221	0.902766	0.027656	0.060651
28259	0.0174	0.219538	0.726887	0.015159	0.021016
28269	0.020862	0.200133	0.746427	0.015961	0.016617
28274	0.014003	0.213576	0.725373	0.01846	0.028588
28279	0.021333	0.1285	0.82802	0.008936	0.013211
28281	0.000091	0.488536	0.510148	0.000416	0.000809
28285	0.019081	0.098189	0.834105	0.023161	0.025464
28304	0.012187	0.006864	0.921195	0.025037	0.034717
28328	0.007012	0.006214	0.925048	0.024905	0.036821
28368	0.011284	0.005764	0.785209	0.022584	0.17516
28375	0.000119	0.347074	0.633275	0.00654	0.012991
28376	0.012428	0.139327	0.798786	0.019171	0.030288
28385	0.010367	0.017855	0.916991	0.019984	0.034803
28386	0.024347	0.088189	0.851036	0.013164	0.023263
28400	0.002485	0.001448	0.970496	0.010939	0.014632
28416	0.001148	0.001211	0.975142	0.01178	0.010719
28417	0.005149	0.002534	0.920097	0.026178	0.046043
28428	0.011385	0.015525	0.925619	0.01598	0.031491
28431	0.019803	0.231324	0.718807	0.011956	0.018111
28436	0.003929	0.001767	0.920525	0.025915	0.047864
28438	0.002585	0.001403	0.925495	0.025596	0.044921
28478	0.010097	0.272737	0.696712	0.004726	0.015728
28495	0.000138	0.381071	0.577195	0.003744	0.037852
28497	0.010914	0.020699	0.912276	0.020961	0.035151
28576	0.008393	0.00595	0.930149	0.024696	0.030813
28591	0.000806	0.001484	0.976908	0.013483	0.007319
28609	0.011973	0.019386	0.920511	0.019792	0.028338
28618	0.000938	0.001079	0.97696	0.014103	0.006921
28635	0.010283	0.110231	0.818802	0.019119	0.041564
28729	0.002369	0.001875	0.93603	0.023044	0.036682
28740	0.014583	0.184735	0.749278	0.019961	0.031443
28742	0.015019	0.02699	0.903925	0.019171	0.034894
28779	0.010614	0.028666	0.895091	0.028676	0.036953
28799	0.001311	0.001022	0.928805	0.024743	0.044118
28801	0.007618	0.004707	0.932917	0.024086	0.030671
28802	0.016449	0.046281	0.895106	0.013899	0.028265
28806	0.00608	0.007212	0.925565	0.024551	0.036592
28830	0.004963	0.003665	0.943008	0.02429	0.024074
28925	0.006692	0.006996	0.928606	0.026909	0.030797
28949	0.02302	0.111574	0.82036	0.020586	0.024459
29156	0.023543	0.086692	0.847898	0.015092	0.026775
29333	0.019919	0.098856	0.839369	0.016972	0.024884
29368	0.007413	0.005802	0.937813	0.02317	0.025802
29565	0.015698	0.258344	0.694682	0.011046	0.020231
29758	0.022295	0.09259	0.834897	0.02263	0.027587
30008	0.00128	0.003246	0.84363	0.13999	0.011854
30033	0.018414	0.095715	0.852199	0.01535	0.018323
30104	0.019766	0.146188	0.785557	0.022106	0.026382
30320	0.021855	0.100718	0.780909	0.022328	0.07419
30366	0.008554	0.009259	0.927002	0.024111	0.031074
30373	0.013099	0.055083	0.845224	0.024219	0.062375
30424	0.017433	0.067584	0.874428	0.021908	0.018647
30508	0.020013	0.160597	0.790218	0.01584	0.013332
30564	0.002852	0.216333	0.745847	0.016853	0.018115
30609	0.007759	0.248145	0.715688	0.009629	0.018779
30611	0.007654	0.065791	0.867585	0.02337	0.0356
30614	0.005204	0.13198	0.834332	0.009911	0.018573
30636	0.002484	0.003153	0.937585	0.026747	0.030032
30650	0.002566	0.003434	0.956498	0.017128	0.020375
30660	0.012053	0.120034	0.821004	0.019002	0.027907
30662	0.001334	0.002244	0.957507	0.017743	0.021172
30673	0.019639	0.110912	0.823474	0.019767	0.026207
30699	0.001887	0.368607	0.614453	0.009076	0.005977
30749	0.018004	0.107486	0.810262	0.021776	0.042472
30751	0.005818	0.131298	0.817717	0.016	0.029167
30752	0.004873	0.003671	0.920664	0.028093	0.042699
30772	0.016312	0.439368	0.538399	0.002931	0.00299
30871	0.005216	0.005171	0.939118	0.025612	0.024883
30878	0.011896	0.028015	0.903569	0.024211	0.03231
30886	0.015804	0.220032	0.72877	0.017853	0.017541
30889	0.009164	0.078385	0.845268	0.020428	0.046755
30907	0.000819	0.210514	0.763837	0.011004	0.013827
30917	0.022381	0.108081	0.814748	0.021081	0.033708
30920	0.022134	0.234707	0.702769	0.014645	0.025746
30927	0.008782	0.083777	0.852724	0.026207	0.028511
30933	0.018668	0.264077	0.690086	0.009951	0.017218
30934	0.006986	0.004605	0.921653	0.027235	0.039521
30961	0.005907	0.003129	0.931894	0.020897	0.038172
30973	0.003371	0.001955	0.941108	0.02026	0.033306
30974	0.018783	0.23806	0.725705	0.009426	0.008027
31011	0.003977	0.226004	0.741786	0.013273	0.01496
31015	0.023828	0.136179	0.722101	0.020527	0.097365
31016	0.00606	0.006264	0.912514	0.027441	0.047721
31021	0.009246	0.13417	0.824741	0.010243	0.0216
31025	0.01253	0.228174	0.646389	0.01308	0.099826
31026	0.003676	0.004482	0.93176	0.023155	0.036928
31028	0.000479	0.302922	0.67129	0.007842	0.017467

K=6

ID	SNPRC P. anubis	amadryas/P. pa	kindae/P. ursin	P. anubis	NPRC P. anubis	P. cynocephalus
1X4777	0.39353	0.034528	0.003892	0.075672	0.489413	0.002965
1X4859	0.607323	0.038424	0.001932	0.281324	0.070172	0.000823
20117	0.607424	0.040687	0.014011	0.06547	0.24892	0.023488
25347	0.534331	0.052609	0.019477	0.075167	0.260521	0.057895
25354	0.617677	0.04616	0.008069	0.156375	0.168405	0.003314
25355	0.298563	0.00001	0.006028	0.034623	0.074491	0.586285
25409	0.40006	0.015482	0.017618	0.133032	0.137373	0.296434
25593	0.498518	0.022212	0.021138	0.123488	0.136159	0.198484
26101	0.609251	0.043519	0.005714	0.188526	0.145552	0.007438
26138	0.473862	0.012578	0.020235	0.10043	0.118557	0.274338
26196	0.483088	0.051061	0.017771	0.184686	0.177586	0.085809
26198	0.714223	0.037147	0.001396	0.132806	0.112864	0.001565
26310	0.619706	0.04092	0.011948	0.064545	0.240513	0.022369
26316	0.579474	0.045864	0.014492	0.069145	0.267889	0.023136
26318	0.602687	0.046345	0.013538	0.062238	0.252461	0.022731
26324	0.510891	0.021728	0.016273	0.110452	0.132457	0.208199
26355	0.427784	0.019376	0.016154	0.097046	0.232795	0.206844
26498	0.266124	0.027955	0.018087	0.097585	0.401654	0.188595
26800	0.300061	0.028814	0.000973	0.06094	0.607751	0.00146
26980	0.231308	0.028476	0.008656	0.028997	0.618337	0.084226
26988	0.634599	0.050678	0.008389	0.161951	0.141602	0.002781
27033	0.434836	0.006894	0.009622	0.104037	0.104678	0.339933
27036	0.516622	0.043243	0.004331	0.130097	0.301341	0.004366
27166	0.600359	0.038843	0.006466	0.063685	0.286546	0.004101
27181	0.515858	0.046138	0.005282	0.144049	0.282399	0.006274
27190	0.380133	0.003824	0.009817	0.07692	0.192261	0.337045
27193	0.378533	0.008298	0.013423	0.098732	0.16883	0.332185
27199	0.673711	0.043462	0.003185	0.158229	0.119075	0.002338
27230	0.487588	0.027692	0.007784	0.175207	0.233936	0.067792
27237	0.548653	0.009106	0.011823	0.051168	0.126934	0.252316
27267	0.215063	0.000988	0.00332	0.017157	0.453975	0.309496
27306	0.614894	0.042402	0.005065	0.261753	0.071827	0.004059
27351	0.253616	0.023283	0.016091	0.039419	0.55347	0.11412
27358	0.433213	0.018096	0.012524	0.030401	0.393761	0.112004
27433	0.417332	0.014006	0.022204	0.104166	0.143163	0.299129
27472	0.494995	0.046889	0.006994	0.1095	0.336679	0.004942
27503	0.717752	0.043466	0.004517	0.126159	0.107013	0.001093
27525	0.519009	0.044417	0.01385	0.113345	0.263394	0.045985
27592	0.473086	0.042774	0.017773	0.107752	0.308604	0.050011
27594	0.333582	0.025106	0.010986	0.040481	0.501194	0.088651
27622	0.50308	0.027093	0.019308	0.126903	0.147335	0.176281
27643	0.410882	0.015239	0.019815	0.109605	0.150493	0.293965
27666	0.273577	0.014699	0.01469	0.047745	0.480508	0.168781
27668	0.362056	0.000712	0.011831	0.083695	0.090443	0.451264
27686	0.432298	0.018965	0.01406	0.099255	0.247576	0.187846
27742	0.3377	0.000444	0.011758	0.074297	0.083663	0.492138
27811	0.270865	0.015926	0.009594	0.061133	0.473383	0.169098
27814	0.392407	0.01583	0.014826	0.101129	0.206432	0.269376
27820	0.458703	0.036278	0.023776	0.057357	0.307672	0.116214
27827	0.64062	0.041045	0.012047	0.059622	0.228507	0.018159
27841	0.298425	0.000092	0.010852	0.059809	0.09185	0.538972
27844	0.59968	0.032801	0.00235	0.055224	0.307661	0.002283
27874	0.595172	0.035625	0.003418	0.060206	0.303327	0.002253
27885	0.311669	0.000264	0.004439	0.063981	0.127434	0.492213
27889	0.609597	0.039893	0.005117	0.064823	0.278123	0.002446
27908	0.306322	0.000289	0.004017	0.063303	0.127304	0.498764
27985	0.364156	0.005718	0.010281	0.109063	0.166476	0.344306
27989	0.590046	0.040375	0.0057	0.222512	0.138581	0.002786
27999	0.563677	0.034228	0.003851	0.252394	0.143621	0.00223
28003	0.526049	0.178704	0.00387	0.086769	0.128409	0.0762
28021	0.478454	0.034775	0.021036	0.068349	0.288739	0.108647
28037	0.411906	0.000294	0.000042	0.096131	0.080999	0.410628
28074	0.474324	0.019782	0.010844	0.113823	0.162433	0.218794
28075	0.365055	0.008283	0.000068	0.219469	0.085069	0.322056
28076	0.469043	0.039407	0.016127	0.090636	0.289801	0.094986
28102	0.523364	0.040798	0.004334	0.125721	0.301469	0.004314
28183	0.615324	0.044759	0.008877	0.269419	0.056352	0.00527
28212	0.31086	0.0249	0.000225	0.043912	0.619356	0.000747
28246	0.623805	0.057878	0.00727	0.172731	0.136137	0.00218
28259	0.438573	0.023019	0.017121	0.118027	0.184001	0.219259
28269	0.506026	0.018389	0.020507	0.118176	0.136858	0.200044
28274	0.487893	0.026458	0.014436	0.151828	0.105621	0.213764
28279	0.268965	0.025153	0.018255	0.037349	0.523164	0.127113
28281	0.323873	0.000224	0.000079	0.051562	0.137893	0.486369
28285	0.556455	0.034556	0.01863	0.123522	0.168515	0.098322
28304	0.483737	0.049122	0.011422	0.111008	0.337938	0.006772
28328	0.520401	0.045733	0.006869	0.144468	0.27628	0.006248
28368	0.504415	0.17944	0.017076	0.132567	0.160596	0.005906
28375	0.429698	0.00409	0.00012	0.116631	0.102401	0.34706
28376	0.515587	0.034616	0.012657	0.127765	0.170029	0.139347
28385	0.590902	0.049117	0.009975	0.069551	0.262351	0.018105
28386	0.592383	0.033032	0.023861	0.049374	0.213145	0.088204
28400	0.290757	0.026938	0.000921	0.056167	0.623945	0.001272
28416	0.269712	0.026436	0.000342	0.040107	0.662386	0.001018
28417	0.590588	0.040237	0.005465	0.23352	0.127711	0.002479
28428	0.626527	0.041638	0.011139	0.066563	0.238227	0.015906
28431	0.49831	0.017617	0.019614	0.100455	0.132635	0.231369
28436	0.579724	0.037011	0.003769	0.262241	0.115555	0.0017
28438	0.563687	0.034452	0.00242	0.258897	0.139204	0.00134
28478	0.411979	0.010945	0.010125	0.090007	0.204698	0.272246
28495	0.38021	0.032197	0.000153	0.068617	0.136973	0.38185
28497	0.589516	0.051001	0.010638	0.066814	0.261159	0.020873
28576	0.494389	0.044652	0.007686	0.115113	0.332323	0.005838
28591	0.307544	0.022632	0.000252	0.046009	0.622362	0.001201
28609	0.627983	0.042635	0.011535	0.067789	0.230454	0.019604
28618	0.295482	0.022816	0.000212	0.043891	0.636738	0.000862
28635	0.505546	0.028765	0.010735	0.22928	0.114558	0.111115
28729	0.511453	0.031044	0.00219	0.216797	0.236678	0.001837
28740	0.489393	0.034434	0.014925	0.13635	0.140057	0.184842
28742	0.569506	0.048708	0.014606	0.067077	0.27283	0.027274
28779	0.608904	0.040107	0.010797	0.194701	0.116577	0.028915
28799	0.571168	0.033438	0.001247	0.252855	0.14029	0.001002
28801	0.494857	0.046026	0.007006	0.09947	0.347989	0.004651
28802	0.62714	0.036605	0.016012	0.061614	0.211812	0.046817
28806	0.605328	0.037362	0.006394	0.18811	0.155494	0.007312
28830	0.680215	0.036589	0.00479	0.104125	0.170591	0.003691
28925	0.489993	0.047727	0.006071	0.108353	0.340948	0.006909
28949	0.557709	0.033167	0.022633	0.105773	0.169022	0.111695
29156	0.571661	0.037653	0.023001	0.053997	0.226893	0.086795
29333	0.52411	0.031366	0.019449	0.096599	0.229711	0.098766
29368	0.502324	0.038365	0.006575	0.113007	0.334021	0.005707
29565	0.450529	0.018243	0.015886	0.107181	0.150076	0.258084
29758	0.521383	0.039991	0.021688	0.099987	0.224453	0.092497
30008	0.426098	0.16065	0.000312	0.03923	0.370762	0.002947
30033	0.550971	0.02577	0.017712	0.082708	0.227248	0.09559
30104	0.530173	0.034405	0.019481	0.11958	0.150152	0.146209
30320	0.476768	0.069481	0.024245	0.18025	0.147799	0.101456
30366	0.532241	0.04439	0.008042	0.108718	0.297379	0.009229
30373	0.523521	0.070794	0.01417	0.127241	0.208815	0.055459
30424	0.5953	0.033766	0.016447	0.074193	0.212587	0.067708
30508	0.569897	0.021889	0.019137	0.071986	0.156696	0.160394
30564	0.471439	0.016559	0.002677	0.150679	0.142325	0.216321
30609	0.421366	0.014614	0.007879	0.114936	0.193512	0.247693
30611	0.593551	0.037514	0.00812	0.165202	0.129408	0.066205
30614	0.283564	0.021938	0.003862	0.093557	0.465958	0.131121
30636	0.672614	0.037615	0.002547	0.151891	0.132081	0.003253
30650	0.42577	0.02672	0.00204	0.117737	0.424354	0.00338
30660	0.46548	0.030386	0.011855	0.145128	0.227292	0.11986
30662	0.431756	0.025772	0.001094	0.131138	0.408083	0.002156
30673	0.525609	0.035287	0.019311	0.099847	0.209146	0.110801
30699	0.415443	0.002244	0.001678	0.113176	0.099531	0.367929
30749	0.469074	0.046221	0.018379	0.137735	0.221213	0.107377
30751	0.575822	0.025987	0.00626	0.13624	0.124019	0.131671
30752	0.538089	0.050088	0.004792	0.168532	0.234849	0.003651
30772	0.352097	0.000114	0.014947	0.099307	0.095079	0.438456
30871	0.657952	0.038017	0.005148	0.111477	0.182197	0.005208
30878	0.54809	0.045719	0.011342	0.103789	0.263095	0.027965
30886	0.514725	0.023228	0.015443	0.10357	0.123105	0.219929
30889	0.479174	0.036681	0.010012	0.205186	0.190335	0.078612
30907	0.516375	0.015656	0.000798	0.08182	0.175411	0.20994
30917	0.402352	0.039309	0.021836	0.140243	0.288445	0.107815
30920	0.441533	0.025256	0.022474	0.122015	0.15413	0.234591
30927	0.565822	0.035084	0.008515	0.166806	0.139724	0.084049
30933	0.397594	0.018242	0.018216	0.081612	0.220876	0.26346
30934	0.540522	0.046627	0.006862	0.163508	0.237905	0.004577
30961	0.643862	0.03973	0.006295	0.142026	0.164873	0.003214
30973	0.631324	0.035986	0.003153	0.114465	0.213147	0.001925
30974	0.527776	0.010592	0.017933	0.065904	0.139956	0.237838
31011	0.445608	0.022032	0.003293	0.071656	0.232165	0.225246
31015	0.459834	0.100536	0.027246	0.112078	0.162981	0.137325
31016	0.575497	0.04615	0.006431	0.217227	0.148432	0.006263
31021	0.258034	0.024468	0.007871	0.096829	0.479158	0.13364
31025	0.418584	0.099927	0.016612	0.075985	0.159725	0.229167
31026	0.501319	0.038136	0.003672	0.17957	0.272832	0.00447
31028	0.413396	0.011986	0.00054	0.107913	0.163525	0.302639

K=7

ID	P. hamadryas	P. cynocephalus	P. anubis	P. papio	P. kindae	P. ursinus	SNPRC P. anubis
1X4777	0.015874	0.0031	0.111144	0.018521	0.003101	0.001772	0.846489
1X4859	0.015051	0.000777	0.418597	0.018738	0.000793	0.000473	0.545571
20117	0.026047	0.023186	0.117335	0.015947	0.0092	0.004505	0.80378
25347	0.035836	0.057694	0.128775	0.017842	0.012427	0.006646	0.740779
25354	0.026884	0.003329	0.236188	0.019461	0.005675	0.002376	0.706086
25355	0.00001	0.586523	0.072172	0.00001	0.00017	0.005433	0.335682
25409	0.008093	0.296876	0.196318	0.005877	0.008016	0.008784	0.476035
25593	0.004323	0.198966	0.192159	0.016553	0.010284	0.010627	0.567088
26101	0.016249	0.007408	0.284039	0.025441	0.003977	0.001821	0.661065
26138	0.004585	0.274743	0.164445	0.007227	0.006475	0.012663	0.529862
26196	0.030424	0.085749	0.247708	0.021239	0.0094	0.0078	0.59768
26198	0.02227	0.001558	0.216042	0.014907	0.001293	0.000198	0.743732
26310	0.024628	0.022098	0.118505	0.017221	0.006847	0.004808	0.805892
26316	0.025571	0.022913	0.11986	0.021375	0.007765	0.006506	0.796009
26318	0.028313	0.022453	0.115728	0.018747	0.00855	0.004841	0.80137
26324	0.005788	0.208679	0.177013	0.014976	0.007462	0.008336	0.577745
26355	0.0103	0.207197	0.149692	0.007816	0.011353	0.00494	0.608703
26498	0.015936	0.189547	0.126165	0.011953	0.008493	0.010192	0.637714
26800	0.016174	0.001587	0.075634	0.013811	0.001423	0.000366	0.891005
26980	0.017829	0.084898	0.028206	0.011101	0.005045	0.005678	0.847243
26988	0.030016	0.002829	0.246512	0.020536	0.005259	0.002977	0.691872
27033	0.000736	0.340504	0.163601	0.005787	0.001595	0.006889	0.480888
27036	0.02291	0.004394	0.186756	0.019628	0.003136	0.001611	0.761566
27166	0.019371	0.004083	0.116158	0.019733	0.004906	0.001812	0.833936
27181	0.021562	0.006316	0.211813	0.023325	0.00345	0.002064	0.731469
27190	0.003148	0.337519	0.125802	0.000252	0.003209	0.005719	0.524351
27193	0.003976	0.332737	0.150479	0.00301	0.007613	0.005343	0.496842
27199	0.026368	0.002344	0.241832	0.017015	0.002299	0.000952	0.70919
27230	0.006734	0.068207	0.259629	0.018267	0.005397	0.002973	0.638792
27237	0.002032	0.252337	0.099715	0.00778	0.003357	0.007747	0.627031
27267	0.002419	0.309004	0.023239	0.000596	0.001935	0.003134	0.659673
27306	0.018213	0.00387	0.390667	0.020204	0.002975	0.001455	0.562616
27351	0.014429	0.114905	0.048841	0.00941	0.010849	0.00697	0.794596
27358	0.005606	0.112737	0.063217	0.012357	0.00479	0.0083	0.792992
27433	0.007122	0.299616	0.164896	0.005744	0.009857	0.011409	0.501356
27472	0.022196	0.005001	0.158941	0.024265	0.005821	0.002084	0.781693
27503	0.025516	0.001141	0.211507	0.01796	0.002964	0.001755	0.739157
27525	0.022756	0.045966	0.170714	0.021142	0.006637	0.007059	0.725725
27592	0.020472	0.050413	0.158591	0.021674	0.011334	0.006998	0.730518
27594	0.015427	0.089264	0.057439	0.010373	0.006045	0.006036	0.815417
27622	0.010317	0.176548	0.199909	0.015208	0.009663	0.009148	0.579207
27643	0.009199	0.294455	0.167391	0.005299	0.007833	0.010834	0.504989
27666	0.008863	0.169488	0.06374	0.006426	0.007699	0.007966	0.735817
27668	0.000201	0.451969	0.132896	0.000541	0.002027	0.008334	0.404031
27686	0.010766	0.188148	0.149906	0.007245	0.009066	0.005018	0.629851
27742	0.00027	0.492225	0.121512	0.000166	0.000204	0.010835	0.374788
27811	0.01009	0.169222	0.080364	0.006737	0.005106	0.005937	0.722544
27814	0.008806	0.269835	0.153427	0.0063	0.005813	0.008393	0.547426
27820	0.021542	0.116569	0.093929	0.015424	0.014904	0.008943	0.728689
27827	0.024456	0.017903	0.112703	0.017652	0.008223	0.003683	0.815379
27841	0.000037	0.539856	0.096919	0.00006	0.002407	0.007076	0.353644
27844	0.015983	0.002284	0.102635	0.01748	0.001794	0.000649	0.859174
27874	0.017153	0.002254	0.10969	0.018977	0.002748	0.000877	0.848301
27885	0.000144	0.492629	0.101356	0.00011	0.000146	0.003694	0.40192
27889	0.018369	0.00245	0.117687	0.021795	0.003046	0.002119	0.834534
27908	0.000095	0.499156	0.100118	0.000171	0.00001	0.003472	0.396978
27985	0.003715	0.344792	0.167083	0.000708	0.003659	0.005739	0.474304
27989	0.016826	0.002709	0.336048	0.020134	0.003093	0.002128	0.619062
27999	0.011478	0.002167	0.374705	0.01848	0.002105	0.001326	0.589739
28003	0.007312	0.077811	0.157713	0.160135	0.005681	0.003945	0.587402
28021	0.014369	0.109144	0.110557	0.020106	0.011895	0.009528	0.724402
28037	0.000034	0.41012	0.15878	0.000238	0.00001	0.000032	0.430786
28074	0.007005	0.219021	0.176041	0.01183	0.00334	0.007158	0.575606
28075	0.006489	0.321713	0.275115	0.002191	0.00001	0.00006	0.394422
28076	0.020597	0.095252	0.140453	0.018287	0.010569	0.005945	0.708896
28102	0.019648	0.004328	0.182649	0.02038	0.00312	0.001645	0.768231
28183	0.020013	0.005031	0.401039	0.021204	0.004523	0.00321	0.54498
28212	0.013039	0.00083	0.050684	0.013856	0.000339	0.000107	0.921145
28246	0.034363	0.002177	0.253153	0.023872	0.004526	0.002471	0.679437
28259	0.00919	0.219677	0.176382	0.012707	0.007231	0.009464	0.565349
28269	0.004049	0.200426	0.185676	0.01334	0.008894	0.011113	0.576502
28274	0.011064	0.214088	0.22005	0.014875	0.005929	0.007628	0.526366
28279	0.01491	0.128161	0.048042	0.010506	0.01227	0.007609	0.778501
28281	0.000093	0.486465	0.084475	0.000126	0.00001	0.000068	0.428764
28285	0.013089	0.098313	0.196984	0.020543	0.008443	0.009994	0.652634
28304	0.025417	0.006883	0.159794	0.023414	0.008116	0.004015	0.772362
28328	0.022482	0.006292	0.21476	0.021818	0.004879	0.002259	0.72751
28368	0.163318	0.00576	0.171778	0.020293	0.007827	0.003712	0.627313
28375	0.000452	0.346864	0.17821	0.003185	0.00001	0.000077	0.471203
28376	0.01714	0.139321	0.200173	0.01633	0.006652	0.005561	0.614823
28385	0.030212	0.017827	0.120101	0.019823	0.007757	0.002291	0.80199
28386	0.020672	0.088011	0.09766	0.013458	0.014504	0.009072	0.756623
28400	0.015415	0.001397	0.067521	0.012676	0.001054	0.000793	0.901143
28416	0.013363	0.001141	0.044788	0.014762	0.000762	0.000086	0.925098
28417	0.01737	0.0024	0.350239	0.019376	0.003471	0.001579	0.605566
28428	0.026852	0.015621	0.121049	0.015843	0.007245	0.003661	0.809728
28431	0.007177	0.231601	0.165716	0.009847	0.008328	0.010528	0.566802
28436	0.014228	0.001628	0.3877	0.018494	0.001808	0.001387	0.574754
28438	0.011916	0.001284	0.380488	0.018298	0.001276	0.00088	0.585859
28478	0.006423	0.272583	0.14577	0.003176	0.005019	0.004667	0.562361
28495	0.031785	0.380157	0.107516	0.002603	0.000011	0.000114	0.477814
28497	0.030806	0.020674	0.118028	0.021086	0.006932	0.003553	0.798921
28576	0.021038	0.005941	0.167587	0.023094	0.005251	0.003051	0.774038
28591	0.00837	0.001373	0.056913	0.016023	0.000294	0.000255	0.916773
28609	0.023852	0.019271	0.122433	0.019517	0.008722	0.003136	0.803068
28618	0.008639	0.000986	0.050789	0.016876	0.000483	0.000153	0.922074
28635	0.012108	0.111092	0.344396	0.012237	0.00644	0.003601	0.510126
28729	0.010684	0.00182	0.31654	0.017074	0.001514	0.000764	0.651605
28740	0.016459	0.185072	0.209587	0.016648	0.008622	0.005701	0.557911
28742	0.0308	0.027069	0.117598	0.018963	0.008296	0.005995	0.791279
28779	0.013731	0.028665	0.298683	0.023701	0.005549	0.004947	0.624723
28799	0.011364	0.000971	0.376229	0.017526	0.000581	0.000459	0.592869
28801	0.02305	0.004702	0.144098	0.02289	0.005875	0.00182	0.797567
28802	0.024249	0.046295	0.11355	0.013656	0.009491	0.006169	0.78659
28806	0.015423	0.007209	0.288827	0.019613	0.003483	0.002647	0.662798
28830	0.013956	0.003645	0.180746	0.022292	0.003789	0.001143	0.774429
28925	0.021757	0.006996	0.157278	0.025503	0.004911	0.001869	0.781685
28949	0.014363	0.111631	0.172006	0.018602	0.011184	0.011145	0.661069
29156	0.023624	0.086627	0.102742	0.015144	0.012791	0.009768	0.749304
29333	0.015845	0.098943	0.156052	0.01549	0.010236	0.00892	0.694513
29368	0.016368	0.005807	0.163899	0.021655	0.004924	0.002329	0.785019
29565	0.008498	0.258406	0.170033	0.008704	0.006066	0.009031	0.539262
29758	0.018738	0.092644	0.15967	0.020912	0.011383	0.010225	0.686427
30008	0.009988	0.003154	0.081733	0.141124	0.000434	0.000518	0.763049
30033	0.011755	0.095693	0.141309	0.014203	0.007266	0.010026	0.719748
30104	0.013923	0.146394	0.191194	0.019507	0.00749	0.011404	0.610087
30320	0.054172	0.101065	0.23708	0.018272	0.01308	0.009096	0.567235
30366	0.021426	0.009256	0.171548	0.022273	0.006024	0.002549	0.766924
30373	0.050069	0.055134	0.19031	0.021693	0.00778	0.005146	0.669868
30424	0.012801	0.067593	0.130757	0.021265	0.009625	0.006975	0.750984
30508	0.007027	0.160493	0.131523	0.014908	0.008322	0.010476	0.667251
30564	0.001252	0.216707	0.23148	0.012572	0.001723	0.001045	0.535222
30609	0.00568	0.248057	0.180263	0.00689	0.004461	0.003409	0.55124
30611	0.016126	0.065928	0.265194	0.018851	0.005716	0.002316	0.625869
30614	0.011898	0.131548	0.129193	0.009158	0.001611	0.003346	0.713246
30636	0.012998	0.003155	0.255366	0.022494	0.001927	0.000672	0.703388
30650	0.01069	0.003509	0.16901	0.015468	0.001252	0.001217	0.798854
30660	0.01271	0.12019	0.218359	0.0155	0.007439	0.00454	0.621262
30662	0.009652	0.002277	0.184928	0.015676	0.000634	0.00069	0.786144
30673	0.017034	0.110896	0.161112	0.018003	0.010446	0.00866	0.673849
30699	0.00018	0.36756	0.172842	0.005258	0.000134	0.001077	0.45295
30749	0.028273	0.107564	0.187173	0.019215	0.00818	0.00935	0.640244
30751	0.012391	0.131415	0.217986	0.012704	0.001282	0.004351	0.619871
30752	0.025202	0.00365	0.232702	0.024698	0.002822	0.002022	0.708905
30772	0.00001	0.439229	0.151449	0.000353	0.002526	0.010703	0.39573
30871	0.014144	0.005145	0.185448	0.023377	0.003793	0.001448	0.766646
30878	0.023291	0.027948	0.157241	0.022769	0.008115	0.00353	0.757107
30886	0.006603	0.220177	0.169607	0.01559	0.007913	0.007246	0.572863
30889	0.021269	0.078569	0.265621	0.015803	0.006677	0.003239	0.608822
30907	0.005681	0.209898	0.13718	0.009612	0.000395	0.000392	0.636843
30917	0.019622	0.108381	0.198997	0.018177	0.013569	0.008702	0.632554
30920	0.012063	0.235092	0.185527	0.011837	0.011553	0.010181	0.533747
30927	0.01137	0.083997	0.254445	0.021773	0.004048	0.00438	0.619987
30933	0.009485	0.263923	0.122806	0.008706	0.008243	0.009601	0.577236
30934	0.022532	0.004576	0.22961	0.023686	0.004955	0.002164	0.712476
30961	0.021221	0.003148	0.228093	0.017621	0.003923	0.002122	0.723872
30973	0.0178	0.001911	0.178809	0.018673	0.002102	0.001051	0.779655
30974	0.001877	0.238022	0.122325	0.008603	0.00744	0.01002	0.611713
31011	0.009234	0.225503	0.114076	0.012459	0.002505	0.001021	0.635202
31015	0.086712	0.136414	0.167102	0.018381	0.010036	0.012881	0.568474
31016	0.021897	0.006128	0.321554	0.02163	0.00391	0.002179	0.622702
31021	0.014691	0.134034	0.130026	0.009447	0.0046	0.004296	0.702904
31025	0.094187	0.227842	0.116335	0.011974	0.005525	0.006311	0.537825
31026	0.017118	0.00448	0.261867	0.018738	0.002773	0.00113	0.693895
31028	0.004907	0.302827	0.168343	0.005379	0.000358	0.000159	0.518025

ID	K=5					K=6						K=7						
	kindae/P. ursin	. cynocephalu	P. anubis	P. papio	P. hamadryas	SNPRC P. anubis	amadryas/P. pa	kindae/P. ursin	P. anubis	NPRC P. anubis	. cynocephalus	P. hamadryas	. cynocephalu	P. anubis	P. papio	P. kindae	P. ursinus	NPRC P. anubis
31035	0.014726	0.379222	0.58993	0.007259	0.008863	0.408284	0.004611	0.014645	0.09574	0.098021	0.378699	0.000509	0.379386	0.15383	0.004604	0.006837	0.006608	0.448227
31064	0.003882	0.005083	0.935781	0.025154	0.030101	0.615803	0.039161	0.003876	0.136783	0.199195	0.005183	0.016164	0.004924	0.210149	0.022246	0.003819	0.000617	0.742081
31065	0.01832	0.279084	0.674418	0.008638	0.01954	0.386168	0.015603	0.018284	0.105449	0.195843	0.278654	0.0085	0.279191	0.153685	0.006545	0.009884	0.007981	0.534215
31072	0.0046	0.004104	0.941763	0.023891	0.025643	0.6281	0.036706	0.004483	0.11528	0.211297	0.004134	0.015108	0.004105	0.185494	0.021647	0.00242	0.002023	0.769204
31086	0.019136	0.118016	0.808863	0.022738	0.031248	0.497468	0.03615	0.019071	0.147775	0.181488	0.118048	0.015315	0.118251	0.221163	0.019363	0.009337	0.009466	0.607105
31110	0.0059	0.006626	0.929013	0.025105	0.033355	0.535241	0.044708	0.005712	0.130534	0.277157	0.006649	0.021249	0.006651	0.193655	0.02269	0.003791	0.002173	0.749791
31115	0.009695	0.005783	0.857666	0.024915	0.101941	0.569922	0.11712	0.012235	0.097263	0.197522	0.005939	0.094197	0.00577	0.152657	0.023291	0.006735	0.003083	0.714268
31120	0.016758	0.292098	0.669746	0.015009	0.006389	0.479856	0.012698	0.015752	0.080114	0.119963	0.291616	0.00006	0.291994	0.137334	0.01327	0.004847	0.010276	0.542219
31124	0.016554	0.046168	0.881219	0.024553	0.031506	0.509004	0.043564	0.016015	0.122756	0.262541	0.04612	0.020397	0.046261	0.181992	0.022411	0.00953	0.006763	0.712645
31130	0.000612	0.3597	0.628546	0.006353	0.004789	0.425111	0.000905	0.000616	0.109292	0.105526	0.358549	0.00001	0.358004	0.167196	0.002961	0.000339	0.000182	0.471308
31131	0.000856	0.22186	0.75629	0.01147	0.009524	0.490227	0.016414	0.000665	0.056322	0.215697	0.220675	0.00514	0.220924	0.101023	0.011117	0.000532	0.000205	0.661059
31134	0.011557	0.021491	0.930713	0.016562	0.019679	0.468064	0.031199	0.009952	0.087493	0.382027	0.021265	0.015332	0.021537	0.129849	0.016029	0.006565	0.004134	0.806554
31145	0.011185	0.110644	0.820224	0.023025	0.034922	0.556153	0.035949	0.011626	0.161388	0.123848	0.111035	0.014876	0.110962	0.255773	0.018624	0.007435	0.004031	0.5883
31152	0.00468	0.004392	0.919763	0.029921	0.041243	0.530372	0.048963	0.004539	0.179294	0.232469	0.004362	0.022705	0.004368	0.245809	0.025976	0.002977	0.001746	0.69642
31177	0.01549	0.397738	0.577442	0.002619	0.006711	0.382682	0.000321	0.015107	0.092514	0.112238	0.397138	0.000051	0.397829	0.149823	0.000388	0.004564	0.008998	0.438347
31178	0.020211	0.262088	0.696816	0.009871	0.011014	0.419427	0.013795	0.019508	0.078149	0.207792	0.261329	0.004264	0.261936	0.124644	0.008883	0.007371	0.011635	0.581267
31234	0.012179	0.093294	0.849257	0.018657	0.026613	0.511062	0.03162	0.012053	0.123659	0.228323	0.093283	0.013758	0.093362	0.19491	0.015955	0.009123	0.003409	0.669483
31235	0.003356	0.002971	0.948978	0.021129	0.023566	0.595698	0.037048	0.003095	0.089235	0.271965	0.00296	0.016666	0.002928	0.146923	0.020148	0.003124	0.00035	0.80986
31255	0.014804	0.073832	0.865743	0.023119	0.022502	0.572855	0.03279	0.014245	0.126123	0.180051	0.073936	0.011484	0.073947	0.196524	0.020282	0.008682	0.005603	0.683479
31275	0.006699	0.162236	0.798708	0.01726	0.015097	0.548306	0.024703	0.005899	0.074676	0.184445	0.161971	0.008115	0.16177	0.129965	0.016193	0.005882	0.00078	0.677296
31284	0.014492	0.112399	0.837009	0.013688	0.022412	0.368341	0.029122	0.013025	0.099758	0.378263	0.111492	0.016186	0.112256	0.142369	0.012454	0.007138	0.00639	0.703207
31288	0.018466	0.321127	0.646249	0.006063	0.008095	0.404245	0.006478	0.017874	0.074696	0.176228	0.32048	0.001605	0.321137	0.119517	0.004687	0.007193	0.009943	0.535918
31297	0.018593	0.051	0.884093	0.019531	0.026782	0.428618	0.038991	0.017367	0.096773	0.367905	0.050346	0.019475	0.050968	0.142627	0.018571	0.010393	0.007776	0.750189
31299	0.014845	0.05196	0.890853	0.016099	0.026243	0.371452	0.036203	0.013467	0.096057	0.431743	0.051078	0.020007	0.051843	0.134052	0.015424	0.007776	0.006439	0.76446
31300	0.019447	0.302829	0.653501	0.011527	0.012696	0.454034	0.010537	0.019322	0.113339	0.100226	0.302542	0.000407	0.303198	0.177659	0.008827	0.006409	0.011964	0.491534
31311	0.011236	0.161196	0.785891	0.018927	0.022751	0.500062	0.030243	0.010804	0.106138	0.191721	0.161032	0.012504	0.161128	0.165887	0.016995	0.005559	0.005205	0.632722
31327	0.006332	0.004877	0.932407	0.021676	0.034708	0.498553	0.043861	0.005984	0.123131	0.323578	0.004893	0.023722	0.00493	0.177483	0.019548	0.005148	0.001516	0.767653
31329	0.003277	0.001916	0.937249	0.023067	0.034491	0.655928	0.036765	0.003267	0.136244	0.165885	0.001911	0.015937	0.001893	0.214139	0.020478	0.001695	0.001457	0.744402
31331	0.014326	0.172398	0.780631	0.011383	0.021262	0.293305	0.023061	0.012748	0.113439	0.385888	0.171559	0.012853	0.17229	0.150232	0.009813	0.00675	0.006711	0.641352
31333	0.004474	0.003878	0.933525	0.024405	0.033718	0.602847	0.040881	0.004365	0.130343	0.217684	0.003881	0.018405	0.00385	0.204686	0.021821	0.003076	0.001362	0.746798
31337	0.000463	0.217237	0.737984	0.012734	0.031583	0.424596	0.022899	0.000526	0.15638	0.177886	0.217713	0.013185	0.217523	0.214278	0.009185	0.00001	0.000543	0.545275
31358	0.022467	0.334356	0.625053	0.004101	0.014023	0.372607	0.008626	0.022446	0.083723	0.178807	0.333791	0.005617	0.334484	0.127679	0.002706	0.008392	0.012843	0.508278
31364	0.023504	0.171369	0.764273	0.013941	0.026914	0.447185	0.03396	0.023094	0.076426	0.248352	0.170983	0.02096	0.171316	0.120725	0.013181	0.013229	0.009377	0.651213
31392	0.008235	0.005165	0.925326	0.026675	0.034598	0.553038	0.044036	0.00794	0.144624	0.245224	0.005137	0.019955	0.005131	0.206962	0.023885	0.005365	0.002872	0.73583
31398	0.003061	0.004162	0.926601	0.026786	0.03939	0.623285	0.042292	0.003212	0.167223	0.15976	0.004228	0.01824	0.004165	0.256098	0.022849	0.001485	0.001483	0.69568
31401	0.016083	0.10819	0.835056	0.016158	0.024513	0.599166	0.030156	0.015889	0.083629	0.162925	0.108235	0.015856	0.107909	0.149138	0.014892	0.007724	0.007771	0.696711
31407	0.008765	0.013691	0.915285	0.025758	0.036501	0.537827	0.046344	0.008497	0.138516	0.255148	0.013668	0.023018	0.013668	0.198278	0.023226	0.00558	0.003169	0.733061
31408	0.009291	0.245131	0.703068	0.013354	0.029156	0.409106	0.024993	0.009448	0.126274	0.185086	0.245093	0.014601	0.245271	0.171747	0.01083	0.004556	0.004535	0.54846
31409	0.0198	0.121278	0.820454	0.015223	0.023244	0.453863	0.030578	0.019042	0.090276	0.285349	0.120892	0.015972	0.121216	0.138046	0.014158	0.010588	0.0085	0.69152
31410	0.016582	0.272068	0.682328	0.00829	0.020732	0.409	0.017811	0.016652	0.098877	0.18592	0.27174	0.01011	0.272159	0.156135	0.006496	0.006455	0.009326	0.539319
31415	0.015682	0.097233	0.831101	0.023049	0.032935	0.579446	0.034709	0.01595	0.162578	0.109517	0.0978	0.014207	0.097503	0.25272	0.01885	0.007562	0.007921	0.601237
31417	0.005192	0.162761	0.789107	0.01545	0.02749	0.381645	0.022492	0.004903	0.158653	0.269981	0.162325	0.010212	0.16278	0.207237	0.01196	0.002351	0.002944	0.602516
31440	0.005551	0.005699	0.919082	0.030004	0.039664	0.635403	0.042807	0.005765	0.202662	0.107582	0.00578	0.015783	0.00568	0.31019	0.024484	0.003263	0.002289	0.638311
31457	0.018466	0.192986	0.763893	0.014779	0.009876	0.556591	0.01725	0.017694	0.071676	0.144065	0.192724	0.003124	0.192919	0.132018	0.013848	0.007407	0.009879	0.640805
31462	0.019399	0.120231	0.816949	0.015599	0.027822	0.454324	0.03191	0.019002	0.111581	0.263075	0.120109	0.017175	0.120378	0.167922	0.013826	0.011032	0.007971	0.661697
31469	0.005185	0.003358	0.915811	0.025527	0.050119	0.514659	0.049812	0.005291	0.193801	0.233104	0.003333	0.028524	0.00333	0.252729	0.021476	0.003809	0.001572	0.688559
31471	0.009101	0.152504	0.800607	0.014038	0.02375	0.451123	0.019704	0.009074	0.150224	0.21748	0.152394	0.006285	0.15287	0.230494	0.010358	0.004252	0.004649	0.591092
31475	0.022523	0.238088	0.715314	0.009185	0.014889	0.434344	0.01423	0.022122	0.093516	0.198104	0.237684	0.005817	0.238158	0.144132	0.007705	0.009676	0.011885	0.582627
31477	0.001466	0.373546	0.598425	0.006855	0.019708	0.361507	0.007902	0.00165	0.133638	0.12152	0.373783	0.003397	0.373743	0.188008	0.003394	0.000291	0.001505	0.429662
31482	0.005958	0.010734	0.913286	0.029613	0.040408	0.618726	0.044727	0.006146	0.192482	0.127031	0.010887	0.018301	0.010701	0.292747	0.024307	0.003638	0.002347	0.647958
31484	0.004565	0.004384	0.921243	0.029037	0.04077	0.546863	0.049295	0.004443	0.164057	0.230987	0.004355	0.02345	0.004358	0.229306	0.025735	0.002344	0.002097	0.712711
31490	0.004789	0.007461	0.92608	0.027863	0.033807	0.604537	0.039562	0.004965	0.174426	0.16895	0.00756	0.014427	0.007444	0.268946	0.023337	0.003468	0.00156	0.680818
31497	0.001077	0.253689	0.704789	0.016018	0.024428	0.489677	0.020403	0.001244	0.148429	0.085996	0.254251	0.006056	0.254192	0.227453	0.012187	0.000741	0.000305	0.499067
31498	0.004146	0.16501	0.798241	0.010558	0.022045	0.258259	0.025749	0.003015	0.087608	0.461286	0.164082	0.015723	0.164418	0.119082	0.010007	0.001652	0.002386	0.686732
31499	0.004259	0.005409	0.932834	0.023382	0.034116	0.534188	0.036734	0.00421	0.170303	0.249105	0.005459	0.015865	0.005416	0.253043	0.019122	0.002869	0.001562	0.702125
31500	0.008256	0.007858	0.919999	0.02515	0.038737	0.542606	0.045774	0.008331	0.154219	0.241089	0.007981	0.022591	0.007934	0.228901	0.021665	0.005666	0.002809	0.710435
31506	0.013664	0.254394	0.706846	0.00649	0.018606	0.42129	0.011754	0.013818	0.113369	0.185621	0.254148	0.005958	0.254548	0.179693	0.003835	0.007834	0.005619	0.542513
31507	0.000741	0.225238	0.752952	0.010341	0.010729	0.532021	0.011112	0.000703	0.0855	0.145939	0.224726	0.002039	0.224643	0.143439	0.008694	0.000299	0.000376	0.620511
31511	0.018954	0.116559	0.819735	0.018046	0.026706	0.49503	0.03253	0.018592	0.113418	0.223922	0.116508	0.016024	0.116713	0.171112	0.015991	0.010768	0.007658	0.661735
31515	0.014412	0.058231	0.871872	0.022632	0.032852	0.525065	0.042388	0.013987	0.120145	0.240106	0.05831	0.021811	0.058349	0.177435	0.02049	0.008008	0.005887	0.70802
31518	0.009832	0.032391	0.896499	0.026201	0.035076	0.5471	0.045771	0.009446	0.13423	0.230964	0.032489	0.021551	0.03246	0.200346	0.023554	0.006439	0.003228	0.712422
31525	0.015954	0.258292	0.712404	0.006847	0.006503	0.292892	0.009255	0.013979	0.069245	0.357281	0.257348	0.003186	0.258044	0.098025	0.006424	0.002946	0.011209	0.620165
31526	0.005015	0.004738	0.936229	0.024675	0.029343	0.543479	0.039966	0.004608	0.126439	0.280839	0.00467	0.017569	0.004704	0.182152	0.022582	0.002848	0.001978	0.768166
31528	0.010068	0.014095	0.924364	0.024084	0.027388	0.582842	0.038966	0.009753	0.118626	0.235539	0.014274	0.016565	0.014224	0.186287	0.021689	0.006136	0.003794	0.751305
31540	0.007339	0.006112	0.93473	0.023173	0.028646	0.564344	0.041038	0.006919	0.10912	0.27239	0.006189	0.019072	0.006155	0.171413	0.021358	0.005724	0.001698	0.77458
31542	0.01817	0.106483	0.809308	0.026664	0.039376	0.515736	0.042949	0.018458	0.175759	0.140179	0.106919	0.018521	0.10697	0.263704	0.022087	0.010319	0.007758	0.570641
31543	0.01396	0.333462	0.632829	0.011097	0.008651	0.43998	0.006513	0.013733	0.108419	0.09829	0.333065	0.00008	0.333546	0.169637	0.007922	0.004139	0.008292	0.476384
31552	0.010664	0.015642	0.920969	0.023737	0.028988	0.590075	0.041165	0.010262	0.11156	0.231109	0.015829	0.019002	0.015743	0.179052	0.021742	0.00671	0.003669	0.754083
31553	0.002786	0.003083	0.955384	0.019174	0.019573	0.489009	0.029542	0.002108	0.105549	0.370749	0.003044	0.011488	0.003123	0.15393	0.017972	0.001846	0.000852	0.81079
31556	0.005666	0.003184	0.885373	0.02436	0.081417	0.500746	0.08349	0.006723	0.171034	0.234813	0.003194	0.063301	0.003158	0.215801	0.021114	0.004568	0.001411	0.690646
31559	0.011861	0.016884	0.928509	0.020027	0.022719	0.453607	0.036848	0.010693	0.091481	0.390803	0.016568	0.016772	0.016898	0.134504	0.0193	0.006747	0.0048	0.800978
31560	0.005266	0.223388	0.736582	0.013745	0.021019	0.461023	0.019757	0.00546	0.121354	0.169288	0.223117	0.006811	0.223355	0.19236	0.010666	0.004279	0.001323	0.561206
31573	0.010405	0.053316	0.870719	0.027506	0.038054	0.582408	0.041502	0.010688	0.182991	0.128652	0.05376	0.016495	0.053533	0.280609	0.022523	0.007382	0.003235	0.616224
31576	0.017308	0.04379	0.875899	0.025123	0.037881	0.499847	0.049216	0.01687	0.124221	0.266187	0.043658	0.026513	0.043801	0.173337	0.023133	0.010448	0.006552	0.716217
31578	0.00475	0.002855	0.947776	0.021471	0.023148	0.577188	0.036422	0.004232	0.090617	0.288698	0.002843	0.015981	0.002828	0.145709	0.020407	0.003215	0.001311	0.81055
31602	0.004969	0.152458	0.732614	0.0213	0.088659	0.507765	0.085139	0.008326	0.163278	0.081696	0.153796	0.069942	0.152752	0.247726	0.016962	0.000913	0.003766	0.507939
31605	0.00373	0.002326	0.936628	0.025678	0.031638	0.549589	0.039188	0.003381	0.147293	0.258258	0.00229	0.016442	0.002305	0.206463	0.022984	0.002413	0.001162	0.748231
31624	0.018832	0.059529	0.868108	0.023268	0.030263	0.505682	0.042568	0.018254	0.111318	0.26274	0.059438	0.020475	0.059673	0.168337	0.021383	0.011399	0.007084	0.711649
31630	0.00537	0.005671	0.914457	0.031225	0.043277	0.61592	0.045032	0.005726	0.21939	0.108275	0.005659	0.017164	0.005544	0.329734	0.025163	0.003435	0.002089	0.616871
31642	0.01711	0.119646	0.812155	0.021298	0.02979	0.5823	0.030896	0.017384	0.154656	0.094472	0.120292	0.011651	0.12003	0.244602	0.017232	0.007845	0.008942	0.589698
31650	0.002064	0.003134	0.950447	0.02171	0.022645	0.424756	0.031386	0.001441	0.140498	0.398907	0.003012	0.011989	0.003174	0.192189	0.019058	0.001246	0.000783	0.771561
31656	0.005123	0.251193	0.719497	0.010814	0.013373	0.414696	0.019133	0.004495	0.061877	0.249636	0.250162	0.008971	0.250473	0.09899	0.010267	0.002186	0.00239	0.626721
31659	0.015826	0.056965	0.877652	0.020056	0.0295	0.524789	0.037926	0.01526	0.111129	0.25376	0.057136	0.01976	0.057143	0.164538	0.018292	0.008452	0.006768	0.725048
31666	0.01806	0.370551	0.595306	0.007685	0.008398	0.416381	0.00417	0.017966	0.096976	0.094424	0.370084	0.000044	0.370749	0.155424	0.00496	0.007588	0.009019	0.452215
31693	0.004961	0.00456	0.917786	0.030512	0.042181	0.529509	0.051943	0.004767	0.170023	0.239226	0.004532	0.024657	0.004516	0.234566	0.026927	0.003453	0.001581	0.704301
31716	0.017993	0.180374	0.763745	0.012522	0.025366	0.421207	0.026539	0.01788	0.105174	0.249205	0.179995	0.015142	0.180354	0.157183	0.010836	0.007844	0.009499	0.619142
31720	0.004259	0.130938	0.814232	0.021133	0.029438	0.564704	0.031882	0.004541	0.145668	0.121908	0.131297	0.012407	0.131224	0.236807	0.017351	0.000644	0.003423	0.598144
31729	0.011365	0.345102	0.630702	0.005183	0.007648	0.43025	0.001034	0.011284	0.104305	0.10852	0.344606	0.000082	0.345131	0.166924	0.002157	0.0041	0.00563	0.475975
31731	0.008836	0.006734	0.934398	0.022765	0.027267	0.588517	0.037911	0.008705	0.115831	0.242216	0.006821	0.016599	0.006747	0.18251	0.0206	0.006552	0.002529	0.764463
31738	0.022416	0.164839	0.771078	0.016018	0.025649	0.445082	0.035102	0.021617	0.076315	0.257359	0.164525	0.019733	0.164841	0.121244	0.015271	0.012277	0.009147	0.657488
31764	0.023359	0.104202	0.82705	0.01958	0.025809	0.488805	0.035784	0.022552	0.098925	0.249723	0.104212	0.016711	0.104367	0.154874	0.018025	0.015708	0.00744	0.682875
31778	0.014153	0.133128	0.80837	0.01517	0.02918	0.448289	0.026369	0.014248	0.148467	0.229563	0.133064	0.012297	0.133425	0.224349	0.011638	0.006546	0.007353	0.604392
31786	0.012429	0.097979	0.852902	0.014541	0.022149	0.398234	0.026668	0.011142	0.120483	0.346011	0.097463	0.013306	0.09797	0.172496	0.012451	0.006004	0.005652	0.692121
31789	0.018615	0.30577	0.655341	0.010924	0.00935	0.418539	0.012529	0.017801	0.075664	0.170279	0.305189	0.001854	0.305854	0.123902	0.009608	0.007909	0.009569	0.541304
31800	0.005723	0.226559	0.729307	0.017009	0.021403	0.444457	0.018643	0.005639	0.158121	0.146484	0.226657	0.002771	0.227036	0.237418	0.012544	0.003651	0.002264	0.514316
31804	0.007307	0.010527	0.93288	0.023078	0.026208	0.574881	0.037997	0.006931	0.112613	0.256929	0.010649	0.016206	0.010613	0.178467	0.021129	0.004839	0.002334	0.766411
31805	0.011997	0.185328	0.756597	0.021566	0.024512	0.479638	0.034204	0.011578	0.106218	0.183292	0.18507	0.013967	0.185188	0.163203	0.01962	0.003002	0.008274	0.606745
31836	0.012901	0.159894	0.781632	0.017427	0.028146	0.519417	0.024551	0.013339	0.162213	0.120285	0.160196	0.008664	0.160439	0.251981	0.013173	0.005536	0.007001	0.553205
31846	0.018107	0.238046	0.71323	0.008654	0.021962	0.438497	0.019909	0.018109	0.097354	0.188237	0.237894	0.011936	0.238212	0.156578	0.006988	0.007056	0.010023	0.569207
31849	0.00021	0.000735	0.967691	0.018596	0.012768	0.29966	0.022184	0.000052	0.130796	0.546663	0.000644	0.007088	0.000722	0.162878	0.016449	0.000053	0.000116	0.812695
31852	0.020567	0.300079	0.660443	0.007972	0.010939	0.391485	0.010367	0.020023	0.081279	0.197382	0.299464	0.002955	0.300157	0.124244	0.006789	0.006726	0.012549	0.54658
31880	0.015842	0.1489	0.79549	0.015048	0.024721	0.452996	0.028302	0.015503	0.110202	0.24429	0.148707	0.014113	0.149002	0.166251	0.013217	0.007305	0.007894	0.642218
31882	0.013517	0.037125	0.905956	0.019229	0.024173	0.427826	0.038173	0.012062	0.087165	0.398307	0.036468	0.01897	0.036988	0.126694	0.018745	0.007201	0.005742	0.785658
31902	0.004469	0.003427	0.926182	0.02496	0.040962	0.567391	0.042252	0.004378	0.157853	0.224732	0.003393	0.020254	0.003363	0.2245	0.021981	0.003019	0.001503	0.725381
31903	0.003591	0.39307	0.578183	0.005575	0.019582	0.350504	0.00439	0.004011	0.138556	0.10916	0.393379	0.002474	0.393629	0.184422	0.001914	0.000114	0.003409	0.414037
31929	0.02081	0.246338	0.714594	0.010731	0.007527	0.533598	0.011338	0.020188	0.062319	0.126592	0.245964	0.001441	0.2463	0.118445	0.009873	0.008235	0.011308	0.604397
31931	0.006431	0.32985	0.645908	0.004091	0.013719	0.41248	0.005462	0.006785	0.100975	0.144758	0.329541	0.001533	0.329248	0.164311	0.001756	0.000124	0.006797	0.496231
31937	0.001335	0.229579	0.747715	0.012627	0.008744	0.478283	0.01571	0.00095	0.062427	0.214141	0.228489	0.003355	0.228777	0.10798	0.011974	0.000873	0.00032	0.646721
31938	0.010802	0.111937	0.840397	0.017124	0.01974	0.4095	0.023414	0.00971	0.141417	0.304451	0.111508	0.007118	0.112017	0.20438	0.014288	0.003289	0.006765	0.652144
31952	0.0155	0.044206	0.898846	0.017355	0.024093	0.392649	0.037514	0.013844	0.082883	0.429615	0.043495	0.020016	0.044265	0.117	0.017173	0.006399	0.008051	0.787097
31958	0.017032	0.069965	0.865817	0.02184	0.025345	0.528235	0.034826	0.016366	0.117754	0.232897	0.069924	0.014185	0.07005	0.181716	0.019714	0.00731	0.008972	0.698054
31960	0.012626	0.044072	0.894711	0.018639	0.029952	0.524609	0.041254	0.011958	0.083662	0.294583	0.043934	0.023429	0.044072	0.133994	0.017924	0.008562	0.003636	0.768383
31966	0.007908	0.116471	0.833257	0.017126	0.025238	0.5112	0.029485	0.007831	0.116376	0.218809	0.1163	0.013233	0.116303	0.184746	0.014701	0.004945	0.003058	0.663014
31970	0.014019	0.147488	0.799494	0.017106	0.021893	0.349824	0.026003	0.012695	0.13581	0.328683	0.146985	0.010146	0.147543	0.189996	0.014425	0.0054	0.007875	0.624615
31980	0.011171	0.148448	0.79772	0.015525	0.027136	0.481892	0.022567	0.011335	0.159844	0.175706	0.148656	0.00822	0.14896	0.247851	0.011264	0.003968	0.006723	0.573014
31984	0.003902	0.00229	0.948911	0.022089	0.022809	0.593051	0.034815	0.003631	0.101548	0.264668	0.002288	0.014043	0.002278	0.169013	0.020289	0.002791	0.001086	0.790499
31993	0.006081	0.005349	0.922397	0.027284	0.038888	0.552328	0.047869	0.00611	0.155984	0.232291	0.005419	0.022732	0.005406	0.232669	0.023693	0.003894	0.00225	0.709357
31994	0.00406	0.003481	0.942668	0.022215	0.027577	0.58548	0.037647	0.00401	0.116201	0.25314	0.003522	0.017071	0.003488	0.182662	0.020111	0.002812	0.001314	0.772543
31997	0.002602	0.003219	0.951213	0.021077	0.021889	0.490923	0.033749	0.002061	0.105362	0.364646	0.003259	0.013994	0.003334	0.152337	0.019894	0.001108	0.001284	0.808049
32025	0.016292	0.029584	0.908675	0.021172	0.024277	0.424818	0.040129	0.014792	0.087426	0.403748	0.029087	0.018852	0.029604	0.126973	0.020584	0.00968	0.006243	0.788063
32039	0.005307	0.113665	0.851115	0.011855	0.018058	0.293497	0.02123	0.00415	0.107444	0.46119	0.112488	0.009644	0.113421	0.146268	0.010585	0.002247	0.00286	0.714975
32040	0.020877	0.076474	0.835231	0.022491	0.044928	0.441854	0.049752	0.021076	0.147185	0.263926	0.076208	0.03037	0.076514	0.192818	0.019894	0.012204	0.008645	0.659556
32043	0.015096	0.052464	0.86633	0.024085	0.042026	0.595063	0.044968	0.015643	0.147476	0.144167	0.052683	0.024144	0.05251	0.226728	0.020705	0.009894	0.005553	0.660466
32053	0.000148	0.324442	0.6441	0.008727	0.022583	0.390968	0.005967	0.000169	0.176762	0.100859	0.325275	0.001366	0.325364	0.237721	0.003844	0.00001	0.000124	0.431571
32089	0.00514	0.367737	0.601279	0.007528	0.018315	0.359304	0.009913	0.005483	0.118452	0.139235	0.367613	0.003519	0.368087	0.181943	0.00426	0.000425	0.004538	0.437228
32103	0.017161	0.284723	0.67525	0.009145	0.013721	0.415032	0.012828	0.016877	0.091998	0.179064	0.284202	0.004629	0.284755	0.141468	0.007459	0.005734	0.010392	0.545563
32130	0.000581	0.275917	0.694361	0.009725	0.019416	0.427108	0.010641	0.000592	0.136618	0.149092	0.275948	0.003784	0.276085	0.190794	0.006446	0.000145	0.000408	0.522338
32132	0.003947	0.00409	0.916449	0.027753	0.047761	0.615025	0.04674	0.004156	0.190143	0.139822	0.004115	0.022335	0.004057	0.280197	0.023199	0.0022	0.001756	0.666256
32187	0.007227	0.282697	0.672016	0.011727	0.026334	0.397282	0.021191	0.007397	0.12058	0.170874	0.282675	0.011995	0.28288	0.167079	0.009281	0.004268	0.002866	0.521632
32207	0.001232	0.393927	0.595979	0.003213	0.005649	0.420149	0.001209	0.001206	0.082186	0.102409	0.392841	0.00037	0.392394	0.133866	0.001112	0.000066	0.001393	0.470799
32209	0.001401	0.359879	0.618066	0.003731	0.016923	0.381781	0.004822	0.001547	0.116755	0.135325	0.35977	0.003345	0.359776	0.165002	0.001101	0.000189	0.001315	0.469272
32215	0.007945	0.01112	0.929547	0.023514	0.027875	0.594048	0.039269	0.007797	0.115818	0.231798	0.01127	0.017393	0.011198	0.185422	0.021278	0.004581	0.003216	0.756913
32224	0.019693	0.166944	0.770397	0.015063	0.027903	0.419827	0.032947	0.019413	0.098003	0.263201	0.166609	0.018722	0.16699	0.148003	0.013631	0.010653	0.008486	0.633516
32227	0.004612	0.003715	0.940275	0.024767	0.026631	0.595169	0.038133	0.004397	0.119778	0.238674	0.003849	0.015221	0.003791	0.186347	0.022507	0.00297	0.001533	0.767632
32298	0.011265	0.0255	0.916417	0.023442	0.023377	0.610876	0.035829	0.010642	0.105737	0.211019	0.025897	0.013693	0.025697	0.172159	0.021682	0.006225	0.004579	0.755965
32299	0.004573	0.004582	0.935116	0.025395	0.030335	0.580828	0.038766	0.004521	0.147635	0.223607	0.004642	0.015513	0.004605	0.225314	0.022083	0.003247	0.001449	0.727789
32311	0.010321	0.013827	0.921047	0.025556	0.029249	0.581186	0.040628	0.009957	0.129261	0.224951	0.014017	0.016966	0.013932	0.200745	0.022877	0.006909	0.003343	0.735229
32356	0.002449	0.312759	0.647874	0.009463	0.027455	0.387244	0.012239	0.002857	0.166097	0.118174	0.31339	0.006586	0.313388	0.222359	0.005188	0.000555	0.002111	0.449813
32358	0.012758	0.244481	0.709918	0.009687	0.023156	0.427253	0.019367	0.013042	0.112152	0.183921	0.244265	0.010211	0.244611	0.17828	0.007159	0.008637	0.004179	0.546923
32363	0.001997	0.002917	0.952874	0.019495	0.022716	0.403904	0.030975	0.001343	0.128769	0.432209	0.0028	0.013126	0.00299	0.174147	0.017564	0.000725	0.001056	0.790392
32372	0.000021	0.155117	0.807219	0.011914	0.02573	0.391984	0.017867	0.00001	0.156883	0.278681	0.154574	0.009196	0.155156	0.206776	0.008534	0.000059	0.00001	0.620268
32451	0.012614	0.149237	0.798267	0.014688	0.025194	0.438064	0.023732	0.012492	0.139998	0.236668	0.149046	0.009815	0.149494	0.212788	0.011624	0.004043	0.008043	0.604193
32467	0.004796	0.101912	0.842997	0.022063	0.028233	0.599033	0.031872	0.005046	0.145451	0.116415	0.102183	0.011148	0.102114	0.242754	0.018138	0.003325	0.001581	0.62094
32469	0.005148	0.197658	0.768937	0.013448	0.014808	0.461325	0.021413	0.004298	0.075791	0.240179	0.196994	0.008521	0.197082	0.120266	0.012636	0.001775	0.00291	0.65681
32472	0.011238	0.143937	0.817998	0.011397	0.01543	0.394127	0.0216	0.010087	0.079097	0.351986	0.143103	0.010355	0.143749	0.120525	0.010819	0.002294	0.007766	0.704492
32539	0.009695	0.236109	0.716925	0.016037	0.021234	0.436521	0.023982	0.009307	0.112029	0.182455	0.235707	0.010206	0.235958	0.154967	0.013999	0.006492	0.002973	0.575405
32540	0.015636	0.14225	0.80245	0.015136	0.024528	0.448501	0.030814	0.015147	0.094546	0.269149	0.141843	0.016572	0.142084	0.141755	0.013962	0.006	0.008846	0.670781
32552	0.004673	0.126293	0.827136	0.01621	0.025689	0.513078	0.027115	0.004779	0.130502	0.19826	0.126266	0.01181	0.126295	0.206492	0.013304	0.001998	0.002535	0.637565
32553	0.016413	0.25263	0.705708	0.012871	0.012379	0.437251	0.016234	0.015747	0.089529	0.189202	0.252038	0.003906	0.252587	0.138124	0.011452	0.007262	0.008293	0.578377
32557	0.003837	0.261022	0.70816	0.009868	0.017113	0.443991	0.014411	0.003626	0.101089	0.176174	0.260709	0.006049	0.26077	0.151065	0.008087	0.000416	0.00311	0.570502
32560	0.003804	0.003754	0.925774	0.025543	0.041125	0.601204	0.043029	0.003857	0.162965	0.185175	0.003771	0.020374	0.00373	0.24171	0.022081	0.002747	0.001123	0.708236
32588	0.018992	0.046527	0.879993	0.022491	0.031998	0.511043	0.042442	0.01844	0.120433	0.260999	0.046644	0.021147	0.046788	0.180709	0.020432	0.011783	0.006895	0.712246
32591	0.012386	0.145823	0.796477	0.016638	0.028676	0.51827	0.025947	0.012864	0.153458	0.143322	0.146139	0.010888	0.146231	0.240264	0.01268	0.004523	0.00742	0.577994
32606	0.008455	0.118786	0.731831	0.023685	0.117243	0.499953	0.117117	0.012952	0.152544	0.096929	0.120505	0.100758	0.118857	0.230574	0.019717	0.005698	0.002954	0.521442
32629	0.010754	0.13911	0.809149	0.016091	0.024896	0.453762	0.024629	0.010656	0.141096	0.230943	0.138914	0.00936	0.139341	0.213932	0.01287	0.003943	0.006412	0.614142

TableS6_ProjectedAdmixture

ID	K=5 kindae/P. ursin	. cynocephalus	P. anubis	P. papio	P. hamadryas
32651	0.005552	0.004398	0.931351	0.025549	0.03315
32736	0.006544	0.184674	0.771278	0.017715	0.01979
32772	0.002282	0.002196	0.951826	0.018261	0.025435
32773	0.013232	0.064256	0.866689	0.024257	0.031566
32785	0.006992	0.128658	0.824237	0.017459	0.022653
32794	0.005195	0.123049	0.818227	0.021297	0.032232
32797	0.000098	0.249869	0.720433	0.012348	0.017252
32801	0.01008	0.108169	0.842507	0.016459	0.022785
32849	0.01645	0.103943	0.825354	0.022127	0.032126
32860	0.008634	0.014469	0.931434	0.020659	0.024804
32863	0.013687	0.27606	0.682404	0.007308	0.02054
32876	0.003393	0.289708	0.679786	0.007731	0.019382
32906	0.003886	0.402712	0.571709	0.004433	0.01726
32909	0.013611	0.047341	0.897126	0.019259	0.022663
32988	0.013436	0.138336	0.806698	0.014424	0.027105
32993	0.004633	0.005201	0.936894	0.025832	0.027441
32995	0.009764	0.08339	0.871354	0.013578	0.021913
32996	0.009226	0.304611	0.662847	0.005964	0.017351
33082	0.007076	0.323163	0.633133	0.012057	0.024572
33083	0.010391	0.096145	0.840298	0.023374	0.029792
33097	0.016588	0.129612	0.814639	0.015108	0.024053
33106	0.015179	0.098303	0.827472	0.022159	0.036888
33112	0.009033	0.265017	0.698544	0.011238	0.016168
33115	0.004777	0.005193	0.924753	0.026498	0.038779
33163	0.016578	0.286744	0.673089	0.008279	0.01531
33524	0.011956	0.154854	0.792328	0.017354	0.023509
33545	0.014335	0.052755	0.876286	0.023022	0.033601
33599	0.017937	0.254229	0.698449	0.009218	0.020168
33605	0.015316	0.238256	0.714752	0.015725	0.015951
33631	0.011904	0.133061	0.823928	0.010837	0.020271
33863	0.014592	0.047191	0.861379	0.025763	0.051075
33874	0.01081	0.110506	0.830987	0.018038	0.029659
33889	0.006269	0.003506	0.940877	0.022806	0.026542
33908	0.017278	0.066718	0.858985	0.021829	0.03519
34568	0.00251	0.353952	0.611189	0.010748	0.021601
34857	0.012027	0.123516	0.812694	0.021026	0.030737
34859	0.003129	0.00401	0.922021	0.025614	0.045227
34897	0.004601	0.004206	0.932912	0.022983	0.035297
6265	0.014641	0.014999	0.892544	0.026969	0.050847
6382	0.002293	0.001299	0.974497	0.010382	0.011529
6716	0.02565	0.300225	0.650913	0.006263	0.01695
6955	0.016918	0.017912	0.88687	0.027336	0.050964
7091	0.008646	0.004408	0.91626	0.024317	0.046369
7158	0.000017	0.512457	0.487164	0.00006	0.000302
7267	0.011192	0.003632	0.868893	0.031606	0.084677
7311	0.011809	0.006223	0.929172	0.02319	0.029606
7478	0.003093	0.001737	0.953707	0.022407	0.019056
7625	0.016075	0.014411	0.891274	0.027775	0.050465
7777	0.003686	0.001671	0.918542	0.026088	0.050013
7790	0.002228	0.002634	0.923018	0.025276	0.046845
7937	0.009441	0.002868	0.937154	0.02554	0.024998
8134	0.002775	0.002215	0.925497	0.024783	0.04473
8170	0.011917	0.007155	0.921333	0.024842	0.034753
8307	0.011916	0.005934	0.928954	0.023453	0.029743
8344	0.001582	0.444191	0.547099	0.002417	0.004711
8395	0.014877	0.014538	0.895431	0.026144	0.049009
8465	0.026352	0.221225	0.711527	0.011365	0.029532
8581	0.022294	0.193055	0.755099	0.012735	0.016817
8596	0.008021	0.178302	0.788047	0.014001	0.011629
8635	0.004564	0.002659	0.920145	0.025449	0.047183
8653	0.000252	0.000022	0.927356	0.024631	0.047739
8780	0.007188	0.005566	0.920137	0.023892	0.043217
8995	0.006957	0.002149	0.86021	0.021211	0.109473
9045	0.009799	0.005664	0.929464	0.02444	0.030632
9086	0.000379	0.350861	0.631368	0.004638	0.012754
9128	0.01358	0.014345	0.896768	0.026602	0.048704
9481	0.00001	0.627011	0.372526	0.000023	0.00043
9514	0.004881	0.001716	0.919788	0.027013	0.046602
9562	0.00001	0.00001	0.855245	0.070703	0.074032
9656	0.004508	0.649869	0.344561	0.000044	0.001019
9841	0.007521	0.002291	0.93506	0.025998	0.02913
9860	0.030519	0.24138	0.703327	0.010357	0.014417
9878	0.002763	0.002036	0.953212	0.023666	0.018323

ID	K=6 SNPRC P. anubis	amadryas/P. pa	kindae/P. ursin	P. anubis	NPRC P. anubis	. cynocephalus
32651	0.550783	0.042294	0.005459	0.145986	0.250992	0.004486
32736	0.486978	0.028666	0.005999	0.088556	0.205541	0.18426
32772	0.450452	0.034775	0.001689	0.106397	0.404503	0.002184
32773	0.525713	0.037359	0.013135	0.162299	0.197111	0.064383
32785	0.533378	0.02706	0.006955	0.114909	0.189162	0.128535
32794	0.524074	0.028824	0.005327	0.186485	0.13171	0.12358
32797	0.436762	0.012808	0.000082	0.136903	0.163804	0.249641
32801	0.429851	0.024978	0.009328	0.146103	0.281992	0.107748
32849	0.585167	0.036595	0.016565	0.126746	0.130705	0.104223
32860	0.416947	0.038887	0.007301	0.098635	0.424164	0.014066
32863	0.419548	0.016226	0.013949	0.100602	0.173879	0.275795
32876	0.410071	0.014196	0.003339	0.100274	0.182771	0.289349
32906	0.350043	0.001632	0.004169	0.13939	0.101907	0.402859
32909	0.427824	0.037072	0.012061	0.086139	0.390363	0.046542
32988	0.454281	0.027984	0.013303	0.122324	0.243882	0.138227
32993	0.584141	0.04047	0.004331	0.119268	0.246581	0.005209
32995	0.312586	0.02613	0.008607	0.112965	0.457099	0.082612
32996	0.418099	0.008224	0.009714	0.119151	0.140384	0.304427
33082	0.382388	0.014403	0.007638	0.152575	0.11968	0.323316
33083	0.601195	0.033361	0.010571	0.152253	0.106085	0.096536
33097	0.456587	0.030061	0.016053	0.095052	0.273006	0.129241
33106	0.46728	0.043509	0.014984	0.133469	0.242534	0.098224
33112	0.467616	0.013853	0.008932	0.111724	0.133022	0.264853
33115	0.613906	0.044226	0.004936	0.151627	0.180046	0.005258
33163	0.412104	0.013904	0.016383	0.089051	0.182308	0.28625
33524	0.510055	0.027855	0.011801	0.112801	0.182694	0.154795
33545	0.537698	0.03834	0.014377	0.159238	0.197386	0.052961
33599	0.415181	0.019445	0.017773	0.091808	0.201885	0.253908
33605	0.461688	0.020604	0.014887	0.102304	0.162689	0.237828
33631	0.393044	0.022859	0.010994	0.098556	0.342169	0.132379
33863	0.567199	0.050419	0.015428	0.175383	0.144047	0.047524
33874	0.470773	0.026188	0.01083	0.171783	0.209859	0.110566
33889	0.583066	0.038919	0.005972	0.105335	0.263131	0.003577
33908	0.501391	0.043073	0.017263	0.128578	0.243026	0.066669
34568	0.376902	0.014149	0.002778	0.132619	0.119404	0.354147
34857	0.567149	0.033177	0.012344	0.141023	0.122418	0.123889
34859	0.598597	0.044431	0.003341	0.18049	0.169084	0.004058
34897	0.557431	0.039187	0.004653	0.15938	0.235063	0.004285
6265	0.515192	0.060146	0.014902	0.145716	0.248958	0.015085
6382	0.252923	0.025902	0.000771	0.046797	0.672454	0.001153
6716	0.403773	0.020574	0.024753	0.035175	0.216039	0.299687
6955	0.515477	0.059925	0.017227	0.14891	0.240444	0.018017
7091	0.549957	0.043298	0.009061	0.205754	0.187529	0.004402
7158	0.292359	0.000112	0.000013	0.029422	0.167803	0.51029
7267	0.396198	0.074576	0.012315	0.281316	0.231985	0.003609
7311	0.490108	0.04722	0.01043	0.080247	0.365922	0.006073
7478	0.552546	0.037941	0.002311	0.06511	0.340416	0.001676
7625	0.520158	0.060468	0.016298	0.148422	0.240163	0.014492
7777	0.644009	0.035124	0.003618	0.281619	0.034101	0.001529
7790	0.612534	0.037424	0.002329	0.249864	0.095253	0.002596
7937	0.62976	0.043194	0.008719	0.077835	0.237668	0.002823
8134	0.600825	0.035888	0.002921	0.241555	0.116641	0.00217
8170	0.476101	0.049194	0.010961	0.111075	0.345645	0.007024
8307	0.5047	0.047433	0.010592	0.082235	0.349197	0.005843
8344	0.382749	0.001397	0.001438	0.057482	0.113857	0.443076
8395	0.509035	0.057928	0.015074	0.144535	0.258834	0.014594
8465	0.422329	0.027733	0.026661	0.109631	0.192505	0.22114
8581	0.447439	0.02443	0.021166	0.062809	0.251562	0.192595
8596	0.633762	0.013785	0.007173	0.095021	0.071392	0.178866
8635	0.596039	0.03768	0.004756	0.249012	0.109957	0.002556
8653	0.514027	0.034177	0.000146	0.266541	0.185091	0.000019
8780	0.506502	0.04913	0.00724	0.159732	0.271807	0.005589
8995	0.181739	0.098059	0.007519	0.256347	0.454377	0.001959
9045	0.521103	0.047919	0.008757	0.086125	0.330548	0.005548
9086	0.434836	0.004891	0.000396	0.100527	0.108707	0.350643
9128	0.515601	0.058355	0.013761	0.142149	0.255744	0.01439
9481	0.259851	0.00001	0.00001	0.045277	0.069732	0.625121
9514	0.642555	0.034704	0.005019	0.269043	0.047066	0.001613
9562	0.419385	0.01096	0.00001	0.219269	0.350365	0.00001
9656	0.226621	0.000021	0.00355	0.046397	0.074833	0.648577
9841	0.757918	0.045291	0.007139	0.084417	0.103026	0.00221
9860	0.447445	0.019146	0.029824	0.061574	0.201324	0.240687
9878	0.783929	0.033887	0.002505	0.075735	0.101959	0.001986

ID	K=7 P. hamadryas	. cynocephalus	P. anubis	P. papio	P. kindae	P. ursinus	NPRC P. anubis
32651	0.018535	0.004454	0.219001	0.022339	0.003756	0.001936	0.729979
32736	0.011944	0.184352	0.141384	0.016296	0.001659	0.004261	0.640104
32772	0.017599	0.002268	0.148389	0.017309	0.001124	0.001014	0.812298
32773	0.015344	0.064355	0.236941	0.020438	0.008278	0.004991	0.649652
32785	0.010454	0.128524	0.186641	0.014965	0.005338	0.001888	0.652188
32794	0.009364	0.123601	0.285364	0.01598	0.002841	0.002334	0.560516
32797	0.001762	0.250093	0.206069	0.008858	0.00001	0.00001	0.533198
32801	0.009463	0.108288	0.212781	0.01341	0.004217	0.005304	0.646537
32849	0.016875	0.104087	0.204331	0.019268	0.00998	0.00647	0.638988
32860	0.018173	0.014523	0.140205	0.019907	0.004206	0.003971	0.799015
32863	0.009542	0.276145	0.160811	0.005472	0.004653	0.008297	0.53508
32876	0.00846	0.289502	0.143367	0.005974	0.00097	0.002016	0.54971
32906	0.000821	0.402856	0.184656	0.000813	0.000403	0.003679	0.406773
32909	0.017644	0.047126	0.126743	0.018798	0.005887	0.006878	0.776924
32988	0.014883	0.138502	0.181936	0.012112	0.007883	0.005192	0.639491
32993	0.01638	0.005168	0.187958	0.023596	0.003479	0.001241	0.762178
32995	0.012611	0.083442	0.156524	0.012141	0.005784	0.003835	0.725663
32996	0.00273	0.305002	0.189139	0.00316	0.002819	0.00585	0.491301
33082	0.005545	0.323319	0.208756	0.008079	0.001936	0.005699	0.446666
33083	0.011714	0.096398	0.25078	0.019272	0.004708	0.005604	0.611524
33097	0.015699	0.129551	0.14321	0.013914	0.008646	0.007324	0.681657
33106	0.023644	0.098419	0.185996	0.01974	0.008917	0.005984	0.657301
33112	0.003878	0.265158	0.177466	0.008615	0.003581	0.004953	0.536349
33115	0.020214	0.005173	0.233032	0.023181	0.003552	0.001384	0.713464
33163	0.006418	0.286794	0.138898	0.006776	0.005605	0.009891	0.545619
33524	0.011624	0.154836	0.179835	0.015096	0.004799	0.006662	0.627149
33545	0.017567	0.052894	0.236114	0.019353	0.007162	0.006929	0.65998
33599	0.010767	0.254313	0.145616	0.00766	0.009368	0.007888	0.564388
33605	0.005693	0.238316	0.160711	0.013761	0.006533	0.00801	0.566977
33631	0.012361	0.133136	0.145386	0.009654	0.003817	0.00704	0.688607
33863	0.029113	0.047336	0.251848	0.021642	0.008491	0.006428	0.635141
33874	0.00941	0.110897	0.257586	0.013691	0.005195	0.005449	0.597773
33889	0.017454	0.003527	0.164466	0.021254	0.004506	0.001784	0.787008
33908	0.022884	0.066801	0.191221	0.019449	0.007993	0.008846	0.682806
34568	0.004396	0.354472	0.202733	0.006941	0.000101	0.002487	0.42887
34857	0.013532	0.123834	0.235656	0.017153	0.0077	0.004537	0.597588
34859	0.021867	0.003976	0.268315	0.02134	0.00208	0.001147	0.681276
34897	0.018489	0.004248	0.237267	0.019228	0.002745	0.001865	0.716157
6265	0.035688	0.015041	0.209337	0.023949	0.010776	0.004305	0.700904
6382	0.013913	0.001241	0.049882	0.012659	0.001449	0.000268	0.92059
6716	0.014837	0.300127	0.071832	0.006203	0.014905	0.009278	0.582818
6955	0.035102	0.017983	0.215691	0.02419	0.012184	0.005196	0.689653
7091	0.021577	0.0043	0.306597	0.018772	0.006392	0.002495	0.639867
7158	0.000087	0.510555	0.0529	0.000015	0.000012	0.00001	0.436421
7267	0.05021	0.003635	0.326199	0.025738	0.008891	0.003578	0.581749
7311	0.024625	0.006156	0.122835	0.022622	0.008189	0.003165	0.812407
7478	0.015963	0.001686	0.106555	0.022539	0.001828	0.00084	0.850589
7625	0.035069	0.01446	0.213311	0.024693	0.012535	0.004085	0.695847
7777	0.012585	0.001492	0.422069	0.017978	0.002414	0.00037	0.543091
7790	0.01494	0.002473	0.376858	0.017852	0.001596	0.000274	0.586008
7937	0.018589	0.002813	0.139692	0.024535	0.006559	0.002562	0.80525
8134	0.014126	0.0021	0.363101	0.017826	0.001261	0.001206	0.60038
8170	0.025298	0.007184	0.159241	0.023371	0.007823	0.003996	0.773086
8307	0.024416	0.005905	0.127538	0.02283	0.008209	0.003218	0.807886
8344	0.000507	0.442964	0.098066	0.000993	0.000167	0.001332	0.455969
8395	0.034206	0.014586	0.207289	0.023179	0.010403	0.004868	0.705467
8465	0.017802	0.221423	0.166451	0.009238	0.014224	0.011704	0.559158
8581	0.01231	0.192969	0.102245	0.012204	0.012625	0.008633	0.659013
8596	0.002462	0.17824	0.155424	0.012199	0.001794	0.005198	0.644684
8635	0.015649	0.002475	0.370723	0.018145	0.002667	0.001359	0.588983
8653	0.012015	0.000018	0.387441	0.01687	0.000115	0.000014	0.583527
8780	0.027068	0.005634	0.227877	0.020548	0.00504	0.002506	0.711326
8995	0.081815	0.002147	0.266984	0.016473	0.005703	0.002059	0.62482
9045	0.024166	0.005558	0.13517	0.023555	0.006984	0.002602	0.801965
9086	0.001983	0.350382	0.156876	0.002291	0.000015	0.000454	0.487999
9128	0.034225	0.01442	0.203317	0.023727	0.009955	0.003941	0.710416
9481	0.00001	0.625083	0.080056	0.000014	0.00001	0.00001	0.294817
9514	0.011599	0.001519	0.404705	0.01918	0.002757	0.001333	0.558907
9562	0.006268	0.00001	0.258198	0.015533	0.000018	0.00001	0.719963
9656	0.000042	0.648632	0.075754	0.00001	0.000016	0.003516	0.27203
9841	0.01957	0.00226	0.190187	0.023951	0.005055	0.002399	0.756578
9860	0.009679	0.241223	0.104593	0.009756	0.014183	0.014979	0.605587
9878	0.010689	0.001999	0.181898	0.021666	0.001576	0.001089	0.781082

Number of individuals with varying levels of minor ancestries based on number of PCs considered by PANE

	P. hamadryas		*P. kindae*		*P. papio*		*P. ursinus*	
**PCs**	>0,05	>0,1	>0,05	>0,1	>0,05	>0,1	>0,05	>0,1
3	7	1	0	0	92	4	7	4
5	84	14	0	0	39	2	4	4
6	77	13	2	2	42	2	4	2
10	190	19	2	2	107	2	4	2
15	194	19	2	2	107	2	4	2
20	458	22	2	2	348	2	6	2
25	559	25	2	2	490	2	6	2
30	607	33	2	2	556	3	6	2
35	752	105	2	2	779	18	6	2
40	771	155	2	2	802	47	6	2

Variance explained by each PC

**PC**	**Variance explained (%)**
**1**	31.90
**2**	5.77
**3**	4.65
**4**	3.84
**5**	2.56
**6**	2.34
**7**	2.11
**8**	1.84
**9**	1.80
**10**	1.79
**11**	1.73
**12**	1.70
**13**	1.68
**14**	1.64
**15**	1.61
**16**	1.60
**17**	1.57
**18**	1.32
**19**	1.31
**20**	1.30
**21**	1.27
**22**	1.26
**23**	1.21
**24**	1.18
**25**	1.17
**26**	1.16
**27**	1.14
**28**	1.13
**29**	1.12
**30**	1.11
**31**	1.10
**32**	1.10
**33**	1.08
**34**	1.08
**35**	1.07
**36**	1.06
**37**	1.04
**38**	1.03
**39**	1.02
**40**	0.99

ID	P. anubis	P. cynocephalu	P.hamadryas	P. kindae	P. papio	P. ursinus	P. kindae + P. ursinus
1X2054	0.9338390293	0	0.02130651573	0	0.04485445499	0	0
1X1960	0.9291713528	0	0.02576069293	0	0.04506795422	0	0
14712	0.9046543708	0	0.04120838039	0	0.05413724881	0	0
1Y0291	0.9323072509	0	0.01560370981	0	0.05208903924	0	0
1X1487	0	0.939903422	0	0.03035347311	0	0.02974310488	0.06009657799
1X4811	0	0.9982861866	0	0	0	0.00171381343	0.00171381343
1X0351	0.5227972715	0.1511304517	0.08990188381	0.1218674932	0.05637252404	0.05793037577	0.1797978689
1X0354	0.9091041059	0	0.04081294238	0	0.05008295172	0	0
1X0356	0.7659201031	0	0.1515272839	0	0.082552613	0	0
1X0843	0.9322524077	0.002495850654	0.03319298997	0.001745524294	0.03031322741	0	0.001745524294
1X1230	0.8434366765	0.01666151212	0.08467064115	0	0.05523117026	0	0
1X1237	0.8472705093	0.0100106388	0.0846778901	0	0.05804096185	0	0
1X1392	0.84765978	0.01213708787	0.0845498304	0	0.05565330173	0	0
1X1958	0.4413066855	0.4892852396	0.02408132161	0.01416195362	0.0216373477	0.009527451965	0.02368940558
1X2055	0.9256005153	0	0.02753637984	0	0.04686310487	0	0
1X2664	0.8690137766	0	0.07251205767	0	0.05847416568	0	0
1X3321	0.1416796262	0.5689445789	0.05936330622	0.131862411	0.02837482745	0.06977525028	0.2016376613
1X4519	0.826688083	0.01940106232	0.09701281523	0	0.05689803945	0	0
10046	0.9645644551	0	0.01287649518	0	0.02255904974	0	0
10099	0.8995026607	0	0.04916722687	0	0.05133011243	0	0
10164	0.6953498157	0.2147959653	0.05143375786	0	0.03842046113	0	0
10173	0.8950297254	0	0.06622252637	0	0.03874774823	0	0
10192	0.9303722027	0	0.02058563911	0	0.04904215819	0	0
10316	0.9624936781	0	0	0	0.03750632188	0	0
10349	0.504059118	0.4819400182	0	0	0.01400086372	0	0
10418	0.5048470393	0.4307424659	0.02697984079	0.01000932638	0.02163424156	0.005787086099	0.01579641248
10482	0.9489203701	0	0.007355023752	0	0.04372460615	0	0
10488	0.4502144496	0.4939777745	0.0128449434	0.01140819004	0.02419845605	0.007356186394	0.01876437643
10489	0.8894527679	0.008553242751	0.05923950769	0.001712191597	0.0410422901	0	0.001712191597
10842	0.9818277339	0	0	0	0.01817226609	0	0
10987	0.8860013453	0.006912237612	0.06059196655	0.003907383987	0.04258706651	0	0.003907383987
10998	0.9268511458	0	0.03877538747	0	0.03437346669	0	0
11004	0.7144226031	0.2329317115	0.0198290292	0.006019214187	0.02679744204	0	0.006019214187
11158	0.7036393429	0.2226013552	0.03932726223	0	0.0344320397	0	0
11562	0.6431078436	0.277411153	0.04090109889	0	0.03857990455	0	0
11608	0.9540012482	0	0.01040205421	0	0.03559669761	0	0
11693	0.772452179	0.1651572502	0.02932391957	0	0.03306665122	0	0
11752	0.9457352095	0	0.01522292129	0	0.03904186924	0	0
11769	0.6971807249	0.2401828731	0.02711540759	0	0.03552099436	0	0
11770	0.5522347294	0.3065135781	0.09678272131	0	0.04446897119	0	0
11885	0.9091165459	0	0.0537506634	0	0.03713279072	0	0
11887	0.9145874296	0.008551770131	0.04108251511	0	0.03577828516	0	0
11959	0.4768390277	0.4447172358	0.03980517261	0	0.03863856389	0	0
11967	0.7528535362	0.1751359338	0.0365634243	0	0.0354471057	0	0
11981	0.7007391103	0.2299800158	0.03419264206	0.002997153653	0.03209107823	0	0.002997153653
12138	0.9381427665	0	0.02682521097	0	0.03503202253	0	0
12141	0.74988564	0.1627527578	0.04439024721	0	0.04297135498	0	0
12152	0.7491594558	0.2147447408	0.01911444187	0	0.01560300086	0.001378360656	0.001378360656
12156	0.5674989628	0.3529214531	0.03830135203	0	0.04127823205	0	0
12175	0.5363630819	0.4394764133	0.007302497455	0	0.01685800732	0	0
12218	0.6814872221	0.2469669549	0.03562247578	0	0.03592334725	0	0
12226	0.6942357191	0.2235616324	0.04410381439	0.002882204322	0.03480199682	0.000414632989	0.003296837311
12242	0.9314461877	0	0.02634077451	0	0.04221303781	0	0
12282	0.7420328564	0.1935168136	0.03058721705	0	0.03301230845	0.000850804518	0.000850804518
12423	0.54720398	0.401018513	0.01822994014	0	0.03354756683	0	0
12427	0.8857468861	0.06688299744	0.02340619666	0	0.02396391985	0	0
12457	0.7712878871	0.1913263254	0.02179054986	0	0.01516255659	0.000432681076	0.000432681076
12473	0.5436137169	0.4157508972	0.01735033448	0	0.02328505143	0	0
12481	0.9834781111	0	0	0	0.01652188891	0	0
12491	0.7927038984	0.1536835266	0.02512466716	0	0.02848790788	0	0
12552	0.9853702438	0	0	0	0.01462975622	0	0
12656	0.7405265059	0.1442621944	0.04127859619	0.02245973921	0.0392051763	0.01226778798	0.03472752719
12681	0.454567633	0.4758467346	0.03758666667	0	0.03143754257	0.000561423188	0.000561423188

ID	P. anubis	P. cynocephalu	P.hamadryas	P. kindae	P. papio	P. ursinus	P. kindae + P. ursinus
12717	0.9801065322	0	0.000251265710	0	0.01964220207	0	0
12719	0.7848727553	0.1862269887	0.01613283837	0	0.01276741762	0	0
12720	0.6910557266	0.2548116767	0.02335222512	0	0.0307803715	0	0
12869	0.8147004848	0	0.1280191477	0	0.05728036746	0	0
12962	0.7689482181	0.02701009582	0.01486360857	0.01790600338	0.01783084429	0.1534412298	0.1713472332
12963	0.9298440842	0	0.02527786683	0	0.04487804901	0	0
13068	0.9749244676	0	0	0	0.0250755324	0	0
13103	0.875546185	0.0714590279	0.02386892677	0	0.02912586029	0	0
13110	0.9880179984	0	0	0	0.01198200163	0	0
13152	0.773372648	0.1417761538	0.04172565312	0.000157590156	0.04100883546	0.001959119449	0.002116709605
13169	0.7995612442	0.1542250881	0.0264449904	0	0.01958893019	0.000179747138	0.000179747138
13225	0.8469646534	0.08054164161	0.03600239455	0	0.03649131043	0	0
13226	0.8499535288	0	0.0863572018	0	0.06368926945	0	0
13228	0.8736686115	0.05701534846	0.0272499634	0	0.04206607666	0	0
13245	0.902144146	0	0.05123830322	0	0.04661755083	0	0
13264	0.4876518513	0.2664696925	0.2141784833	0	0.03169997287	0	0
13387	0.7759600538	0.189419547	0.01909928184	0	0.01534048043	0.000180636955	0.000180636955
13463	0.923407211	0	0.02922452943	0	0.04736825956	0	0
13570	0.6095966121	0.3257942556	0.03199218935	0.001092621595	0.02786636276	0.003657958645	0.00475058024
13575	0.7129034251	0.1977279984	0.04521032582	0	0.04331708164	0.000841169062	0.000841169062
13589	0.8059235754	0.02321533393	0.01517000891	0.01241855117	0.01894412124	0.1243284093	0.1367469605
13597	1	0	0	0	0	0	0
13644	0.8054207646	0.1765807936	0.005257688027	0.002357026357	0.005331531172	0.005052196282	0.007409222638
13645	0.8288923169	0.1154604578	0.02785131037	0	0.02779591484	0	0
13673	0.8524378288	0.09273768576	0.02697756418	0.000781295308	0.02650155451	0.000564071403	0.001345366711
13694	1	0	0	0	0	0	0
13698	0.9536596035	0	0.02021306893	0	0.02612732757	0	0
13739	0.8479778857	0.09958506185	0.02231999255	0.004071605923	0.02431239897	0.001733054985	0.005804660907
13859	0.8206072987	0.1241830493	0.02443380549	0.005127578487	0.02152815647	0.00412011152	0.009247690007
13914	0.751980524	0.2036651742	0.01909578666	0	0.02525851512	0	0
13942	0.8376867031	0.1013421518	0.02850895176	0.003544679771	0.02640264357	0.002514870038	0.006059549809
13951	0.9542987379	0	0.02072113785	0	0.02498012424	0	0
13988	0.9315566323	0	0.03743038177	0	0.03101298594	0	0
14012	0.9168315888	0.000238168634	0.04610772324	0.0050008279	0.03182169141	0	0.0050008279
14013	0.8982234328	0	0.04742078045	0	0.05435578674	0	0
14016	1	0	0	0	0	0	0
14022	0.846778945	0.07494771635	0.0397963763	0.004896976175	0.0322782019	0.001301784272	0.006198760447
14058	0.8895958776	0	0.05630261854	0	0.05410150381	0	0
14062	0.8969281934	0	0.05323509646	0	0.04983671018	0	0
14066	0.9510257324	0	0.01936890183	0	0.02960536575	0	0
14068	0.9171990496	0	0.04902362977	0	0.03377732068	0	0
14076	0.5716854666	0.4067656534	0	0	0.02154888	0	0
14077	0.9651065158	0	0.006302695588	0	0.02859078857	0	0
14154	0.9670315149	0	0.002040486393	0	0.03092799875	0	0
14158	0.7747963161	0.1479840531	0.03175934426	0	0.04546028656	0	0
14167	0.6311210603	0.3008865151	0.03229717765	0	0.03569524697	0	0
14172	0.4024828565	0.5537877745	0.01707720817	0.00355226268	0.02178208528	0.001317812801	0.00487007548
14181	0.5228079893	0.4669446606	0	0	0.01024735018	0	0
14182	0.579368922	0.3551113917	0.03925744908	0	0.02626223721	0	0
14191	0.7359775057	0.1950203551	0.03032241351	0	0.03867972565	0	0
14204	0.9499993555	0	0.02305713234	0	0.02694351215	0	0
14250	0.9329899655	0	0.03061444465	0	0.03639558985	0	0
14273	0.4251937942	0.5340260709	0.01516854525	0	0.01988567358	0.005725915989	0.005725915989
14276	0.590116453	0.359928972	0.02022308182	0.01146266724	0.01826882592	0	0.01146266724
14282	0.9611371222	0	0.009762292164	0	0.02910058561	0	0
14290	0.6819272677	0.2723431477	0.01971779185	0	0.02601179275	0	0
14324	0.8568866934	0.0689510726	0.04020033562	0	0.0339618984	0	0
14330	0.9572587848	0	0.01312566088	0	0.02961555436	0	0
14342	0.9801537558	0	0.01160754824	0	0.008238695946	0	0
14343	0.8640172637	0.06056786292	0.03100424727	0	0.04441062608	0	0
14350	0.9161352417	0	0.04205429411	0	0.04181046421	0	0
14351	0.9303294572	0	0.03080714197	0	0.03886340078	0	0
14355	0.5518977809	0.4100722655	0.01474310283	0	0.02328685076	0	0

ID	P. anubis	P. cynocephalu	P.hamadryas	P. kindae	P. papio	P. ursinus	P. kindae + P. ursinus
14369	0.5974033576	0.3543653653	0.02340255824	0	0.02482871888	0	0
14379	0.8641889211	0.06431987759	0.03676506501	0.002726819452	0.03199931683	0	0.002726819452
14427	0.6163555984	0.3153817152	0.03934525462	0	0.02891743178	0	0
14435	0.9148742322	0.001129220377	0.04699968001	0.004034421054	0.03296244633	0	0.004034421054
14437	0.9583200807	0	0.01111385136	0	0.03056606796	0	0
14460	0.6930381789	0.2363888064	0.03968456443	0	0.03088845033	0	0
14473	0.9863870201	0	0	0	0.01361297994	0	0
14498	0.7775458942	0.1436610533	0.04289297416	0	0.03590007839	0	0
14526	0.900292769	0	0.04848014905	0	0.05122708192	0	0
14642	0.94240617	0	0.01913708742	0	0.03845674256	0	0
14652	0.9551143709	0	0.01692736688	0	0.02795826222	0	0
14668	0.9790812824	0	0.000744256195	0	0.02017446144	0	0
14690	0.9323258435	0	0.03294782713	0	0.03472632939	0	0
14695	0.8500384091	0.1101988201	0.01600263302	0.000530785174	0.02322935262	0	0.000530785174
14696	0.8510855063	0.09419510242	0.02746815361	0	0.02725123772	0	0
14740	0.9074383929	0	0.04046650194	0	0.05209510513	0	0
14753	0.9031132014	0.04321197517	0.01634429306	0	0.03733053035	0	0
14756	0.931697958	0	0.03648890875	0.002512293143	0.02930084012	0	0.002512293143
14795	0.9161473933	0	0.03805501226	0	0.04579759449	0	0
14800	0.5507017827	0.3740527089	0.03587378568	0	0.036597723	0.002773999763	0.002773999763
14833	0.9421668201	0	0.02819450956	0	0.02963867039	0	0
14847	0.6485832369	0.298770629	0.02643408634	0	0.02621204768	0	0
14850	0.9307233813	0	0.03448742746	0	0.03478919127	0	0
14852	0.9541188935	0	0.03407005033	0	0.01181105619	0	0
14860	0.9123177259	0	0.0349796239	0	0.05270265023	0	0
14863	0.9579591145	0	0.005876316689	0	0.03616456882	0	0
14865	0.9593667624	0	0.01319274018	0	0.02744049746	0	0
14867	1	0	0	0	0	0	0
14886	0.9845536126	0	0.002971478082	0	0.01247490936	0	0
14908	0.3357553357	0.6103566026	0.02101739528	0.007310544904	0.0157862217	0.009773899798	0.0170844447
14909	1	0	0	0	0	0	0
14916	0.9162058727	0	0.0437097057	0	0.04008442162	0	0
14922	0.9142231792	0	0.0443077088	0	0.04146911202	0	0
14924	0.9108265611	0	0.04317558232	0	0.04599785662	0	0
14925	0.9895415155	0.000227958425	0.004219233948	0	0.0060112921	0	0
14930	0.9297403324	0	0.03576644654	0	0.03449322103	0	0
14943	0.6787944891	0.2693977255	0.02152348039	0	0.03028430495	0	0
14944	0.5935480599	0.3374524549	0.03330449497	0.002596127588	0.02996355188	0.003135310743	0.005731438331
14948	0.7757988066	0.2169943039	0.006768688078	0	0.000438201422	0	0
14951	0.4981643474	0.3624817151	0.118177646	0	0.02117629153	0	0
14959	0.9298581381	0	0.03562881413	0	0.03451304775	0	0
14989	0.9547039919	0	0.009853032793	0	0.03544297526	0	0
14994	1	0	0	0	0	0	0
15009	0.8731519424	0	0.06904767929	0	0.05780037831	0	0
15107	0.9480981868	0	0.02095060993	0	0.03095120325	0	0
15112	0.7240429895	0.2078058256	0.03049646356	0	0.03765472134	0	0
15113	0.6435046349	0.3136479703	0.01973139735	0	0.02311599747	0	0
15122	0.6249919217	0.3230154887	0.02796609109	0	0.02402649852	0	0
15149	0.7242075164	0.2199543579	0.02199547558	0	0.03384265008	0	0
15150	0.8920347317	0	0.05640398609	0	0.05156128225	0	0
15156	0.9247736841	0	0.03582480969	0	0.03940150624	0	0
15157	0.7644628265	0.1728511932	0.02982738156	0	0.03142968522	0.001428913577	0.001428913577
15163	0.9345598981	0	0.02992213677	0	0.03551796515	0	0
15175	0.8268307527	0.156590245	0.005959444324	0	0.00774869633	0.002870861686	0.002870861686
15178	0.9944829337	0	0	0	0.005517066329	0	0
15190	0.9799307677	0	0.003474724696	0	0.01659450761	0	0
15197	0.6099148022	0.3271601299	0.03796867215	0	0.02495639576	0	0
15211	0.9090018142	0	0.04852570729	0	0.04247247848	0	0
15212	0.9562727584	0	0.006535340361	0	0.03719190124	0	0
15217	0.8392594687	0.1170550755	0.01812470113	0.001913076375	0.02084668131	0.002800996906	0.004714073281
15225	0.3731062915	0.5838480344	0.01362464812	0.000055760421	0.01901610481	0.01034916075	0.01040492117
15232	0.2306375164	0.7429566736	0.01105026289	0.003520147882	0.00639978439	0.005435614862	0.008955762744
15244	0.8471109779	0.09057724378	0.02959438999	0.003616875434	0.0279576312	0.001142881728	0.004759757162

ID	P. anubis	P. cynocephalu	P.hamadryas	P. kindae	P. papio	P. ursinus	P. kindae + P. ursinus
15267	1	0	0	0	0	0	0
15274	0.8716748289	0.06582769389	0.03489930033	0	0.02759817687	0	0
15279	1	0	0	0	0	0	0
15286	0.9941155329	0	0	0	0.005884467087	0	0
15290	0.4963430072	0.4234197543	0.03968206113	0.001212879835	0.03267248606	0.00666981146	0.007882691294
15292	0.9118677768	0	0.04328388616	0	0.04484833707	0	0
15308	0.7007711329	0.2441503971	0.02535772854	0	0.02972074153	0	0
15414	0.9587755586	0	0.01194821843	0	0.02927622301	0	0
15419	0.9429337234	0	0.0289998207	0	0.02806645592	0	0
15421	0.9453226305	0	0.01960181522	0	0.03507555424	0	0
15442	0.5530487883	0.3315849432	0.09527946296	0	0.02008680552	0	0
15444	0.9194123297	0	0.0432015891	0	0.03738608123	0	0
15458	0.9105894024	0	0.04934016927	0	0.0400704283	0	0
15467	0.9325225053	0	0.03577732717	0	0.03170016758	0	0
15475	0.9117230784	0	0.04853173363	0	0.03974518797	0	0
15486	0.7839702944	0.1633704016	0.02327496084	0.001419361418	0.02666143245	0.001303549262	0.00272291068
15494	0.695102756	0.2194302035	0.04694176383	0	0.03852527668	0	0
15509	0.9195589213	0	0.03734918361	0	0.04309189508	0	0
15515	0.7063994647	0.2351264529	0.02527029326	0.004962958435	0.02350597895	0.004734851697	0.009697810132
15523	0.6966178889	0.2550736788	0.02713964967	0.002531686612	0.018637096	0	0.002531686612
15545	0.6484809083	0.2872647264	0.03201831282	0	0.03223605252	0	0
15553	0.8557974445	0.09506660628	0.02437209162	0	0.02476385764	0	0
15560	0.9882751326	0	0.003793090389	0	0.007931776985	0	0
15562	0.7970189663	0.1328124965	0.03807057737	0.003875242509	0.02822271738	0	0.003875242509
15566	0.3807438848	0.5827057357	0.01815055408	0	0.01663323725	0.001766588162	0.001766588162
15568	0.5770997016	0.3343894437	0.04345397252	0	0.04505688227	0	0
15571	0.9280789128	0	0.03503165743	0	0.03688942981	0	0
15575	0.9299774864	0	0.03409237122	0	0.03593014235	0	0
15579	0.7141829849	0.2331762334	0.02431791296	0.002837928758	0.02265463718	0.002830302803	0.005668231561
15581	0.2576167708	0.6991222169	0.01830814832	0.00382502835	0.01284742393	0.008280411616	0.01210543997
15584	0.8169456771	0.1250101733	0.03002163226	0	0.02802251741	0	0
15599	0.5840940014	0.3560555852	0.02997929653	0	0.02987111679	0	0
15600	0.8049048284	0.139787044	0.02713089135	0	0.02817723633	0	0
15626	0.9220337146	0	0.04235740756	0.000889385682	0.03471949219	0	0.000889385682
15628	0.8703547111	0	0.0712564266	0	0.05838886225	0	0
15633	0.9505172387	0	0.01845235081	0	0.03103041049	0	0
15652	0.8231967522	0.08111052519	0.0579829219	0	0.03770980074	0	0
15653	0.566515202	0.402820775	0.009537321637	0	0.02112670135	0	0
15659	0.9433300503	0	0.02076472759	0	0.03590522209	0	0
15664	0.9601663224	0	0.00311962696	0	0.03671405061	0	0
15733	0.8491523925	0.05742041121	0.05235867853	0.009746589826	0.02785598531	0.003465942617	0.01321253244
15736	0.756180928	0.1886327751	0.0254887418	0	0.02969755518	0	0
15820	0.5553293388	0.3637616996	0.04119682154	0.000252937727	0.0390326153	0.000426587041	0.000679524769
15824	0.9898023165	0	0.000983138590	0	0.009214544874	0	0
15845	0.9037685249	0	0.05391019228	0	0.04232128279	0	0
15846	0.3834665101	0.5822955665	0.01333048017	0	0.01881163177	0.002095811436	0.002095811436
15849	0.3963530473	0.577034076	0.01156224179	0	0.01505063485	0	0
15870	0.9880183056	0	0	0	0.01198169443	0	0
15874	0.8912480648	0	0.0535135127	0	0.05523842246	0	0
15889	0.9803536967	0	0.005612658631	0	0.01403364469	0	0
15938	0.9820545136	0	0.002723278798	0	0.01522220761	0	0
15940	0.9872360933	0	0	0	0.01276390667	0	0
15944	0.9298497601	0	0.03563114882	0	0.03451909107	0	0
15975	0.6432916703	0.2700401697	0.0351145348	0	0.0515536252	0	0
15976	0.9375729972	0	0.02792034545	0	0.0345066573	0	0
15979	0.9618820745	0	0.004784012955	0	0.03333391254	0	0
15996	0.717774078	0.236226737	0.02200769127	0	0.02399149377	0	0
16000	0.9842360676	0	0.002172044514	0	0.01359188787	0	0
16006	0.8005967032	0.1381544576	0.02828388267	0	0.03296495651	0	0
16017	0.9244585294	0	0.04011563995	0	0.03542583063	0	0
16019	0.9001882563	0	0.04929079634	0	0.05052094738	0	0
16056	0.9445896605	0	0.02689286903	0	0.02851747052	0	0
16058	0.6478635192	0.2732678809	0.04258401567	0	0.03628458418	0	0

ID	P. anubis	P. cynocephalu	P.hamadryas	P. kindae	P. papio	P. ursinus	P. kindae + P. ursinus
16059	0.5062405998	0.4293987244	0.03512088183	0	0.02923979392	0	0
16065	0.5938721082	0.3925011724	0.00133498531	0	0.01229173406	0	0
16067	0.9514757893	0	0.01571883161	0	0.03280537906	0	0
16072	0.7146871589	0.2323916839	0.02522885539	0	0.02769230175	0	0
16083	0.9403139525	0	0.02876104063	0	0.03092500684	0	0
16092	0.9493103131	0	0.02047043894	0	0.030219248	0	0
16094	0.74967718	0.1800905664	0.0287805245	0	0.04145172913	0	0
16101	0.9155271028	0	0.03664158528	0	0.04783131194	0	0
16104	0.9239039613	0.000167707787	0.04232163708	0	0.03360669386	0	0
16112	0.9963103518	0	0	0	0.003689648201	0	0
16115	0.33916732	0.6249498602	0.01586577134	0.001797822652	0.01120837412	0.007010851616	0.008808674268
16121	0.7315066991	0.2195156746	0.02658128314	0.000919905504	0.02147643769	0	0.000919905504
16122	0.9507147472	0	0.02033899333	0	0.02894625952	0	0
16243	0.6139243398	0.3252595857	0.03420263218	0	0.02661344235	0	0
16244	0.9243768753	0	0.02993208633	0	0.04569103841	0	0
16246	0.2589036525	0.7126004038	0.009078184399	0.004012624191	0.008444404625	0.006960730562	0.01097335475
16254	0.8198019704	0.1158921846	0.03241490776	0	0.03189093722	0	0
16261	0.9292921257	0	0.03354572578	0	0.03716214854	0	0
16263	0.8389330112	0.09707007615	0.03175860328	0	0.03223830939	0	0
16288	0.962302456	0	0.005577008247	0	0.03212053576	0	0
16289	0.6507326357	0.2919785807	0.03133547043	0	0.02595331317	0	0
16300	0.8204513936	0.08573268595	0.05626789561	0	0.03754802483	0	0
16304	0.676161284	0.2761657562	0.02306084978	0	0.02461211005	0	0
16316	0.9498209357	0	0.01721123738	0	0.03296782694	0	0
16321	0.7862530863	0.1994949137	0.005797688771	0	0.008454311264	0	0
16329	0.3827389244	0.5619294976	0.02890142065	0.000883871793	0.02106037573	0.004485909818	0.005369781612
16338	0.9106607455	0	0.03968635784	0	0.04965289665	0	0
16363	0.9947227706	0	0	0	0.005277229356	0	0
16369	0.780036546	0.1722213644	0.02345390252	0	0.02428818708	0	0
16371	0.7242513145	0.1859184699	0.05029302727	0	0.03953718828	0	0
16372	0.957799954	0	0.01352517561	0	0.02867487036	0	0
16383	0.3312173581	0.6254674815	0.01468476689	0.006401395205	0.01373503518	0.008493963186	0.01489535839
16385	0.7944240624	0.1422950399	0.03461698361	0	0.02866391407	0	0
16386	0.7594160431	0.1937014641	0.02532460911	0.000230724434	0.02132715934	0	0.000230724434
16391	0.6639853024	0.2883431874	0.02675177933	0.000730222548	0.01960420329	0.000585305075	0.001315527624
16400	0.632545735	0.2991568851	0.04120808086	0	0.02708929907	0	0
16408	0.9356875181	0	0.02666254847	0	0.03764993342	0	0
16409	0.9812182017	0	0.000021205443	0	0.01876059285	0	0
16413	0.9015343924	0	0.05483529783	0	0.04363030975	0	0
16417	0.5090579767	0.4236030305	0.03550970872	0	0.03182928414	0	0
16420	0.9530781935	0	0.02062415172	0	0.02629765478	0	0
16424	0.4188242531	0.5362000367	0.01623174588	0.000169972017	0.02199812617	0.006575866143	0.00674583816
16436	0.7913256191	0.186707368	0.008382261886	0	0.01274042824	0.00084432277	0.00084432277
16486	0.8262453289	0.1206520601	0.02243860506	0	0.03066400601	0	0
16499	0.6057227518	0.357113498	0.01165566572	0	0.0255080845	0	0
16507	0.915079953	0	0.04368364025	0	0.04123640678	0	0
16510	0.9305873564	0	0.0226267099	0	0.04678593367	0	0
16517	0.9162201044	0	0.04241179142	0	0.04136810419	0	0
16524	0.8866122113	0	0.05981046023	0	0.05357732843	0	0
16527	0.9675771834	0	0.01167415307	0	0.02074866357	0	0
16538	0.9242404844	0	0.03507207361	0	0.04068744196	0	0
16548	0.9262565899	0	0.03475200059	0	0.03899140948	0	0
16550	0.9315578505	0	0.03870777811	0	0.0297343714	0	0
16562	0.927237536	0	0.03047408501	0	0.04228837898	0	0
16569	1	0	0	0	0	0	0
16671	0.7868686691	0.1575128329	0.02713150241	0	0.02848699564	0	0
16678	0.8428160474	0.0971966403	0.0276455561	0	0.03234175625	0	0
16692	0.9503672793	0	0.02308546577	0	0.02654725491	0	0
16702	0.9348022326	0	0.03281446537	0	0.03238330202	0	0
16708	0.9106444072	0	0.04009517338	0	0.0492604194	0	0
16719	0.7831318315	0.1551254402	0.03131522601	0.000680379475	0.02974712289	0	0.000680379475
16731	0.7056674934	0.2386828858	0.03119156706	0.000208641894	0.02424941189	0	0.000208641894
16741	0.8837923759	0	0.06208664112	0	0.05412098298	0	0

ID	P. anubis	P. cynocephalu	P.hamadryas	P. kindae	P. papio	P. ursinus	P. kindae + P. ursinus
16743	0.3339936526	0.6250189919	0.01488657002	0.002487357519	0.01797151912	0.005641908872	0.00812926639
16744	0.7651195857	0.1801724937	0.02744040165	0	0.02726751897	0	0
16748	0.7496419902	0.168163554	0.0445570134	0	0.03763744238	0	0
16761	0.6233203795	0.3394637183	0.01186225794	0	0.02535364428	0	0
16775	0.9272714902	0	0.03760558292	0	0.03512292693	0	0
16780	0.6383664366	0.2907598785	0.03478063585	0	0.0360930491	0	0
16787	0.658866129	0.2923806941	0.02415951245	0	0.0245936645	0	0
16798	0.6256502312	0.2955910252	0.03645345468	0	0.04230528889	0	0
16808	0.9094476047	0	0.0493927092	0	0.04115968607	0	0
16815	0.7206807973	0.2217166795	0.02926414641	0	0.02833837677	0	0
16816	0.5033019308	0.4222847114	0.03746376155	0	0.03403426069	0.002915335511	0.002915335511
16823	0.9488039807	0	0.01633843087	0	0.03485758842	0	0
16827	0.9196511409	0	0.03465253522	0	0.04569632386	0	0
16829	0.9227970964	0	0.03773142228	0	0.03947148134	0	0
16847	0.4632753782	0.5306471922	0	0	0.006077429637	0	0
16853	0.4785565387	0.447125422	0.03632508244	0	0.03799295685	0	0
16856	0.955399927	0	0.007942510892	0	0.03665756216	0	0
16861	0.9201668929	0.01229407513	0.03589114315	0	0.0316478888	0	0
16862	0.3167207024	0.6320166781	0.02631087537	0.005891716488	0.01475907699	0.004300950688	0.01019266718
16866	0.4280453059	0.534955345	0.01842767714	0	0.01852092025	0.000050751735	0.000050751735
16873	0.9460378286	0	0.02107163571	0	0.03289053573	0	0
16879	0.7537853961	0.1934121294	0.02778104613	0.001176992642	0.02384443574	0	0.001176992642
16880	0.9849838303	0	0	0	0.01501616966	0	0
16889	0.3481768095	0.5965404877	0.0192121531	0.006020704814	0.0193281297	0.01072171515	0.01674241997
16890	0.527409833	0.3947878075	0.03674000386	0	0.0410623557	0	0
16986	0.9389087714	0	0.01698664907	0	0.0441045795	0	0
16999	0.9445406471	0	0.01851213953	0	0.0369472134	0	0
17006	0.5297101466	0.4106288745	0.02650880919	0	0.02543320132	0.00771896843	0.00771896843
17024	0.5896337878	0.3506567449	0.0263089703	0	0.03340049698	0	0
17111	0.5444515396	0.4336841067	0.004574863649	0	0.01728949001	0	0
17112	0.9391478356	0	0.02990744437	0	0.03094472	0	0
17139	0.593854549	0.3079245327	0.07322002037	0	0.02500089792	0	0
17141	0.5278535807	0.440529986	0.01026873707	0	0.02134769623	0	0
17153	0.8575241	0.09092380506	0.02560599812	0	0.02594609686	0	0
17158	0.9511137807	0	0.01581761122	0	0.03306860808	0	0
17159	0.7793803343	0.169908946	0.02388371157	0	0.02682700815	0	0
17165	0.5111646913	0.4790575154	0	0	0.009777793263	0	0
17174	0.7747207021	0.1611733916	0.03052256514	0	0.0335833412	0	0
17184	0.8318116937	0.1049825643	0.03054701185	0.002782103028	0.02987662718	0	0.002782103028
17190	0.6143741146	0.3012305569	0.04068282936	0.001687769208	0.03909609084	0.002928639072	0.00461640828
17199	0.9127621879	0	0.04698037978	0	0.04025743231	0	0
17222	0.8729463759	0.06835315709	0.02784061658	0.000360622738	0.03049922772	0	0.000360622738
17228	0.8986430639	0.04002235453	0.02434911594	0	0.03698546563	0	0
17232	0.6774508084	0.2705887093	0.03010764092	0	0.02185284141	0	0
17233	0.9166180565	0	0.03780487159	0	0.04557707191	0	0
17254	0.298174332	0.6827293702	0.009778722928	0	0.006915502455	0.002402072407	0.002402072407
17255	0.5284773498	0.3882237312	0.04147843415	0	0.04182048491	0	0
17268	0.5636503991	0.3726786868	0.04029180634	0	0.02337910773	0	0
17294	0.9413753221	0	0.02710735432	0	0.0315173236	0	0
17295	0.6249608715	0.3338281141	0.01804726052	0	0.02316375393	0	0
17325	0.9474306488	0	0.02143192042	0	0.03113743079	0	0
17328	0.919103745	0	0.03706399331	0	0.04383226165	0	0
17340	0.9370873552	0	0.02493721772	0	0.03797542708	0	0
17776	0.9521426539	0	0.01548259429	0	0.03237475183	0	0
17792	0.5752462534	0.3957209134	0.008185848273	0	0.02084698491	0	0
17795	0.6168071103	0.3410128096	0.01366172586	0	0.02851835421	0	0
17826	0.5425669129	0.4168013774	0.01873067134	0	0.02190103842	0	0
17829	0.5525970001	0.425431867	0.003890050462	0	0.01808108242	0	0
17832	0.9382556308	0	0.02778007379	0	0.03396429542	0	0
17833	0.3507852397	0.60587242	0.01550207764	0.005578227446	0.01804648337	0.004215551856	0.009793779301
17840	0.5776177384	0.3440903003	0.04110973789	0	0.0371822234	0	0
17851	0.7029773055	0.2004872808	0.0487381734	0	0.04779724028	0	0
17852	0.8367310936	0.1022540053	0.03103330426	0	0.02998159679	0	0

ID	P. anubis	P. cynocephalu	P.hamadryas	P. kindae	P. papio	P. ursinus	P. kindae + P. ursinus
17871	0.9083923288	0	0.04223739759	0	0.04937027357	0	0
17873	0.9844355463	0	0	0	0.0155644537	0	0
17879	0.6820411067	0.2763004805	0.02205216029	0	0.01960625253	0	0
17891	0.8909676071	0.009003570009	0.05477119722	0	0.0452576257	0	0
17898	0.5952423529	0.3760793918	0.008925667079	0	0.01975258819	0	0
17903	0.9115326349	0	0.04688444399	0	0.04158292107	0	0
17916	0.982334941	0	0	0	0.01766505902	0	0
17929	0.7661848216	0.170046526	0.03061897205	0	0.0328055391	0.000344141293	0.000344141293
17969	0.7297966137	0.2086209659	0.02628569373	0	0.0352967266	0	0
17970	0.7308726393	0.2100690425	0.02378458579	0	0.03527373236	0	0
17971	0.9682354666	0	0	0	0.03176453344	0	0
17981	0.9246836249	0.01661206637	0.02159177962	0	0.03711252914	0	0
17997	0.9776718901	0	0	0	0.02232810987	0	0
17998	0.9297999977	0	0.03029464502	0	0.03990535728	0	0
17999	0.9778308291	0	0	0	0.02216917094	0	0
18004	0.6618464423	0.301348551	0.01836247114	0	0.01844253556	0	0
18015	0.3541401071	0.5955681332	0.01744361795	0.006009774444	0.01657862016	0.01025974707	0.01626952151
18019	0.5479219618	0.3239944243	0.1009706764	0	0.0271129375	0	0
18022	0.9672462445	0	0.006139174972	0	0.02661458049	0	0
18024	0.9347808455	0.01000955917	0.02339689091	0	0.03181270447	0	0
18141	0.978629593	0	0	0	0.02137040705	0	0
18144	0.8989527727	0.01287911712	0.0432094953	0	0.04495861493	0	0
18168	0.6805496818	0.2982271318	0.000969277612	0	0.02025390872	0	0
18341	0.6981893892	0.1955654035	0.06821754645	0	0.03802766086	0	0
18385	0.9180178954	0	0.04228078654	0	0.0397013181	0	0
18395	0.6907705658	0.2607333347	0.02318085507	0	0.02531524446	0	0
18431	0.9549082431	0	0.01273464201	0	0.03235711491	0	0
18469	0.6710232775	0.2816042516	0.0264351213	0	0.0201808968	0.000756452759	0.000756452759
18523	0.941397896	0	0.02353787119	0	0.03506423283	0	0
18528	0.6284733813	0.3299827251	0.01946746403	0	0.02045792352	0.001618506085	0.001618506085
18529	0.6042096161	0.3595154887	0.01306953439	0	0.02320536088	0	0
18557	0.8394712329	0.1115489663	0.02657450581	0	0.02240529497	0	0
18586	0.6940346742	0.2538695797	0.02141088688	0.003441956152	0.02437050549	0.002872397548	0.0063143537
18599	0.9214822773	0	0.03844457805	0	0.04007314464	0	0
18672	0.8833886608	0	0.05929768676	0	0.0573136524	0	0
18713	0.5999751072	0.3328398977	0.03152086958	0.005776600007	0.02988752556	0	0.005776600007
18715	0.9276279297	0	0.03171481682	0	0.04065725349	0	0
18769	0.6624134125	0.2943242435	0.02086028571	0.001893982298	0.01870151576	0.00180656024	0.003700542538
18778	0.2941310937	0.6828608785	0.01211678647	0	0.00967022633	0.001221015001	0.001221015001
18789	0.8199109001	0.1047343986	0.03603504927	0	0.03931965202	0	0
18818	0.7739052061	0.1752486885	0.02363560252	0	0.02721050288	0	0
18840	0.9258799352	0	0.03410674275	0	0.04001332208	0	0
18841	0.9479234114	0	0.02261153139	0	0.02946505724	0	0
18866	0.6909731859	0.2434054785	0.03221837302	0.003668921717	0.02536450133	0.004369539557	0.008038461274
18892	0.8222728878	0.1161141834	0.03292734758	0	0.02868558121	0	0
18917	0.831927514	0.1214460409	0.02149099828	0.000996750921	0.02413869591	0	0.000996750921
18929	0.7321714292	0.1716937346	0.05176728199	0	0.04436755415	0	0
18938	0.8497983954	0.09019860393	0.02906814645	0	0.03093485419	0	0
18972	0.6158287649	0.3252534619	0.02763061572	0	0.03128715746	0	0
18974	0.2989005002	0.6522182636	0.01305158898	0.01267879477	0.01347236986	0.009678482585	0.02235727735
19012	0.7173459574	0.2237395604	0.02910185039	0.003581406022	0.02267039873	0.003560827088	0.00714223311
19144	0.6100840399	0.3738525555	0.001494029376	0	0.01456937527	0	0
19154	0.6638544214	0.2484952961	0.04845062289	0	0.0391996596	0	0
19165	0.9661639967	0	0	0	0.03383600333	0	0
19173	0.9795041864	0	0	0	0.02049581359	0	0
19181	0.912744843	0	0.04589168893	0	0.04136346802	0	0
19207	0.868254377	0.02308362925	0.06519127778	0	0.04347071596	0	0
19215	0.974294398	0	0	0	0.02570560198	0	0
19237	0.5482120601	0.427697768	0.006106828724	0	0.01798334316	0	0
19348	0.9145472698	0	0.04386161414	0	0.04159111606	0	0
19359	0.9514714644	0.001681812222	0.01812030018	0	0.02872642322	0	0
19365	0.9274617726	0	0.03684312834	0	0.03569509909	0	0
19371	0.9807053638	0	0	0	0.0192946362	0	0

ID	P. anubis	P. cynocephalu	P.hamadryas	P. kindae	P. papio	P. ursinus	P. kindae + P. ursinus
19376	0.5845336136	0.3394763302	0.04134550528	0	0.03464455099	0	0
19378	0.7621949953	0.1571852502	0.04370965724	0	0.03691009732	0	0
19381	0.4168520959	0.5315720399	0.02856383577	0.001083164557	0.02131949017	0.000609373782	0.001692538339
19658	0.7824055125	0.1574414725	0.03127036095	0	0.02888265401	0	0
19674	0.927500932	0	0.03958597383	0	0.03291309421	0	0
19679	0.9084647436	0	0.05599113998	0	0.03554411645	0	0
19750	0.9090231981	0	0.04418639538	0	0.04679040649	0	0
1X0830	0.7441940955	0.1399522037	0.04128693674	0.02228670206	0.04034564218	0.01193441978	0.03422112184
1X1155	0.941369578	0.003844085461	0.02871032591	0.002382734444	0.02369327616	0	0.002382734444
1X1693	0.7436248065	0.1396341521	0.04132425861	0.02328271441	0.04033061409	0.01180345432	0.03508616873
1X2124	0.8304728803	0	0.1184749381	0	0.05105218159	0	0
1X2231	0.9986211038	0	0.001378896168	0	0	0	0
1X2816	0.9589313178	0	0.01824534663	0	0.02282333558	0	0
1X2892	0.7413110844	0.1425791926	0.04190099372	0.02147398458	0.04078209178	0.01195265298	0.03342663757
1X3656	0.9208412055	0	0.02927875572	0	0.04988003874	0	0
1X3697	0.9010753439	0	0.04595937637	0	0.05296527975	0	0
1X3796	0.4796785626	0.4440458219	0.03841085384	0.001747837842	0.03223540608	0.003881517763	0.005629355604
1X3822	0.9053712872	0	0.05320956077	0	0.04141915198	0	0
1X3837	0.5196149734	0.4596439111	0.004009088442	0	0.01673202714	0	0
1X4080	0.9257266299	0.000103220734	0.0347141137	0	0.03945603568	0	0
1X4179	0.9158629264	0	0.03220106456	0	0.05193600905	0	0
1X4209	0.481784666	0.4376202163	0.03668097666	0	0.04391414108	0	0
1X4696	0.903719607	0	0.04718751159	0	0.04909288145	0	0
1X4739	0.9205947368	0	0.0394578274	0	0.0399474358	0	0
1X4777	0.9731529753	0	0.00775320512	0	0.01909381955	0	0
1X4859	0.8987136715	0	0.05097612297	0	0.05031020553	0	0
20117	0.916956345	0.02246773018	0.03289802105	0	0.02767790372	0	0
25347	0.8588438974	0.05911494131	0.04960528166	0.00203076269	0.03021483928	0.000190277662	0.002221040352
25354	0.9131260018	0	0.04989100751	0	0.03698299067	0	0
25355	0.3124181768	0.6621968622	0.01145635483	0	0.008463813674	0.005464792486	0.005464792486
25409	0.6226422451	0.306780509	0.03887312592	0	0.03170412004	0	0
25593	0.7234425111	0.1956817587	0.0318755001	0.004384400689	0.04327159533	0.001344234055	0.005728634744
26101	0.9135900194	0	0.0357369073	0	0.05067307327	0	0
26138	0.6452378729	0.2807159828	0.03449326125	0.002601714083	0.03152935052	0.005421818416	0.008023532498
26196	0.8037568357	0.077062066	0.06935973997	0	0.04982135831	0	0
26198	0.944787289	0	0.02449661288	0	0.03071609811	0	0
26310	0.9228427759	0.02014193456	0.02976108363	0	0.02725420594	0	0
26316	0.9135871345	0.02209323852	0.03231653635	0	0.0320030906	0	0
26318	0.9121057104	0.02291325834	0.03445442727	0.000916108704	0.02961049527	0	0.000916108704
26324	0.7226272408	0.2071053549	0.03143895349	0	0.03882845072	0	0
26355	0.7320494124	0.2054777105	0.03173875324	0.004298675579	0.02643544831	0	0.004298675579
26498	0.7690382412	0.184526028	0.02647746404	0	0.01995826678	0	0
26800	0.9868741425	0	0.003486856333	0	0.009639001139	0	0
26980	0.9089785283	0.07960587379	0.005851892935	0	0.005563704957	0	0
26988	0.9123785883	0	0.04817314668	0	0.03944826506	0	0
27033	0.5841040972	0.353347624	0.03102857604	0	0.03151970276	0	0
27036	0.9332459932	0	0.03624279999	0	0.03051120686	0	0
27166	0.9499025296	0	0.01915881479	0	0.03093865557	0	0
27181	0.9409344196	0	0.02577231186	0	0.03329326851	0	0
27190	0.5969950925	0.3514727474	0.02750428281	0.003491931212	0.0205359461	0	0.003491931212
27193	0.5855188712	0.344005978	0.03195681878	0.01036252552	0.02815580649	0	0.01036252552
27199	0.9330636863	0	0.0339046005	0	0.03303171316	0	0
27230	0.870090837	0.05130752025	0.03422347527	0	0.04437816745	0	0
27237	0.7016899086	0.256673334	0.01506662721	0	0.02657013016	0	0
27267	0.6685791556	0.3174477456	0.006798097454	0	0.007175001339	0	0
27306	0.895310093	0	0.05380770482	0	0.05088220221	0	0
27351	0.8698504334	0.1101518163	0.008515476975	0.001267962506	0.008379889995	0.001834420803	0.003102383309
27358	0.8701300287	0.1064177822	0.003475739091	0	0.01875914951	0.001217300487	0.001217300487
27433	0.6186060778	0.309607592	0.03540779787	0.001940290646	0.03014788298	0.004290358619	0.006230649264
27472	0.9474403458	0	0.02115220733	0	0.03140744686	0	0
27503	0.924604834	0	0.03817648484	0	0.03721868114	0	0
27525	0.8921808142	0.03690736455	0.03678303809	0	0.03412878319	0	0
27592	0.8956920515	0.04215172011	0.03042052659	0	0.03173570177	0	0

ID	P. anubis	P. cynocephalu	P.hamadryas	P. kindae	P. papio	P. ursinus	P. kindae + P. ursinus
27594	0.8967430136	0.08254975969	0.00889468723	0	0.01181253948	0	0
27622	0.7518330306	0.1729956609	0.03696600231	0	0.03820530618	0	0
27643	0.6293936207	0.3023351305	0.03768961749	0	0.02758539142	0.002996239917	0.002996239917
27666	0.8089787485	0.1671489011	0.01112364763	0	0.01176916274	0.000979540032	0.000979540032
27668	0.4592834574	0.4879769171	0.02872815978	0	0.02365902035	0.000352445443	0.000352445443
27686	0.7536426768	0.1850850397	0.03177485109	0.003244428417	0.02625300391	0	0.003244428417
27742	0.4143219101	0.5367628151	0.02396036199	0	0.02071955637	0.004235356485	0.004235356485
27811	0.8049628589	0.1650327112	0.01592219411	0	0.01408223573	0	0
27814	0.670237731	0.2728913244	0.03102226321	0	0.02584868143	0	0
27820	0.8428204412	0.1107955503	0.02325527361	0.000407988758	0.02272074612	0	0.000407988758
27827	0.9250846323	0.01715724182	0.02992437787	0	0.02783374805	0	0
27841	0.359912247	0.5984108734	0.01504231567	0.006328701603	0.01834825094	0.001957611433	0.008286313036
27844	0.9657250516	0	0.009071590756	0	0.02520335765	0	0
27874	0.9638475656	0	0.009090674803	0	0.02706175961	0	0
27885	0.4298524056	0.5370707914	0.01742705687	0	0.01564974618	0	0
27889	0.9512673778	0	0.01576673116	0	0.03296589105	0	0
27908	0.4215798831	0.5456935486	0.01713255363	0	0.01559401464	0	0
27985	0.5754140343	0.3627185638	0.03656437844	0	0.02530302344	0	0
27989	0.908467145	0	0.04558213639	0	0.04595071863	0	0
27999	0.9096537996	0	0.04349825932	0	0.04684794106	0	0
28003	0.7037918166	0.07140082839	0.02317213377	0	0.2016352213	0	0
28021	0.8363216236	0.1040313643	0.02209329712	0.003618784571	0.0323002041	0.001634726315	0.005253510886
28037	0.5275272508	0.4347074285	0.01723895026	0	0.02052637047	0	0
28074	0.7110230072	0.2174803667	0.03512348322	0.000278722862	0.03605640584	0.000038014201	0.000316737064
28075	0.5890653671	0.3209803527	0.05745073056	0	0.0325035497	0	0
28076	0.8538848393	0.08728613749	0.02960987553	0	0.0292191477	0	0
28102	0.9359764724	0	0.03260113155	0	0.03142239604	0	0
28183	0.8798731298	0	0.06532478478	0	0.05480208547	0	0
28212	0.9951552773	0	0	0	0.004844722738	0	0
28246	0.9090604404	0	0.04989433542	0	0.0410452242	0	0
28259	0.7151682477	0.217797367	0.03315738331	0	0.03387700199	0	0
28269	0.7287678059	0.1957964992	0.02962522192	0.004222949804	0.03860116545	0.002986357651	0.007209307455
28274	0.6985170114	0.2109223472	0.04659705847	0	0.04396358291	0	0
28279	0.8480394622	0.1239708411	0.01106003134	0.003038031031	0.01120707196	0.002684562342	0.005722593373
28281	0.4606471395	0.5302058665	0	0	0.009146994015	0	0
28285	0.8294578088	0.09177481264	0.03547211575	0	0.0429142144	0.000381048441	0.000381048441
28304	0.938505755	0	0.03004142722	0	0.03145281783	0	0
28328	0.9389771156	0	0.02808254468	0	0.03294033971	0	0
28368	0.7661383717	0	0.190246772	0.003714441712	0.03889602855	0.001004385953	0.004718827665
28375	0.5992889384	0.3536035898	0.0215276634	0	0.02557980842	0	0
28376	0.7929172925	0.1300490638	0.03968406867	0	0.0373495751	0	0
28385	0.9165310061	0.01575338166	0.03764476425	0	0.030070848	0	0
28386	0.85295091	0.08734604689	0.02709622914	0.006222299012	0.02543228433	0.000952230632	0.007174529645
28400	0.9876580473	0	0.003832668892	0	0.008509283843	0	0
28416	0.9947410573	0	0	0	0.005258942745	0	0
28417	0.9087567199	0	0.0454451003	0	0.04579817982	0	0
28428	0.9273113525	0.01319922729	0.03349823053	0	0.02599118973	0	0
28431	0.6955787096	0.2316115177	0.03319503611	0.003522889537	0.03311254996	0.002979297068	0.006502186605
28436	0.9056355127	0	0.04730943274	0	0.0470550546	0	0
28438	0.9144194041	0	0.04009851597	0	0.04548207993	0	0
28478	0.6668390841	0.2784803279	0.03082515974	0	0.02385542834	0	0
28495	0.5327592876	0.3975582596	0.05032817883	0	0.01935427394	0	0
28497	0.916699914	0.01744153149	0.03697077344	0	0.02888778103	0	0
28576	0.9521776081	0	0.01879532205	0	0.0290270699	0	0
28591	0.9982741288	0	0	0	0.001725871207	0	0
28609	0.9214632496	0.01727946199	0.03073418223	0	0.0305231062	0	0
28618	0.9968450134	0	0	0	0.003154986641	0	0
28635	0.804628279	0.09538698562	0.05823748483	0	0.04174725053	0	0
28729	0.9350640101	0	0.02873624899	0	0.03619974092	0	0
28740	0.733744782	0.1794135002	0.04542673726	0	0.04141498047	0	0
28742	0.9060065029	0.02714234583	0.03816532813	0	0.02868582311	0	0
28779	0.8858060548	0.01659620076	0.04768745791	0	0.04991028657	0	0
28799	0.9195296815	0	0.0370370366	0	0.04343328188	0	0

ID	P. anubis	P. cynocephalu	P.hamadryas	P. kindae	P. papio	P. ursinus	P. kindae + P. ursinus
28801	0.9603137599	0	0.01436319075	0	0.02532304933	0	0
28802	0.8955794234	0.04621600364	0.03196901793	0	0.02623555506	0	0
28806	0.9201659991	0	0.03771563651	0	0.04211836441	0	0
28830	0.9485262395	0	0.01272204325	0	0.03875171728	0	0
28925	0.9500121242	0	0.01881500793	0	0.03117286785	0	0
28949	0.816208635	0.1059551145	0.03410290969	0.002852686833	0.03757858309	0.003302070846	0.00615475768
29156	0.8482828683	0.08626339335	0.0308548891	0.003907874509	0.02770830993	0.002982664851	0.006890539361
29333	0.8425473819	0.09229905808	0.03135914365	0.000869800163	0.03159535796	0.001329258221	0.002199058384
29368	0.9589344343	0	0.01301307791	0	0.02805248776	0	0
29565	0.6712895639	0.2623498221	0.03524262496	0	0.03111798908	0	0
29758	0.837933561	0.08727233704	0.03359839976	0.001939604165	0.03715165432	0.002104443693	0.004044047858
30008	0.8403498961	0	0.005575432478	0	0.1540746714	0	0
30033	0.8564290188	0.08963236483	0.02295234591	0	0.02989850344	0.001087766982	0.001087766982
30104	0.772246021	0.1409822792	0.04011663409	0.000618988949	0.04299874783	0.003037328974	0.003656317923
30320	0.7646980606	0.09522457957	0.09236084509	0.002088577824	0.04517636456	0.000451572312	0.002540150136
30366	0.930042181	0.000217861436	0.03468070762	0	0.03505924995	0	0
30373	0.8426188156	0.04581239245	0.07007282086	0	0.04149597108	0	0
30424	0.8805101802	0.06156699871	0.02127718585	0.000868794582	0.03577684061	0	0.000868794582
30508	0.7869922457	0.1559019927	0.02098333919	0	0.03325914407	0.002863278333	0.002863278333
30564	0.7275912773	0.2050205276	0.02995918024	0	0.03742901482	0	0
30609	0.7028050615	0.2451273953	0.02679041497	0	0.02527712828	0	0
30611	0.8642974243	0.04987690561	0.04358306129	0	0.0422426088	0	0
30614	0.8518224039	0.1177076418	0.01695285726	0	0.0135170971	0	0
30636	0.9459096496	0	0.01395319743	0	0.04013715295	0	0
30650	0.9764032654	0	0.001975563964	0	0.02162117066	0	0
30660	0.8197531689	0.1083859647	0.03502949252	0	0.03683137387	0	0
30662	0.9774409969	0	0.001642462982	0	0.02091654009	0	0
30673	0.8238363988	0.1064091539	0.03296154135	0.001009794846	0.0357831111	0	0.001009794846
30699	0.5693053213	0.3787641784	0.01896796784	0	0.03296253248	0	0
30749	0.8030481188	0.1024247398	0.05477225851	0	0.03945414667	0.000300736278	0.000300736278
30751	0.8165066672	0.1143637311	0.03557739427	0	0.03355220745	0	0
30752	0.9312560924	0	0.02651856483	0	0.04222534274	0	0
30772	0.4651900868	0.4692311022	0.02519931886	0.006499911269	0.02810769685	0.005771883967	0.01227179524
30871	0.9457535477	0	0.015383976	0	0.03886247628	0	0
30878	0.911475515	0.01862397739	0.03287479867	0	0.03702570894	0	0
30886	0.7108149903	0.2185204418	0.03053214276	0	0.04013242508	0	0
30889	0.8460616623	0.06015962789	0.05540922066	0	0.03836948913	0	0
30907	0.7552887401	0.2018291307	0.01651955219	0	0.02636257699	0	0
30917	0.8279610016	0.09820825673	0.04026344528	0.000346047713	0.03322124867	0	0.000346047713
30920	0.6974439693	0.2345159018	0.03728285536	0.000196672399	0.03056060116	0	0.000196672399
30927	0.8473866464	0.06790701833	0.03819140395	0	0.04651493131	0	0
30933	0.6784950614	0.2707135439	0.02604817511	0	0.02474321953	0	0
30934	0.9374282508	0	0.02308574401	0	0.03948600523	0	0
30961	0.9366384333	0	0.0300385634	0	0.03332300327	0	0
30973	0.9527048463	0	0.01508201499	0	0.03221313875	0	0
30974	0.7100254231	0.2376820558	0.01819372892	0.003436778061	0.02820385554	0.002458158513	0.005894936574
31011	0.7485163657	0.2178844113	0.01113456028	0	0.02246466276	0	0
31015	0.7031930234	0.1325160503	0.116351996	0.003414594214	0.03852097737	0.006003358665	0.009417952879
31016	0.8945846909	0	0.05532665982	0	0.05008864924	0	0
31021	0.8423963936	0.1223267551	0.02074673148	0	0.01453011984	0	0
31025	0.6117801408	0.2340422099	0.1233918992	0	0.03078575016	0	0
31026	0.9410971362	0	0.02628023479	0	0.03262262899	0	0
31028	0.6490885723	0.3027489883	0.02421565903	0	0.02394678041	0	0
31035	0.5355955018	0.3987809741	0.02891750651	0.005956012523	0.03075000506	0	0.005956012523
31064	0.9443066368	0	0.0188965338	0	0.03679682936	0	0
31065	0.6564329661	0.2871486736	0.03121185347	0	0.02520650683	0	0
31072	0.9495026018	0	0.01282775965	0	0.03766963854	0	0
31086	0.8088461525	0.1082167047	0.04109597355	0	0.04184116934	0	0
31110	0.9363532709	0	0.02861777548	0	0.0350289536	0	0
31115	0.8586898088	0	0.1014643066	0	0.03984588461	0	0
31120	0.6387184929	0.3001208595	0.02027085331	0	0.03760532559	0.003284468717	0.003284468717
31124	0.8953527109	0.03437286414	0.03508738996	0	0.03518703498	0	0
31130	0.5845229387	0.3668148269	0.01734578819	0	0.03131644624	0	0

ID	P. anubis	P. cynocephalu	P.hamadryas	P. kindae	P. papio	P. ursinus	P. kindae + P. ursinus
31131	0.7565817858	0.211845649	0.007990740496	0	0.02358182469	0	0
31134	0.9397357781	0.01430702531	0.02019135696	0	0.02576583967	0	0
31145	0.8165640105	0.09643027369	0.04364095696	0	0.04336475884	0	0
31152	0.9345456656	0	0.02356581094	0	0.04188852346	0	0
31177	0.5192760418	0.4228668362	0.02619933803	0.002576939155	0.0251297031	0.003951141722	0.006528080876
31178	0.6783171328	0.2657210141	0.02322552306	0.001896182166	0.02747521276	0.003364935137	0.005261117303
31234	0.8548774661	0.08162489474	0.03122429049	0	0.03227334866	0	0
31235	0.9586658773	0	0.01188312447	0	0.02945099822	0	0
31255	0.8726153837	0.06009286008	0.02721881709	0	0.04007293916	0	0
31275	0.8060111291	0.149359688	0.01387939716	0	0.03074978581	0	0
31284	0.847299336	0.1040145228	0.02487938391	0	0.02380675723	0	0
31288	0.6148406941	0.3319225903	0.02159361273	0.004749291005	0.02407664133	0.00281717054	0.007566461545
31297	0.8982024147	0.04541736259	0.02794262997	0.000858601684	0.02757899104	0	0.000858601684
31299	0.9042011128	0.04575025113	0.0274995581	0	0.02254907793	0	0
31300	0.6099880084	0.3108223674	0.03159322611	0.004645632981	0.03763762244	0.005313142658	0.00995877564
31311	0.7894438784	0.1508737398	0.02720923499	0	0.03247314687	0	0
31327	0.9419893714	0	0.02993951344	0	0.0280711152	0	0
31329	0.9480128736	0	0.01571936559	0	0.03626776077	0	0
31331	0.7831575157	0.166671027	0.02669816016	0	0.0234732971	0	0
31333	0.9412426288	0	0.02263306048	0	0.03612431071	0	0
31337	0.7275689036	0.2040144972	0.03962898828	0	0.02878761089	0	0
31358	0.5888221213	0.3460374208	0.02978906244	0.006673002269	0.02330538553	0.005373007624	0.01204600989
31364	0.7695126115	0.1681501146	0.03333248324	0.003070510391	0.02593428031	0	0.003070510391
31392	0.9410118933	0	0.02145764103	0	0.03753046564	0	0
31398	0.933572391	0	0.02756447977	0	0.03886312921	0	0
31401	0.8398778117	0.09962923638	0.02948700603	0	0.03100594589	0	0
31407	0.9229084665	0	0.03900031998	0	0.03809121353	0	0
31408	0.6915821831	0.2413405247	0.03651818376	0	0.03055910837	0	0
31409	0.8303027838	0.1156863307	0.02737003746	0	0.02664084813	0	0
31410	0.6586827585	0.2801433517	0.03547256514	0	0.02570132468	0	0
31415	0.8234135973	0.0870900257	0.04469777889	0	0.04479859815	0	0
31417	0.7855290934	0.1479165409	0.03517190205	0	0.03138246372	0	0
31440	0.9151419909	0	0.03581394526	0	0.04904406387	0	0
31457	0.7549039337	0.1896941646	0.01876204347	0.001276201937	0.03315476776	0.002208888571	0.003485090508
31462	0.8238077569	0.1131673093	0.03528800906	0	0.02773692474	0	0
31469	0.9310983297	0	0.03039061276	0	0.03851105753	0	0
31471	0.7920729877	0.1413907311	0.0340337847	0	0.03250249646	0	0
31475	0.6995674583	0.2379948847	0.02701595512	0.005738339573	0.02630769637	0.003375665914	0.009114005487
31477	0.5532915989	0.3879319972	0.03206132335	0	0.02671508058	0	0
31482	0.9033047578	0	0.04522818054	0	0.05146706165	0	0
31484	0.933414063	0	0.02407422004	0	0.04251171701	0	0
31490	0.9287464325	0	0.02785468003	0	0.04339888745	0	0
31497	0.6870092124	0.2445559366	0.0320257901	0	0.03640906088	0	0
31498	0.8137352558	0.1522229158	0.02045918873	0	0.01358263961	0	0
31499	0.9353246279	0	0.02721983133	0	0.03745554076	0	0
31500	0.9206530183	0	0.0416614012	0	0.03768558046	0	0
31506	0.6965480426	0.2533752043	0.02828385777	0	0.02179289532	0	0
31507	0.7405314242	0.2182881467	0.01455963993	0	0.02662078918	0	0
31511	0.8257008377	0.1090738749	0.0327000816	0	0.0325252058	0	0
31515	0.8818305574	0.04665929601	0.03462765115	0	0.03688249544	0	0
31518	0.9053978304	0.01665507903	0.03820106812	0	0.03974602249	0	0
31525	0.6997179181	0.2624992109	0.0146931188	0	0.02041458671	0.002675165493	0.002675165493
31526	0.9587060235	0	0.008409155853	0	0.03288482069	0	0
31528	0.9330165354	0.002604302353	0.02760300059	0	0.0367761617	0	0
31540	0.9457038742	0	0.02102051954	0	0.03327560622	0	0
31542	0.8029976064	0.09739618011	0.05265562342	0	0.04695059008	0	0
31543	0.5858992667	0.3462794546	0.02926016834	0.000129654314	0.03656938089	0.00186207511	0.001991729424
31552	0.9312061326	0.003091343905	0.02927154007	0	0.03643098345	0	0
31553	0.9680697465	0	0.008164152659	0	0.02376610086	0	0
31556	0.8927734161	0	0.06938863586	0	0.03783794807	0	0
31559	0.9421187092	0.007875928937	0.02257981882	0	0.02742554306	0	0
31560	0.7272105328	0.2151844034	0.02738479121	0	0.03022027265	0	0
31573	0.8618302994	0.03962712786	0.04958146951	0	0.04896110319	0	0

ID	P. anubis	P. cynocephalu	P.hamadryas	P. kindae	P. papio	P. ursinus	P. kindae + P. ursinus
31576	0.8891225545	0.03358166844	0.04083633269	0	0.03645944435	0	0
31578	0.9559940514	0	0.01306138983	0	0.03094455876	0	0
31602	0.7117509827	0.139512833	0.1064693975	0	0.04226678673	0	0
31605	0.9589498988	0	0.005905368185	0	0.03514473302	0	0
31624	0.8825282321	0.04985495817	0.03342262506	0	0.03419418463	0	0
31630	0.9025418303	0	0.04455586251	0	0.0529023072	0	0
31642	0.7990522142	0.1129220361	0.04368099799	0	0.04434475173	0	0
31650	0.9634671014	0	0.00942128012	0	0.02711161847	0	0
31656	0.7224491768	0.2459584632	0.01058239595	0	0.02100996411	0	0
31659	0.8888827062	0.04639557138	0.03250159065	0	0.03222013181	0	0
31666	0.5398227528	0.3887554363	0.02747133209	0.007858626888	0.03279784011	0.00329401186	0.01115263875
31693	0.9305547375	0	0.02615493157	0	0.04329033091	0	0
31716	0.7624030694	0.17678075	0.03425355269	0	0.02656262791	0	0
31720	0.8100055321	0.1128343067	0.0368581963	0	0.04030196487	0	0
31729	0.5827431528	0.3601268988	0.02680110318	0	0.03032884519	0	0
31731	0.9417425536	0	0.02289104523	0	0.03536640115	0	0
31738	0.7786464927	0.1611770728	0.03059043452	0.001699313796	0.02788668617	0	0.001699313796
31764	0.8368703246	0.09799234524	0.03102478535	0.002343577081	0.03176896768	0	0.002343577081
31778	0.8036958923	0.1256713423	0.03929660891	0	0.03133615646	0	0
31786	0.8594781392	0.08636173965	0.02700297098	0	0.02715715013	0	0
31789	0.6273634377	0.3171101189	0.02242175481	0.001548478958	0.02992622486	0.001629984732	0.00317846369
31800	0.7101073633	0.2180278924	0.03439747404	0	0.03746727032	0	0
31804	0.9375574076	0	0.02746958483	0	0.03497300753	0	0
31805	0.7552232381	0.1774063382	0.02911408849	0	0.03825633519	0	0
31836	0.7659246813	0.1513401137	0.04306167981	0	0.03967352511	0	0
31846	0.6979994407	0.2411636856	0.03516268235	0	0.0252668104	0.000407381027	0.000407381027
31849	0.9799761401	0	0	0	0.02002385988	0	0
31852	0.6349212293	0.3065700463	0.02420966164	0.003162548879	0.02659895643	0.004537557471	0.00770010635
31880	0.7966558682	0.1423093136	0.03205671152	0	0.0289781067	0	0
31882	0.9223089359	0.02939259981	0.02235481026	0	0.02594365402	0	0
31902	0.9387949657	0	0.02622785224	0	0.03497718204	0	0
31903	0.5243218884	0.4121239383	0.0365022943	0	0.027051879	0	0
31929	0.6949771828	0.2473008614	0.01858983823	0.00469784145	0.03052146549	0.0039128106	0.00861065205
31931	0.6016398813	0.3400454978	0.03195956364	0	0.02635505728	0	0
31937	0.7450991376	0.2221207387	0.006495873622	0	0.02628425002	0	0
31938	0.8386667908	0.1005254491	0.02780601862	0	0.03300174146	0	0
31952	0.9135168607	0.03947530165	0.02433133816	0	0.02267649946	0	0
31958	0.8719906736	0.06124300392	0.03026399014	0	0.03650233236	0	0
31960	0.9063711692	0.03643265684	0.02961684056	0	0.02757933337	0	0
31966	0.8378575299	0.102625373	0.02885869713	0	0.03065839993	0	0
31970	0.7937725285	0.1394797916	0.0319359907	0	0.03481168919	0	0
31980	0.7910079697	0.1367226956	0.03840250548	0	0.03386682922	0	0
31984	0.9660449984	0	0.004135911185	0	0.02981909046	0	0
31993	0.9301032682	0	0.0307142489	0	0.03918248287	0	0
31994	0.9616293191	0	0.007357500654	0	0.03101318029	0	0
31997	0.9658014086	0	0.007767363599	0	0.02643122785	0	0
32025	0.9234626316	0.0235109768	0.02422073821	0.000982183320	0.02748727691	0.000336193195	0.001318376516
32039	0.8686218657	0.0985514225	0.01598207319	0	0.01684463859	0	0
32040	0.8390276452	0.06795671594	0.05502153089	0	0.03799410793	0	0
32043	0.8608674517	0.0419803254	0.05420539573	0	0.04294682719	0	0
32053	0.6010610395	0.327134025	0.03826715611	0	0.0335377794	0	0
32089	0.5570076846	0.3834821753	0.03181203704	0	0.02769810306	0	0
32103	0.6546107288	0.2895110831	0.02684136589	0	0.02764263604	0.001394186172	0.001394186172
32130	0.6696210426	0.2709408024	0.02984502917	0	0.02959312584	0	0
32132	0.9198969344	0	0.03726804709	0	0.04283501846	0	0
32187	0.6566758806	0.2828889796	0.03420735683	0	0.02622778295	0	0
32207	0.5523143254	0.4118317177	0.01311493737	0	0.02273901961	0	0
32209	0.5753928817	0.3686373746	0.03143772393	0	0.0245320197	0	0
32215	0.9383229206	0	0.02526079925	0	0.03641628018	0	0
32224	0.7737649351	0.1638755245	0.03490280686	0	0.02745673354	0	0
32227	0.9464992862	0	0.0174227131	0	0.03607800075	0	0
32298	0.9196759564	0.01637286819	0.02584583616	0	0.03810533923	0	0
32299	0.9473863018	0	0.01652531897	0	0.03608837923	0	0

ID	P. anubis	P. cynocephalu	P.hamadryas	P. kindae	P. papio	P. ursinus	P. kindae + P. ursinus
32311	0.9274658921	0.000904077193	0.03161038854	0	0.04001964216	0	0
32356	0.6114385203	0.3134854478	0.04352317681	0	0.03155285509	0	0
32358	0.7031899728	0.2418047371	0.0303140581	0	0.02469123205	0	0
32363	0.9672438292	0	0.007416315168	0	0.02533985562	0	0
32372	0.8053655665	0.1372498873	0.03081186513	0	0.02657268114	0	0
32451	0.7930829995	0.1407879418	0.03550168143	0	0.03062737723	0	0
32467	0.8412006224	0.08405592758	0.03412118137	0	0.04062226864	0	0
32469	0.7774745757	0.1854174099	0.0123252017	0	0.02478281276	0	0
32472	0.8263407095	0.136048049	0.01583895547	0	0.02177228597	0	0
32539	0.7104058278	0.2293805436	0.02749571124	0	0.03271791736	0	0
32540	0.8121743917	0.13303936	0.02816990064	0	0.02661634767	0	0
32552	0.8292758216	0.1103771746	0.02957031762	0	0.03077668611	0	0
32553	0.6912734475	0.2550774592	0.0236774614	0.00E+00	0.02997163194	0	0.00E+00
32557	0.6996208997	0.2552669482	0.02012058722	0	0.02499156489	0	0
32560	0.9342092835	0	0.02885015354	0	0.03694056293	0	0
32588	0.892440862	0.0389340543	0.03625779408	0	0.03236728958	0	0
32591	0.7843127938	0.1363153785	0.04210935354	0	0.03726247421	0	0
32606	0.7083822727	0.1097139844	0.1372024365	0	0.04470130639	0	0
32629	0.8047316352	0.1284317691	0.03366276486	0	0.0331738309	0	0
32651	0.9423924739	0	0.02291852137	0	0.03468900476	0	0
32736	0.7731069549	0.1740071366	0.02253851458	0	0.03034739392	0	0
32772	0.9649510623	0	0.01312018538	0	0.02192875229	0	0
32773	0.8691466025	0.04935208886	0.03987265035	0	0.04162865828	0	0
32785	0.8280097419	0.114375525	0.02555821763	0	0.03205651543	0	0
32794	0.8123176338	0.1057400107	0.04181672386	0	0.04012563163	0	0
32797	0.7017049695	0.2398988131	0.02726805581	0	0.03112816163	0	0
32801	0.8392059992	0.09602263077	0.03201531453	0	0.03275605549	0	0
32849	0.8190559295	0.09525829934	0.04400365128	0	0.04168211992	0	0
32860	0.9448363022	0.00510904791	0.02441136594	0	0.02564328395	0	0
32863	0.6582133863	0.2815315903	0.03484167203	0	0.02541335133	0	0
32876	0.6647630746	0.2883395955	0.02344173716	0	0.02345559267	0	0
32906	0.5137057488	0.4215819782	0.03727847028	0	0.0274338027	0	0
32909	0.9138134102	0.03981831432	0.02074120498	0	0.02562707052	0	0
32988	0.8088031982	0.1279903534	0.03425704221	0	0.02894940615	0	0
32993	0.9455965028	0	0.01680766522	0	0.03759583198	0	0
32995	0.889661614	0.070764822	0.0204896284	0	0.01908393561	0	0
32996	0.6222248193	0.3136193541	0.03649460176	0	0.02766122486	0	0
33082	0.5973678349	0.3289795243	0.03958059463	0	0.03407204623	0	0
33083	0.8369269496	0.08152540546	0.0395827867	0	0.04196485823	0	0
33097	0.8220806263	0.122039007	0.02839007224	0	0.02749029449	0	0
33106	0.8337377305	0.08644059087	0.04348357435	0	0.03633810432	0	0
33112	0.6721080037	0.2650414023	0.03138305583	0	0.03146753809	0	0
33115	0.9279077443	0	0.03144708253	0	0.04064517322	0	0
33163	0.6527058357	0.2910368602	0.02877872223	0	0.02666678689	0.000811794913	0.000811794913
33524	0.7896911365	0.1453041775	0.03116103621	0	0.03384364978	0	0
33545	0.8781561974	0.03990337432	0.04203309471	0	0.03990733356	0	0
33599	0.6807847662	0.2599359707	0.03354927424	0	0.0257299889	0	0
33605	0.7000149033	0.2368699089	0.02841207148	0	0.03470311624	0	0
33631	0.8286757845	0.1251097824	0.02474524655	0	0.02146918661	0	0
33863	0.8536263912	0.03501835984	0.06342370981	0	0.04793153916	0	0
33874	0.8248070391	0.0982924025	0.04067893436	0	0.03622162407	0	0
33889	0.9458885845	0	0.02025011849	0	0.03386129705	0	0
33908	0.8632885645	0.05889129363	0.0426920523	0	0.03462903017	0.000499059410	0.000499059410
34568	0.5682523083	0.3645836096	0.03497231793	0	0.03219176422	0	0
34857	0.8091609256	0.1111385434	0.04055370605	0	0.03914682491	0	0
34859	0.9271425302	0	0.03407472098	0	0.03878274881	0	0
34897	0.9386099789	0	0.02599313793	0	0.03539688316	0	0
6265	0.8914165247	0.006593654427	0.05926522677	0.001656670508	0.0410679236	0	0.001656670508
6382	0.9990531901	0	0	0	0.000946809850	0	0
6716	0.64692781	0.3142057386	0.02001271656	0.003056288141	0.01579744669	0	0.003056288141
6955	0.8837543232	0.007912713582	0.06131229845	0.00428090773	0.04273975699	0	0.00428090773
7091	0.9024501897	0	0.05359627864	0	0.0439535317	0	0
7158	0.4367076436	0.5632923564	0	0	0	0	0

ID	P. anubis	P. cynocephalı	P.hamadryas	P. kindae	P. papio	P. ursinus	P. kindae + P. ursinus
7267	0.8623170007	0	0.08521456109	0	0.05246843819	0	0
7311	0.9427279714	0	0.02760142507	0	0.02967060354	0	0
7478	0.9662770348	0	0.005951889939	0	0.02777107525	0	0
7625	0.8892426232	0.004899774248	0.05985426294	0.003792651922	0.04221068769	0	0.003792651922
7777	0.8981382506	0	0.04996303711	0	0.05189871229	0	0
7790	0.9047595502	0	0.04695376691	0	0.04828668289	0	0
7937	0.9428956934	0	0.01903473444	0	0.03806957219	0	0
8134	0.9113138134	0	0.04251036189	0	0.04617582468	0	0
8170	0.9351601838	0	0.03337956562	0	0.03146025061	0	0
8307	0.9436824953	0	0.02611813612	0	0.03019936856	0	0
8344	0.4959600929	0.4765248919	0.008916131797	0	0.01859888342	0	0
8395	0.8961394541	0.006125944211	0.05783637946	0.001547288278	0.03835093392	0	0.001547288278
8465	0.7052736836	0.2246049481	0.04057888654	0.001602585568	0.02654593429	0.001393961973	0.002996547541
8581	0.7610798525	0.1924480908	0.02039564334	0.001882708597	0.02419370473	0	0.001882708597
8596	0.7697208043	0.1726027958	0.02107095857	0	0.03660544129	0	0
8635	0.9010700761	0	0.0514517893	0	0.0474781346	0	0
8653	0.9067167431	0	0.04779278875	0	0.04549046815	0	0
8780	0.9140495605	0	0.05000729833	0	0.03594314119	0	0
8995	0.8584664069	0	0.1084849112	0	0.03304868183	0	0
9045	0.943587723	0	0.02517338939	0	0.03123888759	0	0
9086	0.5954136449	0.3597793315	0.02121858513	0	0.02358843847	0	0
9128	0.8970686585	0.005723039873	0.05646149226	0.001551427333	0.03919538202	0	0.001551427333
9481	0.2751391597	0.703097733	0.00983714993	0	0.01192595739	0	0
9514	0.9038018082	0	0.04525935121	0	0.05093884063	0	0
9562	0.9588374184	0	0.007413613259	0	0.03374896834	0	0
9656	0.2300653724	0.7510991581	0.01074407455	0	0.008091394959	0	0
9841	0.9300642713	0	0.02791927139	0	0.04201645728	0	0
9860	0.6878406076	0.2437177998	0.02256615064	0.01120653346	0.02556031244	0.009108595985	0.02031512945
9878	0.9562964528	0	0.00644984903	0	0.03725369813	0	0

FID1	ID1	FID2	ID2	N_SNP	HetHet	IBS0	HetConc	HomIBS0	Kinship	IBD1Seg	IBD2Seg	PropIBD	InfType
1X0576	1X0576	1X0580	1X0580	4023297	0.0979	0.043	0.2694	0.3479	0.0252	0.1169	0	0.0584	4th
1X2054	1X2054	1X1960	1X1960	4023297	0.1011	0.0444	0.268	0.3802	0.0239	0.1827	0.0026	0.0939	3rd
14712	14712	1X0026	1X0026	4023297	0.1017	0.045	0.2626	0.3865	0.0224	0.0661	0	0.0331	UN
1X0102	1X0102	1X0110	1X0110	4023297	0.1426	0.0656	0.273	0.1769	0.0134	0.142	0.0023	0.0733	4th
1X0014	1X0014	1X0035	1X0035	4023297	0.1078	0.0383	0.2746	0.3087	0.0584	0.2762	0.0042	0.1423	3rd
1X2054	1X2054	1X2055	1X2055	4023297	0.1025	0.0427	0.2686	0.3744	0.0314	0.1815	0.0018	0.0926	3rd
1X2054	1X2054	1X2891	1X2891	4023297	0.1004	0.0458	0.2599	0.399	0.0115	0.0857	0.0023	0.0452	4th
1X1960	1X1960	1X2055	1X2055	4023297	0.1044	0.0384	0.2763	0.3333	0.0521	0.2701	0.0045	0.1396	3rd
1Y0291	1Y0291	1X1181	1X1181	4023297	0.0993	0.0443	0.2602	0.3836	0.012	0.0997	0	0.0498	4th
1Y0291	1Y0291	10192	10192	4023297	0.1031	0.0425	0.2624	0.3853	0.0319	0.0433	0	0.0216	UN
1X0102	1X0102	10349	10349	4023297	0.152	0.0564	0.2758	0.1864	0.0338	0.652	0.0883	0.4143	FS
1X4811	1X4811	10488	10488	4023297	0.1511	0.0003	0.238	0.0009	0.0671	0.9976	0	0.4988	PO
1X0014	1X0014	1X1392	1X1392	4023297	0.1051	0.0439	0.2659	0.348	0.0301	0.1594	0	0.0797	4th
1X0035	1X0035	1X1392	1X1392	4023297	0.1068	0.0451	0.2684	0.3597	0.0329	0.1457	0.0013	0.0742	4th
1X0351	1X0351	1X3321	1X3321	4023297	0.1857	0.0138	0.3016	0.0588	0.1937	0.5152	0	0.2576	2nd
1X0351	1X0351	10418	10418	4023297	0.167	0.0686	0.2687	0.4162	0.0242	0	0	0	UN
1X0351	1X0351	10488	10488	4023297	0.2179	0.0466	0.3142	0.3508	0.0917	0	0	0	UN
1X0832	1X0832	10489	10489	4023297	0.1239	0.0262	0.298	0.2664	0.1174	0.4524	0	0.2262	2nd
1X0832	1X0832	10987	10987	4023297	0.1219	0.0279	0.2924	0.2806	0.1074	0.4346	0	0.2173	2nd
1X2054	1X2054	12242	12242	4023297	0.114	0.028	0.2955	0.2735	0.1005	0.4531	0.0038	0.2304	2nd
1X0153	1X0153	12242	12242	4023297	0.1062	0.0453	0.2622	0.4283	0.0227	0.0455	0	0.0228	UN
1X0351	1X0351	11959	11959	4023297	0.1714	0.0708	0.2715	0.4124	0.0324	0	0	0	UN
1X0351	1X0351	11981	11981	4023297	0.1692	0.0435	0.2764	0.3406	0.0898	0	0	0	UN
1X1032	1X1032	11887	11887	4023297	0.1089	0.0465	0.2639	0.4155	0.0229	0.0371	0	0.0185	UN
1X2054	1X2054	13463	13463	4023297	0.1019	0.0361	0.2709	0.3264	0.0601	0.2568	0	0.1284	3rd
1X0832	1X0832	13698	13698	4023297	0.1015	0.0219	0.281	0.187	0.0736	0.6762	0.0116	0.3497	2nd
1X0832	1X0832	13739	13739	4023297	0.0927	0.0295	0.2363	0.2353	0.0382	0.2091	0.0055	0.1101	3rd
1X0832	1X0832	13942	13942	4023297	0.1043	0.0317	0.255	0.2676	0.0755	0.1375	0.0021	0.0708	4th
1X0832	1X0832	13951	13951	4023297	0.1054	0.0197	0.2869	0.1723	0.1016	0.6928	0.006	0.3524	2nd
1X0951	1X0951	13245	13245	4023297	0.1098	0.0405	0.2721	0.3595	0.0432	0.2449	0	0.1225	3rd
1X1032	1X1032	13644	13644	4023297	0.1322	0.0218	0.2799	0.2078	0.0841	0.6053	0	0.3026	2nd
1X1125	1X1125	13110	13110	4023297	0.0982	0.0296	0.2715	0.2637	0.0529	0.0576	0	0.0288	UN
1X1125	1X1125	13644	13644	4023297	0.1236	0.018	0.2628	0.1716	0.0752	0.6444	0	0.3222	2nd
1X0351	1X0351	14182	14182	4023297	0.1678	0.0692	0.2679	0.4461	0.0271	0	0	0	UN
1X0832	1X0832	14012	14012	4023297	0.1119	0.0272	0.2912	0.2461	0.0984	0.5528	0.0104	0.2868	2nd
1X0832	1X0832	14068	14068	4023297	0.0963	0.0163	0.2674	0.1332	0.0836	0.7926	0.0056	0.4018	2nd
1X0832	1X0832	14204	14204	4023297	0.1043	0.0282	0.2888	0.2394	0.0527	0.6067	0.0181	0.3214	2nd
1X0832	1X0832	14435	14435	4023297	0.1134	0.0225	0.2919	0.2104	0.1242	0.5983	0.0065	0.3056	2nd
1X0832	1X0832	14652	14652	4023297	0.1107	0.0134	0.3025	0.1222	0.1453	0.7758	0.0206	0.4085	2nd
1X0832	1X0832	14756	14756	4023297	0.1011	0.0221	0.2805	0.1834	0.0709	0.6961	0.0128	0.3609	2nd
1X1125	1X1125	14668	14668	4023297	0.0992	0.035	0.2739	0.298	0.0315	0.1139	0.0037	0.0606	4th
1X0580	1X0580	15190	15190	4023297	0.1028	0.0395	0.2756	0.3293	0.0361	0.4963	0	0.2482	2nd
1X1947	1X1947	15107	15107	4023297	0.0991	0.0313	0.2752	0.259	0.0763	0.5386	0	0.2693	2nd
1X4811	1X4811	15232	15232	4023297	0.1414	0.0396	0.3062	0.1129	0.0725	0.3575	0.1574	0.3361	FS
1X0351	1X0351	15113	15113	4023297	0.1668	0.0594	0.2684	0.4263	0.0478	0	0	0	UN
1X1032	1X1032	14925	14925	4023297	0.1134	0.0304	0.302	0.2631	0.0867	0.5941	0.0044	0.3015	2nd
1X1125	1X1125	14925	14925	4023297	0.1101	0.0277	0.2988	0.2395	0.1025	0.6484	0.0039	0.3281	2nd
1X1125	1X1125	15190	15190	4023297	0.1141	0.0268	0.3024	0.2414	0.1222	0.5664	0.0202	0.3034	2nd
1X1947	1X1947	15421	15421	4023297	0.1126	0.0236	0.3243	0.2039	0.1399	0.545	0.0911	0.3636	FS
1X1032	1X1032	15824	15824	4023297	0.1038	0.0371	0.2797	0.3074	0.0236	0.35	0.0073	0.1823	2nd
1X2054	1X2054	16413	16413	4023297	0.1069	0.0375	0.276	0.3402	0.052	0.352	0	0.176	3rd
1X2054	1X2054	16517	16517	4023297	0.1043	0.0399	0.2687	0.3613	0.0384	0.2879	0.0017	0.1457	3rd
1X0832	1X0832	16692	16692	4023297	0.0992	0.0311	0.2671	0.273	0.0316	0.3152	0.0023	0.1599	3rd
1X0832	1X0832	16702	16702	4023297	0.1074	0.0274	0.2811	0.2475	0.0832	0.5127	0.0019	0.2582	2nd
1X4811	1X4811	16862	16862	4023297	0.1396	0.0484	0.2951	0.1416	0.0297	0.5803	0.1774	0.4675	FS
1X2054	1X2054	17199	17199	4023297	0.103	0.0383	0.2718	0.3365	0.0531	0.3816	0	0.1908	2nd
1X4811	1X4811	17833	17833	4023297	0.1442	0.0453	0.2961	0.136	0.0322	0.1306	0.1814	0.2467	2nd
1X0351	1X0351	17295	17295	4023297	0.1663	0.062	0.2678	0.4295	0.0399	0	0	0	UN
1X1947	1X1947	17981	17981	4023297	0.0927	0.0326	0.2698	0.2603	0.0351	0.1526	0.0105	0.0868	4th
1X2054	1X2054	19181	19181	4023297	0.1033	0.0424	0.2666	0.3791	0.0274	0.2814	0.0021	0.1428	3rd
1X2054	1X2054	19348	19348	4023297	0.1023	0.0411	0.2695	0.3585	0.04	0.3205	0.0017	0.162	3rd
1X0351	1X0351	18866	18866	4023297	0.1585	0.0521	0.2647	0.377	0.039	0	0	0	UN
1X2054	1X2054	1X3656	1X3656	4023297	0.1297	0.0001	0.3531	0.0009	0.2522	0.9966	0.0027	0.501	PO
1X2054	1X2054	1X4179	1X4179	4023297	0.1038	0.0421	0.2626	0.3883	0.0213	0.1143	0	0.0572	4th
1X1960	1X1960	1X4179	1X4179	4023297	0.1037	0.0418	0.2634	0.3796	0.0203	0.13	0	0.065	4th
1X0153	1X0153	1X4080	1X4080	4023297	0.1076	0.0439	0.2639	0.4029	0.0266	0.0606	0	0.0303	UN
1X0102	1X0102	1X3837	1X3837	4023297	0.169	0.0002	0.2615	0.0008	0.1431	0.9981	0	0.499	PO
1X0026	1X0026	1X3656	1X3656	4023297	0.1038	0.0435	0.2604	0.4033	0.0223	0.0261	0	0.0131	UN
1X0026	1X0026	1X3837	1X3837	4023297	0.1312	0.0001	0.2207	0.0014	0.0274	0.9966	0	0.4983	PO
1X0351	1X0351	1X3796	1X3796	4023297	0.2211	0.0266	0.3359	0.2132	0.1648	0	0	0	UN
1X0351	1X0351	1X3837	1X3837	4023297	0.2065	0.0485	0.3039	0.363	0.0887	0	0	0	UN
1X0351	1X0351	1X4209	1X4209	4023297	0.2072	0.0484	0.3055	0.365	0.0903	0	0	0	UN
1X0354	1X0354	1X4080	1X4080	4023297	0.1286	0.0001	0.3431	0.0009	0.2424	0.9981	0	0.499	PO
1X0812	1X0812	1X2124	1X2124	4023297	0.1204	0.0003	0.31	0.0025	0.1974	0.9962	0	0.4981	PO
1X0832	1X0832	1X2816	1X2816	4023297	0.1421	0.0001	0.3722	0.0007	0.2685	0.9695	0.0297	0.5145	PO
1X0832	1X0832	1X4080	1X4080	4023297	0.1092	0.0474	0.2637	0.4403	0.0226	0.0283	0	0.0141	UN
1X0832	1X0832	1X4777	1X4777	4023297	0.1114	0.0468	0.269	0.4415	0.0274	0.0339	0	0.0169	UN
1X1032	1X1032	1X2231	1X2231	4023297	0.1066	0.0472	0.2612	0.4256	0.0225	0.0506	0	0.0253	UN
1X1032	1X1032	1X2816	1X2816	4023297	0.1086	0.0465	0.263	0.4447	0.0225	0.038	0	0.019	UN
1X1032	1X1032	1X4080	1X4080	4023297	0.1093	0.0462	0.2657	0.4205	0.0257	0.0465	0	0.0232	UN
1X1032	1X1032	1X4179	1X4179	4023297	0.1066	0.047	0.2607	0.4342	0.0224	0.0042	0	0.0021	UN
1X1032	1X1032	1X4777	1X4777	4023297	0.1096	0.0463	0.2651	0.4293	0.0237	0.0396	0	0.0198	UN
1X1125	1X1125	1X1155	1X1155	4023297	0.0993	0.0453	0.2568	0.3842	0.0111	0.1473	0.0018	0.0754	4th
1X1125	1X1125	1X2231	1X2231	4023297	0.1339	0	0.3614	0.0005	0.2597	0.9491	0.0493	0.5238	PO
1X2054	1X2054	26988	26988	4023297	0.1028	0.041	0.2688	0.363	0.0381	0.3256	0	0.1628	3rd
1X0351	1X0351	26498	26498	4023297	0.1978	0.0416	0.3028	0.3957	0.1165	0	0	0	UN
1X1032	1X1032	26498	26498	4023297	0.154	0.0223	0.2809	0.2683	0.0294	0.0659	0	0.033	UN
1X1125	1X1125	26800	26800	4023297	0.095	0.0329	0.2647	0.278	0.0234	0.128	0.004	0.068	4th
1X1125	1X1125	27351	27351	4023297	0.109	0.0317	0.2568	0.2699	0.0512	0.5039	0.0058	0.2578	2nd
1X2054	1X2054	28246	28246	4023297	0.107	0.0362	0.2745	0.3433	0.0545	0.3393	0.0021	0.1717	3rd
1X0102	1X0102	28281	28281	4023297	0.1495	0.0593	0.3047	0.1957	0.026	0.184	0.1151	0.2071	2nd
1X1032	1X1032	28279	28279	4023297	0.1167	0.0391	0.2601	0.3438	0.0242	0.3165	0.0047	0.1629	3rd
1X1125	1X1125	28279	28279	4023297	0.1149	0.0299	0.261	0.2663	0.0485	0.4499	0.0034	0.2284	2nd
1X0351	1X0351	30917	30917	4023297	0.1647	0.0468	0.2738	0.4275	0.0674	0	0	0	UN
1X0351	1X0351	30920	30920	4023297	0.1844	0.0472	0.2907	0.4124	0.1062	0	0	0	UN
1X0832	1X0832	30917	30917	4023297	0.1326	0.034	0.2723	0.3945	0.0267	0.0056	0	0.0028	UN
1X1947	1X1947	31134	31134	4023297	0.1001	0.0324	0.2674	0.2801	0.0634	0.0782	0	0.0391	UN
1X0832	1X0832	31011	31011	4023297	0.1202	0.0315	0.2455	0.3002	0.0219	0.2292	0.0012	0.1158	3rd

FID1	ID1	FID2	ID2	N_SNP	HetHet	IBS0	HetConc	HomIBS0	Kinship	IBD1Seg	IBD2Seg	PropIBD	InfType
1X1125	1X1125	31021	31021	4023297	0.1131	0.0364	0.2517	0.334	0.0111	0.2589	0	0.1295	3rd
1X1125	1X1125	31134	31134	4023297	0.1006	0.0402	0.2588	0.3551	0.0382	0.0607	0	0.0303	UN
1X0832	1X0832	31624	31624	4023297	0.1034	0.0397	0.2553	0.3624	0.0386	0.0554	0	0.0277	UN
1X0351	1X0351	32089	32089	4023297	0.1622	0.0619	0.2668	0.3786	0.0248	0	0	0	UN
1X0832	1X0832	32040	32040	4023297	0.1135	0.0392	0.2616	0.3696	0.0392	0.2624	0.0014	0.1326	3rd
1X1947	1X1947	7937	7937	4023297	0.1106	0.0312	0.2936	0.2839	0.0771	0.4469	0.0045	0.228	2nd
1X0102	1X0102	7158	7158	4023297	0.17	0.042	0.3282	0.1415	0.115	0.5777	0.2024	0.4912	FS
1X0351	1X0351	34568	34568	4023297	0.1643	0.0586	0.2701	0.3701	0.038	0	0	0	UN
1X0351	1X0351	6716	6716	4023297	0.18	0.056	0.2814	0.4304	0.0786	0	0	0	UN
1X0351	1X0351	8344	8344	4023297	0.1744	0.0727	0.2729	0.4263	0.0343	0	0	0	UN
1X0354	1X0354	7311	7311	4023297	0.1114	0.0328	0.2839	0.3013	0.0697	0.3886	0	0.1943	2nd
1X0354	1X0354	8307	8307	4023297	0.1128	0.0325	0.2889	0.2996	0.0741	0.4478	0	0.2239	2nd
1X0356	1X0356	7267	7267	4023297	0.1232	0.0003	0.3162	0.0029	0.2097	0.986	0	0.493	PO
1X0808	1X0808	7937	7937	4023297	0.1068	0.0429	0.2698	0.3916	0.033	0.1264	0	0.0632	4th
1X0832	1X0832	6265	6265	4023297	0.1229	0.0279	0.2969	0.2814	0.1114	0.4436	0	0.2218	2nd
1X0832	1X0832	6955	6955	4023297	0.1214	0.0329	0.2897	0.3271	0.0857	0.3468	0	0.1734	3rd
1X0832	1X0832	7267	7267	4023297	0.126	0.0241	0.3035	0.2453	0.1278	0.548	0	0.274	2nd
1X0832	1X0832	7311	7311	4023297	0.122	0.0239	0.3041	0.2438	0.1381	0.4848	0.0045	0.2469	2nd
1X0832	1X0832	7625	7625	4023297	0.1251	0.0241	0.3029	0.2452	0.129	0.5241	0	0.2621	2nd
1X0832	1X0832	8170	8170	4023297	0.1285	0.017	0.3221	0.1805	0.1732	0.6668	0	0.3334	2nd
1X0832	1X0832	8307	8307	4023297	0.1243	0.0237	0.3118	0.2425	0.1439	0.5001	0.0058	0.2558	2nd
1X0832	1X0832	8395	8395	4023297	0.1273	0.0195	0.3093	0.2007	0.1504	0.5854	0	0.2927	2nd
1X0843	1X0843	7091	7091	4023297	0.1306	0.0001	0.3378	0.0006	0.2361	0.9986	0	0.4993	PO
1X1032	1X1032	7311	7311	4023297	0.1093	0.0462	0.2656	0.4317	0.0256	0.0512	0.0016	0.0271	UN
1X1032	1X1032	8170	8170	4023297	0.1092	0.0456	0.2626	0.4344	0.0237	0.0386	0.0016	0.0209	UN
1X1032	1X1032	8307	8307	4023297	0.1091	0.0466	0.2651	0.4351	0.0241	0.0494	0.0015	0.0262	UN
1X0580	1X0580	9562	9562	4023297	0.124	0.0001	0.3261	0.0013	0.2219	0.9933	0	0.4967	PO
1X2054	1X2054	9841	9841	4023297	0.1184	0.0176	0.3082	0.1776	0.1497	0.5931	0.0055	0.302	2nd
1X0153	1X0153	9841	9841	4023297	0.1059	0.0441	0.2594	0.4185	0.0241	0.0428	0	0.0214	UN
1X2049	1X2049	9656	9656	4023297	0.1784	0.0325	0.3164	0.1048	0.1001	0.5396	0	0.2698	2nd
1X2304	1X2304	9481	9481	4023297	0.1185	0.0366	0.2477	0.1071	0.072	0.2253	0	0.1127	3rd
1X4811	1X4811	9656	9656	4023297	0.1524	0.0003	0.2782	0.0008	0.1462	0.9993	0	0.4997	PO
1X0351	1X0351	8465	8465	4023297	0.1674	0.0568	0.2702	0.4423	0.0547	0	0	0	UN
1X0351	1X0351	9860	9860	4023297	0.171	0.0568	0.2761	0.4249	0.0611	0	0	0	UN
1X0354	1X0354	9045	9045	4023297	0.1186	0.0194	0.3094	0.1834	0.1426	0.6623	0	0.3311	2nd
1X0832	1X0832	9045	9045	4023297	0.1237	0.0208	0.311	0.212	0.1552	0.5577	0.0056	0.2844	2nd
1X0832	1X0832	9128	9128	4023297	0.1266	0.0176	0.3076	0.183	0.1571	0.6394	0	0.3197	2nd
1X1126	1X1126	10842	10842	4023297	0.1021	0.0137	0.2898	0.1208	0.1264	0.0465	0	0.0233	UN
1X1672	1X1672	10099	10099	4023297	0.0978	0.0058	0.3376	0.0468	0.1398	0.5314	0.0592	0.3249	2nd
1X1672	1X1672	10164	10164	4023297	0.1054	0.0178	0.2763	0.1388	0.1356	0.3587	0.0507	0.2301	2nd
1X1765	1X1765	10173	10173	4023297	0.1363	0.0001	0.3453	0.0012	0.2474	0.9984	0	0.4992	PO
1X1939	1X1939	10316	10316	4023297	0.0952	0.0138	0.2886	0.119	0.1117	0.0688	0	0.0344	UN
1X1958	1X1958	10418	10418	4023297	0.1671	0.0718	0.2796	0.3639	0.0297	0	0	0	UN
1X1958	1X1958	10488	10488	4023297	0.2194	0.0437	0.3285	0.2599	0.0916	0.029	0	0.0145	UN
1X3321	1X3321	10488	10488	4023297	0.214	0.0532	0.3111	0.2315	0.0664	0.0055	0	0.0027	UN
1X4519	1X4519	10489	10489	4023297	0.136	0.0001	0.3403	0.0007	0.2413	0.9986	0	0.4993	PO
1X4519	1X4519	10987	10987	4023297	0.1359	0.0001	0.3408	0.0008	0.2419	0.9979	0	0.4989	PO
10173	10173	10489	10489	4023297	0.1167	0.0442	0.266	0.4573	0.0463	0.0357	0	0.0179	UN
10173	10173	10987	10987	4023297	0.1175	0.0437	0.2687	0.4518	0.0499	0.0526	0	0.0263	UN
10349	10349	10488	10488	4023297	0.208	0.053	0.3123	0.3196	0.0451	0.0113	0	0.0057	UN
10418	10418	10488	10488	4023297	0.2643	0.0245	0.4229	0.1776	0.2007	0.0184	0	0.0092	UN
10489	10489	10987	10987	4023297	0.1896	0.0089	0.5112	0.1145	0.3064	0.4459	0.3577	0.5807	FS
1X1765	1X1765	10998	10998	4023297	0.0947	0.0166	0.2716	0.1485	0.072	0.031	0	0.0155	UN
1X1765	1X1765	11608	11608	4023297	0.0919	0.0164	0.2719	0.1462	0.0521	0.0378	0	0.0189	UN
1X1765	1X1765	12242	12242	4023297	0.137	0.0001	0.3617	0.0007	0.2638	0.993	0.0048	0.5013	PO
1X3162	1X3162	11752	11752	4023297	0.0915	0.0306	0.2635	0.257	0.0299	0.0608	0	0.0304	UN
10173	10173	10998	10998	4023297	0.1243	0.0204	0.3683	0.2028	0.1062	0.3526	0	0.1763	3rd
10173	10173	11887	11887	4023297	0.1118	0.0458	0.2612	0.4459	0.0287	0.0089	0	0.0044	UN
10173	10173	12242	12242	4023297	0.124	0.0252	0.3021	0.2733	0.1273	0.4709	0.0019	0.2373	2nd
10192	10192	11887	11887	4023297	0.112	0.0372	0.2778	0.3443	0.0604	0.217	0	0.1085	3rd
10349	10349	12218	12218	4023297	0.1627	0.0567	0.2965	0.3324	0.0537	0.0946	0	0.0473	4th
10418	10418	11959	11959	4023297	0.1705	0.069	0.2795	0.3836	0.0331	0	0	0	UN
10418	10418	11981	11981	4023297	0.1583	0.0639	0.2635	0.4498	0.0358	0	0	0	UN
10488	10488	11959	11959	4023297	0.2245	0.0446	0.3308	0.2977	0.1019	0.0165	0	0.0082	UN
10488	10488	11981	11981	4023297	0.2058	0.0417	0.3044	0.3829	0.0765	0.0056	0	0.0028	UN
10488	10488	12473	12473	4023297	0.2052	0.0478	0.3089	0.3185	0.0528	0.0049	0	0.0025	UN
10489	10489	11885	11885	4023297	0.1006	0.025	0.2689	0.2056	0.019	0.7085	0	0.3543	2nd
10489	10489	11887	11887	4023297	0.1135	0.0458	0.2627	0.4407	0.0267	0.0094	0	0.0047	UN
10489	10489	11981	11981	4023297	0.1307	0.0356	0.2485	0.3592	0.0208	0.2667	0.0013	0.1346	3rd
10987	10987	11885	11885	4023297	0.1	0.0255	0.2675	0.2094	0.0155	0.7068	0	0.3534	2nd
10987	10987	11887	11887	4023297	0.1139	0.0461	0.2642	0.4412	0.027	0.0033	0	0.0017	UN
1X1126	1X1126	13597	13597	4023297	0.0936	0.0186	0.2972	0.15	0.0324	0.3385	0.0315	0.2007	2nd
1X1126	1X1126	13644	13644	4023297	0.1215	0.0306	0.2549	0.2779	0.0234	0.4579	0.0013	0.2302	2nd
1X1126	1X1126	13694	13694	4023297	0.0964	0.0189	0.2989	0.1539	0.0518	0.3687	0.024	0.2084	2nd
1X1181	1X1181	13110	13110	4023297	0.0935	0.0322	0.2632	0.2782	0.0424	0.0493	0	0.0247	UN
1X1765	1X1765	13463	13463	4023297	0.1152	0.0246	0.3043	0.2399	0.1194	0.4974	0.0026	0.2513	2nd
1X2055	1X2055	13463	13463	4023297	0.1031	0.0364	0.2723	0.3292	0.0575	0.269	0.0042	0.1387	3rd
10173	10173	13245	13245	4023297	0.1109	0.0462	0.2592	0.4536	0.0246	0.0064	0	0.0032	UN
10173	10173	13463	13463	4023297	0.11	0.0369	0.2734	0.3634	0.0373	0.2471	0	0.1235	3rd
10488	10488	13644	13644	4023297	0.1886	0.0461	0.2835	0.4118	0.0249	0.0027	0	0.0013	UN
10489	10489	13245	13245	4023297	0.1131	0.0442	0.2623	0.4297	0.0313	0.0164	0	0.0082	UN
10489	10489	13698	13698	4023297	0.1025	0.0221	0.2687	0.1952	0.0487	0.6213	0.0012	0.3118	2nd
10489	10489	13914	13914	4023297	0.1066	0.0372	0.2348	0.3144	0.0572	0.0179	0	0.0089	UN
10489	10489	13942	13942	4023297	0.1109	0.0288	0.2616	0.2556	0.0789	0.1289	0	0.0644	4th
10489	10489	13951	13951	4023297	0.1092	0.029	0.2836	0.2573	0.0421	0.6137	0	0.3069	2nd
10987	10987	13245	13245	4023297	0.1136	0.0448	0.2642	0.4338	0.0302	0.0132	0	0.0066	UN
10987	10987	13698	13698	4023297	0.1035	0.0277	0.2723	0.241	0.0241	0.604	0.0012	0.3032	2nd
10987	10987	13914	13914	4023297	0.1051	0.041	0.231	0.3425	0.041	0.0143	0	0.0071	UN
10987	10987	13942	13942	4023297	0.107	0.0326	0.2508	0.2852	0.0569	0.1155	0	0.0577	4th
1X1672	1X1672	14058	14058	4023297	0.1044	0.02	0.3228	0.171	0.1056	0.3161	0.0281	0.1861	2nd
1X1672	1X1672	14062	14062	4023297	0.1	0.015	0.33	0.1246	0.1009	0.4044	0.0547	0.2569	2nd
1X1672	1X1672	14250	14250	4023297	0.0823	0.0152	0.271	0.1156	0.0221	0.4148	0.0104	0.2178	2nd
1X1672	1X1672	14350	14350	4023297	0.0924	0.0152	0.2952	0.1235	0.0789	0.3729	0.0288	0.2152	2nd
1X1672	1X1672	14351	14351	4023297	0.0912	0.0154	0.3105	0.1247	0.0495	0.4532	0.0147	0.2413	2nd
1X3576	1X3576	13988	13988	4023297	0.099	0.0218	0.3072	0.1815	0.0495	0.3097	0.0141	0.169	3rd
1X3576	1X3576	14022	14022	4023297	0.1231	0.0127	0.31	0.1215	0.1739	0.6873	0.1114	0.4551	FS
1X3576	1X3576	14324	14324	4023297	0.0951	0.0278	0.259	0.2298	0.0512	0.2308	0.0083	0.1237	3rd
1X4519	1X4519	14012	14012	4023297	0.1086	0.0363	0.2829	0.2888	0.0573	0.5971	0	0.2986	2nd

FID1	ID1	FID2	ID2	N_SNP	HetHet	IBS0	HetConc	HomIBS0	Kinship	IBD1Seg	IBD2Seg	PropIBD	InfType
10349	10349	14182	14182	4023297	0.1862	0.035	0.3263	0.2002	0.144	0.5956	0.0579	0.3556	2nd
10418	10418	14182	14182	4023297	0.1621	0.071	0.2659	0.4315	0.0225	0	0	0	UN
10418	10418	14276	14276	4023297	0.1621	0.0711	0.2697	0.4204	0.0242	0	0	0	UN
10488	10488	14172	14172	4023297	0.2071	0.0199	0.3397	0.1057	0.1121	0.3601	0.0012	0.1813	2nd
10488	10488	14182	14182	4023297	0.2131	0.0466	0.3126	0.3507	0.0788	0.0071	0	0.0036	UN
10488	10488	14276	14276	4023297	0.2138	0.046	0.318	0.3349	0.0775	0.0059	0	0.0029	UN
10489	10489	14012	14012	4023297	0.1439	0.0118	0.3848	0.1212	0.2081	0.6802	0.138	0.4781	FS
10489	10489	14204	14204	4023297	0.1043	0.0232	0.2724	0.2051	0.0504	0.6651	0	0.3326	2nd
10987	10987	14012	14012	4023297	0.142	0.0146	0.3783	0.1473	0.1929	0.6581	0.1296	0.4586	FS
1X1126	1X1126	14473	14473	4023297	0.1111	0.0186	0.3213	0.1669	0.1254	0.2107	0.0153	0.1207	3rd
1X1126	1X1126	14668	14668	4023297	0.1167	0.0197	0.3344	0.1795	0.1387	0.243	0.0465	0.168	3rd
1X1126	1X1126	14909	14909	4023297	0.0941	0.0231	0.2873	0.1857	0.0241	0.2754	0.0198	0.1575	3rd
1X1672	1X1672	14460	14460	4023297	0.1228	0.008	0.267	0.0726	0.1127	0.7541	0.1065	0.4836	FS
1X1672	1X1672	14526	14526	4023297	0.0915	0.024	0.2901	0.1951	0.0259	0.2623	0.0201	0.1512	3rd
1X1672	1X1672	14795	14795	4023297	0.1133	0.0158	0.3554	0.142	0.1533	0.3677	0.0663	0.2501	2nd
1X1765	1X1765	14850	14850	4023297	0.1169	0.0129	0.3372	0.1233	0.161	0.326	0.0499	0.2129	2nd
1X1765	1X1765	14922	14922	4023297	0.1015	0.0238	0.3005	0.2109	0.047	0.1867	0.0159	0.1093	3rd
1X2055	1X2055	14860	14860	4023297	0.1122	0.0232	0.352	0.1952	0.1005	0.522	0.1811	0.442	FS
1X3576	1X3576	14690	14690	4023297	0.1148	0.0176	0.3211	0.1673	0.1494	0.4467	0.1027	0.326	2nd
1X4519	1X4519	14435	14435	4023297	0.1118	0.0297	0.2894	0.2437	0.0955	0.6503	0	0.3251	2nd
10173	10173	14850	14850	4023297	0.13	0.0036	0.3691	0.0365	0.2149	0.4643	0.0351	0.2672	2nd
10173	10173	14922	14922	4023297	0.1081	0.0108	0.3093	0.0996	0.1111	0.1723	0	0.0861	4th
10489	10489	14435	14435	4023297	0.1455	0.0122	0.3847	0.1283	0.2106	0.6251	0.1367	0.4492	FS
10489	10489	14652	14652	4023297	0.1105	0.0255	0.285	0.2344	0.0644	0.6179	0	0.309	2nd
10489	10489	14756	14756	4023297	0.1243	0.0141	0.3464	0.128	0.1406	0.7048	0.1168	0.4692	FS
10987	10987	14435	14435	4023297	0.1457	0.0136	0.3862	0.1423	0.206	0.5902	0.1611	0.4562	FS
10987	10987	14652	14652	4023297	0.1112	0.0214	0.2879	0.198	0.0857	0.6662	0	0.3331	2nd
10987	10987	14696	14696	4023297	0.0957	0.0215	0.2385	0.182	0.0495	0.1788	0	0.0894	3rd
10987	10987	14756	14756	4023297	0.124	0.0158	0.3462	0.1422	0.1325	0.6874	0.1261	0.4699	FS
1X1126	1X1126	14925	14925	4023297	0.1218	0.0175	0.3375	0.1584	0.1673	0.5632	0.0939	0.3754	FS
1X1126	1X1126	15190	15190	4023297	0.1369	0.0127	0.3819	0.1241	0.2221	0.6156	0.1611	0.4689	FS
1X1126	1X1126	15267	15267	4023297	0.1053	0.0176	0.3138	0.1507	0.105	0.3475	0.0341	0.2079	2nd
1X1237	1X1237	15009	15009	4023297	0.1076	0.0303	0.2865	0.2461	0.0868	0.5344	0.0033	0.2705	2nd
1X1672	1X1672	15009	15009	4023297	0.127	0.0139	0.3539	0.1317	0.1985	0.653	0.1249	0.4514	FS
1X1672	1X1672	15150	15150	4023297	0.1261	0.0057	0.4074	0.0511	0.2385	0.6026	0.2955	0.5968	FS
1X1765	1X1765	14930	14930	4023297	0.1212	0.0188	0.3182	0.1853	0.1598	0.5995	0.05	0.3498	2nd
1X1765	1X1765	15211	15211	4023297	0.1206	0.0178	0.3382	0.1738	0.1528	0.2866	0.0708	0.2141	2nd
1X2891	1X2891	14930	14930	4023297	0.1109	0.028	0.2906	0.2553	0.1108	0.5251	0	0.2625	2nd
1X2891	1X2891	15212	15212	4023297	0.0983	0.0359	0.267	0.3201	0.0295	0.0684	0	0.0342	UN
1X3576	1X3576	14959	14959	4023297	0.1251	0.0179	0.3507	0.1758	0.1745	0.5118	0.1601	0.4161	FS
1X3576	1X3576	15156	15156	4023297	0.1197	0.0219	0.3068	0.2231	0.1405	0.5362	0.0254	0.2935	2nd
1X3576	1X3576	15212	15212	4023297	0.099	0.0367	0.2678	0.329	0.0254	0.0755	0	0.0378	UN
1X3576	1X3576	15274	15274	4023297	0.102	0.021	0.2873	0.1785	0.0952	0.3288	0.0331	0.1975	2nd
1Y1979	1Y1979	15156	15156	4023297	0.1115	0.0364	0.283	0.3328	0.0625	0.3531	0	0.1765	3rd
10173	10173	14930	14930	4023297	0.1407	0.0001	0.3701	0.0013	0.2562	0.9429	0.0555	0.527	PO
10173	10173	15211	15211	4023297	0.1365	0.0031	0.3799	0.0335	0.2338	0.4508	0.0682	0.2935	2nd
10349	10349	14951	14951	4023297	0.1655	0.0573	0.2929	0.2954	0.066	0.3946	0.005	0.2023	2nd
10418	10418	15113	15113	4023297	0.1758	0.0588	0.2979	0.3959	0.0756	0.0019	0	0.001	UN
10488	10488	14944	14944	4023297	0.2084	0.0418	0.3324	0.299	0.0554	0.0077	0	0.0038	UN
10488	10488	14951	14951	4023297	0.2012	0.0506	0.3017	0.3384	0.0387	0	0	0	UN
10488	10488	15113	15113	4023297	0.2351	0.0346	0.3597	0.2993	0.1363	0.0466	0	0.0233	UN
10488	10488	15197	15197	4023297	0.1994	0.0484	0.2974	0.3501	0.0433	0.0076	0	0.0038	UN
10488	10488	15232	15232	4023297	0.1965	0.0293	0.3106	0.1218	0.0717	0.4637	0	0.2318	2nd
1X1126	1X1126	15560	15560	4023297	0.0955	0.0248	0.2927	0.2008	0.019	0.2998	0.0149	0.1648	3rd
1X1672	1X1672	15494	15494	4023297	0.0957	0.0288	0.2371	0.216	0.0557	0.2579	0.0375	0.1664	3rd
1X1672	1X1672	15509	15509	4023297	0.1147	0.0212	0.3199	0.1958	0.1506	0.4602	0.0889	0.319	2nd
1X1672	1X1672	15571	15571	4023297	0.0941	0.0162	0.2938	0.1326	0.0874	0.3105	0.0141	0.1694	3rd
1X1672	1X1672	15575	15575	4023297	0.0964	0.0218	0.2962	0.1834	0.0692	0.2403	0.041	0.1612	3rd
1X1765	1X1765	15444	15444	4023297	0.1374	0.0083	0.3942	0.0851	0.2343	0.6183	0.2131	0.5222	FS
1X1765	1X1765	15458	15458	4023297	0.1382	0.0166	0.3689	0.1763	0.2046	0.5153	0.146	0.4037	FS
1X1765	1X1765	15475	15475	4023297	0.1072	0.0301	0.298	0.2772	0.0572	0.168	0.0038	0.0878	4th
1X3576	1X3576	15419	15419	4023297	0.1003	0.0187	0.2939	0.1634	0.0901	0.3673	0.0335	0.2171	2nd
1X3576	1X3576	15467	15467	4023297	0.1173	0.0081	0.328	0.0768	0.1984	0.7724	0.0746	0.4608	2nd
1X3576	1X3576	15562	15562	4023297	0.1082	0.0183	0.2598	0.1607	0.1172	0.346	0.0495	0.2225	2nd
10173	10173	15444	15444	4023297	0.1436	0.0001	0.3976	0.0012	0.263	0.8537	0.1437	0.5705	PO
10173	10173	15458	15458	4023297	0.1543	0.0002	0.4093	0.0024	0.2822	0.8866	0.1068	0.5501	PO
10173	10173	15475	15475	4023297	0.1347	0.0068	0.3842	0.0709	0.2114	0.2867	0.004	0.1474	3rd
10418	10418	15566	15566	4023297	0.1758	0.0521	0.3188	0.2441	0.0757	0.0162	0	0.0081	UN
10488	10488	15566	15566	4023297	0.2172	0.0266	0.3433	0.1376	0.1207	0.3075	0	0.1538	3rd
10488	10488	15599	15599	4023297	0.2069	0.0435	0.3316	0.3056	0.0451	0.0077	0	0.0038	UN
1X1126	1X1126	15659	15659	4023297	0.103	0.032	0.2729	0.2864	0.0624	0.0935	0	0.0468	4th
1X1126	1X1126	15824	15824	4023297	0.1196	0.0095	0.3428	0.0868	0.1933	0.5931	0.112	0.4086	FS
1X1126	1X1126	15870	15870	4023297	0.1099	0.0176	0.3327	0.1532	0.1172	0.2955	0.025	0.1728	3rd
1X1126	1X1126	15938	15938	4023297	0.096	0.0199	0.2943	0.1623	0.0491	0.3012	0.0168	0.1674	3rd
1X1126	1X1126	15940	15940	4023297	0.1093	0.0184	0.3168	0.1613	0.1207	0.296	0.0312	0.1792	2nd
1X1672	1X1672	15628	15628	4023297	0.1182	0.0092	0.3556	0.0814	0.2043	0.6508	0.1771	0.5025	FS
1X1672	1X1672	16019	16019	4023297	0.1109	0.0127	0.3381	0.1131	0.1661	0.2955	0.0952	0.243	2nd
1X1765	1X1765	15845	15845	4023297	0.1332	0.0147	0.37	0.1525	0.1986	0.3114	0.0975	0.2532	2nd
1X2055	1X2055	15659	15659	4023297	0.0981	0.0367	0.2611	0.3279	0.0387	0.078	0	0.039	UN
1X2664	1X2664	15845	15845	4023297	0.1049	0.0357	0.2736	0.3123	0.0554	0.0984	0	0.0492	4th
1X3576	1X3576	15944	15944	4023297	0.1219	0.0099	0.3397	0.0989	0.202	0.6311	0.1003	0.4159	FS
1X3576	1X3576	16056	16056	4023297	0.0967	0.0223	0.3074	0.1859	0.0275	0.3995	0.0113	0.2111	2nd
10173	10173	15845	15845	4023297	0.143	0.0028	0.3881	0.0314	0.25	0.4952	0.0802	0.3278	2nd
10842	10842	15824	15824	4023297	0.093	0.0389	0.2824	0.3132	0.02	0.0902	0	0.0451	4th
1X1126	1X1126	16321	16321	4023297	0.0955	0.0342	0.2318	0.255	0.0477	0.186	0.007	0.1	3rd
1X1672	1X1672	16244	16244	4023297	0.0939	0.0267	0.289	0.2236	0.0321	0.227	0.0271	0.1406	3rd
1X1672	1X1672	16261	16261	4023297	0.1133	0.0127	0.3381	0.1151	0.1757	0.3382	0.1202	0.2894	2nd
1X1672	1X1672	16338	16338	4023297	0.0947	0.0156	0.2954	0.1305	0.0927	0.2533	0.0332	0.1598	3rd
1X1672	1X1672	16371	16371	4023297	0.1012	0.0231	0.2626	0.1773	0.105	0.3634	0.0446	0.2263	2nd
1X3162	1X3162	16372	16372	4023297	0.1181	0.0116	0.3526	0.1067	0.1945	0.5543	0.1487	0.4259	FS
1X3576	1X3576	16083	16083	4023297	0.1024	0.0205	0.2964	0.1847	0.091	0.2123	0.0106	0.1168	3rd
1X3576	1X3576	16104	16104	4023297	0.1016	0.0209	0.2962	0.1818	0.0847	0.2404	0.0171	0.1373	3rd
1X3576	1X3576	16254	16254	4023297	0.0941	0.0308	0.2428	0.2497	0.0526	0.1801	0.0016	0.0916	3rd
10418	10418	16115	16115	4023297	0.1584	0.0439	0.309	0.186	0.0401	0.0036	0	0.0018	UN
10489	10489	16369	16369	4023297	0.1056	0.0424	0.2307	0.3602	0.035	0.0694	0	0.0347	UN
10987	10987	16369	16369	4023297	0.1048	0.0442	0.2288	0.3726	0.0266	0.0773	0	0.0387	UN
1X1126	1X1126	16409	16409	4023297	0.1033	0.0185	0.307	0.158	0.0941	0.3211	0.0127	0.1733	3rd
1X1126	1X1126	16527	16527	4023297	0.1111	0.0249	0.3082	0.2209	0.1054	0.2682	0.0612	0.1953	2nd

FID1	ID1	FID2	ID2	N_SNP	HetHet	IBS0	HetConc	HomIBS0	Kinship	IBD1Seg	IBD2Seg	PropIBD	InfType
1X1672	1X1672	16524	16524	4023297	0.0894	0.0192	0.27	0.153	0.0629	0.2963	0.018	0.1661	3rd
1X1672	1X1672	16538	16538	4023297	0.0911	0.018	0.3009	0.143	0.0448	0.4148	0.0324	0.2397	2nd
1X1672	1X1672	16708	16708	4023297	0.0874	0.0213	0.2852	0.1688	0.0121	0.3673	0.026	0.2096	2nd
1X1672	1X1672	16741	16741	4023297	0.1075	0.0116	0.3362	0.0991	0.1583	0.3865	0.029	0.2223	2nd
1X1765	1X1765	16408	16408	4023297	0.11	0.0197	0.3327	0.1786	0.0947	0.3663	0.0151	0.1983	2nd
1X1765	1X1765	16413	16413	4023297	0.1313	0.015	0.3465	0.1554	0.1974	0.5913	0.1057	0.4013	FS
1X1765	1X1765	16517	16517	4023297	0.133	0.0143	0.3543	0.1508	0.2031	0.6061	0.0993	0.4023	FS
1X1765	1X1765	16548	16548	4023297	0.1029	0.0238	0.294	0.2122	0.0645	0.2268	0.0091	0.1225	3rd
1X2055	1X2055	16510	16510	4023297	0.1118	0.0111	0.3346	0.1005	0.1698	0.3857	0.0876	0.2804	2nd
10046	10046	16527	16527	4023297	0.0873	0.0227	0.2693	0.1854	0.0697	0.006	0	0.003	UN
10173	10173	16408	16408	4023297	0.1094	0.007	0.3124	0.0657	0.136	0.2984	0	0.1492	3rd
10173	10173	16413	16413	4023297	0.1612	0.0001	0.4385	0.0009	0.2965	0.8239	0.1727	0.5847	PO
10173	10173	16517	16517	4023297	0.1557	0.0001	0.4194	0.0007	0.2861	0.8576	0.1381	0.5669	PO
10173	10173	16548	16548	4023297	0.1188	0.0061	0.3372	0.0593	0.1716	0.3282	0.0025	0.1666	3rd
10488	10488	16424	16424	4023297	0.1991	0.0348	0.3267	0.1853	0.0458	0.1992	0	0.0996	3rd
10488	10488	16743	16743	4023297	0.2125	0.0374	0.3316	0.1836	0.0841	0.263	0	0.1315	3rd
10489	10489	16671	16671	4023297	0.1	0.0394	0.225	0.328	0.0246	0.1281	0	0.0641	4th
10842	10842	16527	16527	4023297	0.0907	0.0354	0.2713	0.2916	0.0281	0.0411	0	0.0205	UN
10987	10987	16671	16671	4023297	0.0989	0.0363	0.2221	0.3034	0.0348	0.1563	0	0.0781	4th
10987	10987	16702	16702	4023297	0.1105	0.035	0.2763	0.3241	0.0342	0.3741	0.0014	0.1884	2nd
1X1672	1X1672	16827	16827	4023297	0.0978	0.0228	0.2926	0.1941	0.0772	0.1893	0.0253	0.12	3rd
1X1672	1X1672	16829	16829	4023297	0.1031	0.0142	0.3378	0.1201	0.1192	0.4029	0.0486	0.25	2nd
1X2055	1X2055	16986	16986	4023297	0.1082	0.0206	0.317	0.1867	0.1165	0.2449	0.0201	0.1426	3rd
1X2891	1X2891	16986	16986	4023297	0.0978	0.0281	0.2757	0.2428	0.0512	0.1193	0	0.0596	4th
1X3162	1X3162	16861	16861	4023297	0.1009	0.0351	0.2666	0.3017	0.0601	0.1238	0	0.0619	4th
1X3576	1X3576	16775	16775	4023297	0.1133	0.019	0.3208	0.1779	0.1372	0.2998	0.0583	0.2082	2nd
1X3576	1X3576	17112	17112	4023297	0.1128	0.0161	0.3402	0.1478	0.138	0.3493	0.0951	0.2697	2nd
10173	10173	16808	16808	4023297	0.0962	0.0126	0.283	0.1083	0.0447	0.2064	0	0.1032	3rd
10488	10488	16780	16780	4023297	0.1981	0.0468	0.3125	0.3483	0.0222	0.0145	0	0.0072	UN
10488	10488	16853	16853	4023297	0.2063	0.0557	0.3126	0.3331	0.0306	0.0067	0	0.0034	UN
10488	10488	16862	16862	4023297	0.2048	0.0294	0.3231	0.137	0.0892	0.4712	0	0.2356	2nd
1X1672	1X1672	17233	17233	4023297	0.0884	0.025	0.2742	0.2017	0.0159	0.2091	0.0261	0.1307	3rd
1X1672	1X1672	17871	17871	4023297	0.1035	0.0157	0.3321	0.1331	0.117	0.3644	0.0548	0.237	2nd
1X1672	1X1672	17891	17891	4023297	0.1028	0.0251	0.2764	0.2201	0.1091	0.0483	0	0.0242	UN
1X1765	1X1765	17199	17199	4023297	0.1311	0.0158	0.3571	0.1605	0.191	0.5334	0.1329	0.3996	FS
1X1765	1X1765	17340	17340	4023297	0.0994	0.0201	0.2936	0.1767	0.0602	0.2186	0.0087	0.118	3rd
1X3576	1X3576	17325	17325	4023297	0.1008	0.0186	0.3	0.1679	0.0881	0.211	0.0022	0.1077	3rd
10173	10173	17199	17199	4023297	0.1479	0.0001	0.4009	0.0009	0.2713	0.8738	0.1232	0.5602	PO
10173	10173	17340	17340	4023297	0.109	0.0088	0.3138	0.0813	0.1236	0.2614	0.0015	0.1322	3rd
10173	10173	17832	17832	4023297	0.1026	0.0123	0.297	0.1111	0.0788	0.1785	0	0.0893	3rd
10418	10418	17295	17295	4023297	0.179	0.058	0.3054	0.3796	0.0817	0	0	0	UN
10418	10418	17833	17833	4023297	0.1667	0.0574	0.2936	0.2604	0.0512	0	0	0	UN
10488	10488	17254	17254	4023297	0.198	0.031	0.3112	0.1374	0.072	0.4459	0	0.2229	2nd
10488	10488	17295	17295	4023297	0.2369	0.0351	0.3639	0.2923	0.1369	0.0424	0	0.0212	UN
10488	10488	17833	17833	4023297	0.2392	0.0179	0.3872	0.0964	0.1796	0.3936	0.0075	0.2044	2nd
1X1126	1X1126	17999	17999	4023297	0.0993	0.0238	0.29	0.2001	0.0578	0.2441	0.0195	0.1415	3rd
1X1126	1X1126	18141	18141	4023297	0.1044	0.022	0.3196	0.1844	0.0702	0.3209	0.0155	0.176	3rd
1X1672	1X1672	17998	17998	4023297	0.1059	0.0206	0.2856	0.1908	0.1355	0.0837	0	0.0419	UN
1X1765	1X1765	17903	17903	4023297	0.1274	0.0095	0.3511	0.0948	0.2082	0.7098	0.1034	0.4583	FS
1X1765	1X1765	18385	18385	4023297	0.1257	0.011	0.3543	0.1074	0.1958	0.6653	0.131	0.4637	FS
1X1765	1X1765	18523	18523	4023297	0.098	0.0288	0.2843	0.2541	0.0151	0.1434	0.0054	0.0771	4th
1X1765	1X1765	18599	18599	4023297	0.1133	0.0193	0.3268	0.1822	0.1193	0.2504	0.0165	0.1417	3rd
1X2055	1X2055	18144	18144	4023297	0.0983	0.0385	0.2587	0.3371	0.0357	0.0624	0	0.0312	UN
1X3162	1X3162	17998	17998	4023297	0.1061	0.0202	0.2867	0.1869	0.1372	0.1158	0	0.0579	4th
10173	10173	17903	17903	4023297	0.1406	0.0001	0.3817	0.0014	0.2563	0.9077	0.0899	0.5437	PO
10173	10173	18385	18385	4023297	0.1347	0.0001	0.3695	0.0013	0.2434	0.9141	0.0842	0.5412	PO
10173	10173	18523	18523	4023297	0.1083	0.0113	0.3066	0.1065	0.116	0.1465	0	0.0733	4th
10173	10173	18599	18599	4023297	0.1216	0.0078	0.3405	0.0776	0.1729	0.2546	0.0023	0.1296	3rd
10173	10173	18715	18715	4023297	0.1081	0.0111	0.3005	0.1055	0.1169	0.0626	0	0.0313	UN
10418	10418	18528	18528	4023297	0.1672	0.0638	0.2913	0.3999	0.0378	0	0	0	UN
10488	10488	18528	18528	4023297	0.2245	0.0378	0.3511	0.298	0.1046	0.013	0	0.0065	UN
1X1126	1X1126	18938	18938	4023297	0.1006	0.0369	0.2485	0.304	0.0487	0.1214	0.01	0.0707	4th
1X1126	1X1126	19173	19173	4023297	0.102	0.0272	0.2998	0.2306	0.0471	0.2422	0.007	0.1281	3rd
1X1126	1X1126	19215	19215	4023297	0.1012	0.0211	0.2986	0.1789	0.0752	0.2165	0.0287	0.137	3rd
1X1126	1X1126	19371	19371	4023297	0.1005	0.0299	0.2862	0.2531	0.0398	0.1514	0.0105	0.0862	4th
1X1765	1X1765	19181	19181	4023297	0.1395	0.0111	0.3798	0.1194	0.2283	0.6165	0.1613	0.4696	FS
1X1765	1X1765	19348	19348	4023297	0.1381	0.0091	0.3838	0.0957	0.2331	0.6685	0.1595	0.4937	FS
1X1765	1X1765	19365	19365	4023297	0.0969	0.0252	0.2932	0.2171	0.012	0.2285	0.0146	0.1288	3rd
1X3576	1X3576	19674	19674	4023297	0.1132	0.0239	0.3361	0.211	0.1033	0.289	0.1135	0.258	2nd
10173	10173	18840	18840	4023297	0.1021	0.013	0.2858	0.1206	0.0837	0.0426	0	0.0213	UN
10173	10173	19181	19181	4023297	0.1502	0.0001	0.4003	0.001	0.2752	0.8839	0.1128	0.5547	PO
10173	10173	19207	19207	4023297	0.1151	0.046	0.2624	0.443	0.0382	0.0032	0	0.0016	UN
10173	10173	19348	19348	4023297	0.1478	0.0001	0.4009	0.0012	0.2711	0.8651	0.1327	0.5653	PO
10173	10173	19365	19365	4023297	0.1056	0.0113	0.3103	0.1027	0.0913	0.2479	0	0.1239	3rd
10173	10173	19679	19679	4023297	0.1268	0.0022	0.4001	0.02	0.2057	0.6551	0.0488	0.3763	2nd
10349	10349	19237	19237	4023297	0.1682	0.0564	0.3062	0.2951	0.066	0.139	0.0428	0.1123	3rd
10488	10488	18866	18866	4023297	0.1952	0.0445	0.2945	0.3756	0.0411	0	0	0	UN
10488	10488	18929	18929	4023297	0.1865	0.0435	0.2817	0.3824	0.0259	0.0172	0	0.0086	UN
10489	10489	18818	18818	4023297	0.1095	0.0347	0.2369	0.3097	0.0617	0.1004	0	0.0502	4th
10489	10489	19207	19207	4023297	0.1162	0.0454	0.2622	0.4334	0.0439	0.004	0	0.002	UN
10987	10987	18818	18818	4023297	0.1086	0.0347	0.2349	0.3074	0.0599	0.1066	0	0.0533	4th
10987	10987	19207	19207	4023297	0.1164	0.0461	0.2633	0.4365	0.0423	0.0012	0	0.0006	UN
10987	10987	19378	19378	4023297	0.1113	0.0432	0.2524	0.3593	0.0391	0.1017	0	0.0509	4th
1X1126	1X1126	1X2231	1X2231	4023297	0.1368	0	0.368	0.0004	0.2653	0.9476	0.0505	0.5243	PO
1X1126	1X1126	1X3822	1X3822	4023297	0.1121	0.0446	0.2702	0.4124	0.0196	0.192	0	0.096	3rd
1X1126	1X1126	1X4080	1X4080	4023297	0.1083	0.0474	0.2666	0.4222	0.0135	0.1271	0	0.0635	4th
1X1126	1X1126	1X4777	1X4777	4023297	0.1333	0.0001	0.3475	0.0006	0.2499	0.9954	0.0035	0.5012	PO
1X1672	1X1672	1X3697	1X3697	4023297	0.1283	0	0.3365	0.0005	0.2342	0.9985	0	0.4992	PO
1X1672	1X1672	1X3822	1X3822	4023297	0.1279	0.0001	0.3307	0.0009	0.228	0.9984	0	0.4992	PO
1X1672	1X1672	1X4696	1X4696	4023297	0.1077	0.0089	0.3539	0.0775	0.1659	0.3726	0.0449	0.2312	2nd
1X1672	1X1672	1X4739	1X4739	4023297	0.0877	0.0076	0.2991	0.0607	0.0864	0.4506	0.0628	0.2882	2nd
1X1672	1X1672	1X4859	1X4859	4023297	0.0776	0.0065	0.2804	0.0485	0.016	0.5858	0.0633	0.3562	2nd
1X1765	1X1765	1X4777	1X4777	4023297	0.1093	0.0456	0.2646	0.4378	0.0254	0.0521	0	0.0261	UN
1X1765	1X1765	25354	25354	4023297	0.1347	0.0166	0.3754	0.1694	0.1939	0.3612	0.1826	0.3632	FS
1X1765	1X1765	26198	26198	4023297	0.1002	0.0201	0.2896	0.1819	0.071	0.2633	0.0047	0.1364	3rd
1X1958	1X1958	1X3796	1X3796	4023297	0.2042	0.0528	0.3136	0.3086	0.0688	0.0096	0	0.0048	UN
1X1958	1X1958	1X3837	1X3837	4023297	0.2049	0.0545	0.3118	0.3212	0.0608	0.0076	0	0.0038	UN
1X1958	1X1958	1X4209	1X4209	4023297	0.2034	0.0547	0.309	0.3243	0.0585	0.0124	0	0.0062	UN

FID1	ID1	FID2	ID2	N_SNP	HetHet	IBS0	HetConc	HomIBS0	Kinship	IBD1Seg	IBD2Seg	PropIBD	InfType
1X2055	1X2055	1X4179	1X4179	4023297	0.131	0.0001	0.3523	0.0005	0.2529	0.9962	0.0025	0.5006	PO
1X2664	1X2664	1X3697	1X3697	4023297	0.1095	0.0435	0.2647	0.4083	0.0251	0.0431	0	0.0216	UN
1X3321	1X3321	1X3796	1X3796	4023297	0.1978	0.0676	0.2944	0.2899	0.0298	0.0014	0	0.0007	UN
1X3321	1X3321	1X4209	1X4209	4023297	0.2006	0.0693	0.2971	0.2997	0.0254	0.0018	0	0.0009	UN
10046	10046	1X2231	1X2231	4023297	0.0979	0.0167	0.2793	0.1476	0.0802	0.0272	0	0.0136	UN
10173	10173	1X2124	1X2124	4023297	0.1523	0.0001	0.3725	0.0016	0.2661	0.9982	0	0.4991	PO
10173	10173	1X2816	1X2816	4023297	0.1128	0.0441	0.2644	0.4654	0.037	0.0796	0	0.0398	UN
10173	10173	1X3697	1X3697	4023297	0.113	0.0451	0.2609	0.4754	0.0389	0	0	0	UN
10173	10173	1X3822	1X3822	4023297	0.1152	0.0448	0.2642	0.4643	0.0449	0.0134	0	0.0067	UN
10173	10173	1X4080	1X4080	4023297	0.1124	0.0448	0.2635	0.4441	0.0332	0.0451	0	0.0226	UN
10173	10173	1X4179	1X4179	4023297	0.1107	0.0453	0.2617	0.4571	0.0236	0.0053	0	0.0026	UN
10173	10173	1X4777	1X4777	4023297	0.1149	0.0446	0.2694	0.4524	0.0405	0.0378	0	0.0189	UN
10173	10173	25354	25354	4023297	0.1384	0.0019	0.3704	0.0204	0.2442	0.7041	0.0729	0.425	2nd
10173	10173	26198	26198	4023297	0.1078	0.0067	0.3017	0.0637	0.137	0.2498	0	0.1249	3rd
10192	10192	1X3656	1X3656	4023297	0.1054	0.0432	0.2622	0.4086	0.0314	0.0213	0	0.0107	UN
10192	10192	1X4179	1X4179	4023297	0.1061	0.0427	0.2633	0.403	0.0327	0.0194	0	0.0097	UN
10349	10349	1X3796	1X3796	4023297	0.1937	0.061	0.2988	0.3595	0.0257	0.02	0	0.01	UN
10349	10349	1X3837	1X3837	4023297	0.2241	0.0403	0.3588	0.2453	0.1187	0.3844	0	0.1922	2nd
10349	10349	1X4209	1X4209	4023297	0.1979	0.0591	0.3045	0.3551	0.0323	0.0328	0	0.0164	UN
10418	10418	1X3796	1X3796	4023297	0.2084	0.0491	0.3214	0.3377	0.0848	0	0	0	UN
10418	10418	1X3837	1X3837	4023297	0.2087	0.0496	0.3186	0.3447	0.0794	0	0	0	UN
10418	10418	1X4209	1X4209	4023297	0.2089	0.0495	0.3194	0.3464	0.0804	0	0	0	UN
10488	10488	1X3796	1X3796	4023297	0.2753	0.0304	0.3909	0.2694	0.2094	0.0032	0	0.0016	UN
10488	10488	1X3837	1X3837	4023297	0.2766	0.0313	0.3897	0.2826	0.2095	0.0058	0	0.0029	UN
10488	10488	1X4209	1X4209	4023297	0.2772	0.0304	0.3912	0.2776	0.212	0.0097	0	0.0048	UN
10488	10488	25355	25355	4023297	0.1892	0.035	0.3012	0.1555	0.0353	0.2618	0	0.1309	3rd
10488	10488	25593	25593	4023297	0.1949	0.0125	0.3244	0.1027	0.1063	0.0381	0	0.019	UN
10489	10489	1X2124	1X2124	4023297	0.1212	0.0432	0.272	0.4321	0.0569	0.0344	0	0.0172	UN
10489	10489	1X2816	1X2816	4023297	0.1405	0.0001	0.3472	0.0008	0.2502	0.9992	0	0.4996	PO
10489	10489	1X3697	1X3697	4023297	0.1158	0.044	0.2655	0.4583	0.0428	0	0	0	UN
10489	10489	1X3822	1X3822	4023297	0.1167	0.0439	0.2649	0.4501	0.0485	0.0161	0	0.008	UN
10489	10489	1X4080	1X4080	4023297	0.1128	0.0451	0.2613	0.4425	0.0271	0.0149	0	0.0075	UN
10489	10489	1X4777	1X4777	4023297	0.1141	0.0456	0.2635	0.4553	0.0296	0.0222	0	0.0111	UN
10489	10489	25347	25347	4023297	0.114	0.0495	0.2526	0.4503	0.023	0.0028	0	0.0014	UN
10842	10842	1X2231	1X2231	4023297	0.0994	0.0268	0.274	0.238	0.0459	0.0191	0	0.0095	UN
10842	10842	1X4777	1X4777	4023297	0.1268	0.0204	0.3688	0.2025	0.1343	0.2706	0.011	0.1463	3rd
10987	10987	1X2124	1X2124	4023297	0.1215	0.0434	0.2733	0.4315	0.0564	0.0527	0	0.0263	UN
10987	10987	1X2816	1X2816	4023297	0.1395	0.0001	0.3445	0.0007	0.249	0.9989	0	0.4994	PO
10987	10987	1X3697	1X3697	4023297	0.1157	0.0434	0.2655	0.4502	0.0455	0.0031	0	0.0015	UN
10987	10987	1X3822	1X3822	4023297	0.117	0.0434	0.2664	0.4423	0.0516	0.0121	0	0.0061	UN
10987	10987	1X4080	1X4080	4023297	0.1132	0.0456	0.2627	0.4445	0.0267	0.0105	0	0.0053	UN
10987	10987	1X4777	1X4777	4023297	0.1141	0.0459	0.264	0.4562	0.0294	0.0123	0	0.0062	UN
10987	10987	25347	25347	4023297	0.1143	0.0492	0.2539	0.4447	0.0243	0.0058	0	0.0029	UN
1X1126	1X1126	26498	26498	4023297	0.169	0.0125	0.3206	0.1526	0.0928	0.3229	0	0.1614	3rd
1X1126	1X1126	26800	26800	4023297	0.107	0.0257	0.3049	0.2253	0.0822	0.1978	0.046	0.1449	3rd
1X1126	1X1126	26980	26980	4023297	0.0959	0.0307	0.2605	0.2382	0.038	0.2476	0.021	0.1448	3rd
1X1126	1X1126	27351	27351	4023297	0.1119	0.027	0.263	0.2298	0.079	0.5562	0.0183	0.2964	2nd
1X1672	1X1672	27306	27306	4023297	0.0978	0.0162	0.3074	0.1322	0.0991	0.3668	0.0678	0.2512	2nd
1X1765	1X1765	26988	26988	4023297	0.1225	0.0179	0.3235	0.1797	0.1655	0.5592	0.0574	0.3371	2nd
1X1765	1X1765	27503	27503	4023297	0.1342	0.0137	0.3667	0.1418	0.2063	0.5548	0.1404	0.4178	FS
1X1958	1X1958	26498	26498	4023297	0.1814	0.0652	0.2809	0.4488	0.0249	0	0	0	UN
1X2891	1X2891	27503	27503	4023297	0.1048	0.0377	0.2716	0.3343	0.0574	0.3471	0	0.1736	3rd
1X3162	1X3162	26318	26318	4023297	0.0987	0.0363	0.2621	0.3026	0.0547	0.0723	0	0.0362	UN
10173	10173	26498	26498	4023297	0.1531	0.0275	0.27	0.3743	0.0233	0.0021	0	0.001	UN
10173	10173	26988	26988	4023297	0.1505	0.0001	0.4076	0.0009	0.2765	0.8658	0.1305	0.5634	PO
10173	10173	27199	27199	4023297	0.0921	0.0064	0.2899	0.0534	0.0358	0.532	0	0.266	2nd
10173	10173	27503	27503	4023297	0.1422	0.0005	0.3778	0.0054	0.2579	0.8976	0.0695	0.5184	PO
10418	10418	26498	26498	4023297	0.1792	0.0491	0.2759	0.4169	0.0649	0	0	0	UN
10488	10488	26324	26324	4023297	0.1923	0.0138	0.322	0.1107	0.0944	0.0286	0	0.0143	UN
10488	10488	26498	26498	4023297	0.2338	0.0294	0.3257	0.3543	0.1624	0.0015	0	0.0007	UN
10488	10488	27033	27033	4023297	0.1984	0.0432	0.3208	0.2929	0.0236	0.0065	0	0.0032	UN
10489	10489	26498	26498	4023297	0.1556	0.028	0.2727	0.3725	0.0304	0.0042	0	0.0021	UN
10987	10987	26498	26498	4023297	0.1554	0.0285	0.2727	0.3759	0.0279	0.0042	0	0.0021	UN
1X1126	1X1126	28212	28212	4023297	0.0944	0.0246	0.2896	0.1967	0.0145	0.2949	0.0231	0.1705	3rd
1X1126	1X1126	28279	28279	4023297	0.1188	0.0298	0.2697	0.2649	0.0599	0.475	0.0227	0.2602	2nd
1X1672	1X1672	27989	27989	4023297	0.1116	0.0121	0.3326	0.1075	0.1743	0.3963	0.1306	0.3287	2nd
1X1672	1X1672	27999	27999	4023297	0.0984	0.0077	0.3255	0.0634	0.1389	0.4589	0.0516	0.281	2nd
1X1672	1X1672	28183	28183	4023297	0.1136	0.0074	0.3406	0.0654	0.2015	0.644	0.1308	0.4528	FS
1X1765	1X1765	28246	28246	4023297	0.1338	0.0205	0.3528	0.2218	0.1804	0.4402	0.1288	0.3489	2nd
10173	10173	28246	28246	4023297	0.1503	0.0001	0.3939	0.0016	0.2745	0.9359	0.0613	0.5292	PO
10488	10488	28274	28274	4023297	0.1834	0.0171	0.3092	0.1302	0.058	0.0118	0	0.0059	UN
1X1126	1X1126	28416	28416	4023297	0.1079	0.0197	0.3151	0.1726	0.1081	0.258	0.0567	0.1857	2nd
1X1672	1X1672	28417	28417	4023297	0.0911	0.0153	0.2832	0.1248	0.0823	0.2907	0.0341	0.1794	2nd
1X1672	1X1672	28436	28436	4023297	0.0907	0.0095	0.3062	0.0764	0.0903	0.4784	0.0478	0.287	2nd
1X1672	1X1672	28635	28635	4023297	0.1345	0.0054	0.3718	0.0503	0.2386	0.627	0.2939	0.6074	FS
10164	10164	28635	28635	4023297	0.0989	0.0398	0.2425	0.2717	0.0293	0.1673	0.0535	0.1372	3rd
1X1126	1X1126	30614	30614	4023297	0.0981	0.0383	0.2373	0.3216	0.0325	0.0343	0.0023	0.0194	UN
1X1126	1X1126	30917	30917	4023297	0.1354	0.0276	0.2846	0.307	0.0501	0.0087	0	0.0044	UN
1X1672	1X1672	30611	30611	4023297	0.0948	0.0342	0.25	0.2925	0.0534	0.0305	0	0.0153	UN
1X1672	1X1672	30917	30917	4023297	0.1274	0.0276	0.2702	0.2985	0.0231	0	0	0	UN
1X1958	1X1958	30920	30920	4023297	0.1804	0.0558	0.2935	0.3665	0.0694	0	0	0	UN
10173	10173	30917	30917	4023297	0.1349	0.0313	0.2696	0.4014	0.0534	0	0	0	UN
10418	10418	30920	30920	4023297	0.1808	0.0497	0.2937	0.3948	0.0865	0	0	0	UN
10488	10488	30886	30886	4023297	0.1966	0.0139	0.329	0.1117	0.1041	0.0286	0	0.0143	UN
10488	10488	30917	30917	4023297	0.1847	0.038	0.2709	0.4298	0.0499	0	0	0	UN
10488	10488	30920	30920	4023297	0.2344	0.0313	0.3423	0.3401	0.1518	0	0	0	UN
10489	10489	30917	30917	4023297	0.1404	0.0304	0.2804	0.3835	0.0705	0.0054	0	0.0027	UN
10987	10987	30917	30917	4023297	0.1394	0.0319	0.2781	0.3979	0.0628	0.0019	0	0.0009	UN
1X1126	1X1126	31021	31021	4023297	0.1169	0.0268	0.26	0.2488	0.061	0.4471	0.0032	0.2268	2nd
1X1126	1X1126	31134	31134	4023297	0.1068	0.0329	0.276	0.293	0.0767	0.0867	0.002	0.0454	4th
1X1672	1X1672	31016	31016	4023297	0.1053	0.0253	0.2839	0.2266	0.1147	0.11	0	0.055	4th
1X1672	1X1672	31145	31145	4023297	0.1154	0.0239	0.2679	0.226	0.0685	0.5846	0	0.2923	2nd
1X1672	1X1672	31234	31234	4023297	0.1017	0.0362	0.2519	0.314	0.0311	0.0532	0	0.0266	UN
1X1765	1X1765	30961	30961	4023297	0.1072	0.0336	0.2756	0.3257	0.0665	0.1468	0	0.0734	4th
1X1765	1X1765	31145	31145	4023297	0.1136	0.0391	0.2523	0.3815	0.0185	0.2416	0	0.1208	3rd
1X2891	1X2891	30961	30961	4023297	0.1	0.0388	0.2586	0.3563	0.0397	0.0518	0	0.0259	UN
10173	10173	30961	30961	4023297	0.1136	0.0271	0.2833	0.2794	0.0876	0.179	0	0.0895	3rd
10173	10173	31145	31145	4023297	0.1152	0.0431	0.2463	0.4382	0.0225	0.1088	0	0.0544	4th

FID1	ID1	FID2	ID2	N_SNP	HetHet	IBS0	HetConc	HomIBS0	Kinship	IBD1Seg	IBD2Seg	PropIBD	InfType
10349	10349	30933	30933	4023297	0.1574	0.0673	0.2765	0.3954	0.0252	0.0641	0	0.0321	UN
10418	10418	31065	31065	4023297	0.1671	0.062	0.2891	0.4115	0.0448	0	0	0	UN
10488	10488	30933	30933	4023297	0.2003	0.044	0.3015	0.3765	0.0537	0	0	0	UN
10488	10488	30974	30974	4023297	0.1853	0.0201	0.3159	0.148	0.0471	0.0142	0	0.0071	UN
10488	10488	31011	31011	4023297	0.1892	0.0454	0.2833	0.4087	0.0297	0.004	0	0.002	UN
10488	10488	31065	31065	4023297	0.2186	0.0382	0.3367	0.3232	0.0968	0.0204	0	0.0102	UN
10488	10488	31120	31120	4023297	0.182	0.0168	0.3085	0.1143	0.0531	0.0964	0	0.0482	4th
10488	10488	31177	31177	4023297	0.1853	0.0185	0.3105	0.1069	0.0603	0.1166	0	0.0583	4th
10488	10488	31178	31178	4023297	0.189	0.0222	0.3222	0.1586	0.0489	0.0018	0	0.0009	UN
10489	10489	31011	31011	4023297	0.1248	0.0358	0.2463	0.3564	0.032	0.1428	0	0.0714	4th
10987	10987	31011	31011	4023297	0.1252	0.0341	0.2477	0.3386	0.0383	0.1582	0	0.0791	4th
1X1126	1X1126	31284	31284	4023297	0.1098	0.0339	0.2527	0.3044	0.0404	0.123	0	0.0615	4th
1X1126	1X1126	31297	31297	4023297	0.1155	0.029	0.2786	0.2726	0.0857	0.3902	0.0025	0.1977	2nd
1X1126	1X1126	31299	31299	4023297	0.1085	0.0322	0.2663	0.2947	0.073	0.1381	0.0012	0.0703	4th
1X1126	1X1126	31331	31331	4023297	0.1134	0.0306	0.2441	0.2736	0.0274	0.1769	0.0017	0.0901	3rd
1X3576	1X3576	31297	31297	4023297	0.1156	0.0301	0.2798	0.2935	0.0804	0.4252	0.0033	0.2159	2nd
1X3576	1X3576	31299	31299	4023297	0.1063	0.037	0.2605	0.3472	0.0484	0.1384	0.0017	0.0709	4th
1X3576	1X3576	31327	31327	4023297	0.1	0.0319	0.2703	0.295	0.0506	0.0759	0	0.0379	UN
1X3576	1X3576	31364	31364	4023297	0.1107	0.0305	0.245	0.2799	0.0348	0.1282	0	0.0641	4th
10173	10173	31297	31297	4023297	0.1124	0.048	0.2542	0.4793	0.0254	0.0102	0	0.0051	UN
10349	10349	31275	31275	4023297	0.1218	0.0261	0.2419	0.1531	0.0275	0.0278	0	0.0139	UN
10349	10349	31337	31337	4023297	0.1514	0.0536	0.2698	0.3255	0.0469	0.1378	0	0.0689	4th
10488	10488	31300	31300	4023297	0.1971	0.0145	0.3306	0.1012	0.102	0.0764	0	0.0382	UN
10488	10488	31475	31475	4023297	0.1852	0.0201	0.3136	0.1482	0.0497	0.0013	0	0.0006	UN
10489	10489	31284	31284	4023297	0.116	0.0417	0.253	0.4038	0.046	0.0847	0	0.0424	UN
10489	10489	31297	31297	4023297	0.111	0.0475	0.247	0.4687	0.0278	0.0076	0	0.0038	UN
10489	10489	31462	31462	4023297	0.1087	0.0441	0.2406	0.41	0.036	0.0275	0	0.0137	UN
10987	10987	31284	31284	4023297	0.1149	0.0434	0.2502	0.416	0.0373	0.0656	0	0.0328	UN
10987	10987	31297	31297	4023297	0.1112	0.0482	0.2479	0.4719	0.0261	0.0016	0	0.0008	UN
10987	10987	31462	31462	4023297	0.11	0.0422	0.2445	0.3915	0.0457	0.0212	0	0.0106	UN
1X1126	1X1126	31498	31498	4023297	0.1162	0.0279	0.2523	0.2583	0.0449	0.3254	0.0021	0.1647	3rd
1X3576	1X3576	31500	31500	4023297	0.1015	0.038	0.2611	0.3588	0.0448	0.0957	0	0.0479	4th
1X3576	1X3576	31559	31559	4023297	0.0983	0.0314	0.2652	0.2844	0.0477	0.0616	0	0.0308	UN
10349	10349	31497	31497	4023297	0.1839	0.01	0.3438	0.0631	0.2212	0.2988	0	0.1494	3rd
10349	10349	31656	31656	4023297	0.1769	0.0146	0.3354	0.0902	0.1958	0.101	0.0018	0.0522	4th
10488	10488	31497	31497	4023297	0.1913	0.0455	0.2876	0.3921	0.032	0	0	0	UN
10489	10489	31500	31500	4023297	0.1106	0.0375	0.2689	0.3749	0.0329	0.0749	0	0.0375	UN
10987	10987	31500	31500	4023297	0.1098	0.0377	0.2668	0.3736	0.0313	0.0533	0	0.0267	UN
1X1126	1X1126	31786	31786	4023297	0.1081	0.0366	0.2548	0.3268	0.038	0.0938	0	0.0469	4th
1X1126	1X1126	31849	31849	4023297	0.1024	0.0197	0.3125	0.163	0.0771	0.3034	0.0556	0.2073	2nd
1X1126	1X1126	31952	31952	4023297	0.1012	0.0374	0.2555	0.3282	0.0501	0.0425	0	0.0212	UN
1X3576	1X3576	31764	31764	4023297	0.1052	0.0401	0.2544	0.3531	0.0293	0.0937	0	0.0468	4th
1X3576	1X3576	31882	31882	4023297	0.0956	0.0339	0.2559	0.2983	0.0309	0.0717	0	0.0358	UN
1X3576	1X3576	31952	31952	4023297	0.1043	0.03	0.2662	0.2779	0.0877	0.0925	0	0.0462	4th
10173	10173	31902	31902	4023297	0.1038	0.0269	0.2759	0.2543	0.0371	0.0591	0	0.0296	UN
10349	10349	31937	31937	4023297	0.1509	0.0242	0.2969	0.14	0.1097	0.0408	0	0.0204	UN
10418	10418	31903	31903	4023297	0.1672	0.065	0.286	0.3735	0.0407	0	0	0	UN
10488	10488	31666	31666	4023297	0.1906	0.0137	0.3169	0.0852	0.0931	0.166	0	0.083	4th
10488	10488	31789	31789	4023297	0.1852	0.0105	0.3024	0.0712	0.0977	0.1833	0	0.0917	3rd
10488	10488	31903	31903	4023297	0.2163	0.0397	0.3286	0.2768	0.0926	0.0459	0	0.0229	UN
10488	10488	31929	31929	4023297	0.2017	0.0165	0.3405	0.127	0.1037	0.0266	0	0.0133	UN
10489	10489	31764	31764	4023297	0.1107	0.045	0.2518	0.4145	0.0283	0.0322	0	0.0161	UN
10489	10489	31786	31786	4023297	0.1124	0.0439	0.2497	0.4226	0.0424	0.0408	0	0.0204	UN
10987	10987	31786	31786	4023297	0.1118	0.044	0.2483	0.4215	0.0404	0.0318	0	0.0159	UN
1X1126	1X1126	32025	32025	4023297	0.1159	0.0293	0.2867	0.2753	0.0953	0.4077	0.0047	0.2085	2nd
1X1765	1X1765	32043	32043	4023297	0.1228	0.0239	0.2915	0.2411	0.1154	0.5247	0	0.2624	2nd
1X3576	1X3576	32025	32025	4023297	0.1184	0.0221	0.296	0.2195	0.1284	0.5178	0.0015	0.2604	2nd
1X3576	1X3576	32040	32040	4023297	0.1146	0.0279	0.2711	0.268	0.0783	0.4565	0.0026	0.2308	2nd
10173	10173	32025	32025	4023297	0.1104	0.0478	0.2544	0.4763	0.0228	0.0085	0	0.0042	UN
10173	10173	32043	32043	4023297	0.1304	0.0201	0.3018	0.2151	0.1523	0.5736	0	0.2868	2nd
10349	10349	32187	32187	4023297	0.1494	0.052	0.2629	0.2951	0.0514	0.1029	0	0.0514	4th
10349	10349	32207	32207	4023297	0.159	0.022	0.3053	0.1162	0.1394	0.0693	0	0.0347	UN
10488	10488	32089	32089	4023297	0.2032	0.0473	0.3045	0.3269	0.0522	0.0071	0	0.0036	UN
10489	10489	32040	32040	4023297	0.1188	0.0389	0.264	0.3864	0.0661	0.1785	0	0.0892	3rd
10489	10489	32043	32043	4023297	0.116	0.0463	0.2564	0.4572	0.0352	0.0077	0	0.0038	UN
10489	10489	32224	32224	4023297	0.1231	0.0376	0.2509	0.3729	0.0385	0.1317	0	0.0658	4th
10987	10987	32040	32040	4023297	0.116	0.0438	0.2564	0.4275	0.0432	0.1305	0	0.0652	4th
10987	10987	32043	32043	4023297	0.1158	0.047	0.2562	0.4602	0.0318	0.0082	0	0.0041	UN
10987	10987	32224	32224	4023297	0.1242	0.0374	0.2542	0.3705	0.0406	0.1627	0	0.0814	4th
1X1126	1X1126	32772	32772	4023297	0.1014	0.0336	0.2725	0.2956	0.0458	0.0782	0.0015	0.0406	UN
1X1672	1X1672	33083	33083	4023297	0.0959	0.0389	0.2448	0.323	0.0261	0.0224	0	0.0112	UN
1X1765	1X1765	32849	32849	4023297	0.1241	0.0235	0.2812	0.2367	0.0984	0.5509	0	0.2754	2nd
1X3576	1X3576	32909	32909	4023297	0.0967	0.0364	0.2548	0.3174	0.0288	0.0438	0	0.0219	UN
10173	10173	32849	32849	4023297	0.1326	0.0189	0.2937	0.2016	0.1411	0.6042	0	0.3021	2nd
1X1126	1X1126	6382	6382	4023297	0.1013	0.0083	0.348	0.0665	0.1076	0.5499	0.0228	0.2977	2nd
1X1672	1X1672	34857	34857	4023297	0.1155	0.0275	0.2644	0.2566	0.0474	0.5101	0	0.255	2nd
1X1672	1X1672	7091	7091	4023297	0.1272	0.0001	0.3301	0.0008	0.2287	0.9984	0	0.4992	PO
1X1672	1X1672	7777	7777	4023297	0.111	0.0047	0.3925	0.0406	0.1936	0.5542	0.0853	0.3624	FS
1X1672	1X1672	7790	7790	4023297	0.0857	0.0084	0.2821	0.0664	0.0841	0.4121	0.0409	0.247	2nd
1X1672	1X1672	8134	8134	4023297	0.1002	0.0121	0.3252	0.1021	0.1234	0.3448	0.0515	0.224	2nd
1X1765	1X1765	33863	33863	4023297	0.1228	0.0257	0.2923	0.2564	0.1095	0.5129	0	0.2565	2nd
1X1765	1X1765	34857	34857	4023297	0.1177	0.0318	0.26	0.316	0.0487	0.3655	0	0.1828	2nd
1X1958	1X1958	6716	6716	4023297	0.1747	0.0634	0.2815	0.3802	0.0416	0.0101	0	0.005	UN
1X3576	1X3576	33889	33889	4023297	0.0998	0.0381	0.264	0.3477	0.0297	0.0781	0	0.0391	UN
1X3576	1X3576	33908	33908	4023297	0.1193	0.0246	0.2932	0.2401	0.1123	0.4556	0.0554	0.2832	2nd
1X3576	1X3576	7478	7478	4023297	0.1054	0.0102	0.3008	0.0967	0.1542	0.1022	0	0.0511	4th
1X4519	1X4519	6265	6265	4023297	0.1337	0.0001	0.3349	0.0009	0.2393	0.9975	0	0.4987	PO
1X4519	1X4519	6955	6955	4023297	0.1363	0.0001	0.3408	0.0009	0.2411	0.9929	0.0015	0.498	PO
1X4519	1X4519	7625	7625	4023297	0.1341	0.0001	0.3352	0.0008	0.2388	0.9972	0	0.4986	PO
1X4519	1X4519	8395	8395	4023297	0.1355	0.0001	0.3394	0.0007	0.2411	0.998	0	0.499	PO
10173	10173	33863	33863	4023297	0.133	0.019	0.3105	0.2035	0.162	0.613	0.0057	0.3121	2nd
10173	10173	34857	34857	4023297	0.1188	0.0394	0.2527	0.4047	0.0367	0.1809	0	0.0904	3rd
10173	10173	6265	6265	4023297	0.1153	0.0441	0.2635	0.4545	0.0463	0.0381	0	0.019	UN
10173	10173	6955	6955	4023297	0.1153	0.0445	0.2615	0.4587	0.0417	0.0288	0	0.0144	UN
10173	10173	7091	7091	4023297	0.1136	0.0449	0.2607	0.4663	0.0431	0.0028	0	0.0014	UN
10173	10173	7267	7267	4023297	0.1191	0.0419	0.272	0.4343	0.0576	0.0221	0	0.011	UN
10173	10173	7311	7311	4023297	0.113	0.0445	0.2652	0.4555	0.0355	0.0732	0	0.0366	UN
10173	10173	7625	7625	4023297	0.1155	0.0446	0.2634	0.4595	0.0437	0.0346	0	0.0173	UN

FID1	ID1	FID2	ID2	N_SNP	HetHet	IBS0	HetConc	HomIBS0	Kinship	IBD1Seg	IBD2Seg	PropIBD	InfType
10173	10173	8170	8170	4023297	0.1124	0.0445	0.2608	0.4662	0.0375	0.0411	0	0.0206	UN
10173	10173	8307	8307	4023297	0.1117	0.0453	0.2616	0.4632	0.0295	0.044	0	0.022	UN
10173	10173	8395	8395	4023297	0.1174	0.0442	0.2686	0.4549	0.0482	0.0404	0	0.0202	UN
10192	10192	7937	7937	4023297	0.1051	0.0453	0.2618	0.4208	0.023	0.0079	0	0.0039	UN
10349	10349	7158	7158	4023297	0.1742	0.0379	0.3185	0.1758	0.1286	0.6313	0.0769	0.3925	2nd
10349	10349	8344	8344	4023297	0.1857	0.0448	0.3145	0.2344	0.1034	0.6398	0.0017	0.3216	2nd
10418	10418	6716	6716	4023297	0.176	0.0613	0.2834	0.4337	0.0497	0	0	0	UN
10418	10418	8344	8344	4023297	0.1744	0.0688	0.2828	0.3859	0.0319	0	0	0	UN
10488	10488	34568	34568	4023297	0.2025	0.047	0.3021	0.3355	0.0531	0.0103	0	0.0051	UN
10488	10488	6716	6716	4023297	0.2304	0.0384	0.3341	0.3563	0.13	0.0048	0	0.0024	UN
10488	10488	8344	8344	4023297	0.2305	0.0437	0.3374	0.2978	0.1152	0.0155	0	0.0078	UN
10489	10489	33863	33863	4023297	0.1159	0.0466	0.2569	0.458	0.035	0.0211	0	0.0106	UN
10489	10489	6265	6265	4023297	0.1749	0.0092	0.456	0.1136	0.2794	0.5624	0.2288	0.5099	FS
10489	10489	6716	6716	4023297	0.1407	0.0238	0.2542	0.2496	0.047	0.5654	0	0.2827	2nd
10489	10489	6955	6955	4023297	0.1741	0.0118	0.4491	0.1451	0.2676	0.5131	0.2235	0.48	FS
10489	10489	7091	7091	4023297	0.1176	0.0432	0.2688	0.4465	0.0511	0.0116	0	0.0058	UN
10489	10489	7267	7267	4023297	0.1334	0.0242	0.3107	0.2625	0.1499	0.4275	0	0.2138	2nd
10489	10489	7311	7311	4023297	0.127	0.0224	0.3038	0.2403	0.1402	0.4982	0	0.2491	2nd
10489	10489	7625	7625	4023297	0.179	0.0116	0.4702	0.1437	0.278	0.4831	0.2609	0.5025	FS
10489	10489	8170	8170	4023297	0.1271	0.0259	0.3014	0.2838	0.1289	0.4259	0.0014	0.2143	2nd
10489	10489	8307	8307	4023297	0.1255	0.0246	0.2997	0.262	0.129	0.4804	0	0.2402	2nd
10489	10489	8395	8395	4023297	0.1858	0.0067	0.4962	0.0859	0.3075	0.565	0.2743	0.5568	FS
10987	10987	33863	33863	4023297	0.1166	0.0463	0.2591	0.452	0.0368	0.0295	0	0.0147	UN
10987	10987	6265	6265	4023297	0.1694	0.0108	0.4362	0.1311	0.2643	0.5491	0.1955	0.4701	FS
10987	10987	6716	6716	4023297	0.1383	0.0273	0.2488	0.2822	0.0298	0.4842	0	0.2421	2nd
10987	10987	6955	6955	4023297	0.1804	0.012	0.4739	0.1502	0.2782	0.4515	0.2708	0.4966	FS
10987	10987	7091	7091	4023297	0.1171	0.0433	0.2678	0.4446	0.0505	0.0088	0	0.0044	UN
10987	10987	7267	7267	4023297	0.1313	0.0268	0.3048	0.2868	0.1368	0.4046	0	0.2023	2nd
10987	10987	7311	7311	4023297	0.1252	0.0278	0.2989	0.2925	0.1171	0.4097	0	0.2048	2nd
10987	10987	7625	7625	4023297	0.1726	0.0108	0.4466	0.132	0.2698	0.55	0.2091	0.4841	FS
10987	10987	8170	8170	4023297	0.1295	0.0245	0.3093	0.2684	0.1394	0.4595	0.0049	0.2346	2nd
10987	10987	8307	8307	4023297	0.1227	0.03	0.2916	0.3137	0.1036	0.3648	0	0.1824	2nd
10987	10987	8395	8395	4023297	0.1839	0.0079	0.4893	0.1005	0.3002	0.5337	0.2821	0.549	FS
1X1126	1X1126	8995	8995	4023297	0.1286	0.0016	0.3173	0.0159	0.2172	0.8929	0	0.4465	2nd
1X1126	1X1126	9562	9562	4023297	0.1352	0.0001	0.348	0.0006	0.2469	0.9992	0	0.4996	PO
1X1672	1X1672	8635	8635	4023297	0.106	0.0086	0.3406	0.0741	0.1649	0.37	0.0311	0.2161	2nd
1X1672	1X1672	8653	8653	4023297	0.147	0	0.4828	0.0004	0.3152	0.5989	0.394	0.6935	PO
1X1672	1X1672	9514	9514	4023297	0.1064	0.0067	0.3577	0.0575	0.1711	0.4611	0.0651	0.2957	2nd
1X1765	1X1765	9841	9841	4023297	0.1385	0.0001	0.3644	0.0006	0.264	0.9956	0.0032	0.501	PO
1X1765	1X1765	9878	9878	4023297	0.096	0.0138	0.2817	0.1237	0.0844	0.0663	0	0.0331	UN
1X1958	1X1958	9860	9860	4023297	0.163	0.0716	0.2701	0.4191	0.0239	0.0253	0	0.0126	UN
1X3162	1X3162	8596	8596	4023297	0.1009	0.0174	0.2431	0.1433	0.0959	0.0206	0	0.0103	UN
1X3321	1X3321	9656	9656	4023297	0.1831	0.0656	0.2892	0.2236	0.0501	0.0026	0	0.0013	UN
1X3576	1X3576	8780	8780	4023297	0.1328	0.0001	0.3402	0.0006	0.241	0.9966	0	0.4983	PO
1X4519	1X4519	9128	9128	4023297	0.135	0.0001	0.3384	0.0009	0.2408	0.9972	0	0.4986	PO
1Y1979	1Y1979	8780	8780	4023297	0.1184	0.0255	0.2956	0.2455	0.1075	0.4739	0	0.2369	2nd
10173	10173	8780	8780	4023297	0.1133	0.0452	0.2601	0.4819	0.0416	0.0065	0	0.0032	UN
10173	10173	8995	8995	4023297	0.119	0.0434	0.2709	0.4278	0.0507	0.0153	0	0.0077	UN
10173	10173	9045	9045	4023297	0.1127	0.0442	0.2655	0.4493	0.0349	0.0642	0.0017	0.0338	UN
10173	10173	9128	9128	4023297	0.1165	0.0439	0.2663	0.4545	0.0482	0.0425	0	0.0213	UN
10173	10173	9562	9562	4023297	0.1165	0.0442	0.27	0.4583	0.0502	0.0148	0	0.0074	UN
10173	10173	9841	9841	4023297	0.1256	0.0222	0.3051	0.2432	0.1431	0.4955	0	0.2477	2nd
10418	10418	8465	8465	4023297	0.1586	0.0639	0.2617	0.4563	0.0392	0	0	0	UN
10418	10418	9656	9656	4023297	0.1993	0.0554	0.33	0.2449	0.0913	0.0051	0	0.0025	UN
10418	10418	9860	9860	4023297	0.1647	0.062	0.2731	0.4295	0.0521	0	0	0	UN
10488	10488	8465	8465	4023297	0.2077	0.0419	0.3055	0.3929	0.0808	0.0019	0	0.001	UN
10488	10488	9656	9656	4023297	0.2653	0.0235	0.4016	0.1183	0.2087	0.5374	0	0.2687	2nd
10488	10488	9860	9860	4023297	0.2169	0.0387	0.3219	0.3491	0.1026	0.0065	0	0.0032	UN
10489	10489	8465	8465	4023297	0.1406	0.0262	0.2696	0.2797	0.0671	0.4678	0	0.2339	2nd
10489	10489	8780	8780	4023297	0.1172	0.0431	0.2678	0.4588	0.0508	0.0101	0	0.005	UN
10489	10489	8995	8995	4023297	0.1176	0.0472	0.2634	0.4571	0.0385	0.0121	0	0.0061	UN
10489	10489	9045	9045	4023297	0.1311	0.0174	0.3182	0.1886	0.1664	0.6233	0	0.3117	2nd
10489	10489	9128	9128	4023297	0.1713	0.0122	0.441	0.1495	0.2618	0.4969	0.2164	0.4649	FS
10489	10489	9562	9562	4023297	0.1164	0.0442	0.2661	0.4523	0.0447	0.0083	0	0.0041	UN
10489	10489	9841	9841	4023297	0.111	0.0443	0.2569	0.4525	0.0253	0.0046	0	0.0023	UN
10842	10842	9562	9562	4023297	0.1058	0.027	0.2848	0.2504	0.0424	0.032	0	0.016	UN
10987	10987	8465	8465	4023297	0.1428	0.0242	0.2754	0.259	0.0778	0.5292	0.0017	0.2663	2nd
10987	10987	8780	8780	4023297	0.1158	0.044	0.264	0.4629	0.0457	0.0058	0	0.0029	UN
10987	10987	8995	8995	4023297	0.1173	0.0479	0.2628	0.4609	0.035	0.0022	0	0.0011	UN
10987	10987	9045	9045	4023297	0.1266	0.0235	0.3044	0.2489	0.1349	0.5043	0	0.2521	2nd
10987	10987	9128	9128	4023297	0.1745	0.0106	0.4541	0.1303	0.2741	0.5312	0.2279	0.4935	FS
10987	10987	9562	9562	4023297	0.1154	0.0444	0.2637	0.4526	0.0429	0.0068	0	0.0034	UN
10987	10987	9841	9841	4023297	0.1126	0.0448	0.2619	0.4559	0.0271	0.0076	0	0.0038	UN
11158	11158	11693	11693	4023297	0.1342	0.0434	0.3037	0.3249	0.0698	0	0	0	UN
11158	11158	11769	11769	4023297	0.1343	0.0509	0.2809	0.371	0.0385	0.0058	0	0.0029	UN
11158	11158	12218	12218	4023297	0.1534	0.0342	0.3154	0.2761	0.1037	0.0372	0	0.0186	UN
11562	11562	11981	11981	4023297	0.1707	0.0399	0.3194	0.3185	0.1026	0.023	0	0.0115	UN
11608	11608	12242	12242	4023297	0.1108	0.0218	0.3434	0.2113	0.0699	0.336	0.0018	0.1698	3rd
11769	11769	12218	12218	4023297	0.158	0.0413	0.3153	0.3211	0.098	0.0237	0	0.0119	UN
11887	11887	12242	12242	4023297	0.1092	0.0458	0.2627	0.4406	0.0286	0.0117	0	0.0059	UN
11959	11959	11981	11981	4023297	0.1613	0.0598	0.2635	0.4027	0.0414	0.0223	0	0.0111	UN
11981	11981	12218	12218	4023297	0.149	0.0527	0.2613	0.4465	0.0396	0	0	0	UN
11981	11981	12282	12282	4023297	0.1485	0.0393	0.2986	0.3077	0.0308	0.0188	0	0.0094	UN
11885	11885	13951	13951	4023297	0.0817	0.0221	0.2505	0.1632	0.0711	0.7296	0.0457	0.4105	2nd
11887	11887	13245	13245	4023297	0.1089	0.0465	0.2594	0.4269	0.0291	0.0039	0	0.0019	UN
11981	11981	13644	13644	4023297	0.1499	0.0476	0.2613	0.4393	0.0584	0.0181	0	0.0091	UN
12152	12152	13644	13644	4023297	0.1312	0.0379	0.2598	0.3059	0.045	0.0422	0	0.0211	UN
12242	12242	13245	13245	4023297	0.1089	0.0449	0.2627	0.4374	0.0329	0.0329	0	0.0165	UN
12242	12242	13463	13463	4023297	0.1245	0.0169	0.3338	0.1774	0.1677	0.5556	0.0568	0.3346	2nd
12552	12552	13110	13110	4023297	0.0984	0.0318	0.3155	0.2814	0.0673	0.0138	0	0.0069	UN
11769	11769	14182	14182	4023297	0.1697	0.0456	0.3171	0.3238	0.0668	0.0903	0.0038	0.049	4th
11885	11885	14068	14068	4023297	0.0938	0.015	0.315	0.1141	0.1597	0.7043	0.1658	0.518	FS
11885	11885	14204	14204	4023297	0.0816	0.0236	0.2559	0.1744	0.0725	0.6911	0.0789	0.4245	2nd
11959	11959	14182	14182	4023297	0.1717	0.0719	0.2795	0.4243	0.0305	0.0264	0	0.0132	UN
11981	11981	14182	14182	4023297	0.157	0.0613	0.2583	0.4661	0.0373	0.004	0	0.002	UN
11981	11981	14276	14276	4023297	0.1571	0.059	0.2622	0.4361	0.0491	0.0061	0	0.0031	UN
12218	12218	14182	14182	4023297	0.1722	0.0354	0.3075	0.2623	0.1144	0.0796	0	0.0398	UN
12473	12473	14182	14182	4023297	0.165	0.0679	0.2808	0.4013	0.023	0.0561	0	0.0281	UN

FID1	ID1	FID2	ID2	N_SNP	HetHet	IBS0	HetConc	HomIBS0	Kinship	IBD1Seg	IBD2Seg	PropIBD	InfType
11769	11769	14460	14460	4023297	0.1415	0.047	0.2725	0.3564	0.0527	0.0486	0	0.0243	UN
11885	11885	14435	14435	4023297	0.0891	0.0193	0.256	0.1514	0.0669	0.7074	0.0539	0.4076	2nd
11885	11885	14652	14652	4023297	0.092	0.0289	0.2879	0.2181	0.058	0.6361	0.1047	0.4228	FS
11885	11885	14756	14756	4023297	0.091	0.0122	0.2979	0.0931	0.1609	0.7187	0.1769	0.5363	FS
11981	11981	14460	14460	4023297	0.1498	0.0555	0.2625	0.4605	0.0334	0.0232	0	0.0116	UN
12218	12218	14460	14460	4023297	0.1475	0.0498	0.273	0.389	0.069	0.0391	0	0.0196	UN
11959	11959	15113	15113	4023297	0.1684	0.0613	0.2753	0.3921	0.0504	0.0046	0	0.0023	UN
11959	11959	15197	15197	4023297	0.1632	0.0671	0.273	0.3839	0.0168	0.1374	0	0.0687	4th
11981	11981	15113	15113	4023297	0.1597	0.0512	0.2665	0.445	0.0716	0	0	0	UN
11981	11981	15197	15197	4023297	0.153	0.0635	0.2608	0.4632	0.0275	0.0097	0	0.0049	UN
12218	12218	14951	14951	4023297	0.1504	0.0508	0.271	0.3294	0.0573	0.0085	0	0.0042	UN
12242	12242	14930	14930	4023297	0.1119	0.0352	0.2843	0.3399	0.0703	0.358	0.0032	0.1822	2nd
12242	12242	15156	15156	4023297	0.107	0.0472	0.2587	0.4752	0.0233	0.0066	0	0.0033	UN
12242	12242	15211	15211	4023297	0.1023	0.0329	0.2703	0.3062	0.0389	0.1916	0	0.0958	3rd
11959	11959	15568	15568	4023297	0.1647	0.048	0.3182	0.2692	0.022	0.2924	0	0.1462	3rd
12242	12242	15444	15444	4023297	0.1216	0.0218	0.3305	0.2113	0.1373	0.5961	0.0689	0.367	2nd
12242	12242	15458	15458	4023297	0.1178	0.0277	0.2955	0.2819	0.1185	0.4165	0	0.2083	2nd
11959	11959	15975	15975	4023297	0.1624	0.0446	0.2917	0.2613	0.0554	0.5877	0	0.2938	2nd
12242	12242	15845	15845	4023297	0.1077	0.0304	0.2769	0.2958	0.0749	0.2188	0	0.1094	3rd
12242	12242	16413	16413	4023297	0.1346	0.0151	0.3552	0.1619	0.2	0.6249	0.0979	0.4103	FS
12242	12242	16517	16517	4023297	0.1485	0.0001	0.4087	0.0008	0.2867	0.8431	0.1539	0.5754	PO
11158	11158	17111	17111	4023297	0.1256	0.0388	0.2805	0.2131	0.069	0.0027	0	0.0013	UN
11959	11959	16853	16853	4023297	0.2362	0.0002	0.4532	0.0013	0.3016	0.7369	0.2598	0.6283	PO
11981	11981	16815	16815	4023297	0.1571	0.0417	0.2971	0.3491	0.0655	0.0293	0	0.0146	UN
12156	12156	16890	16890	4023297	0.1096	0.044	0.2709	0.2094	0.0288	0.0251	0.0015	0.014	UN
12282	12282	16815	16815	4023297	0.1317	0.0434	0.294	0.3214	0.046	0.0086	0	0.0043	UN
11959	11959	17295	17295	4023297	0.1655	0.0669	0.2698	0.4148	0.0314	0	0	0	UN
11981	11981	17295	17295	4023297	0.1605	0.0534	0.2686	0.4446	0.0674	0	0	0	UN
12242	12242	17199	17199	4023297	0.1221	0.0223	0.3218	0.2238	0.1418	0.5606	0.0394	0.3197	2nd
11981	11981	17970	17970	4023297	0.1419	0.0519	0.252	0.4487	0.0221	0	0	0	UN
11981	11981	18528	18528	4023297	0.1478	0.0594	0.2522	0.4661	0.0278	0	0	0	UN
12218	12218	17970	17970	4023297	0.136	0.0501	0.2536	0.4001	0.0435	0	0	0	UN
12242	12242	17903	17903	4023297	0.1331	0.0001	0.3689	0.0008	0.2566	0.8994	0.0986	0.5483	PO
12242	12242	18385	18385	4023297	0.1189	0.0165	0.3254	0.1598	0.1519	0.651	0.0722	0.3977	2nd
12242	12242	18599	18599	4023297	0.1225	0.0073	0.359	0.0734	0.1961	0.304	0.004	0.156	3rd
10998	10998	19679	19679	4023297	0.0837	0.0233	0.3076	0.1772	0.0834	0.0396	0	0.0198	UN
11562	11562	18866	18866	4023297	0.1587	0.0462	0.3039	0.3432	0.0834	0	0	0	UN
11769	11769	19237	19237	4023297	0.1457	0.0526	0.2802	0.3152	0.0376	0.0049	0	0.0025	UN
11887	11887	19207	19207	4023297	0.1144	0.0453	0.2664	0.4094	0.032	0.0107	0	0.0054	UN
11981	11981	18866	18866	4023297	0.1872	0.03	0.3463	0.271	0.1635	0.1292	0	0.0646	4th
11981	11981	18929	18929	4023297	0.1594	0.0447	0.2851	0.4051	0.0776	0.0061	0	0.0031	UN
12218	12218	18929	18929	4023297	0.1363	0.0565	0.2481	0.4583	0.0335	0	0	0	UN
12218	12218	19237	19237	4023297	0.154	0.0586	0.2858	0.3589	0.0492	0.0199	0.0018	0.0118	UN
12242	12242	19181	19181	4023297	0.1512	0.0001	0.4212	0.0007	0.2924	0.8191	0.1773	0.5869	PO
12242	12242	19348	19348	4023297	0.1306	0.0176	0.3523	0.1808	0.1787	0.599	0.0995	0.399	FS
12282	12282	18866	18866	4023297	0.1566	0.0324	0.337	0.2533	0.0941	0.0555	0	0.0277	UN
12552	12552	19371	19371	4023297	0.0868	0.0242	0.2792	0.2014	0.0921	0.009	0	0.0045	UN
10998	10998	1X2124	1X2124	4023297	0.1033	0.0168	0.2791	0.1519	0.0539	0.0075	0	0.0037	UN
11562	11562	1X3796	1X3796	4023297	0.2295	0.0121	0.4001	0.0974	0.2019	0.0427	0	0.0213	UN
11608	11608	1X3656	1X3656	4023297	0.0914	0.0168	0.2691	0.1528	0.0464	0.0353	0	0.0176	UN
11770	11770	1X3837	1X3837	4023297	0.2079	0.0381	0.3436	0.2709	0.0867	0.0046	0	0.0023	UN
11885	11885	1X2816	1X2816	4023297	0.1283	0.0001	0.3885	0.0008	0.2392	0.7738	0.2243	0.6112	PO
11887	11887	1X2231	1X2231	4023297	0.1095	0.0468	0.2647	0.4295	0.0241	0.0393	0	0.0197	UN
11887	11887	1X2816	1X2816	4023297	0.1098	0.0476	0.2616	0.4629	0.0275	0.0106	0	0.0053	UN
11887	11887	1X3656	1X3656	4023297	0.1082	0.0457	0.2614	0.4336	0.0249	0.0139	0	0.0069	UN
11887	11887	1X3697	1X3697	4023297	0.1112	0.0466	0.2614	0.4536	0.0279	0.0012	0	0.0006	UN
11887	11887	1X3822	1X3822	4023297	0.1126	0.0449	0.2624	0.431	0.0323	0.032	0	0.016	UN
11887	11887	1X4080	1X4080	4023297	0.1216	0.0271	0.2984	0.2646	0.1266	0.4045	0	0.2022	2nd
11887	11887	1X4179	1X4179	4023297	0.109	0.046	0.2627	0.4326	0.0269	0.0063	0	0.0031	UN
11887	11887	1X4777	1X4777	4023297	0.1132	0.0426	0.2706	0.4056	0.0513	0.0647	0	0.0323	UN
11959	11959	1X3796	1X3796	4023297	0.21	0.0477	0.3177	0.3134	0.0962	0.03	0	0.015	UN
11959	11959	1X3837	1X3837	4023297	0.2133	0.0513	0.321	0.3394	0.0869	0.0223	0	0.0112	UN
11959	11959	1X4209	1X4209	4023297	0.2572	0.0002	0.4149	0.0016	0.2712	0.9948	0.0027	0.5001	PO
11959	11959	25409	25409	4023297	0.1769	0.0102	0.3276	0.0624	0.1842	0.2475	0.0118	0.1355	3rd
11967	11967	1X3822	1X3822	4023297	0.0956	0.027	0.2301	0.2169	0.044	0.0141	0	0.0071	UN
11981	11981	1X2816	1X2816	4023297	0.1296	0.0222	0.2537	0.2338	0.0558	0.5723	0	0.2861	2nd
11981	11981	1X3796	1X3796	4023297	0.2481	0.0003	0.4124	0.0033	0.264	0.992	0.0028	0.4988	PO
11981	11981	1X3837	1X3837	4023297	0.1945	0.0429	0.2938	0.39	0.0751	0.0146	0	0.0073	UN
11981	11981	1X4209	1X4209	4023297	0.1968	0.0427	0.2986	0.3924	0.079	0.017	0	0.0085	UN
12138	12138	1X3822	1X3822	4023297	0.1125	0.0112	0.3041	0.1088	0.1332	0.0594	0	0.0297	UN
12152	12152	1X2231	1X2231	4023297	0.1044	0.0213	0.2359	0.1779	0.09	0.0062	0	0.0031	UN
12218	12218	1X3837	1X3837	4023297	0.1998	0.04	0.3203	0.3337	0.0748	0.0329	0	0.0164	UN
12218	12218	1X4209	1X4209	4023297	0.1809	0.0481	0.2817	0.3989	0.0238	0.0064	0	0.0032	UN
12226	12226	1X3796	1X3796	4023297	0.175	0.0136	0.3033	0.1017	0.09	0.0913	0	0.0456	4th
12242	12242	1X3656	1X3656	4023297	0.1359	0.0001	0.3566	0.0014	0.2612	0.9972	0	0.4986	PO
12242	12242	1X3697	1X3697	4023297	0.1101	0.0441	0.2615	0.4589	0.0311	0.0044	0	0.0022	UN
12242	12242	1X3822	1X3822	4023297	0.1103	0.045	0.2591	0.4589	0.0232	0.0139	0	0.0069	UN
12242	12242	1X4080	1X4080	4023297	0.109	0.0458	0.2627	0.449	0.0292	0.0104	0	0.0052	UN
12242	12242	1X4179	1X4179	4023297	0.1141	0.0331	0.282	0.3408	0.0917	0.2474	0	0.1237	3rd
12242	12242	1X4777	1X4777	4023297	0.1101	0.0443	0.2646	0.4438	0.035	0.0322	0	0.0161	UN
12242	12242	25354	25354	4023297	0.1098	0.0291	0.2835	0.2795	0.0849	0.3138	0.0016	0.1585	3rd
12282	12282	1X3796	1X3796	4023297	0.1777	0.0181	0.3141	0.1396	0.0729	0.0102	0	0.0051	UN
12423	12423	1X4209	1X4209	4023297	0.1823	0.0145	0.3108	0.0892	0.1001	0.1427	0	0.0714	4th
12427	12427	1X3822	1X3822	4023297	0.1102	0.014	0.2803	0.1296	0.1266	0.0611	0	0.0305	UN
12457	12457	1X2231	1X2231	4023297	0.0895	0.0322	0.2191	0.2411	0.033	0.0021	0	0.0011	UN
12473	12473	1X3796	1X3796	4023297	0.1944	0.0492	0.3023	0.322	0.0563	0.0102	0	0.0051	UN
12473	12473	1X3837	1X3837	4023297	0.2402	0.0051	0.3977	0.0368	0.2358	0.3518	0	0.1759	3rd
12473	12473	1X4209	1X4209	4023297	0.1947	0.0517	0.3001	0.3437	0.0459	0.0061	0	0.0031	UN
12481	12481	1X2231	1X2231	4023297	0.0934	0.0176	0.2779	0.1508	0.0426	0.0752	0	0.0376	UN
12552	12552	1X2231	1X2231	4023297	0.1028	0.0136	0.2919	0.1248	0.1145	0.0662	0	0.0331	UN
12681	12681	1X4209	1X4209	4023297	0.1805	0.0085	0.3273	0.0433	0.0984	0.4429	0	0.2215	2nd
11562	11562	26498	26498	4023297	0.1658	0.0406	0.2723	0.39	0.0402	0	0	0	UN
11769	11769	26498	26498	4023297	0.1591	0.0396	0.2641	0.3983	0.0241	0	0	0	UN
11959	11959	26498	26498	4023297	0.2017	0.0435	0.3146	0.3518	0.1139	0	0	0	UN
11981	11981	26498	26498	4023297	0.1884	0.0291	0.2975	0.3682	0.1268	0.0034	0	0.0017	UN
11981	11981	27622	27622	4023297	0.1406	0.0141	0.3058	0.1042	0.082	0.2354	0	0.1177	3rd
12218	12218	26498	26498	4023297	0.1718	0.0357	0.2785	0.3858	0.0718	0	0	0	UN
12242	12242	26988	26988	4023297	0.1366	0.0001	0.3709	0.001	0.2633	0.9286	0.0675	0.5318	PO

FID1	ID1	FID2	ID2	N_SNP	HetHet	IBS0	HetConc	HomIBS0	Kinship	IBD1Seg	IBD2Seg	PropIBD	InfType
12242	12242	27503	27503	4023297	0.1121	0.0287	0.2863	0.2787	0.0961	0.4464	0	0.2232	2nd
12473	12473	26498	26498	4023297	0.1764	0.0522	0.2788	0.416	0.0429	0	0	0	UN
10998	10998	28246	28246	4023297	0.094	0.0251	0.2684	0.2288	0.0233	0.074	0	0.037	UN
12242	12242	28246	28246	4023297	0.1489	0.0001	0.4048	0.001	0.2867	0.8536	0.1448	0.5716	PO
11981	11981	28740	28740	4023297	0.1457	0.0095	0.3207	0.0681	0.1132	0.2869	0.0014	0.1448	3rd
11562	11562	30917	30917	4023297	0.1504	0.0444	0.2788	0.4115	0.0696	0	0	0	UN
11562	11562	30920	30920	4023297	0.1522	0.047	0.2578	0.4164	0.0244	0	0	0	UN
11769	11769	30917	30917	4023297	0.13	0.0464	0.2378	0.4473	0.0237	0	0	0	UN
11887	11887	30917	30917	4023297	0.1318	0.0351	0.2668	0.4046	0.0259	0.0013	0	0.0007	UN
11959	11959	30920	30920	4023297	0.1797	0.0562	0.2849	0.4215	0.0746	0	0	0	UN
11981	11981	30917	30917	4023297	0.2038	0.0122	0.3824	0.1526	0.238	0.0122	0	0.0061	UN
11981	11981	30920	30920	4023297	0.176	0.0362	0.287	0.4067	0.1128	0	0	0	UN
12218	12218	30917	30917	4023297	0.1503	0.038	0.2715	0.3959	0.0955	0	0	0	UN
12218	12218	30920	30920	4023297	0.1593	0.0424	0.2668	0.417	0.0574	0	0	0	UN
12242	12242	30917	30917	4023297	0.1289	0.0326	0.2622	0.4093	0.0253	0	0	0	UN
12473	12473	30920	30920	4023297	0.164	0.0575	0.2676	0.4288	0.0332	0	0	0	UN
11769	11769	31011	31011	4023297	0.1452	0.0504	0.2782	0.4055	0.0426	0	0	0	UN
11887	11887	31016	31016	4023297	0.1128	0.0228	0.289	0.2154	0.1129	0.1422	0	0.0711	4th
11981	11981	30933	30933	4023297	0.1479	0.0484	0.2521	0.4268	0.0592	0.0126	0	0.0063	UN
11981	11981	31011	31011	4023297	0.1607	0.0382	0.2839	0.3674	0.1025	0.1128	0	0.0564	4th
11981	11981	31065	31065	4023297	0.1549	0.0468	0.2657	0.4084	0.0748	0.0107	0	0.0053	UN
11981	11981	31145	31145	4023297	0.1418	0.0468	0.2614	0.45	0.023	0.0124	0	0.0062	UN
12218	12218	30933	30933	4023297	0.1499	0.0527	0.2716	0.4211	0.0536	0	0	0	UN
12218	12218	31011	31011	4023297	0.1547	0.0443	0.2868	0.3765	0.0908	0.0028	0	0.0014	UN
12242	12242	30961	30961	4023297	0.1046	0.0387	0.2649	0.3832	0.0359	0.1056	0	0.0528	4th
12242	12242	31145	31145	4023297	0.1178	0.0325	0.2618	0.3353	0.055	0.3177	0	0.1589	3rd
11769	11769	31337	31337	4023297	0.1446	0.0478	0.2803	0.3832	0.0552	0.0031	0	0.0016	UN
11959	11959	31331	31331	4023297	0.1613	0.0386	0.2864	0.2603	0.0754	0.1502	0	0.0751	4th
11981	11981	31331	31331	4023297	0.1502	0.0438	0.2716	0.4013	0.0585	0.0321	0.0016	0.0177	UN
11981	11981	31364	31364	4023297	0.1422	0.0458	0.2599	0.4113	0.031	0.0165	0	0.0082	UN
11981	11981	31408	31408	4023297	0.143	0.0528	0.253	0.4326	0.0237	0.0305	0	0.0152	UN
11981	11981	31410	31410	4023297	0.1445	0.0535	0.2524	0.4262	0.0294	0.019	0	0.0095	UN
12218	12218	31337	31337	4023297	0.1426	0.0497	0.2615	0.4149	0.0621	0.0054	0	0.0027	UN
12218	12218	31408	31408	4023297	0.1424	0.0521	0.267	0.3921	0.0494	0	0	0	UN
11158	11158	31497	31497	4023297	0.1439	0.0406	0.286	0.3257	0.06	0.0358	0	0.0179	UN
11769	11769	31497	31497	4023297	0.1464	0.0472	0.2813	0.373	0.055	0.0043	0	0.0022	UN
11981	11981	31497	31497	4023297	0.1433	0.0531	0.2457	0.4675	0.0346	0.0019	0	0.001	UN
11981	11981	31498	31498	4023297	0.1463	0.0485	0.2633	0.4541	0.0372	0.0076	0	0.0038	UN
11981	11981	31542	31542	4023297	0.1528	0.0123	0.321	0.1114	0.1321	0.0702	0	0.0351	UN
11981	11981	31656	31656	4023297	0.1433	0.0492	0.2519	0.4274	0.0372	0.0083	0	0.0041	UN
12218	12218	31497	31497	4023297	0.1737	0.0347	0.334	0.3049	0.1466	0.0756	0.002	0.0398	UN
12218	12218	31498	31498	4023297	0.1352	0.0534	0.2532	0.4491	0.0306	0	0	0	UN
12218	12218	31656	31656	4023297	0.1473	0.0496	0.2768	0.3936	0.0663	0	0	0	UN
11981	11981	31805	31805	4023297	0.1439	0.0502	0.2573	0.4546	0.0291	0.0054	0	0.0027	UN
11981	11981	31970	31970	4023297	0.1459	0.041	0.2806	0.3607	0.0358	0.0414	0	0.0207	UN
12218	12218	31805	31805	4023297	0.1363	0.0536	0.2553	0.4415	0.0321	0	0	0	UN
12218	12218	31903	31903	4023297	0.1474	0.0583	0.2611	0.3845	0.0262	0	0	0	UN
12242	12242	31720	31720	4023297	0.0993	0.0416	0.2344	0.3684	0.0278	0.0177	0	0.0089	UN
11887	11887	32025	32025	4023297	0.1092	0.0467	0.2567	0.4371	0.0253	0.0136	0	0.0068	UN
11981	11981	32089	32089	4023297	0.1666	0.0487	0.2901	0.3473	0.0872	0.2419	0.0038	0.1248	3rd
11981	11981	32187	32187	4023297	0.1471	0.0533	0.2543	0.4259	0.039	0.0452	0	0.0226	UN
11981	11981	32224	32224	4023297	0.1487	0.0441	0.2653	0.4173	0.0586	0.0686	0.0012	0.0355	UN
11981	11981	32358	32358	4023297	0.1465	0.0527	0.262	0.4344	0.0272	0.0295	0	0.0147	UN
12218	12218	32187	32187	4023297	0.1386	0.056	0.2501	0.4103	0.0341	0	0	0	UN
12218	12218	32358	32358	4023297	0.1367	0.055	0.255	0.4144	0.0303	0	0	0	UN
12242	12242	32043	32043	4023297	0.1212	0.029	0.2844	0.3	0.0946	0.3799	0	0.19	2nd
11981	11981	32849	32849	4023297	0.1407	0.0463	0.2583	0.4365	0.0241	0.0038	0	0.0019	UN
12242	12242	32849	32849	4023297	0.1224	0.0293	0.274	0.3003	0.0751	0.4116	0	0.2058	2nd
12242	12242	33083	33083	4023297	0.0993	0.0385	0.2424	0.3501	0.0343	0.0261	0	0.0131	UN
11693	11693	7091	7091	4023297	0.1062	0.0357	0.2373	0.3137	0.0588	0.0155	0	0.0077	UN
11769	11769	34857	34857	4023297	0.1219	0.0516	0.2397	0.4268	0.0284	0.0042	0	0.0021	UN
11885	11885	6955	6955	4023297	0.0994	0.0221	0.2643	0.1836	0.0297	0.69	0	0.345	2nd
11885	11885	7267	7267	4023297	0.1284	0.0001	0.3693	0.0006	0.2169	0.8346	0.1606	0.5779	PO
11885	11885	7311	7311	4023297	0.097	0.0234	0.2686	0.1934	0.0387	0.6891	0.0031	0.3476	2nd
11885	11885	7625	7625	4023297	0.0974	0.0225	0.2591	0.1866	0.025	0.6501	0	0.3251	2nd
11885	11885	8395	8395	4023297	0.0999	0.0248	0.2673	0.2037	0.019	0.6891	0	0.3445	2nd
11887	11887	6265	6265	4023297	0.1127	0.0453	0.2621	0.4347	0.0297	0.0075	0	0.0038	UN
11887	11887	6955	6955	4023297	0.1133	0.0449	0.2616	0.4307	0.029	0.0086	0	0.0043	UN
11887	11887	7091	7091	4023297	0.1136	0.0447	0.2669	0.4332	0.0367	0.013	0	0.0065	UN
11887	11887	7311	7311	4023297	0.1154	0.0362	0.279	0.3523	0.0808	0.2348	0	0.1174	3rd
11887	11887	7625	7625	4023297	0.1135	0.0458	0.2633	0.4387	0.0279	0.0068	0	0.0034	UN
11887	11887	8170	8170	4023297	0.1137	0.0417	0.2708	0.41	0.0538	0.1133	0	0.0567	4th
11887	11887	8307	8307	4023297	0.1171	0.0352	0.2844	0.3447	0.0874	0.2419	0	0.1209	3rd
11887	11887	8395	8395	4023297	0.1133	0.045	0.2626	0.4313	0.0298	0.0104	0	0.0052	UN
11959	11959	6716	6716	4023297	0.1819	0.0637	0.2891	0.4294	0.058	0.0112	0	0.0056	UN
11959	11959	8344	8344	4023297	0.1827	0.0605	0.2935	0.3255	0.071	0.166	0	0.083	4th
11981	11981	34568	34568	4023297	0.172	0.0424	0.3012	0.3149	0.1127	0.2971	0.0041	0.1526	3rd
11981	11981	6265	6265	4023297	0.131	0.0345	0.2505	0.3478	0.0235	0.2998	0	0.1499	3rd
11981	11981	6716	6716	4023297	0.1794	0.0318	0.2939	0.3169	0.1287	0.326	0	0.163	3rd
11981	11981	7267	7267	4023297	0.1322	0.0349	0.2513	0.352	0.0275	0.2872	0	0.1436	3rd
11981	11981	7311	7311	4023297	0.1261	0.0308	0.2452	0.3103	0.0165	0.3719	0.0015	0.1874	2nd
11981	11981	7625	7625	4023297	0.134	0.029	0.257	0.2974	0.0495	0.3938	0	0.1969	2nd
11981	11981	8307	8307	4023297	0.1275	0.0306	0.2488	0.3095	0.0192	0.3851	0	0.1925	2nd
11981	11981	8395	8395	4023297	0.131	0.0358	0.2497	0.3592	0.0202	0.2608	0.0013	0.1317	3rd
12218	12218	34857	34857	4023297	0.1329	0.0489	0.2533	0.4249	0.0327	0.003	0	0.0015	UN
12218	12218	8344	8344	4023297	0.1705	0.0529	0.2937	0.3448	0.0473	0.0667	0	0.0333	UN
12242	12242	33863	33863	4023297	0.1287	0.018	0.3082	0.1904	0.1527	0.6213	0.0016	0.3122	2nd
12242	12242	34857	34857	4023297	0.1194	0.0287	0.2626	0.2975	0.0669	0.3687	0.0019	0.1862	2nd
12242	12242	7937	7937	4023297	0.1073	0.0463	0.2628	0.4562	0.0253	0.0218	0	0.0109	UN
12473	12473	6716	6716	4023297	0.1682	0.0618	0.276	0.4169	0.0267	0.0059	0	0.003	UN
12473	12473	8344	8344	4023297	0.1715	0.0657	0.2858	0.362	0.0247	0.0565	0	0.0282	UN
11608	11608	9841	9841	4023297	0.1052	0.0239	0.3177	0.2254	0.038	0.2237	0	0.1119	3rd
11885	11885	9045	9045	4023297	0.0968	0.0288	0.2689	0.2327	0.0126	0.6598	0	0.3299	2nd
11887	11887	8780	8780	4023297	0.1118	0.0449	0.2615	0.4446	0.0328	0.0041	0	0.0021	UN
11887	11887	9045	9045	4023297	0.1124	0.0427	0.2708	0.4081	0.049	0.0956	0	0.0478	4th
11887	11887	9128	9128	4023297	0.1126	0.0454	0.2611	0.4369	0.028	0.0073	0	0.0037	UN
11887	11887	9562	9562	4023297	0.1142	0.0439	0.2693	0.4248	0.0419	0.0233	0	0.0117	UN
11887	11887	9841	9841	4023297	0.1089	0.0446	0.2602	0.4304	0.0351	0.0112	0	0.0056	UN

FID1	ID1	FID2	ID2	N_SNP	HetHet	IBS0	HetConc	HomIBS0	Kinship	IBD1Seg	IBD2Seg	PropIBD	InfType
11959	11959	8465	8465	4023297	0.1739	0.0537	0.2877	0.3695	0.0769	0.167	0	0.0835	4th
11959	11959	9656	9656	4023297	0.1864	0.0783	0.2955	0.3352	0.0232	0.0578	0	0.0289	UN
11959	11959	9860	9860	4023297	0.1687	0.0615	0.2752	0.4068	0.0515	0.0047	0	0.0023	UN
11981	11981	8465	8465	4023297	0.1751	0.0305	0.3008	0.3114	0.1483	0.3029	0.0017	0.1532	3rd
11981	11981	9045	9045	4023297	0.1301	0.0216	0.2559	0.2207	0.0576	0.58	0.0015	0.2915	2nd
11981	11981	9128	9128	4023297	0.1326	0.0326	0.2539	0.3321	0.0339	0.3362	0	0.1681	3rd
11981	11981	9860	9860	4023297	0.1641	0.0467	0.275	0.4298	0.0883	0.0028	0	0.0014	UN
12218	12218	8465	8465	4023297	0.1461	0.0528	0.2526	0.4555	0.0312	0.0042	0	0.0021	UN
12242	12242	9562	9562	4023297	0.1115	0.0439	0.2644	0.4499	0.0324	0.0055	0	0.0028	UN
12242	12242	9841	9841	4023297	0.1731	0.0067	0.4956	0.0863	0.3047	0.546	0.3114	0.5844	FS
12473	12473	9860	9860	4023297	0.1556	0.0644	0.2624	0.4243	0.0223	0.0014	0	0.0007	UN
12719	12719	13644	13644	4023297	0.1256	0.0415	0.2462	0.3413	0.0222	0.0115	0	0.0057	UN
12962	12962	13644	13644	4023297	0.128	0.0351	0.2587	0.2936	0.0391	0.0195	0	0.0097	UN
13169	13169	13644	13644	4023297	0.1247	0.0356	0.2523	0.2992	0.0282	0.0195	0	0.0098	UN
13245	13245	13575	13575	4023297	0.1087	0.0362	0.2353	0.3065	0.0284	0.041	0	0.0205	UN
13387	13387	13644	13644	4023297	0.1353	0.0346	0.2675	0.2958	0.0665	0.0531	0	0.0266	UN
13673	13673	13951	13951	4023297	0.073	0.0275	0.217	0.1981	0.0225	0.2455	0.0703	0.193	2nd
13698	13698	13951	13951	4023297	0.09	0.0389	0.275	0.2967	0.0172	0.5109	0.0585	0.314	2nd
13739	13739	13942	13942	4023297	0.1008	0.0381	0.2659	0.2791	0.0233	0.0495	0.0068	0.0316	UN
13739	13739	13951	13951	4023297	0.0867	0.0275	0.2455	0.2105	0.0601	0.1948	0.077	0.1744	3rd
13942	13942	13951	13951	4023297	0.0938	0.0238	0.2507	0.1902	0.0611	0.19	0.0252	0.1202	3rd
13698	13698	14012	14012	4023297	0.098	0.0166	0.2857	0.1383	0.1174	0.7389	0.0499	0.4193	2nd
13698	13698	14068	14068	4023297	0.0869	0.0225	0.2766	0.1701	0.0987	0.7047	0.0824	0.4348	FS
13698	13698	14204	14204	4023297	0.0847	0.0248	0.2605	0.191	0.0827	0.6274	0.0757	0.3894	2nd
13739	13739	14012	14012	4023297	0.092	0.0388	0.2478	0.2927	0.0194	0.2028	0.0158	0.1172	3rd
13942	13942	14012	14012	4023297	0.1039	0.0216	0.2681	0.181	0.1103	0.2179	0.0296	0.1386	3rd
13942	13942	14204	14204	4023297	0.0906	0.0263	0.245	0.2051	0.0346	0.1813	0.0276	0.1183	3rd
13951	13951	14012	14012	4023297	0.1061	0.0317	0.3075	0.2602	0.0725	0.5648	0.0757	0.3581	2nd
13951	13951	14068	14068	4023297	0.0922	0.0387	0.2887	0.2849	0.0169	0.5748	0.0772	0.3646	2nd
13951	13951	14204	14204	4023297	0.1067	0.0229	0.3401	0.186	0.1387	0.6143	0.162	0.4691	FS
13110	13110	14473	14473	4023297	0.0857	0.0345	0.2565	0.2892	0.032	0	0	0	UN
13110	13110	14668	14668	4023297	0.0884	0.0367	0.26	0.3078	0.0331	0	0	0	UN
13644	13644	14460	14460	4023297	0.1403	0.0594	0.2542	0.4802	0.0289	0.0236	0	0.0118	UN
13645	13645	14652	14652	4023297	0.0746	0.0331	0.2106	0.2383	0.012	0.1671	0.0262	0.1098	3rd
13673	13673	14756	14756	4023297	0.0764	0.025	0.2376	0.1796	0.0582	0.2913	0.1008	0.2465	2nd
13698	13698	14435	14435	4023297	0.0971	0.031	0.2779	0.2505	0.0376	0.6577	0.0248	0.3537	2nd
13698	13698	14652	14652	4023297	0.1191	0.0201	0.3946	0.1713	0.1769	0.5563	0.2834	0.5616	FS
13698	13698	14756	14756	4023297	0.0839	0.0381	0.2605	0.2781	0.0176	0.561	0.0633	0.3438	2nd
13739	13739	14435	14435	4023297	0.0937	0.0341	0.2497	0.2645	0.0375	0.2195	0.0178	0.1275	3rd
13739	13739	14652	14652	4023297	0.0887	0.0253	0.2501	0.1958	0.078	0.27	0.0384	0.1733	3rd
13942	13942	14435	14435	4023297	0.1045	0.0235	0.2661	0.1978	0.1068	0.1999	0.0145	0.1144	3rd
13942	13942	14652	14652	4023297	0.0983	0.0289	0.2634	0.2334	0.0514	0.2102	0.0418	0.1469	3rd
13942	13942	14696	14696	4023297	0.1132	0.0393	0.3161	0.2986	0.0372	0.0594	0.0211	0.0508	4th
13951	13951	14435	14435	4023297	0.1075	0.0295	0.3075	0.2498	0.0794	0.5651	0.0798	0.3624	2nd
13951	13951	14652	14652	4023297	0.1068	0.0257	0.3288	0.2158	0.1249	0.6039	0.1284	0.4303	FS
13951	13951	14756	14756	4023297	0.0937	0.0195	0.2903	0.1517	0.1212	0.7452	0.0755	0.4481	2nd
12962	12962	15190	15190	4023297	0.1002	0.0358	0.2387	0.2856	0.0277	0.0229	0	0.0114	UN
13110	13110	15190	15190	4023297	0.099	0.0315	0.2756	0.277	0.0473	0.0477	0	0.0238	UN
13245	13245	15156	15156	4023297	0.1088	0.0462	0.2616	0.4456	0.0286	0.0213	0	0.0106	UN
13644	13644	14925	14925	4023297	0.1321	0.0234	0.2951	0.2166	0.0592	0.5286	0.1219	0.3862	FS
13644	13644	15113	15113	4023297	0.1483	0.0521	0.2548	0.4436	0.0383	0	0	0	UN
13644	13644	15175	15175	4023297	0.1438	0.0291	0.3272	0.2429	0.0629	0.3831	0.0056	0.1972	2nd
13644	13644	15190	15190	4023297	0.1236	0.0181	0.2634	0.1698	0.0735	0.694	0.0053	0.3523	2nd
13859	13859	15217	15217	4023297	0.0999	0.0369	0.3014	0.2535	0.0514	0.0055	0.0526	0.0554	4th
12720	12720	15308	15308	4023297	0.1429	0.0447	0.3231	0.3083	0.0457	0.0047	0	0.0023	UN
13463	13463	15444	15444	4023297	0.1062	0.0268	0.2939	0.2393	0.1061	0.5265	0.0221	0.2853	2nd
13463	13463	15458	15458	4023297	0.1048	0.0404	0.2688	0.3743	0.0304	0.1971	0.0026	0.1011	3rd
12720	12720	15975	15975	4023297	0.1541	0.0484	0.3148	0.3159	0.0889	0.0058	0	0.0029	UN
13463	13463	15659	15659	4023297	0.0979	0.0353	0.2646	0.3215	0.0509	0.0146	0	0.0073	UN
13942	13942	16121	16121	4023297	0.1141	0.0363	0.2835	0.252	0.0734	0.0385	0.0105	0.0298	UN
13951	13951	16369	16369	4023297	0.0984	0.0255	0.247	0.2035	0.0303	0.1889	0.0471	0.1416	3rd
13463	13463	16413	16413	4023297	0.1106	0.0238	0.2902	0.2279	0.1155	0.5231	0.0042	0.2658	2nd
13463	13463	16517	16517	4023297	0.1116	0.0281	0.2947	0.2705	0.1012	0.3928	0.0287	0.2251	2nd
13698	13698	16692	16692	4023297	0.0823	0.0326	0.2471	0.2553	0.0317	0.2889	0.0427	0.1872	2nd
13942	13942	16671	16671	4023297	0.1056	0.0426	0.2558	0.3153	0.0302	0.0214	0.0164	0.0271	UN
13951	13951	16692	16692	4023297	0.0887	0.0323	0.2632	0.2562	0.054	0.31	0.0209	0.1759	3rd
13951	13951	16702	16702	4023297	0.1071	0.0309	0.3174	0.2583	0.0861	0.5182	0.0969	0.356	FS
13463	13463	17199	17199	4023297	0.1082	0.0206	0.2912	0.1933	0.1363	0.5495	0.0037	0.2785	2nd
13463	13463	17903	17903	4023297	0.1028	0.0328	0.2786	0.2932	0.0757	0.4029	0.0196	0.221	2nd
13463	13463	18144	18144	4023297	0.097	0.0371	0.2584	0.3318	0.0454	0.0054	0	0.0027	UN
13463	13463	18385	18385	4023297	0.0988	0.0267	0.2719	0.235	0.0862	0.4591	0.006	0.2356	2nd
13942	13942	18469	18469	4023297	0.1267	0.0391	0.2843	0.2766	0.0321	0.0449	0.0038	0.0262	UN
13951	13951	18557	18557	4023297	0.0869	0.0338	0.2524	0.2505	0.0411	0.2406	0.0508	0.1711	3rd
13245	13245	19207	19207	4023297	0.113	0.0452	0.2631	0.4136	0.0291	0.0087	0	0.0044	UN
13463	13463	19181	19181	4023297	0.1091	0.0333	0.2879	0.3124	0.0761	0.3688	0.0186	0.203	2nd
13463	13463	19348	19348	4023297	0.1063	0.0219	0.285	0.2041	0.1274	0.5153	0.0035	0.2611	2nd
13644	13644	18866	18866	4023297	0.1396	0.0545	0.2494	0.4583	0.0411	0.0019	0	0.001	UN
13644	13644	18929	18929	4023297	0.1346	0.0529	0.2422	0.4634	0.0382	0.0061	0	0.0031	UN
13942	13942	19012	19012	4023297	0.1195	0.0342	0.2762	0.2487	0.057	0.1143	0.0079	0.0651	4th
13951	13951	18917	18917	4023297	0.0906	0.0149	0.276	0.116	0.1373	0.3039	0.1144	0.2663	2nd
12717	12717	1X2231	1X2231	4023297	0.0965	0.0166	0.2806	0.1466	0.0696	0.0435	0	0.0218	UN
12719	12719	1X2231	1X2231	4023297	0.1087	0.0181	0.2484	0.1568	0.1113	0.0265	0	0.0132	UN
12720	12720	1X4209	1X4209	4023297	0.2203	0.0124	0.3789	0.1019	0.1811	0.0151	0	0.0076	UN
12869	12869	1X4179	1X4179	4023297	0.1026	0.0143	0.2805	0.126	0.1175	0.0015	0	0.0008	UN
12962	12962	1X2231	1X2231	4023297	0.1085	0.0155	0.2555	0.135	0.1336	0.0228	0	0.0114	UN
13110	13110	1X2231	1X2231	4023297	0.1116	0.0101	0.3101	0.0973	0.162	0.1025	0	0.0512	4th
13169	13169	1X2231	1X2231	4023297	0.1058	0.0176	0.2497	0.1541	0.1238	0.0115	0	0.0058	UN
13245	13245	1X3656	1X3656	4023297	0.1091	0.0425	0.2648	0.4117	0.0402	0.0531	0.0014	0.028	UN
13245	13245	1X3697	1X3697	4023297	0.1129	0.0418	0.2672	0.4178	0.0483	0.0365	0	0.0183	UN
13245	13245	1X3822	1X3822	4023297	0.1138	0.0423	0.2667	0.4146	0.0436	0.0386	0	0.0193	UN
13245	13245	1X4080	1X4080	4023297	0.1096	0.0462	0.2619	0.4326	0.0325	0.005	0	0.0025	UN
13245	13245	1X4179	1X4179	4023297	0.1132	0.0382	0.2762	0.3714	0.0664	0.1646	0	0.0823	4th
13245	13245	1X4777	1X4777	4023297	0.1105	0.0467	0.2632	0.4448	0.0301	0.011	0	0.0055	UN
13387	13387	1X2231	1X2231	4023297	0.1098	0.0157	0.2484	0.1387	0.1178	0.018	0	0.009	UN
13463	13463	1X3656	1X3656	4023297	0.1292	0.0188	0.3533	0.1988	0.1725	0.4181	0.1122	0.3213	2nd
13463	13463	1X4179	1X4179	4023297	0.1272	0.002	0.3442	0.0209	0.2369	0.7836	0.0158	0.4076	2nd
13597	13597	1X2231	1X2231	4023297	0.1028	0.0081	0.3281	0.0691	0.1159	0.4498	0.0043	0.2292	2nd
13644	13644	1X2231	1X2231	4023297	0.1363	0.0001	0.2903	0.0006	0.1771	0.991	0.0065	0.502	PO

FID1	ID1	FID2	ID2	N_SNP	HetHet	IBS0	HetConc	HomIBS0	Kinship	IBD1Seg	IBD2Seg	PropIBD	InfType
13644	13644	1X3796	1X3796	4023297	0.1776	0.0488	0.2759	0.4222	0.0245	0.0093	0	0.0047	UN
13644	13644	1X3837	1X3837	4023297	0.1788	0.0473	0.2753	0.418	0.0256	0.0377	0	0.0189	UN
13645	13645	1X2816	1X2816	4023297	0.1006	0.0107	0.268	0.0914	0.1239	0.3853	0.0044	0.1971	2nd
13673	13673	1X2816	1X2816	4023297	0.1044	0.0078	0.2935	0.0665	0.1387	0.5089	0.0084	0.2629	2nd
13694	13694	1X2231	1X2231	4023297	0.0974	0.0086	0.2959	0.0723	0.1049	0.3442	0.0066	0.1787	2nd
13698	13698	1X2816	1X2816	4023297	0.1338	0	0.4001	0.0004	0.2533	0.7896	0.2093	0.6041	PO
13739	13739	1X2816	1X2816	4023297	0.1288	0.0057	0.3559	0.054	0.217	0.5333	0.034	0.3006	2nd
13859	13859	1X2816	1X2816	4023297	0.1157	0.0104	0.3133	0.0924	0.1649	0.5065	0.0118	0.2651	2nd
13914	13914	1X2816	1X2816	4023297	0.106	0.0208	0.2415	0.1827	0.1072	0.0247	0	0.0123	UN
13942	13942	1X2816	1X2816	4023297	0.143	0.0049	0.3805	0.0513	0.2515	0.4031	0.0783	0.2799	2nd
13951	13951	1X2816	1X2816	4023297	0.1446	0.0001	0.4329	0.0006	0.2784	0.7713	0.2272	0.6128	PO
13951	13951	1X4080	1X4080	4023297	0.1082	0.0367	0.2927	0.312	0.0226	0.5561	0.0106	0.2887	2nd
12719	12719	26498	26498	4023297	0.1542	0.0292	0.2662	0.2971	0.0296	0	0	0	UN
12720	12720	26498	26498	4023297	0.1896	0.0284	0.3281	0.2847	0.1103	0	0	0	UN
13387	13387	26498	26498	4023297	0.1582	0.0289	0.2723	0.3022	0.0414	0.0036	0	0.0018	UN
13387	13387	27351	27351	4023297	0.1127	0.0431	0.2409	0.3353	0.0406	0.0279	0	0.0139	UN
13463	13463	26988	26988	4023297	0.1069	0.0349	0.2842	0.3229	0.0702	0.3504	0.0137	0.1889	2nd
13463	13463	27503	27503	4023297	0.0998	0.0423	0.2613	0.3776	0.0253	0.1601	0	0.0801	4th
13575	13575	26498	26498	4023297	0.1577	0.0369	0.2653	0.3779	0.0234	0	0	0	UN
13644	13644	26498	26498	4023297	0.1912	0.0187	0.3176	0.2351	0.1508	0.1163	0	0.0581	4th
13644	13644	27267	27267	4023297	0.1323	0.0171	0.2788	0.1161	0.1037	0.3011	0	0.1506	3rd
13644	13644	27351	27351	4023297	0.1929	0.0001	0.4366	0.0007	0.283	0.8782	0.1185	0.5576	PO
13644	13644	27358	27358	4023297	0.1482	0.0143	0.3568	0.1211	0.1244	0.2281	0.007	0.1211	3rd
13644	13644	27594	27594	4023297	0.146	0.0106	0.345	0.0938	0.1393	0.2508	0.0014	0.1268	3rd
13644	13644	27666	27666	4023297	0.1399	0.0147	0.3228	0.1147	0.1096	0.2774	0.0036	0.1423	3rd
12719	12719	28279	28279	4023297	0.1139	0.0429	0.2357	0.3395	0.0305	0.0149	0	0.0075	UN
13387	13387	28279	28279	4023297	0.119	0.0433	0.2462	0.3486	0.042	0.0272	0	0.0136	UN
13463	13463	28246	28246	4023297	0.111	0.0287	0.2891	0.2862	0.0924	0.4352	0.0194	0.237	2nd
13644	13644	27811	27811	4023297	0.12	0.012	0.2767	0.0878	0.061	0.4035	0.0016	0.2033	2nd
13644	13644	28279	28279	4023297	0.2078	0.0001	0.463	0.0008	0.3047	0.8835	0.1145	0.5563	PO
13951	13951	28304	28304	4023297	0.0943	0.0381	0.2992	0.2976	0.023	0.2121	0.0815	0.1875	2nd
12720	12720	30917	30917	4023297	0.1351	0.0479	0.2469	0.4492	0.031	0	0	0	UN
13245	13245	30871	30871	4023297	0.094	0.0178	0.2731	0.1522	0.0392	0.0336	0	0.0168	UN
13245	13245	30917	30917	4023297	0.1321	0.0339	0.2682	0.3976	0.0301	0	0	0	UN
13387	13387	30917	30917	4023297	0.1355	0.0369	0.2613	0.3677	0.0478	0	0	0	UN
13463	13463	30611	30611	4023297	0.102	0.0131	0.2744	0.1229	0.1584	0.0154	0	0.0077	UN
13644	13644	30917	30917	4023297	0.1551	0.0309	0.2803	0.3601	0.1248	0.0043	0	0.0021	UN
13644	13644	30920	30920	4023297	0.1638	0.0398	0.2742	0.4312	0.0739	0	0	0	UN
13387	13387	31021	31021	4023297	0.1168	0.0452	0.2371	0.3799	0.0257	0.008	0	0.004	UN
13463	13463	31145	31145	4023297	0.1244	0.0025	0.2953	0.0256	0.1775	0.6317	0	0.3158	2nd
13644	13644	30933	30933	4023297	0.1419	0.0536	0.2514	0.4592	0.0419	0.0013	0	0.0007	UN
13644	13644	31011	31011	4023297	0.1406	0.0496	0.252	0.4491	0.0571	0.0147	0	0.0073	UN
13644	13644	31021	31021	4023297	0.1421	0.0331	0.2724	0.3172	0.0953	0.3202	0.0029	0.1631	3rd
13644	13644	31065	31065	4023297	0.1418	0.0568	0.2497	0.4728	0.0305	0	0	0	UN
12720	12720	31331	31331	4023297	0.1348	0.0564	0.2619	0.4355	0.0299	0.0016	0	0.0008	UN
13387	13387	31284	31284	4023297	0.1119	0.0481	0.2354	0.3931	0.0263	0.0014	0	0.0007	UN
13644	13644	31284	31284	4023297	0.1213	0.0399	0.233	0.3655	0.0249	0.0853	0	0.0427	UN
13644	13644	31331	31331	4023297	0.1445	0.034	0.2723	0.3162	0.1015	0.1286	0	0.0643	4th
13644	13644	31337	31337	4023297	0.1373	0.0533	0.2474	0.4736	0.0421	0.0012	0	0.0006	UN
13644	13644	31417	31417	4023297	0.1349	0.0457	0.2589	0.3986	0.0385	0.0416	0	0.0208	UN
12719	12719	31498	31498	4023297	0.122	0.0412	0.248	0.3419	0.0357	0.0029	0	0.0015	UN
13644	13644	31497	31497	4023297	0.1382	0.0565	0.2468	0.4836	0.0343	0.002	0	0.001	UN
13644	13644	31498	31498	4023297	0.1459	0.0357	0.2764	0.3408	0.0975	0.1661	0	0.0831	4th
13644	13644	31525	31525	4023297	0.13	0.0465	0.2444	0.3683	0.0324	0.018	0	0.009	UN
13698	13698	31552	31552	4023297	0.0738	0.0247	0.237	0.1915	0.0369	0.011	0	0.0055	UN
13698	13698	31659	31659	4023297	0.0733	0.0268	0.226	0.2025	0.0385	0.0434	0.0012	0.0229	UN
13463	13463	31720	31720	4023297	0.103	0.0161	0.2591	0.1443	0.1222	0.0087	0	0.0043	UN
13644	13644	31805	31805	4023297	0.1317	0.0525	0.2425	0.4648	0.0248	0	0	0	UN
13644	13644	31970	31970	4023297	0.1289	0.0382	0.2534	0.3353	0.0406	0.0446	0	0.0223	UN
13698	13698	31731	31731	4023297	0.0798	0.0194	0.2512	0.1541	0.0937	0.0453	0	0.0227	UN
12720	12720	32224	32224	4023297	0.1363	0.0543	0.2627	0.4296	0.0343	0.013	0	0.0065	UN
13463	13463	32043	32043	4023297	0.111	0.0343	0.2677	0.3286	0.0368	0.2708	0	0.1354	3rd
13463	13463	32467	32467	4023297	0.087	0.0241	0.2404	0.1996	0.0603	0.0041	0	0.0021	UN
13644	13644	32187	32187	4023297	0.1394	0.0604	0.25	0.4675	0.0256	0.0063	0	0.0031	UN
13644	13644	32224	32224	4023297	0.1324	0.05	0.2414	0.4548	0.0379	0.0256	0	0.0128	UN
13698	13698	31984	31984	4023297	0.0803	0.0196	0.256	0.1574	0.0912	0.0196	0	0.0098	UN
13698	13698	32311	32311	4023297	0.0841	0.0176	0.2598	0.1429	0.1194	0.0326	0	0.0163	UN
13463	13463	32849	32849	4023297	0.113	0.0326	0.2602	0.3119	0.0251	0.3157	0	0.1578	3rd
13463	13463	33083	33083	4023297	0.1036	0.0144	0.2702	0.132	0.1452	0.0089	0	0.0045	UN
13245	13245	6265	6265	4023297	0.1126	0.0448	0.2625	0.4336	0.0306	0.0148	0	0.0074	UN
13245	13245	6955	6955	4023297	0.1137	0.0445	0.2633	0.4328	0.0301	0.014	0	0.007	UN
13245	13245	7091	7091	4023297	0.1142	0.0424	0.2692	0.4177	0.0457	0.0315	0	0.0157	UN
13245	13245	7267	7267	4023297	0.1139	0.0465	0.2635	0.45	0.0224	0.0058	0	0.0029	UN
13245	13245	7311	7311	4023297	0.109	0.0477	0.26	0.4567	0.0255	0.0114	0	0.0057	UN
13245	13245	7625	7625	4023297	0.113	0.0447	0.2625	0.4327	0.0301	0.0136	0	0.0068	UN
13245	13245	8170	8170	4023297	0.1087	0.0463	0.2567	0.4528	0.0265	0.0052	0	0.0026	UN
13245	13245	8307	8307	4023297	0.1078	0.0472	0.2568	0.4524	0.0253	0.0104	0	0.0052	UN
13245	13245	8395	8395	4023297	0.1132	0.0447	0.2628	0.4319	0.0301	0.0157	0	0.0078	UN
13463	13463	33863	33863	4023297	0.1087	0.039	0.2614	0.3669	0.0131	0.2156	0	0.1078	3rd
13463	13463	34857	34857	4023297	0.1245	0.0028	0.291	0.0291	0.1695	0.568	0.0018	0.2858	2nd
13570	13570	6716	6716	4023297	0.1348	0.0209	0.2571	0.1308	0.017	0.1104	0	0.0552	4th
13644	13644	34857	34857	4023297	0.1366	0.0494	0.2601	0.4613	0.0338	0.0116	0	0.0058	UN
13644	13644	6716	6716	4023297	0.1576	0.0492	0.2609	0.4551	0.0375	0.0339	0	0.0169	UN
13698	13698	6265	6265	4023297	0.1075	0.0186	0.2876	0.1656	0.0815	0.7591	0	0.3796	2nd
13698	13698	7267	7267	4023297	0.1046	0.0303	0.2743	0.2618	0.0114	0.6044	0	0.3022	2nd
13698	13698	7311	7311	4023297	0.0992	0.0279	0.269	0.2426	0.0319	0.5657	0	0.2829	2nd
13698	13698	8170	8170	4023297	0.1031	0.0247	0.2794	0.2197	0.0522	0.6586	0.002	0.3313	2nd
13698	13698	8307	8307	4023297	0.103	0.0267	0.2826	0.2332	0.0475	0.6359	0.0061	0.3241	2nd
13698	13698	8395	8395	4023297	0.1063	0.0235	0.2819	0.2063	0.052	0.6729	0.0012	0.3376	2nd
13739	13739	6265	6265	4023297	0.0968	0.0216	0.2378	0.1805	0.0612	0.2193	0	0.1096	3rd
13739	13739	7311	7311	4023297	0.0925	0.027	0.2327	0.2228	0.0428	0.2157	0	0.1079	3rd
13739	13739	7625	7625	4023297	0.0954	0.0284	0.2328	0.2343	0.0264	0.1988	0	0.0994	3rd
13739	13739	8307	8307	4023297	0.0944	0.0269	0.2388	0.2223	0.048	0.2011	0.0015	0.1021	3rd
13739	13739	8395	8395	4023297	0.0962	0.032	0.2348	0.2607	0.0118	0.176	0	0.088	4th
13914	13914	8395	8395	4023297	0.1045	0.0427	0.2293	0.3556	0.0336	0.0128	0	0.0064	UN
13942	13942	6265	6265	4023297	0.108	0.0319	0.2547	0.2792	0.0635	0.1274	0	0.0637	4th
13942	13942	6716	6716	4023297	0.1429	0.0249	0.2722	0.2192	0.0259	0.1862	0.0066	0.0997	3rd
13942	13942	6955	6955	4023297	0.1092	0.0274	0.2562	0.2424	0.0804	0.1174	0	0.0587	4th

FID1	ID1	FID2	ID2	N_SNP	HetHet	IBS0	HetConc	HomIBS0	Kinship	IBD1Seg	IBD2Seg	PropIBD	InfType
13942	13942	7267	7267	4023297	0.1085	0.0257	0.2535	0.2287	0.0844	0.139	0	0.0695	4th
13942	13942	7311	7311	4023297	0.1046	0.0318	0.2527	0.2778	0.0705	0.1193	0	0.0597	4th
13942	13942	7625	7625	4023297	0.1078	0.0296	0.2533	0.2608	0.0709	0.1487	0	0.0743	4th
13942	13942	8170	8170	4023297	0.1091	0.0238	0.2641	0.2159	0.1071	0.1624	0	0.0812	4th
13942	13942	8307	8307	4023297	0.1057	0.029	0.2563	0.2556	0.0841	0.1397	0	0.0698	4th
13942	13942	8395	8395	4023297	0.1081	0.0299	0.2538	0.2619	0.0697	0.135	0	0.0675	4th
13951	13951	6265	6265	4023297	0.1059	0.032	0.2746	0.2823	0.023	0.5222	0	0.2611	2nd
13951	13951	6955	6955	4023297	0.107	0.0311	0.2756	0.2746	0.0258	0.5444	0	0.2722	2nd
13951	13951	7267	7267	4023297	0.1073	0.0244	0.276	0.219	0.0567	0.5886	0	0.2943	2nd
13951	13951	7311	7311	4023297	0.1576	0.0001	0.4917	0.0006	0.3093	0.6345	0.3622	0.6795	PO
13951	13951	7625	7625	4023297	0.1072	0.0216	0.2777	0.1939	0.0733	0.6472	0	0.3236	2nd
13951	13951	8170	8170	4023297	0.1162	0.0184	0.3174	0.1729	0.1218	0.6845	0.0565	0.3988	2nd
13951	13951	8307	8307	4023297	0.1207	0.0159	0.3381	0.1499	0.1492	0.6679	0.0807	0.4146	FS
13951	13951	8395	8395	4023297	0.1085	0.0315	0.2818	0.2778	0.0292	0.5473	0	0.2736	2nd
12720	12720	8465	8465	4023297	0.1529	0.0525	0.2778	0.4246	0.0283	0.0065	0	0.0032	UN
13169	13169	9562	9562	4023297	0.1037	0.0427	0.2351	0.3666	0.0318	0.0064	0	0.0032	UN
13245	13245	8780	8780	4023297	0.1136	0.0429	0.2675	0.4335	0.0426	0.0196	0	0.0098	UN
13245	13245	9045	9045	4023297	0.1076	0.0472	0.2569	0.4487	0.024	0.0116	0	0.0058	UN
13245	13245	9128	9128	4023297	0.1137	0.0449	0.265	0.4373	0.031	0.0124	0	0.0062	UN
13245	13245	9562	9562	4023297	0.1131	0.0442	0.2667	0.4317	0.0377	0.005	0	0.0025	UN
13245	13245	9841	9841	4023297	0.1092	0.0452	0.2617	0.4409	0.0346	0.0224	0	0.0112	UN
13463	13463	9841	9841	4023297	0.136	0.0014	0.3732	0.015	0.2542	0.8283	0.0703	0.4844	2nd
13644	13644	8465	8465	4023297	0.1522	0.0478	0.2639	0.4504	0.0572	0.0239	0.0013	0.0133	UN
13644	13644	8995	8995	4023297	0.1276	0.0397	0.2535	0.37	0.0287	0.2086	0	0.1043	3rd
13644	13644	9562	9562	4023297	0.1289	0.0327	0.2619	0.3247	0.0482	0.3398	0	0.1699	3rd
13644	13644	9860	9860	4023297	0.1571	0.0458	0.2731	0.4151	0.0675	0.0416	0	0.0208	UN
13698	13698	9045	9045	4023297	0.1012	0.0243	0.2775	0.2118	0.0568	0.6339	0.0042	0.3212	2nd
13739	13739	9045	9045	4023297	0.0904	0.0302	0.227	0.2452	0.0259	0.1913	0	0.0956	3rd
13739	13739	9128	9128	4023297	0.0973	0.0199	0.2386	0.1679	0.0685	0.2393	0	0.1197	3rd
13914	13914	9045	9045	4023297	0.1014	0.0371	0.2298	0.3097	0.0351	0.0181	0	0.009	UN
13914	13914	9128	9128	4023297	0.1042	0.0412	0.229	0.3458	0.0379	0.0128	0	0.0064	UN
13942	13942	8465	8465	4023297	0.1307	0.0225	0.259	0.2027	0.0435	0.2119	0.0075	0.1135	3rd
13942	13942	8581	8581	4023297	0.1053	0.0395	0.2506	0.2941	0.0346	0.0077	0.0022	0.0061	UN
13942	13942	9045	9045	4023297	0.1031	0.0335	0.2493	0.2888	0.0626	0.1128	0	0.0564	4th
13942	13942	9128	9128	4023297	0.1068	0.0339	0.2505	0.2962	0.0521	0.116	0	0.058	4th
13951	13951	9045	9045	4023297	0.1376	0.0178	0.4061	0.1721	0.1814	0.5943	0.2074	0.5046	FS
13951	13951	9128	9128	4023297	0.1109	0.0187	0.2905	0.1703	0.0959	0.7347	0	0.3673	2nd
14012	14012	14068	14068	4023297	0.0875	0.025	0.2517	0.1929	0.0443	0.6575	0.0347	0.3635	2nd
14012	14012	14204	14204	4023297	0.0973	0.02	0.2806	0.1649	0.1012	0.6906	0.0644	0.4096	2nd
14022	14022	14324	14324	4023297	0.1038	0.0266	0.2725	0.2138	0.0505	0.2611	0.0254	0.156	3rd
14068	14068	14204	14204	4023297	0.0845	0.0246	0.2645	0.185	0.0779	0.6983	0.0554	0.4045	2nd
14182	14182	14276	14276	4023297	0.1603	0.0677	0.2633	0.4288	0.0272	0.0012	0	0.0006	UN
14012	14012	14435	14435	4023297	0.1421	0.0149	0.4199	0.1454	0.2304	0.5931	0.2408	0.5373	FS
14012	14012	14652	14652	4023297	0.1109	0.0274	0.3223	0.2345	0.1061	0.5429	0.1171	0.3885	FS
14012	14012	14696	14696	4023297	0.0939	0.0338	0.2603	0.2663	0.0372	0.1604	0.051	0.1312	3rd
14012	14012	14756	14756	4023297	0.114	0.0209	0.3498	0.1718	0.1351	0.6434	0.179	0.5007	FS
14022	14022	14690	14690	4023297	0.1035	0.0336	0.2648	0.2854	0.0275	0.3255	0.0563	0.219	2nd
14068	14068	14435	14435	4023297	0.0955	0.0231	0.2768	0.183	0.0672	0.704	0.0517	0.4037	2nd
14068	14068	14652	14652	4023297	0.0894	0.021	0.2744	0.1613	0.0945	0.7816	0.044	0.4348	2nd
14068	14068	14756	14756	4023297	0.0971	0.0264	0.3204	0.1997	0.106	0.6682	0.1169	0.451	FS
14182	14182	14460	14460	4023297	0.1659	0.0659	0.2925	0.4613	0.0176	0.2042	0.0195	0.1216	3rd
14204	14204	14435	14435	4023297	0.1018	0.0307	0.2925	0.2539	0.0533	0.5712	0.0727	0.3583	2nd
14204	14204	14652	14652	4023297	0.097	0.0281	0.2964	0.2254	0.0855	0.621	0.0799	0.3904	2nd
14204	14204	14756	14756	4023297	0.0942	0.0362	0.2994	0.2717	0.0491	0.5688	0.1143	0.3987	FS
13988	13988	14959	14959	4023297	0.0835	0.0242	0.2596	0.1916	0.0137	0.2634	0.0261	0.1578	3rd
14013	14013	15009	15009	4023297	0.1032	0.0225	0.3241	0.1891	0.061	0.299	0.0246	0.1741	3rd
14022	14022	14959	14959	4023297	0.1117	0.0222	0.2846	0.1974	0.103	0.5898	0.0802	0.3751	FS
14022	14022	15156	15156	4023297	0.1266	0.0228	0.312	0.2213	0.1454	0.5577	0.0916	0.3704	FS
14022	14022	15274	15274	4023297	0.0962	0.0199	0.2509	0.1589	0.0595	0.2521	0.0363	0.1624	3rd
14058	14058	15009	15009	4023297	0.1239	0.0123	0.395	0.113	0.1851	0.3992	0.0465	0.246	2nd
14058	14058	15150	15150	4023297	0.0792	0.034	0.2574	0.2495	0.0193	0.1683	0.0382	0.1223	3rd
14167	14167	14944	14944	4023297	0.1525	0.0492	0.3147	0.3169	0.0701	0.0015	0.003	0.0038	UN
14167	14167	15113	15113	4023297	0.1529	0.0505	0.2844	0.3506	0.0231	0.0051	0	0.0026	UN
14172	14172	15232	15232	4023297	0.1366	0.0591	0.2746	0.2101	0.0197	0.1005	0.0042	0.0545	4th
14182	14182	14951	14951	4023297	0.1793	0.0479	0.3136	0.2958	0.0969	0.3714	0.0337	0.2193	2nd
14182	14182	15113	15113	4023297	0.1614	0.0607	0.2645	0.4348	0.0482	0	0	0	UN
14191	14191	15112	15112	4023297	0.1189	0.0516	0.2828	0.35	0.0275	0	0	0	UN
14276	14276	15113	15113	4023297	0.1614	0.0631	0.2684	0.4391	0.0446	0	0	0	UN
14276	14276	15197	15197	4023297	0.1722	0.0302	0.3012	0.2047	0.1423	0.6931	0	0.3466	2nd
14290	14290	15113	15113	4023297	0.1744	0.0325	0.3484	0.2468	0.1099	0.05	0	0.025	UN
14324	14324	15156	15156	4023297	0.0971	0.0343	0.2573	0.2869	0.0111	0.1377	0.0052	0.074	4th
14022	14022	15467	15467	4023297	0.1054	0.024	0.269	0.2044	0.0773	0.6119	0.0618	0.3678	2nd
14022	14022	15562	15562	4023297	0.1067	0.0233	0.2426	0.1968	0.1071	0.2684	0.0664	0.2006	2nd
14167	14167	15599	15599	4023297	0.1583	0.0428	0.334	0.2778	0.1043	0.0077	0.0207	0.0245	UN
14172	14172	15566	15566	4023297	0.15	0.0563	0.2972	0.225	0.0334	0.0763	0.005	0.0432	UN
14330	14330	15421	15421	4023297	0.0861	0.0254	0.2703	0.1996	0.0257	0.2184	0.0159	0.1251	3rd
14013	14013	15874	15874	4023297	0.0793	0.0296	0.3062	0.22	0.0464	0.0211	0.0571	0.0676	4th
14022	14022	15944	15944	4023297	0.1176	0.0267	0.3051	0.2422	0.0958	0.4962	0.1215	0.3696	FS
14077	14077	15633	15633	4023297	0.0854	0.0224	0.3167	0.1724	0.0447	0.2808	0.0774	0.2178	2nd
14324	14324	15944	15944	4023297	0.091	0.0275	0.2568	0.2248	0.0625	0.1152	0.0266	0.0842	4th
14330	14330	15633	15633	4023297	0.0851	0.0301	0.2877	0.226	0.0296	0.2384	0.026	0.1452	3rd
14276	14276	16243	16243	4023297	0.1473	0.042	0.2642	0.2559	0.055	0.2363	0	0.1182	3rd
14012	14012	16692	16692	4023297	0.0932	0.0285	0.2618	0.2375	0.0552	0.3299	0.0187	0.1836	2nd
14012	14012	16702	16702	4023297	0.1019	0.0373	0.2784	0.3128	0.0517	0.4805	0.016	0.2562	2nd
14182	14182	16400	16400	4023297	0.1809	0.0301	0.3625	0.2009	0.1236	0.2789	0.0054	0.1448	3rd
14204	14204	16702	16702	4023297	0.0978	0.0352	0.2884	0.2791	0.0375	0.5146	0.0647	0.3221	2nd
14022	14022	16775	16775	4023297	0.102	0.0269	0.2635	0.2315	0.0489	0.2522	0.0315	0.1576	3rd
14167	14167	16780	16780	4023297	0.1785	0.0304	0.3921	0.2191	0.1763	0.028	0.0307	0.0447	4th
14182	14182	17111	17111	4023297	0.1533	0.0399	0.2991	0.2084	0.0318	0.2558	0.008	0.136	3rd
14012	14012	17222	17222	4023297	0.0902	0.04	0.239	0.3134	0.0142	0.1618	0.0099	0.0908	3rd
14167	14167	17295	17295	4023297	0.1604	0.0499	0.3033	0.3362	0.0382	0	0	0	UN
14172	14172	17833	17833	4023297	0.1556	0.046	0.3073	0.1898	0.07	0.0681	0.0034	0.0375	UN
14182	14182	17268	17268	4023297	0.1714	0.0311	0.3602	0.1801	0.0854	0.398	0.0164	0.2154	2nd
14182	14182	17295	17295	4023297	0.1633	0.0667	0.2689	0.461	0.0347	0	0	0	UN
14182	14182	17829	17829	4023297	0.169	0.0348	0.3193	0.1979	0.0964	0.1885	0.0052	0.0994	3rd
14276	14276	17295	17295	4023297	0.1627	0.0651	0.2715	0.4385	0.0418	0.0017	0	0.0009	UN
14290	14290	17295	17295	4023297	0.1781	0.0351	0.3591	0.2634	0.1079	0.0448	0	0.0224	UN
14167	14167	18528	18528	4023297	0.155	0.0528	0.3037	0.3429	0.0396	0	0	0	UN

FID1	ID1	FID2	ID2	N_SNP	HetHet	IBS0	HetConc	HomIBS0	Kinship	IBD1Seg	IBD2Seg	PropIBD	InfType
14182	14182	17970	17970	4023297	0.1551	0.0511	0.2757	0.3738	0.0351	0.0721	0	0.036	UN
14182	14182	18019	18019	4023297	0.157	0.0538	0.2797	0.3278	0.0301	0.1123	0.0053	0.0615	4th
14290	14290	18528	18528	4023297	0.16	0.0424	0.3265	0.2969	0.0726	0.0066	0	0.0033	UN
14182	14182	19144	19144	4023297	0.1566	0.0494	0.2955	0.2856	0.0208	0.0996	0.006	0.0558	4th
14182	14182	19237	19237	4023297	0.1911	0.0328	0.3493	0.1979	0.1516	0.236	0.0285	0.1465	3rd
14012	14012	1X2816	1X2816	4023297	0.1523	0.0001	0.4355	0.0006	0.2918	0.7532	0.2423	0.6189	PO
14013	14013	1X3697	1X3697	4023297	0.1144	0.0069	0.3458	0.0651	0.1489	0.3917	0	0.1958	2nd
14016	14016	1X2231	1X2231	4023297	0.1048	0.0072	0.3478	0.0613	0.1193	0.5713	0.0145	0.3002	2nd
14058	14058	1X3697	1X3697	4023297	0.1129	0.0073	0.3243	0.0685	0.1514	0.3113	0.0012	0.1568	3rd
14066	14066	1X2816	1X2816	4023297	0.1015	0.0072	0.3198	0.0636	0.1033	0.4455	0.0247	0.2475	2nd
14068	14068	1X2816	1X2816	4023297	0.1311	0.0001	0.3957	0.0007	0.2464	0.7736	0.2156	0.6024	PO
14158	14158	1X4209	1X4209	4023297	0.1844	0.01	0.3172	0.0879	0.1178	0.0448	0	0.0224	UN
14182	14182	1X3796	1X3796	4023297	0.201	0.051	0.3038	0.375	0.0726	0.0182	0	0.0091	UN
14182	14182	1X3837	1X3837	4023297	0.234	0.0337	0.3683	0.2574	0.1553	0.3439	0.0025	0.1744	3rd
14182	14182	1X4209	1X4209	4023297	0.2043	0.0491	0.3076	0.37	0.0779	0.0276	0	0.0138	UN
14191	14191	1X4209	1X4209	4023297	0.1812	0.0168	0.3183	0.1319	0.0793	0	0	0	UN
14204	14204	1X2816	1X2816	4023297	0.1352	0.0001	0.4024	0.0007	0.2566	0.8044	0.1922	0.5944	PO
14276	14276	1X3796	1X3796	4023297	0.1994	0.0502	0.3047	0.3577	0.0687	0.0089	0	0.0044	UN
14276	14276	1X3837	1X3837	4023297	0.1998	0.0498	0.3023	0.3589	0.0658	0.0014	0	0.0007	UN
14276	14276	1X4209	1X4209	4023297	0.2089	0.0414	0.3208	0.3045	0.1002	0.1625	0	0.0812	4th
14342	14342	1X2231	1X2231	4023297	0.1032	0.0109	0.3234	0.0944	0.1047	0.3648	0.0113	0.1936	2nd
14343	14343	1X4179	1X4179	4023297	0.1072	0.0125	0.2816	0.1161	0.1469	0.0693	0	0.0347	UN
14013	14013	27306	27306	4023297	0.0828	0.0159	0.3081	0.1234	0.141	0.0834	0.075	0.1167	3rd
14158	14158	26498	26498	4023297	0.1672	0.0278	0.2964	0.2979	0.0556	0.0014	0	0.0007	UN
14182	14182	26498	26498	4023297	0.1887	0.0453	0.2922	0.4285	0.0897	0.0048	0	0.0024	UN
14276	14276	26355	26355	4023297	0.1742	0.006	0.3625	0.0417	0.1993	0.5329	0.0849	0.3514	2nd
14276	14276	26498	26498	4023297	0.1899	0.0429	0.2986	0.3897	0.0938	0.0015	0	0.0007	UN
14276	14276	27190	27190	4023297	0.1652	0.0076	0.341	0.046	0.1757	0.4664	0.0668	0.3	2nd
14276	14276	27193	27193	4023297	0.1779	0.0048	0.3473	0.0301	0.2147	0.4555	0.055	0.2827	2nd
14276	14276	27686	27686	4023297	0.1597	0.0124	0.3468	0.0866	0.1349	0.3556	0.0285	0.2064	2nd
14324	14324	27525	27525	4023297	0.0895	0.0214	0.3091	0.1588	0.0693	0.0168	0.0788	0.0873	4th
14013	14013	28183	28183	4023297	0.1066	0.0098	0.386	0.0849	0.1995	0.2938	0.0851	0.232	2nd
14058	14058	27989	27989	4023297	0.0845	0.0348	0.2688	0.2671	0.0134	0.0749	0.0129	0.0504	4th
14058	14058	28183	28183	4023297	0.0861	0.0273	0.2756	0.2097	0.0578	0.1693	0.0188	0.1035	3rd
14172	14172	27841	27841	4023297	0.1367	0.035	0.3172	0.1389	0.0773	0.064	0.0042	0.0362	UN
14182	14182	28281	28281	4023297	0.159	0.0516	0.2972	0.2545	0.0227	0.2504	0.0049	0.1301	3rd
14276	14276	27985	27985	4023297	0.1742	0.0049	0.3433	0.0302	0.2072	0.4916	0.0999	0.3457	2nd
14282	14282	27889	27889	4023297	0.0807	0.0179	0.3112	0.1373	0.1015	0.0426	0.0405	0.0618	4th
14330	14330	27874	27874	4023297	0.0698	0.0251	0.2786	0.1833	0.0139	0.0187	0.042	0.0513	4th
14351	14351	27999	27999	4023297	0.0686	0.0246	0.2854	0.1781	0.0393	0.033	0.0814	0.0979	3rd
14276	14276	28478	28478	4023297	0.1741	0.0064	0.3552	0.0433	0.1991	0.4202	0.0868	0.2969	2nd
14350	14350	28436	28436	4023297	0.069	0.0188	0.2794	0.1357	0.0741	0.0352	0.1066	0.1242	3rd
14068	14068	30752	30752	4023297	0.0718	0.0252	0.2345	0.1865	0.0361	0.0127	0	0.0063	UN
14182	14182	30917	30917	4023297	0.1509	0.0542	0.2522	0.4901	0.0395	0	0	0	UN
14182	14182	30920	30920	4023297	0.1746	0.0499	0.2783	0.434	0.0805	0	0	0	UN
14276	14276	30917	30917	4023297	0.1526	0.0549	0.2594	0.4754	0.0457	0	0	0	UN
14276	14276	30920	30920	4023297	0.1803	0.044	0.2941	0.3714	0.0997	0	0	0	UN
14182	14182	30933	30933	4023297	0.1658	0.0548	0.2851	0.4	0.0574	0.0443	0	0.0221	UN
14182	14182	31011	31011	4023297	0.1718	0.0463	0.3025	0.3572	0.0859	0.173	0.0028	0.0893	3rd
14022	14022	31297	31297	4023297	0.1418	0.0019	0.3467	0.0187	0.2466	0.8475	0.0014	0.4252	2nd
14022	14022	31299	31299	4023297	0.1366	0.0052	0.3413	0.0494	0.2322	0.37	0.0014	0.1864	2nd
14022	14022	31358	31358	4023297	0.103	0.0271	0.2192	0.1815	0.062	0.0504	0	0.0252	UN
14022	14022	31364	31364	4023297	0.123	0.007	0.2665	0.0635	0.1622	0.1784	0	0.0892	3rd
14068	14068	31484	31484	4023297	0.078	0.0197	0.2473	0.1511	0.0965	0.0351	0	0.0176	UN
14182	14182	31337	31337	4023297	0.2063	0.0064	0.3916	0.0523	0.2487	0.5017	0	0.2508	2nd
14182	14182	31408	31408	4023297	0.1806	0.0068	0.334	0.0518	0.2087	0.2634	0.0024	0.1342	3rd
14182	14182	31417	31417	4023297	0.1706	0.0072	0.3243	0.0572	0.188	0.2429	0	0.1215	3rd
14182	14182	31477	31477	4023297	0.1746	0.0091	0.3158	0.0558	0.194	0.2775	0	0.1387	3rd
14276	14276	31410	31410	4023297	0.1471	0.0546	0.2561	0.3632	0.0267	0.056	0	0.028	UN
14022	14022	31559	31559	4023297	0.1065	0.0114	0.277	0.1021	0.1321	0.0831	0	0.0415	UN
14182	14182	31497	31497	4023297	0.1865	0.038	0.3373	0.2911	0.1303	0.2146	0.0213	0.1286	3rd
14182	14182	31656	31656	4023297	0.1688	0.0506	0.3034	0.3673	0.0614	0.0361	0.0012	0.0192	UN
14022	14022	31738	31738	4023297	0.1213	0.0081	0.2736	0.0698	0.1735	0.156	0	0.078	4th
14022	14022	31764	31764	4023297	0.1252	0.0069	0.301	0.0617	0.2049	0.2218	0.0012	0.1121	3rd
14022	14022	31852	31852	4023297	0.0948	0.0332	0.2111	0.2236	0.0511	0.029	0	0.0145	UN
14022	14022	31882	31882	4023297	0.1106	0.0136	0.2901	0.119	0.1312	0.0615	0	0.0308	UN
14022	14022	31952	31952	4023297	0.1246	0.0085	0.3165	0.0783	0.1931	0.1381	0	0.0691	4th
14068	14068	31693	31693	4023297	0.0765	0.022	0.2447	0.167	0.0772	0.008	0	0.004	UN
14182	14182	31903	31903	4023297	0.1924	0.01	0.3405	0.0689	0.2203	0.1548	0.0018	0.0791	4th
14191	14191	31800	31800	4023297	0.1305	0.0288	0.2856	0.2104	0.092	0.0023	0	0.0011	UN
14022	14022	32025	32025	4023297	0.1331	0.002	0.3264	0.0191	0.2381	0.8406	0	0.4203	2nd
14022	14022	32040	32040	4023297	0.137	0.002	0.3242	0.0189	0.2295	0.8077	0.0012	0.405	2nd
14022	14022	32103	32103	4023297	0.0926	0.0331	0.2141	0.2195	0.0351	0.0339	0	0.017	UN
14182	14182	32053	32053	4023297	0.1466	0.0236	0.2838	0.1443	0.0769	0.0671	0	0.0336	UN
14182	14182	32130	32130	4023297	0.142	0.0249	0.2836	0.1547	0.0489	0.0504	0	0.0252	UN
14182	14182	32187	32187	4023297	0.1918	0.0052	0.351	0.0392	0.2313	0.3656	0.0058	0.1886	2nd
14182	14182	32356	32356	4023297	0.1426	0.0275	0.275	0.1652	0.0539	0.0687	0	0.0344	UN
14276	14276	32224	32224	4023297	0.1479	0.0435	0.2614	0.3271	0.0562	0.2816	0	0.1408	3rd
14022	14022	32909	32909	4023297	0.1162	0.0132	0.3038	0.1151	0.149	0.0492	0.0013	0.0259	UN
14182	14182	32557	32557	4023297	0.1394	0.0276	0.2777	0.1726	0.032	0.0417	0	0.0208	UN
14182	14182	32876	32876	4023297	0.15	0.0266	0.3006	0.1615	0.0623	0.0468	0	0.0234	UN
14182	14182	33082	33082	4023297	0.1527	0.0196	0.2889	0.12	0.1117	0.0987	0	0.0493	4th
14012	14012	6265	6265	4023297	0.1422	0.016	0.3811	0.1636	0.1893	0.5893	0.1557	0.4504	FS
14012	14012	6955	6955	4023297	0.1477	0.0147	0.3982	0.1516	0.2028	0.602	0.1839	0.4849	FS
14012	14012	7267	7267	4023297	0.1181	0.0243	0.2942	0.2301	0.0993	0.5819	0	0.291	2nd
14012	14012	7311	7311	4023297	0.1136	0.0301	0.2926	0.2795	0.0839	0.5617	0	0.2808	2nd
14012	14012	7625	7625	4023297	0.1376	0.0198	0.3631	0.1956	0.1625	0.5745	0.1169	0.4041	FS
14012	14012	8170	8170	4023297	0.1177	0.0194	0.3033	0.1884	0.1335	0.7096	0.0012	0.3559	2nd
14012	14012	8307	8307	4023297	0.1156	0.0187	0.3	0.1781	0.137	0.6913	0.0012	0.3468	2nd
14012	14012	8395	8395	4023297	0.1556	0	0.4305	0.0005	0.283	0.8049	0.1915	0.594	PO
14022	14022	33163	33163	4023297	0.0913	0.0332	0.2123	0.2173	0.0283	0.0368	0	0.0184	UN
14022	14022	33908	33908	4023297	0.1139	0.0336	0.2621	0.3	0.0806	0.4281	0.0343	0.2484	2nd
14068	14068	6265	6265	4023297	0.0982	0.0266	0.2602	0.2203	0.0122	0.6438	0	0.3219	2nd
14068	14068	7267	7267	4023297	0.1382	0.0001	0.4045	0.0012	0.2419	0.7502	0.2377	0.6128	PO
14068	14068	7625	7625	4023297	0.0996	0.0245	0.2642	0.2034	0.0249	0.671	0	0.3355	2nd
14068	14068	8170	8170	4023297	0.097	0.0269	0.263	0.2256	0.02	0.642	0.0012	0.3222	2nd
14068	14068	8307	8307	4023297	0.096	0.025	0.2626	0.2077	0.0322	0.6676	0	0.3338	2nd
14068	14068	8395	8395	4023297	0.1015	0.0239	0.2699	0.1988	0.0317	0.6896	0	0.3448	2nd

FID1	ID1	FID2	ID2	N_SNP	HetHet	IBS0	HetConc	HomIBS0	Kinship	IBD1Seg	IBD2Seg	PropIBD	InfType
14077	14077	7937	7937	4023297	0.0943	0.0111	0.302	0.0943	0.0645	0.3866	0.0022	0.1954	2nd
14154	14154	7937	7937	4023297	0.1056	0.0106	0.3264	0.0969	0.1239	0.2954	0.0024	0.1501	3rd
14181	14181	7158	7158	4023297	0.1279	0.0197	0.2713	0.085	0.0717	0.442	0.0218	0.2428	2nd
14182	14182	34568	34568	4023297	0.156	0.0652	0.2601	0.4047	0.0201	0.1363	0	0.0682	4th
14182	14182	6716	6716	4023297	0.1791	0.0548	0.2871	0.4219	0.0732	0.0176	0	0.0088	UN
14182	14182	7158	7158	4023297	0.1975	0.0001	0.3629	0.0006	0.2541	0.8446	0.1505	0.5728	PO
14182	14182	8344	8344	4023297	0.1927	0.0457	0.3192	0.282	0.1181	0.5338	0.0017	0.2686	2nd
14204	14204	6265	6265	4023297	0.1006	0.0296	0.2621	0.2566	0.0137	0.5584	0	0.2792	2nd
14204	14204	6955	6955	4023297	0.1065	0.0266	0.2794	0.233	0.0387	0.6391	0	0.3196	2nd
14204	14204	7267	7267	4023297	0.105	0.0215	0.2736	0.1902	0.0585	0.673	0	0.3365	2nd
14204	14204	7311	7311	4023297	0.126	0.0211	0.3655	0.1959	0.1331	0.5427	0.1613	0.4326	FS
14204	14204	7625	7625	4023297	0.1024	0.0294	0.2669	0.2551	0.0174	0.5423	0	0.2711	2nd
14204	14204	8170	8170	4023297	0.1125	0.0271	0.3104	0.2431	0.0664	0.6125	0.0465	0.3527	2nd
14204	14204	8307	8307	4023297	0.1392	0.0001	0.4205	0.0006	0.2675	0.7785	0.2185	0.6078	PO
14204	14204	8395	8395	4023297	0.1032	0.0259	0.2694	0.2266	0.0355	0.6163	0	0.3081	2nd
14276	14276	6716	6716	4023297	0.1705	0.0566	0.2733	0.4205	0.053	0.0159	0	0.0079	UN
14282	14282	7937	7937	4023297	0.0932	0.0095	0.2916	0.081	0.0786	0.3482	0.0034	0.1775	2nd
14330	14330	7937	7937	4023297	0.1013	0.0087	0.3062	0.0772	0.1242	0.3112	0.0019	0.1576	3rd
14355	14355	8344	8344	4023297	0.1419	0.0219	0.2782	0.1098	0.0333	0.3203	0.0027	0.1629	3rd
14369	14369	8344	8344	4023297	0.1537	0.0168	0.3037	0.0904	0.0836	0.357	0.0046	0.183	2nd
13988	13988	8780	8780	4023297	0.1189	0.0044	0.3628	0.0414	0.1713	0.5288	0.002	0.2664	2nd
14012	14012	9045	9045	4023297	0.1121	0.024	0.2892	0.2249	0.1085	0.5878	0.0025	0.2964	2nd
14012	14012	9128	9128	4023297	0.136	0.0175	0.3574	0.1735	0.1689	0.6489	0.0872	0.4116	FS
14022	14022	8780	8780	4023297	0.1421	0.0001	0.352	0.0007	0.2587	0.8986	0.0993	0.5486	PO
14062	14062	8653	8653	4023297	0.088	0.0107	0.3033	0.0837	0.1288	0.322	0.0692	0.2302	2nd
14068	14068	9045	9045	4023297	0.0976	0.0299	0.2694	0.2439	0.0137	0.6614	0.0012	0.3319	2nd
14167	14167	9656	9656	4023297	0.1497	0.0354	0.2591	0.1624	0.0368	0.074	0	0.037	UN
14172	14172	9656	9656	4023297	0.1743	0.019	0.3131	0.0777	0.1313	0.2975	0.0048	0.1536	3rd
14182	14182	8465	8465	4023297	0.1646	0.0621	0.2719	0.4806	0.0483	0.0183	0	0.0091	UN
14182	14182	9860	9860	4023297	0.1587	0.0592	0.2581	0.442	0.0499	0.0142	0	0.0071	UN
14204	14204	9045	9045	4023297	0.1231	0.0208	0.3557	0.1918	0.1294	0.5894	0.1408	0.4355	FS
14204	14204	9128	9128	4023297	0.1026	0.0238	0.2678	0.2095	0.0452	0.6361	0	0.3181	2nd
14250	14250	8653	8653	4023297	0.081	0.0094	0.2893	0.0705	0.0991	0.4698	0.0458	0.2807	2nd
14273	14273	9656	9656	4023297	0.1563	0.0251	0.2849	0.1026	0.0675	0.1994	0	0.0997	3rd
14276	14276	8465	8465	4023297	0.1617	0.0571	0.2695	0.4307	0.0617	0.0957	0	0.0478	4th
14276	14276	9860	9860	4023297	0.1646	0.0579	0.2741	0.4206	0.0613	0.0113	0	0.0056	UN
14324	14324	8780	8780	4023297	0.1119	0.0086	0.2977	0.0794	0.1504	0.3004	0.0061	0.1563	3rd
14343	14343	8596	8596	4023297	0.1078	0.0293	0.2695	0.2244	0.0534	0	0	0	UN
14350	14350	8653	8653	4023297	0.0822	0.0097	0.2756	0.0753	0.1187	0.3277	0.0526	0.2164	2nd
14435	14435	14652	14652	4023297	0.1106	0.0209	0.3159	0.1839	0.1288	0.6492	0.0957	0.4203	FS
14435	14435	14696	14696	4023297	0.0905	0.0257	0.2448	0.2064	0.0603	0.1819	0.0444	0.1353	3rd
14435	14435	14756	14756	4023297	0.1198	0.017	0.3679	0.1469	0.1618	0.5988	0.2369	0.5363	FS
14473	14473	14668	14668	4023297	0.1009	0.0259	0.3147	0.223	0.1083	0.0474	0.0091	0.0328	UN
14652	14652	14696	14696	4023297	0.0916	0.0368	0.2669	0.2795	0.041	0.2028	0.0275	0.1289	3rd
14652	14652	14756	14756	4023297	0.099	0.0259	0.3082	0.2033	0.0976	0.6997	0.0864	0.4362	FS
14696	14696	14756	14756	4023297	0.086	0.0341	0.2577	0.2523	0.0256	0.1991	0.0676	0.1672	3rd
14473	14473	14925	14925	4023297	0.0974	0.0306	0.2854	0.2536	0.0565	0.1216	0.0063	0.0671	4th
14473	14473	15190	15190	4023297	0.1122	0.0212	0.331	0.1902	0.1222	0.2394	0.0149	0.1346	3rd
14668	14668	14925	14925	4023297	0.0986	0.0332	0.2827	0.2755	0.0549	0.1428	0.026	0.0974	3rd
14668	14668	15190	15190	4023297	0.1326	0.0169	0.4047	0.1614	0.1947	0.3864	0.0692	0.2624	2nd
14668	14668	15267	15267	4023297	0.0869	0.031	0.273	0.2432	0.0328	0.0385	0.0094	0.0286	UN
14690	14690	14959	14959	4023297	0.1015	0.0324	0.2861	0.2776	0.0713	0.3904	0.0441	0.2393	2nd
14690	14690	15156	15156	4023297	0.1167	0.0294	0.3177	0.2791	0.0877	0.3651	0.0645	0.247	2nd
14795	14795	15009	15009	4023297	0.0933	0.029	0.268	0.2405	0.0218	0.1941	0.0257	0.1228	3rd
14795	14795	15150	15150	4023297	0.0804	0.0355	0.2587	0.2611	0.0196	0.1781	0.0456	0.1346	3rd
14850	14850	14930	14930	4023297	0.0966	0.0287	0.2706	0.2449	0.0483	0.1804	0.0101	0.1003	3rd
14850	14850	15211	15211	4023297	0.0939	0.0322	0.2803	0.2676	0.0548	0.0821	0.0063	0.0474	4th
14690	14690	15467	15467	4023297	0.1014	0.0276	0.2914	0.2366	0.1	0.4228	0.0599	0.2714	2nd
14795	14795	15509	15509	4023297	0.1146	0.0168	0.3647	0.1527	0.1558	0.3062	0.0324	0.1855	2nd
14833	14833	15467	15467	4023297	0.0933	0.0205	0.3159	0.1681	0.0633	0.3137	0.0759	0.2327	2nd
14850	14850	15444	15444	4023297	0.1048	0.022	0.3151	0.1922	0.1198	0.258	0.0625	0.1915	2nd
14850	14850	15458	15458	4023297	0.108	0.0243	0.3034	0.2177	0.0843	0.22	0.0134	0.1234	3rd
14850	14850	15475	15475	4023297	0.0909	0.0335	0.2778	0.2752	0.0539	0.0206	0.0049	0.0152	UN
14690	14690	15944	15944	4023297	0.1072	0.0178	0.3081	0.164	0.1506	0.3595	0.057	0.2367	2nd
14795	14795	16019	16019	4023297	0.08	0.0349	0.2547	0.2679	0.0176	0.0347	0.0313	0.0486	4th
14850	14850	15845	15845	4023297	0.1005	0.0318	0.2921	0.2749	0.0533	0.0951	0.0024	0.05	4th
14690	14690	16104	16104	4023297	0.0908	0.0291	0.2769	0.2354	0.0483	0.1155	0.0121	0.0698	4th
14435	14435	16692	16692	4023297	0.0969	0.0332	0.2707	0.2799	0.035	0.2951	0.0197	0.1673	3rd
14435	14435	16702	16702	4023297	0.1016	0.0355	0.273	0.3014	0.0526	0.4756	0.0075	0.2453	2nd
14652	14652	16702	16702	4023297	0.0919	0.0393	0.2579	0.3164	0.0157	0.4743	0.0087	0.2459	2nd
14668	14668	16527	16527	4023297	0.0914	0.0385	0.2652	0.3163	0.0267	0.0279	0.0161	0.03	UN
14850	14850	16408	16408	4023297	0.0893	0.0281	0.2947	0.2247	0.0587	0.0504	0.0188	0.044	UN
14850	14850	16413	16413	4023297	0.1102	0.0262	0.3138	0.2332	0.0836	0.2315	0.0345	0.1503	3rd
14850	14850	16517	16517	4023297	0.1025	0.0284	0.287	0.2481	0.0562	0.2219	0.0096	0.1205	3rd
14860	14860	16510	16510	4023297	0.0835	0.0323	0.273	0.2381	0.0309	0.2023	0.0289	0.13	3rd
14865	14865	16420	16420	4023297	0.0844	0.0285	0.3068	0.2278	0.0368	0.0419	0.0489	0.0698	4th
14690	14690	16775	16775	4023297	0.0948	0.0345	0.2741	0.2885	0.0531	0.0937	0.0068	0.0537	4th
14690	14690	17112	17112	4023297	0.1141	0.0177	0.3748	0.1584	0.1659	0.2886	0.075	0.2193	2nd
14860	14860	16986	16986	4023297	0.0863	0.0292	0.2815	0.2264	0.0501	0.1308	0.0265	0.0919	3rd
14435	14435	17222	17222	4023297	0.0948	0.04	0.2505	0.3216	0.0178	0.1338	0.0097	0.0766	4th
14850	14850	17199	17199	4023297	0.1055	0.0235	0.3068	0.2056	0.0996	0.2714	0.0353	0.171	3rd
14850	14850	17903	17903	4023297	0.099	0.0308	0.2889	0.2604	0.0583	0.2093	0.0431	0.1477	3rd
14850	14850	18385	18385	4023297	0.0987	0.0306	0.2963	0.2574	0.0703	0.207	0.0631	0.1666	3rd
14850	14850	18599	18599	4023297	0.088	0.0286	0.272	0.2334	0.0709	0.0331	0.0094	0.026	UN
14460	14460	18929	18929	4023297	0.1382	0.0584	0.2517	0.4654	0.0298	0.0239	0	0.0119	UN
14850	14850	19181	19181	4023297	0.1069	0.0173	0.3043	0.156	0.1223	0.3141	0.0203	0.1774	2nd
14850	14850	19348	19348	4023297	0.0966	0.0294	0.2738	0.2508	0.0498	0.2064	0.0224	0.1256	3rd
14435	14435	1X2816	1X2816	4023297	0.1514	0	0.4247	0.0005	0.2889	0.7803	0.2174	0.6076	PO
14460	14460	1X3697	1X3697	4023297	0.121	0.0196	0.2444	0.185	0.0833	0.6755	0	0.3377	2nd
14460	14460	1X3796	1X3796	4023297	0.1846	0.0524	0.2913	0.4158	0.0221	0.0294	0	0.0147	UN
14460	14460	1X3822	1X3822	4023297	0.1208	0.029	0.2416	0.2641	0.0521	0.5117	0	0.2558	2nd
14460	14460	1X3837	1X3837	4023297	0.195	0.0483	0.3095	0.3916	0.0443	0.1629	0.0017	0.0831	4th
14473	14473	1X2231	1X2231	4023297	0.1228	0.0082	0.3596	0.0796	0.1953	0.2487	0.0145	0.1389	3rd
14526	14526	1X3697	1X3697	4023297	0.1	0.0115	0.2939	0.1027	0.0758	0.2453	0	0.1226	3rd
14652	14652	1X2816	1X2816	4023297	0.1545	0	0.4714	0.0004	0.3008	0.6845	0.3128	0.6551	PO
14668	14668	1X2231	1X2231	4023297	0.1217	0.0051	0.3461	0.0492	0.2094	0.3046	0.0326	0.1849	2nd
14668	14668	1X4777	1X4777	4023297	0.1037	0.0309	0.2744	0.2803	0.0378	0.1249	0	0.0624	4th
14695	14695	1X2816	1X2816	4023297	0.102	0.0086	0.2845	0.0719	0.1286	0.5256	0.0265	0.2893	2nd

FID1	ID1	FID2	ID2	N_SNP	HetHet	IBS0	HetConc	HomIBS0	Kinship	IBD1Seg	IBD2Seg	PropIBD	InfType
14696	14696	1X2816	1X2816	4023297	0.1272	0.0089	0.3586	0.0858	0.197	0.4876	0.0526	0.2964	2nd
14756	14756	1X2816	1X2816	4023297	0.145	0	0.4502	0.0005	0.2811	0.7024	0.2941	0.6453	PO
14795	14795	1X3697	1X3697	4023297	0.1157	0.0056	0.3308	0.0539	0.1693	0.3291	0.0066	0.1712	3rd
14850	14850	25354	25354	4023297	0.1034	0.0319	0.3028	0.2735	0.0594	0.1363	0.0151	0.0832	4th
14852	14852	1X2231	1X2231	4023297	0.0909	0.0152	0.2749	0.1251	0.0402	0.2422	0.0077	0.1287	3rd
14860	14860	1X4179	1X4179	4023297	0.1345	0.0006	0.4314	0.0055	0.2603	0.7116	0.2633	0.6191	PO
14867	14867	1X2231	1X2231	4023297	0.0932	0.0129	0.2935	0.107	0.0479	0.3236	0.0083	0.1701	3rd
14886	14886	1X2231	1X2231	4023297	0.0952	0.0091	0.3091	0.0767	0.0701	0.4679	0.0067	0.2407	2nd
14909	14909	1X2231	1X2231	4023297	0.1044	0.0098	0.3212	0.0849	0.1203	0.368	0.0109	0.195	2nd
14460	14460	26498	26498	4023297	0.1757	0.0376	0.286	0.3985	0.0725	0.0119	0	0.0059	UN
14668	14668	26800	26800	4023297	0.0909	0.037	0.2742	0.3046	0.0312	0.0457	0.016	0.0388	UN
14833	14833	27525	27525	4023297	0.0601	0.0273	0.2238	0.1927	0.0122	0.0014	0.0713	0.072	4th
14850	14850	26988	26988	4023297	0.1016	0.0217	0.2894	0.1915	0.095	0.2405	0.0342	0.1545	3rd
14850	14850	27503	27503	4023297	0.1025	0.0221	0.2938	0.193	0.0966	0.2063	0.0092	0.1123	3rd
14795	14795	27989	27989	4023297	0.0949	0.0265	0.308	0.2143	0.0886	0.1119	0.0384	0.0943	3rd
14795	14795	28183	28183	4023297	0.0826	0.0284	0.2582	0.2225	0.0474	0.0998	0.0471	0.097	3rd
14833	14833	28102	28102	4023297	0.067	0.0239	0.2866	0.1716	0.0248	0.0147	0.093	0.1003	3rd
14850	14850	28246	28246	4023297	0.1024	0.0271	0.2827	0.2462	0.0564	0.3291	0.0123	0.1769	2nd
14460	14460	28635	28635	4023297	0.1248	0.0243	0.2612	0.1975	0.0639	0.5637	0.0816	0.3634	FS
14460	14460	30609	30609	4023297	0.1653	0.0083	0.3223	0.0692	0.2147	0.3093	0.0018	0.1565	3rd
14460	14460	30611	30611	4023297	0.1232	0.0135	0.2697	0.116	0.0886	0.0426	0	0.0213	UN
14460	14460	30614	30614	4023297	0.1479	0.0153	0.3232	0.1274	0.1447	0.0443	0	0.0222	UN
14460	14460	30917	30917	4023297	0.1481	0.0369	0.2659	0.3772	0.0963	0	0	0	UN
14460	14460	30920	30920	4023297	0.1635	0.0409	0.275	0.3965	0.0685	0	0	0	UN
14460	14460	31011	31011	4023297	0.1453	0.0575	0.2642	0.4694	0.0393	0.0434	0	0.0217	UN
14460	14460	31021	31021	4023297	0.1762	0.0019	0.3637	0.0176	0.2502	0.8736	0.0066	0.4434	2nd
14460	14460	31028	31028	4023297	0.1631	0.0106	0.3208	0.079	0.2036	0.1334	0.0019	0.0686	4th
14460	14460	31145	31145	4023297	0.1696	0.0009	0.3513	0.0082	0.2429	0.9764	0.0016	0.4898	PO
14460	14460	31234	31234	4023297	0.1443	0.0091	0.3084	0.0803	0.1637	0.1034	0	0.0517	4th
14652	14652	31011	31011	4023297	0.1102	0.0044	0.2405	0.0403	0.0801	0.4414	0.0013	0.222	2nd
14652	14652	31255	31255	4023297	0.0787	0.0261	0.2298	0.2028	0.048	0.0067	0.0016	0.0049	UN
14668	14668	31134	31134	4023297	0.0956	0.0327	0.2634	0.2809	0.0376	0.0072	0	0.0036	UN
14460	14460	31337	31337	4023297	0.1379	0.0561	0.2502	0.4551	0.0373	0.0737	0.0013	0.0382	UN
14435	14435	31624	31624	4023297	0.098	0.0411	0.2485	0.3587	0.0261	0.0505	0	0.0252	UN
14460	14460	31497	31497	4023297	0.1623	0.0415	0.3046	0.3469	0.1108	0.1007	0.0235	0.0738	4th
14460	14460	31498	31498	4023297	0.1882	0.0031	0.39	0.029	0.2647	0.659	0.0077	0.3371	2nd
14460	14460	31506	31506	4023297	0.1417	0.0133	0.2916	0.0954	0.149	0.1424	0	0.0712	4th
14460	14460	31560	31560	4023297	0.148	0.0097	0.2901	0.08	0.1804	0.2038	0.0019	0.1037	3rd
14652	14652	31511	31511	4023297	0.079	0.0272	0.2313	0.2005	0.0425	0.1444	0.0171	0.0892	3rd
14652	14652	31515	31515	4023297	0.0811	0.0232	0.2396	0.1827	0.0666	0.0308	0	0.0154	UN
14652	14652	31540	31540	4023297	0.084	0.0195	0.2604	0.1598	0.0815	0.0286	0	0.0143	UN
14460	14460	31720	31720	4023297	0.1483	0.0132	0.323	0.1094	0.1542	0.0261	0	0.0131	UN
14460	14460	31800	31800	4023297	0.1607	0.008	0.3211	0.0652	0.2067	0.1752	0	0.0876	4th
14460	14460	31966	31966	4023297	0.1491	0.0097	0.3201	0.0843	0.1714	0.0751	0	0.0375	UN
14668	14668	31849	31849	4023297	0.0901	0.0277	0.2955	0.2201	0.0474	0.0692	0.0335	0.0681	4th
14460	14460	32039	32039	4023297	0.1306	0.018	0.2903	0.1419	0.0854	0.0418	0	0.0209	UN
14460	14460	32089	32089	4023297	0.1695	0.0031	0.314	0.0221	0.2223	0.6241	0.0134	0.3254	2nd
14460	14460	32358	32358	4023297	0.1764	0.0055	0.3543	0.0445	0.2396	0.294	0.0041	0.151	3rd
14460	14460	32785	32785	4023297	0.137	0.0179	0.3047	0.1422	0.1031	0.0322	0	0.0161	UN
14460	14460	32794	32794	4023297	0.1223	0.0168	0.2971	0.1255	0.0291	0.1723	0.0659	0.1521	3rd
14460	14460	32797	32797	4023297	0.1273	0.0281	0.282	0.1824	0.0342	0.0287	0	0.0143	UN
14460	14460	33083	33083	4023297	0.1352	0.0142	0.2949	0.1187	0.119	0.0237	0	0.0118	UN
14435	14435	6265	6265	4023297	0.1537	0.0139	0.4184	0.1473	0.2231	0.6198	0.1996	0.5095	FS
14435	14435	6955	6955	4023297	0.1541	0.0115	0.4161	0.1237	0.2302	0.6587	0.1834	0.5128	FS
14435	14435	7267	7267	4023297	0.1254	0.017	0.3136	0.1681	0.1478	0.753	0	0.3765	2nd
14435	14435	7311	7311	4023297	0.1176	0.0215	0.3019	0.2087	0.132	0.6473	0.0027	0.3264	2nd
14435	14435	7625	7625	4023297	0.1483	0.0103	0.3963	0.1092	0.2255	0.6727	0.1493	0.4856	FS
14435	14435	8170	8170	4023297	0.1208	0.0203	0.3094	0.2014	0.1391	0.6763	0.0047	0.3428	2nd
14435	14435	8307	8307	4023297	0.1162	0.0223	0.2974	0.2164	0.1258	0.6177	0.0036	0.3125	2nd
14435	14435	8395	8395	4023297	0.1437	0.0109	0.3791	0.1133	0.2131	0.688	0.1154	0.4594	FS
14460	14460	34568	34568	4023297	0.1682	0.0026	0.3096	0.0193	0.2202	0.7165	0.0064	0.3646	2nd
14460	14460	34857	34857	4023297	0.1761	0.0014	0.3649	0.0129	0.2518	0.948	0.0029	0.477	PO
14460	14460	7091	7091	4023297	0.1187	0.0237	0.2372	0.2191	0.0662	0.5382	0	0.2691	2nd
14642	14642	8170	8170	4023297	0.1047	0.0136	0.2927	0.131	0.1034	0.05	0	0.025	UN
14652	14652	6265	6265	4023297	0.1122	0.0246	0.2927	0.2259	0.0757	0.6484	0	0.3242	2nd
14652	14652	6955	6955	4023297	0.1086	0.0342	0.2783	0.3058	0.0194	0.5057	0	0.2529	2nd
14652	14652	7267	7267	4023297	0.1113	0.0275	0.2863	0.2503	0.055	0.6187	0	0.3094	2nd
14652	14652	7311	7311	4023297	0.1067	0.0263	0.2846	0.2399	0.0708	0.5862	0.0016	0.2946	2nd
14652	14652	7625	7625	4023297	0.1123	0.0216	0.2919	0.1995	0.0877	0.7025	0	0.3512	2nd
14652	14652	8170	8170	4023297	0.1085	0.0257	0.2874	0.2383	0.0727	0.6135	0.0032	0.3099	2nd
14652	14652	8307	8307	4023297	0.1087	0.0282	0.2917	0.2571	0.067	0.5674	0.0088	0.2925	2nd
14652	14652	8395	8395	4023297	0.1142	0.02	0.2982	0.1863	0.0991	0.7224	0	0.3612	2nd
14690	14690	33908	33908	4023297	0.1037	0.035	0.2613	0.3101	0.0149	0.1963	0.0318	0.13	3rd
14753	14753	7937	7937	4023297	0.0943	0.0159	0.2663	0.1376	0.08	0.1138	0	0.0569	4th
14756	14756	6265	6265	4023297	0.1159	0.0215	0.3179	0.1877	0.0868	0.6847	0.0702	0.4125	2nd
14756	14756	6955	6955	4023297	0.1234	0.0229	0.3425	0.2025	0.0944	0.6226	0.1253	0.4366	FS
14756	14756	7267	7267	4023297	0.1063	0.022	0.281	0.1884	0.0554	0.7422	0	0.3711	2nd
14756	14756	7311	7311	4023297	0.1034	0.0274	0.2846	0.2319	0.0437	0.6972	0.0046	0.3533	2nd
14756	14756	7625	7625	4023297	0.1233	0.0196	0.344	0.1746	0.1129	0.6618	0.1127	0.4436	FS
14756	14756	8307	8307	4023297	0.0994	0.0287	0.271	0.2411	0.0282	0.6123	0	0.3062	2nd
14756	14756	8395	8395	4023297	0.1428	0	0.4206	0.0004	0.2569	0.7426	0.2534	0.6247	PO
14865	14865	8170	8170	4023297	0.0975	0.0107	0.2909	0.0967	0.0734	0.2883	0.0014	0.1455	3rd
14379	14379	8780	8780	4023297	0.111	0.0106	0.3134	0.095	0.1258	0.4025	0	0.2013	2nd
14435	14435	9045	9045	4023297	0.1132	0.0262	0.2885	0.2502	0.105	0.5383	0	0.2691	2nd
14435	14435	9128	9128	4023297	0.1554	0	0.4237	0.0005	0.2824	0.8287	0.1676	0.582	PO
14460	14460	8653	8653	4023297	0.1115	0.0001	0.2499	0.0006	0.1071	0.9119	0.0851	0.5411	PO
14473	14473	9562	9562	4023297	0.1243	0.0173	0.3496	0.1698	0.1363	0.2499	0.0053	0.1302	3rd
14652	14652	9045	9045	4023297	0.1097	0.0242	0.2961	0.2204	0.0894	0.6848	0	0.3424	2nd
14652	14652	9128	9128	4023297	0.1109	0.0157	0.2877	0.1476	0.1124	0.7308	0	0.3654	2nd
14668	14668	9562	9562	4023297	0.1264	0.0147	0.3492	0.1452	0.1581	0.3068	0.0258	0.1792	2nd
14690	14690	8780	8780	4023297	0.1366	0.0014	0.3784	0.0146	0.2426	0.7204	0.1001	0.4603	FS
14756	14756	9045	9045	4023297	0.1004	0.0209	0.2754	0.1783	0.0705	0.6987	0	0.3493	2nd
14756	14756	9128	9128	4023297	0.1278	0.0168	0.3614	0.1527	0.1382	0.6493	0.17	0.4946	FS
14795	14795	8653	8653	4023297	0.0971	0.0278	0.3132	0.2234	0.082	0.2606	0.0655	0.1958	2nd
14833	14833	8780	8780	4023297	0.1044	0.0074	0.3138	0.0672	0.1035	0.3974	0	0.1987	2nd
14850	14850	9841	9841	4023297	0.0995	0.0292	0.2685	0.2642	0.0326	0.1933	0.0012	0.0979	3rd
14908	14908	9656	9656	4023297	0.1535	0.0199	0.2957	0.0743	0.0594	0.3662	0	0.1831	2nd
14916	14916	8780	8780	4023297	0.11	0.006	0.3376	0.0544	0.1286	0.4795	0	0.2398	2nd

FID1	ID1	FID2	ID2	N_SNP	HetHet	IBS0	HetConc	HomIBS0	Kinship	IBD1Seg	IBD2Seg	PropIBD	InfType
14925	14925	15190	15190	4023297	0.1162	0.0235	0.322	0.2096	0.1352	0.5604	0.0788	0.359	2nd
14925	14925	15267	15267	4023297	0.0934	0.0334	0.2838	0.2625	0.0146	0.2001	0.0327	0.1327	3rd
14930	14930	15211	15211	4023297	0.1074	0.0252	0.2987	0.2264	0.1008	0.2118	0.0209	0.1268	3rd
14944	14944	15113	15113	4023297	0.165	0.05	0.3015	0.3471	0.0583	0	0	0	UN
14959	14959	15156	15156	4023297	0.126	0.016	0.3425	0.16	0.1718	0.6711	0.0658	0.4014	2nd
15009	15009	15150	15150	4023297	0.0987	0.0232	0.2849	0.1871	0.0686	0.6031	0.0846	0.3862	FS
15122	15122	15197	15197	4023297	0.1641	0.0495	0.3117	0.2964	0.071	0.4971	0.0865	0.3351	2nd
15190	15190	15267	15267	4023297	0.1001	0.0229	0.2987	0.1908	0.0705	0.2713	0.0214	0.157	3rd
14930	14930	15444	15444	4023297	0.109	0.0232	0.297	0.2081	0.1186	0.5971	0.0367	0.3352	2nd
14930	14930	15458	15458	4023297	0.1207	0.0156	0.3161	0.154	0.1711	0.6532	0.0253	0.3519	2nd
14930	14930	15475	15475	4023297	0.0986	0.0286	0.2756	0.2495	0.0562	0.0577	0	0.0289	UN
14944	14944	15599	15599	4023297	0.1649	0.0445	0.337	0.2896	0.1133	0.0015	0.003	0.0037	UN
14959	14959	15419	15419	4023297	0.093	0.0337	0.2795	0.2745	0.0141	0.1904	0.0457	0.1409	3rd
14959	14959	15467	15467	4023297	0.1115	0.0262	0.321	0.231	0.1233	0.5692	0.0947	0.3793	FS
14959	14959	15562	15562	4023297	0.1023	0.0227	0.2519	0.1908	0.0766	0.2815	0.0585	0.1992	2nd
15009	15009	15509	15509	4023297	0.1142	0.0228	0.3097	0.2116	0.1324	0.451	0.0796	0.3051	2nd
15107	15107	15421	15421	4023297	0.1052	0.0249	0.2994	0.211	0.1209	0.5246	0.0583	0.3206	2nd
15113	15113	15566	15566	4023297	0.1598	0.0613	0.2818	0.3059	0.0258	0.002	0	0.001	UN
15113	15113	15599	15599	4023297	0.1646	0.0501	0.3033	0.3432	0.0545	0.003	0	0.0015	UN
15156	15156	15419	15419	4023297	0.1012	0.031	0.2872	0.2723	0.0134	0.2085	0.0406	0.1449	3rd
15156	15156	15467	15467	4023297	0.1197	0.0245	0.3258	0.2343	0.1177	0.5865	0.0507	0.344	2nd
15156	15156	15562	15562	4023297	0.1138	0.0231	0.2694	0.2088	0.1157	0.2859	0.0342	0.1771	2nd
15211	15211	15444	15444	4023297	0.1036	0.0311	0.2984	0.2667	0.084	0.2252	0.0348	0.1474	3rd
15211	15211	15458	15458	4023297	0.1154	0.0197	0.3185	0.1875	0.1316	0.2551	0.0426	0.1701	3rd
15211	15211	15475	15475	4023297	0.0969	0.0269	0.2895	0.2311	0.0901	0.0367	0	0.0184	UN
15232	15232	15566	15566	4023297	0.1462	0.0496	0.2806	0.175	0.0563	0.1246	0.0278	0.0901	3rd
15274	15274	15467	15467	4023297	0.0897	0.0299	0.2603	0.2346	0.05	0.2168	0.0539	0.1623	3rd
14925	14925	15824	15824	4023297	0.0956	0.0396	0.2695	0.3124	0.0213	0.3166	0.0262	0.1845	2nd
14930	14930	15845	15845	4023297	0.111	0.0274	0.2986	0.2522	0.1093	0.2435	0.0262	0.1479	3rd
14951	14951	15846	15846	4023297	0.1437	0.0561	0.2667	0.2507	0.0165	0.0372	0.035	0.0536	4th
14959	14959	15944	15944	4023297	0.1232	0.0212	0.3608	0.2023	0.173	0.4555	0.1869	0.4146	FS
15009	15009	15628	15628	4023297	0.1045	0.0261	0.2941	0.2148	0.0814	0.5868	0.0611	0.3545	2nd
15009	15009	16019	16019	4023297	0.0994	0.0249	0.2848	0.2102	0.0646	0.1997	0.0371	0.137	3rd
15107	15107	15633	15633	4023297	0.0986	0.023	0.2953	0.1844	0.1	0.607	0.079	0.3825	2nd
15107	15107	16067	16067	4023297	0.1111	0.0215	0.3402	0.1863	0.1404	0.2557	0.0326	0.1604	3rd
15150	15150	15628	15628	4023297	0.0843	0.031	0.2591	0.2278	0.0374	0.4837	0.1045	0.3464	2nd
15150	15150	16019	16019	4023297	0.0992	0.0197	0.3325	0.1577	0.1477	0.2657	0.123	0.2559	2nd
15156	15156	15944	15944	4023297	0.1236	0.0194	0.335	0.1996	0.1514	0.5099	0.0835	0.3384	2nd
15190	15190	15824	15824	4023297	0.0987	0.0358	0.2708	0.2945	0.0311	0.3679	0.0142	0.1982	2nd
15211	15211	15845	15845	4023297	0.1068	0.023	0.3043	0.2094	0.1199	0.0735	0.0052	0.0419	UN
15274	15274	15944	15944	4023297	0.0882	0.028	0.2508	0.2262	0.049	0.1477	0.035	0.1088	3rd
14959	14959	16083	16083	4023297	0.0977	0.0279	0.2925	0.2381	0.0624	0.1415	0.0206	0.0913	3rd
15009	15009	16338	16338	4023297	0.102	0.0247	0.3158	0.2058	0.0489	0.2699	0.0294	0.1643	3rd
15122	15122	16243	16243	4023297	0.1774	0.024	0.3741	0.1485	0.1978	0.2483	0.1185	0.2427	2nd
15122	15122	16289	16289	4023297	0.1337	0.0468	0.2819	0.2662	0.0309	0.1313	0.014	0.0797	4th
15150	15150	16261	16261	4023297	0.0847	0.0336	0.2623	0.2544	0.028	0.2258	0.0545	0.1674	3rd
15150	15150	16338	16338	4023297	0.0765	0.0304	0.2567	0.221	0.0156	0.195	0.0486	0.1461	3rd
15156	15156	16083	16083	4023297	0.1011	0.0301	0.2819	0.2735	0.0247	0.0963	0.0106	0.0587	4th
15197	15197	16243	16243	4023297	0.162	0.0415	0.3077	0.2537	0.0917	0.261	0.0325	0.1629	3rd
15197	15197	16289	16289	4023297	0.1507	0.0419	0.3047	0.2583	0.0456	0.1972	0.0093	0.1078	3rd
14930	14930	16413	16413	4023297	0.1155	0.0249	0.3003	0.2347	0.1252	0.5712	0.0059	0.2915	2nd
14930	14930	16517	16517	4023297	0.1183	0.0187	0.3114	0.1807	0.1578	0.6461	0.0192	0.3423	2nd
14930	14930	16548	16548	4023297	0.0973	0.0306	0.282	0.257	0.0293	0.1396	0.0169	0.0867	4th
15009	15009	16524	16524	4023297	0.0976	0.0283	0.2937	0.2227	0.0225	0.276	0.0117	0.1497	3rd
15113	15113	16743	16743	4023297	0.1646	0.0401	0.291	0.1958	0.0962	0.0068	0	0.0034	UN
15122	15122	16744	16744	4023297	0.1384	0.0485	0.2847	0.3083	0.0453	0.1289	0.0154	0.0799	4th
15150	15150	16524	16524	4023297	0.0753	0.0235	0.2477	0.1706	0.0572	0.2043	0.0642	0.1663	3rd
15190	15190	16527	16527	4023297	0.1004	0.0325	0.2747	0.2791	0.053	0.2093	0.0172	0.1219	3rd
15197	15197	16744	16744	4023297	0.1509	0.0371	0.2954	0.2621	0.0733	0.162	0.0092	0.0902	3rd
15211	15211	16413	16413	4023297	0.1117	0.0295	0.3075	0.2699	0.0819	0.2408	0.0224	0.1428	3rd
15211	15211	16517	16517	4023297	0.1112	0.0229	0.3069	0.2136	0.1127	0.2848	0.0231	0.1655	3rd
15232	15232	16743	16743	4023297	0.1499	0.0416	0.2879	0.1466	0.0844	0.1598	0.0448	0.1247	3rd
14944	14944	16780	16780	4023297	0.1638	0.0413	0.3331	0.2836	0.1219	0.0275	0.0017	0.0154	UN
14951	14951	17139	17139	4023297	0.1531	0.0329	0.32	0.1871	0.0755	0.1919	0.0208	0.1168	3rd
14959	14959	16775	16775	4023297	0.1031	0.0244	0.2968	0.2149	0.1072	0.2485	0.0377	0.1619	3rd
14959	14959	17112	17112	4023297	0.0886	0.0304	0.2608	0.2496	0.0232	0.228	0.0302	0.1442	3rd
15009	15009	16827	16827	4023297	0.093	0.0275	0.2667	0.2318	0.0286	0.1363	0.0191	0.0873	4th
15113	15113	16780	16780	4023297	0.1735	0.0396	0.3242	0.2934	0.1011	0.005	0	0.0025	UN
15150	15150	16827	16827	4023297	0.0785	0.0253	0.2512	0.1907	0.0675	0.1438	0.026	0.098	3rd
15156	15156	16775	16775	4023297	0.1121	0.0234	0.306	0.2244	0.1002	0.2404	0.0207	0.1409	3rd
15156	15156	17112	17112	4023297	0.1001	0.03	0.2809	0.266	0.0186	0.2563	0.0231	0.1513	3rd
15197	15197	16787	16787	4023297	0.1535	0.0407	0.3049	0.2559	0.0626	0.1433	0.01	0.0816	4th
15197	15197	16853	16853	4023297	0.1629	0.0742	0.2904	0.3907	0.0177	0.2343	0.0037	0.1208	3rd
15232	15232	16862	16862	4023297	0.1358	0.054	0.2614	0.1793	0.0359	0.3163	0.0321	0.1902	2nd
14930	14930	17199	17199	4023297	0.1084	0.0281	0.2857	0.2564	0.1036	0.5025	0.0162	0.2675	2nd
14930	14930	17832	17832	4023297	0.104	0.0288	0.3299	0.2448	0.0294	0.281	0.0101	0.1506	3rd
14944	14944	17295	17295	4023297	0.166	0.0524	0.3043	0.3515	0.053	0	0	0	UN
14959	14959	17325	17325	4023297	0.1079	0.0206	0.3448	0.1833	0.1179	0.2781	0.0183	0.1573	3rd
15113	15113	17295	17295	4023297	0.2321	0.021	0.4358	0.1918	0.2482	0.159	0	0.0795	4th
15113	15113	17826	17826	4023297	0.1631	0.0414	0.3135	0.2454	0.0653	0.0196	0	0.0098	UN
15113	15113	17833	17833	4023297	0.1695	0.0512	0.3002	0.2577	0.073	0.0012	0	0.0006	UN
15150	15150	17233	17233	4023297	0.0827	0.0276	0.2881	0.2066	0.0441	0.2227	0.0605	0.1719	3rd
15150	15150	17871	17871	4023297	0.0812	0.0289	0.2773	0.2114	0.0377	0.2644	0.0595	0.1917	2nd
15156	15156	17325	17325	4023297	0.0989	0.0251	0.2826	0.2276	0.0323	0.1114	0.0023	0.058	4th
15211	15211	17199	17199	4023297	0.0992	0.0274	0.2726	0.2413	0.0767	0.2145	0.0048	0.1121	3rd
15232	15232	17254	17254	4023297	0.1644	0.0519	0.3376	0.1762	0.0895	0.286	0.121	0.264	2nd
15232	15232	17833	17833	4023297	0.1624	0.0425	0.3172	0.1565	0.098	0.1142	0.0847	0.1418	3rd
14930	14930	17903	17903	4023297	0.114	0.0273	0.3114	0.246	0.1142	0.5355	0.0533	0.3211	2nd
14930	14930	18385	18385	4023297	0.1041	0.0236	0.2843	0.2083	0.1027	0.6013	0.0257	0.3264	2nd
14930	14930	18599	18599	4023297	0.0954	0.0314	0.269	0.2677	0.0282	0.1166	0	0.0583	4th
14944	14944	18528	18528	4023297	0.1658	0.0506	0.3179	0.3285	0.0766	0	0	0	UN
14951	14951	17969	17969	4023297	0.1519	0.0387	0.2981	0.2486	0.0718	0.0026	0	0.0013	UN
14951	14951	17970	17970	4023297	0.1584	0.0435	0.2973	0.2892	0.0833	0.0141	0	0.007	UN
14951	14951	18019	18019	4023297	0.1754	0.0351	0.3396	0.21	0.135	0.1396	0.0196	0.0894	3rd
15009	15009	17998	17998	4023297	0.0993	0.0412	0.2565	0.3693	0.0255	0.0415	0	0.0207	UN
15107	15107	17981	17981	4023297	0.0927	0.0341	0.2726	0.2723	0.0315	0.161	0.0239	0.1044	3rd
15107	15107	18022	18022	4023297	0.0937	0.0248	0.2969	0.2011	0.0573	0.2326	0.0334	0.1497	3rd
15113	15113	18528	18528	4023297	0.2076	0.0267	0.3896	0.2261	0.1981	0.067	0	0.0335	UN

FID1	ID1	FID2	ID2	N_SNP	HetHet	IBS0	HetConc	HomIBS0	Kinship	IBD1Seg	IBD2Seg	PropIBD	InfType
15211	15211	17903	17903	4023297	0.1127	0.0234	0.3289	0.2116	0.1343	0.2925	0.0683	0.2145	2nd
15211	15211	18385	18385	4023297	0.1012	0.023	0.2941	0.198	0.1212	0.3188	0.0218	0.1812	2nd
15211	15211	18599	18599	4023297	0.0895	0.0341	0.2667	0.2825	0.0314	0.031	0.0025	0.018	UN
14930	14930	19181	19181	4023297	0.117	0.0213	0.3083	0.2022	0.1468	0.6082	0.0318	0.3359	2nd
14930	14930	19348	19348	4023297	0.1138	0.0218	0.3046	0.2008	0.1407	0.7003	0.0015	0.3516	2nd
14959	14959	19674	19674	4023297	0.0963	0.0351	0.2853	0.2826	0.0256	0.2794	0.0385	0.1783	2nd
15113	15113	18866	18866	4023297	0.1501	0.0552	0.2568	0.4424	0.0344	0	0	0	UN
15113	15113	18929	18929	4023297	0.1458	0.0528	0.2517	0.4394	0.0295	0	0	0	UN
15156	15156	19674	19674	4023297	0.0987	0.0299	0.2716	0.2616	0.0225	0.2492	0.0168	0.1414	3rd
15157	15157	18866	18866	4023297	0.1407	0.0327	0.316	0.2461	0.0355	0.0394	0	0.0197	UN
15190	15190	18938	18938	4023297	0.1075	0.0345	0.274	0.2883	0.0684	0.1788	0.008	0.0973	3rd
15190	15190	19371	19371	4023297	0.098	0.0327	0.2816	0.2752	0.0262	0.1279	0.005	0.0689	4th
15211	15211	19181	19181	4023297	0.1086	0.0238	0.2993	0.2186	0.1049	0.3012	0.0105	0.1611	3rd
15211	15211	19348	19348	4023297	0.1034	0.0303	0.2878	0.2662	0.0736	0.2217	0.0169	0.1278	3rd
15232	15232	18778	18778	4023297	0.1539	0.0447	0.3294	0.1536	0.0876	0.1965	0.0112	0.1095	3rd
14925	14925	1X2231	1X2231	4023297	0.1344	0	0.3775	0.0005	0.2612	0.8821	0.113	0.5541	PO
14925	14925	1X4777	1X4777	4023297	0.1068	0.0401	0.2727	0.3533	0.0206	0.279	0.0043	0.1438	3rd
14930	14930	1X2124	1X2124	4023297	0.1237	0.0297	0.3027	0.2847	0.0897	0.4665	0	0.2333	2nd
14930	14930	25354	25354	4023297	0.1069	0.0289	0.2841	0.2598	0.0942	0.3406	0.003	0.1733	3rd
14948	14948	1X2231	1X2231	4023297	0.1027	0.0159	0.26	0.1228	0.1287	0.4144	0.0235	0.2308	2nd
14951	14951	1X3796	1X3796	4023297	0.1883	0.0567	0.2907	0.3695	0.0265	0.0164	0	0.0082	UN
14951	14951	1X3837	1X3837	4023297	0.2155	0.0402	0.3437	0.2703	0.1051	0.2793	0	0.1396	3rd
14951	14951	1X4209	1X4209	4023297	0.1914	0.0528	0.2941	0.3523	0.0372	0.0145	0	0.0072	UN
15009	15009	1X3697	1X3697	4023297	0.1544	0.0001	0.4233	0.0008	0.2874	0.8158	0.1786	0.5865	PO
15009	15009	1X3822	1X3822	4023297	0.1206	0.0221	0.2989	0.2167	0.1251	0.5684	0	0.2842	2nd
15107	15107	1X1155	1X1155	4023297	0.0971	0.0384	0.2619	0.3173	0.0318	0.3587	0.0025	0.1819	2nd
15112	15112	1X4209	1X4209	4023297	0.1818	0.0178	0.3207	0.1371	0.0754	0.0144	0	0.0072	UN
15113	15113	1X3796	1X3796	4023297	0.2025	0.0441	0.3097	0.3658	0.09	0	0	0	UN
15113	15113	1X3837	1X3837	4023297	0.2003	0.0452	0.3022	0.3814	0.0798	0.0013	0	0.0007	UN
15113	15113	1X4209	1X4209	4023297	0.2029	0.0439	0.3075	0.3747	0.0868	0	0	0	UN
15122	15122	1X4209	1X4209	4023297	0.1934	0.0386	0.3155	0.2586	0.0605	0.3405	0.0012	0.1714	3rd
15149	15149	1X4179	1X4179	4023297	0.1195	0.0086	0.2573	0.082	0.1336	0.1362	0	0.0681	4th
15150	15150	1X3697	1X3697	4023297	0.1151	0.0001	0.3255	0.0006	0.1974	0.9497	0.0463	0.5211	PO
15150	15150	1X3822	1X3822	4023297	0.1031	0.0255	0.2784	0.214	0.0318	0.6426	0	0.3213	2nd
15156	15156	1X3656	1X3656	4023297	0.107	0.0454	0.2604	0.4526	0.028	0.0131	0	0.0065	UN
15156	15156	1X3697	1X3697	4023297	0.1112	0.0443	0.264	0.4589	0.0332	0.0128	0	0.0064	UN
15156	15156	1X3822	1X3822	4023297	0.112	0.0454	0.2633	0.4594	0.0259	0.0268	0	0.0134	UN
15156	15156	1X4080	1X4080	4023297	0.1079	0.0473	0.2585	0.4584	0.0222	0.0102	0	0.0051	UN
15156	15156	1X4179	1X4179	4023297	0.1074	0.0455	0.2605	0.45	0.0299	0.0061	0	0.003	UN
15156	15156	1X4777	1X4777	4023297	0.1098	0.0468	0.2628	0.4638	0.0257	0.0131	0	0.0065	UN
15175	15175	1X2231	1X2231	4023297	0.0934	0.0262	0.2332	0.2076	0.0632	0.0346	0	0.0173	UN
15178	15178	1X2231	1X2231	4023297	0.0887	0.0141	0.2723	0.1169	0.0304	0.2152	0.0082	0.1158	3rd
15190	15190	1X2231	1X2231	4023297	0.1436	0	0.3995	0.0004	0.2795	0.8562	0.1424	0.5705	PO
15190	15190	1X4777	1X4777	4023297	0.1178	0.0262	0.2994	0.2508	0.1115	0.4758	0.0021	0.24	2nd
15197	15197	1X3796	1X3796	4023297	0.1904	0.0521	0.2943	0.3683	0.0431	0.0137	0	0.0069	UN
15197	15197	1X3837	1X3837	4023297	0.1939	0.0523	0.2981	0.3746	0.0426	0.0128	0	0.0064	UN
15197	15197	1X4209	1X4209	4023297	0.2158	0.0353	0.3437	0.2602	0.1199	0.3423	0.0012	0.1724	3rd
15211	15211	1X2124	1X2124	4023297	0.1146	0.0291	0.2917	0.2683	0.0539	0.1583	0	0.0792	4th
15211	15211	25354	25354	4023297	0.1053	0.0247	0.2983	0.2217	0.1081	0.1328	0.0146	0.081	4th
15217	15217	1X2816	1X2816	4023297	0.1098	0.0103	0.2998	0.0901	0.1478	0.4727	0.0042	0.2405	2nd
15232	15232	25355	25355	4023297	0.1633	0.041	0.3465	0.144	0.1212	0.1634	0.0598	0.1415	3rd
15267	15267	1X2231	1X2231	4023297	0.1189	0.0049	0.3608	0.0456	0.1973	0.4572	0.0276	0.2562	2nd
14925	14925	26498	26498	4023297	0.1576	0.0056	0.303	0.064	0.0856	0.3297	0	0.1649	3rd
14925	14925	26800	26800	4023297	0.114	0.0071	0.35	0.0649	0.2104	0.311	0.0692	0.2247	2nd
14925	14925	26980	26980	4023297	0.1095	0.0069	0.3256	0.0571	0.2026	0.4042	0.0704	0.2725	2nd
14925	14925	27351	27351	4023297	0.1129	0.0275	0.2778	0.2294	0.0654	0.5431	0.0758	0.3473	2nd
14930	14930	26988	26988	4023297	0.1087	0.0291	0.2841	0.2678	0.1019	0.4599	0.012	0.242	2nd
14930	14930	27503	27503	4023297	0.1153	0.025	0.3077	0.2335	0.1315	0.5052	0.0497	0.3023	2nd
14951	14951	26498	26498	4023297	0.1721	0.0513	0.2706	0.4112	0.0384	0	0	0	UN
15107	15107	27166	27166	4023297	0.114	0.0042	0.4143	0.0364	0.2239	0.7074	0.0059	0.3596	2nd
15113	15113	26498	26498	4023297	0.1863	0.0331	0.2902	0.3716	0.1159	0	0	0	UN
15113	15113	27033	27033	4023297	0.16	0.0532	0.2998	0.3495	0.0286	0	0	0	UN
15122	15122	26498	26498	4023297	0.1765	0.05	0.2964	0.3966	0.026	0.0051	0	0.0026	UN
15149	15149	26498	26498	4023297	0.1656	0.0342	0.2737	0.3705	0.0569	0	0	0	UN
15150	15150	27306	27306	4023297	0.1011	0.0047	0.3687	0.0382	0.2303	0.3537	0.1681	0.345	FS
15190	15190	26498	26498	4023297	0.1548	0.0176	0.2889	0.2093	0.0393	0.1755	0	0.0877	4th
15190	15190	26800	26800	4023297	0.1065	0.0287	0.3078	0.2519	0.073	0.2061	0.0477	0.1507	3rd
15190	15190	27351	27351	4023297	0.1118	0.0292	0.2659	0.2492	0.066	0.5371	0.0295	0.298	2nd
15197	15197	26355	26355	4023297	0.1333	0.03	0.2638	0.1964	0.0524	0.4015	0.0366	0.2374	2nd
15197	15197	26498	26498	4023297	0.189	0.0454	0.3046	0.4076	0.0787	0.0044	0	0.0022	UN
15197	15197	27193	27193	4023297	0.1438	0.0494	0.2714	0.2813	0.0288	0.2563	0.0242	0.1524	3rd
15211	15211	26988	26988	4023297	0.1046	0.0284	0.2893	0.2541	0.0808	0.2589	0.0204	0.1499	3rd
15211	15211	27503	27503	4023297	0.1089	0.0231	0.3057	0.2127	0.1158	0.1951	0.0483	0.1459	3rd
14925	14925	28212	28212	4023297	0.0937	0.0089	0.3039	0.073	0.1313	0.3381	0.0374	0.2065	2nd
14925	14925	28279	28279	4023297	0.1154	0.0283	0.271	0.2458	0.0438	0.4832	0.0547	0.2963	2nd
14930	14930	28246	28246	4023297	0.1189	0.027	0.3096	0.2648	0.1206	0.5424	0.0116	0.2828	2nd
15009	15009	27989	27989	4023297	0.1057	0.0251	0.3012	0.2187	0.0867	0.2763	0.087	0.2251	2nd
15009	15009	28183	28183	4023297	0.115	0.0181	0.3365	0.1584	0.142	0.5072	0.1545	0.4081	FS
15107	15107	27844	27844	4023297	0.0974	0.01	0.3315	0.0837	0.1378	0.3889	0.0344	0.2289	2nd
15107	15107	27874	27874	4023297	0.0869	0.0121	0.3043	0.097	0.0726	0.3623	0.0575	0.2387	2nd
15107	15107	27889	27889	4023297	0.1086	0.0057	0.3591	0.0501	0.204	0.4428	0.0777	0.299	2nd
15112	15112	28259	28259	4023297	0.1168	0.0388	0.2775	0.251	0.0724	0.0012	0	0.0006	UN
15122	15122	27985	27985	4023297	0.1277	0.0527	0.2553	0.2715	0.0163	0.1903	0.0178	0.113	3rd
15150	15150	27989	27989	4023297	0.0987	0.0036	0.3206	0.0297	0.2168	0.4511	0.1388	0.3644	FS
15150	15150	28183	28183	4023297	0.1074	0.0018	0.3593	0.0153	0.2481	0.6538	0.2146	0.5415	FS
15190	15190	28279	28279	4023297	0.116	0.0284	0.265	0.2536	0.0555	0.4653	0.0211	0.2537	2nd
15197	15197	27985	27985	4023297	0.1584	0.0402	0.3126	0.2316	0.0778	0.3191	0.0846	0.2441	2nd
15211	15211	28246	28246	4023297	0.115	0.0169	0.3168	0.1665	0.1429	0.4912	0.019	0.2646	2nd
14925	14925	28400	28400	4023297	0.1041	0.0084	0.3356	0.0711	0.1711	0.3662	0.0247	0.2078	2nd
14925	14925	28416	28416	4023297	0.1028	0.0067	0.3121	0.058	0.1832	0.2682	0.0401	0.1742	3rd
15009	15009	28635	28635	4023297	0.1036	0.0362	0.2575	0.2923	0.0527	0.4532	0.0596	0.2862	2nd
15150	15150	28417	28417	4023297	0.0933	0.008	0.3345	0.064	0.1889	0.3273	0.1156	0.2792	2nd
15190	15190	28416	28416	4023297	0.0962	0.0335	0.2759	0.2753	0.0171	0.1497	0.0131	0.0879	4th
15009	15009	30917	30917	4023297	0.1297	0.0303	0.271	0.3337	0.0257	0	0	0	UN
15113	15113	30609	30609	4023297	0.1772	0.0136	0.3288	0.1168	0.1879	0.0101	0	0.0051	UN
15113	15113	30917	30917	4023297	0.1535	0.0416	0.2603	0.4421	0.0821	0	0	0	UN
15113	15113	30920	30920	4023297	0.1817	0.0388	0.2957	0.3943	0.1161	0	0	0	UN
15149	15149	30917	30917	4023297	0.1359	0.0433	0.2472	0.4451	0.0484	0	0	0	UN

FID1	ID1	FID2	ID2	N_SNP	HetHet	IBS0	HetConc	HomIBS0	Kinship	IBD1Seg	IBD2Seg	PropIBD	InfType
15149	15149	30920	30920	4023297	0.1551	0.0425	0.266	0.4165	0.04	0	0	0	UN
15197	15197	30917	30917	4023297	0.145	0.0592	0.2502	0.5066	0.0347	0	0	0	UN
15197	15197	30920	30920	4023297	0.1899	0.0313	0.3234	0.2673	0.1409	0	0	0	UN
14925	14925	31134	31134	4023297	0.0989	0.0376	0.2626	0.3177	0.0393	0.0903	0	0.0452	4th
14930	14930	30961	30961	4023297	0.117	0.0049	0.3171	0.0492	0.2171	0.3015	0	0.1507	3rd
14930	14930	30973	30973	4023297	0.0958	0.0142	0.2809	0.1254	0.1042	0.0786	0	0.0393	UN
14944	14944	31065	31065	4023297	0.1531	0.0576	0.2845	0.391	0.0331	0	0	0	UN
14951	14951	30933	30933	4023297	0.1623	0.0517	0.2905	0.341	0.0796	0.0125	0	0.0062	UN
14951	14951	31011	31011	4023297	0.1531	0.0652	0.2733	0.4371	0.0242	0.0189	0	0.0094	UN
15009	15009	31016	31016	4023297	0.1047	0.0337	0.2746	0.3003	0.0679	0.0937	0	0.0468	4th
15009	15009	31145	31145	4023297	0.1148	0.0353	0.2604	0.33	0.0285	0.3613	0	0.1807	2nd
15107	15107	31234	31234	4023297	0.0996	0.031	0.2516	0.2646	0.0394	0.0594	0	0.0297	UN
15113	15113	30933	30933	4023297	0.1563	0.0547	0.2671	0.4449	0.0486	0	0	0	UN
15113	15113	31011	31011	4023297	0.1509	0.0542	0.259	0.4618	0.0378	0	0	0	UN
15113	15113	31028	31028	4023297	0.1708	0.0165	0.3169	0.1261	0.1682	0.0029	0	0.0015	UN
15113	15113	31065	31065	4023297	0.1782	0.0426	0.3147	0.3484	0.1141	0.0013	0	0.0006	UN
15149	15149	31145	31145	4023297	0.1218	0.0458	0.2382	0.4037	0.0353	0.017	0.0015	0.01	UN
15156	15156	31124	31124	4023297	0.1105	0.0099	0.2893	0.099	0.1645	0.0819	0	0.0409	UN
15190	15190	31021	31021	4023297	0.1146	0.03	0.2566	0.2771	0.0386	0.3987	0.0013	0.2006	2nd
15190	15190	31134	31134	4023297	0.1171	0.0069	0.3158	0.0656	0.2108	0.1965	0.0022	0.1004	3rd
15267	15267	31021	31021	4023297	0.0985	0.0085	0.2415	0.0733	0.0489	0.1624	0	0.0812	4th
14930	14930	31401	31401	4023297	0.0873	0.0239	0.2359	0.189	0.0527	0.0264	0	0.0132	UN
14951	14951	31337	31337	4023297	0.153	0.0641	0.2764	0.4272	0.0231	0.012	0	0.006	UN
14959	14959	31297	31297	4023297	0.107	0.0336	0.264	0.3074	0.0354	0.4004	0	0.2002	2nd
14959	14959	31299	31299	4023297	0.1009	0.0391	0.2539	0.3471	0.0138	0.1797	0	0.0899	3rd
14959	14959	31327	31327	4023297	0.0928	0.0349	0.2569	0.3066	0.0389	0.0782	0	0.0391	UN
15009	15009	31415	31415	4023297	0.0936	0.0231	0.2495	0.1861	0.0767	0.003	0	0.0015	UN
15113	15113	31337	31337	4023297	0.1456	0.0547	0.2504	0.4606	0.0246	0	0	0	UN
15156	15156	31297	31297	4023297	0.1189	0.0301	0.282	0.3095	0.0944	0.3465	0	0.1733	3rd
15156	15156	31299	31299	4023297	0.1136	0.0321	0.2753	0.3204	0.0905	0.1697	0	0.0849	4th
15156	15156	31311	31311	4023297	0.1131	0.0118	0.265	0.1128	0.1544	0.0837	0	0.0419	UN
15156	15156	31327	31327	4023297	0.102	0.0337	0.2682	0.3256	0.0335	0.0549	0	0.0274	UN
15156	15156	31333	31333	4023297	0.1025	0.0137	0.2887	0.1306	0.1094	0.0349	0	0.0174	UN
15156	15156	31364	31364	4023297	0.1125	0.0367	0.2436	0.3489	0.0247	0.0621	0	0.0311	UN
15190	15190	31284	31284	4023297	0.1185	0.0051	0.2817	0.0494	0.1707	0.305	0	0.1525	3rd
15190	15190	31331	31331	4023297	0.1208	0.0048	0.2676	0.0463	0.1426	0.3505	0.0017	0.1769	2nd
15197	15197	31409	31409	4023297	0.1328	0.0197	0.2756	0.1399	0.0734	0.0308	0	0.0154	UN
15197	15197	31410	31410	4023297	0.1745	0.0084	0.3289	0.0596	0.2145	0.2331	0.0018	0.1184	3rd
15197	15197	31462	31462	4023297	0.1462	0.0117	0.2939	0.0883	0.1444	0.0884	0	0.0442	UN
14951	14951	31497	31497	4023297	0.1534	0.0562	0.2742	0.3711	0.05	0.0142	0	0.0071	UN
14959	14959	31500	31500	4023297	0.1004	0.0333	0.2685	0.3033	0.0634	0.1588	0	0.0794	4th
15009	15009	31499	31499	4023297	0.0913	0.0191	0.2662	0.1614	0.0605	0.0223	0	0.0112	UN
15009	15009	31602	31602	4023297	0.0872	0.0295	0.2214	0.2209	0.0447	0.0088	0	0.0044	UN
15009	15009	31630	31630	4023297	0.0907	0.0188	0.2677	0.1566	0.0551	0.0341	0	0.017	UN
15107	15107	31556	31556	4023297	0.0892	0.0181	0.263	0.1489	0.0977	0.0291	0	0.0146	UN
15113	15113	31497	31497	4023297	0.1504	0.0561	0.258	0.4581	0.0315	0	0	0	UN
15113	15113	31560	31560	4023297	0.1648	0.0152	0.3101	0.1274	0.1591	0.0013	0	0.0007	UN
15156	15156	31500	31500	4023297	0.1242	0.0043	0.3286	0.0472	0.2191	0.2972	0	0.1486	3rd
15156	15156	31553	31553	4023297	0.1075	0.011	0.3002	0.1079	0.1405	0.0738	0	0.0369	UN
15156	15156	31578	31578	4023297	0.1082	0.0103	0.304	0.0998	0.1448	0.1012	0.0021	0.0527	4th
15156	15156	31624	31624	4023297	0.1155	0.0079	0.2926	0.0809	0.1886	0.1272	0	0.0636	4th
15156	15156	31650	31650	4023297	0.0922	0.0172	0.2674	0.1544	0.0441	0.0233	0.0015	0.0131	UN
15190	15190	31498	31498	4023297	0.1131	0.0284	0.2469	0.2629	0.0319	0.2785	0	0.1392	3rd
15190	15190	31525	31525	4023297	0.1008	0.0207	0.2199	0.1683	0.0503	0.0175	0	0.0088	UN
15244	15244	31624	31624	4023297	0.0903	0.0258	0.2546	0.1925	0.0302	0.0046	0	0.0023	UN
15267	15267	31498	31498	4023297	0.0978	0.0103	0.2336	0.0884	0.0244	0.1169	0	0.0585	4th
14951	14951	31903	31903	4023297	0.1541	0.0621	0.267	0.3543	0.0366	0.0102	0	0.0051	UN
15107	15107	31966	31966	4023297	0.0971	0.0305	0.2419	0.2573	0.0327	0.0595	0	0.0298	UN
15113	15113	31903	31903	4023297	0.1834	0.0426	0.3228	0.2904	0.1236	0	0	0	UN
15156	15156	31764	31764	4023297	0.1072	0.0412	0.253	0.3793	0.039	0.076	0.0013	0.0393	UN
15156	15156	31805	31805	4023297	0.1301	0.0036	0.2843	0.0378	0.1723	0.4491	0	0.2246	2nd
15156	15156	31902	31902	4023297	0.1068	0.0122	0.2971	0.1184	0.1328	0.0532	0	0.0266	UN
15156	15156	31952	31952	4023297	0.1048	0.0381	0.26	0.3637	0.0439	0.0477	0	0.0239	UN
15156	15156	31958	31958	4023297	0.1009	0.0152	0.2717	0.1362	0.1082	0.1231	0	0.0615	4th
15190	15190	31786	31786	4023297	0.117	0.0062	0.2854	0.0598	0.1755	0.2141	0	0.1071	3rd
15190	15190	31849	31849	4023297	0.1086	0.006	0.3436	0.0525	0.178	0.4012	0.068	0.2686	2nd
15190	15190	31970	31970	4023297	0.1092	0.0112	0.2566	0.1011	0.1313	0.0651	0	0.0326	UN
15197	15197	31716	31716	4023297	0.1754	0.0119	0.3534	0.0892	0.2007	0.0883	0	0.0441	UN
15197	15197	31778	31778	4023297	0.137	0.0203	0.2845	0.1422	0.0823	0.0357	0	0.0179	UN
15197	15197	31846	31846	4023297	0.1521	0.0139	0.2945	0.096	0.1553	0.1003	0	0.0502	4th
15197	15197	31880	31880	4023297	0.1473	0.0189	0.3049	0.1343	0.1143	0.0487	0	0.0243	UN
15197	15197	31931	31931	4023297	0.1257	0.021	0.2791	0.1103	0.0191	0.3881	0.0164	0.2105	2nd
15197	15197	31938	31938	4023297	0.1313	0.0197	0.2728	0.1391	0.0688	0.0324	0	0.0162	UN
14930	14930	32043	32043	4023297	0.1128	0.0348	0.2681	0.3275	0.0455	0.3224	0	0.1612	3rd
14959	14959	32025	32025	4023297	0.1085	0.0319	0.2754	0.2944	0.0566	0.408	0	0.204	2nd
14959	14959	32298	32298	4023297	0.0923	0.0165	0.2711	0.1413	0.1073	0.0351	0	0.0175	UN
15122	15122	32224	32224	4023297	0.1354	0.0587	0.2582	0.3943	0.0225	0.2299	0	0.1149	3rd
15156	15156	31993	31993	4023297	0.1096	0.0101	0.3034	0.1018	0.1519	0.0792	0	0.0396	UN
15156	15156	31997	31997	4023297	0.1021	0.0129	0.2887	0.1225	0.1109	0.0462	0	0.0231	UN
15156	15156	32025	32025	4023297	0.117	0.0293	0.2828	0.3022	0.1033	0.3247	0.0016	0.1639	3rd
15156	15156	32040	32040	4023297	0.1165	0.0321	0.2692	0.3225	0.0738	0.3211	0	0.1606	3rd
15156	15156	32132	32132	4023297	0.1022	0.0149	0.2881	0.1402	0.1019	0.026	0	0.013	UN
15156	15156	32227	32227	4023297	0.099	0.0142	0.2874	0.1312	0.0865	0.101	0	0.0505	4th
15156	15156	32363	32363	4023297	0.1	0.0144	0.2854	0.1354	0.0932	0.0485	0.0015	0.0258	UN
15190	15190	32025	32025	4023297	0.1072	0.0412	0.263	0.3768	0.0252	0.1931	0	0.0965	3rd
15197	15197	32224	32224	4023297	0.1832	0.003	0.3564	0.0238	0.2429	0.7635	0.0013	0.383	2nd
15197	15197	32451	32451	4023297	0.1324	0.0258	0.2814	0.1724	0.0386	0.0133	0	0.0066	UN
15197	15197	32472	32472	4023297	0.1314	0.0254	0.2792	0.1702	0.0374	0.0205	0	0.0102	UN
15211	15211	32132	32132	4023297	0.0887	0.0196	0.2704	0.1684	0.0941	0.0012	0	0.0006	UN
14930	14930	32849	32849	4023297	0.1134	0.0366	0.2565	0.3409	0.0172	0.3282	0	0.1641	3rd
15009	15009	32606	32606	4023297	0.0862	0.0288	0.2241	0.2148	0.0363	0.0043	0	0.0021	UN
15156	15156	32553	32553	4023297	0.1008	0.0247	0.2244	0.2034	0.0716	0.0095	0	0.0048	UN
15156	15156	32560	32560	4023297	0.0967	0.0173	0.2782	0.1593	0.0635	0.0253	0	0.0126	UN
15156	15156	32588	32588	4023297	0.1061	0.0129	0.2815	0.1228	0.1373	0.0517	0	0.0259	UN
15156	15156	32651	32651	4023297	0.1013	0.0145	0.2879	0.1384	0.0987	0.0405	0	0.0203	UN
15156	15156	32736	32736	4023297	0.0997	0.0211	0.2389	0.1787	0.1079	0.0472	0	0.0236	UN
15156	15156	32772	32772	4023297	0.1172	0.0074	0.3196	0.0761	0.1871	0.1417	0.0015	0.0724	4th
15156	15156	32773	32773	4023297	0.1043	0.0152	0.2751	0.1415	0.1227	0.0425	0	0.0212	UN

FID1	ID1	FID2	ID2	N_SNP	HetHet	IBS0	HetConc	HomIBS0	Kinship	IBD1Seg	IBD2Seg	PropIBD	InfType
15156	15156	33115	33115	4023297	0.1109	0.0111	0.3072	0.1102	0.1512	0.0526	0	0.0263	UN
15197	15197	32591	32591	4023297	0.1389	0.0223	0.2925	0.1541	0.0748	0.0204	0	0.0102	UN
15197	15197	32801	32801	4023297	0.1249	0.022	0.2618	0.1531	0.0376	0.0159	0	0.0079	UN
15197	15197	32863	32863	4023297	0.1364	0.0229	0.2703	0.1389	0.0851	0.0801	0	0.0401	UN
15197	15197	32996	32996	4023297	0.1494	0.015	0.3214	0.0876	0.1249	0.3727	0.0198	0.2061	2nd
15197	15197	33097	33097	4023297	0.1276	0.0247	0.27	0.1679	0.031	0.0285	0	0.0142	UN
14930	14930	33863	33863	4023297	0.1162	0.0344	0.279	0.323	0.0549	0.3949	0	0.1975	2nd
14951	14951	7158	7158	4023297	0.1611	0.0714	0.2909	0.3259	0.0194	0.3333	0.004	0.1706	3rd
14951	14951	8344	8344	4023297	0.2168	0.0031	0.3918	0.0194	0.2593	0.7117	0.0941	0.45	FS
14959	14959	33889	33889	4023297	0.095	0.0403	0.2592	0.3499	0.0272	0.0546	0	0.0273	UN
14959	14959	33908	33908	4023297	0.1122	0.0283	0.2819	0.2591	0.0715	0.5112	0.0291	0.2846	2nd
14989	14989	7937	7937	4023297	0.1031	0.0102	0.3228	0.0907	0.114	0.3448	0	0.1724	3rd
15009	15009	34857	34857	4023297	0.1144	0.0369	0.2554	0.3424	0.0146	0.3058	0	0.1529	3rd
15009	15009	7091	7091	4023297	0.1187	0.0242	0.2941	0.237	0.115	0.4995	0	0.2497	2nd
15107	15107	33889	33889	4023297	0.0985	0.0374	0.275	0.3195	0.0512	0.0792	0.015	0.0546	4th
15107	15107	7937	7937	4023297	0.131	0.0001	0.3712	0.0008	0.2562	0.9088	0.0874	0.5418	PO
15113	15113	6716	6716	4023297	0.1754	0.0489	0.2823	0.4284	0.081	0.0013	0	0.0006	UN
15113	15113	8344	8344	4023297	0.1752	0.0639	0.2848	0.4147	0.0456	0.0017	0	0.0009	UN
15149	15149	34857	34857	4023297	0.1251	0.0466	0.2432	0.4088	0.0422	0.0096	0.0015	0.0063	UN
15150	15150	7091	7091	4023297	0.1038	0.0294	0.282	0.2468	0.0161	0.5889	0.01	0.3044	2nd
15150	15150	8134	8134	4023297	0.0907	0.0139	0.3277	0.1086	0.1451	0.3278	0.0729	0.2367	2nd
15156	15156	33524	33524	4023297	0.1059	0.0165	0.2541	0.1471	0.1389	0.0438	0	0.0219	UN
15156	15156	33545	33545	4023297	0.0925	0.0205	0.2547	0.18	0.0472	0.0114	0	0.0057	UN
15156	15156	33605	33605	4023297	0.0909	0.0317	0.2127	0.2455	0.0502	0.0054	0	0.0027	UN
15156	15156	33889	33889	4023297	0.1232	0.0054	0.3362	0.0569	0.2106	0.257	0.008	0.1365	3rd
15156	15156	33908	33908	4023297	0.1313	0.0009	0.3227	0.0095	0.2325	0.954	0.0092	0.4862	PO
15156	15156	34859	34859	4023297	0.1038	0.0144	0.2935	0.1374	0.109	0.03	0	0.015	UN
15156	15156	34897	34897	4023297	0.1061	0.0119	0.2982	0.1163	0.13	0.0749	0	0.0374	UN
15156	15156	7091	7091	4023297	0.112	0.0446	0.2646	0.4568	0.0307	0.024	0	0.012	UN
15156	15156	7478	7478	4023297	0.1204	0.0091	0.3466	0.093	0.1817	0.201	0.0041	0.1046	3rd
15156	15156	7937	7937	4023297	0.1109	0.0397	0.2731	0.3939	0.057	0.1924	0	0.0962	3rd
15156	15156	8170	8170	4023297	0.1097	0.0458	0.2615	0.4691	0.0276	0.0266	0	0.0133	UN
15197	15197	33599	33599	4023297	0.1463	0.0204	0.2884	0.132	0.1181	0.061	0	0.0305	UN
15197	15197	33631	33631	4023297	0.1527	0.0117	0.3062	0.0872	0.1584	0.0996	0	0.0498	4th
15197	15197	33874	33874	4023297	0.1245	0.0234	0.263	0.1629	0.0272	0.0081	0	0.0041	UN
15197	15197	6716	6716	4023297	0.1667	0.0574	0.2727	0.4224	0.0368	0.0139	0	0.0069	UN
15211	15211	34859	34859	4023297	0.0882	0.0202	0.2679	0.1732	0.0905	0.0012	0	0.0006	UN
15244	15244	8170	8170	4023297	0.1003	0.013	0.2758	0.1087	0.0972	0.3939	0	0.197	2nd
14924	14924	8653	8653	4023297	0.0738	0.0173	0.2581	0.1287	0.02	0.2343	0.0531	0.1703	3rd
14925	14925	8995	8995	4023297	0.1091	0.0361	0.2682	0.3131	0.0242	0.3548	0	0.1774	2nd
14925	14925	9562	9562	4023297	0.1099	0.031	0.2777	0.2824	0.059	0.3903	0	0.1951	2nd
14930	14930	9841	9841	4023297	0.1123	0.0327	0.2833	0.3193	0.0785	0.3329	0.0044	0.1709	3rd
14943	14943	9860	9860	4023297	0.1377	0.018	0.2768	0.1206	0.0686	0.0772	0	0.0386	UN
14944	14944	9656	9656	4023297	0.1689	0.0346	0.2908	0.1585	0.0826	0.0557	0	0.0278	UN
14959	14959	8780	8780	4023297	0.1388	0.0001	0.3768	0.001	0.2531	0.9212	0.0767	0.5373	PO
15009	15009	8653	8653	4023297	0.1035	0.0252	0.2894	0.2128	0.0839	0.5506	0.0694	0.3447	2nd
15113	15113	8465	8465	4023297	0.1618	0.0503	0.2685	0.4462	0.0792	0	0	0	UN
15113	15113	9656	9656	4023297	0.1808	0.0138	0.2907	0.069	0.1759	0.1247	0	0.0623	4th
15113	15113	9860	9860	4023297	0.1788	0.0359	0.3037	0.322	0.1384	0.0457	0	0.0228	UN
15122	15122	8465	8465	4023297	0.1565	0.0483	0.2839	0.3325	0.0497	0.2764	0.0013	0.1395	3rd
15149	15149	9860	9860	4023297	0.1503	0.0447	0.2686	0.3762	0.0478	0.0169	0	0.0085	UN
15150	15150	8653	8653	4023297	0.0941	0.0305	0.2975	0.23	0.0638	0.5625	0.1322	0.4135	FS
15156	15156	8780	8780	4023297	0.1586	0.0001	0.4212	0.001	0.2908	0.8831	0.1142	0.5557	PO
15156	15156	9562	9562	4023297	0.1118	0.0444	0.2646	0.452	0.0319	0.0136	0	0.0068	UN
15156	15156	9841	9841	4023297	0.1078	0.0464	0.2594	0.4694	0.0273	0.0062	0	0.0031	UN
15163	15163	8653	8653	4023297	0.078	0.0105	0.2738	0.0791	0.0845	0.3453	0.0719	0.2446	2nd
15190	15190	8995	8995	4023297	0.1171	0.03	0.2848	0.2768	0.0774	0.3586	0	0.1793	2nd
15190	15190	9562	9562	4023297	0.1457	0.0106	0.391	0.1124	0.225	0.6861	0.1287	0.4718	FS
15197	15197	8465	8465	4023297	0.1693	0.0556	0.2939	0.4179	0.0677	0.2204	0	0.1102	3rd
15197	15197	9860	9860	4023297	0.1544	0.0626	0.2598	0.4487	0.0256	0.0092	0	0.0046	UN
15211	15211	9841	9841	4023297	0.1017	0.036	0.2664	0.3335	0.0203	0.1326	0	0.0663	4th
15225	15225	9656	9656	4023297	0.1503	0.0321	0.2721	0.1204	0.0308	0.1977	0	0.0988	3rd
15232	15232	9656	9656	4023297	0.2001	0.0048	0.3684	0.0183	0.2196	0.6818	0.0589	0.3998	2nd
15274	15274	8780	8780	4023297	0.1083	0.0074	0.2894	0.0668	0.1453	0.3592	0.0013	0.1809	2nd
15419	15419	15467	15467	4023297	0.0879	0.0302	0.2657	0.2423	0.0278	0.2162	0.0515	0.1596	3rd
15444	15444	15458	15458	4023297	0.1172	0.016	0.3174	0.1523	0.1563	0.6539	0.0384	0.3654	2nd
15444	15444	15475	15475	4023297	0.1008	0.0293	0.2968	0.2518	0.0773	0.1602	0.0019	0.082	4th
15458	15458	15475	15475	4023297	0.1107	0.0241	0.3106	0.2227	0.0937	0.1182	0	0.0591	4th
15467	15467	15562	15562	4023297	0.1024	0.0267	0.2565	0.2181	0.0538	0.2852	0.0726	0.2152	2nd
15414	15414	15633	15633	4023297	0.0809	0.027	0.282	0.2002	0.0185	0.2659	0.0477	0.1806	2nd
15419	15419	15944	15944	4023297	0.0948	0.0295	0.2876	0.247	0.0423	0.1741	0.0352	0.1223	3rd
15421	15421	15633	15633	4023297	0.1059	0.0252	0.3235	0.2055	0.1063	0.5342	0.1389	0.406	FS
15421	15421	16067	16067	4023297	0.0969	0.0267	0.2842	0.2228	0.0811	0.2104	0.0133	0.1185	3rd
15444	15444	15845	15845	4023297	0.1089	0.0237	0.3043	0.2136	0.1262	0.2948	0.0282	0.1756	3rd
15458	15458	15845	15845	4023297	0.1241	0.0178	0.3361	0.176	0.1662	0.2767	0.0501	0.1884	2nd
15467	15467	15944	15944	4023297	0.1068	0.0226	0.3042	0.2014	0.13	0.5666	0.0605	0.3438	2nd
15475	15475	15845	15845	4023297	0.0952	0.0309	0.2704	0.2667	0.0484	0.0058	0	0.0029	UN
15509	15509	16019	16019	4023297	0.101	0.0287	0.3021	0.2425	0.0659	0.1386	0.0293	0.0986	3rd
15562	15562	15944	15944	4023297	0.0977	0.0347	0.2386	0.2853	0.014	0.1582	0.0212	0.1003	3rd
15575	15575	15628	15628	4023297	0.0801	0.0289	0.2537	0.2144	0.0214	0.2135	0.0149	0.1216	3rd
15419	15419	16083	16083	4023297	0.0954	0.0264	0.3224	0.2173	0.1029	0.0862	0.0439	0.087	4th
15467	15467	16083	16083	4023297	0.0963	0.0255	0.2929	0.216	0.0797	0.1197	0.0336	0.0934	3rd
15467	15467	16104	16104	4023297	0.0908	0.0304	0.2743	0.2462	0.0376	0.1509	0.0386	0.114	3rd
15494	15494	16371	16371	4023297	0.1205	0.0315	0.3096	0.2121	0.1024	0.0223	0.0579	0.0691	4th
15515	15515	16121	16121	4023297	0.127	0.0416	0.3288	0.2624	0.0755	0.0381	0.0245	0.0436	UN
15523	15523	16391	16391	4023297	0.1316	0.049	0.3172	0.2912	0.0562	0.0267	0.0112	0.0245	UN
15562	15562	16254	16254	4023297	0.1226	0.0311	0.3176	0.2397	0.0839	0.1155	0.0053	0.0631	4th
15566	15566	16115	16115	4023297	0.1506	0.0405	0.3129	0.1541	0.0723	0.139	0.0331	0.1026	3rd
15571	15571	16261	16261	4023297	0.0783	0.0249	0.2539	0.1931	0.0328	0.0451	0.0303	0.0528	4th
15575	15575	16261	16261	4023297	0.0855	0.027	0.2776	0.2147	0.0493	0.0694	0.0364	0.0711	4th
15626	15626	16263	16263	4023297	0.0927	0.03	0.2594	0.2287	0.0448	0.2273	0.035	0.1486	3rd
15292	15292	16562	16562	4023297	0.0831	0.0369	0.308	0.2819	0.0197	0.0275	0.065	0.0788	4th
15444	15444	16413	16413	4023297	0.1498	0.0114	0.4486	0.1183	0.25	0.5523	0.2942	0.5703	FS
15444	15444	16517	16517	4023297	0.1229	0.0182	0.3422	0.173	0.1639	0.6428	0.0955	0.4169	FS
15444	15444	16548	16548	4023297	0.0921	0.0235	0.2754	0.195	0.0723	0.165	0.0214	0.1039	3rd
15458	15458	16413	16413	4023297	0.1298	0.0193	0.3409	0.1947	0.1767	0.5703	0.0668	0.352	2nd
15458	15458	16517	16517	4023297	0.1261	0.019	0.3295	0.191	0.1705	0.5963	0.0429	0.3411	2nd
15458	15458	16548	16548	4023297	0.1028	0.0251	0.2933	0.2186	0.0572	0.1709	0.0017	0.0871	4th

FID1	ID1	FID2	ID2	N_SNP	HetHet	IBS0	HetConc	HomIBS0	Kinship	IBD1Seg	IBD2Seg	PropIBD	InfType
15475	15475	16413	16413	4023297	0.1131	0.0237	0.3218	0.2176	0.1043	0.1507	0.003	0.0783	4th
15475	15475	16517	16517	4023297	0.1066	0.0259	0.2992	0.2361	0.0804	0.1236	0	0.0618	4th
15475	15475	16548	16548	4023297	0.0908	0.0367	0.2872	0.297	0.0263	0.0098	0.0021	0.007	UN
15566	15566	16743	16743	4023297	0.1537	0.065	0.2857	0.2454	0.0319	0.0859	0.0115	0.0545	4th
15571	15571	16538	16538	4023297	0.0797	0.0256	0.3159	0.1904	0.059	0.017	0.069	0.0775	4th
15626	15626	16550	16550	4023297	0.1286	0.0131	0.4488	0.1142	0.2423	0.5071	0.3585	0.6121	FS
15626	15626	16692	16692	4023297	0.0933	0.0385	0.2832	0.2967	0.0383	0.3325	0.0431	0.2094	2nd
15626	15626	16702	16702	4023297	0.1011	0.0215	0.2968	0.176	0.1148	0.696	0.0507	0.3987	2nd
15419	15419	17112	17112	4023297	0.0777	0.0358	0.2502	0.2726	0.0122	0.0562	0.028	0.0561	4th
15467	15467	16775	16775	4023297	0.1026	0.0195	0.3009	0.1692	0.1365	0.3188	0.031	0.1904	2nd
15467	15467	17112	17112	4023297	0.0878	0.0249	0.2629	0.2031	0.0579	0.2523	0.0288	0.155	3rd
15562	15562	16775	16775	4023297	0.1005	0.0253	0.2561	0.2118	0.0482	0.0883	0.027	0.0711	4th
15566	15566	16862	16862	4023297	0.162	0.0642	0.3148	0.2418	0.0419	0.1549	0.0567	0.1342	3rd
15599	15599	16780	16780	4023297	0.1738	0.0378	0.3644	0.2593	0.1503	0.0199	0.0163	0.0262	UN
15419	15419	17325	17325	4023297	0.0799	0.0343	0.2658	0.2684	0.0237	0.0291	0.0166	0.0311	UN
15444	15444	17199	17199	4023297	0.1321	0.0104	0.3888	0.101	0.2289	0.6387	0.2177	0.537	FS
15458	15458	17199	17199	4023297	0.1252	0.0281	0.3355	0.2702	0.1277	0.4399	0.0926	0.3125	2nd
15458	15458	17340	17340	4023297	0.1016	0.0263	0.3016	0.2275	0.0317	0.2024	0.0034	0.1046	3rd
15467	15467	17325	17325	4023297	0.0957	0.026	0.3005	0.219	0.0653	0.1805	0.0167	0.1069	3rd
15475	15475	17199	17199	4023297	0.105	0.0339	0.3023	0.2966	0.0513	0.1412	0.0012	0.0718	4th
15566	15566	17254	17254	4023297	0.1458	0.0584	0.277	0.2124	0.0323	0.1329	0.0462	0.1126	3rd
15566	15566	17295	17295	4023297	0.1665	0.0565	0.2978	0.2784	0.0503	0	0	0	UN
15566	15566	17833	17833	4023297	0.1529	0.0574	0.2818	0.2251	0.0499	0.0499	0.007	0.032	UN
15599	15599	17295	17295	4023297	0.1637	0.0541	0.3015	0.3569	0.0414	0	0	0	UN
15626	15626	17222	17222	4023297	0.0852	0.0318	0.2392	0.2389	0.0287	0.1801	0.0022	0.0923	3rd
15414	15414	18022	18022	4023297	0.0809	0.0303	0.307	0.2287	0.0337	0.0463	0.0435	0.0667	4th
15421	15421	17981	17981	4023297	0.098	0.0232	0.2921	0.1909	0.0969	0.2155	0.0195	0.1272	3rd
15421	15421	18022	18022	4023297	0.0903	0.0307	0.2826	0.245	0.0149	0.1753	0.032	0.1196	3rd
15444	15444	17903	17903	4023297	0.1187	0.0148	0.3436	0.1371	0.1889	0.6417	0.1344	0.4552	FS
15444	15444	18385	18385	4023297	0.136	0.0109	0.4273	0.1057	0.2481	0.5448	0.2919	0.5643	FS
15444	15444	18599	18599	4023297	0.0954	0.0301	0.2818	0.2525	0.0546	0.1185	0.0164	0.0756	4th
15458	15458	17903	17903	4023297	0.1182	0.0231	0.3171	0.2187	0.1295	0.5482	0.0631	0.3372	2nd
15458	15458	18385	18385	4023297	0.1144	0.0202	0.3122	0.1864	0.1293	0.6597	0.0405	0.3703	2nd
15458	15458	18523	18523	4023297	0.1063	0.0249	0.3155	0.2235	0.0577	0.189	0	0.0945	3rd
15458	15458	18599	18599	4023297	0.1009	0.0274	0.2804	0.244	0.0481	0.0879	0.004	0.048	4th
15475	15475	17903	17903	4023297	0.1014	0.0277	0.2952	0.2411	0.0813	0.1452	0.0082	0.0808	4th
15475	15475	18385	18385	4023297	0.0943	0.0255	0.2769	0.2174	0.0864	0.0905	0.0131	0.0583	4th
15475	15475	18599	18599	4023297	0.0863	0.0354	0.2628	0.2889	0.0298	0.0031	0	0.0015	UN
15509	15509	17998	17998	4023297	0.1	0.0398	0.2676	0.3601	0.0396	0.0134	0	0.0067	UN
15515	15515	18469	18469	4023297	0.1421	0.0288	0.3329	0.1921	0.1014	0.1032	0.0112	0.0628	4th
15523	15523	18469	18469	4023297	0.1385	0.0373	0.3081	0.2313	0.0737	0.064	0.0035	0.0355	UN
15566	15566	18528	18528	4023297	0.1552	0.0616	0.2838	0.2951	0.0364	0	0	0	UN
15579	15579	18469	18469	4023297	0.1307	0.039	0.2951	0.2524	0.0417	0.0553	0.0103	0.038	UN
15419	15419	19674	19674	4023297	0.083	0.0303	0.267	0.2324	0.0472	0.0772	0.0391	0.0777	4th
15444	15444	19181	19181	4023297	0.1176	0.0201	0.3239	0.1877	0.1459	0.6262	0.0659	0.379	2nd
15444	15444	19348	19348	4023297	0.1396	0.0099	0.4207	0.0979	0.2478	0.612	0.26	0.566	FS
15458	15458	19181	19181	4023297	0.1233	0.018	0.3211	0.18	0.168	0.6422	0.0275	0.3486	2nd
15458	15458	19348	19348	4023297	0.1225	0.0199	0.326	0.1925	0.1555	0.6397	0.0386	0.3585	2nd
15467	15467	19674	19674	4023297	0.0978	0.0189	0.2967	0.1561	0.118	0.3459	0.0541	0.227	2nd
15475	15475	19181	19181	4023297	0.1044	0.0248	0.2927	0.2234	0.0829	0.1167	0	0.0584	4th
15475	15475	19348	19348	4023297	0.1047	0.0306	0.3015	0.2687	0.0664	0.1741	0	0.0871	4th
15523	15523	18818	18818	4023297	0.1168	0.0444	0.2627	0.2897	0.0319	0.0288	0.0026	0.017	UN
15523	15523	19012	19012	4023297	0.1266	0.0332	0.2866	0.2151	0.0853	0.0667	0.0253	0.0587	4th
15579	15579	18818	18818	4023297	0.1238	0.044	0.2925	0.2921	0.0349	0.0162	0.0276	0.0357	UN
15279	15279	1X2231	1X2231	4023297	0.0993	0.006	0.3326	0.0497	0.1005	0.5912	0.0196	0.3152	2nd
15290	15290	1X3796	1X3796	4023297	0.2042	0.0095	0.3693	0.0551	0.1586	0.3512	0	0.1756	3rd
15292	15292	1X3697	1X3697	4023297	0.1025	0.0078	0.2947	0.0695	0.1135	0.2951	0.0028	0.1503	3rd
15308	15308	1X4209	1X4209	4023297	0.1807	0.0218	0.3213	0.1573	0.0547	0.0045	0	0.0022	UN
15421	15421	1X1155	1X1155	4023297	0.1096	0.0199	0.3055	0.1746	0.1403	0.5926	0.0554	0.3516	2nd
15444	15444	1X2124	1X2124	4023297	0.1155	0.0365	0.2882	0.3282	0.0308	0.4429	0	0.2214	2nd
15444	15444	1X3656	1X3656	4023297	0.1057	0.0393	0.2771	0.3522	0.0294	0.3869	0	0.1934	2nd
15444	15444	25354	25354	4023297	0.1152	0.0217	0.3272	0.1971	0.1479	0.3725	0.1061	0.2924	2nd
15458	15458	1X2124	1X2124	4023297	0.131	0.0208	0.3182	0.2097	0.1455	0.6161	0	0.3081	2nd
15458	15458	25354	25354	4023297	0.1179	0.0267	0.3136	0.2519	0.1157	0.33	0.0362	0.2012	2nd
15458	15458	26198	26198	4023297	0.1038	0.029	0.3024	0.2564	0.0331	0.2154	0.0088	0.1165	3rd
15475	15475	1X2124	1X2124	4023297	0.1121	0.0245	0.2915	0.2248	0.0601	0.0347	0	0.0173	UN
15475	15475	25354	25354	4023297	0.0978	0.0303	0.2793	0.2624	0.0565	0.0417	0.0084	0.0293	UN
15509	15509	1X3697	1X3697	4023297	0.146	0.0013	0.4051	0.0142	0.2665	0.7102	0.1597	0.5149	FS
15509	15509	1X3822	1X3822	4023297	0.1138	0.0273	0.2863	0.2665	0.082	0.3495	0.0018	0.1765	3rd
15523	15523	1X2816	1X2816	4023297	0.1063	0.0461	0.2482	0.3454	0.0216	0.233	0.0122	0.1288	3rd
15560	15560	1X2231	1X2231	4023297	0.0982	0.0112	0.2962	0.0951	0.095	0.2755	0.0027	0.1405	3rd
15566	15566	25355	25355	4023297	0.1392	0.0569	0.2698	0.2076	0.0147	0.0403	0.0332	0.0533	4th
15568	15568	1X4209	1X4209	4023297	0.1853	0.0161	0.3197	0.1096	0.0975	0.0847	0	0.0423	UN
15579	15579	1X2816	1X2816	4023297	0.1019	0.0405	0.2435	0.3045	0.0317	0.2644	0.0044	0.1366	3rd
15584	15584	1X2816	1X2816	4023297	0.1038	0.0263	0.272	0.2191	0.0661	0.3697	0.0356	0.2205	2nd
15626	15626	1X2816	1X2816	4023297	0.1148	0.0288	0.3178	0.2586	0.0722	0.642	0.0586	0.3796	2nd
15444	15444	26988	26988	4023297	0.1237	0.0148	0.3521	0.1397	0.1881	0.6807	0.1057	0.4461	FS
15444	15444	27503	27503	4023297	0.11	0.0172	0.3024	0.1572	0.149	0.6711	0.0359	0.3714	2nd
15458	15458	26988	26988	4023297	0.1213	0.0248	0.3189	0.2398	0.1347	0.5343	0.0391	0.3063	2nd
15458	15458	27503	27503	4023297	0.1319	0.0163	0.3577	0.1632	0.1904	0.5628	0.1094	0.3908	FS
15475	15475	26988	26988	4023297	0.1009	0.0307	0.2844	0.2709	0.0528	0.1136	0.0124	0.0691	4th
15475	15475	27503	27503	4023297	0.103	0.0295	0.2931	0.2579	0.0651	0.0982	0	0.0491	4th
15421	15421	27889	27889	4023297	0.0871	0.0289	0.2685	0.2258	0.0179	0.2327	0.0129	0.1293	3rd
15444	15444	28246	28246	4023297	0.1223	0.0249	0.3355	0.2416	0.1284	0.5049	0.0958	0.3482	2nd
15458	15458	28246	28246	4023297	0.1302	0.0223	0.3398	0.2346	0.1666	0.5192	0.0622	0.3218	2nd
15475	15475	28246	28246	4023297	0.1062	0.0229	0.294	0.2166	0.0883	0.1696	0	0.0848	4th
15509	15509	27989	27989	4023297	0.1246	0.0039	0.3901	0.0376	0.2482	0.336	0.1174	0.2854	2nd
15509	15509	28183	28183	4023297	0.0979	0.0338	0.2829	0.2787	0.0413	0.191	0.0589	0.1545	3rd
15626	15626	28304	28304	4023297	0.1169	0.0178	0.4031	0.1524	0.1878	0.369	0.1911	0.3756	FS
15494	15494	28635	28635	4023297	0.1082	0.0299	0.263	0.2118	0.0907	0.2407	0.0505	0.1708	3rd
15509	15509	28635	28635	4023297	0.0954	0.0351	0.24	0.2843	0.0292	0.3596	0.0358	0.2156	2nd
15421	15421	30907	30907	4023297	0.0844	0.0301	0.2116	0.2216	0.0239	0.0224	0	0.0112	UN
15458	15458	30961	30961	4023297	0.1041	0.0357	0.2652	0.3415	0.0509	0.0809	0	0.0405	UN
15467	15467	31235	31235	4023297	0.0803	0.0202	0.2515	0.1627	0.0392	0.0314	0	0.0157	UN
15599	15599	31065	31065	4023297	0.1536	0.0577	0.2882	0.3861	0.0304	0	0	0	UN
15626	15626	30933	30933	4023297	0.1015	0.0104	0.2167	0.0874	0.0166	0.102	0	0.051	4th
15626	15626	31086	31086	4023297	0.0807	0.0251	0.2233	0.1899	0.0484	0.0238	0	0.0119	UN
15458	15458	31333	31333	4023297	0.1012	0.0131	0.2875	0.1217	0.1143	0.0443	0	0.0222	UN

FID1	ID1	FID2	ID2	N_SNP	HetHet	IBS0	HetConc	HomIBS0	Kinship	IBD1Seg	IBD2Seg	PropIBD	InfType
15562	15562	31297	31297	4023297	0.1029	0.0436	0.2275	0.367	0.0246	0.0797	0.0014	0.0412	UN
15562	15562	31364	31364	4023297	0.115	0.0388	0.2427	0.318	0.0334	0.0343	0	0.0171	UN
15523	15523	31506	31506	4023297	0.1119	0.0287	0.2537	0.1757	0.0888	0.0021	0	0.001	UN
15458	15458	31902	31902	4023297	0.1068	0.0117	0.3009	0.1102	0.1404	0.03	0	0.015	UN
15444	15444	32043	32043	4023297	0.1145	0.0252	0.284	0.2322	0.0765	0.6094	0	0.3047	2nd
15458	15458	32043	32043	4023297	0.1194	0.0314	0.2809	0.3076	0.0798	0.3681	0.0015	0.1855	2nd
15467	15467	32025	32025	4023297	0.1046	0.0332	0.2674	0.2986	0.0362	0.3963	0.0016	0.1997	2nd
15523	15523	32358	32358	4023297	0.1277	0.018	0.2705	0.1215	0.1145	0.0139	0	0.007	UN
15562	15562	32025	32025	4023297	0.0996	0.0393	0.2235	0.334	0.0336	0.1256	0	0.0628	4th
15562	15562	32040	32040	4023297	0.1053	0.0383	0.2296	0.3228	0.0402	0.1187	0	0.0593	4th
15599	15599	32207	32207	4023297	0.1354	0.0454	0.2701	0.2492	0.061	0	0	0	UN
15444	15444	32849	32849	4023297	0.1131	0.0259	0.2652	0.2364	0.0469	0.6193	0	0.3097	2nd
15458	15458	32560	32560	4023297	0.095	0.0166	0.2755	0.1488	0.0686	0.0244	0	0.0122	UN
15458	15458	32849	32849	4023297	0.1188	0.0323	0.2657	0.3142	0.0542	0.3365	0	0.1682	3rd
15458	15458	33115	33115	4023297	0.1076	0.0108	0.2986	0.1026	0.1499	0.0581	0	0.029	UN
15414	15414	7937	7937	4023297	0.0953	0.0094	0.2943	0.0802	0.0916	0.3541	0	0.1771	2nd
15421	15421	33889	33889	4023297	0.0963	0.0375	0.2666	0.3199	0.0462	0.0785	0	0.0392	UN
15421	15421	7937	7937	4023297	0.1294	0.0001	0.3643	0.0008	0.2526	0.9105	0.0863	0.5415	PO
15444	15444	33863	33863	4023297	0.1089	0.0349	0.2671	0.3119	0.0231	0.431	0	0.2155	2nd
15458	15458	33863	33863	4023297	0.1192	0.0324	0.281	0.3153	0.0768	0.345	0	0.1725	3rd
15467	15467	33908	33908	4023297	0.1081	0.0303	0.2735	0.267	0.0485	0.4992	0.022	0.2716	2nd
15509	15509	7091	7091	4023297	0.1134	0.0284	0.2865	0.2777	0.0784	0.3409	0	0.1704	3rd
15515	15515	6716	6716	4023297	0.1667	0.0105	0.3348	0.0788	0.1279	0.3511	0.0071	0.1826	2nd
15523	15523	6716	6716	4023297	0.1835	0.0066	0.3666	0.0515	0.182	0.3918	0.0169	0.2128	2nd
15545	15545	8344	8344	4023297	0.1445	0.0275	0.2849	0.153	0.0153	0.212	0.0013	0.1073	3rd
15553	15553	7311	7311	4023297	0.0952	0.0151	0.2665	0.1259	0.0718	0.3992	0	0.1996	2nd
15562	15562	33908	33908	4023297	0.1117	0.0347	0.2532	0.2946	0.0752	0.1431	0.0369	0.1084	3rd
15579	15579	6716	6716	4023297	0.1828	0.0074	0.3755	0.0586	0.1738	0.4665	0.0053	0.2386	2nd
15584	15584	6716	6716	4023297	0.1502	0.0092	0.3099	0.075	0.0794	0.3635	0.0051	0.1869	2nd
15626	15626	7311	7311	4023297	0.1078	0.0355	0.2932	0.3074	0.0242	0.5168	0.0389	0.2973	2nd
15626	15626	8170	8170	4023297	0.1401	0.0001	0.4128	0.0006	0.2637	0.775	0.2205	0.608	PO
15419	15419	8780	8780	4023297	0.1174	0.003	0.3359	0.0293	0.183	0.5123	0.0274	0.2836	2nd
15444	15444	9841	9841	4023297	0.1382	0.0001	0.39	0.0006	0.2648	0.8635	0.1339	0.5657	PO
15458	15458	9841	9841	4023297	0.1192	0.0267	0.2981	0.2737	0.1224	0.4095	0	0.2048	2nd
15467	15467	8780	8780	4023297	0.1495	0	0.4258	0.0005	0.2769	0.805	0.1924	0.5949	PO
15509	15509	8653	8653	4023297	0.0953	0.0339	0.2701	0.2783	0.0388	0.4082	0.0354	0.2395	2nd
15562	15562	8780	8780	4023297	0.1418	0.0027	0.3476	0.0279	0.2475	0.435	0.1683	0.3858	FS
15566	15566	9656	9656	4023297	0.211	0.0086	0.3816	0.0366	0.2266	0.4073	0.092	0.2956	2nd
15581	15581	9656	9656	4023297	0.1584	0.0158	0.3081	0.0558	0.0851	0.4883	0.0015	0.2457	2nd
15599	15599	9656	9656	4023297	0.17	0.0373	0.2957	0.1691	0.0734	0.08	0	0.04	UN
15633	15633	16067	16067	4023297	0.0906	0.0275	0.2802	0.2173	0.0811	0.1858	0.0238	0.1168	3rd
15824	15824	15940	15940	4023297	0.098	0.0224	0.3026	0.1843	0.1132	0.1225	0.0295	0.0908	3rd
15870	15870	15940	15940	4023297	0.0834	0.0327	0.2688	0.2549	0.0292	0.0397	0.0136	0.0334	UN
15628	15628	16261	16261	4023297	0.0902	0.0188	0.2716	0.1513	0.1227	0.3046	0.0627	0.215	2nd
15944	15944	16083	16083	4023297	0.0921	0.0311	0.272	0.2663	0.0334	0.0637	0.0205	0.0523	4th
15975	15975	16094	16094	4023297	0.129	0.0496	0.2659	0.335	0.0257	0.0065	0	0.0032	UN
15628	15628	16741	16741	4023297	0.1176	0.0116	0.4145	0.0976	0.2192	0.4786	0.0919	0.3312	2nd
15824	15824	16409	16409	4023297	0.0877	0.0321	0.2744	0.2505	0.0237	0.1332	0.0276	0.0942	3rd
15824	15824	16527	16527	4023297	0.0968	0.0291	0.283	0.239	0.0852	0.1062	0.0299	0.083	4th
15845	15845	16413	16413	4023297	0.1195	0.0209	0.3219	0.2046	0.1461	0.281	0.0487	0.1892	2nd
15845	15845	16517	16517	4023297	0.1136	0.0223	0.3026	0.2146	0.1295	0.2716	0.026	0.1618	3rd
15940	15940	16527	16527	4023297	0.095	0.0266	0.2878	0.2204	0.0813	0.0552	0.0251	0.0527	4th
15976	15976	16702	16702	4023297	0.1059	0.0239	0.3549	0.1962	0.0854	0.3963	0.0148	0.2129	2nd
15659	15659	16986	16986	4023297	0.09	0.0335	0.2603	0.2857	0.0266	0.0073	0	0.0037	UN
15846	15846	16866	16866	4023297	0.1491	0.0546	0.3308	0.2237	0.0349	0.0731	0.0181	0.0547	4th
15944	15944	16775	16775	4023297	0.1008	0.0258	0.2891	0.2297	0.0968	0.1577	0.0258	0.1047	3rd
15944	15944	17112	17112	4023297	0.095	0.0257	0.286	0.2191	0.0652	0.2099	0.0374	0.1424	3rd
15975	15975	16853	16853	4023297	0.156	0.0493	0.2964	0.2698	0.0592	0.5805	0.0534	0.3437	2nd
15628	15628	17891	17891	4023297	0.093	0.0217	0.2617	0.1776	0.0883	0.0828	0	0.0414	UN
15845	15845	17199	17199	4023297	0.1102	0.0329	0.299	0.2989	0.0885	0.2252	0.0314	0.1441	3rd
15944	15944	17325	17325	4023297	0.0985	0.0235	0.3066	0.2091	0.0786	0.1717	0.0122	0.098	3rd
16019	16019	17233	17233	4023297	0.0866	0.0295	0.3023	0.2284	0.0396	0.0417	0.0284	0.0493	4th
16019	16019	17871	17871	4023297	0.0874	0.0312	0.3015	0.2389	0.0374	0.063	0.048	0.0795	4th
15628	15628	17998	17998	4023297	0.0939	0.0306	0.2633	0.2529	0.0463	0.0831	0	0.0416	UN
15633	15633	17981	17981	4023297	0.0828	0.0364	0.2535	0.2719	0.0241	0.1403	0.0251	0.0953	3rd
15633	15633	18022	18022	4023297	0.0846	0.0287	0.2806	0.2161	0.043	0.2261	0.0184	0.1314	3rd
15659	15659	18144	18144	4023297	0.0978	0.0326	0.2667	0.2924	0.066	0.003	0	0.0015	UN
15845	15845	17903	17903	4023297	0.1112	0.0235	0.3089	0.2149	0.1339	0.3511	0.0295	0.205	2nd
15845	15845	18385	18385	4023297	0.1054	0.0287	0.296	0.253	0.093	0.2871	0.042	0.1855	2nd
15845	15845	18599	18599	4023297	0.0979	0.0312	0.2854	0.2707	0.0467	0.0542	0.0092	0.0363	UN
15845	15845	19181	19181	4023297	0.119	0.0216	0.3229	0.21	0.1454	0.3074	0.0592	0.2129	2nd
15845	15845	19348	19348	4023297	0.1107	0.0221	0.3008	0.2036	0.1354	0.2959	0.015	0.163	3rd
15944	15944	19674	19674	4023297	0.0939	0.0348	0.2768	0.2858	0.0222	0.1945	0.0206	0.1178	3rd
15628	15628	1X3697	1X3697	4023297	0.1051	0.0243	0.2777	0.2137	0.0635	0.5782	0	0.2891	2nd
15628	15628	1X3822	1X3822	4023297	0.1035	0.0242	0.2687	0.2092	0.0543	0.5695	0	0.2848	2nd
15633	15633	1X1155	1X1155	4023297	0.0972	0.0238	0.2801	0.1946	0.0784	0.5323	0.0614	0.3275	2nd
15652	15652	1X2816	1X2816	4023297	0.1005	0.0189	0.2693	0.163	0.0833	0.361	0.0071	0.1876	2nd
15659	15659	1X3656	1X3656	4023297	0.1023	0.0383	0.266	0.3642	0.0264	0.0426	0	0.0213	UN
15659	15659	1X4179	1X4179	4023297	0.1195	0.0059	0.3235	0.0608	0.2026	0.2083	0	0.1042	3rd
15659	15659	1X4777	1X4777	4023297	0.1056	0.0379	0.2702	0.3588	0.0252	0.0404	0	0.0202	UN
15820	15820	1X3796	1X3796	4023297	0.16	0.0134	0.29	0.0781	0.0324	0.3528	0	0.1764	3rd
15824	15824	1X2231	1X2231	4023297	0.1034	0.0299	0.2773	0.2571	0.054	0.4233	0.0026	0.2142	2nd
15824	15824	1X4777	1X4777	4023297	0.1329	0.0015	0.378	0.0147	0.2424	0.7034	0.1248	0.4765	FS
15845	15845	1X2124	1X2124	4023297	0.1195	0.0289	0.2962	0.2773	0.0782	0.1926	0	0.0963	3rd
15845	15845	25354	25354	4023297	0.1139	0.0259	0.3163	0.2402	0.1307	0.1257	0.0144	0.0773	4th
15845	15845	26198	26198	4023297	0.095	0.031	0.2862	0.2654	0.0255	0.0854	0.0049	0.0476	4th
15870	15870	1X2231	1X2231	4023297	0.1001	0.022	0.2875	0.1916	0.0578	0.1896	0.0087	0.1035	3rd
15870	15870	1X4777	1X4777	4023297	0.1082	0.0092	0.3106	0.084	0.1355	0.2209	0	0.1104	3rd
15874	15874	1X3697	1X3697	4023297	0.1022	0.0079	0.3064	0.0689	0.0999	0.3886	0	0.1943	2nd
15889	15889	1X4777	1X4777	4023297	0.1083	0.0114	0.3111	0.1047	0.1244	0.1781	0	0.0891	3rd
15938	15938	1X4777	1X4777	4023297	0.1021	0.0089	0.3036	0.0775	0.1076	0.3057	0.0039	0.1568	3rd
15940	15940	1X4777	1X4777	4023297	0.1279	0.0046	0.3734	0.0455	0.2141	0.4223	0.0225	0.2337	2nd
15975	15975	1X4209	1X4209	4023297	0.251	0.0006	0.4556	0.0048	0.2653	0.7504	0.232	0.6072	PO
16000	16000	1X4777	1X4777	4023297	0.0923	0.0068	0.3058	0.0542	0.0365	0.6113	0	0.3057	2nd
16019	16019	1X3697	1X3697	4023297	0.1279	0.0037	0.3717	0.0363	0.2121	0.4098	0.0547	0.2596	2nd
15845	15845	26988	26988	4023297	0.1144	0.0305	0.3113	0.2817	0.1043	0.3154	0.0262	0.1839	2nd
15845	15845	27503	27503	4023297	0.1088	0.0242	0.2923	0.2237	0.1198	0.1867	0.0124	0.1057	3rd
15975	15975	26498	26498	4023297	0.1898	0.0339	0.3285	0.2999	0.0933	0	0	0	UN

FID1	ID1	FID2	ID2	N_SNP	HetHet	IBS0	HetConc	HomIBS0	Kinship	IBD1Seg	IBD2Seg	PropIBD	InfType
15628	15628	27989	27989	4023297	0.0848	0.0389	0.252	0.2932	0.0129	0.2295	0.0486	0.1633	3rd
15628	15628	28183	28183	4023297	0.0923	0.0266	0.2809	0.2074	0.0897	0.4179	0.0958	0.3047	2nd
15845	15845	28246	28246	4023297	0.1181	0.0207	0.3143	0.2087	0.1407	0.4494	0.0353	0.26	2nd
15874	15874	28183	28183	4023297	0.0859	0.0212	0.2991	0.1621	0.0645	0.2194	0.0531	0.1628	3rd
15940	15940	28212	28212	4023297	0.0812	0.0171	0.2775	0.132	0.0884	0.0358	0.0256	0.0434	UN
16019	16019	27989	27989	4023297	0.0913	0.0233	0.287	0.1872	0.1008	0.1148	0.024	0.0814	4th
16019	16019	28183	28183	4023297	0.0938	0.0305	0.2972	0.2399	0.0716	0.1507	0.0566	0.132	3rd
15628	15628	28635	28635	4023297	0.0991	0.022	0.2671	0.1689	0.076	0.6467	0.1017	0.4251	FS
15940	15940	28416	28416	4023297	0.0847	0.0339	0.2652	0.2657	0.0374	0.0455	0.0151	0.0379	UN
15628	15628	30917	30917	4023297	0.1219	0.0168	0.2704	0.1634	0.0334	0.0067	0	0.0033	UN
15633	15633	30878	30878	4023297	0.0738	0.0261	0.2363	0.1941	0.0278	0.0161	0	0.008	UN
15628	15628	31016	31016	4023297	0.1017	0.0074	0.2919	0.0639	0.1742	0.1878	0	0.0939	3rd
15846	15846	30933	30933	4023297	0.144	0.0269	0.2691	0.1352	0.1108	0.0012	0	0.0006	UN
16067	16067	30973	30973	4023297	0.082	0.0235	0.2577	0.1918	0.0674	0	0	0	UN
15944	15944	31297	31297	4023297	0.1066	0.0369	0.2634	0.3434	0.0194	0.2309	0.0014	0.1169	3rd
15944	15944	31299	31299	4023297	0.101	0.039	0.2548	0.3554	0.0131	0.0913	0	0.0456	4th
15628	15628	31542	31542	4023297	0.0897	0.0182	0.2389	0.1442	0.0774	0.0473	0	0.0236	UN
15846	15846	31525	31525	4023297	0.1295	0.0432	0.2563	0.2061	0.0639	0	0	0	UN
15940	15940	31553	31553	4023297	0.0857	0.0206	0.2656	0.1722	0.1081	0.0038	0	0.0019	UN
15944	15944	31500	31500	4023297	0.0971	0.0389	0.2582	0.3578	0.0313	0.0522	0	0.0261	UN
16067	16067	31578	31578	4023297	0.0905	0.0189	0.2809	0.1604	0.122	0.0057	0	0.0029	UN
15633	15633	31960	31960	4023297	0.0816	0.0219	0.2474	0.168	0.09	0.0127	0	0.0063	UN
15845	15845	32043	32043	4023297	0.1104	0.0339	0.2667	0.3196	0.0362	0.1242	0	0.0621	4th
15940	15940	31997	31997	4023297	0.081	0.0234	0.2549	0.1892	0.0762	0	0	0	UN
15944	15944	32025	32025	4023297	0.108	0.0333	0.2743	0.3137	0.0481	0.2352	0	0.1176	3rd
15944	15944	32040	32040	4023297	0.1047	0.0346	0.2519	0.3201	0.0154	0.2288	0	0.1144	3rd
16067	16067	32227	32227	4023297	0.0798	0.0248	0.2552	0.2004	0.046	0.0039	0	0.0019	UN
15633	15633	32993	32993	4023297	0.0816	0.0186	0.26	0.1464	0.0985	0.0229	0.0014	0.0128	UN
15845	15845	32849	32849	4023297	0.112	0.035	0.2579	0.3283	0.0122	0.1436	0	0.0718	4th
15940	15940	32772	32772	4023297	0.092	0.0161	0.2747	0.1375	0.1231	0.0084	0	0.0042	UN
15628	15628	7091	7091	4023297	0.1042	0.0292	0.2726	0.2519	0.0349	0.5314	0	0.2657	2nd
15633	15633	7937	7937	4023297	0.1239	0.0001	0.3677	0.0006	0.2395	0.8652	0.1285	0.561	PO
15652	15652	6716	6716	4023297	0.1391	0.0085	0.2875	0.0726	0.0466	0.3532	0.0013	0.1779	2nd
15652	15652	7267	7267	4023297	0.1105	0.01	0.2901	0.0869	0.1286	0.3879	0	0.1939	2nd
15736	15736	7937	7937	4023297	0.1028	0.0204	0.2509	0.1675	0.1208	0.1347	0	0.0674	4th
15845	15845	33863	33863	4023297	0.1098	0.0347	0.2654	0.3246	0.0327	0.1444	0	0.0722	4th
15846	15846	8344	8344	4023297	0.183	0.011	0.3357	0.0544	0.183	0.4421	0.0049	0.226	2nd
15944	15944	33908	33908	4023297	0.1107	0.027	0.2778	0.2536	0.0731	0.3739	0.0262	0.2131	2nd
15976	15976	8170	8170	4023297	0.1189	0.0057	0.3677	0.0533	0.1731	0.4876	0.0266	0.2704	2nd
15979	15979	7937	7937	4023297	0.0857	0.0118	0.2726	0.0973	0.0207	0.3448	0	0.1724	3rd
16006	16006	33908	33908	4023297	0.099	0.0274	0.2568	0.2115	0.0222	0.0553	0	0.0277	UN
16019	16019	8134	8134	4023297	0.0811	0.0209	0.2799	0.1606	0.0709	0.0432	0.0229	0.0445	4th
16065	16065	8344	8344	4023297	0.1645	0.0207	0.3168	0.1116	0.1033	0.2767	0	0.1383	3rd
16067	16067	33889	33889	4023297	0.1002	0.013	0.2964	0.1166	0.1542	0.012	0	0.006	UN
16067	16067	7937	7937	4023297	0.1248	0.0052	0.3665	0.0514	0.2177	0.3685	0.0243	0.2086	2nd
15628	15628	8653	8653	4023297	0.0951	0.0291	0.2878	0.2241	0.0859	0.5307	0.1027	0.368	FS
15664	15664	9562	9562	4023297	0.1026	0.0151	0.2873	0.1387	0.0773	0.0172	0	0.0086	UN
15824	15824	8995	8995	4023297	0.1046	0.0314	0.2636	0.2688	0.0208	0.2345	0	0.1172	3rd
15824	15824	9562	9562	4023297	0.108	0.0298	0.2818	0.2665	0.0478	0.3914	0	0.1957	2nd
15845	15845	9841	9841	4023297	0.1086	0.0316	0.2778	0.308	0.0686	0.1846	0	0.0923	3rd
15944	15944	8780	8780	4023297	0.1464	0.0009	0.4068	0.0103	0.2659	0.7726	0.142	0.5283	FS
16017	16017	8780	8780	4023297	0.1091	0.0118	0.3223	0.111	0.1007	0.2397	0	0.1198	3rd
16019	16019	8653	8653	4023297	0.0843	0.0333	0.2561	0.2532	0.0285	0.1853	0.0422	0.1348	3rd
16056	16056	8780	8780	4023297	0.104	0.0089	0.3125	0.0791	0.0932	0.3749	0	0.1874	2nd
16121	16121	16369	16369	4023297	0.1252	0.0425	0.298	0.2907	0.0572	0.0213	0.0261	0.0368	UN
16243	16243	16744	16744	4023297	0.131	0.0485	0.2663	0.3093	0.0344	0.0339	0	0.0169	UN
16261	16261	16741	16741	4023297	0.0871	0.0256	0.2791	0.2018	0.0671	0.0947	0.0229	0.0703	4th
16263	16263	16702	16702	4023297	0.0942	0.0254	0.2512	0.205	0.0852	0.1301	0.0244	0.0894	3rd
16369	16369	16671	16671	4023297	0.1194	0.0358	0.2793	0.2681	0.0726	0.0113	0.0461	0.0518	4th
16083	16083	16775	16775	4023297	0.0874	0.0316	0.2657	0.2602	0.0374	0.0337	0.0058	0.0226	UN
16094	16094	16853	16853	4023297	0.1394	0.0447	0.2716	0.262	0.0272	0.0252	0	0.0126	UN
16104	16104	17112	17112	4023297	0.0776	0.0337	0.2474	0.2596	0.0257	0.0227	0.0333	0.0447	4th
16243	16243	16787	16787	4023297	0.1419	0.0544	0.2982	0.3174	0.0295	0.0662	0.0075	0.0406	UN
16244	16244	16827	16827	4023297	0.0912	0.0309	0.3223	0.2512	0.0626	0.0306	0.0153	0.0306	UN
16261	16261	16829	16829	4023297	0.0982	0.0211	0.3484	0.172	0.1054	0.1872	0.0438	0.1375	3rd
16372	16372	16861	16861	4023297	0.0945	0.0344	0.2606	0.2821	0.0293	0.0387	0	0.0193	UN
16083	16083	17325	17325	4023297	0.0802	0.0315	0.2617	0.2532	0.0311	0.0092	0.0042	0.0088	UN
16121	16121	18469	18469	4023297	0.1417	0.0389	0.3228	0.2586	0.0687	0.0733	0.0039	0.0405	UN
16261	16261	17998	17998	4023297	0.0984	0.0213	0.2808	0.1905	0.099	0.0104	0	0.0052	UN
16369	16369	18341	18341	4023297	0.1313	0.043	0.3005	0.3044	0.0777	0.0119	0.0015	0.0074	UN
16369	16369	18469	18469	4023297	0.1374	0.0387	0.2962	0.2706	0.0746	0.0375	0.0107	0.0295	UN
16369	16369	18586	18586	4023297	0.1189	0.0453	0.2581	0.3077	0.0377	0.0114	0.0033	0.009	UN
16391	16391	18341	18341	4023297	0.1232	0.0523	0.2809	0.3194	0.0256	0.0025	0.0114	0.0127	UN
16391	16391	18469	18469	4023297	0.1348	0.0468	0.293	0.2805	0.0367	0.0242	0	0.0121	UN
16083	16083	19674	19674	4023297	0.0834	0.0343	0.2633	0.2675	0.0342	0.0613	0.0135	0.0442	UN
16121	16121	19012	19012	4023297	0.1372	0.043	0.3238	0.2884	0.0631	0.0724	0.0168	0.053	4th
16369	16369	18818	18818	4023297	0.1309	0.0394	0.2953	0.3016	0.0847	0.0173	0.0162	0.0248	UN
16369	16369	19012	19012	4023297	0.1215	0.039	0.2643	0.2774	0.0634	0.0233	0.0254	0.037	UN
16391	16391	18818	18818	4023297	0.1207	0.0495	0.2699	0.3132	0.0258	0.0068	0.003	0.0064	UN
16094	16094	1X4209	1X4209	4023297	0.1942	0.0115	0.3361	0.1	0.1323	0.0266	0	0.0133	UN
16121	16121	1X2816	1X2816	4023297	0.1155	0.0348	0.2805	0.2779	0.0853	0.2615	0.0075	0.1383	3rd
16243	16243	1X4209	1X4209	4023297	0.1916	0.0387	0.3126	0.2625	0.0566	0.1673	0	0.0837	4th
16244	16244	1X3697	1X3697	4023297	0.1116	0.0085	0.3279	0.0816	0.1362	0.2941	0	0.1471	3rd
16261	16261	1X3697	1X3697	4023297	0.1065	0.0252	0.2839	0.2341	0.0608	0.2726	0.0014	0.1377	3rd
16261	16261	1X3822	1X3822	4023297	0.1079	0.0229	0.285	0.2112	0.0692	0.3401	0.0042	0.1742	3rd
16263	16263	1X2816	1X2816	4023297	0.105	0.0258	0.2635	0.2297	0.0846	0.2366	0.0041	0.1224	3rd
16321	16321	1X2231	1X2231	4023297	0.1004	0.0392	0.2423	0.2998	0.0418	0.1989	0.0055	0.105	3rd
16321	16321	1X4777	1X4777	4023297	0.0994	0.0201	0.2344	0.1594	0.1058	0.1407	0	0.0704	4th
16338	16338	1X3697	1X3697	4023297	0.1231	0.006	0.3783	0.0583	0.1802	0.4797	0.0024	0.2422	2nd
16338	16338	1X4696	1X4696	4023297	0.0809	0.0211	0.3	0.1636	0.1068	0.0178	0.0729	0.0818	4th
16363	16363	1X4777	1X4777	4023297	0.097	0.0122	0.2908	0.1048	0.0652	0.2821	0	0.141	3rd
16369	16369	1X2816	1X2816	4023297	0.1181	0.0238	0.2752	0.2201	0.116	0.2606	0.0137	0.1439	3rd
16094	16094	26498	26498	4023297	0.1657	0.0303	0.2897	0.317	0.0486	0.0017	0	0.0008	UN
16243	16243	26498	26498	4023297	0.1694	0.0453	0.2819	0.365	0.0284	0	0	0	UN
16289	16289	27190	27190	4023297	0.1152	0.0342	0.2645	0.1778	0.0761	0.0271	0.0108	0.0243	UN
16243	16243	27985	27985	4023297	0.1437	0.0433	0.2976	0.2385	0.0751	0.0549	0.0475	0.0749	4th
16261	16261	27989	27989	4023297	0.0845	0.0355	0.2523	0.2791	0.0309	0.0558	0.0184	0.0463	4th
16261	16261	28183	28183	4023297	0.0827	0.0321	0.2459	0.2497	0.0429	0.0931	0.0192	0.0658	4th

FID1	ID1	FID2	ID2	N_SNP	HetHet	IBS0	HetConc	HomIBS0	Kinship	IBD1Seg	IBD2Seg	PropIBD	InfType
16263	16263	28304	28304	4023297	0.088	0.0301	0.2541	0.2346	0.0158	0.0712	0.0457	0.0813	4th
16261	16261	28635	28635	4023297	0.1032	0.0181	0.2827	0.1497	0.1025	0.3122	0.1214	0.2775	2nd
16289	16289	28478	28478	4023297	0.1213	0.0288	0.2731	0.1679	0.1109	0.0348	0.0108	0.0282	UN
16371	16371	28635	28635	4023297	0.118	0.0191	0.304	0.1418	0.1508	0.375	0.082	0.2695	2nd
16243	16243	31410	31410	4023297	0.1341	0.0556	0.2522	0.3435	0.0226	0.0111	0	0.0056	UN
16369	16369	31284	31284	4023297	0.1219	0.047	0.268	0.3751	0.0396	0.0113	0	0.0057	UN
16369	16369	31786	31786	4023297	0.122	0.049	0.2753	0.3907	0.0419	0.0052	0	0.0026	UN
16243	16243	32224	32224	4023297	0.1398	0.0593	0.2697	0.4032	0.0261	0.0265	0	0.0133	UN
16243	16243	32996	32996	4023297	0.129	0.0241	0.289	0.1325	0.087	0.106	0.004	0.057	4th
16092	16092	8170	8170	4023297	0.1076	0.0094	0.3426	0.0843	0.1021	0.4865	0.0012	0.2444	2nd
16121	16121	6716	6716	4023297	0.1775	0.0092	0.3557	0.0745	0.1589	0.3617	0.0047	0.1855	2nd
16122	16122	8170	8170	4023297	0.097	0.0074	0.3103	0.0645	0.0656	0.5193	0.0014	0.261	2nd
16261	16261	33874	33874	4023297	0.0828	0.0269	0.2289	0.2094	0.0405	0.0016	0	0.0008	UN
16261	16261	7091	7091	4023297	0.105	0.0255	0.2765	0.2347	0.0521	0.2942	0.0034	0.1505	3rd
16263	16263	7311	7311	4023297	0.0983	0.0372	0.243	0.3147	0.0233	0.1373	0.007	0.0756	4th
16263	16263	8170	8170	4023297	0.1256	0.0078	0.3292	0.0751	0.1991	0.3545	0.023	0.2002	2nd
16369	16369	6265	6265	4023297	0.1057	0.0443	0.2324	0.3732	0.0264	0.0444	0	0.0222	UN
16369	16369	6716	6716	4023297	0.2053	0.0057	0.4176	0.0534	0.2268	0.3589	0.0149	0.1943	2nd
16369	16369	6955	6955	4023297	0.1053	0.0429	0.2294	0.3633	0.0332	0.0729	0	0.0365	UN
16369	16369	7267	7267	4023297	0.1049	0.0405	0.228	0.3449	0.0417	0.0921	0	0.046	4th
16369	16369	7311	7311	4023297	0.12	0.0088	0.2809	0.0817	0.1762	0.2726	0.0165	0.1528	3rd
16369	16369	7625	7625	4023297	0.1058	0.0426	0.2317	0.3606	0.0336	0.0793	0	0.0397	UN
16369	16369	8170	8170	4023297	0.1047	0.0383	0.2345	0.3333	0.0388	0.1138	0.0031	0.06	4th
16369	16369	8307	8307	4023297	0.1094	0.0332	0.2502	0.29	0.0633	0.1357	0.0183	0.0861	4th
16386	16386	6716	6716	4023297	0.1375	0.0091	0.287	0.0653	0.0332	0.4581	0.0038	0.2328	2nd
16391	16391	6716	6716	4023297	0.2028	0.0103	0.4158	0.0789	0.2051	0.4176	0.0038	0.2126	2nd
16083	16083	8780	8780	4023297	0.1272	0.006	0.3676	0.062	0.1945	0.3527	0.0013	0.1777	2nd
16104	16104	8780	8780	4023297	0.1177	0.0059	0.3341	0.0571	0.1701	0.3147	0	0.1573	3rd
16115	16115	9656	9656	4023297	0.1606	0.0162	0.2933	0.0588	0.1079	0.4311	0.0133	0.2289	2nd
16243	16243	8465	8465	4023297	0.1516	0.0511	0.2733	0.3556	0.0324	0.1334	0	0.0667	4th
16254	16254	8780	8780	4023297	0.1164	0.012	0.2976	0.1101	0.1537	0.2577	0.0073	0.1362	3rd
16261	16261	8653	8653	4023297	0.0963	0.021	0.2945	0.1718	0.1254	0.3028	0.0715	0.2229	2nd
16329	16329	9656	9656	4023297	0.1693	0.0209	0.3226	0.083	0.0995	0.3802	0.0015	0.1915	2nd
16369	16369	9045	9045	4023297	0.1103	0.0369	0.2535	0.3177	0.05	0.1108	0.0112	0.0666	4th
16369	16369	9128	9128	4023297	0.1044	0.0432	0.2282	0.3661	0.0288	0.0931	0	0.0466	4th
16383	16383	9656	9656	4023297	0.1632	0.0194	0.3192	0.0737	0.0814	0.4163	0	0.2082	2nd
16408	16408	16413	16413	4023297	0.0989	0.0265	0.2911	0.2292	0.0304	0.1782	0.0376	0.1267	3rd
16408	16408	16517	16517	4023297	0.0929	0.027	0.27	0.2304	0.0136	0.1815	0.0221	0.1129	3rd
16413	16413	16517	16517	4023297	0.1458	0.0132	0.4046	0.1412	0.235	0.6065	0.176	0.4793	FS
16420	16420	16550	16550	4023297	0.0849	0.0319	0.2701	0.2456	0.0425	0.1863	0.0076	0.1007	3rd
16420	16420	16702	16702	4023297	0.0924	0.0276	0.2773	0.2272	0.0502	0.168	0.0098	0.0938	3rd
16517	16517	16548	16548	4023297	0.0994	0.0283	0.2846	0.2444	0.038	0.2088	0.0034	0.1078	3rd
16550	16550	16692	16692	4023297	0.0903	0.029	0.2776	0.2284	0.0697	0.2474	0.0382	0.1619	3rd
16550	16550	16702	16702	4023297	0.0997	0.0205	0.2976	0.1685	0.1115	0.593	0.0736	0.3701	2nd
16692	16692	16702	16702	4023297	0.0992	0.03	0.2891	0.2513	0.0702	0.2192	0.0214	0.131	3rd
16510	16510	16986	16986	4023297	0.1101	0.0218	0.3696	0.1916	0.1597	0.1716	0.0497	0.1354	3rd
16743	16743	16862	16862	4023297	0.1551	0.0439	0.2954	0.1636	0.0899	0.2677	0.0578	0.1916	2nd
16744	16744	16787	16787	4023297	0.12	0.0485	0.2547	0.3114	0.0352	0.0262	0	0.0131	UN
16408	16408	17199	17199	4023297	0.0948	0.0267	0.2857	0.2246	0.0343	0.1706	0.0211	0.1064	3rd
16413	16413	17199	17199	4023297	0.1466	0.0173	0.4197	0.1767	0.2193	0.5308	0.2395	0.5049	FS
16517	16517	17199	17199	4023297	0.126	0.0143	0.3423	0.1414	0.1908	0.6762	0.0792	0.4173	2nd
16548	16548	17199	17199	4023297	0.0908	0.0305	0.261	0.255	0.0177	0.1313	0.0246	0.0902	3rd
16550	16550	17222	17222	4023297	0.0842	0.0277	0.2406	0.2121	0.0388	0.1296	0.0273	0.0921	3rd
16692	16692	17184	17184	4023297	0.0796	0.0346	0.2258	0.2599	0.0142	0.0544	0.0359	0.0631	4th
16702	16702	17222	17222	4023297	0.0933	0.0373	0.2539	0.2954	0.0401	0.0917	0.0123	0.0581	4th
16743	16743	17254	17254	4023297	0.1542	0.0512	0.2957	0.1847	0.0644	0.1928	0.029	0.1254	3rd
16743	16743	17295	17295	4023297	0.1613	0.0571	0.2838	0.2704	0.043	0.0052	0	0.0026	UN
16743	16743	17833	17833	4023297	0.1578	0.0477	0.2918	0.1833	0.087	0.071	0.0112	0.0467	4th
16408	16408	18385	18385	4023297	0.0865	0.0299	0.2682	0.2394	0.0187	0.1677	0.036	0.1198	3rd
16413	16413	17903	17903	4023297	0.1232	0.0249	0.3378	0.2333	0.1357	0.5701	0.0902	0.3753	FS
16413	16413	18385	18385	4023297	0.1382	0.0161	0.4064	0.159	0.2033	0.5462	0.2298	0.5029	FS
16413	16413	18599	18599	4023297	0.1131	0.0214	0.3279	0.1973	0.1109	0.221	0.0174	0.1279	3rd
16517	16517	17903	17903	4023297	0.1431	0.0132	0.4169	0.1364	0.2297	0.5525	0.2423	0.5185	FS
16517	16517	18385	18385	4023297	0.1342	0.0141	0.3916	0.1391	0.2048	0.544	0.2159	0.4879	FS
16517	16517	18599	18599	4023297	0.1238	0.0182	0.3724	0.1745	0.1549	0.324	0.0151	0.1771	2nd
16548	16548	17903	17903	4023297	0.0929	0.0298	0.2749	0.2473	0.0365	0.169	0.0284	0.1129	3rd
16548	16548	18385	18385	4023297	0.0919	0.0301	0.2795	0.2473	0.0452	0.1476	0.0558	0.1296	3rd
16671	16671	18341	18341	4023297	0.1212	0.0468	0.2829	0.3222	0.032	0.0048	0.0033	0.0056	UN
16671	16671	18469	18469	4023297	0.1293	0.0394	0.2855	0.2686	0.0446	0.0311	0.0069	0.0225	UN
16671	16671	18586	18586	4023297	0.12	0.0402	0.2724	0.2733	0.0441	0.0189	0.0131	0.0226	UN
16743	16743	18528	18528	4023297	0.1506	0.0627	0.2714	0.2891	0.0287	0	0	0	UN
16744	16744	18395	18395	4023297	0.1294	0.0411	0.3163	0.2652	0.0387	0.0456	0.012	0.0348	UN
16408	16408	19181	19181	4023297	0.0943	0.0283	0.2766	0.2403	0.0128	0.1999	0.0169	0.1169	3rd
16408	16408	19348	19348	4023297	0.0894	0.0282	0.2652	0.2313	0.0123	0.1953	0.0139	0.1116	3rd
16413	16413	19181	19181	4023297	0.1397	0.0129	0.383	0.1342	0.224	0.6492	0.1584	0.483	FS
16413	16413	19348	19348	4023297	0.1377	0.0105	0.3848	0.1067	0.2288	0.6742	0.1449	0.482	FS
16517	16517	19181	19181	4023297	0.1536	0.0109	0.4399	0.1192	0.2613	0.5793	0.2515	0.5411	FS
16517	16517	19348	19348	4023297	0.1311	0.0137	0.3612	0.1375	0.2034	0.6162	0.1335	0.4416	FS
16548	16548	19348	19348	4023297	0.0933	0.025	0.2707	0.2108	0.0529	0.167	0.0235	0.107	3rd
16671	16671	18818	18818	4023297	0.1513	0.0272	0.3745	0.2209	0.1577	0.097	0.0315	0.08	4th
16743	16743	18778	18778	4023297	0.1472	0.0555	0.2952	0.2024	0.0194	0.1031	0.014	0.0656	4th
16743	16743	18974	18974	4023297	0.1565	0.04	0.3355	0.1457	0.0734	0.2069	0.0216	0.125	3rd
16409	16409	1X4777	1X4777	4023297	0.1173	0.0056	0.3468	0.0521	0.1786	0.372	0.0171	0.2031	2nd
16413	16413	1X2124	1X2124	4023297	0.1324	0.0228	0.3247	0.2269	0.1391	0.6151	0	0.3076	2nd
16413	16413	1X3656	1X3656	4023297	0.1179	0.0252	0.3	0.252	0.1297	0.5333	0	0.2667	2nd
16413	16413	1X4179	1X4179	4023297	0.1066	0.0451	0.2623	0.4264	0.0271	0.1001	0	0.05	4th
16413	16413	25354	25354	4023297	0.1209	0.0238	0.3264	0.226	0.1369	0.3596	0.0736	0.2533	2nd
16420	16420	1X2816	1X2816	4023297	0.1043	0.0171	0.2934	0.161	0.0906	0.256	0.0112	0.1392	3rd
16436	16436	1X4777	1X4777	4023297	0.0982	0.0257	0.2337	0.2056	0.0786	0.0181	0	0.0091	UN
16510	16510	1X4179	1X4179	4023297	0.117	0.0034	0.3402	0.0319	0.2025	0.4534	0.0352	0.2619	2nd
16517	16517	1X2124	1X2124	4023297	0.1288	0.0255	0.3143	0.2544	0.1205	0.5456	0	0.2728	2nd
16517	16517	1X3656	1X3656	4023297	0.1198	0.0231	0.3075	0.2346	0.141	0.5958	0	0.2979	2nd
16517	16517	1X4179	1X4179	4023297	0.1083	0.0423	0.2689	0.405	0.0403	0.1406	0	0.0703	4th
16517	16517	25354	25354	4023297	0.1138	0.0211	0.3028	0.2021	0.1349	0.4361	0.0255	0.2435	2nd
16524	16524	1X3697	1X3697	4023297	0.1219	0.0043	0.3674	0.0396	0.1885	0.5061	0.0159	0.2689	2nd
16527	16527	1X2231	1X2231	4023297	0.1084	0.0231	0.2925	0.2101	0.0989	0.2802	0.0143	0.1544	3rd
16527	16527	1X4777	1X4777	4023297	0.1225	0.0039	0.3356	0.0383	0.208	0.3889	0.023	0.2175	2nd
16550	16550	1X2816	1X2816	4023297	0.1126	0.0174	0.3162	0.161	0.1163	0.6659	0.0795	0.4125	2nd

FID1	ID1	FID2	ID2	N_SNP	HetHet	IBS0	HetConc	HomIBS0	Kinship	IBD1Seg	IBD2Seg	PropIBD	InfType
16562	16562	1X3697	1X3697	4023297	0.1033	0.0105	0.3017	0.097	0.0967	0.244	0	0.122	3rd
16671	16671	1X2816	1X2816	4023297	0.1106	0.0211	0.2645	0.19	0.1289	0.2611	0.0126	0.1431	3rd
16692	16692	1X2816	1X2816	4023297	0.1161	0.0109	0.3221	0.108	0.1601	0.4217	0.0637	0.2746	2nd
16702	16702	1X2816	1X2816	4023297	0.1307	0.0179	0.3585	0.1824	0.1687	0.5688	0.1298	0.4142	FS
16702	16702	1X4080	1X4080	4023297	0.1049	0.0381	0.2691	0.3395	0.0259	0.3103	0	0.1551	3rd
16708	16708	1X3697	1X3697	4023297	0.0989	0.0087	0.3014	0.0752	0.0764	0.4289	0	0.2144	2nd
16743	16743	25355	25355	4023297	0.141	0.0489	0.2722	0.1777	0.0402	0.0582	0.0127	0.0418	UN
16761	16761	1X3837	1X3837	4023297	0.1796	0.0151	0.3288	0.0947	0.0646	0.3064	0	0.1532	3rd
16413	16413	26988	26988	4023297	0.132	0.0203	0.3595	0.201	0.1772	0.5939	0.1022	0.3992	FS
16413	16413	27503	27503	4023297	0.1179	0.0219	0.3102	0.2075	0.1417	0.5975	0.0206	0.3194	2nd
16517	16517	26988	26988	4023297	0.1609	0.0081	0.4783	0.091	0.288	0.5311	0.3234	0.5889	FS
16517	16517	27503	27503	4023297	0.1249	0.0149	0.3365	0.1477	0.1865	0.6348	0.0698	0.3872	2nd
16524	16524	27306	27306	4023297	0.0727	0.0302	0.2532	0.2182	0.0287	0.0333	0.0813	0.0979	3rd
16548	16548	27503	27503	4023297	0.0938	0.028	0.2704	0.2361	0.0357	0.1163	0.0158	0.074	4th
16702	16702	27472	27472	4023297	0.1002	0.0166	0.3533	0.1385	0.0921	0.4082	0.0705	0.2746	2nd
16413	16413	28246	28246	4023297	0.1354	0.0195	0.3604	0.2051	0.1866	0.6133	0.0879	0.3946	FS
16420	16420	28304	28304	4023297	0.0801	0.0333	0.2576	0.2589	0.0339	0.0519	0.014	0.04	UN
16517	16517	28246	28246	4023297	0.1501	0.0157	0.4177	0.1757	0.2306	0.5013	0.2213	0.472	FS
16524	16524	28183	28183	4023297	0.0879	0.0288	0.2899	0.2173	0.0463	0.2112	0.044	0.1496	3rd
16548	16548	28246	28246	4023297	0.0992	0.0291	0.28	0.2592	0.0272	0.1907	0.0053	0.1007	3rd
16550	16550	28304	28304	4023297	0.1097	0.0161	0.3785	0.136	0.1875	0.3227	0.1352	0.2965	2nd
16692	16692	28304	28304	4023297	0.0875	0.033	0.2738	0.2605	0.0343	0.0899	0.0094	0.0544	4th
16702	16702	28076	28076	4023297	0.0996	0.0284	0.2613	0.241	0.0716	0.087	0.0123	0.0558	4th
16702	16702	28304	28304	4023297	0.1082	0.0235	0.3406	0.201	0.1119	0.2502	0.0669	0.192	2nd
16538	16538	28438	28438	4023297	0.0663	0.0196	0.319	0.1382	0.0346	0.0253	0.1421	0.1547	3rd
16550	16550	28576	28576	4023297	0.0756	0.0139	0.3049	0.1063	0.0229	0.4568	0.0821	0.3105	2nd
16562	16562	28417	28417	4023297	0.0716	0.0231	0.2584	0.1757	0.0719	0.0188	0.0441	0.0535	4th
16741	16741	28635	28635	4023297	0.0907	0.0205	0.2545	0.1572	0.0404	0.2993	0.0303	0.1799	2nd
16748	16748	28635	28635	4023297	0.0886	0.0296	0.2301	0.2067	0.0192	0.2034	0.0528	0.1545	3rd
16517	16517	30961	30961	4023297	0.1047	0.0368	0.2701	0.35	0.052	0.121	0	0.0605	4th
16409	16409	31297	31297	4023297	0.0981	0.0092	0.2647	0.0807	0.0913	0.132	0	0.066	4th
16550	16550	31462	31462	4023297	0.0939	0.0124	0.2409	0.1044	0.0763	0.0282	0	0.0141	UN
16692	16692	31409	31409	4023297	0.0876	0.0219	0.2339	0.1792	0.0574	0	0	0	UN
16409	16409	31882	31882	4023297	0.0802	0.024	0.2435	0.1913	0.0441	0.0016	0	0.0008	UN
16671	16671	31786	31786	4023297	0.1119	0.0452	0.2575	0.3534	0.0236	0.0025	0	0.0013	UN
16692	16692	31880	31880	4023297	0.0893	0.0219	0.2294	0.1776	0.0423	0.0044	0	0.0022	UN
16743	16743	31903	31903	4023297	0.1614	0.0211	0.291	0.099	0.1562	0.0242	0	0.0121	UN
16744	16744	31716	31716	4023297	0.122	0.0484	0.2523	0.3692	0.034	0.0014	0	0.0007	UN
16413	16413	32043	32043	4023297	0.1195	0.0246	0.2831	0.2424	0.1053	0.4899	0	0.245	2nd
16517	16517	32043	32043	4023297	0.1193	0.0293	0.2834	0.2882	0.0853	0.4616	0	0.2308	2nd
16744	16744	32224	32224	4023297	0.1272	0.041	0.2525	0.3351	0.0461	0.024	0	0.012	UN
16409	16409	32909	32909	4023297	0.081	0.024	0.2415	0.1895	0.0371	0	0.0013	0.0013	UN
16413	16413	32849	32849	4023297	0.1205	0.0244	0.2722	0.2392	0.0865	0.5123	0	0.2561	2nd
16517	16517	32849	32849	4023297	0.1201	0.0242	0.2719	0.2391	0.0857	0.5322	0	0.2661	2nd
16692	16692	33097	33097	4023297	0.0814	0.0279	0.222	0.2165	0.0312	0	0	0	UN
16400	16400	7158	7158	4023297	0.146	0.0264	0.2931	0.129	0.1072	0.2158	0.0048	0.1127	3rd
16413	16413	33863	33863	4023297	0.1222	0.0244	0.2921	0.2393	0.1124	0.5723	0	0.2862	2nd
16413	16413	34857	34857	4023297	0.1123	0.0371	0.2463	0.3572	0.0157	0.2804	0	0.1402	3rd
16420	16420	8170	8170	4023297	0.1266	0.0049	0.3755	0.0492	0.2056	0.4292	0.0219	0.2365	2nd
16499	16499	8344	8344	4023297	0.1549	0.012	0.3194	0.0657	0.0924	0.5335	0	0.2667	2nd
16517	16517	33863	33863	4023297	0.1216	0.0242	0.2914	0.24	0.111	0.5301	0	0.265	2nd
16517	16517	34857	34857	4023297	0.1125	0.0374	0.2477	0.3623	0.0132	0.2656	0	0.1328	3rd
16550	16550	7311	7311	4023297	0.1051	0.03	0.2895	0.2623	0.0369	0.527	0.0515	0.315	2nd
16550	16550	8170	8170	4023297	0.1401	0.0004	0.4218	0.0044	0.2625	0.718	0.2548	0.6138	PO
16671	16671	6716	6716	4023297	0.1897	0.0058	0.3885	0.053	0.1952	0.3708	0.0089	0.1943	2nd
16671	16671	6955	6955	4023297	0.0996	0.0409	0.2233	0.34	0.0172	0.1299	0	0.065	4th
16671	16671	7311	7311	4023297	0.0971	0.0334	0.2251	0.2812	0.0572	0.1742	0	0.0871	4th
16671	16671	8170	8170	4023297	0.0953	0.043	0.218	0.3603	0.0135	0.1373	0	0.0687	4th
16671	16671	8307	8307	4023297	0.0956	0.0446	0.2211	0.3661	0.0118	0.1076	0	0.0538	4th
16692	16692	7311	7311	4023297	0.1069	0.0287	0.2897	0.2637	0.0549	0.3123	0.0226	0.1787	2nd
16692	16692	8170	8170	4023297	0.1266	0.0023	0.3581	0.0234	0.2212	0.5528	0.091	0.3674	FS
16692	16692	8307	8307	4023297	0.1056	0.0301	0.2857	0.2753	0.0459	0.312	0.0282	0.1842	2nd
16702	16702	6265	6265	4023297	0.1088	0.039	0.2723	0.3576	0.0154	0.3067	0	0.1533	3rd
16702	16702	6955	6955	4023297	0.1125	0.0345	0.2818	0.3193	0.0394	0.3927	0	0.1963	2nd
16702	16702	7267	7267	4023297	0.1098	0.0366	0.2726	0.3369	0.0232	0.3733	0	0.1867	2nd
16702	16702	7311	7311	4023297	0.1189	0.0252	0.3163	0.2426	0.1117	0.4904	0.0562	0.3013	2nd
16702	16702	7625	7625	4023297	0.1124	0.035	0.2828	0.324	0.0389	0.4028	0	0.2014	2nd
16702	16702	8170	8170	4023297	0.1606	0.0006	0.4749	0.0072	0.3046	0.6616	0.3004	0.6312	PO
16702	16702	8307	8307	4023297	0.1188	0.0282	0.3165	0.2692	0.0993	0.4231	0.0697	0.2813	2nd
16702	16702	8395	8395	4023297	0.1084	0.0391	0.2697	0.3572	0.0119	0.3045	0	0.1522	3rd
16731	16731	6716	6716	4023297	0.1305	0.01	0.2655	0.0655	0.0178	0.3695	0.0022	0.187	2nd
16744	16744	33631	33631	4023297	0.1173	0.0478	0.2507	0.3639	0.0288	0	0	0	UN
16413	16413	9841	9841	4023297	0.1405	0.0001	0.3737	0.0006	0.268	0.9234	0.0743	0.536	PO
16424	16424	9656	9656	4023297	0.1672	0.0254	0.3011	0.1032	0.0955	0.194	0	0.097	3rd
16517	16517	9841	9841	4023297	0.1362	0.0157	0.3597	0.1712	0.1974	0.5999	0.1008	0.4008	FS
16527	16527	9562	9562	4023297	0.1052	0.0355	0.2703	0.3226	0.0182	0.1422	0	0.0711	4th
16702	16702	9045	9045	4023297	0.114	0.0274	0.3007	0.2583	0.0938	0.4551	0.0287	0.2563	2nd
16702	16702	9128	9128	4023297	0.1123	0.031	0.2826	0.2907	0.0562	0.4464	0	0.2232	2nd
16719	16719	9128	9128	4023297	0.0913	0.0228	0.2316	0.173	0.0226	0.2449	0	0.1225	3rd
16741	16741	8653	8653	4023297	0.0857	0.0324	0.2708	0.2454	0.0238	0.201	0.0434	0.1439	3rd
16743	16743	9656	9656	4023297	0.2197	0.0048	0.4011	0.0205	0.2501	0.6028	0.1248	0.4262	FS
16775	16775	17112	17112	4023297	0.0892	0.0332	0.2755	0.2692	0.03	0.077	0.0159	0.0544	4th
16780	16780	17295	17295	4023297	0.1679	0.0457	0.3109	0.3239	0.0744	0	0	0	UN
16862	16862	17254	17254	4023297	0.1451	0.0573	0.2814	0.1999	0.0434	0.3465	0.0699	0.2432	2nd
16862	16862	17833	17833	4023297	0.149	0.0394	0.2787	0.147	0.0915	0.1551	0.0497	0.1272	3rd
16780	16780	18528	18528	4023297	0.1551	0.0518	0.2932	0.3501	0.0545	0	0	0	UN
17139	17139	18019	18019	4023297	0.1436	0.0423	0.3153	0.2341	0.0532	0.0439	0.0093	0.0312	UN
17141	17141	18019	18019	4023297	0.1421	0.0556	0.2979	0.2812	0.0186	0.0676	0.0125	0.0463	4th
16775	16775	19674	19674	4023297	0.0959	0.0317	0.297	0.2567	0.0606	0.1117	0.0064	0.0623	4th
16815	16815	18866	18866	4023297	0.173	0.0287	0.3541	0.2455	0.153	0.1037	0	0.0519	4th
16815	16815	1X3796	1X3796	4023297	0.2065	0.0123	0.3577	0.102	0.1614	0.023	0	0.0115	UN
16816	16816	1X3796	1X3796	4023297	0.1984	0.006	0.3729	0.0352	0.1514	0.5966	0	0.2983	2nd
16827	16827	1X3697	1X3697	4023297	0.1148	0.0079	0.3276	0.0768	0.1551	0.2491	0	0.1246	3rd
16853	16853	1X3796	1X3796	4023297	0.1931	0.0599	0.3012	0.3521	0.023	0.0185	0	0.0092	UN
16853	16853	1X4209	1X4209	4023297	0.279	0.0001	0.4972	0.0007	0.3039	0.7126	0.2816	0.6379	PO
16853	16853	25409	25409	4023297	0.1608	0.0402	0.3097	0.2199	0.094	0.1727	0.0105	0.0969	3rd
16879	16879	1X2816	1X2816	4023297	0.0993	0.0344	0.2545	0.2572	0.0234	0.3381	0.0049	0.1739	3rd
16880	16880	1X4777	1X4777	4023297	0.1001	0.013	0.2919	0.1157	0.0826	0.153	0	0.0765	4th

TableS9_KING_SNRPC

FID1	ID1	FID2	ID2	N_SNP	HetHet	IBS0	HetConc	HomIBS0	Kinship	IBD1Seg	IBD2Seg	PropIBD	InfType
16890	16890	1X4209	1X4209	4023297	0.2037	0.0053	0.3767	0.0326	0.1612	0.6272	0.0027	0.3163	2nd
16986	16986	1X4179	1X4179	4023297	0.1196	0.0052	0.3466	0.0516	0.2006	0.3687	0.0097	0.194	2nd
17159	17159	1X2816	1X2816	4023297	0.1004	0.0302	0.2484	0.2499	0.0575	0.2551	0.0043	0.1318	3rd
16853	16853	26498	26498	4023297	0.1911	0.05	0.3109	0.3544	0.0671	0.0012	0	0.0006	UN
16861	16861	26318	26318	4023297	0.1044	0.0306	0.2783	0.2633	0.0866	0.0044	0	0.0022	UN
16827	16827	28183	28183	4023297	0.0819	0.0272	0.2553	0.2116	0.0518	0.1029	0.0103	0.0617	4th
16815	16815	30917	30917	4023297	0.138	0.0433	0.2592	0.4235	0.0422	0	0	0	UN
16815	16815	30920	30920	4023297	0.1517	0.0419	0.2653	0.3939	0.0261	0	0	0	UN
16780	16780	31065	31065	4023297	0.1552	0.0537	0.2914	0.3835	0.0459	0	0	0	UN
16815	16815	31789	31789	4023297	0.1291	0.0301	0.2734	0.1911	0.1024	0	0	0	UN
16816	16816	32089	32089	4023297	0.1353	0.0196	0.2784	0.0965	0.0818	0.1503	0	0.0752	4th
16815	16815	33908	33908	4023297	0.1229	0.038	0.2646	0.3192	0.0551	0.056	0	0.028	UN
16816	16816	34568	34568	4023297	0.134	0.0179	0.2735	0.0908	0.0836	0.1727	0	0.0864	4th
16847	16847	7158	7158	4023297	0.1178	0.0156	0.2679	0.0633	0.0306	0.5092	0.0388	0.2934	2nd
16866	16866	8344	8344	4023297	0.1556	0.019	0.2926	0.0915	0.0957	0.3707	0	0.1854	2nd
16879	16879	6716	6716	4023297	0.1427	0.0094	0.2877	0.071	0.0649	0.385	0.0027	0.1952	2nd
17111	17111	7158	7158	4023297	0.1492	0.021	0.3104	0.0976	0.125	0.2728	0.0705	0.2069	2nd
17139	17139	8344	8344	4023297	0.1531	0.0156	0.2922	0.0893	0.0977	0.237	0.0133	0.1318	3rd
17141	17141	8344	8344	4023297	0.1909	0.01	0.3771	0.0546	0.193	0.4953	0.0519	0.2995	2nd
17159	17159	6716	6716	4023297	0.1776	0.01	0.3728	0.0845	0.147	0.3817	0.0022	0.1931	2nd
16775	16775	8780	8780	4023297	0.1395	0.0027	0.3958	0.0292	0.243	0.4777	0.0921	0.3309	2nd
16780	16780	9656	9656	4023297	0.1538	0.0249	0.2596	0.1177	0.0873	0.1064	0	0.0532	4th
16829	16829	8653	8653	4023297	0.0898	0.0302	0.306	0.2277	0.0226	0.3023	0.0346	0.1857	2nd
16853	16853	8465	8465	4023297	0.1649	0.0682	0.286	0.4177	0.025	0.2184	0	0.1092	3rd
16862	16862	9656	9656	4023297	0.199	0.0022	0.3594	0.0085	0.2269	0.8394	0.1263	0.546	PO
17112	17112	8780	8780	4023297	0.1162	0.0059	0.3288	0.0571	0.1663	0.4588	0.0229	0.2523	2nd
17254	17254	17833	17833	4023297	0.1497	0.0637	0.2824	0.2265	0.0165	0.1229	0.0452	0.1067	3rd
17295	17295	17826	17826	4023297	0.1575	0.0457	0.3	0.2638	0.0427	0.0118	0	0.0059	UN
17295	17295	17833	17833	4023297	0.1716	0.0607	0.3057	0.2978	0.0497	0	0	0	UN
17199	17199	17903	17903	4023297	0.1184	0.0215	0.331	0.1995	0.1529	0.5697	0.1045	0.3894	FS
17199	17199	18385	18385	4023297	0.1214	0.0166	0.3519	0.1561	0.1771	0.6252	0.1521	0.4647	FS
17199	17199	18599	18599	4023297	0.1022	0.027	0.2972	0.236	0.0717	0.1738	0.0076	0.0945	3rd
17295	17295	18528	18528	4023297	0.2249	0.0245	0.4367	0.2054	0.2289	0.0665	0	0.0332	UN
17826	17826	18528	18528	4023297	0.1522	0.0473	0.3005	0.2662	0.0481	0.0097	0	0.0049	UN
17891	17891	17998	17998	4023297	0.1014	0.0338	0.2715	0.3096	0.0692	0.0012	0	0.0006	UN
17199	17199	19181	19181	4023297	0.1257	0.0178	0.3427	0.1742	0.1774	0.6076	0.0896	0.3934	FS
17199	17199	19348	19348	4023297	0.1388	0.0057	0.4025	0.059	0.2633	0.6927	0.203	0.5493	FS
17254	17254	18778	18778	4023297	0.1449	0.0428	0.3008	0.1522	0.0742	0.2279	0.0027	0.1166	3rd
17295	17295	18866	18866	4023297	0.1549	0.0563	0.2677	0.4347	0.0386	0	0	0	UN
17295	17295	18929	18929	4023297	0.1478	0.0564	0.2564	0.4502	0.0223	0	0	0	UN
17325	17325	19674	19674	4023297	0.0818	0.0312	0.2662	0.2438	0.034	0.0508	0.004	0.0294	UN
17829	17829	19237	19237	4023297	0.1524	0.0526	0.3007	0.2625	0.0439	0.0176	0.011	0.0198	UN
17898	17898	19237	19237	4023297	0.1419	0.0368	0.3148	0.1968	0.0314	0.0719	0.0589	0.0948	3rd
17184	17184	1X2816	1X2816	4023297	0.0972	0.0258	0.2505	0.2174	0.0535	0.2542	0.0013	0.1285	3rd
17199	17199	1X2124	1X2124	4023297	0.1233	0.0301	0.3044	0.2856	0.0844	0.5338	0	0.2669	2nd
17199	17199	1X3656	1X3656	4023297	0.1148	0.0278	0.2988	0.2682	0.1065	0.5561	0	0.278	2nd
17199	17199	25354	25354	4023297	0.1141	0.0215	0.3125	0.1969	0.1451	0.4348	0.0521	0.2695	2nd
17222	17222	1X2816	1X2816	4023297	0.1148	0.0217	0.3023	0.2027	0.1174	0.3016	0.0077	0.1585	3rd
17233	17233	1X3697	1X3697	4023297	0.1122	0.0085	0.3382	0.0796	0.1326	0.3927	0	0.1964	2nd
17254	17254	25355	25355	4023297	0.1563	0.0376	0.3232	0.1384	0.1164	0.2037	0.0608	0.1627	3rd
17255	17255	1X4209	1X4209	4023297	0.2248	0.0035	0.4359	0.0222	0.2074	0.737	0	0.3685	2nd
17295	17295	1X3796	1X3796	4023297	0.2033	0.0459	0.3116	0.3659	0.0858	0	0	0	UN
17295	17295	1X3837	1X3837	4023297	0.202	0.0467	0.3059	0.3783	0.0777	0	0	0	UN
17295	17295	1X4209	1X4209	4023297	0.2033	0.0456	0.3087	0.3732	0.0825	0.0014	0	0.0007	UN
17840	17840	1X3796	1X3796	4023297	0.1802	0.0044	0.3397	0.0269	0.1118	0.6362	0.0021	0.3202	2nd
17852	17852	1X2816	1X2816	4023297	0.1001	0.0261	0.2758	0.2164	0.0374	0.3216	0.0015	0.1623	3rd
17871	17871	1X3697	1X3697	4023297	0.106	0.0064	0.3095	0.0573	0.1301	0.3691	0	0.1845	2nd
17891	17891	1X3697	1X3697	4023297	0.1084	0.0367	0.2715	0.3522	0.0369	0.0265	0	0.0132	UN
17891	17891	1X3822	1X3822	4023297	0.1123	0.034	0.2803	0.3236	0.0513	0.0469	0	0.0235	UN
17199	17199	26988	26988	4023297	0.1348	0.012	0.3828	0.1217	0.2257	0.5939	0.1803	0.4772	FS
17199	17199	27503	27503	4023297	0.1139	0.0268	0.3061	0.2456	0.1226	0.5215	0.0362	0.2969	2nd
17255	17255	26498	26498	4023297	0.1608	0.0253	0.2947	0.1583	0.0339	0.0042	0	0.0021	UN
17295	17295	26498	26498	4023297	0.1833	0.0364	0.2846	0.385	0.1032	0	0	0	UN
17340	17340	27503	27503	4023297	0.0909	0.0274	0.2717	0.2258	0.0139	0.1456	0.0083	0.0811	4th
17199	17199	28246	28246	4023297	0.1335	0.0162	0.3653	0.1699	0.1935	0.6106	0.1239	0.4292	FS
17222	17222	28076	28076	4023297	0.1129	0.0364	0.3074	0.2906	0.0656	0.0527	0.0053	0.0317	UN
17829	17829	28281	28281	4023297	0.1295	0.0545	0.2668	0.2423	0.0299	0.0081	0.0194	0.0235	UN
17871	17871	28183	28183	4023297	0.0775	0.0285	0.2512	0.214	0.0128	0.1201	0.0428	0.1029	3rd
17891	17891	28635	28635	4023297	0.0958	0.0282	0.2405	0.2266	0.0603	0.07	0	0.035	UN
17295	17295	30609	30609	4023297	0.1634	0.0359	0.296	0.2843	0.1008	0.004	0	0.002	UN
17295	17295	30917	30917	4023297	0.1519	0.0452	0.2573	0.4534	0.0708	0	0	0	UN
17295	17295	30920	30920	4023297	0.1804	0.0409	0.2933	0.3952	0.1084	0	0	0	UN
17295	17295	30933	30933	4023297	0.1502	0.0592	0.2544	0.4612	0.0282	0	0	0	UN
17295	17295	31011	31011	4023297	0.1503	0.0564	0.258	0.4607	0.0315	0	0	0	UN
17295	17295	31028	31028	4023297	0.1514	0.0482	0.2714	0.3381	0.0421	0	0	0	UN
17295	17295	31065	31065	4023297	0.1684	0.0497	0.2928	0.3876	0.0814	0.0016	0	0.0008	UN
17891	17891	31016	31016	4023297	0.0996	0.0368	0.2657	0.3258	0.0533	0	0	0	UN
17795	17795	31311	31311	4023297	0.1072	0.0429	0.2461	0.2583	0.0264	0	0	0	UN
17295	17295	31560	31560	4023297	0.151	0.0423	0.2775	0.3255	0.0515	0.0058	0	0.0029	UN
17295	17295	31903	31903	4023297	0.1714	0.0521	0.2959	0.3411	0.0823	0.0012	0	0.0006	UN
17795	17795	31805	31805	4023297	0.1258	0.0301	0.2705	0.1897	0.0645	0.0091	0	0.0046	UN
17199	17199	32043	32043	4023297	0.1166	0.0225	0.2822	0.2173	0.1004	0.5956	0	0.2978	2nd
17199	17199	32849	32849	4023297	0.1189	0.0208	0.275	0.2	0.0902	0.6195	0	0.3098	2nd
17165	17165	7158	7158	4023297	0.1283	0.0254	0.2683	0.1051	0.0549	0.4072	0.016	0.2196	2nd
17184	17184	8170	8170	4023297	0.1074	0.011	0.2817	0.0967	0.1393	0.3349	0.0015	0.169	3rd
17199	17199	33863	33863	4023297	0.1143	0.0304	0.2758	0.2862	0.0641	0.4554	0	0.2277	2nd
17222	17222	7311	7311	4023297	0.0999	0.0364	0.2534	0.3123	0.0219	0.136	0.0023	0.0703	4th
17222	17222	8170	8170	4023297	0.1159	0.008	0.3031	0.0755	0.1756	0.2808	0.0085	0.1489	3rd
17232	17232	6716	6716	4023297	0.1593	0.0114	0.3247	0.0793	0.1005	0.4343	0	0.2171	2nd
17233	17233	8134	8134	4023297	0.0718	0.0282	0.2649	0.2087	0.0418	0.008	0.0303	0.0343	UN
17268	17268	7158	7158	4023297	0.1318	0.0342	0.2749	0.1475	0.0317	0.2799	0	0.1399	3rd
17295	17295	6716	6716	4023297	0.1734	0.0516	0.2785	0.433	0.0709	0.005	0	0.0025	UN
17295	17295	8344	8344	4023297	0.1749	0.0647	0.2846	0.4081	0.0425	0	0	0	UN
17792	17792	7158	7158	4023297	0.1215	0.0256	0.2655	0.1108	0.0154	0.3617	0.0373	0.2182	2nd
17795	17795	8344	8344	4023297	0.1551	0.0266	0.3001	0.1467	0.0567	0.1387	0	0.0693	4th
17829	17829	7158	7158	4023297	0.1627	0.02	0.3255	0.0949	0.1632	0.287	0.0176	0.1611	3rd
17879	17879	6716	6716	4023297	0.1312	0.013	0.2629	0.0821	0.014	0.3214	0	0.1607	3rd
17891	17891	7091	7091	4023297	0.1122	0.0316	0.2815	0.3049	0.0633	0.0522	0	0.0261	UN

FID1	ID1	FID2	ID2	N_SNP	HetHet	IBS0	HetConc	HomIBS0	Kinship	IBD1Seg	IBD2Seg	PropIBD	InfType
17199	17199	9841	9841	4023297	0.1409	0.0001	0.3874	0.0006	0.2696	0.8965	0.1007	0.5489	PO
17254	17254	9656	9656	4023297	0.2004	0.0028	0.3655	0.0111	0.2268	0.797	0.1022	0.5007	FS
17294	17294	8780	8780	4023297	0.1086	0.0076	0.3315	0.069	0.1151	0.4435	0.0033	0.2251	2nd
17295	17295	8465	8465	4023297	0.1602	0.0542	0.2655	0.4595	0.0674	0	0	0	UN
17295	17295	9656	9656	4023297	0.1854	0.0169	0.3008	0.0832	0.1735	0.0739	0	0.0369	UN
17295	17295	9860	9860	4023297	0.1751	0.0377	0.2959	0.3238	0.1285	0.0309	0	0.0154	UN
17325	17325	8780	8780	4023297	0.122	0.0078	0.3585	0.0785	0.1682	0.3061	0	0.1531	3rd
17826	17826	9656	9656	4023297	0.1647	0.0203	0.2963	0.0902	0.107	0.3361	0.0179	0.1859	2nd
17833	17833	9656	9656	4023297	0.2084	0.0109	0.3702	0.0455	0.2167	0.3896	0.0281	0.2229	2nd
17852	17852	9128	9128	4023297	0.0965	0.0196	0.2532	0.1588	0.0428	0.2354	0	0.1177	3rd
17871	17871	8653	8653	4023297	0.0795	0.028	0.2561	0.2095	0.0153	0.2283	0.0427	0.1569	3rd
17873	17873	9562	9562	4023297	0.0939	0.0133	0.2799	0.1134	0.0274	0.2587	0	0.1294	3rd
17903	17903	18385	18385	4023297	0.1273	0.0136	0.3844	0.1291	0.2124	0.5556	0.2342	0.512	FS
17903	17903	18599	18599	4023297	0.1105	0.0213	0.3374	0.1885	0.1296	0.2225	0.0127	0.124	3rd
17969	17969	17970	17970	4023297	0.1353	0.048	0.2745	0.3704	0.0409	0	0	0	UN
17969	17969	18019	18019	4023297	0.1369	0.0511	0.2783	0.32	0.0328	0	0	0	UN
17970	17970	18019	18019	4023297	0.1437	0.0502	0.279	0.3301	0.0654	0.0013	0	0.0007	UN
18341	18341	18469	18469	4023297	0.1329	0.0404	0.2823	0.2689	0.0627	0.0267	0.0018	0.0151	UN
18385	18385	18599	18599	4023297	0.0958	0.0285	0.2882	0.2378	0.0703	0.131	0.0185	0.084	4th
18469	18469	18586	18586	4023297	0.1332	0.0349	0.2766	0.2282	0.0888	0.0377	0.0017	0.0206	UN
17903	17903	19181	19181	4023297	0.1367	0.0128	0.3928	0.1266	0.22	0.664	0.1663	0.4983	FS
17903	17903	19348	19348	4023297	0.1184	0.0204	0.3313	0.1891	0.1577	0.5553	0.1113	0.3889	FS
17970	17970	18929	18929	4023297	0.1305	0.0543	0.2413	0.4492	0.0232	0	0	0	UN
17999	17999	19173	19173	4023297	0.0914	0.0329	0.3138	0.2617	0.0664	0.0385	0.0323	0.0515	4th
18341	18341	18818	18818	4023297	0.1377	0.0389	0.3135	0.2835	0.1	0.0051	0.0044	0.0069	UN
18385	18385	19181	19181	4023297	0.118	0.019	0.3306	0.1757	0.1493	0.639	0.0878	0.4072	FS
18385	18385	19348	19348	4023297	0.1382	0.0134	0.4217	0.1316	0.2293	0.5019	0.2856	0.5366	FS
18469	18469	18818	18818	4023297	0.1489	0.0315	0.3232	0.2297	0.1243	0.0576	0.0038	0.0326	UN
18469	18469	19012	19012	4023297	0.1693	0.0455	0.3785	0.3178	0.1144	0.1616	0.0138	0.0946	3rd
18586	18586	18818	18818	4023297	0.1288	0.0385	0.2805	0.2688	0.0842	0.0324	0.0035	0.0197	UN
18586	18586	19012	19012	4023297	0.1271	0.0405	0.2718	0.2724	0.0762	0.0302	0.0126	0.0277	UN
18599	18599	19181	19181	4023297	0.199	0.0001	0.7792	0.0015	0.4303	0.8872	0.1038	0.5475	PO
18599	18599	19348	19348	4023297	0.1019	0.0245	0.2965	0.2156	0.0836	0.1783	0.0136	0.1027	3rd
17903	17903	1X2124	1X2124	4023297	0.1207	0.0324	0.3019	0.2983	0.0636	0.5354	0	0.2677	2nd
17903	17903	1X3656	1X3656	4023297	0.109	0.0373	0.2852	0.3423	0.0489	0.4179	0	0.2089	2nd
17903	17903	25354	25354	4023297	0.1156	0.0164	0.325	0.1531	0.1736	0.4817	0.0818	0.3227	2nd
17970	17970	1X3837	1X3837	4023297	0.1858	0.0422	0.2978	0.357	0.0389	0.039	0	0.0195	UN
17998	17998	1X3697	1X3697	4023297	0.1147	0.0305	0.2903	0.3149	0.0779	0.0743	0	0.0372	UN
17998	17998	1X3822	1X3822	4023297	0.1143	0.0309	0.2852	0.313	0.0701	0.0781	0	0.0391	UN
17999	17999	1X2231	1X2231	4023297	0.0978	0.0285	0.2782	0.2437	0.0192	0.223	0.0043	0.1158	3rd
17999	17999	1X4777	1X4777	4023297	0.1008	0.0246	0.2825	0.2155	0.0365	0.1625	0.0083	0.0895	3rd
18019	18019	1X3837	1X3837	4023297	0.1858	0.0474	0.2976	0.3142	0.0238	0.0729	0	0.0365	UN
18024	18024	1X2231	1X2231	4023297	0.0994	0.0318	0.274	0.2756	0.0219	0.1207	0.0097	0.0701	4th
18144	18144	1X3656	1X3656	4023297	0.1014	0.0383	0.2599	0.3566	0.029	0.0522	0	0.0261	UN
18144	18144	1X4179	1X4179	4023297	0.1182	0.0064	0.315	0.0642	0.1989	0.1778	0.0016	0.0905	3rd
18341	18341	1X2816	1X2816	4023297	0.1052	0.04	0.2363	0.335	0.028	0.1239	0.0036	0.0656	4th
18385	18385	1X2124	1X2124	4023297	0.114	0.0385	0.2875	0.3399	0.0134	0.4525	0	0.2263	2nd
18385	18385	1X3656	1X3656	4023297	0.1055	0.0376	0.2807	0.3356	0.031	0.4305	0.0016	0.2169	2nd
18385	18385	25354	25354	4023297	0.111	0.0238	0.3166	0.2124	0.127	0.4243	0.0898	0.302	2nd
18469	18469	1X2816	1X2816	4023297	0.1243	0.0382	0.2709	0.3169	0.04	0.1475	0.0539	0.1276	3rd
18523	18523	26198	26198	4023297	0.0873	0.034	0.3012	0.275	0.0468	0.0193	0.0036	0.0133	UN
18528	18528	1X3796	1X3796	4023297	0.1903	0.0511	0.2966	0.385	0.0421	0	0	0	UN
18528	18528	1X3837	1X3837	4023297	0.1889	0.0507	0.2909	0.3888	0.0367	0	0	0	UN
18528	18528	1X4209	1X4209	4023297	0.1903	0.0504	0.2939	0.3889	0.04	0	0	0	UN
18557	18557	1X2816	1X2816	4023297	0.1016	0.0235	0.2671	0.1996	0.0715	0.3621	0.0016	0.1827	2nd
18586	18586	1X2816	1X2816	4023297	0.1169	0.0398	0.2631	0.3218	0.0404	0.1978	0.0156	0.1145	3rd
17903	17903	26988	26988	4023297	0.1445	0.0121	0.4315	0.1229	0.2453	0.5716	0.2597	0.5455	FS
17903	17903	27503	27503	4023297	0.1118	0.0222	0.3053	0.2043	0.1334	0.5343	0.0643	0.3315	2nd
17970	17970	26498	26498	4023297	0.167	0.0338	0.2748	0.3755	0.0624	0	0	0	UN
18385	18385	26988	26988	4023297	0.1209	0.0223	0.3468	0.2064	0.1468	0.5611	0.1389	0.4194	FS
18385	18385	27503	27503	4023297	0.1046	0.0246	0.2875	0.2164	0.1017	0.5379	0.036	0.3049	2nd
18523	18523	27503	27503	4023297	0.1131	0.0212	0.3563	0.1925	0.1126	0.2975	0.0154	0.1642	3rd
18528	18528	26498	26498	4023297	0.1725	0.0406	0.2734	0.399	0.0662	0	0	0	UN
18599	18599	26988	26988	4023297	0.1247	0.0206	0.3846	0.1949	0.1542	0.3247	0.0162	0.1786	2nd
18599	18599	27503	27503	4023297	0.095	0.0332	0.2694	0.2823	0.0214	0.0976	0.008	0.0568	4th
17903	17903	28246	28246	4023297	0.1498	0.0122	0.439	0.132	0.2434	0.5372	0.2705	0.539	FS
18385	18385	28246	28246	4023297	0.1394	0.0125	0.4076	0.1289	0.2185	0.6021	0.2185	0.5195	FS
18599	18599	28246	28246	4023297	0.1254	0.0148	0.3739	0.1483	0.1699	0.35	0.0131	0.1881	2nd
17998	17998	28635	28635	4023297	0.0984	0.02	0.247	0.1694	0.1019	0.1029	0	0.0515	4th
17970	17970	30917	30917	4023297	0.1376	0.0422	0.2493	0.4436	0.057	0	0	0	UN
17970	17970	30920	30920	4023297	0.1533	0.0417	0.2603	0.4185	0.0422	0	0	0	UN
18528	18528	30609	30609	4023297	0.1502	0.0446	0.2777	0.3306	0.0732	0.0027	0	0.0014	UN
18528	18528	30917	30917	4023297	0.1384	0.0517	0.2385	0.482	0.0471	0	0	0	UN
18528	18528	30920	30920	4023297	0.1677	0.0461	0.2778	0.4157	0.0667	0	0	0	UN
17903	17903	30961	30961	4023297	0.0979	0.04	0.2602	0.3553	0.032	0.1314	0	0.0657	4th
17969	17969	30933	30933	4023297	0.1473	0.0459	0.2884	0.3578	0.0432	0	0	0	UN
17970	17970	30933	30933	4023297	0.148	0.0493	0.2742	0.4033	0.0527	0.0016	0	0.0008	UN
17970	17970	31011	31011	4023297	0.1402	0.0483	0.2597	0.4157	0.0498	0	0	0	UN
17998	17998	31016	31016	4023297	0.0999	0.0376	0.2653	0.3466	0.0515	0	0	0	UN
18019	18019	30933	30933	4023297	0.1509	0.0508	0.2808	0.3336	0.053	0.0016	0	0.0008	UN
18019	18019	31011	31011	4023297	0.1386	0.0543	0.2556	0.3655	0.0295	0.0059	0	0.003	UN
18469	18469	31011	31011	4023297	0.133	0.0509	0.248	0.383	0.0232	0.0039	0	0.002	UN
18528	18528	30933	30933	4023297	0.1453	0.0628	0.2543	0.4652	0.027	0	0	0	UN
18528	18528	31065	31065	4023297	0.1648	0.0521	0.2971	0.3867	0.0821	0	0	0	UN
17970	17970	31337	31337	4023297	0.1394	0.0503	0.2611	0.4267	0.0473	0	0	0	UN
18469	18469	31364	31364	4023297	0.1357	0.0458	0.2736	0.3347	0.0664	0.0032	0	0.0016	UN
18469	18469	31408	31408	4023297	0.1353	0.0453	0.2626	0.312	0.0591	0.0032	0	0.0016	UN
18469	18469	31410	31410	4023297	0.1406	0.0454	0.2711	0.3076	0.0602	0	0	0	UN
18586	18586	31364	31364	4023297	0.1216	0.0497	0.249	0.3528	0.0233	0.0067	0	0.0034	UN
18586	18586	31408	31408	4023297	0.1329	0.0475	0.2677	0.3264	0.0336	0.0107	0	0.0054	UN
17970	17970	31497	31497	4023297	0.1461	0.0457	0.274	0.3805	0.0668	0.0012	0	0.0006	UN
17970	17970	31656	31656	4023297	0.1356	0.0546	0.256	0.4365	0.0347	0	0	0	UN
18528	18528	31560	31560	4023297	0.14	0.0485	0.2635	0.3548	0.0333	0.0016	0	0.0008	UN
17969	17969	31805	31805	4023297	0.1326	0.0496	0.2686	0.3938	0.0326	0	0	0	UN
17970	17970	31805	31805	4023297	0.1489	0.0415	0.2935	0.3555	0.0992	0	0	0	UN
18469	18469	31738	31738	4023297	0.1212	0.0463	0.2472	0.3252	0.0276	0.0035	0	0.0017	UN
18469	18469	31846	31846	4023297	0.1296	0.0432	0.2627	0.2881	0.0597	0.0014	0	0.0007	UN
18528	18528	31903	31903	4023297	0.1659	0.0533	0.2959	0.3357	0.0754	0	0	0	UN

FID1	ID1	FID2	ID2	N_SNP	HetHet	IBS0	HetConc	HomIBS0	Kinship	IBD1Seg	IBD2Seg	PropIBD	InfType
17903	17903	32043	32043	4023297	0.1091	0.0385	0.2644	0.3488	0.0112	0.3511	0	0.1755	3rd
17970	17970	32224	32224	4023297	0.128	0.0557	0.2397	0.473	0.0221	0	0	0	UN
18341	18341	32040	32040	4023297	0.1085	0.0445	0.2332	0.348	0.0317	0.0359	0	0.018	UN
18385	18385	32043	32043	4023297	0.1073	0.0291	0.2649	0.2624	0.0386	0.5056	0	0.2528	2nd
18469	18469	32187	32187	4023297	0.1437	0.0559	0.2742	0.3722	0.0256	0.0026	0	0.0013	UN
18469	18469	32358	32358	4023297	0.1377	0.0451	0.2698	0.3103	0.0657	0.0064	0	0.0032	UN
18586	18586	32358	32358	4023297	0.1235	0.0461	0.2455	0.3104	0.0249	0.0018	0	0.0009	UN
17903	17903	32849	32849	4023297	0.1101	0.0301	0.2539	0.2774	0.0261	0.4349	0	0.2175	2nd
18385	18385	32849	32849	4023297	0.1074	0.0254	0.2519	0.2301	0.0317	0.536	0	0.268	2nd
17903	17903	33863	33863	4023297	0.1105	0.0325	0.2693	0.2956	0.0413	0.4206	0	0.2103	2nd
17969	17969	8344	8344	4023297	0.1691	0.0179	0.3143	0.1182	0.1321	0.044	0	0.022	UN
17970	17970	8344	8344	4023297	0.1856	0.0091	0.3365	0.0635	0.1945	0.1838	0	0.0919	3rd
17981	17981	7937	7937	4023297	0.1179	0.0067	0.3435	0.0629	0.1926	0.3292	0.0136	0.1782	2nd
17998	17998	7091	7091	4023297	0.1201	0.0248	0.3057	0.2581	0.1102	0.1355	0	0.0677	4th
18019	18019	8344	8344	4023297	0.1897	0.0097	0.3461	0.0591	0.1989	0.3043	0.0165	0.1687	3rd
18022	18022	7937	7937	4023297	0.1086	0.008	0.33	0.0733	0.1522	0.3448	0.0014	0.1738	3rd
18341	18341	6716	6716	4023297	0.1989	0.0097	0.3972	0.0839	0.2018	0.1981	0.0049	0.104	3rd
18341	18341	7267	7267	4023297	0.1243	0.0137	0.2803	0.1204	0.1689	0.2136	0.0015	0.1083	3rd
18469	18469	6716	6716	4023297	0.2237	0.0041	0.44	0.0357	0.2634	0.4002	0.0388	0.239	2nd
18528	18528	6716	6716	4023297	0.1618	0.0565	0.2651	0.4473	0.029	0.0018	0	0.0009	UN
18529	18529	8344	8344	4023297	0.1474	0.0221	0.2836	0.1169	0.0556	0.1741	0	0.087	4th
18557	18557	6716	6716	4023297	0.1473	0.0086	0.3043	0.0723	0.0731	0.319	0.004	0.1635	3rd
18557	18557	7311	7311	4023297	0.1055	0.0107	0.2804	0.0923	0.1395	0.3779	0.0026	0.1915	2nd
18557	18557	8307	8307	4023297	0.0946	0.0331	0.2446	0.2666	0.0122	0.2035	0.0027	0.1044	3rd
18586	18586	6716	6716	4023297	0.1956	0.0064	0.3796	0.0535	0.2092	0.3413	0.0102	0.1808	2nd
18599	18599	33863	33863	4023297	0.1083	0.0099	0.2831	0.0919	0.1154	0.1221	0	0.0611	4th
17903	17903	9841	9841	4023297	0.1228	0.016	0.3283	0.1596	0.1635	0.6382	0.0649	0.384	2nd
17916	17916	9562	9562	4023297	0.0966	0.0125	0.2848	0.1074	0.0497	0.2566	0	0.1283	3rd
17970	17970	8465	8465	4023297	0.1447	0.0512	0.2557	0.4518	0.0244	0	0	0	UN
17971	17971	9562	9562	4023297	0.1097	0.0097	0.3417	0.0874	0.1027	0.4465	0	0.2233	2nd
17997	17997	9562	9562	4023297	0.0929	0.0123	0.2702	0.1053	0.0411	0.2045	0	0.1022	3rd
17998	17998	8653	8653	4023297	0.0923	0.0347	0.2568	0.2904	0.0241	0.0551	0	0.0276	UN
17999	17999	9562	9562	4023297	0.1106	0.0071	0.3124	0.0659	0.1447	0.263	0.0013	0.1328	3rd
18015	18015	9656	9656	4023297	0.1459	0.0231	0.2856	0.0862	0.0168	0.3667	0	0.1833	2nd
18024	18024	9562	9562	4023297	0.1132	0.0114	0.3107	0.107	0.1368	0.1874	0.0014	0.0951	3rd
18141	18141	9562	9562	4023297	0.1147	0.008	0.3383	0.0732	0.1449	0.3433	0	0.1716	3rd
18385	18385	9841	9841	4023297	0.1339	0	0.3794	0.0005	0.2557	0.8893	0.1089	0.5536	PO
18469	18469	8465	8465	4023297	0.146	0.0435	0.2638	0.3434	0.0432	0.1223	0.0019	0.063	4th
18528	18528	8465	8465	4023297	0.1496	0.0585	0.2537	0.4675	0.0291	0	0	0	UN
18528	18528	9656	9656	4023297	0.176	0.0258	0.2924	0.1234	0.1305	0.0281	0	0.0141	UN
18528	18528	9860	9860	4023297	0.1633	0.0413	0.2819	0.3345	0.094	0.0154	0	0.0077	UN
18586	18586	8465	8465	4023297	0.138	0.0391	0.2555	0.3025	0.0295	0.1005	0	0.0503	4th
18599	18599	9841	9841	4023297	0.1094	0.0266	0.3065	0.2507	0.0658	0.163	0.0013	0.0828	4th
18818	18818	19012	19012	4023297	0.1277	0.0405	0.2763	0.2899	0.0741	0.0205	0.0138	0.024	UN
18866	18866	18929	18929	4023297	0.141	0.054	0.2548	0.4456	0.0414	0	0	0	UN
19181	19181	19348	19348	4023297	0.1258	0.0153	0.3434	0.1508	0.1879	0.6423	0.0896	0.4108	FS
18778	18778	25355	25355	4023297	0.1429	0.0469	0.3062	0.165	0.0713	0.0547	0.0066	0.0339	UN
18818	18818	1X2816	1X2816	4023297	0.1169	0.0215	0.2663	0.2044	0.1144	0.2313	0.0184	0.134	3rd
18866	18866	1X3796	1X3796	4023297	0.2376	0.0058	0.4042	0.0514	0.2346	0.2299	0	0.115	3rd
18866	18866	1X3837	1X3837	4023297	0.1848	0.0478	0.2854	0.3984	0.0352	0.0013	0	0.0006	UN
18866	18866	1X4209	1X4209	4023297	0.1874	0.046	0.2909	0.3874	0.0447	0.0012	0	0.0006	UN
18917	18917	1X2816	1X2816	4023297	0.0951	0.0191	0.254	0.1555	0.0661	0.3789	0.0058	0.1952	2nd
18929	18929	1X3796	1X3796	4023297	0.1785	0.0465	0.2799	0.3972	0.0291	0.0028	0	0.0014	UN
18929	18929	1X3837	1X3837	4023297	0.1799	0.0459	0.2797	0.3989	0.0278	0.0027	0	0.0013	UN
18929	18929	1X4209	1X4209	4023297	0.1778	0.0457	0.2759	0.4004	0.026	0.0054	0	0.0027	UN
18929	18929	26196	26196	4023297	0.1692	0.0264	0.3576	0.2481	0.1583	0.3896	0.0901	0.2849	2nd
18938	18938	1X2231	1X2231	4023297	0.1096	0.0283	0.2717	0.2485	0.1009	0.1697	0.0074	0.0923	3rd
19012	19012	1X2816	1X2816	4023297	0.1198	0.0298	0.2702	0.2551	0.0818	0.2196	0.049	0.1589	3rd
19144	19144	1X3837	1X3837	4023297	0.1909	0.0294	0.3249	0.1876	0.0684	0.1668	0	0.0834	4th
19173	19173	1X4777	1X4777	4023297	0.1008	0.0279	0.2823	0.2449	0.0197	0.1249	0	0.0624	4th
19181	19181	1X2124	1X2124	4023297	0.1261	0.0296	0.3069	0.2903	0.0977	0.4732	0	0.2366	2nd
19181	19181	1X3656	1X3656	4023297	0.1167	0.0269	0.2985	0.2667	0.1191	0.5215	0	0.2608	2nd
19181	19181	1X4179	1X4179	4023297	0.1055	0.0456	0.261	0.4296	0.0199	0.1052	0	0.0526	4th
19181	19181	25354	25354	4023297	0.1174	0.0293	0.3171	0.2745	0.11	0.3299	0.0776	0.2425	2nd
19181	19181	26198	26198	4023297	0.0983	0.03	0.2871	0.2598	0.0215	0.24	0	0.12	3rd
19207	19207	1X2124	1X2124	4023297	0.1185	0.047	0.2653	0.4386	0.0371	0	0	0	UN
19207	19207	1X3697	1X3697	4023297	0.1165	0.0444	0.2688	0.4324	0.0444	0.0141	0	0.0071	UN
19207	19207	1X3822	1X3822	4023297	0.1182	0.0441	0.2705	0.4223	0.0522	0.0026	0	0.0013	UN
19207	19207	1X4080	1X4080	4023297	0.1128	0.0463	0.2623	0.4258	0.0243	0	0	0	UN
19207	19207	1X4777	1X4777	4023297	0.1141	0.047	0.2648	0.439	0.0265	0.004	0	0.002	UN
19207	19207	25347	25347	4023297	0.1203	0.0425	0.2715	0.3715	0.0581	0.066	0	0.033	UN
19237	19237	1X3837	1X3837	4023297	0.2261	0.0366	0.3744	0.24	0.1249	0.197	0.008	0.1064	3rd
19348	19348	1X2124	1X2124	4023297	0.1202	0.035	0.2949	0.3267	0.0577	0.4049	0	0.2024	2nd
19348	19348	1X3656	1X3656	4023297	0.1139	0.0295	0.2961	0.2821	0.0975	0.5144	0	0.2572	2nd
19348	19348	25354	25354	4023297	0.1107	0.0203	0.3006	0.1856	0.143	0.4384	0.0235	0.2427	2nd
19371	19371	1X2231	1X2231	4023297	0.1123	0.0263	0.324	0.24	0.0777	0.2708	0.0069	0.1423	3rd
19376	19376	1X4209	1X4209	4023297	0.1911	0.0238	0.3173	0.1595	0.096	0.2273	0.0029	0.1165	3rd
19376	19376	25409	25409	4023297	0.138	0.0556	0.2788	0.3227	0.0379	0.0471	0.0034	0.027	UN
19378	19378	1X2816	1X2816	4023297	0.1105	0.0371	0.2591	0.3194	0.0612	0.1367	0.0094	0.0777	4th
18866	18866	26498	26498	4023297	0.175	0.0341	0.2812	0.3781	0.0851	0	0	0	UN
18929	18929	26498	26498	4023297	0.1778	0.031	0.2914	0.3652	0.094	0	0	0	UN
19181	19181	26988	26988	4023297	0.1546	0.0136	0.4533	0.146	0.2544	0.4937	0.2776	0.5245	FS
19181	19181	27503	27503	4023297	0.116	0.0232	0.3066	0.2202	0.1361	0.5606	0.0349	0.3152	2nd
19207	19207	26498	26498	4023297	0.1562	0.0296	0.2749	0.3566	0.0244	0	0	0	UN
19348	19348	26988	26988	4023297	0.1345	0.0196	0.3817	0.1934	0.1936	0.5159	0.1758	0.4338	FS
19348	19348	27503	27503	4023297	0.1094	0.0252	0.2908	0.2307	0.1196	0.5415	0.0199	0.2907	2nd
19181	19181	28246	28246	4023297	0.1553	0.0102	0.441	0.1168	0.2627	0.621	0.2225	0.533	FS
19348	19348	28246	28246	4023297	0.1343	0.0192	0.3688	0.1977	0.1826	0.5715	0.1298	0.4155	FS
18866	18866	30917	30917	4023297	0.1605	0.0354	0.2909	0.3767	0.1211	0	0	0	UN
18866	18866	30920	30920	4023297	0.1625	0.039	0.2697	0.3938	0.0763	0	0	0	UN
18929	18929	30917	30917	4023297	0.1532	0.0364	0.2785	0.4065	0.104	0	0	0	UN
18929	18929	30920	30920	4023297	0.1602	0.0388	0.269	0.4096	0.069	0	0	0	UN
19207	19207	30917	30917	4023297	0.1358	0.0347	0.2696	0.3962	0.0457	0	0	0	UN
18818	18818	31011	31011	4023297	0.129	0.0414	0.2512	0.3495	0.0281	0.0089	0	0.0045	UN
18866	18866	31011	31011	4023297	0.1403	0.0566	0.2494	0.4773	0.0379	0	0	0	UN
18929	18929	31011	31011	4023297	0.1457	0.0492	0.2661	0.4356	0.0631	0.0033	0	0.0016	UN
18929	18929	31065	31065	4023297	0.1399	0.0564	0.2479	0.4608	0.0256	0	0	0	UN
19181	19181	30961	30961	4023297	0.1019	0.0385	0.2622	0.3607	0.0409	0.1215	0	0.0607	4th

FID1	ID1	FID2	ID2	N_SNP	HetHet	IBS0	HetConc	HomIBS0	Kinship	IBD1Seg	IBD2Seg	PropIBD	InfType
19348	19348	30961	30961	4023297	0.0996	0.042	0.2609	0.379	0.0309	0.1007	0	0.0504	4th
18818	18818	31284	31284	4023297	0.1136	0.0497	0.2409	0.4055	0.0221	0.004	0	0.002	UN
18818	18818	31364	31364	4023297	0.1378	0.0438	0.2951	0.3537	0.0673	0.0047	0	0.0024	UN
18866	18866	31337	31337	4023297	0.1348	0.0534	0.2401	0.4492	0.0354	0.003	0	0.0015	UN
18929	18929	31331	31331	4023297	0.1346	0.0524	0.2513	0.4511	0.0341	0	0	0	UN
18929	18929	31337	31337	4023297	0.1291	0.0543	0.2315	0.4719	0.0285	0.0044	0	0.0022	UN
19207	19207	31297	31297	4023297	0.1119	0.0482	0.2507	0.4451	0.027	0.0025	0	0.0013	UN
19237	19237	31337	31337	4023297	0.1606	0.0548	0.3012	0.3503	0.0705	0.0203	0	0.0101	UN
19237	19237	31477	31477	4023297	0.1424	0.0605	0.2609	0.3145	0.0235	0.0044	0	0.0022	UN
19376	19376	31477	31477	4023297	0.1432	0.0184	0.2813	0.1051	0.1495	0.0279	0.0014	0.0153	UN
18929	18929	31498	31498	4023297	0.137	0.0505	0.2578	0.4497	0.0425	0.0025	0	0.0012	UN
19237	19237	31497	31497	4023297	0.1498	0.0545	0.2723	0.3423	0.0575	0.0075	0	0.0037	UN
18818	18818	31738	31738	4023297	0.118	0.05	0.2529	0.3836	0.0293	0.0025	0	0.0012	UN
18818	18818	31786	31786	4023297	0.123	0.0423	0.273	0.3508	0.0598	0.0115	0	0.0058	UN
18929	18929	31805	31805	4023297	0.1293	0.0534	0.2393	0.4609	0.0224	0.0052	0	0.0026	UN
18818	18818	32040	32040	4023297	0.1157	0.0413	0.2492	0.3496	0.0547	0.0611	0	0.0305	UN
18866	18866	32089	32089	4023297	0.1627	0.0576	0.2937	0.3844	0.0582	0.011	0	0.0055	UN
18929	18929	32043	32043	4023297	0.1621	0.0001	0.3464	0.0012	0.2337	0.9956	0.003	0.5008	PO
18929	18929	32224	32224	4023297	0.1339	0.0518	0.2471	0.4595	0.0384	0.0021	0	0.001	UN
19012	19012	32187	32187	4023297	0.1338	0.0452	0.2603	0.3096	0.0299	0.0162	0	0.0081	UN
19012	19012	32358	32358	4023297	0.1234	0.04	0.2447	0.2804	0.0464	0.0073	0	0.0037	UN
19181	19181	32043	32043	4023297	0.1162	0.0314	0.2753	0.3036	0.0694	0.409	0	0.2045	2nd
19207	19207	32043	32043	4023297	0.1162	0.0462	0.258	0.4294	0.0344	0.0056	0	0.0028	UN
19237	19237	32187	32187	4023297	0.1456	0.0549	0.2632	0.324	0.0512	0.0182	0	0.0091	UN
19348	19348	32043	32043	4023297	0.1286	0.0001	0.3208	0.0007	0.218	0.9965	0	0.4982	PO
19376	19376	32356	32356	4023297	0.1188	0.0405	0.2547	0.2184	0.0319	0	0.0015	0.0015	UN
18818	18818	32557	32557	4023297	0.1092	0.0356	0.2512	0.236	0.037	0	0	0	UN
18929	18929	32849	32849	4023297	0.1835	0.0002	0.3917	0.0016	0.2692	0.9874	0.0074	0.5012	PO
19181	19181	32849	32849	4023297	0.1186	0.032	0.2688	0.3087	0.0503	0.4343	0	0.2172	2nd
19348	19348	32849	32849	4023297	0.1295	0.0001	0.3071	0.0009	0.1974	0.9986	0	0.4993	PO
19376	19376	33082	33082	4023297	0.1256	0.0306	0.2616	0.1715	0.0923	0.0074	0.0013	0.005	UN
19378	19378	32773	32773	4023297	0.1021	0.0275	0.2602	0.2078	0.0498	0	0	0	UN
18769	18769	6716	6716	4023297	0.1647	0.0091	0.3246	0.0643	0.133	0.4152	0.0029	0.2105	2nd
18818	18818	6265	6265	4023297	0.1087	0.0402	0.236	0.3521	0.0387	0.0706	0	0.0353	UN
18818	18818	6716	6716	4023297	0.2108	0.0044	0.4261	0.0433	0.2412	0.3816	0.0367	0.2275	2nd
18818	18818	6955	6955	4023297	0.1082	0.0414	0.2329	0.3628	0.0363	0.0792	0	0.0396	UN
18818	18818	7267	7267	4023297	0.1076	0.0414	0.231	0.3626	0.036	0.1102	0	0.0551	4th
18818	18818	7311	7311	4023297	0.1036	0.0343	0.2292	0.3041	0.0404	0.1648	0	0.0824	4th
18818	18818	7625	7625	4023297	0.1079	0.0418	0.2331	0.3659	0.0325	0.0806	0	0.0403	UN
18818	18818	8170	8170	4023297	0.1038	0.0406	0.2277	0.3605	0.0207	0.1127	0	0.0564	4th
18818	18818	8395	8395	4023297	0.1072	0.0443	0.2312	0.383	0.0231	0.0442	0	0.0221	UN
18866	18866	33908	33908	4023297	0.1298	0.0352	0.26	0.309	0.0401	0.0434	0.0102	0.0319	UN
18866	18866	34568	34568	4023297	0.1704	0.0557	0.3107	0.3838	0.0731	0.0177	0	0.0089	UN
18866	18866	6716	6716	4023297	0.1615	0.0505	0.2673	0.4378	0.0419	0.0066	0	0.0033	UN
18917	18917	6716	6716	4023297	0.1455	0.0099	0.3073	0.0793	0.0516	0.389	0.0054	0.1999	2nd
18917	18917	7311	7311	4023297	0.0937	0.0222	0.2497	0.177	0.0479	0.3606	0	0.1803	2nd
18929	18929	33863	33863	4023297	0.1382	0.0385	0.2814	0.3544	0.0581	0.2264	0	0.1132	3rd
18929	18929	34857	34857	4023297	0.1314	0.0493	0.2501	0.4512	0.0296	0.0136	0	0.0068	UN
18929	18929	6716	6716	4023297	0.1533	0.0506	0.254	0.4561	0.0238	0.0058	0	0.0029	UN
18972	18972	8344	8344	4023297	0.1564	0.0148	0.3165	0.084	0.0912	0.3349	0.0075	0.1749	3rd
19012	19012	6716	6716	4023297	0.2041	0.0038	0.4016	0.0344	0.2322	0.4047	0.0475	0.2499	2nd
19012	19012	7267	7267	4023297	0.1109	0.0472	0.2362	0.3763	0.015	0.1119	0	0.056	4th
19012	19012	7625	7625	4023297	0.1101	0.0462	0.2355	0.3683	0.0147	0.1184	0	0.0592	4th
19144	19144	7158	7158	4023297	0.1434	0.0263	0.2827	0.1239	0.1062	0.1408	0.0139	0.0843	4th
19181	19181	33863	33863	4023297	0.1331	0.0001	0.3293	0.0009	0.2292	0.9979	0	0.4989	PO
19207	19207	33863	33863	4023297	0.1171	0.0461	0.2612	0.4257	0.0374	0.0135	0	0.0068	UN
19207	19207	6265	6265	4023297	0.1163	0.0453	0.2641	0.4306	0.0456	0.0027	0	0.0014	UN
19207	19207	6955	6955	4023297	0.1164	0.0451	0.2623	0.4294	0.0444	0.0026	0	0.0013	UN
19207	19207	7091	7091	4023297	0.1164	0.0439	0.2666	0.4235	0.0485	0.0069	0	0.0034	UN
19207	19207	7267	7267	4023297	0.1164	0.0473	0.2617	0.4477	0.0358	0.0015	0	0.0007	UN
19207	19207	7625	7625	4023297	0.1162	0.0455	0.2629	0.4321	0.0446	0.0026	0	0.0013	UN
19207	19207	8170	8170	4023297	0.1136	0.047	0.2621	0.4516	0.0269	0.0047	0	0.0023	UN
19207	19207	8395	8395	4023297	0.1166	0.0457	0.2639	0.4323	0.0441	0.0026	0	0.0013	UN
19237	19237	7158	7158	4023297	0.1835	0.0088	0.3538	0.0438	0.235	0.278	0.076	0.215	2nd
19348	19348	33863	33863	4023297	0.1112	0.0326	0.2668	0.303	0.0486	0.4073	0	0.2036	2nd
19378	19378	6265	6265	4023297	0.1064	0.0439	0.2397	0.3629	0.029	0.083	0	0.0415	UN
19378	19378	6955	6955	4023297	0.1119	0.044	0.2533	0.3662	0.0359	0.0868	0.0048	0.0483	4th
19378	19378	7625	7625	4023297	0.1097	0.0443	0.248	0.3686	0.0323	0.0846	0.0014	0.0437	UN
19378	19378	8395	8395	4023297	0.1133	0.0313	0.258	0.2663	0.0862	0.1521	0	0.0761	4th
19658	19658	6716	6716	4023297	0.1623	0.0137	0.3382	0.1109	0.0924	0.2762	0.0015	0.1396	3rd
18778	18778	9656	9656	4023297	0.1908	0.0159	0.3616	0.0624	0.1643	0.422	0.0078	0.2188	2nd
18818	18818	8465	8465	4023297	0.1335	0.0361	0.2476	0.3163	0.0281	0.1023	0	0.0512	4th
18818	18818	9045	9045	4023297	0.104	0.0402	0.2311	0.3491	0.0175	0.0997	0.0014	0.0512	4th
18818	18818	9128	9128	4023297	0.1072	0.0355	0.2314	0.315	0.0539	0.1249	0	0.0624	4th
18866	18866	8465	8465	4023297	0.1536	0.0508	0.265	0.4476	0.0527	0.0025	0	0.0013	UN
18866	18866	9860	9860	4023297	0.1567	0.0505	0.2703	0.4254	0.0558	0	0	0	UN
18929	18929	8465	8465	4023297	0.1494	0.0474	0.2601	0.4351	0.0513	0.0054	0	0.0027	UN
18929	18929	9860	9860	4023297	0.148	0.0495	0.2554	0.4331	0.0407	0	0	0	UN
18938	18938	9562	9562	4023297	0.1238	0.0084	0.306	0.0806	0.1918	0.2179	0.0026	0.1116	3rd
18974	18974	9656	9656	4023297	0.1764	0.0128	0.3398	0.0483	0.1424	0.5105	0.0143	0.2695	2nd
19012	19012	8465	8465	4023297	0.1363	0.036	0.2509	0.2906	0.0384	0.1484	0.0049	0.0791	4th
19165	19165	9562	9562	4023297	0.0976	0.0081	0.3055	0.0675	0.0579	0.4989	0	0.2494	2nd
19173	19173	9562	9562	4023297	0.1149	0.0095	0.3282	0.0896	0.1436	0.2397	0	0.1199	3rd
19181	19181	9841	9841	4023297	0.1347	0.0186	0.356	0.1992	0.1825	0.5465	0.0906	0.3639	FS
19207	19207	8780	8780	4023297	0.1171	0.0424	0.2687	0.4208	0.0551	0.0163	0	0.0081	UN
19207	19207	8995	8995	4023297	0.1162	0.0482	0.2606	0.438	0.0312	0	0	0	UN
19207	19207	9128	9128	4023297	0.1161	0.0447	0.2629	0.426	0.0476	0.0013	0	0.0006	UN
19207	19207	9562	9562	4023297	0.1168	0.0458	0.2685	0.4374	0.0412	0	0	0	UN
19215	19215	9562	9562	4023297	0.1166	0.0073	0.3367	0.0687	0.1583	0.3213	0	0.1607	3rd
19348	19348	9841	9841	4023297	0.1473	0.0001	0.4127	0.0006	0.2829	0.841	0.1564	0.5769	PO
19359	19359	9562	9562	4023297	0.1135	0.0113	0.331	0.1045	0.1246	0.321	0	0.1605	3rd
19371	19371	9562	9562	4023297	0.1125	0.0088	0.3108	0.0838	0.146	0.1454	0	0.0727	4th
19376	19376	8465	8465	4023297	0.1708	0.0058	0.326	0.0413	0.1997	0.3188	0.0039	0.1633	3rd
19378	19378	8465	8465	4023297	0.1692	0.0069	0.3492	0.0614	0.1852	0.2492	0.0109	0.1355	3rd
19378	19378	9128	9128	4023297	0.1077	0.0465	0.2427	0.3852	0.0209	0.1114	0	0.0557	4th
19674	19674	8780	8780	4023297	0.1312	0.0023	0.3809	0.023	0.2237	0.531	0.0976	0.3631	FS
1X2124	1X2124	1X2816	1X2816	4023297	0.1162	0.0444	0.2673	0.4514	0.0312	0.0826	0	0.0413	UN
1X2124	1X2124	1X3697	1X3697	4023297	0.1175	0.0445	0.267	0.4529	0.039	0	0	0	UN

FID1	ID1	FID2	ID2	N_SNP	HetHet	IBS0	HetConc	HomIBS0	Kinship	IBD1Seg	IBD2Seg	PropIBD	InfType
1X2124	1X2124	1X3822	1X3822	4023297	0.1185	0.0448	0.2668	0.4474	0.0435	0.0157	0	0.0078	UN
1X2124	1X2124	1X4080	1X4080	4023297	0.1162	0.0452	0.2676	0.4343	0.0277	0.0332	0	0.0166	UN
1X2124	1X2124	1X4777	1X4777	4023297	0.1161	0.0455	0.2659	0.4449	0.0284	0.0249	0	0.0125	UN
1X2124	1X2124	25347	25347	4023297	0.1163	0.0504	0.2557	0.4485	0.0258	0.0061	0	0.0031	UN
1X2124	1X2124	25354	25354	4023297	0.1195	0.0322	0.2956	0.3009	0.0644	0.3148	0	0.1574	3rd
1X2124	1X2124	26196	26196	4023297	0.1205	0.0457	0.2588	0.4185	0.0391	0.0156	0	0.0078	UN
1X2231	1X2231	1X3822	1X3822	4023297	0.112	0.0468	0.265	0.4527	0.018	0.0962	0	0.0481	4th
1X2231	1X2231	1X4080	1X4080	4023297	0.11	0.0436	0.2669	0.4102	0.0386	0.1103	0	0.0552	4th
1X2231	1X2231	1X4777	1X4777	4023297	0.1233	0.0254	0.3074	0.2561	0.1323	0.4748	0.0021	0.2395	2nd
1X2816	1X2816	1X3822	1X3822	4023297	0.1121	0.0463	0.2613	0.48	0.0258	0.0367	0	0.0183	UN
1X2816	1X2816	1X4080	1X4080	4023297	0.1092	0.0457	0.2602	0.4544	0.0331	0.0282	0	0.0141	UN
1X2816	1X2816	1X4777	1X4777	4023297	0.1111	0.0451	0.2645	0.4585	0.0377	0.024	0	0.012	UN
1X3656	1X3656	1X3697	1X3697	4023297	0.1105	0.0422	0.2643	0.4352	0.0367	0.0075	0	0.0037	UN
1X3656	1X3656	1X3822	1X3822	4023297	0.1105	0.0443	0.2613	0.4464	0.0239	0.0123	0	0.0061	UN
1X3656	1X3656	1X4080	1X4080	4023297	0.108	0.0447	0.2615	0.4322	0.0294	0.012	0	0.006	UN
1X3656	1X3656	1X4179	1X4179	4023297	0.1238	0.0222	0.3155	0.2347	0.1525	0.4411	0.0085	0.229	2nd
1X3656	1X3656	1X4777	1X4777	4023297	0.1087	0.0444	0.2619	0.4383	0.0296	0.0099	0	0.0049	UN
1X3697	1X3697	1X3822	1X3822	4023297	0.1357	0.0178	0.3292	0.2007	0.1796	0.5788	0	0.2894	2nd
1X3697	1X3697	1X4080	1X4080	4023297	0.1105	0.0442	0.2601	0.4424	0.035	0.0029	0	0.0015	UN
1X3697	1X3697	1X4179	1X4179	4023297	0.1125	0.0427	0.2692	0.4372	0.0401	0.0031	0	0.0015	UN
1X3697	1X3697	1X4777	1X4777	4023297	0.1127	0.0441	0.2651	0.4499	0.0415	0.0159	0	0.0079	UN
1X3796	1X3796	1X3837	1X3837	4023297	0.2602	0.0329	0.375	0.2896	0.2017	0.0386	0	0.0193	UN
1X3796	1X3796	1X4209	1X4209	4023297	0.2603	0.0329	0.3755	0.2919	0.2022	0.0146	0	0.0073	UN
1X3822	1X3822	1X4080	1X4080	4023297	0.1131	0.0443	0.2647	0.4352	0.0349	0.0354	0	0.0177	UN
1X3822	1X3822	1X4179	1X4179	4023297	0.1122	0.0437	0.2651	0.4375	0.0311	0.0029	0	0.0014	UN
1X3822	1X3822	1X4777	1X4777	4023297	0.1171	0.0415	0.2751	0.4177	0.0549	0.101	0	0.0505	4th
1X3822	1X3822	25347	25347	4023297	0.1156	0.0487	0.2595	0.4443	0.0253	0.0044	0	0.0022	UN
1X3837	1X3837	1X4209	1X4209	4023297	0.263	0.0327	0.3772	0.296	0.2057	0.0267	0	0.0134	UN
1X4080	1X4080	1X4179	1X4179	4023297	0.1094	0.0447	0.2645	0.4303	0.0338	0.0069	0	0.0034	UN
1X4080	1X4080	1X4777	1X4777	4023297	0.1134	0.0413	0.272	0.4016	0.0563	0.0725	0	0.0363	UN
1X4179	1X4179	1X4777	1X4777	4023297	0.1088	0.045	0.2612	0.4406	0.029	0.0012	0	0.0006	UN
1X4209	1X4209	25409	25409	4023297	0.1933	0.0226	0.3189	0.1633	0.1063	0.1817	0.0054	0.0962	3rd
1X2124	1X2124	26498	26498	4023297	0.1583	0.028	0.2759	0.3617	0.0394	0.004	0	0.002	UN
1X2124	1X2124	26988	26988	4023297	0.1269	0.0266	0.3138	0.2594	0.1083	0.5953	0	0.2976	2nd
1X2124	1X2124	27503	27503	4023297	0.1204	0.0329	0.294	0.3103	0.0687	0.4174	0	0.2087	2nd
1X2231	1X2231	26498	26498	4023297	0.1661	0.0131	0.309	0.1708	0.0893	0.2361	0	0.118	3rd
1X2231	1X2231	26800	26800	4023297	0.1216	0.0124	0.3536	0.1196	0.1724	0.3352	0.0591	0.2266	2nd
1X2231	1X2231	26980	26980	4023297	0.1079	0.0164	0.2966	0.1383	0.124	0.4146	0.0211	0.2284	2nd
1X2231	1X2231	27351	27351	4023297	0.1473	0.0134	0.37	0.1333	0.2052	0.5373	0.2411	0.5097	FS
1X2231	1X2231	27594	27594	4023297	0.101	0.0338	0.2667	0.2873	0.0342	0.1844	0.0119	0.1041	3rd
1X2231	1X2231	27666	27666	4023297	0.0927	0.0345	0.2371	0.2597	0.0165	0.1919	0.0033	0.0992	3rd
1X3656	1X3656	26988	26988	4023297	0.1172	0.0227	0.3044	0.2246	0.1343	0.6243	0.0021	0.3142	2nd
1X3697	1X3697	27306	27306	4023297	0.1149	0.0088	0.3437	0.0793	0.1422	0.4971	0.0431	0.2917	2nd
1X3796	1X3796	26498	26498	4023297	0.225	0.0314	0.3241	0.3637	0.1663	0.0019	0	0.001	UN
1X3822	1X3822	26498	26498	4023297	0.156	0.0267	0.2757	0.3573	0.0325	0.0125	0	0.0062	UN
1X3837	1X3837	26498	26498	4023297	0.225	0.0299	0.321	0.3554	0.1658	0	0	0	UN
1X4179	1X4179	26988	26988	4023297	0.1041	0.0449	0.2604	0.4176	0.0151	0.1354	0	0.0677	4th
1X4209	1X4209	26498	26498	4023297	0.2715	0.013	0.4151	0.1626	0.2563	0.0024	0	0.0012	UN
1X4777	1X4777	26498	26498	4023297	0.1708	0.0115	0.3155	0.1563	0.1091	0.1619	0	0.081	4th
1X4777	1X4777	26800	26800	4023297	0.1039	0.0281	0.281	0.2549	0.0438	0.1629	0.0045	0.086	4th
1X4777	1X4777	27351	27351	4023297	0.1098	0.0412	0.2474	0.3607	0.0321	0.2552	0.0021	0.1297	3rd
25347	25347	26498	26498	4023297	0.1577	0.0311	0.2754	0.3545	0.0263	0.0012	0	0.0006	UN
25354	25354	26988	26988	4023297	0.1107	0.0215	0.2978	0.2008	0.1345	0.4442	0.0328	0.2549	2nd
25354	25354	27503	27503	4023297	0.1097	0.0231	0.2952	0.2106	0.1269	0.3198	0.0266	0.1865	2nd
25409	25409	26498	26498	4023297	0.1694	0.0438	0.2844	0.3695	0.0296	0	0	0	UN
26196	26196	26498	26498	4023297	0.1608	0.0304	0.2752	0.3566	0.0453	0	0	0	UN
26198	26198	27503	27503	4023297	0.1017	0.0245	0.3058	0.2167	0.0681	0.2179	0.0335	0.1425	3rd
1X2124	1X2124	28246	28246	4023297	0.1318	0.0239	0.3202	0.2504	0.1348	0.5602	0	0.2801	2nd
1X2231	1X2231	28279	28279	4023297	0.1462	0.0149	0.3474	0.1521	0.1764	0.541	0.1702	0.4407	FS
1X2816	1X2816	28076	28076	4023297	0.1128	0.0301	0.2806	0.2891	0.0906	0.1693	0.0081	0.0927	3rd
1X2816	1X2816	28304	28304	4023297	0.1264	0.0196	0.3786	0.189	0.1347	0.3306	0.1058	0.2711	2nd
1X3656	1X3656	28246	28246	4023297	0.1242	0.0171	0.3186	0.1838	0.175	0.7116	0	0.3558	2nd
1X3697	1X3697	27989	27989	4023297	0.1372	0.0087	0.3996	0.0891	0.2119	0.4426	0.1695	0.3908	FS
1X3697	1X3697	28183	28183	4023297	0.1289	0.0048	0.3667	0.0472	0.2106	0.6724	0.1314	0.4676	FS
1X3822	1X3822	27989	27989	4023297	0.1058	0.0271	0.2786	0.2436	0.0427	0.3244	0.0014	0.1636	3rd
1X3822	1X3822	28183	28183	4023297	0.1034	0.0254	0.2707	0.2236	0.0449	0.466	0	0.233	2nd
1X3837	1X3837	28037	28037	4023297	0.1899	0.0344	0.319	0.202	0.0544	0.2028	0	0.1014	3rd
1X3837	1X3837	28281	28281	4023297	0.184	0.0336	0.3059	0.1747	0.0478	0.2148	0	0.1074	3rd
1X4080	1X4080	28279	28279	4023297	0.1154	0.0426	0.2523	0.3828	0.0148	0.2194	0	0.1097	3rd
1X4179	1X4179	28246	28246	4023297	0.1107	0.0395	0.2731	0.3972	0.0596	0.1937	0	0.0969	3rd
1X4777	1X4777	28279	28279	4023297	0.1172	0.0395	0.2559	0.3637	0.032	0.2476	0	0.1238	3rd
25354	25354	28246	28246	4023297	0.1198	0.0212	0.3199	0.2102	0.1422	0.521	0.0378	0.2983	2nd
26198	26198	28246	28246	4023297	0.0962	0.0234	0.2741	0.2129	0.0423	0.2563	0.0082	0.1364	3rd
1X2231	1X2231	28400	28400	4023297	0.1148	0.0107	0.3529	0.0982	0.152	0.3933	0.023	0.2197	2nd
1X2231	1X2231	28416	28416	4023297	0.1105	0.0218	0.3179	0.1954	0.0947	0.2897	0.0257	0.1705	3rd
1X3697	1X3697	28417	28417	4023297	0.1122	0.0153	0.3358	0.1396	0.0949	0.4082	0.0128	0.2169	2nd
1X3697	1X3697	28635	28635	4023297	0.1083	0.0229	0.2572	0.2027	0.1081	0.6099	0	0.305	2nd
1X3822	1X3822	28635	28635	4023297	0.1107	0.0272	0.2611	0.2345	0.0914	0.5958	0.012	0.3099	2nd
1X4777	1X4777	28416	28416	4023297	0.1045	0.0229	0.2887	0.2033	0.0637	0.2244	0.0027	0.1149	3rd
26138	26138	28431	28431	4023297	0.1365	0.049	0.3108	0.3201	0.0594	0	0	0	UN
1X2124	1X2124	30917	30917	4023297	0.1389	0.0328	0.2734	0.3993	0.0631	0	0	0	UN
1X2231	1X2231	30614	30614	4023297	0.1017	0.0324	0.2436	0.2862	0.0685	0.0207	0	0.0103	UN
1X2231	1X2231	30917	30917	4023297	0.1407	0.0225	0.2942	0.2703	0.0861	0.0066	0	0.0033	UN
1X2816	1X2816	30917	30917	4023297	0.1373	0.0277	0.2813	0.36	0.0639	0.0063	0	0.0031	UN
1X3697	1X3697	30611	30611	4023297	0.1057	0.036	0.2632	0.3443	0.0334	0.0263	0	0.0131	UN
1X3697	1X3697	30917	30917	4023297	0.1413	0.026	0.2878	0.34	0.0822	0.0037	0	0.0019	UN
1X3697	1X3697	30920	30920	4023297	0.1432	0.0288	0.2645	0.3521	0.0271	0	0	0	UN
1X3796	1X3796	30917	30917	4023297	0.2015	0.0286	0.3183	0.3181	0.1216	0	0	0	UN
1X3796	1X3796	30920	30920	4023297	0.2145	0.035	0.3188	0.3658	0.1382	0	0	0	UN
1X3822	1X3822	30917	30917	4023297	0.1464	0.0251	0.2985	0.322	0.098	0.0048	0	0.0024	UN
1X3837	1X3837	30917	30917	4023297	0.1774	0.0385	0.2672	0.4297	0.0562	0	0	0	UN
1X3837	1X3837	30920	30920	4023297	0.2131	0.035	0.313	0.3743	0.1324	0	0	0	UN
1X4080	1X4080	30917	30917	4023297	0.1334	0.0322	0.2715	0.3835	0.0393	0.0034	0	0.0017	UN
1X4179	1X4179	30611	30611	4023297	0.1003	0.0307	0.2544	0.2857	0.0578	0.0491	0	0.0246	UN
1X4179	1X4179	30886	30886	4023297	0.1032	0.029	0.2325	0.2426	0.059	0.0206	0	0.0103	UN
1X4209	1X4209	30917	30917	4023297	0.1801	0.0383	0.2727	0.4338	0.061	0	0	0	UN
1X4209	1X4209	30920	30920	4023297	0.2209	0.0322	0.3285	0.3493	0.1492	0	0	0	UN
1X4777	1X4777	30917	30917	4023297	0.1385	0.0285	0.2833	0.3513	0.0645	0.0055	0	0.0028	UN

FID1	ID1	FID2	ID2	N_SNP	HetHet	IBS0	HetConc	HomIBS0	Kinship	IBD1Seg	IBD2Seg	PropIBD	InfType
25347	25347	30917	30917	4023297	0.135	0.0386	0.2645	0.4165	0.0346	0	0	0	UN
26196	26196	30917	30917	4023297	0.1417	0.0361	0.2733	0.4019	0.0651	0	0	0	UN
1X2231	1X2231	31021	31021	4023297	0.1234	0.0187	0.2737	0.1855	0.1104	0.5332	0	0.2666	2nd
1X2231	1X2231	31134	31134	4023297	0.1139	0.0232	0.2941	0.2217	0.1236	0.1231	0.0033	0.0648	4th
1X2816	1X2816	30961	30961	4023297	0.1054	0.041	0.264	0.4107	0.023	0.0429	0	0.0215	UN
1X2816	1X2816	31011	31011	4023297	0.1273	0.0173	0.2607	0.182	0.0937	0.3181	0	0.1591	3rd
1X2816	1X2816	31124	31124	4023297	0.1043	0.0339	0.266	0.327	0.043	0.0351	0	0.0176	UN
1X3656	1X3656	31145	31145	4023297	0.1181	0.03	0.2642	0.3075	0.0635	0.3486	0	0.1743	3rd
1X3697	1X3697	30961	30961	4023297	0.1081	0.0401	0.2681	0.4054	0.0252	0.0327	0	0.0164	UN
1X3697	1X3697	31016	31016	4023297	0.1112	0.0338	0.2792	0.3345	0.0564	0.0686	0	0.0343	UN
1X3697	1X3697	31021	31021	4023297	0.1231	0.0356	0.265	0.3629	0.0543	0.2769	0	0.1385	3rd
1X3697	1X3697	31145	31145	4023297	0.1294	0.0267	0.2876	0.2883	0.1063	0.4597	0	0.2298	2nd
1X3697	1X3697	31234	31234	4023297	0.1127	0.0364	0.2644	0.3576	0.0707	0.0449	0	0.0225	UN
1X3796	1X3796	30933	30933	4023297	0.1882	0.0478	0.2921	0.3977	0.0489	0	0	0	UN
1X3796	1X3796	31011	31011	4023297	0.1798	0.0491	0.2789	0.4289	0.0287	0	0	0	UN
1X3796	1X3796	31065	31065	4023297	0.1912	0.0474	0.2967	0.3862	0.056	0	0	0	UN
1X3822	1X3822	31016	31016	4023297	0.1134	0.0323	0.2829	0.3153	0.0624	0.0819	0	0.041	UN
1X3822	1X3822	31021	31021	4023297	0.1283	0.0353	0.2763	0.3576	0.0682	0.2531	0.0141	0.1407	3rd
1X3822	1X3822	31145	31145	4023297	0.1258	0.0304	0.2745	0.3175	0.089	0.3314	0.0027	0.1684	3rd
1X3822	1X3822	31234	31234	4023297	0.1122	0.0394	0.2598	0.3767	0.0541	0.0448	0	0.0224	UN
1X3837	1X3837	30933	30933	4023297	0.1969	0.043	0.3067	0.3667	0.0697	0.0181	0	0.009	UN
1X3837	1X3837	31011	31011	4023297	0.2065	0.0375	0.3304	0.3407	0.0949	0.1311	0	0.0656	4th
1X3837	1X3837	31065	31065	4023297	0.1908	0.0475	0.2928	0.3942	0.0504	0	0	0	UN
1X4179	1X4179	31145	31145	4023297	0.1259	0.018	0.2853	0.1892	0.1261	0.6005	0	0.3003	2nd
1X4209	1X4209	30933	30933	4023297	0.19	0.0464	0.293	0.3968	0.0509	0	0	0	UN
1X4209	1X4209	31011	31011	4023297	0.1835	0.0468	0.2834	0.4208	0.0363	0.0014	0	0.0007	UN
1X4209	1X4209	31065	31065	4023297	0.192	0.0467	0.2954	0.391	0.0545	0	0	0	UN
1X4777	1X4777	31021	31021	4023297	0.1189	0.037	0.2563	0.3611	0.0377	0.2181	0	0.1091	3rd
1X4777	1X4777	31134	31134	4023297	0.1076	0.0392	0.2678	0.368	0.0361	0.0456	0	0.0228	UN
26196	26196	31145	31145	4023297	0.1178	0.0497	0.2404	0.4532	0.0237	0.0063	0	0.0031	UN
1X2124	1X2124	31297	31297	4023297	0.1139	0.0474	0.2519	0.4578	0.0281	0.008	0	0.004	UN
1X2231	1X2231	31284	31284	4023297	0.1172	0.0282	0.2694	0.2683	0.0831	0.1894	0	0.0947	3rd
1X2231	1X2231	31297	31297	4023297	0.1141	0.0402	0.2692	0.386	0.0445	0.1803	0.0019	0.092	3rd
1X2231	1X2231	31299	31299	4023297	0.1074	0.041	0.2581	0.3853	0.0422	0.0677	0	0.0339	UN
1X2231	1X2231	31331	31331	4023297	0.1184	0.0241	0.2533	0.2293	0.0688	0.1854	0	0.0927	3rd
1X2816	1X2816	31284	31284	4023297	0.1159	0.035	0.2617	0.3502	0.0588	0.1393	0.0024	0.072	4th
1X2816	1X2816	31327	31327	4023297	0.1024	0.0334	0.2669	0.3287	0.0314	0.0384	0	0.0192	UN
1X2816	1X2816	31409	31409	4023297	0.0979	0.0389	0.2344	0.344	0.0259	0.0273	0	0.0137	UN
1X2816	1X2816	31462	31462	4023297	0.1098	0.033	0.2526	0.318	0.0686	0.0348	0	0.0174	UN
1X2816	1X2816	31484	31484	4023297	0.099	0.0243	0.274	0.227	0.0408	0.0364	0	0.0182	UN
1X3697	1X3697	31297	31297	4023297	0.1106	0.0464	0.2512	0.4672	0.0251	0.0127	0	0.0064	UN
1X3822	1X3822	31297	31297	4023297	0.1143	0.044	0.2588	0.4375	0.0446	0.0568	0	0.0284	UN
1X3837	1X3837	31337	31337	4023297	0.1973	0.0399	0.3143	0.3562	0.0717	0.1123	0	0.0561	4th
1X3837	1X3837	31477	31477	4023297	0.1886	0.042	0.2991	0.272	0.0497	0.1268	0	0.0634	4th
1X4209	1X4209	31331	31331	4023297	0.1913	0.0346	0.3104	0.3131	0.0699	0.174	0	0.087	4th
1X4209	1X4209	31337	31337	4023297	0.1787	0.0467	0.2768	0.4152	0.0256	0.0062	0	0.0031	UN
1X4209	1X4209	31410	31410	4023297	0.1882	0.043	0.2975	0.335	0.0481	0.047	0	0.0235	UN
1X4209	1X4209	31477	31477	4023297	0.1924	0.035	0.3072	0.2321	0.0765	0.1313	0	0.0657	4th
1X4777	1X4777	31284	31284	4023297	0.1131	0.0402	0.2528	0.3831	0.0354	0.0714	0	0.0357	UN
1X4777	1X4777	31297	31297	4023297	0.1256	0.0188	0.2986	0.195	0.1527	0.4654	0	0.2327	2nd
1X4777	1X4777	31299	31299	4023297	0.1189	0.0209	0.2879	0.2105	0.1443	0.1839	0	0.0919	3rd
1X4777	1X4777	31331	31331	4023297	0.1159	0.0389	0.2423	0.3672	0.0142	0.0961	0	0.048	4th
25409	25409	31331	31331	4023297	0.1602	0.013	0.329	0.095	0.2038	0.0116	0	0.0058	UN
1X2231	1X2231	31498	31498	4023297	0.1238	0.0177	0.2689	0.1757	0.1055	0.4234	0	0.2117	2nd
1X2816	1X2816	31500	31500	4023297	0.1066	0.0384	0.2668	0.3897	0.0375	0.0669	0	0.0335	UN
1X2816	1X2816	31540	31540	4023297	0.0954	0.0246	0.2663	0.2251	0.0223	0.0168	0	0.0084	UN
1X2816	1X2816	31624	31624	4023297	0.1097	0.0297	0.2712	0.2967	0.0855	0.0734	0	0.0367	UN
1X3697	1X3697	31497	31497	4023297	0.1235	0.0234	0.2478	0.2319	0.0686	0.1497	0	0.0748	4th
1X3697	1X3697	31498	31498	4023297	0.1209	0.0387	0.2536	0.3909	0.03	0.1675	0	0.0837	4th
1X3796	1X3796	31497	31497	4023297	0.1805	0.0483	0.2803	0.4053	0.0317	0.0012	0	0.0006	UN
1X3822	1X3822	31498	31498	4023297	0.1239	0.0395	0.2589	0.3937	0.0364	0.1341	0.0057	0.0727	4th
1X3837	1X3837	31497	31497	4023297	0.2014	0.0399	0.3199	0.3468	0.0808	0.0728	0	0.0364	UN
1X3837	1X3837	31656	31656	4023297	0.193	0.0435	0.3095	0.3646	0.0505	0.0201	0	0.01	UN
1X4209	1X4209	31497	31497	4023297	0.182	0.047	0.2807	0.4055	0.0331	0.0017	0	0.0009	UN
1X4777	1X4777	31498	31498	4023297	0.1191	0.0363	0.2515	0.3556	0.0311	0.1985	0	0.0992	3rd
1X4777	1X4777	31559	31559	4023297	0.1061	0.0259	0.2791	0.2453	0.0706	0.0508	0	0.0254	UN
1X2231	1X2231	31786	31786	4023297	0.1113	0.0339	0.2594	0.3182	0.0611	0.0715	0	0.0357	UN
1X2231	1X2231	31970	31970	4023297	0.107	0.0331	0.2426	0.2956	0.0482	0.05	0	0.025	UN
1X2816	1X2816	31716	31716	4023297	0.1101	0.0352	0.238	0.3309	0.0343	0.0388	0	0.0194	UN
1X2816	1X2816	31738	31738	4023297	0.1118	0.042	0.2506	0.3831	0.0257	0.0657	0	0.0328	UN
1X2816	1X2816	31764	31764	4023297	0.1097	0.0378	0.2581	0.3564	0.0596	0.0588	0	0.0294	UN
1X2816	1X2816	31786	31786	4023297	0.1127	0.036	0.2596	0.3587	0.0605	0.0847	0	0.0423	UN
1X3656	1X3656	31720	31720	4023297	0.0999	0.0398	0.2377	0.3505	0.0337	0.013	0	0.0065	UN
1X3697	1X3697	31720	31720	4023297	0.1056	0.0433	0.2463	0.3902	0.0282	0.0105	0	0.0053	UN
1X3697	1X3697	31966	31966	4023297	0.1089	0.0422	0.2515	0.4009	0.0444	0.0334	0	0.0167	UN
1X3796	1X3796	31903	31903	4023297	0.1938	0.0515	0.2988	0.3474	0.052	0.0056	0	0.0028	UN
1X3796	1X3796	31970	31970	4023297	0.1786	0.0235	0.3054	0.2014	0.0683	0.0333	0	0.0167	UN
1X3822	1X3822	31966	31966	4023297	0.1085	0.0446	0.2476	0.4139	0.0303	0.0296	0	0.0148	UN
1X3837	1X3837	31903	31903	4023297	0.2042	0.0449	0.3167	0.3095	0.0795	0.0407	0	0.0204	UN
1X4179	1X4179	31720	31720	4023297	0.0999	0.0395	0.2366	0.3464	0.0366	0.0207	0	0.0103	UN
1X4209	1X4209	31800	31800	4023297	0.185	0.0282	0.3023	0.2354	0.0742	0.0958	0	0.0479	4th
1X4209	1X4209	31903	31903	4023297	0.1968	0.0512	0.3019	0.3534	0.0525	0.0033	0	0.0016	UN
1X4777	1X4777	31786	31786	4023297	0.1106	0.0397	0.2524	0.3787	0.0437	0.0594	0	0.0297	UN
1X4777	1X4777	31882	31882	4023297	0.1014	0.0298	0.2632	0.2729	0.0427	0.0472	0	0.0236	UN
1X4777	1X4777	31938	31938	4023297	0.1008	0.037	0.2434	0.3147	0.0361	0.0265	0	0.0132	UN
1X4777	1X4777	31952	31952	4023297	0.115	0.0193	0.2887	0.1898	0.1349	0.0837	0	0.0418	UN
1X2124	1X2124	32040	32040	4023297	0.1153	0.049	0.251	0.4641	0.0285	0.002	0	0.001	UN
1X2124	1X2124	32043	32043	4023297	0.1243	0.0367	0.2764	0.3643	0.0874	0.1931	0	0.0966	3rd
1X2231	1X2231	32025	32025	4023297	0.1131	0.0376	0.2728	0.3636	0.0623	0.1938	0.0014	0.0983	3rd
1X2816	1X2816	32040	32040	4023297	0.1189	0.0286	0.2739	0.294	0.0944	0.3852	0	0.1926	2nd
1X2816	1X2816	32224	32224	4023297	0.1212	0.0286	0.2543	0.2949	0.0561	0.2867	0	0.1433	3rd
1X3656	1X3656	32043	32043	4023297	0.1186	0.0328	0.2784	0.3329	0.0734	0.2931	0	0.1465	3rd
1X3697	1X3697	32025	32025	4023297	0.1103	0.0465	0.2559	0.469	0.0303	0.0063	0	0.0031	UN
1X3697	1X3697	32043	32043	4023297	0.1143	0.0449	0.2568	0.4523	0.0299	0.0101	0	0.005	UN
1X3796	1X3796	32089	32089	4023297	0.2254	0.0133	0.3677	0.0959	0.1979	0.5777	0.003	0.2918	2nd
1X3822	1X3822	32025	32025	4023297	0.1119	0.045	0.2577	0.4461	0.0344	0.0344	0	0.0172	UN
1X3822	1X3822	32043	32043	4023297	0.1162	0.0455	0.2594	0.449	0.0355	0.0145	0	0.0073	UN
1X3837	1X3837	32089	32089	4023297	0.1988	0.0515	0.3075	0.3537	0.0519	0.0447	0	0.0223	UN

FID1	ID1	FID2	ID2	N_SNP	HetHet	IBS0	HetConc	HomIBS0	Kinship	IBD1Seg	IBD2Seg	PropIBD	InfType
1X3837	1X3837	32187	32187	4023297	0.1915	0.0423	0.3	0.3282	0.0593	0.0915	0	0.0457	4th
1X4080	1X4080	32025	32025	4023297	0.1088	0.0468	0.256	0.4466	0.0234	0.0173	0	0.0086	UN
1X4179	1X4179	32043	32043	4023297	0.1122	0.0429	0.2583	0.4191	0.0232	0.0456	0	0.0228	UN
1X4209	1X4209	32089	32089	4023297	0.1954	0.0508	0.301	0.3517	0.0497	0.0152	0	0.0076	UN
1X4209	1X4209	32224	32224	4023297	0.1843	0.0429	0.293	0.3889	0.0379	0.1115	0	0.0558	4th
1X4777	1X4777	32025	32025	4023297	0.1249	0.0214	0.3038	0.2211	0.1509	0.4433	0	0.2216	2nd
1X4777	1X4777	32043	32043	4023297	0.1143	0.0459	0.2598	0.445	0.0223	0.0305	0	0.0153	UN
25354	25354	32043	32043	4023297	0.1095	0.0361	0.2633	0.332	0.0255	0.2109	0	0.1054	3rd
26196	26196	32043	32043	4023297	0.1217	0.0388	0.2614	0.3587	0.0663	0.2185	0	0.1092	3rd
1X2124	1X2124	32849	32849	4023297	0.1258	0.0379	0.2677	0.3721	0.0672	0.1884	0	0.0942	3rd
1X3656	1X3656	32849	32849	4023297	0.1176	0.0344	0.2619	0.3433	0.0441	0.2717	0	0.1358	3rd
1X3656	1X3656	33083	33083	4023297	0.1002	0.0387	0.2467	0.349	0.0381	0.0194	0	0.0097	UN
1X3697	1X3697	32591	32591	4023297	0.0999	0.0363	0.2369	0.3109	0.033	0.0066	0	0.0033	UN
1X3697	1X3697	33083	33083	4023297	0.1068	0.0397	0.258	0.3679	0.0328	0.0163	0	0.0082	UN
1X4179	1X4179	33083	33083	4023297	0.0999	0.0398	0.2445	0.3563	0.0308	0.0204	0	0.0102	UN
1X4777	1X4777	32772	32772	4023297	0.11	0.0259	0.2897	0.249	0.0817	0.0716	0	0.0358	UN
1X4777	1X4777	32909	32909	4023297	0.104	0.0275	0.2667	0.2517	0.0645	0.0376	0	0.0188	UN
25354	25354	32849	32849	4023297	0.1117	0.0316	0.2566	0.2939	0.0261	0.2873	0	0.1437	3rd
26196	26196	32849	32849	4023297	0.1268	0.0406	0.2628	0.3725	0.0679	0.1979	0.0036	0.1026	3rd
1X1155	1X1155	7937	7937	4023297	0.1127	0.0248	0.2941	0.2322	0.1146	0.5264	0	0.2632	2nd
1X2124	1X2124	33863	33863	4023297	0.1257	0.0348	0.2809	0.3437	0.0977	0.2381	0	0.119	3rd
1X2124	1X2124	6265	6265	4023297	0.12	0.0439	0.2701	0.4368	0.0505	0.0378	0	0.0189	UN
1X2124	1X2124	6955	6955	4023297	0.1202	0.0438	0.2686	0.4364	0.0534	0.027	0	0.0135	UN
1X2124	1X2124	7091	7091	4023297	0.1178	0.0442	0.2659	0.4443	0.0427	0.0014	0	0.0007	UN
1X2124	1X2124	7267	7267	4023297	0.1281	0.0374	0.2907	0.3788	0.0908	0.0229	0	0.0115	UN
1X2124	1X2124	7311	7311	4023297	0.1159	0.0445	0.2667	0.4399	0.0301	0.0701	0	0.035	UN
1X2124	1X2124	7625	7625	4023297	0.1202	0.0442	0.2698	0.4408	0.0506	0.0334	0	0.0167	UN
1X2124	1X2124	8170	8170	4023297	0.1161	0.0444	0.2647	0.4485	0.0342	0.0471	0	0.0236	UN
1X2124	1X2124	8307	8307	4023297	0.115	0.0456	0.2642	0.45	0.0236	0.0335	0	0.0168	UN
1X2124	1X2124	8395	8395	4023297	0.1214	0.0438	0.273	0.4339	0.0548	0.0375	0	0.0188	UN
1X2231	1X2231	7311	7311	4023297	0.1096	0.0456	0.2656	0.4388	0.0298	0.0869	0.0023	0.0458	4th
1X2231	1X2231	7937	7937	4023297	0.1067	0.0454	0.2619	0.4295	0.029	0.0744	0	0.0372	UN
1X2231	1X2231	8307	8307	4023297	0.1095	0.0457	0.2656	0.4394	0.0296	0.0819	0.0048	0.0458	4th
1X2816	1X2816	6265	6265	4023297	0.1403	0.0001	0.3489	0.0009	0.2523	0.9975	0.0013	0.5001	PO
1X2816	1X2816	6716	6716	4023297	0.1402	0.0001	0.2603	0.0011	0.1234	0.9961	0.0019	0.5	PO
1X2816	1X2816	6955	6955	4023297	0.142	0.0001	0.3514	0.0011	0.2521	0.9993	0	0.4997	PO
1X2816	1X2816	7091	7091	4023297	0.1113	0.0468	0.2602	0.4861	0.0244	0.0227	0	0.0113	UN
1X2816	1X2816	7267	7267	4023297	0.1406	0.0001	0.346	0.0011	0.2486	0.9974	0	0.4987	PO
1X2816	1X2816	7311	7311	4023297	0.1396	0.0001	0.3586	0.001	0.2634	0.9971	0.0016	0.5001	PO
1X2816	1X2816	7625	7625	4023297	0.1411	0.0001	0.3501	0.0008	0.2524	0.9989	0	0.4995	PO
1X2816	1X2816	8170	8170	4023297	0.1407	0.0001	0.3585	0.0007	0.262	0.9893	0.0063	0.5009	PO
1X2816	1X2816	8307	8307	4023297	0.1412	0.0001	0.3645	0.001	0.2664	0.9878	0.0086	0.5025	PO
1X2816	1X2816	8395	8395	4023297	0.1405	0.0001	0.3479	0.0008	0.2509	0.9985	0	0.4993	PO
1X3656	1X3656	33863	33863	4023297	0.118	0.0337	0.277	0.3372	0.0696	0.2691	0	0.1346	3rd
1X3656	1X3656	34857	34857	4023297	0.1174	0.0341	0.2586	0.3468	0.0399	0.2697	0	0.1349	3rd
1X3656	1X3656	7091	7091	4023297	0.1098	0.0447	0.2603	0.4534	0.0227	0.0012	0	0.0006	UN
1X3656	1X3656	7937	7937	4023297	0.1075	0.0448	0.2651	0.438	0.0341	0.0289	0	0.0144	UN
1X3697	1X3697	33863	33863	4023297	0.1144	0.0445	0.2577	0.4462	0.0326	0.0157	0	0.0079	UN
1X3697	1X3697	34857	34857	4023297	0.1298	0.028	0.2847	0.2992	0.0966	0.4025	0	0.2013	2nd
1X3697	1X3697	6265	6265	4023297	0.1148	0.0436	0.2641	0.4526	0.0448	0.0012	0	0.0006	UN
1X3697	1X3697	6955	6955	4023297	0.1154	0.0439	0.2639	0.456	0.0419	0.0017	0	0.0009	UN
1X3697	1X3697	7091	7091	4023297	0.1364	0.0182	0.3332	0.2071	0.1815	0.5631	0	0.2816	2nd
1X3697	1X3697	7267	7267	4023297	0.1151	0.0443	0.2624	0.4599	0.0387	0	0	0	UN
1X3697	1X3697	7311	7311	4023297	0.11	0.0466	0.2583	0.4778	0.0251	0	0	0	UN
1X3697	1X3697	7625	7625	4023297	0.1161	0.0442	0.2671	0.4586	0.0439	0	0	0	UN
1X3697	1X3697	7937	7937	4023297	0.1089	0.0444	0.2603	0.4465	0.0245	0.0063	0	0.0032	UN
1X3697	1X3697	8170	8170	4023297	0.112	0.0458	0.2619	0.4811	0.0352	0.0028	0	0.0014	UN
1X3697	1X3697	8307	8307	4023297	0.1095	0.0463	0.257	0.4744	0.0248	0.0014	0	0.0007	UN
1X3697	1X3697	8395	8395	4023297	0.1154	0.0441	0.2649	0.4564	0.0423	0.0025	0	0.0012	UN
1X3796	1X3796	34568	34568	4023297	0.224	0.014	0.3632	0.1049	0.1942	0.5774	0.0074	0.2961	2nd
1X3796	1X3796	6716	6716	4023297	0.2211	0.0399	0.3317	0.3604	0.1345	0.0312	0	0.0156	UN
1X3796	1X3796	8344	8344	4023297	0.2178	0.0494	0.328	0.3288	0.1054	0.0325	0	0.0162	UN
1X3822	1X3822	33863	33863	4023297	0.1158	0.0462	0.259	0.4541	0.0332	0.0081	0	0.004	UN
1X3822	1X3822	34857	34857	4023297	0.1258	0.0331	0.2706	0.3422	0.0736	0.2707	0.0027	0.138	3rd
1X3822	1X3822	6265	6265	4023297	0.1164	0.0436	0.2659	0.4452	0.0514	0.0222	0	0.0111	UN
1X3822	1X3822	6955	6955	4023297	0.1177	0.0436	0.2674	0.4458	0.0509	0.0208	0	0.0104	UN
1X3822	1X3822	7091	7091	4023297	0.1372	0.0191	0.3317	0.2135	0.1787	0.5157	0.0162	0.2741	2nd
1X3822	1X3822	7267	7267	4023297	0.1179	0.0452	0.2676	0.4596	0.0445	0.0174	0	0.0087	UN
1X3822	1X3822	7311	7311	4023297	0.1127	0.0459	0.2632	0.4631	0.028	0.0438	0	0.0219	UN
1X3822	1X3822	7625	7625	4023297	0.1168	0.0444	0.2662	0.4521	0.0482	0.0253	0	0.0127	UN
1X3822	1X3822	8170	8170	4023297	0.1141	0.0445	0.2648	0.4607	0.039	0.0269	0	0.0134	UN
1X3822	1X3822	8307	8307	4023297	0.1131	0.0459	0.2647	0.4632	0.0282	0.0269	0	0.0134	UN
1X3822	1X3822	8395	8395	4023297	0.1177	0.0431	0.2683	0.4395	0.0538	0.0194	0	0.0097	UN
1X3837	1X3837	34568	34568	4023297	0.1999	0.0421	0.3086	0.301	0.0804	0.1256	0	0.0628	4th
1X3837	1X3837	6716	6716	4023297	0.2193	0.0408	0.3248	0.3748	0.1262	0.0154	0	0.0077	UN
1X3837	1X3837	7158	7158	4023297	0.2105	0.0318	0.338	0.1693	0.1177	0.4929	0	0.2464	2nd
1X3837	1X3837	8344	8344	4023297	0.2355	0.0332	0.3608	0.2277	0.1628	0.2757	0.0059	0.1438	3rd
1X4080	1X4080	6265	6265	4023297	0.1122	0.0459	0.2609	0.4479	0.0255	0.0187	0	0.0093	UN
1X4080	1X4080	6955	6955	4023297	0.1127	0.0451	0.2605	0.4407	0.0264	0.0171	0	0.0085	UN
1X4080	1X4080	7091	7091	4023297	0.1128	0.0435	0.2651	0.4317	0.039	0.0174	0	0.0087	UN
1X4080	1X4080	7267	7267	4023297	0.1138	0.0464	0.2631	0.4523	0.0227	0.0103	0	0.0051	UN
1X4080	1X4080	7311	7311	4023297	0.1409	0.0001	0.3636	0.001	0.2663	0.9845	0.012	0.5042	PO
1X4080	1X4080	7625	7625	4023297	0.1126	0.0456	0.2613	0.4454	0.026	0.0169	0	0.0084	UN
1X4080	1X4080	7937	7937	4023297	0.109	0.0453	0.2651	0.4321	0.0282	0.0266	0	0.0133	UN
1X4080	1X4080	8170	8170	4023297	0.1179	0.0324	0.2845	0.3321	0.0966	0.2792	0	0.1396	3rd
1X4080	1X4080	8307	8307	4023297	0.1394	0.0001	0.3588	0.001	0.2636	0.9916	0.0075	0.5033	PO
1X4080	1X4080	8395	8395	4023297	0.1136	0.0455	0.2641	0.4424	0.0281	0.026	0	0.013	UN
1X4179	1X4179	33863	33863	4023297	0.1126	0.0426	0.2601	0.4136	0.0261	0.0748	0	0.0374	UN
1X4179	1X4179	34857	34857	4023297	0.1239	0.0248	0.2757	0.2562	0.0898	0.4554	0.0012	0.2289	2nd
1X4179	1X4179	7091	7091	4023297	0.1121	0.0443	0.266	0.4466	0.0304	0.0013	0	0.0007	UN
1X4179	1X4179	7311	7311	4023297	0.1076	0.0464	0.2589	0.4566	0.0233	0.0093	0	0.0046	UN
1X4179	1X4179	7937	7937	4023297	0.1073	0.0447	0.2632	0.4327	0.0323	0.0203	0	0.0101	UN
1X4179	1X4179	8170	8170	4023297	0.1089	0.0458	0.2603	0.4613	0.0242	0.0157	0	0.0078	UN
1X4179	1X4179	8307	8307	4023297	0.1074	0.046	0.2584	0.4526	0.0251	0.0051	0	0.0026	UN
1X4209	1X4209	34568	34568	4023297	0.194	0.05	0.297	0.3578	0.0511	0.0125	0	0.0062	UN
1X4209	1X4209	6716	6716	4023297	0.2174	0.0404	0.3214	0.3744	0.1253	0.0122	0	0.0061	UN
1X4209	1X4209	8344	8344	4023297	0.2189	0.0473	0.3274	0.3215	0.1082	0.031	0	0.0155	UN
1X4777	1X4777	33631	33631	4023297	0.1166	0.025	0.2666	0.2321	0.1053	0.0616	0	0.0308	UN

FID1	ID1	FID2	ID2	N_SNP	HetHet	IBS0	HetConc	HomIBS0	Kinship	IBD1Seg	IBD2Seg	PropIBD	InfType
1X4777	1X4777	6265	6265	4023297	0.1138	0.0459	0.2642	0.4564	0.0304	0.0165	0	0.0083	UN
1X4777	1X4777	6955	6955	4023297	0.1152	0.0455	0.2663	0.4542	0.0314	0.0167	0	0.0083	UN
1X4777	1X4777	7091	7091	4023297	0.1138	0.0453	0.2664	0.456	0.036	0.0113	0	0.0057	UN
1X4777	1X4777	7267	7267	4023297	0.1147	0.0462	0.2643	0.4604	0.0269	0.0158	0	0.0079	UN
1X4777	1X4777	7311	7311	4023297	0.1121	0.0424	0.2678	0.4226	0.0497	0.0558	0	0.0279	UN
1X4777	1X4777	7625	7625	4023297	0.1136	0.0466	0.263	0.4633	0.0262	0.0179	0	0.009	UN
1X4777	1X4777	7937	7937	4023297	0.1093	0.045	0.2644	0.4373	0.0274	0.0299	0	0.0149	UN
1X4777	1X4777	8170	8170	4023297	0.1122	0.0436	0.2654	0.4431	0.045	0.0393	0	0.0196	UN
1X4777	1X4777	8307	8307	4023297	0.1131	0.0427	0.2712	0.4262	0.0499	0.0655	0	0.0327	UN
1X4777	1X4777	8395	8395	4023297	0.1149	0.0458	0.2663	0.4544	0.031	0.016	0	0.008	UN
25347	25347	6955	6955	4023297	0.1149	0.0495	0.2548	0.4492	0.0254	0.0038	0	0.0019	UN
25347	25347	7091	7091	4023297	0.1147	0.0483	0.2582	0.4429	0.0238	0.0025	0	0.0012	UN
25347	25347	8395	8395	4023297	0.1152	0.0499	0.2565	0.4514	0.0231	0.0063	0	0.0031	UN
25354	25354	33863	33863	4023297	0.1092	0.0371	0.2631	0.3375	0.0219	0.223	0	0.1115	3rd
26196	26196	33863	33863	4023297	0.1598	0.0002	0.3745	0.0018	0.2666	0.9938	0.0016	0.4985	PO
26196	26196	7267	7267	4023297	0.119	0.0459	0.257	0.4288	0.0327	0.0014	0	0.0007	UN
1X2124	1X2124	8780	8780	4023297	0.1186	0.0449	0.2683	0.4629	0.0416	0.0068	0	0.0034	UN
1X2124	1X2124	8995	8995	4023297	0.1281	0.0385	0.2899	0.3728	0.0878	0.017	0	0.0085	UN
1X2124	1X2124	9045	9045	4023297	0.116	0.0441	0.268	0.4331	0.0305	0.0604	0	0.0302	UN
1X2124	1X2124	9128	9128	4023297	0.1215	0.0437	0.2739	0.4372	0.0545	0.0377	0	0.0189	UN
1X2124	1X2124	9562	9562	4023297	0.1205	0.0423	0.2743	0.4252	0.0537	0.0221	0	0.0111	UN
1X2231	1X2231	8995	8995	4023297	0.1233	0.0253	0.2947	0.2471	0.1163	0.4349	0	0.2175	2nd
1X2231	1X2231	9045	9045	4023297	0.108	0.0471	0.2618	0.4493	0.0225	0.0769	0	0.0384	UN
1X2231	1X2231	9562	9562	4023297	0.1326	0.0143	0.3323	0.151	0.1865	0.7053	0	0.3527	2nd
1X2816	1X2816	8465	8465	4023297	0.141	0.0001	0.2792	0.001	0.1558	0.9981	0	0.4991	PO
1X2816	1X2816	8581	8581	4023297	0.1045	0.022	0.2419	0.1915	0.1077	0.0264	0	0.0132	UN
1X2816	1X2816	8780	8780	4023297	0.1114	0.0464	0.2604	0.4983	0.0257	0.0107	0	0.0054	UN
1X2816	1X2816	9045	9045	4023297	0.1389	0.0001	0.3577	0.0009	0.2622	0.9962	0.0028	0.501	PO
1X2816	1X2816	9128	9128	4023297	0.1396	0.0001	0.3454	0.0008	0.2499	0.9975	0.0014	0.5001	PO
1X2816	1X2816	9562	9562	4023297	0.1124	0.046	0.2639	0.4764	0.0304	0.0211	0	0.0106	UN
1X2816	1X2816	9841	9841	4023297	0.1079	0.0469	0.2574	0.4857	0.025	0.0125	0	0.0063	UN
1X3656	1X3656	8780	8780	4023297	0.1102	0.0449	0.2616	0.4665	0.0227	0	0	0	UN
1X3656	1X3656	9562	9562	4023297	0.1109	0.0424	0.2645	0.4288	0.0352	0	0	0	UN
1X3656	1X3656	9841	9841	4023297	0.1374	0.0001	0.3594	0.0014	0.2614	0.9937	0.0037	0.5006	PO
1X3697	1X3697	8653	8653	4023297	0.1112	0.016	0.2979	0.1466	0.1176	0.7502	0	0.3751	2nd
1X3697	1X3697	8780	8780	4023297	0.115	0.0415	0.267	0.4503	0.0564	0.0183	0	0.0092	UN
1X3697	1X3697	8995	8995	4023297	0.1145	0.0469	0.26	0.4631	0.0271	0	0	0	UN
1X3697	1X3697	9045	9045	4023297	0.1096	0.0463	0.2583	0.4704	0.0241	0.0014	0	0.0007	UN
1X3697	1X3697	9128	9128	4023297	0.1153	0.0443	0.265	0.4609	0.0423	0.0013	0	0.0007	UN
1X3697	1X3697	9562	9562	4023297	0.1143	0.0428	0.2657	0.4477	0.0513	0	0	0	UN
1X3697	1X3697	9841	9841	4023297	0.111	0.0434	0.2623	0.4541	0.0374	0.0032	0	0.0016	UN
1X3796	1X3796	8465	8465	4023297	0.1942	0.0438	0.2939	0.3982	0.0793	0.0119	0	0.006	UN
1X3796	1X3796	9656	9656	4023297	0.2225	0.0572	0.3315	0.2776	0.0968	0.0371	0	0.0186	UN
1X3796	1X3796	9860	9860	4023297	0.2035	0.0425	0.3107	0.3701	0.0962	0.0061	0	0.0031	UN
1X3822	1X3822	8653	8653	4023297	0.1141	0.0217	0.3037	0.1938	0.0915	0.6894	0.0096	0.3543	2nd
1X3822	1X3822	8780	8780	4023297	0.1175	0.0429	0.271	0.4565	0.0559	0.0166	0	0.0083	UN
1X3822	1X3822	8995	8995	4023297	0.1192	0.0436	0.2704	0.4267	0.0515	0.0629	0	0.0315	UN
1X3822	1X3822	9045	9045	4023297	0.1129	0.0457	0.2649	0.4588	0.0275	0.0355	0	0.0177	UN
1X3822	1X3822	9128	9128	4023297	0.1176	0.0435	0.2685	0.446	0.0528	0.0251	0	0.0126	UN
1X3822	1X3822	9562	9562	4023297	0.1203	0.0392	0.2802	0.4068	0.0738	0.1125	0	0.0562	4th
1X3822	1X3822	9841	9841	4023297	0.1117	0.0446	0.2615	0.456	0.0297	0.0141	0	0.007	UN
1X3837	1X3837	8465	8465	4023297	0.1978	0.0427	0.2978	0.3966	0.0824	0.0165	0	0.0083	UN
1X3837	1X3837	9656	9656	4023297	0.2264	0.0633	0.3358	0.3084	0.0829	0.024	0	0.012	UN
1X3837	1X3837	9860	9860	4023297	0.2013	0.0429	0.3032	0.3802	0.0879	0.0026	0	0.0013	UN
1X4080	1X4080	8780	8780	4023297	0.1121	0.0447	0.2628	0.4538	0.033	0.01	0	0.005	UN
1X4080	1X4080	8995	8995	4023297	0.1143	0.0456	0.2638	0.4282	0.0252	0.0436	0	0.0218	UN
1X4080	1X4080	9045	9045	4023297	0.1393	0.0001	0.3598	0.0007	0.2636	0.9873	0.0116	0.5053	PO
1X4080	1X4080	9128	9128	4023297	0.1131	0.0449	0.263	0.4411	0.0299	0.0206	0	0.0103	UN
1X4080	1X4080	9562	9562	4023297	0.1148	0.0404	0.2716	0.4017	0.0557	0.0784	0	0.0392	UN
1X4080	1X4080	9841	9841	4023297	0.1106	0.0447	0.2658	0.4403	0.0387	0.0173	0	0.0086	UN
1X4179	1X4179	8780	8780	4023297	0.1118	0.0439	0.2651	0.4538	0.0313	0	0	0	UN
1X4179	1X4179	9045	9045	4023297	0.1067	0.0465	0.2574	0.4552	0.023	0.007	0	0.0035	UN
1X4179	1X4179	9562	9562	4023297	0.1114	0.0433	0.2647	0.4336	0.0341	0.0029	0	0.0015	UN
1X4179	1X4179	9841	9841	4023297	0.1127	0.0366	0.2758	0.374	0.0729	0.1654	0	0.0827	4th
1X4209	1X4209	8465	8465	4023297	0.23	0.0311	0.3643	0.2978	0.1554	0.3352	0.0014	0.169	3rd
1X4209	1X4209	9656	9656	4023297	0.2249	0.0605	0.3332	0.2969	0.088	0.0203	0	0.0101	UN
1X4209	1X4209	9860	9860	4023297	0.2011	0.0421	0.3031	0.3777	0.09	0	0	0	UN
1X4739	1X4739	8653	8653	4023297	0.0813	0.0166	0.2963	0.1237	0.0454	0.3876	0.1081	0.3019	2nd
1X4777	1X4777	8780	8780	4023297	0.1125	0.0456	0.2627	0.4703	0.0326	0.0116	0	0.0058	UN
1X4777	1X4777	8995	8995	4023297	0.129	0.0223	0.3065	0.2266	0.1422	0.4786	0	0.2393	2nd
1X4777	1X4777	9045	9045	4023297	0.1118	0.0443	0.268	0.4376	0.0403	0.0344	0	0.0172	UN
1X4777	1X4777	9128	9128	4023297	0.1138	0.0454	0.2637	0.4529	0.0314	0.0143	0	0.0071	UN
1X4777	1X4777	9562	9562	4023297	0.1301	0.0241	0.3177	0.2568	0.1473	0.4394	0	0.2197	2nd
1X4777	1X4777	9841	9841	4023297	0.1118	0.0429	0.268	0.4327	0.0457	0.039	0	0.0195	UN
25347	25347	8780	8780	4023297	0.1142	0.0477	0.2568	0.4495	0.0249	0.0013	0	0.0006	UN
25347	25347	9128	9128	4023297	0.1142	0.0492	0.2541	0.447	0.0233	0.0056	0	0.0028	UN
25354	25354	9841	9841	4023297	0.1105	0.0291	0.2839	0.28	0.0836	0.2913	0	0.1456	3rd
25355	25355	9656	9656	4023297	0.1942	0.0126	0.3616	0.0501	0.184	0.437	0.0308	0.2493	2nd
25409	25409	8465	8465	4023297	0.1751	0.0063	0.3331	0.0502	0.2058	0.2913	0.0016	0.1472	3rd
26196	26196	8995	8995	4023297	0.1183	0.0479	0.2544	0.4283	0.0253	0.0043	0	0.0022	UN
26355	26355	27193	27193	4023297	0.1271	0.0377	0.278	0.2167	0.0621	0.092	0.05	0.096	3rd
26355	26355	27686	27686	4023297	0.1181	0.0353	0.2977	0.231	0.0631	0.1464	0.0387	0.1119	3rd
26498	26498	27351	27351	4023297	0.1619	0.0229	0.2837	0.2506	0.0642	0.1313	0	0.0657	4th
26800	26800	26980	26980	4023297	0.0821	0.0366	0.242	0.2724	0.0136	0.0455	0.024	0.0468	4th
26988	26988	27503	27503	4023297	0.1152	0.0182	0.3083	0.1719	0.1604	0.6481	0.0224	0.3464	2nd
27351	27351	27594	27594	4023297	0.111	0.0365	0.2792	0.2801	0.0119	0.0927	0.0106	0.0569	4th
26355	26355	27985	27985	4023297	0.112	0.0366	0.2414	0.2026	0.0462	0.084	0.04	0.082	4th
26498	26498	28279	28279	4023297	0.1732	0.021	0.2981	0.2477	0.1017	0.1321	0	0.066	4th
26988	26988	28246	28246	4023297	0.1513	0.0113	0.4312	0.1251	0.2505	0.6169	0.2321	0.5406	FS
27181	27181	28328	28328	4023297	0.0832	0.0372	0.2896	0.3043	0.0237	0.0219	0.0129	0.0238	UN
27193	27193	27985	27985	4023297	0.1378	0.0483	0.2911	0.2503	0.0616	0.1054	0.034	0.0867	4th
27306	27306	27989	27989	4023297	0.0789	0.0246	0.2561	0.1842	0.0395	0.0829	0.0242	0.0657	4th
27306	27306	28183	28183	4023297	0.1115	0.0087	0.4052	0.0735	0.221	0.3246	0.1523	0.3146	2nd
27351	27351	28279	28279	4023297	0.1887	0.0128	0.4633	0.1237	0.2652	0.524	0.2791	0.5411	FS
27503	27503	28246	28246	4023297	0.1115	0.029	0.2864	0.2799	0.0965	0.4431	0.0042	0.2257	2nd
27594	27594	28279	28279	4023297	0.1251	0.028	0.3087	0.2258	0.0576	0.1287	0.0053	0.0697	4th
26355	26355	28478	28478	4023297	0.1242	0.0401	0.2861	0.238	0.0719	0.0906	0.0339	0.0792	4th
26800	26800	28400	28400	4023297	0.0899	0.0289	0.3002	0.2313	0.0533	0.0878	0.0111	0.055	4th

FID1	ID1	FID2	ID2	N_SNP	HetHet	IBS0	HetConc	HomIBS0	Kinship	IBD1Seg	IBD2Seg	PropIBD	InfType
26800	26800	28416	28416	4023297	0.0937	0.0279	0.2986	0.2303	0.0854	0.0698	0.0281	0.063	4th
27190	27190	28478	28478	4023297	0.1314	0.0473	0.3113	0.2547	0.0547	0.1345	0.0339	0.1012	3rd
27193	27193	28478	28478	4023297	0.1274	0.0532	0.2731	0.2871	0.0141	0.0737	0.0089	0.0457	4th
27306	27306	28417	28417	4023297	0.0741	0.0263	0.2659	0.1943	0.0571	0.046	0.0741	0.0971	3rd
26498	26498	30104	30104	4023297	0.1571	0.034	0.2671	0.3707	0.0269	0.0013	0	0.0006	UN
26498	26498	30609	30609	4023297	0.1698	0.0376	0.2788	0.4007	0.0579	0.0024	0	0.0012	UN
26498	26498	30917	30917	4023297	0.2014	0.0167	0.3331	0.3042	0.174	0	0	0	UN
26498	26498	30920	30920	4023297	0.2127	0.0219	0.3292	0.3605	0.1848	0	0	0	UN
27351	27351	30917	30917	4023297	0.1364	0.0348	0.2667	0.3568	0.0523	0.0044	0	0.0022	UN
26498	26498	30933	30933	4023297	0.182	0.0337	0.2925	0.3832	0.099	0.0023	0	0.0012	UN
26498	26498	31011	31011	4023297	0.1778	0.0307	0.2874	0.3766	0.0986	0.0048	0	0.0024	UN
26498	26498	31021	31021	4023297	0.1771	0.0211	0.3028	0.2683	0.111	0.0339	0	0.0169	UN
26498	26498	31065	31065	4023297	0.179	0.0358	0.2849	0.3926	0.0906	0	0	0	UN
26498	26498	31145	31145	4023297	0.1652	0.029	0.2807	0.3755	0.0622	0.0099	0	0.005	UN
26988	26988	30961	30961	4023297	0.0971	0.0414	0.2503	0.3815	0.0245	0.0852	0	0.0426	UN
27351	27351	31021	31021	4023297	0.1196	0.042	0.2473	0.3598	0.0368	0.2516	0.0042	0.13	3rd
27503	27503	30961	30961	4023297	0.0984	0.041	0.2553	0.3737	0.03	0.0598	0	0.0299	UN
26498	26498	31284	31284	4023297	0.1635	0.0236	0.2839	0.2896	0.0689	0.0101	0	0.005	UN
26498	26498	31297	31297	4023297	0.1598	0.0234	0.2825	0.3036	0.0535	0.0355	0	0.0178	UN
26498	26498	31299	31299	4023297	0.1564	0.019	0.282	0.24	0.0533	0.024	0	0.012	UN
26498	26498	31331	31331	4023297	0.1858	0.021	0.3165	0.2565	0.1294	0.0241	0	0.0121	UN
26498	26498	31337	31337	4023297	0.174	0.0323	0.2825	0.3887	0.0854	0	0	0	UN
26498	26498	31364	31364	4023297	0.1614	0.0337	0.2701	0.3914	0.0446	0	0	0	UN
26498	26498	31408	31408	4023297	0.1697	0.0402	0.279	0.411	0.0493	0	0	0	UN
26498	26498	31410	31410	4023297	0.1724	0.0397	0.2807	0.3895	0.0598	0	0	0	UN
26498	26498	31417	31417	4023297	0.1628	0.0304	0.2755	0.3469	0.0538	0.0032	0	0.0016	UN
26498	26498	31477	31477	4023297	0.1709	0.0493	0.2786	0.3812	0.0278	0	0	0	UN
27351	27351	31331	31331	4023297	0.1202	0.0406	0.2431	0.3406	0.0328	0.0878	0.0034	0.0473	4th
26498	26498	31497	31497	4023297	0.1737	0.0344	0.279	0.3939	0.0819	0	0	0	UN
26498	26498	31498	31498	4023297	0.1827	0.0207	0.3102	0.2636	0.1251	0.0368	0	0.0184	UN
26498	26498	31525	31525	4023297	0.1653	0.0324	0.278	0.3189	0.0555	0	0	0	UN
26498	26498	31656	31656	4023297	0.169	0.0347	0.2757	0.3834	0.0668	0	0	0	UN
27351	27351	31498	31498	4023297	0.1201	0.0444	0.2435	0.3782	0.0207	0.1368	0.0074	0.0757	4th
26498	26498	31716	31716	4023297	0.1622	0.0339	0.2744	0.3762	0.0415	0	0	0	UN
26498	26498	31786	31786	4023297	0.158	0.0263	0.2773	0.3198	0.0419	0.0015	0	0.0007	UN
26498	26498	31800	31800	4023297	0.1712	0.0339	0.2896	0.3522	0.0615	0.0021	0	0.001	UN
26498	26498	31805	31805	4023297	0.1681	0.0315	0.2781	0.3761	0.0699	0.0021	0	0.0011	UN
26498	26498	31903	31903	4023297	0.176	0.0509	0.2758	0.4268	0.0486	0	0	0	UN
26498	26498	31952	31952	4023297	0.1493	0.0176	0.2749	0.212	0.0301	0.0123	0	0.0062	UN
26498	26498	31970	31970	4023297	0.1589	0.028	0.2757	0.3137	0.043	0.002	0	0.001	UN
26498	26498	32025	32025	4023297	0.1558	0.0223	0.2784	0.2893	0.043	0.0508	0	0.0254	UN
26498	26498	32043	32043	4023297	0.1577	0.0286	0.2739	0.3646	0.0374	0	0	0	UN
26498	26498	32089	32089	4023297	0.1816	0.0491	0.2888	0.4146	0.059	0.0041	0	0.002	UN
26498	26498	32187	32187	4023297	0.1726	0.0438	0.2773	0.4326	0.0528	0	0	0	UN
26498	26498	32224	32224	4023297	0.1768	0.0292	0.2937	0.361	0.0935	0.0017	0	0.0008	UN
26498	26498	32358	32358	4023297	0.1698	0.0398	0.2804	0.4095	0.0487	0	0	0	UN
26988	26988	32043	32043	4023297	0.1159	0.0289	0.2781	0.2772	0.0752	0.4816	0	0.2408	2nd
27351	27351	32025	32025	4023297	0.1081	0.0462	0.2408	0.3974	0.0128	0.1345	0.0014	0.0686	4th
27503	27503	32043	32043	4023297	0.1128	0.0362	0.2693	0.3388	0.038	0.3112	0	0.1556	3rd
26498	26498	32849	32849	4023297	0.1648	0.0286	0.2792	0.3623	0.0637	0	0	0	UN
26988	26988	32849	32849	4023297	0.1189	0.0279	0.2728	0.2684	0.0631	0.5216	0	0.2608	2nd
27503	27503	32849	32849	4023297	0.1136	0.0324	0.2583	0.3039	0.0329	0.3682	0	0.1841	2nd
26498	26498	33631	33631	4023297	0.1618	0.0253	0.2833	0.2833	0.0558	0.0046	0	0.0023	UN
26498	26498	33863	33863	4023297	0.1565	0.029	0.2718	0.3657	0.0333	0.0014	0	0.0007	UN
26498	26498	34568	34568	4023297	0.1805	0.0473	0.2855	0.4172	0.0635	0	0	0	UN
26498	26498	34857	34857	4023297	0.1676	0.0291	0.2831	0.376	0.0698	0.0053	0	0.0026	UN
26498	26498	6265	6265	4023297	0.1547	0.0281	0.2721	0.3711	0.0265	0.004	0	0.002	UN
26498	26498	6716	6716	4023297	0.2026	0.0297	0.3084	0.3806	0.1538	0.0017	0	0.0009	UN
26498	26498	6955	6955	4023297	0.1563	0.0281	0.274	0.3722	0.0321	0.0026	0	0.0013	UN
26498	26498	7091	7091	4023297	0.1542	0.0272	0.2725	0.3663	0.0258	0.0075	0	0.0037	UN
26498	26498	7267	7267	4023297	0.1571	0.0286	0.2753	0.3788	0.0324	0.0015	0	0.0007	UN
26498	26498	7625	7625	4023297	0.1547	0.0282	0.2714	0.3729	0.0271	0	0	0	UN
26498	26498	8344	8344	4023297	0.195	0.0534	0.2961	0.436	0.0851	0.0013	0	0.0007	UN
26498	26498	8395	8395	4023297	0.1558	0.0282	0.2736	0.3719	0.0295	0.0026	0	0.0013	UN
26988	26988	33863	33863	4023297	0.1178	0.0252	0.2845	0.243	0.0952	0.5204	0	0.2602	2nd
27166	27166	7937	7937	4023297	0.0961	0.021	0.299	0.1718	0.021	0.3658	0	0.1829	2nd
27181	27181	8170	8170	4023297	0.1037	0.0125	0.296	0.1192	0.1002	0.1466	0	0.0733	4th
27472	27472	8170	8170	4023297	0.1012	0.0068	0.3159	0.06	0.0977	0.4875	0.0044	0.2481	2nd
27503	27503	33863	33863	4023297	0.1128	0.0363	0.27	0.336	0.0388	0.3351	0	0.1676	3rd
27592	27592	8170	8170	4023297	0.1139	0.0077	0.3577	0.0694	0.1412	0.6431	0.0049	0.3265	2nd
26498	26498	8465	8465	4023297	0.2098	0.0237	0.3399	0.3132	0.1709	0	0	0	UN
26498	26498	8780	8780	4023297	0.1529	0.0269	0.2696	0.378	0.0245	0.0041	0	0.0021	UN
26498	26498	8995	8995	4023297	0.1677	0.0197	0.2987	0.2512	0.0835	0.0387	0	0.0194	UN
26498	26498	9128	9128	4023297	0.1554	0.0286	0.2731	0.3791	0.027	0.0022	0	0.0011	UN
26498	26498	9562	9562	4023297	0.1678	0.0158	0.3046	0.2183	0.0916	0.1482	0	0.0741	4th
26498	26498	9860	9860	4023297	0.1934	0.0283	0.3036	0.3452	0.1381	0.0087	0	0.0043	UN
26988	26988	9841	9841	4023297	0.1306	0.0193	0.3463	0.202	0.1699	0.5275	0.0866	0.3504	2nd
27033	27033	9656	9656	4023297	0.1611	0.0438	0.2825	0.1938	0.0304	0.0104	0	0.0052	UN
27230	27230	9562	9562	4023297	0.1054	0.0164	0.2734	0.1452	0.1028	0.0385	0	0.0193	UN
27351	27351	8995	8995	4023297	0.1129	0.0423	0.2465	0.3602	0.0467	0.2137	0	0.1068	3rd
27351	27351	9562	9562	4023297	0.1165	0.032	0.2623	0.2907	0.0833	0.4168	0	0.2084	2nd
27433	27433	8465	8465	4023297	0.1368	0.0144	0.2645	0.0987	0.0984	0.1596	0	0.0798	4th
27503	27503	9841	9841	4023297	0.1146	0.0275	0.2924	0.2695	0.1031	0.4272	0	0.2136	2nd
27525	27525	8780	8780	4023297	0.0924	0.0168	0.2659	0.1425	0.0135	0.3805	0	0.1902	2nd
27643	27643	8465	8465	4023297	0.14	0.0168	0.2762	0.1178	0.0915	0.1717	0	0.0859	4th
27668	27668	9656	9656	4023297	0.149	0.0255	0.292	0.1019	0.0158	0.47	0	0.235	2nd
27885	27885	27908	27908	4023297	0.1744	0.0063	0.5945	0.0299	0.3405	0.1688	0.2256	0.31	2nd
27989	27989	28183	28183	4023297	0.0938	0.0207	0.2896	0.1682	0.1251	0.204	0.0516	0.1536	3rd
27985	27985	28478	28478	4023297	0.1225	0.044	0.2646	0.2337	0.0453	0.0632	0.0314	0.063	4th
27989	27989	28417	28417	4023297	0.0797	0.0284	0.2619	0.2162	0.0163	0.076	0.0348	0.0728	4th
27999	27999	28635	28635	4023297	0.0843	0.0147	0.2507	0.1116	0.0218	0.3475	0.0755	0.2493	2nd
28183	28183	28417	28417	4023297	0.0805	0.0198	0.2654	0.1527	0.0679	0.1483	0.0474	0.1216	3rd
28246	28246	30917	30917	4023297	0.1277	0.0321	0.2607	0.4023	0.0228	0	0	0	UN
28279	28279	30917	30917	4023297	0.1444	0.0317	0.2749	0.3473	0.0892	0.0043	0	0.0021	UN
28285	28285	30104	30104	4023297	0.1342	0.0281	0.3455	0.231	0.0895	0.0531	0	0.0265	UN
28246	28246	30961	30961	4023297	0.1032	0.0402	0.2621	0.3945	0.0297	0.1553	0	0.0777	4th
28279	28279	31021	31021	4023297	0.1239	0.0398	0.2472	0.3576	0.0657	0.2167	0	0.1084	3rd
28279	28279	31284	31284	4023297	0.1133	0.0462	0.2314	0.4029	0.023	0.0653	0	0.0326	UN

FID1	ID1	FID2	ID2	N_SNP	HetHet	IBS0	HetConc	HomIBS0	Kinship	IBD1Seg	IBD2Seg	PropIBD	InfType
28279	28279	31331	31331	4023297	0.1252	0.0402	0.2448	0.3506	0.0575	0.1062	0	0.0531	4th
28279	28279	31498	31498	4023297	0.1299	0.0384	0.2571	0.3471	0.0719	0.1805	0.0019	0.0922	3rd
28246	28246	32043	32043	4023297	0.1209	0.0277	0.2853	0.2846	0.0977	0.4525	0	0.2262	2nd
28279	28279	32025	32025	4023297	0.1173	0.0439	0.2544	0.3974	0.0184	0.1379	0.0026	0.0715	4th
28246	28246	32849	32849	4023297	0.1224	0.0292	0.2758	0.2986	0.0734	0.4533	0	0.2267	2nd
27889	27889	7937	7937	4023297	0.113	0.0113	0.3463	0.103	0.1472	0.425	0.0211	0.2336	2nd
27989	27989	7091	7091	4023297	0.1051	0.0314	0.2778	0.2816	0.0232	0.2703	0.0071	0.1423	3rd
28076	28076	7311	7311	4023297	0.1061	0.0351	0.2601	0.321	0.0577	0.1066	0.0044	0.0577	4th
28076	28076	8170	8170	4023297	0.1306	0.0086	0.3367	0.0875	0.2085	0.2729	0.0145	0.1509	3rd
28076	28076	8307	8307	4023297	0.1016	0.0398	0.2465	0.3568	0.0301	0.0567	0	0.0284	UN
28183	28183	7091	7091	4023297	0.1045	0.0254	0.276	0.2252	0.0501	0.4781	0	0.2391	2nd
28246	28246	33863	33863	4023297	0.125	0.0216	0.2984	0.2246	0.1301	0.5641	0	0.282	2nd
28246	28246	34857	34857	4023297	0.114	0.0384	0.2492	0.387	0.0166	0.2338	0	0.1169	3rd
28281	28281	7158	7158	4023297	0.1443	0.0339	0.281	0.1449	0.0864	0.2601	0.0347	0.1647	3rd
28304	28304	7311	7311	4023297	0.104	0.0291	0.2921	0.2574	0.0293	0.2467	0.0242	0.1475	3rd
28304	28304	8170	8170	4023297	0.1322	0.0025	0.3984	0.0253	0.2321	0.4982	0.1131	0.3622	FS
28328	28328	8170	8170	4023297	0.1045	0.0116	0.2993	0.1116	0.1072	0.1625	0	0.0813	4th
27814	27814	8465	8465	4023297	0.1288	0.0157	0.2684	0.1028	0.0447	0.196	0	0.098	3rd
27841	27841	9656	9656	4023297	0.1687	0.0161	0.3319	0.0629	0.1066	0.5276	0.0201	0.2839	2nd
27885	27885	9656	9656	4023297	0.1516	0.0151	0.3065	0.0606	0.0541	0.5365	0.019	0.2872	2nd
27908	27908	9656	9656	4023297	0.1633	0.0122	0.3276	0.0498	0.1025	0.5198	0.053	0.3129	2nd
27989	27989	8653	8653	4023297	0.0872	0.0323	0.2602	0.2499	0.0492	0.2526	0.0576	0.1839	2nd
27999	27999	8653	8653	4023297	0.078	0.02	0.2622	0.1481	0.0389	0.3092	0.0638	0.2184	2nd
28021	28021	9860	9860	4023297	0.1579	0.0113	0.3276	0.0988	0.1383	0.068	0	0.034	UN
28102	28102	8780	8780	4023297	0.0944	0.0114	0.297	0.095	0.0125	0.5221	0	0.2611	2nd
28183	28183	8653	8653	4023297	0.0879	0.0278	0.2629	0.2132	0.072	0.5189	0.041	0.3004	2nd
28246	28246	9841	9841	4023297	0.1359	0.0136	0.3544	0.1539	0.2063	0.6455	0.0822	0.405	FS
28259	28259	8465	8465	4023297	0.1536	0.0119	0.3091	0.0944	0.137	0.1356	0	0.0678	4th
28279	28279	8995	8995	4023297	0.1185	0.0416	0.25	0.3707	0.04	0.1729	0	0.0865	4th
28279	28279	9562	9562	4023297	0.1212	0.032	0.2628	0.305	0.0719	0.3657	0	0.1829	2nd
28285	28285	9860	9860	4023297	0.1377	0.0167	0.2928	0.1365	0.0527	0.0161	0	0.0081	UN
28386	28386	29156	29156	4023297	0.1029	0.0407	0.306	0.2918	0.0251	0	0	0	UN
28576	28576	8170	8170	4023297	0.0973	0.0054	0.335	0.047	0.0502	0.7265	0.0014	0.3646	2nd
28635	28635	34857	34857	4023297	0.1111	0.0379	0.2409	0.314	0.014	0.3895	0	0.1948	2nd
28635	28635	7091	7091	4023297	0.109	0.0163	0.2574	0.1447	0.1322	0.7061	0.0027	0.3558	2nd
28635	28635	8653	8653	4023297	0.1209	0.0109	0.345	0.0923	0.1799	0.5962	0.2816	0.5797	FS
28949	28949	9860	9860	4023297	0.1387	0.0177	0.2949	0.1416	0.0513	0.0228	0	0.0114	UN
30104	30104	30917	30917	4023297	0.135	0.0409	0.257	0.4256	0.0379	0	0	0	UN
30609	30609	30917	30917	4023297	0.1405	0.0441	0.2538	0.4483	0.0581	0	0	0	UN
30609	30609	30920	30920	4023297	0.1577	0.0441	0.2678	0.4278	0.0443	0	0	0	UN
30917	30917	30920	30920	4023297	0.1783	0.0253	0.2992	0.3839	0.1406	0	0	0	UN
30609	30609	31011	31011	4023297	0.1381	0.0561	0.2528	0.4615	0.0258	0.0049	0	0.0024	UN
30609	30609	31021	31021	4023297	0.1384	0.0364	0.2707	0.3143	0.0902	0.0454	0	0.0227	UN
30609	30609	31028	31028	4023297	0.1646	0.0352	0.3319	0.266	0.1391	0.1114	0.0019	0.0576	4th
30609	30609	31065	31065	4023297	0.1437	0.0573	0.2606	0.4421	0.0223	0.0016	0	0.0008	UN
30609	30609	31145	31145	4023297	0.138	0.0337	0.2741	0.3022	0.094	0.1166	0.0135	0.0719	4th
30611	30611	31145	31145	4023297	0.1405	0.0193	0.3484	0.1985	0.1397	0.2812	0.006	0.1466	3rd
30611	30611	31234	31234	4023297	0.1064	0.036	0.2681	0.3146	0.0391	0.0015	0	0.0007	UN
30614	30614	31021	31021	4023297	0.1563	0.0205	0.3714	0.1994	0.1682	0.2445	0.0015	0.1237	3rd
30614	30614	31145	31145	4023297	0.132	0.0284	0.302	0.2585	0.0991	0.0304	0	0.0152	UN
30614	30614	31234	31234	4023297	0.1129	0.0392	0.2716	0.3323	0.0601	0.0012	0	0.0006	UN
30917	30917	30933	30933	4023297	0.143	0.0419	0.2481	0.4508	0.081	0	0	0	UN
30917	30917	31011	31011	4023297	0.1492	0.0349	0.2652	0.4063	0.1062	0	0	0	UN
30917	30917	31016	31016	4023297	0.1317	0.0237	0.2819	0.2735	0.0481	0	0	0	UN
30917	30917	31021	31021	4023297	0.1501	0.029	0.2849	0.3462	0.1105	0	0	0	UN
30917	30917	31065	31065	4023297	0.146	0.0418	0.2534	0.4368	0.0858	0	0	0	UN
30917	30917	31145	31145	4023297	0.1432	0.0321	0.2725	0.3929	0.0854	0	0	0	UN
30920	30920	30933	30933	4023297	0.1667	0.0417	0.2754	0.4264	0.0778	0	0	0	UN
30920	30920	31011	31011	4023297	0.1607	0.0403	0.2662	0.4396	0.0697	0	0	0	UN
30920	30920	31021	31021	4023297	0.1562	0.0379	0.2724	0.4196	0.0503	0	0	0	UN
30920	30920	31065	31065	4023297	0.1737	0.0409	0.2888	0.407	0.0914	0	0	0	UN
30920	30920	31145	31145	4023297	0.1507	0.0349	0.2642	0.4015	0.0459	0	0	0	UN
30609	30609	31337	31337	4023297	0.134	0.0566	0.2465	0.4609	0.0231	0.0141	0	0.007	UN
30917	30917	31284	31284	4023297	0.1342	0.0354	0.2578	0.4049	0.0513	0	0	0	UN
30917	30917	31297	31297	4023297	0.1349	0.0331	0.2669	0.3994	0.0505	0	0	0	UN
30917	30917	31331	31331	4023297	0.1502	0.0328	0.2792	0.3732	0.1038	0.0048	0	0.0024	UN
30917	30917	31337	31337	4023297	0.138	0.0391	0.2433	0.4436	0.0751	0	0	0	UN
30917	30917	31364	31364	4023297	0.1376	0.0399	0.2565	0.4385	0.0547	0	0	0	UN
30917	30917	31408	31408	4023297	0.1428	0.0476	0.2595	0.4648	0.0503	0	0	0	UN
30917	30917	31410	31410	4023297	0.1394	0.0496	0.2478	0.4646	0.0446	0	0	0	UN
30917	30917	31417	31417	4023297	0.1344	0.039	0.2515	0.421	0.0489	0	0	0	UN
30920	30920	31331	31331	4023297	0.1528	0.0416	0.2597	0.4387	0.0408	0	0	0	UN
30920	30920	31337	31337	4023297	0.1648	0.0344	0.278	0.3763	0.0893	0.0021	0	0.0011	UN
30920	30920	31364	31364	4023297	0.1547	0.0359	0.2703	0.3809	0.0524	0	0	0	UN
30920	30920	31408	31408	4023297	0.1576	0.0461	0.2679	0.43	0.0375	0	0	0	UN
30920	30920	31410	31410	4023297	0.1698	0.0374	0.2904	0.3421	0.0863	0	0	0	UN
30920	30920	31462	31462	4023297	0.1504	0.0346	0.277	0.3576	0.026	0	0	0	UN
30609	30609	31497	31497	4023297	0.139	0.0537	0.2551	0.429	0.0345	0.0107	0	0.0053	UN
30609	30609	31498	31498	4023297	0.1436	0.0359	0.2784	0.3104	0.1046	0.051	0	0.0255	UN
30609	30609	31560	31560	4023297	0.1788	0.0314	0.3818	0.2725	0.1691	0.1553	0.029	0.1067	3rd
30614	30614	31498	31498	4023297	0.1669	0.0202	0.3972	0.2005	0.1799	0.2069	0.0019	0.1053	3rd
30907	30907	31507	31507	4023297	0.1162	0.0504	0.2911	0.3355	0.0249	0	0.0053	0.0053	UN
30917	30917	31497	31497	4023297	0.1458	0.0357	0.2579	0.3921	0.0989	0	0	0	UN
30917	30917	31498	31498	4023297	0.1541	0.0287	0.2894	0.3448	0.1217	0.0012	0	0.0006	UN
30917	30917	31542	31542	4023297	0.1494	0.0224	0.3218	0.2409	0.1005	0.0104	0	0.0052	UN
30917	30917	31560	31560	4023297	0.1355	0.0444	0.2515	0.4436	0.0368	0	0	0	UN
30917	30917	31656	31656	4023297	0.14	0.0423	0.2514	0.4433	0.0643	0	0	0	UN
30920	30920	31497	31497	4023297	0.1665	0.0382	0.2787	0.3977	0.0836	0	0	0	UN
30920	30920	31498	31498	4023297	0.1566	0.0386	0.2687	0.426	0.055	0	0	0	UN
30920	30920	31656	31656	4023297	0.1586	0.0433	0.2684	0.4304	0.0499	0	0	0	UN
30609	30609	31800	31800	4023297	0.1456	0.0368	0.2884	0.287	0.1004	0.0096	0.002	0.0068	UN
30609	30609	31966	31966	4023297	0.1302	0.0398	0.2749	0.3325	0.0352	0.0417	0	0.0209	UN
30611	30611	31720	31720	4023297	0.12	0.0343	0.3168	0.3	0.0802	0.0501	0.0015	0.0265	UN
30614	30614	31966	31966	4023297	0.1122	0.0425	0.2677	0.3533	0.0432	0.0016	0	0.0008	UN
30917	30917	31716	31716	4023297	0.1279	0.0435	0.2367	0.457	0.0235	0	0	0	UN
30917	30917	31786	31786	4023297	0.1351	0.0338	0.266	0.3876	0.0501	0	0	0	UN
30917	30917	31800	31800	4023297	0.1384	0.0427	0.2567	0.4212	0.0492	0	0	0	UN
30917	30917	31805	31805	4023297	0.1405	0.0387	0.2568	0.4361	0.0706	0	0	0	UN

FID1	ID1	FID2	ID2	N_SNP	HetHet	IBS0	HetConc	HomIBS0	Kinship	IBD1Seg	IBD2Seg	PropIBD	InfType
30917	30917	31970	31970	4023297	0.1423	0.034	0.28	0.3613	0.0671	0	0	0	UN
30920	30920	31716	31716	4023297	0.1579	0.0361	0.2802	0.3665	0.0533	0	0	0	UN
30920	30920	31805	31805	4023297	0.1593	0.0347	0.2741	0.3752	0.0713	0	0	0	UN
30920	30920	31846	31846	4023297	0.1573	0.0411	0.2806	0.3616	0.034	0	0	0	UN
30920	30920	31903	31903	4023297	0.1742	0.0511	0.2865	0.4018	0.0674	0	0	0	UN
30609	30609	32089	32089	4023297	0.1554	0.0399	0.2863	0.2728	0.0897	0.0564	0	0.0282	UN
30609	30609	32358	32358	4023297	0.1464	0.0375	0.2835	0.2906	0.1054	0.0129	0	0.0065	UN
30611	30611	32467	32467	4023297	0.1099	0.0332	0.3263	0.2787	0.074	0.0271	0	0.0136	UN
30614	30614	32039	32039	4023297	0.1294	0.0311	0.3523	0.2646	0.1157	0.0262	0.0032	0.0163	UN
30917	30917	32025	32025	4023297	0.1352	0.0292	0.2729	0.3548	0.0582	0.0016	0	0.0008	UN
30917	30917	32040	32040	4023297	0.137	0.0348	0.2676	0.4101	0.0542	0.0013	0	0.0007	UN
30917	30917	32043	32043	4023297	0.1365	0.0341	0.2666	0.4098	0.0551	0	0	0	UN
30917	30917	32089	32089	4023297	0.1519	0.058	0.2649	0.473	0.047	0	0	0	UN
30917	30917	32187	32187	4023297	0.143	0.0493	0.2522	0.4666	0.0556	0	0	0	UN
30917	30917	32224	32224	4023297	0.1434	0.0357	0.2604	0.4168	0.0875	0	0	0	UN
30917	30917	32358	32358	4023297	0.1458	0.0462	0.2679	0.4553	0.0576	0	0	0	UN
30920	30920	32089	32089	4023297	0.1681	0.053	0.2755	0.4168	0.0517	0	0	0	UN
30920	30920	32187	32187	4023297	0.1616	0.0493	0.2688	0.4453	0.0445	0	0	0	UN
30920	30920	32224	32224	4023297	0.1711	0.0317	0.2974	0.353	0.1017	0	0	0	UN
30920	30920	32358	32358	4023297	0.1574	0.0462	0.2689	0.4327	0.0351	0	0	0	UN
30611	30611	33083	33083	4023297	0.1167	0.0294	0.3168	0.2659	0.1083	0.0433	0	0.0216	UN
30614	30614	32995	32995	4023297	0.1128	0.0353	0.3149	0.2861	0.0402	0.0139	0.0062	0.0132	UN
30917	30917	32849	32849	4023297	0.1438	0.034	0.2731	0.4047	0.0812	0	0	0	UN
30920	30920	32849	32849	4023297	0.15	0.0369	0.2619	0.4151	0.0391	0	0	0	UN
30609	30609	34568	34568	4023297	0.1578	0.0306	0.2909	0.2177	0.1197	0.0555	0	0.0278	UN
30609	30609	34857	34857	4023297	0.151	0.0315	0.3039	0.2843	0.1249	0.1145	0.01	0.0672	4th
30611	30611	34857	34857	4023297	0.141	0.0213	0.3446	0.2165	0.1254	0.2338	0	0.1169	3rd
30614	30614	34857	34857	4023297	0.1338	0.0291	0.3031	0.2645	0.0937	0.0184	0	0.0092	UN
30917	30917	33631	33631	4023297	0.1293	0.0399	0.2493	0.4172	0.0223	0	0	0	UN
30917	30917	33863	33863	4023297	0.1377	0.0343	0.2702	0.4097	0.056	0	0	0	UN
30917	30917	33908	33908	4023297	0.1298	0.0366	0.2554	0.4182	0.027	0	0	0	UN
30917	30917	34568	34568	4023297	0.1573	0.0546	0.2758	0.4665	0.0621	0.002	0	0.001	UN
30917	30917	34857	34857	4023297	0.1461	0.0312	0.2763	0.3827	0.0963	0.0021	0	0.001	UN
30917	30917	6265	6265	4023297	0.1401	0.0299	0.2811	0.3759	0.0699	0	0	0	UN
30917	30917	6716	6716	4023297	0.166	0.0332	0.2726	0.4008	0.1011	0.0033	0	0.0017	UN
30917	30917	6955	6955	4023297	0.1396	0.0314	0.278	0.3934	0.0662	0.0013	0	0.0006	UN
30917	30917	7091	7091	4023297	0.142	0.0264	0.2879	0.34	0.0838	0	0	0	UN
30917	30917	7267	7267	4023297	0.1411	0.0304	0.2812	0.382	0.0728	0	0	0	UN
30917	30917	7311	7311	4023297	0.1361	0.0303	0.2783	0.378	0.0515	0.0071	0	0.0036	UN
30917	30917	7625	7625	4023297	0.1418	0.0286	0.2846	0.3605	0.0787	0.0027	0	0.0014	UN
30917	30917	8170	8170	4023297	0.137	0.0306	0.2784	0.3916	0.0554	0.0065	0	0.0032	UN
30917	30917	8307	8307	4023297	0.1363	0.03	0.2792	0.3745	0.0528	0.0095	0	0.0048	UN
30917	30917	8395	8395	4023297	0.1395	0.0309	0.2783	0.3859	0.0665	0.0034	0	0.0017	UN
30920	30920	34568	34568	4023297	0.1676	0.052	0.2735	0.4247	0.0552	0	0	0	UN
30920	30920	34857	34857	4023297	0.155	0.0346	0.2707	0.3987	0.0577	0	0	0	UN
30920	30920	6716	6716	4023297	0.1906	0.0364	0.2993	0.4128	0.1421	0	0	0	UN
30920	30920	8344	8344	4023297	0.1817	0.0573	0.2841	0.4366	0.0789	0	0	0	UN
29565	29565	9860	9860	4023297	0.1627	0.0185	0.3279	0.1337	0.1286	0.0308	0	0.0154	UN
29758	29758	9860	9860	4023297	0.139	0.0159	0.2932	0.132	0.0634	0.0206	0.0012	0.0115	UN
30104	30104	9860	9860	4023297	0.1904	0.0065	0.3855	0.0596	0.2251	0.1971	0	0.0986	3rd
30320	30320	9860	9860	4023297	0.1327	0.0197	0.2807	0.1524	0.0256	0.011	0	0.0055	UN
30609	30609	9860	9860	4023297	0.1509	0.0463	0.2661	0.3851	0.0491	0.0677	0	0.0338	UN
30611	30611	9841	9841	4023297	0.1014	0.0318	0.2557	0.3001	0.0519	0.0611	0	0.0306	UN
30749	30749	9860	9860	4023297	0.1347	0.019	0.2862	0.1485	0.0329	0.0218	0	0.0109	UN
30917	30917	8465	8465	4023297	0.1631	0.0292	0.2817	0.3622	0.1308	0.0016	0	0.0008	UN
30917	30917	8780	8780	4023297	0.1346	0.0311	0.2689	0.4101	0.0533	0	0	0	UN
30917	30917	8995	8995	4023297	0.1428	0.0301	0.2848	0.357	0.0778	0	0	0	UN
30917	30917	9045	9045	4023297	0.1364	0.0282	0.2801	0.3492	0.0588	0.0045	0	0.0022	UN
30917	30917	9128	9128	4023297	0.1414	0.0289	0.2837	0.3651	0.0767	0.0031	0	0.0016	UN
30917	30917	9562	9562	4023297	0.143	0.0266	0.2912	0.3395	0.0843	0.0042	0	0.0021	UN
30917	30917	9841	9841	4023297	0.1303	0.0324	0.2642	0.4077	0.0312	0	0	0	UN
30917	30917	9860	9860	4023297	0.1589	0.0366	0.2709	0.4195	0.1019	0	0	0	UN
30920	30920	8465	8465	4023297	0.1778	0.0359	0.2882	0.4144	0.118	0	0	0	UN
30920	30920	8780	8780	4023297	0.149	0.0204	0.2766	0.2616	0.0704	0.0012	0	0.0006	UN
30920	30920	9656	9656	4023297	0.1877	0.0826	0.2907	0.4327	0.0231	0	0	0	UN
30920	30920	9860	9860	4023297	0.1786	0.037	0.2884	0.4008	0.1175	0	0	0	UN
30933	30933	31011	31011	4023297	0.1489	0.046	0.2656	0.4047	0.0756	0.0033	0	0.0017	UN
30933	30933	31065	31065	4023297	0.165	0.0312	0.297	0.2729	0.1408	0.0013	0	0.0007	UN
31011	31011	31021	31021	4023297	0.135	0.0494	0.2537	0.4538	0.0297	0.0122	0	0.0061	UN
31011	31011	31065	31065	4023297	0.1444	0.05	0.2541	0.4289	0.0555	0.0031	0	0.0016	UN
31021	31021	31028	31028	4023297	0.145	0.0419	0.2909	0.322	0.0879	0.0115	0	0.0058	UN
31021	31021	31145	31145	4023297	0.1466	0.0286	0.3071	0.2855	0.1386	0.3448	0	0.1724	3rd
31021	31021	31234	31234	4023297	0.1368	0.028	0.3061	0.2654	0.1055	0.0236	0.0015	0.0133	UN
31028	31028	31145	31145	4023297	0.1411	0.0375	0.2856	0.2966	0.0919	0.0521	0	0.026	UN
31145	31145	31234	31234	4023297	0.1269	0.0294	0.2831	0.2832	0.0893	0.0446	0	0.0223	UN
30933	30933	31337	31337	4023297	0.1499	0.0509	0.271	0.4348	0.0595	0	0	0	UN
30933	30933	31408	31408	4023297	0.1414	0.0534	0.2572	0.4154	0.0322	0.003	0	0.0015	UN
31011	31011	31331	31331	4023297	0.1368	0.0494	0.2526	0.4414	0.0403	0.0093	0	0.0047	UN
31011	31011	31337	31337	4023297	0.1549	0.0429	0.2866	0.3913	0.0957	0.0181	0	0.009	UN
31011	31011	31408	31408	4023297	0.1488	0.0474	0.2783	0.3908	0.0671	0.0251	0	0.0125	UN
31011	31011	31410	31410	4023297	0.134	0.0568	0.2401	0.4452	0.0228	0.0057	0	0.0029	UN
31011	31011	31417	31417	4023297	0.1362	0.0495	0.2605	0.4288	0.0257	0.0039	0	0.002	UN
31021	31021	31284	31284	4023297	0.1178	0.0407	0.2393	0.3781	0.0431	0.0135	0	0.0068	UN
31021	31021	31331	31331	4023297	0.1261	0.0429	0.2435	0.3933	0.0546	0.0159	0	0.0079	UN
31021	31021	31337	31337	4023297	0.132	0.0504	0.2498	0.4575	0.027	0.0127	0	0.0064	UN
31021	31021	31417	31417	4023297	0.1283	0.0476	0.2587	0.4219	0.0473	0.0223	0.0013	0.0124	UN
31065	31065	31337	31337	4023297	0.1432	0.0564	0.2544	0.4679	0.0316	0	0	0	UN
31134	31134	31284	31284	4023297	0.1096	0.0302	0.2564	0.2787	0.0488	0.0074	0	0.0037	UN
30933	30933	31497	31497	4023297	0.1576	0.0472	0.2858	0.3956	0.0843	0.0031	0	0.0016	UN
30933	30933	31525	31525	4023297	0.1623	0.0429	0.3177	0.3272	0.0863	0.0028	0	0.0014	UN
30933	30933	31656	31656	4023297	0.1526	0.0426	0.2813	0.3604	0.0834	0.009	0	0.0045	UN
30961	30961	31500	31500	4023297	0.1032	0.0345	0.2729	0.3338	0.0697	0.003	0	0.0015	UN
31011	31011	31497	31497	4023297	0.1477	0.0478	0.2668	0.4189	0.0741	0.0071	0	0.0035	UN
31011	31011	31498	31498	4023297	0.1413	0.0492	0.2638	0.4522	0.0467	0.0186	0	0.0093	UN
31011	31011	31656	31656	4023297	0.1352	0.0482	0.245	0.4156	0.0467	0.0097	0	0.0049	UN
31021	31021	31497	31497	4023297	0.1361	0.0455	0.2565	0.404	0.0443	0.0252	0	0.0126	UN
31021	31021	31498	31498	4023297	0.2149	0.0086	0.5029	0.1019	0.3049	0.2895	0.2708	0.4156	FS
31021	31021	31560	31560	4023297	0.1273	0.0422	0.2534	0.3563	0.0664	0.0206	0	0.0103	UN

FID1	ID1	FID2	ID2	N_SNP	HetHet	IBS0	HetConc	HomIBS0	Kinship	IBD1Seg	IBD2Seg	PropIBD	InfType
31028	31028	31498	31498	4023297	0.1482	0.0408	0.2935	0.3131	0.1014	0.0137	0	0.0069	UN
31028	31028	31560	31560	4023297	0.1501	0.0407	0.3061	0.3033	0.0988	0.0797	0.0019	0.0417	UN
31065	31065	31497	31497	4023297	0.1411	0.0587	0.247	0.471	0.0257	0	0	0	UN
31065	31065	31656	31656	4023297	0.1446	0.0522	0.2612	0.422	0.0408	0	0	0	UN
31124	31124	31500	31500	4023297	0.0958	0.0373	0.2541	0.3439	0.0356	0.0109	0	0.0054	UN
31124	31124	31624	31624	4023297	0.1325	0.0222	0.3798	0.2232	0.1707	0.1089	0.004	0.0585	4th
31145	31145	31497	31497	4023297	0.1363	0.0409	0.2609	0.3759	0.0539	0.0523	0	0.0262	UN
31145	31145	31498	31498	4023297	0.1566	0.0283	0.3279	0.2828	0.1475	0.2414	0	0.1207	3rd
31145	31145	31506	31506	4023297	0.125	0.0456	0.2681	0.341	0.0376	0.0303	0	0.0152	UN
31145	31145	31560	31560	4023297	0.1373	0.0277	0.2836	0.2487	0.1284	0.1091	0.0043	0.0589	4th
31234	31234	31498	31498	4023297	0.1438	0.0301	0.3197	0.2837	0.1015	0.0353	0	0.0177	UN
31234	31234	31560	31560	4023297	0.1298	0.0293	0.2876	0.2517	0.0898	0.0573	0.0018	0.0304	UN
30933	30933	31716	31716	4023297	0.133	0.0469	0.2492	0.3925	0.0222	0.0012	0	0.0006	UN
30933	30933	31805	31805	4023297	0.147	0.0476	0.273	0.4075	0.055	0.0013	0	0.0006	UN
30933	30933	31903	31903	4023297	0.1511	0.0644	0.2623	0.4279	0.0241	0	0	0	UN
31011	31011	31805	31805	4023297	0.138	0.0501	0.2556	0.4487	0.0395	0.0027	0	0.0013	UN
31021	31021	31720	31720	4023297	0.1352	0.0311	0.3046	0.2716	0.0881	0.0051	0	0.0025	UN
31021	31021	31800	31800	4023297	0.1317	0.0418	0.2626	0.3491	0.0754	0.0181	0	0.0091	UN
31021	31021	31805	31805	4023297	0.13	0.052	0.2534	0.4656	0.0327	0.0044	0	0.0022	UN
31021	31021	31966	31966	4023297	0.1285	0.0339	0.2805	0.3103	0.0701	0.0241	0	0.0121	UN
31021	31021	31970	31970	4023297	0.1216	0.046	0.251	0.3993	0.0282	0.0058	0	0.0029	UN
31028	31028	31800	31800	4023297	0.1337	0.05	0.2619	0.3481	0.045	0.0061	0.0021	0.0052	UN
31065	31065	31903	31903	4023297	0.1585	0.0568	0.2772	0.3758	0.0572	0	0	0	UN
31134	31134	31786	31786	4023297	0.1078	0.0301	0.2583	0.2772	0.0579	0.0029	0	0.0014	UN
31145	31145	31720	31720	4023297	0.143	0.0263	0.3342	0.2547	0.1291	0.2494	0.0045	0.1292	3rd
31145	31145	31800	31800	4023297	0.1276	0.0392	0.2567	0.3365	0.0728	0.0466	0	0.0233	UN
31145	31145	31805	31805	4023297	0.1294	0.0505	0.256	0.4659	0.0306	0.0119	0	0.0059	UN
31145	31145	31966	31966	4023297	0.136	0.0292	0.3074	0.2764	0.1088	0.0241	0	0.0121	UN
31234	31234	31720	31720	4023297	0.1097	0.0459	0.2606	0.3787	0.0297	0	0	0	UN
31234	31234	31800	31800	4023297	0.1215	0.0393	0.2626	0.3218	0.034	0	0	0	UN
31234	31234	31966	31966	4023297	0.1517	0.0191	0.3929	0.1913	0.2094	0.0917	0.0016	0.0474	4th
30933	30933	32187	32187	4023297	0.1479	0.0559	0.264	0.4217	0.0451	0.0057	0	0.0028	UN
30933	30933	32224	32224	4023297	0.146	0.0412	0.2674	0.3707	0.0764	0.0054	0	0.0027	UN
31011	31011	32187	32187	4023297	0.1551	0.0481	0.2843	0.3866	0.0832	0.0267	0.0152	0.0286	UN
31011	31011	32224	32224	4023297	0.1363	0.0458	0.2488	0.424	0.0537	0.0157	0.0026	0.0104	UN
31011	31011	32358	32358	4023297	0.1417	0.0536	0.263	0.4359	0.0364	0.0179	0	0.0089	UN
31021	31021	32039	32039	4023297	0.1484	0.0234	0.3676	0.2197	0.13	0.2852	0.0096	0.1522	3rd
31021	31021	32089	32089	4023297	0.1408	0.0396	0.2606	0.2851	0.059	0.1227	0	0.0614	4th
31021	31021	32224	32224	4023297	0.1268	0.0499	0.2426	0.461	0.0291	0.0089	0	0.0045	UN
31021	31021	32358	32358	4023297	0.1489	0.0345	0.2996	0.2889	0.1157	0.0457	0	0.0229	UN
31028	31028	32089	32089	4023297	0.1531	0.0334	0.2841	0.2161	0.1031	0.0251	0	0.0126	UN
31028	31028	32358	32358	4023297	0.1421	0.0442	0.276	0.3064	0.08	0.0035	0	0.0018	UN
31065	31065	32224	32224	4023297	0.1433	0.0448	0.2596	0.3915	0.0591	0.0013	0	0.0007	UN
31145	31145	32043	32043	4023297	0.1217	0.0404	0.2568	0.3985	0.0535	0.1567	0.0045	0.0828	4th
31145	31145	32089	32089	4023297	0.1408	0.0309	0.2646	0.2306	0.082	0.3224	0	0.1612	3rd
31145	31145	32358	32358	4023297	0.1409	0.0342	0.2838	0.2947	0.1003	0.0867	0	0.0433	UN
31145	31145	32467	32467	4023297	0.124	0.024	0.3138	0.2167	0.065	0.2383	0	0.1191	3rd
31021	31021	32849	32849	4023297	0.1236	0.0499	0.2462	0.4648	0.0329	0.0126	0	0.0063	UN
31021	31021	32995	32995	4023297	0.1361	0.0224	0.3488	0.2028	0.091	0.2498	0.0079	0.1328	3rd
31021	31021	33083	33083	4023297	0.1244	0.0346	0.2821	0.3052	0.0436	0.0078	0	0.0039	UN
31124	31124	32588	32588	4023297	0.1233	0.0227	0.3732	0.2164	0.1645	0.0521	0.0067	0.0328	UN
31145	31145	32849	32849	4023297	0.1232	0.0403	0.2493	0.3963	0.0679	0.2027	0	0.1013	3rd
31145	31145	33083	33083	4023297	0.1403	0.0221	0.3363	0.2197	0.134	0.2456	0	0.1228	3rd
31234	31234	32785	32785	4023297	0.1238	0.0283	0.3211	0.2501	0.1127	0.0549	0.0016	0.029	UN
31234	31234	33083	33083	4023297	0.1129	0.0365	0.2797	0.313	0.062	0	0	0	UN
30933	30933	6716	6716	4023297	0.1727	0.0437	0.2878	0.396	0.0804	0.0209	0	0.0104	UN
30933	30933	8170	8170	4023297	0.1222	0.0217	0.2422	0.2121	0.0628	0.091	0	0.0455	4th
30933	30933	8344	8344	4023297	0.1844	0.0288	0.3169	0.1958	0.1426	0.0994	0	0.0497	4th
30961	30961	7091	7091	4023297	0.1182	0.0235	0.2985	0.244	0.1126	0.1091	0	0.0546	4th
30961	30961	8395	8395	4023297	0.1087	0.0383	0.2645	0.3771	0.0252	0.0529	0	0.0264	UN
31011	31011	34857	34857	4023297	0.1341	0.0498	0.2525	0.4689	0.0257	0.0122	0	0.0061	UN
31011	31011	6265	6265	4023297	0.1232	0.0345	0.2438	0.342	0.0318	0.1496	0	0.0748	4th
31011	31011	6716	6716	4023297	0.1692	0.0339	0.284	0.3321	0.0996	0.1811	0	0.0905	3rd
31011	31011	7267	7267	4023297	0.1262	0.0308	0.2489	0.3084	0.0536	0.1883	0	0.0942	3rd
31011	31011	7311	7311	4023297	0.1209	0.0357	0.2445	0.3518	0.0117	0.1371	0.0097	0.0782	4th
31011	31011	7625	7625	4023297	0.1251	0.0311	0.2476	0.3111	0.0484	0.1637	0	0.0818	4th
31011	31011	8170	8170	4023297	0.1204	0.0363	0.2412	0.3627	0.0121	0.1469	0	0.0735	4th
31011	31011	8307	8307	4023297	0.1202	0.0352	0.2431	0.3466	0.0119	0.1639	0.0052	0.0872	4th
31011	31011	8344	8344	4023297	0.1686	0.057	0.2858	0.3891	0.0374	0.1528	0	0.0764	4th
31011	31011	8395	8395	4023297	0.1248	0.0337	0.2467	0.3338	0.0391	0.1805	0	0.0903	3rd
31016	31016	7091	7091	4023297	0.1125	0.0333	0.2813	0.327	0.058	0.0828	0	0.0414	UN
31021	31021	34568	34568	4023297	0.1434	0.0424	0.2658	0.3148	0.0526	0.1646	0	0.0823	4th
31021	31021	34857	34857	4023297	0.153	0.028	0.3204	0.2794	0.1527	0.2857	0.0019	0.1448	3rd
31021	31021	7091	7091	4023297	0.1233	0.0363	0.2638	0.3651	0.0545	0.2244	0	0.1122	3rd
31028	31028	34568	34568	4023297	0.1496	0.0383	0.2747	0.2505	0.081	0.0194	0	0.0097	UN
31028	31028	34857	34857	4023297	0.1362	0.0414	0.2696	0.3242	0.0746	0.0341	0	0.0171	UN
31065	31065	6716	6716	4023297	0.1703	0.0432	0.2813	0.3837	0.0798	0.0192	0	0.0096	UN
31065	31065	8170	8170	4023297	0.1203	0.0245	0.2359	0.2326	0.0458	0.0325	0	0.0163	UN
31086	31086	8170	8170	4023297	0.0954	0.0327	0.236	0.2797	0.0245	0.0112	0	0.0056	UN
31124	31124	33889	33889	4023297	0.0927	0.0324	0.2518	0.2904	0.0577	0.0036	0	0.0018	UN
31124	31124	8170	8170	4023297	0.1104	0.0245	0.2833	0.242	0.093	0.0669	0	0.0334	UN
31134	31134	7937	7937	4023297	0.105	0.0384	0.2664	0.3581	0.0446	0.0542	0	0.0271	UN
31145	31145	33863	33863	4023297	0.1198	0.0423	0.2522	0.4121	0.0427	0.1214	0	0.0607	4th
31145	31145	34568	34568	4023297	0.1419	0.0417	0.2661	0.3178	0.047	0.2839	0	0.142	3rd
31145	31145	34857	34857	4023297	0.1922	0.0109	0.4471	0.1324	0.2717	0.399	0.3	0.4996	FS
31145	31145	7091	7091	4023297	0.1256	0.0291	0.275	0.3062	0.0924	0.3593	0	0.1796	2nd
31234	31234	34857	34857	4023297	0.1407	0.0255	0.319	0.2477	0.1237	0.035	0	0.0175	UN
31234	31234	7091	7091	4023297	0.1117	0.0377	0.2596	0.3645	0.0613	0.0312	0	0.0156	UN
31234	31234	7937	7937	4023297	0.1093	0.0264	0.2638	0.2521	0.0993	0.0631	0	0.0315	UN
30933	30933	8465	8465	4023297	0.1584	0.0383	0.2723	0.3552	0.0981	0.0234	0	0.0117	UN
30933	30933	9860	9860	4023297	0.1536	0.0521	0.2605	0.4442	0.0507	0.004	0	0.002	UN
30961	30961	9128	9128	4023297	0.1136	0.0307	0.2801	0.3096	0.0681	0.0901	0	0.0451	4th
31011	31011	8465	8465	4023297	0.1665	0.031	0.2944	0.3135	0.1272	0.1641	0.0025	0.0846	4th
31011	31011	9045	9045	4023297	0.1194	0.0345	0.2416	0.338	0.0118	0.1601	0.0033	0.0834	4th
31011	31011	9128	9128	4023297	0.1245	0.0325	0.2463	0.3246	0.0422	0.149	0	0.0745	4th
31011	31011	9860	9860	4023297	0.1539	0.05	0.2645	0.4513	0.0528	0.0015	0	0.0008	UN
31015	31015	9860	9860	4023297	0.1602	0.0153	0.3276	0.124	0.1312	0.0126	0	0.0063	UN
31021	31021	8995	8995	4023297	0.1235	0.0354	0.2594	0.3381	0.0644	0.1299	0	0.0649	4th

FID1	ID1	FID2	ID2	N_SNP	HetHet	IBS0	HetConc	HomIBS0	Kinship	IBD1Seg	IBD2Seg	PropIBD	InfType
31021	31021	9562	9562	4023297	0.123	0.0332	0.2637	0.3339	0.0646	0.2719	0	0.1359	3rd
31065	31065	8465	8465	4023297	0.1603	0.0398	0.2751	0.3604	0.098	0.0222	0	0.0111	UN
31065	31065	9656	9656	4023297	0.1752	0.053	0.2889	0.2559	0.0554	0.0556	0	0.0278	UN
31065	31065	9860	9860	4023297	0.1581	0.0531	0.2686	0.4431	0.0558	0	0	0	UN
31124	31124	8780	8780	4023297	0.1075	0.0262	0.2696	0.2602	0.0727	0.0743	0	0.0372	UN
31134	31134	9562	9562	4023297	0.1161	0.0267	0.2899	0.2631	0.0979	0.0967	0	0.0483	4th
31145	31145	9841	9841	4023297	0.1224	0.0257	0.2732	0.2702	0.0919	0.4671	0	0.2335	2nd
31284	31284	31297	31297	4023297	0.1136	0.0467	0.247	0.4348	0.0235	0.0088	0	0.0044	UN
31284	31284	31331	31331	4023297	0.1333	0.0298	0.2729	0.2834	0.0966	0.0496	0.006	0.0308	UN
31297	31297	31299	31299	4023297	0.1525	0.0188	0.3884	0.2014	0.203	0.1858	0.0342	0.1271	3rd
31297	31297	31364	31364	4023297	0.1203	0.0312	0.2544	0.2934	0.0735	0.0301	0	0.015	UN
31299	31299	31364	31364	4023297	0.113	0.0367	0.2426	0.3352	0.0295	0.0088	0	0.0044	UN
31337	31337	31364	31364	4023297	0.1305	0.0471	0.2475	0.4186	0.0333	0.0066	0	0.0033	UN
31337	31337	31408	31408	4023297	0.1548	0.0377	0.2967	0.3086	0.1105	0.0143	0	0.0071	UN
31337	31337	31417	31417	4023297	0.1662	0.0274	0.3419	0.2456	0.1515	0.0241	0	0.012	UN
31337	31337	31477	31477	4023297	0.1484	0.0473	0.2774	0.3104	0.0752	0.019	0	0.0095	UN
31408	31408	31410	31410	4023297	0.1368	0.0557	0.2549	0.3968	0.0317	0.0015	0	0.0008	UN
31408	31408	31417	31417	4023297	0.138	0.0425	0.2746	0.3341	0.0662	0.0029	0	0.0015	UN
31408	31408	31477	31477	4023297	0.1438	0.0534	0.2728	0.3264	0.0509	0.0065	0	0.0032	UN
31409	31409	31462	31462	4023297	0.1256	0.0376	0.3103	0.3166	0.0715	0.034	0	0.017	UN
31417	31417	31477	31477	4023297	0.1383	0.0524	0.272	0.3375	0.0295	0.0053	0	0.0027	UN
31284	31284	31498	31498	4023297	0.1197	0.0383	0.2392	0.3569	0.0459	0.0213	0	0.0107	UN
31284	31284	31525	31525	4023297	0.1177	0.0429	0.2399	0.3451	0.0371	0	0	0	UN
31297	31297	31559	31559	4023297	0.1122	0.0266	0.2896	0.256	0.066	0.0525	0.0027	0.029	UN
31299	31299	31559	31559	4023297	0.1164	0.0259	0.3154	0.2503	0.0952	0.068	0.0012	0.0352	UN
31331	31331	31498	31498	4023297	0.133	0.0433	0.2556	0.3984	0.07	0.0038	0	0.0019	UN
31331	31331	31525	31525	4023297	0.1239	0.0464	0.2393	0.3681	0.039	0	0	0	UN
31337	31337	31497	31497	4023297	0.1621	0.0418	0.3042	0.3676	0.1094	0.0272	0	0.0136	UN
31337	31337	31498	31498	4023297	0.1294	0.0524	0.2391	0.473	0.0238	0.0189	0	0.0095	UN
31337	31337	31656	31656	4023297	0.1529	0.0484	0.2898	0.4099	0.0773	0.0015	0	0.0008	UN
31408	31408	31497	31497	4023297	0.1414	0.053	0.261	0.4126	0.0395	0	0	0	UN
31408	31408	31656	31656	4023297	0.1438	0.052	0.274	0.4015	0.057	0.0197	0.0013	0.0112	UN
31417	31417	31497	31497	4023297	0.1357	0.0481	0.2597	0.4024	0.0299	0.0094	0	0.0047	UN
31417	31417	31498	31498	4023297	0.1264	0.0497	0.2489	0.4376	0.0292	0.0113	0.0031	0.0087	UN
31477	31477	31497	31497	4023297	0.1442	0.0591	0.2646	0.3753	0.0296	0.005	0	0.0025	UN
31275	31275	31937	31937	4023297	0.1243	0.0465	0.2932	0.3346	0.0282	0.0027	0	0.0014	UN
31284	31284	31786	31786	4023297	0.1677	0.0176	0.4104	0.1881	0.2242	0.2194	0.0195	0.1292	3rd
31284	31284	31970	31970	4023297	0.1109	0.0382	0.2345	0.3332	0.0559	0.0048	0	0.0024	UN
31297	31297	31738	31738	4023297	0.1225	0.0247	0.2719	0.2244	0.1185	0.0269	0.0016	0.0151	UN
31297	31297	31764	31764	4023297	0.1218	0.0285	0.2848	0.2655	0.1108	0.0358	0	0.0179	UN
31297	31297	31882	31882	4023297	0.1497	0.0163	0.4272	0.1724	0.1984	0.3253	0.0063	0.169	3rd
31297	31297	31952	31952	4023297	0.1323	0.0248	0.3356	0.2484	0.1344	0.1264	0.0044	0.0677	4th
31299	31299	31738	31738	4023297	0.114	0.032	0.2563	0.2805	0.0681	0.0082	0	0.0041	UN
31299	31299	31764	31764	4023297	0.1165	0.0293	0.2782	0.2664	0.1048	0.0092	0	0.0046	UN
31299	31299	31882	31882	4023297	0.1151	0.0306	0.3106	0.2824	0.0711	0.065	0	0.0325	UN
31299	31299	31952	31952	4023297	0.1336	0.0256	0.3528	0.2564	0.1481	0.1027	0.0067	0.0581	4th
31311	31311	31805	31805	4023297	0.1599	0.0234	0.3586	0.2225	0.1598	0.1568	0.0082	0.0866	4th
31331	31331	31786	31786	4023297	0.1283	0.0346	0.2666	0.3236	0.0645	0.0283	0.0074	0.0216	UN
31331	31331	31805	31805	4023297	0.1282	0.0556	0.2436	0.4818	0.0256	0	0	0	UN
31331	31331	31970	31970	4023297	0.1227	0.0373	0.2482	0.3248	0.0504	0.0014	0	0.0007	UN
31333	31333	31902	31902	4023297	0.0946	0.0332	0.3076	0.2893	0.0609	0.0151	0.0035	0.0111	UN
31337	31337	31805	31805	4023297	0.1414	0.0438	0.2668	0.3966	0.0689	0.0399	0.0085	0.0285	UN
31337	31337	31903	31903	4023297	0.1554	0.042	0.2786	0.297	0.0858	0.0067	0	0.0033	UN
31358	31358	31852	31852	4023297	0.1406	0.0541	0.3243	0.3133	0.0327	0.0092	0	0.0046	UN
31364	31364	31738	31738	4023297	0.1394	0.0317	0.2982	0.2765	0.1125	0.0551	0.0045	0.032	UN
31364	31364	31764	31764	4023297	0.1296	0.0346	0.2856	0.3084	0.0716	0.0632	0.0012	0.0327	UN
31364	31364	31805	31805	4023297	0.123	0.051	0.2378	0.4423	0.0229	0.0023	0	0.0011	UN
31408	31408	31903	31903	4023297	0.1504	0.0506	0.273	0.3324	0.0465	0.0028	0	0.0014	UN
31409	31409	31716	31716	4023297	0.1331	0.0372	0.313	0.3056	0.06	0.0213	0	0.0107	UN
31409	31409	31880	31880	4023297	0.1357	0.0303	0.3554	0.249	0.1337	0.0192	0	0.0096	UN
31410	31410	31716	31716	4023297	0.1523	0.0385	0.3065	0.2983	0.0953	0.0306	0.0031	0.0184	UN
31410	31410	31846	31846	4023297	0.1863	0.0211	0.4054	0.1668	0.2066	0.1408	0.0013	0.0717	4th
31410	31410	31880	31880	4023297	0.136	0.0383	0.2881	0.2848	0.0418	0.0216	0	0.0108	UN
31462	31462	31716	31716	4023297	0.1353	0.0373	0.299	0.3271	0.0834	0.0433	0	0.0216	UN
31462	31462	31846	31846	4023297	0.1311	0.0443	0.2893	0.3389	0.054	0.0268	0.0017	0.0151	UN
31462	31462	31880	31880	4023297	0.131	0.0379	0.3151	0.3125	0.0911	0.0336	0	0.0168	UN
31477	31477	31903	31903	4023297	0.1488	0.0563	0.2664	0.3116	0.0312	0.0047	0	0.0024	UN
31297	31297	32025	32025	4023297	0.1516	0.0149	0.3812	0.1652	0.2166	0.2258	0.1359	0.2487	2nd
31297	31297	32040	32040	4023297	0.1291	0.0278	0.2939	0.2759	0.1236	0.182	0.0054	0.0964	3rd
31297	31297	32043	32043	4023297	0.1135	0.0479	0.25	0.4588	0.0243	0.0127	0	0.0064	UN
31299	31299	32025	32025	4023297	0.1315	0.028	0.3258	0.2871	0.1382	0.1167	0.0315	0.0898	3rd
31299	31299	32040	32040	4023297	0.1194	0.0314	0.2749	0.3025	0.0849	0.0622	0	0.0311	UN
31331	31331	32224	32224	4023297	0.1299	0.0485	0.2447	0.4363	0.0458	0.0057	0	0.0028	UN
31337	31337	32187	32187	4023297	0.1728	0.0314	0.3316	0.2551	0.1559	0.0327	0	0.0163	UN
31337	31337	32224	32224	4023297	0.1325	0.0535	0.243	0.4791	0.0299	0	0	0	UN
31364	31364	32025	32025	4023297	0.1162	0.0313	0.2488	0.2934	0.059	0.021	0	0.0105	UN
31364	31364	32040	32040	4023297	0.1444	0.0268	0.3155	0.2603	0.1357	0.113	0.0145	0.071	4th
31364	31364	32224	32224	4023297	0.1282	0.0493	0.2473	0.4377	0.0315	0.0043	0	0.0021	UN
31408	31408	32187	32187	4023297	0.1879	0.027	0.3803	0.2197	0.1886	0.1569	0.0277	0.1062	3rd
31408	31408	32358	32358	4023297	0.1435	0.0534	0.2767	0.3921	0.0537	0.0094	0	0.0047	UN
31409	31409	32224	32224	4023297	0.1465	0.0302	0.335	0.2693	0.0892	0.1387	0	0.0693	4th
31410	31410	32187	32187	4023297	0.1403	0.059	0.2549	0.4095	0.0266	0.002	0	0.001	UN
31410	31410	32224	32224	4023297	0.1451	0.0451	0.2742	0.3611	0.0764	0.0307	0.0012	0.0166	UN
31417	31417	32187	32187	4023297	0.1557	0.0316	0.3102	0.2478	0.1164	0.0101	0	0.005	UN
31417	31417	32372	32372	4023297	0.1334	0.032	0.3319	0.2535	0.0637	0.0862	0	0.0431	UN
31462	31462	32224	32224	4023297	0.1623	0.0252	0.3599	0.248	0.1521	0.1808	0.0077	0.0981	3rd
31477	31477	32187	32187	4023297	0.1529	0.0435	0.2857	0.2659	0.0894	0.0226	0	0.0113	UN
31477	31477	32356	32356	4023297	0.1481	0.0415	0.32	0.2369	0.0582	0.0561	0.004	0.032	UN
31297	31297	32909	32909	4023297	0.1464	0.0189	0.4061	0.1929	0.1815	0.291	0.0029	0.1484	3rd
31299	31299	32860	32860	4023297	0.1089	0.0245	0.3176	0.2231	0.0548	0.0943	0	0.0471	4th
31299	31299	32909	32909	4023297	0.1184	0.0332	0.3163	0.303	0.0727	0.0661	0	0.033	UN
31311	31311	32736	32736	4023297	0.1355	0.0323	0.339	0.2594	0.1164	0.024	0	0.012	UN
31333	31333	33115	33115	4023297	0.1052	0.0258	0.3477	0.2367	0.1181	0.0289	0.0031	0.0175	UN
31408	31408	32876	32876	4023297	0.1554	0.0406	0.3555	0.2738	0.0732	0.0834	0	0.0417	UN
31409	31409	33097	33097	4023297	0.1253	0.0345	0.3464	0.2766	0.104	0.0133	0.0012	0.0078	UN
31410	31410	32863	32863	4023297	0.1758	0.0294	0.3972	0.2079	0.1535	0.1224	0	0.0612	4th
31417	31417	33082	33082	4023297	0.1242	0.0479	0.2608	0.3072	0.0353	0.0018	0	0.0009	UN
31462	31462	33097	33097	4023297	0.1202	0.0401	0.3033	0.3201	0.0386	0.0286	0	0.0143	UN

FID1	ID1	FID2	ID2	N_SNP	HetHet	IBS0	HetConc	HomIBS0	Kinship	IBD1Seg	IBD2Seg	PropIBD	InfType
31477	31477	33082	33082	4023297	0.1708	0.0309	0.371	0.1896	0.1469	0.1118	0.0036	0.0595	4th
31275	31275	7937	7937	4023297	0.0976	0.0371	0.2348	0.3089	0.0447	0.037	0	0.0185	UN
31284	31284	6716	6716	4023297	0.1715	0.0218	0.3197	0.2119	0.1155	0.1956	0	0.0978	3rd
31284	31284	6955	6955	4023297	0.1144	0.0428	0.2482	0.4112	0.04	0.0648	0	0.0324	UN
31284	31284	7267	7267	4023297	0.1158	0.0418	0.2516	0.4024	0.0467	0.0722	0	0.0361	UN
31284	31284	7625	7625	4023297	0.1148	0.0426	0.2505	0.4092	0.0397	0.0596	0	0.0298	UN
31284	31284	8395	8395	4023297	0.1167	0.0415	0.2553	0.3985	0.0476	0.0737	0	0.0368	UN
31297	31297	33863	33863	4023297	0.1145	0.048	0.2534	0.4564	0.0267	0.0107	0	0.0054	UN
31297	31297	33908	33908	4023297	0.1121	0.0398	0.252	0.3781	0.0564	0.1392	0	0.0696	4th
31297	31297	6265	6265	4023297	0.1113	0.0479	0.2495	0.4706	0.0262	0.0153	0	0.0076	UN
31297	31297	6955	6955	4023297	0.1125	0.0476	0.2507	0.468	0.0293	0.0089	0	0.0045	UN
31297	31297	7091	7091	4023297	0.112	0.0462	0.2533	0.4602	0.0309	0.0137	0	0.0069	UN
31297	31297	7267	7267	4023297	0.1121	0.0479	0.2493	0.4703	0.027	0.0072	0	0.0036	UN
31297	31297	7625	7625	4023297	0.1117	0.0483	0.2498	0.4739	0.0268	0.0075	0	0.0038	UN
31297	31297	8395	8395	4023297	0.1124	0.048	0.2514	0.4698	0.0291	0.0065	0	0.0032	UN
31299	31299	33908	33908	4023297	0.1065	0.0426	0.2443	0.3927	0.0291	0.0391	0	0.0195	UN
31311	31311	33908	33908	4023297	0.1063	0.0368	0.2363	0.3173	0.0577	0.0075	0	0.0038	UN
31327	31327	8170	8170	4023297	0.1091	0.0216	0.2868	0.2186	0.0959	0.0818	0	0.0409	UN
31331	31331	6716	6716	4023297	0.1502	0.0424	0.254	0.3911	0.0338	0.0562	0	0.0281	UN
31337	31337	6716	6716	4023297	0.1604	0.0489	0.2681	0.4507	0.0402	0.0038	0	0.0019	UN
31337	31337	7158	7158	4023297	0.1391	0.0448	0.2493	0.2394	0.0657	0.2084	0	0.1042	3rd
31364	31364	6716	6716	4023297	0.1809	0.0248	0.3309	0.2344	0.1291	0.0843	0	0.0422	UN
31364	31364	6955	6955	4023297	0.1169	0.0426	0.2446	0.4008	0.028	0.0434	0	0.0217	UN
31408	31408	6716	6716	4023297	0.1814	0.0223	0.3209	0.1971	0.1443	0.1812	0	0.0906	3rd
31409	31409	8170	8170	4023297	0.1026	0.0316	0.2465	0.2831	0.0607	0.0273	0	0.0137	UN
31410	31410	33599	33599	4023297	0.1925	0.0227	0.4394	0.1784	0.2096	0.1467	0	0.0733	4th
31410	31410	33631	33631	4023297	0.1339	0.0461	0.2708	0.3455	0.026	0.0012	0	0.0006	UN
31410	31410	6716	6716	4023297	0.1824	0.0245	0.3186	0.2066	0.1422	0.1468	0	0.0734	4th
31417	31417	34857	34857	4023297	0.1218	0.0523	0.2434	0.4636	0.023	0.0034	0	0.0017	UN
31462	31462	33631	33631	4023297	0.1226	0.04	0.2758	0.3348	0.0693	0.0037	0	0.0018	UN
31462	31462	6265	6265	4023297	0.1081	0.0442	0.2403	0.4081	0.0335	0.0172	0	0.0086	UN
31462	31462	6955	6955	4023297	0.1085	0.0443	0.2396	0.4081	0.0344	0.0113	0	0.0057	UN
31462	31462	7267	7267	4023297	0.1095	0.0425	0.242	0.3941	0.0416	0.0211	0	0.0106	UN
31462	31462	7311	7311	4023297	0.107	0.0391	0.2448	0.3632	0.0398	0.037	0	0.0185	UN
31462	31462	7625	7625	4023297	0.1078	0.0451	0.2389	0.4164	0.0309	0.0188	0	0.0094	UN
31462	31462	8170	8170	4023297	0.1149	0.0225	0.2651	0.2206	0.1195	0.0824	0	0.0412	UN
31462	31462	8395	8395	4023297	0.1081	0.047	0.2394	0.4311	0.025	0.0166	0	0.0083	UN
31477	31477	7158	7158	4023297	0.1497	0.0421	0.2762	0.2006	0.0861	0.1716	0.0036	0.0894	3rd
31484	31484	7267	7267	4023297	0.1051	0.021	0.2816	0.1955	0.0506	0.0331	0	0.0166	UN
31284	31284	8995	8995	4023297	0.1169	0.0405	0.2537	0.3753	0.0542	0.0108	0	0.0054	UN
31284	31284	9128	9128	4023297	0.114	0.0436	0.2483	0.418	0.0347	0.0476	0	0.0238	UN
31284	31284	9562	9562	4023297	0.1189	0.0292	0.2651	0.2898	0.0916	0.1163	0	0.0581	4th
31297	31297	8780	8780	4023297	0.1276	0.0214	0.2993	0.2326	0.1499	0.4975	0	0.2488	2nd
31297	31297	8995	8995	4023297	0.1182	0.0391	0.2656	0.3775	0.068	0.0714	0	0.0357	UN
31297	31297	9128	9128	4023297	0.1117	0.0479	0.2498	0.4713	0.028	0.0064	0	0.0032	UN
31297	31297	9562	9562	4023297	0.1185	0.0364	0.2727	0.3695	0.078	0.1748	0	0.0874	4th
31299	31299	8780	8780	4023297	0.123	0.0209	0.2951	0.2226	0.1446	0.2652	0	0.1326	3rd
31299	31299	8995	8995	4023297	0.1122	0.0398	0.2569	0.3747	0.0443	0.0124	0	0.0062	UN
31299	31299	9562	9562	4023297	0.1111	0.0406	0.2598	0.3984	0.0488	0.0354	0	0.0177	UN
31311	31311	8780	8780	4023297	0.1088	0.0303	0.2448	0.287	0.0837	0.0594	0	0.0297	UN
31327	31327	8780	8780	4023297	0.1127	0.019	0.2944	0.1966	0.1086	0.0968	0	0.0484	4th
31331	31331	8465	8465	4023297	0.1765	0.0236	0.3315	0.2297	0.1564	0.2258	0.0013	0.1142	3rd
31331	31331	8995	8995	4023297	0.1201	0.0424	0.2446	0.3881	0.0233	0.0156	0	0.0078	UN
31331	31331	9562	9562	4023297	0.1248	0.0254	0.2621	0.2521	0.0857	0.2294	0	0.1147	3rd
31331	31331	9860	9860	4023297	0.1482	0.0497	0.2628	0.4393	0.0308	0.008	0	0.004	UN
31337	31337	8465	8465	4023297	0.1489	0.049	0.2581	0.4584	0.047	0.0014	0	0.0007	UN
31337	31337	8780	8780	4023297	0.1282	0.0202	0.2613	0.2109	0.0961	0.3263	0	0.1632	3rd
31337	31337	9860	9860	4023297	0.1457	0.0514	0.2497	0.4557	0.033	0.0083	0	0.0041	UN
31364	31364	8465	8465	4023297	0.1384	0.0405	0.2488	0.3776	0.0374	0.0327	0	0.0164	UN
31364	31364	8581	8581	4023297	0.1218	0.0421	0.2629	0.3237	0.031	0	0	0	UN
31364	31364	8780	8780	4023297	0.1236	0.0216	0.2662	0.2204	0.1108	0.1545	0	0.0772	4th
31408	31408	8465	8465	4023297	0.1458	0.0445	0.2567	0.3804	0.0487	0.071	0	0.0355	UN
31410	31410	8465	8465	4023297	0.1534	0.043	0.2696	0.3538	0.0694	0.0747	0	0.0374	UN
31417	31417	9562	9562	4023297	0.1209	0.0288	0.2626	0.2742	0.0842	0.1405	0	0.0702	4th
31462	31462	9128	9128	4023297	0.1078	0.0447	0.2388	0.4137	0.032	0.0312	0	0.0156	UN
31475	31475	8780	8780	4023297	0.0965	0.0363	0.2157	0.2867	0.0401	0.0029	0	0.0015	UN
31477	31477	8465	8465	4023297	0.1596	0.044	0.2847	0.2992	0.0742	0.1254	0	0.0627	4th
31497	31497	31498	31498	4023297	0.1423	0.044	0.2663	0.3917	0.0644	0.0252	0	0.0126	UN
31497	31497	31656	31656	4023297	0.1783	0.0327	0.3508	0.2764	0.1573	0.0012	0	0.0006	UN
31498	31498	31506	31506	4023297	0.1301	0.045	0.2716	0.3299	0.0328	0.0117	0	0.0059	UN
31498	31498	31560	31560	4023297	0.1412	0.0315	0.2834	0.2714	0.1149	0.0424	0	0.0212	UN
31498	31498	31656	31656	4023297	0.1334	0.0541	0.2523	0.4631	0.0307	0.0077	0	0.0038	UN
31500	31500	31624	31624	4023297	0.1014	0.0332	0.2604	0.3152	0.0639	0.0126	0.0012	0.0075	UN
31497	31497	31805	31805	4023297	0.1454	0.0499	0.2734	0.4281	0.0519	0.0019	0	0.001	UN
31497	31497	31903	31903	4023297	0.154	0.0628	0.2724	0.4241	0.0273	0.0012	0	0.0006	UN
31497	31497	31937	31937	4023297	0.1456	0.0467	0.2936	0.3536	0.0384	0.0012	0	0.0006	UN
31498	31498	31720	31720	4023297	0.1306	0.0377	0.2847	0.3234	0.0451	0.003	0	0.0015	UN
31498	31498	31800	31800	4023297	0.142	0.0313	0.2835	0.2659	0.1179	0.0337	0	0.0169	UN
31498	31498	31805	31805	4023297	0.1344	0.0513	0.2591	0.4597	0.0482	0.0032	0	0.0016	UN
31498	31498	31966	31966	4023297	0.1463	0.0266	0.3249	0.2483	0.1207	0.0297	0	0.0148	UN
31559	31559	31882	31882	4023297	0.097	0.035	0.2826	0.3064	0.0608	0.0136	0	0.0068	UN
31559	31559	31952	31952	4023297	0.1099	0.032	0.3079	0.2974	0.0737	0.0495	0	0.0248	UN
31560	31560	31800	31800	4023297	0.1322	0.0367	0.2653	0.2829	0.091	0.0018	0	0.0009	UN
31560	31560	31966	31966	4023297	0.1359	0.0313	0.3034	0.265	0.0957	0.0448	0.002	0.0244	UN
31656	31656	31805	31805	4023297	0.136	0.0534	0.2579	0.4446	0.0376	0.0013	0	0.0007	UN
31656	31656	31937	31937	4023297	0.15	0.042	0.3145	0.3155	0.0744	0.0012	0	0.0006	UN
31497	31497	32089	32089	4023297	0.1442	0.0569	0.2525	0.388	0.0331	0.0079	0	0.004	UN
31497	31497	32187	32187	4023297	0.1576	0.0461	0.2906	0.354	0.0927	0.0053	0	0.0026	UN
31497	31497	32207	32207	4023297	0.1448	0.0497	0.2798	0.3028	0.0418	0	0	0	UN
31497	31497	32358	32358	4023297	0.1396	0.0531	0.2583	0.4143	0.0348	0.0027	0	0.0014	UN
31498	31498	32039	32039	4023297	0.1336	0.0287	0.312	0.2565	0.0658	0.1522	0.0024	0.0785	4th
31498	31498	32089	32089	4023297	0.1446	0.04	0.2647	0.2878	0.0693	0.0759	0	0.038	UN
31498	31498	32224	32224	4023297	0.1292	0.0509	0.2438	0.4682	0.0365	0.0015	0	0.0008	UN
31498	31498	32358	32358	4023297	0.1493	0.0343	0.2948	0.2868	0.1211	0.0262	0	0.0131	UN
31500	31500	31993	31993	4023297	0.1207	0.0218	0.3652	0.2218	0.1464	0.1279	0	0.0639	4th
31506	31506	32358	32358	4023297	0.1698	0.0239	0.3834	0.1844	0.1743	0.1508	0.0079	0.0833	4th
31552	31552	32311	32311	4023297	0.0961	0.0308	0.3318	0.2624	0.0638	0.0142	0	0.0071	UN
31553	31553	31997	31997	4023297	0.0983	0.0315	0.3263	0.2792	0.0781	0.0294	0	0.0147	UN

FID1	ID1	FID2	ID2	N_SNP	HetHet	IBS0	HetConc	HomIBS0	Kinship	IBD1Seg	IBD2Seg	PropIBD	InfType
31559	31559	32025	32025	4023297	0.1279	0.0204	0.3535	0.2072	0.1415	0.1928	0.0095	0.1059	3rd
31559	31559	32040	32040	4023297	0.1081	0.0277	0.2702	0.2554	0.0421	0.0218	0	0.0109	UN
31560	31560	32089	32089	4023297	0.1382	0.0479	0.2558	0.3207	0.0268	0.012	0	0.006	UN
31560	31560	32358	32358	4023297	0.1324	0.0413	0.2593	0.3157	0.0665	0.0118	0	0.0059	UN
31656	31656	32187	32187	4023297	0.1489	0.0538	0.2774	0.4039	0.0516	0.011	0	0.0055	UN
31656	31656	32224	32224	4023297	0.1324	0.0412	0.2465	0.3626	0.0727	0.0013	0	0.0006	UN
31656	31656	32358	32358	4023297	0.1373	0.0551	0.2597	0.4197	0.036	0.0025	0	0.0012	UN
31656	31656	32469	32469	4023297	0.1428	0.0374	0.319	0.2912	0.0532	0.0211	0.0014	0.0119	UN
31498	31498	32849	32849	4023297	0.1268	0.0496	0.2493	0.463	0.0312	0.0058	0	0.0029	UN
31498	31498	32995	32995	4023297	0.1257	0.0271	0.3064	0.2373	0.0321	0.1655	0	0.0827	4th
31500	31500	32651	32651	4023297	0.106	0.0279	0.3236	0.2629	0.0669	0.0657	0.0015	0.0344	UN
31500	31500	32772	32772	4023297	0.0958	0.0371	0.2598	0.3415	0.0281	0	0	0	UN
31553	31553	32772	32772	4023297	0.1072	0.0304	0.3342	0.283	0.0909	0.0631	0.0013	0.0328	UN
31559	31559	32909	32909	4023297	0.0994	0.0404	0.2858	0.351	0.0336	0.012	0	0.006	UN
31624	31624	32588	32588	4023297	0.1161	0.0303	0.3265	0.28	0.094	0.0504	0.0038	0.029	UN
31497	31497	34568	34568	4023297	0.1499	0.0579	0.264	0.4052	0.0369	0.013	0	0.0065	UN
31497	31497	34857	34857	4023297	0.1364	0.0418	0.2582	0.3829	0.0553	0.0164	0	0.0082	UN
31497	31497	6716	6716	4023297	0.1591	0.0521	0.2628	0.4614	0.033	0	0	0	UN
31497	31497	7158	7158	4023297	0.1469	0.0508	0.2639	0.267	0.0629	0.1163	0	0.0581	4th
31497	31497	8344	8344	4023297	0.17	0.0473	0.2889	0.3185	0.0666	0.1356	0	0.0678	4th
31498	31498	34568	34568	4023297	0.1503	0.0318	0.2768	0.2388	0.1016	0.105	0	0.0525	4th
31498	31498	34857	34857	4023297	0.1573	0.0278	0.3257	0.2762	0.1523	0.1865	0.0016	0.0949	3rd
31498	31498	7091	7091	4023297	0.1232	0.0384	0.2582	0.3861	0.0374	0.1417	0	0.0708	4th
31500	31500	33889	33889	4023297	0.0989	0.0332	0.2662	0.3133	0.0572	0.0013	0	0.0006	UN
31500	31500	33908	33908	4023297	0.1123	0.0198	0.2763	0.1954	0.1134	0.0973	0	0.0486	4th
31500	31500	34897	34897	4023297	0.1161	0.0214	0.3559	0.2116	0.1324	0.0957	0	0.0478	4th
31500	31500	6265	6265	4023297	0.1111	0.0363	0.2722	0.3621	0.042	0.0821	0.0018	0.0429	UN
31500	31500	7091	7091	4023297	0.1156	0.0283	0.2891	0.2924	0.088	0.1106	0	0.0553	4th
31500	31500	7625	7625	4023297	0.1101	0.0401	0.2682	0.3974	0.0226	0.0511	0	0.0255	UN
31500	31500	8395	8395	4023297	0.1106	0.0375	0.2693	0.3729	0.0338	0.0562	0	0.0281	UN
31506	31506	34857	34857	4023297	0.122	0.0418	0.2567	0.3131	0.0405	0.0282	0	0.0141	UN
31525	31525	8344	8344	4023297	0.1591	0.0456	0.2824	0.2833	0.0337	0.0245	0	0.0122	UN
31560	31560	34568	34568	4023297	0.1442	0.0472	0.2689	0.3272	0.0367	0.0145	0	0.0073	UN
31560	31560	34857	34857	4023297	0.1408	0.0339	0.289	0.2991	0.1159	0.0715	0.0034	0.0392	UN
31578	31578	33889	33889	4023297	0.1138	0.0234	0.3573	0.2227	0.1333	0.0881	0.0048	0.0489	4th
31578	31578	7478	7478	4023297	0.0862	0.0314	0.2663	0.2679	0.0538	0.0048	0	0.0024	UN
31624	31624	33889	33889	4023297	0.0946	0.0359	0.2464	0.3242	0.0274	0	0	0	UN
31624	31624	6955	6955	4023297	0.108	0.0403	0.2554	0.3855	0.023	0.0227	0	0.0114	UN
31624	31624	7311	7311	4023297	0.1042	0.0409	0.2544	0.3886	0.0303	0.0364	0	0.0182	UN
31624	31624	8170	8170	4023297	0.1172	0.0219	0.2925	0.223	0.1283	0.1024	0	0.0512	4th
31624	31624	8307	8307	4023297	0.1027	0.0421	0.2502	0.399	0.0229	0.0452	0	0.0226	UN
31656	31656	6716	6716	4023297	0.163	0.0425	0.2777	0.3804	0.0583	0.0181	0	0.0091	UN
31656	31656	8170	8170	4023297	0.1169	0.0274	0.2397	0.2603	0.0526	0.0502	0	0.0251	UN
31497	31497	8465	8465	4023297	0.1519	0.0495	0.2618	0.4465	0.0533	0.0081	0	0.004	UN
31497	31497	9860	9860	4023297	0.1489	0.053	0.254	0.4542	0.0369	0.0039	0	0.002	UN
31498	31498	8465	8465	4023297	0.1415	0.0488	0.25	0.465	0.0248	0.0108	0	0.0054	UN
31498	31498	8995	8995	4023297	0.124	0.0358	0.2554	0.3422	0.055	0.0757	0	0.0379	UN
31498	31498	9562	9562	4023297	0.1225	0.0356	0.2568	0.3555	0.0457	0.1768	0	0.0884	3rd
31500	31500	8780	8780	4023297	0.1183	0.0204	0.2977	0.2203	0.1263	0.2014	0.0015	0.1022	3rd
31500	31500	9128	9128	4023297	0.1184	0.0254	0.2947	0.2626	0.1009	0.128	0.0018	0.0658	4th
31559	31559	8780	8780	4023297	0.1066	0.0273	0.275	0.2702	0.0564	0.0524	0	0.0262	UN
31624	31624	8780	8780	4023297	0.1107	0.0278	0.2679	0.282	0.0854	0.0673	0	0.0336	UN
31656	31656	8465	8465	4023297	0.1471	0.0433	0.2578	0.3884	0.0564	0.0083	0	0.0041	UN
31716	31716	31846	31846	4023297	0.1317	0.0474	0.2739	0.3529	0.0579	0.023	0	0.0115	UN
31716	31716	31880	31880	4023297	0.1306	0.0422	0.2942	0.3394	0.0484	0.0078	0	0.0039	UN
31720	31720	31966	31966	4023297	0.109	0.0431	0.2569	0.3524	0.0363	0	0	0	UN
31738	31738	31764	31764	4023297	0.1326	0.0309	0.308	0.2734	0.1093	0.0908	0.0014	0.0468	4th
31764	31764	31952	31952	4023297	0.1068	0.039	0.2605	0.338	0.0351	0.0013	0	0.0007	UN
31786	31786	31970	31970	4023297	0.1125	0.0369	0.2448	0.3238	0.0618	0	0	0	UN
31800	31800	31966	31966	4023297	0.1235	0.0368	0.2662	0.2978	0.0493	0	0	0	UN
31846	31846	31880	31880	4023297	0.1302	0.0423	0.2952	0.3088	0.0502	0.0135	0	0.0068	UN
31882	31882	31952	31952	4023297	0.1082	0.0346	0.3012	0.3094	0.0581	0.0366	0	0.0183	UN
31716	31716	32224	32224	4023297	0.194	0.0159	0.4339	0.1652	0.2426	0.2711	0.0063	0.1419	3rd
31720	31720	32467	32467	4023297	0.1123	0.0367	0.3105	0.2918	0.0306	0.0026	0	0.0013	UN
31738	31738	32025	32025	4023297	0.1151	0.0318	0.2569	0.285	0.0737	0.0307	0	0.0153	UN
31738	31738	32040	32040	4023297	0.1314	0.029	0.2919	0.2653	0.1232	0.1076	0.0161	0.0699	4th
31764	31764	32025	32025	4023297	0.1195	0.0292	0.2846	0.2708	0.1133	0.034	0	0.017	UN
31764	31764	32040	32040	4023297	0.1256	0.0314	0.2904	0.29	0.0993	0.0746	0.0041	0.0414	UN
31778	31778	32224	32224	4023297	0.1373	0.0363	0.3046	0.2985	0.05	0.0149	0	0.0075	UN
31778	31778	32451	32451	4023297	0.1157	0.0425	0.3061	0.3154	0.048	0.0127	0	0.0063	UN
31800	31800	32089	32089	4023297	0.1445	0.048	0.2691	0.318	0.0388	0.0096	0	0.0048	UN
31800	31800	32358	32358	4023297	0.1407	0.0409	0.2783	0.3061	0.0831	0	0	0	UN
31805	31805	32224	32224	4023297	0.1319	0.0524	0.2495	0.4673	0.0367	0	0	0	UN
31846	31846	32224	32224	4023297	0.1444	0.0353	0.2926	0.2814	0.0975	0.0522	0.0034	0.0294	UN
31880	31880	32224	32224	4023297	0.147	0.0335	0.3244	0.292	0.0878	0.1046	0	0.0523	4th
31882	31882	32025	32025	4023297	0.1184	0.0261	0.3187	0.2497	0.0943	0.1346	0	0.0673	4th
31903	31903	32187	32187	4023297	0.1585	0.0493	0.2833	0.3184	0.0718	0.0071	0.0012	0.0047	UN
31952	31952	32025	32025	4023297	0.1277	0.0231	0.3282	0.2315	0.1421	0.1105	0.0059	0.0611	4th
31952	31952	32040	32040	4023297	0.1142	0.0319	0.2712	0.2972	0.0602	0.0015	0	0.0008	UN
31716	31716	32988	32988	4023297	0.1384	0.0298	0.3445	0.2459	0.088	0.0266	0	0.0133	UN
31720	31720	33083	33083	4023297	0.136	0.0264	0.3613	0.234	0.1532	0.0309	0	0.0155	UN
31805	31805	32553	32553	4023297	0.1201	0.0429	0.2421	0.3293	0.0264	0.0025	0	0.0013	UN
31805	31805	32736	32736	4023297	0.1476	0.029	0.3388	0.2442	0.1059	0.1174	0.0028	0.0615	4th
31846	31846	32863	32863	4023297	0.1425	0.0373	0.3246	0.2478	0.098	0.0371	0.0034	0.0219	UN
31880	31880	33097	33097	4023297	0.1216	0.0391	0.3187	0.3033	0.0598	0.0073	0	0.0037	UN
31882	31882	32909	32909	4023297	0.1193	0.0264	0.3633	0.2399	0.1429	0.0393	0	0.0197	UN
31902	31902	32560	32560	4023297	0.0896	0.0342	0.2998	0.2897	0.028	0.015	0	0.0075	UN
31902	31902	33115	33115	4023297	0.1058	0.0264	0.3404	0.2425	0.1215	0.0328	0.0035	0.0199	UN
31938	31938	32801	32801	4023297	0.1229	0.032	0.3381	0.2555	0.1129	0.0122	0	0.0061	UN
31952	31952	32860	32860	4023297	0.1027	0.0305	0.3103	0.2675	0.031	0.0355	0	0.0177	UN
31952	31952	32909	32909	4023297	0.1189	0.0278	0.3345	0.2548	0.1176	0.0397	0	0.0198	UN
31966	31966	32785	32785	4023297	0.1367	0.0252	0.3638	0.224	0.149	0.0349	0.0045	0.022	UN
31693	31693	7267	7267	4023297	0.1035	0.0218	0.2794	0.2004	0.038	0.0059	0	0.003	UN
31716	31716	33599	33599	4023297	0.133	0.046	0.2863	0.3235	0.0548	0.0152	0	0.0076	UN
31716	31716	33631	33631	4023297	0.1337	0.0401	0.2895	0.3303	0.0751	0	0	0	UN
31716	31716	8170	8170	4023297	0.1167	0.0223	0.2539	0.2151	0.0975	0.053	0	0.0265	UN
31720	31720	34857	34857	4023297	0.1547	0.0214	0.3661	0.209	0.1642	0.2386	0.0092	0.1285	3rd
31738	31738	6716	6716	4023297	0.1643	0.0212	0.3025	0.1916	0.1048	0.1131	0	0.0565	4th

FID1	ID1	FID2	ID2	N_SNP	HetHet	IBS0	HetConc	HomIBS0	Kinship	IBD1Seg	IBD2Seg	PropIBD	InfType
31764	31764	6716	6716	4023297	0.1457	0.0272	0.2707	0.2491	0.0356	0.0759	0	0.0379	UN
31764	31764	6955	6955	4023297	0.1106	0.0454	0.2512	0.416	0.026	0.0294	0	0.0147	UN
31764	31764	7311	7311	4023297	0.1084	0.0428	0.2546	0.3923	0.0379	0.0578	0	0.0289	UN
31764	31764	7625	7625	4023297	0.1111	0.0441	0.2538	0.4055	0.0338	0.0364	0	0.0182	UN
31764	31764	8307	8307	4023297	0.1074	0.0445	0.2521	0.4058	0.0294	0.0415	0	0.0208	UN
31786	31786	6265	6265	4023297	0.1099	0.0468	0.244	0.4448	0.0254	0.0145	0	0.0072	UN
31786	31786	6716	6716	4023297	0.1619	0.0252	0.303	0.2444	0.0805	0.1151	0.0025	0.06	4th
31786	31786	6955	6955	4023297	0.1113	0.0448	0.2461	0.429	0.0378	0.0373	0	0.0187	UN
31786	31786	7267	7267	4023297	0.1134	0.0431	0.2515	0.4136	0.0482	0.0313	0	0.0156	UN
31786	31786	7311	7311	4023297	0.1086	0.0431	0.2479	0.4112	0.0254	0.057	0	0.0285	UN
31786	31786	7625	7625	4023297	0.1117	0.0441	0.2485	0.4227	0.0397	0.0386	0	0.0193	UN
31786	31786	8170	8170	4023297	0.1085	0.0444	0.2456	0.431	0.0238	0.032	0	0.016	UN
31786	31786	8395	8395	4023297	0.1123	0.044	0.2499	0.4198	0.0416	0.0329	0	0.0164	UN
31800	31800	34568	34568	4023297	0.1465	0.0353	0.2726	0.2465	0.0802	0.0159	0.0016	0.0096	UN
31800	31800	34857	34857	4023297	0.1359	0.036	0.2745	0.3106	0.0998	0.0445	0	0.0223	UN
31805	31805	33908	33908	4023297	0.121	0.0309	0.2504	0.2873	0.062	0.1049	0.0014	0.0538	4th
31805	31805	34857	34857	4023297	0.1273	0.0515	0.2476	0.4737	0.0285	0.0039	0	0.0019	UN
31805	31805	8344	8344	4023297	0.1647	0.0373	0.2889	0.2568	0.076	0.1975	0	0.0987	3rd
31846	31846	33599	33599	4023297	0.1427	0.0382	0.316	0.2639	0.1014	0.0364	0	0.0182	UN
31846	31846	6716	6716	4023297	0.1705	0.0239	0.311	0.1965	0.1114	0.0947	0	0.0473	4th
31880	31880	8170	8170	4023297	0.1046	0.0314	0.2428	0.2793	0.0769	0.0312	0	0.0156	UN
31903	31903	6716	6716	4023297	0.1671	0.0632	0.2713	0.442	0.0246	0.0038	0	0.0019	UN
31903	31903	7158	7158	4023297	0.1545	0.0571	0.2724	0.27	0.0457	0.0883	0	0.0442	UN
31903	31903	8344	8344	4023297	0.1704	0.0595	0.281	0.3386	0.0432	0.0637	0	0.0319	UN
31938	31938	33631	33631	4023297	0.118	0.0383	0.2823	0.3073	0.044	0.0184	0	0.0092	UN
31958	31958	33908	33908	4023297	0.1114	0.0271	0.2951	0.2345	0.0574	0.2008	0.0064	0.1068	3rd
31966	31966	34857	34857	4023297	0.1398	0.0291	0.3143	0.2749	0.1103	0.0174	0	0.0087	UN
31966	31966	7091	7091	4023297	0.1093	0.0406	0.251	0.383	0.0481	0.0346	0	0.0173	UN
31966	31966	7937	7937	4023297	0.1062	0.0276	0.2528	0.2586	0.0854	0.0407	0	0.0203	UN
31716	31716	8465	8465	4023297	0.1487	0.0415	0.2752	0.3787	0.0469	0.0638	0.0022	0.0341	UN
31720	31720	9841	9841	4023297	0.1054	0.0298	0.2508	0.2719	0.0865	0.0288	0	0.0144	UN
31738	31738	8780	8780	4023297	0.117	0.0323	0.2595	0.3048	0.0782	0.1273	0	0.0636	4th
31764	31764	8780	8780	4023297	0.1183	0.0301	0.2778	0.2945	0.1035	0.1598	0.0012	0.0811	4th
31764	31764	9045	9045	4023297	0.1056	0.044	0.2476	0.3984	0.0267	0.03	0	0.015	UN
31786	31786	8995	8995	4023297	0.1131	0.0427	0.2498	0.3942	0.0479	0.0071	0	0.0036	UN
31786	31786	9128	9128	4023297	0.1104	0.0449	0.2448	0.4297	0.0341	0.0257	0	0.0128	UN
31786	31786	9562	9562	4023297	0.118	0.0269	0.2697	0.2684	0.1094	0.1199	0	0.06	4th
31805	31805	8465	8465	4023297	0.1462	0.0498	0.26	0.4604	0.0297	0.0046	0	0.0023	UN
31805	31805	8780	8780	4023297	0.1241	0.0246	0.26	0.2522	0.0887	0.2491	0	0.1245	3rd
31849	31849	9562	9562	4023297	0.1078	0.0208	0.3121	0.1825	0.054	0.3108	0.034	0.1894	2nd
31882	31882	8780	8780	4023297	0.1033	0.0295	0.264	0.2824	0.0393	0.0353	0	0.0177	UN
31903	31903	9656	9656	4023297	0.1816	0.04	0.2991	0.1826	0.1031	0.0873	0	0.0436	UN
31952	31952	8780	8780	4023297	0.1132	0.0279	0.2772	0.2817	0.0885	0.0842	0	0.0421	UN
31970	31970	9562	9562	4023297	0.1117	0.0343	0.2474	0.3185	0.0639	0.0243	0	0.0121	UN
32025	32025	32040	32040	4023297	0.1272	0.0241	0.2952	0.241	0.1292	0.2022	0	0.1011	3rd
32089	32089	32358	32358	4023297	0.1525	0.0438	0.2813	0.289	0.0718	0.0232	0	0.0116	UN
32187	32187	32358	32358	4023297	0.1484	0.0548	0.2797	0.3964	0.0439	0.0172	0.0013	0.0099	UN
32224	32224	32358	32358	4023297	0.1328	0.0555	0.2506	0.4506	0.0303	0.0032	0	0.0016	UN
31993	31993	32651	32651	4023297	0.0977	0.0313	0.3211	0.281	0.0685	0.0158	0.0015	0.0094	UN
31997	31997	32772	32772	4023297	0.1116	0.0252	0.3642	0.2361	0.1207	0.0468	0.002	0.0254	UN
32025	32025	32909	32909	4023297	0.1169	0.029	0.3075	0.273	0.0831	0.1077	0	0.0539	4th
32039	32039	32995	32995	4023297	0.108	0.0329	0.3198	0.2609	0.07	0.0019	0.0112	0.0121	UN
32040	32040	33106	33106	4023297	0.1124	0.0286	0.3055	0.2404	0.0178	0.2268	0	0.1134	3rd
32043	32043	32849	32849	4023297	0.19	0.0099	0.4665	0.1174	0.277	0.4586	0.3375	0.5668	FS
32187	32187	32876	32876	4023297	0.1413	0.0412	0.3017	0.2652	0.0273	0.0704	0	0.0352	UN
32187	32187	33082	33082	4023297	0.1362	0.0434	0.2692	0.2635	0.0359	0.015	0	0.0075	UN
32224	32224	32591	32591	4023297	0.1342	0.0379	0.2988	0.3041	0.0337	0.0014	0	0.0007	UN
32224	32224	32988	32988	4023297	0.1418	0.0269	0.3347	0.2328	0.0806	0.1803	0	0.0901	3rd
32467	32467	33083	33083	4023297	0.1146	0.0327	0.3316	0.2699	0.0712	0.0042	0	0.0021	UN
31993	31993	34897	34897	4023297	0.0984	0.0338	0.3152	0.3044	0.066	0.0234	0	0.0117	UN
32025	32025	33908	33908	4023297	0.112	0.0365	0.2574	0.35	0.0651	0.174	0.0012	0.0882	4th
32025	32025	7091	7091	4023297	0.1105	0.0453	0.2547	0.4512	0.0324	0.0279	0	0.014	UN
32025	32025	8170	8170	4023297	0.1085	0.0472	0.2527	0.4706	0.0249	0.0077	0	0.0039	UN
32040	32040	33908	33908	4023297	0.1139	0.0399	0.252	0.3725	0.0514	0.1456	0.0043	0.0771	4th
32040	32040	6265	6265	4023297	0.1174	0.0394	0.2614	0.389	0.06	0.1815	0	0.0908	3rd
32040	32040	6716	6716	4023297	0.1489	0.0271	0.2691	0.268	0.0555	0.3816	0	0.1908	2nd
32040	32040	6955	6955	4023297	0.1188	0.0394	0.2634	0.3891	0.0648	0.1713	0	0.0857	4th
32040	32040	7267	7267	4023297	0.134	0.0163	0.3067	0.1704	0.174	0.5913	0	0.2957	2nd
32040	32040	7311	7311	4023297	0.1148	0.0392	0.2623	0.3857	0.0462	0.2164	0.0016	0.1098	3rd
32040	32040	7625	7625	4023297	0.1204	0.0344	0.2691	0.3449	0.0841	0.2473	0	0.1236	3rd
32040	32040	8170	8170	4023297	0.1145	0.0404	0.2589	0.4051	0.0441	0.1786	0	0.0893	3rd
32040	32040	8307	8307	4023297	0.1164	0.0362	0.2669	0.359	0.0598	0.256	0	0.128	3rd
32040	32040	8395	8395	4023297	0.1171	0.0413	0.2595	0.405	0.0538	0.1524	0	0.0762	4th
32043	32043	33863	33863	4023297	0.1243	0.0365	0.276	0.3575	0.0886	0.2424	0.0083	0.1294	3rd
32043	32043	34857	34857	4023297	0.1225	0.0395	0.2555	0.3891	0.0524	0.1812	0	0.0906	3rd
32043	32043	6265	6265	4023297	0.1153	0.0466	0.2561	0.4577	0.031	0.0099	0	0.005	UN
32043	32043	6955	6955	4023297	0.116	0.0468	0.2561	0.461	0.0341	0.0082	0	0.0041	UN
32043	32043	7091	7091	4023297	0.1146	0.0453	0.256	0.4495	0.0317	0.0105	0	0.0052	UN
32043	32043	7267	7267	4023297	0.1176	0.0459	0.2599	0.4515	0.041	0.0027	0	0.0013	UN
32043	32043	7625	7625	4023297	0.1156	0.0468	0.2559	0.4599	0.0318	0.0028	0	0.0014	UN
32043	32043	8395	8395	4023297	0.1164	0.0462	0.2581	0.4529	0.0357	0.0054	0	0.0027	UN
32089	32089	34568	34568	4023297	0.2409	0.022	0.4906	0.159	0.2682	0.2117	0.2691	0.375	FS
32089	32089	34857	34857	4023297	0.1388	0.047	0.2569	0.3434	0.0312	0.1621	0	0.0811	4th
32089	32089	6716	6716	4023297	0.1686	0.0628	0.2762	0.442	0.0252	0.0166	0	0.0083	UN
32089	32089	8344	8344	4023297	0.1688	0.0644	0.2795	0.3653	0.0253	0.0518	0	0.0259	UN
32132	32132	34859	34859	4023297	0.0917	0.0333	0.3048	0.2863	0.0631	0.0073	0	0.0037	UN
32187	32187	6716	6716	4023297	0.1938	0.0252	0.3403	0.2167	0.159	0.2408	0.0045	0.1249	3rd
32187	32187	7158	7158	4023297	0.1492	0.0505	0.2698	0.2578	0.0664	0.2052	0	0.1026	3rd
32224	32224	33599	33599	4023297	0.1397	0.0422	0.2891	0.3143	0.0576	0.0546	0	0.0273	UN
32224	32224	33631	33631	4023297	0.1423	0.0355	0.2975	0.3143	0.0841	0.0219	0	0.0109	UN
32224	32224	6265	6265	4023297	0.1222	0.0377	0.2498	0.3719	0.0345	0.1321	0	0.066	4th
32224	32224	6716	6716	4023297	0.1641	0.0353	0.2813	0.345	0.0797	0.201	0.0019	0.1024	3rd
32224	32224	6955	6955	4023297	0.1231	0.0388	0.2505	0.3834	0.0348	0.1307	0	0.0654	4th
32224	32224	7267	7267	4023297	0.1216	0.0406	0.2461	0.3987	0.0263	0.1235	0	0.0617	4th
32224	32224	7311	7311	4023297	0.119	0.0316	0.2486	0.3138	0.0404	0.2601	0	0.1301	3rd
32224	32224	7625	7625	4023297	0.1227	0.0387	0.2504	0.3819	0.0329	0.1473	0	0.0737	4th
32224	32224	8170	8170	4023297	0.1279	0.0163	0.2699	0.1719	0.117	0.4787	0.003	0.2424	2nd
32224	32224	8307	8307	4023297	0.1189	0.0344	0.2487	0.3401	0.0293	0.201	0.0014	0.1019	3rd

FID1	ID1	FID2	ID2	N_SNP	HetHet	IBS0	HetConc	HomIBS0	Kinship	IBD1Seg	IBD2Seg	PropIBD	InfType
32224	32224	8395	8395	4023297	0.1216	0.0407	0.2474	0.3972	0.0242	0.1262	0	0.0631	4th
32227	32227	33889	33889	4023297	0.1016	0.0282	0.3277	0.2522	0.0604	0.0484	0	0.0242	UN
32358	32358	34568	34568	4023297	0.1499	0.0382	0.2742	0.261	0.0831	0.0319	0	0.016	UN
32358	32358	34857	34857	4023297	0.1491	0.0256	0.3013	0.2236	0.1435	0.0956	0	0.0478	4th
32358	32358	6716	6716	4023297	0.1793	0.0175	0.3176	0.1547	0.1547	0.2042	0	0.1021	3rd
32467	32467	34857	34857	4023297	0.1275	0.0286	0.3205	0.255	0.0444	0.2482	0	0.1241	3rd
32472	32472	33631	33631	4023297	0.1341	0.0313	0.3422	0.25	0.0985	0.0265	0	0.0133	UN
31993	31993	8780	8780	4023297	0.1025	0.0301	0.2687	0.2973	0.0237	0.0401	0	0.02	UN
32025	32025	8780	8780	4023297	0.1294	0.0183	0.312	0.2014	0.1678	0.5506	0	0.2753	2nd
32025	32025	8995	8995	4023297	0.1163	0.0401	0.266	0.3852	0.054	0.0517	0	0.0258	UN
32025	32025	9562	9562	4023297	0.118	0.0348	0.2777	0.3551	0.0863	0.1923	0	0.0961	3rd
32040	32040	8465	8465	4023297	0.1351	0.0393	0.2527	0.3841	0.0175	0.161	0	0.0805	4th
32040	32040	8780	8780	4023297	0.1284	0.0201	0.2955	0.2167	0.1478	0.5076	0	0.2538	2nd
32040	32040	9045	9045	4023297	0.1128	0.0407	0.2573	0.3952	0.0352	0.1751	0	0.0876	4th
32040	32040	9128	9128	4023297	0.1185	0.0397	0.2641	0.3936	0.0618	0.1773	0	0.0886	3rd
32043	32043	8780	8780	4023297	0.1133	0.0459	0.2525	0.4672	0.0272	0.003	0	0.0015	UN
32043	32043	8995	8995	4023297	0.1179	0.0469	0.2601	0.4425	0.0391	0.0165	0.0013	0.0096	UN
32043	32043	9128	9128	4023297	0.116	0.0464	0.2572	0.4575	0.0338	0.0061	0	0.003	UN
32043	32043	9562	9562	4023297	0.1156	0.0446	0.2596	0.4423	0.0353	0.0107	0	0.0053	UN
32043	32043	9841	9841	4023297	0.1314	0.0148	0.3137	0.16	0.1699	0.6859	0	0.3429	2nd
32089	32089	9860	9860	4023297	0.1561	0.0647	0.2631	0.4425	0.0229	0.0042	0	0.0021	UN
32187	32187	8465	8465	4023297	0.1546	0.0438	0.2684	0.3652	0.0729	0.1085	0	0.0542	4th
32224	32224	8465	8465	4023297	0.1644	0.0364	0.2987	0.3626	0.1012	0.1572	0.0507	0.1293	3rd
32224	32224	9045	9045	4023297	0.1157	0.0369	0.2409	0.3592	0.0125	0.1777	0	0.0889	3rd
32224	32224	9128	9128	4023297	0.1234	0.0357	0.2525	0.356	0.0445	0.1673	0	0.0837	4th
32224	32224	9860	9860	4023297	0.1434	0.0498	0.2496	0.4505	0.0269	0.0138	0	0.0069	UN
32298	32298	8780	8780	4023297	0.0991	0.0261	0.2641	0.2424	0.0241	0.0251	0	0.0126	UN
32358	32358	8465	8465	4023297	0.1444	0.0472	0.2549	0.401	0.0365	0.0666	0	0.0333	UN
32560	32560	33115	33115	4023297	0.0895	0.0335	0.2936	0.2855	0.0235	0.0164	0	0.0082	UN
32588	32588	33889	33889	4023297	0.0891	0.0347	0.2463	0.2978	0.0369	0	0	0	UN
32588	32588	8170	8170	4023297	0.1037	0.0301	0.2683	0.2808	0.0457	0.0361	0	0.018	UN
32651	32651	34897	34897	4023297	0.0954	0.0339	0.3204	0.2967	0.0609	0.0108	0	0.0054	UN
32772	32772	33889	33889	4023297	0.0956	0.033	0.2682	0.2986	0.0599	0.0014	0	0.0007	UN
32772	32772	33908	33908	4023297	0.102	0.0322	0.2561	0.2938	0.0237	0.0299	0	0.0149	UN
32785	32785	7937	7937	4023297	0.0955	0.0352	0.237	0.2989	0.0374	0.02	0	0.01	UN
32801	32801	33631	33631	4023297	0.1232	0.0371	0.3064	0.2968	0.051	0.0176	0	0.0088	UN
32849	32849	33863	33863	4023297	0.1246	0.0381	0.2644	0.371	0.0648	0.2468	0.017	0.1404	3rd
32849	32849	34857	34857	4023297	0.1284	0.038	0.2592	0.3729	0.0807	0.2181	0	0.109	3rd
32863	32863	33599	33599	4023297	0.1495	0.0448	0.3584	0.2857	0.0968	0.0228	0	0.0114	UN
32863	32863	6716	6716	4023297	0.1512	0.0341	0.2801	0.2373	0.0264	0.0772	0	0.0386	UN
33083	33083	34857	34857	4023297	0.1625	0.0172	0.405	0.1786	0.1917	0.3323	0.0046	0.1708	3rd
32588	32588	8780	8780	4023297	0.1022	0.0287	0.2592	0.2716	0.0419	0.0348	0	0.0174	UN
32772	32772	8780	8780	4023297	0.1103	0.0261	0.2849	0.2628	0.0728	0.062	0	0.031	UN
32849	32849	9841	9841	4023297	0.1307	0.0151	0.2963	0.1618	0.1467	0.6933	0	0.3467	2nd
32909	32909	8780	8780	4023297	0.1059	0.0299	0.2677	0.2848	0.0499	0.0484	0	0.0242	UN
33083	33083	9841	9841	4023297	0.1059	0.0267	0.261	0.2504	0.0922	0.0365	0	0.0182	UN
33115	33115	8780	8780	4023297	0.107	0.0254	0.2826	0.2498	0.0585	0.0402	0	0.0201	UN
33599	33599	6716	6716	4023297	0.1554	0.0333	0.2835	0.2527	0.046	0.0374	0	0.0187	UN
33863	33863	34857	34857	4023297	0.1195	0.0432	0.2481	0.4185	0.0337	0.1135	0	0.0567	4th
33863	33863	6265	6265	4023297	0.1149	0.0474	0.2556	0.4625	0.0283	0.0155	0	0.0077	UN
33863	33863	6955	6955	4023297	0.1156	0.0467	0.2556	0.4569	0.0349	0.0178	0	0.0089	UN
33863	33863	7091	7091	4023297	0.1156	0.0454	0.2594	0.4494	0.0339	0.0033	0	0.0016	UN
33863	33863	7267	7267	4023297	0.1173	0.0447	0.2596	0.438	0.0454	0.0045	0	0.0023	UN
33863	33863	7625	7625	4023297	0.1156	0.0472	0.2568	0.4601	0.0316	0.016	0	0.008	UN
33863	33863	8395	8395	4023297	0.1162	0.0467	0.2581	0.4542	0.0348	0.0245	0	0.0123	UN
33889	33889	7478	7478	4023297	0.0927	0.0309	0.2703	0.2762	0.0479	0	0	0	UN
33889	33889	7937	7937	4023297	0.1159	0.0211	0.314	0.2098	0.1312	0.151	0.0144	0.0899	3rd
34568	34568	34857	34857	4023297	0.1451	0.0465	0.2707	0.3512	0.0411	0.1902	0	0.0951	3rd
34568	34568	6716	6716	4023297	0.1707	0.0561	0.2796	0.4085	0.0478	0.0159	0	0.0079	UN
34857	34857	7091	7091	4023297	0.1274	0.0308	0.2761	0.322	0.0836	0.3112	0	0.1556	3rd
6265	6265	6716	6716	4023297	0.1363	0.0298	0.2453	0.3056	0.0155	0.4324	0	0.2162	2nd
6265	6265	6955	6955	4023297	0.1819	0.0078	0.4822	0.0986	0.2961	0.5077	0.2944	0.5483	FS
6265	6265	7091	7091	4023297	0.1158	0.0439	0.2653	0.4502	0.0479	0.0043	0	0.0022	UN
6265	6265	7267	7267	4023297	0.1333	0.0233	0.3123	0.2517	0.1522	0.492	0	0.246	2nd
6265	6265	7311	7311	4023297	0.1273	0.0216	0.307	0.2316	0.1463	0.5255	0	0.2627	2nd
6265	6265	7625	7625	4023297	0.1727	0.0105	0.449	0.1278	0.2717	0.5338	0.2382	0.5051	FS
6265	6265	8170	8170	4023297	0.1267	0.0268	0.3019	0.2919	0.1272	0.4061	0	0.2031	2nd
6265	6265	8307	8307	4023297	0.129	0.019	0.3124	0.2047	0.1592	0.621	0	0.3105	2nd
6265	6265	8395	8395	4023297	0.1793	0.0075	0.4737	0.0948	0.2937	0.5579	0.2694	0.5484	FS
6716	6716	6955	6955	4023297	0.1386	0.0268	0.2489	0.2787	0.033	0.4657	0	0.2329	2nd
6716	6716	7267	7267	4023297	0.1384	0.0242	0.2481	0.2525	0.0426	0.5366	0	0.2683	2nd
6716	6716	7311	7311	4023297	0.1338	0.0213	0.2457	0.2214	0.0309	0.6161	0	0.308	2nd
6716	6716	7625	7625	4023297	0.1386	0.0214	0.2499	0.2233	0.0511	0.5921	0	0.2961	2nd
6716	6716	8170	8170	4023297	0.1329	0.0254	0.2417	0.2666	0.0172	0.5137	0	0.2569	2nd
6716	6716	8307	8307	4023297	0.1322	0.0235	0.2422	0.2428	0.019	0.5585	0	0.2793	2nd
6716	6716	8344	8344	4023297	0.1857	0.0603	0.2919	0.4147	0.0761	0.0257	0	0.0129	UN
6716	6716	8395	8395	4023297	0.138	0.0276	0.2483	0.2836	0.0282	0.4675	0	0.2338	2nd
6955	6955	7091	7091	4023297	0.1183	0.0435	0.2703	0.4474	0.0508	0.0111	0	0.0056	UN
6955	6955	7267	7267	4023297	0.1386	0.0168	0.3262	0.1867	0.1857	0.6241	0	0.3121	2nd
6955	6955	7311	7311	4023297	0.1281	0.0226	0.307	0.243	0.1407	0.5307	0	0.2654	2nd
6955	6955	7625	7625	4023297	0.1734	0.0124	0.4478	0.1519	0.2643	0.469	0.2427	0.4773	FS
6955	6955	8170	8170	4023297	0.1304	0.0231	0.311	0.2532	0.1449	0.4891	0	0.2445	2nd
6955	6955	8307	8307	4023297	0.1282	0.0209	0.3074	0.2247	0.1469	0.5462	0	0.2731	2nd
6955	6955	8395	8395	4023297	0.1766	0.0101	0.4594	0.1244	0.2781	0.5064	0.2542	0.5074	FS
7091	7091	7267	7267	4023297	0.1166	0.0449	0.265	0.4601	0.0419	0.0101	0	0.0051	UN
7091	7091	7311	7311	4023297	0.1124	0.0449	0.2636	0.457	0.033	0.0233	0	0.0117	UN
7091	7091	7625	7625	4023297	0.1168	0.044	0.2673	0.4517	0.0479	0.0113	0	0.0057	UN
7091	7091	8170	8170	4023297	0.1138	0.0442	0.2654	0.4602	0.0417	0.0266	0	0.0133	UN
7091	7091	8307	8307	4023297	0.1124	0.045	0.2639	0.4579	0.0323	0.0304	0	0.0152	UN
7091	7091	8395	8395	4023297	0.1168	0.0434	0.2669	0.4446	0.0498	0.011	0	0.0055	UN
7158	7158	8344	8344	4023297	0.1733	0.0621	0.295	0.2879	0.0306	0.4427	0.0015	0.2228	2nd
7267	7267	7311	7311	4023297	0.1312	0.0187	0.3159	0.2031	0.1605	0.6108	0	0.3054	2nd
7267	7267	7625	7625	4023297	0.139	0.0169	0.329	0.1876	0.1857	0.6302	0	0.3151	2nd
7267	7267	8170	8170	4023297	0.1287	0.0248	0.3051	0.2705	0.1346	0.4756	0	0.2378	2nd
7267	7267	8307	8307	4023297	0.1314	0.0177	0.3168	0.1925	0.1644	0.6317	0	0.3159	2nd
7267	7267	8395	8395	4023297	0.1356	0.0232	0.3181	0.2511	0.1571	0.4937	0	0.2468	2nd
7311	7311	7625	7625	4023297	0.1261	0.0246	0.3019	0.2623	0.131	0.4617	0	0.2308	2nd
7311	7311	8170	8170	4023297	0.1432	0.0167	0.3678	0.1937	0.2039	0.5343	0.111	0.3781	FS

FID1	ID1	FID2	ID2	N_SNP	HetHet	IBS0	HetConc	HomIBS0	Kinship	IBD1Seg	IBD2Seg	PropIBD	InfType
7311	7311	8307	8307	4023297	0.1624	0.0093	0.4443	0.113	0.2723	0.5467	0.2324	0.5058	FS
7311	7311	8395	8395	4023297	0.1229	0.0308	0.2919	0.3216	0.1012	0.3456	0	0.1728	3rd
7625	7625	8170	8170	4023297	0.1273	0.0281	0.3027	0.3049	0.1223	0.3782	0	0.1891	2nd
7625	7625	8307	8307	4023297	0.1293	0.019	0.3124	0.2049	0.1584	0.6034	0	0.3017	2nd
7625	7625	8395	8395	4023297	0.1776	0.0114	0.4653	0.1416	0.2764	0.4736	0.2573	0.4941	FS
8170	8170	8307	8307	4023297	0.14	0.0218	0.3569	0.2487	0.1784	0.3921	0.1206	0.3166	2nd
8170	8170	8395	8395	4023297	0.1255	0.0298	0.2968	0.3207	0.1121	0.3355	0.0025	0.1702	3rd
8307	8307	8395	8395	4023297	0.1289	0.0218	0.3108	0.2332	0.1465	0.5513	0	0.2756	2nd
33524	33524	8780	8780	4023297	0.1013	0.0392	0.2331	0.3421	0.0317	0.0119	0	0.0059	UN
33631	33631	8995	8995	4023297	0.1123	0.0429	0.2447	0.374	0.0432	0.0073	0	0.0037	UN
33863	33863	8780	8780	4023297	0.115	0.0459	0.2577	0.4647	0.0309	0.0029	0	0.0015	UN
33863	33863	8995	8995	4023297	0.1179	0.0466	0.2607	0.437	0.041	0.0094	0	0.0047	UN
33863	33863	9128	9128	4023297	0.1168	0.0466	0.2601	0.4577	0.0352	0.0271	0	0.0136	UN
33863	33863	9562	9562	4023297	0.1155	0.0459	0.2598	0.4506	0.0312	0.0114	0	0.0057	UN
33863	33863	9841	9841	4023297	0.1224	0.0293	0.2869	0.3013	0.0986	0.3782	0	0.1891	2nd
33889	33889	8780	8780	4023297	0.1145	0.0259	0.2944	0.2649	0.0873	0.1095	0	0.0548	4th
33908	33908	8780	8780	4023297	0.1339	0.0137	0.3206	0.1488	0.1915	0.6819	0.0274	0.3683	2nd
34857	34857	9841	9841	4023297	0.1255	0.0203	0.2781	0.2164	0.1124	0.557	0.0019	0.2804	2nd
34859	34859	8780	8780	4023297	0.1022	0.0231	0.2772	0.2226	0.0434	0.0283	0	0.0141	UN
6265	6265	8465	8465	4023297	0.1382	0.0281	0.2652	0.2959	0.0544	0.4307	0.0012	0.2165	2nd
6265	6265	8780	8780	4023297	0.1157	0.0431	0.265	0.4554	0.0504	0.0104	0	0.0052	UN
6265	6265	8995	8995	4023297	0.1163	0.0474	0.2613	0.4572	0.0337	0.0142	0	0.0071	UN
6265	6265	9045	9045	4023297	0.1294	0.017	0.3147	0.1839	0.1669	0.6458	0	0.3229	2nd
6265	6265	9128	9128	4023297	0.1836	0.0075	0.4918	0.0949	0.3024	0.5377	0.2973	0.5662	FS
6265	6265	9562	9562	4023297	0.1157	0.0439	0.2656	0.4478	0.0468	0.0191	0	0.0095	UN
6265	6265	9841	9841	4023297	0.1117	0.0448	0.2606	0.4556	0.0275	0.0081	0	0.004	UN
6716	6716	8465	8465	4023297	0.1902	0.0194	0.3143	0.2094	0.1772	0.5819	0.0035	0.2944	2nd
6716	6716	9128	9128	4023297	0.1367	0.0303	0.2457	0.3129	0.0153	0.4433	0	0.2216	2nd
6716	6716	9860	9860	4023297	0.177	0.0459	0.2846	0.4262	0.0915	0.0092	0	0.0046	UN
6955	6955	8465	8465	4023297	0.14	0.0242	0.2678	0.2577	0.0739	0.4506	0	0.2253	2nd
6955	6955	8581	8581	4023297	0.1038	0.0416	0.2309	0.3446	0.0288	0.0142	0	0.0071	UN
6955	6955	8780	8780	4023297	0.1175	0.0431	0.2681	0.4562	0.0506	0.0117	0	0.0059	UN
6955	6955	8995	8995	4023297	0.1172	0.0465	0.2618	0.4509	0.0409	0.0153	0	0.0076	UN
6955	6955	9045	9045	4023297	0.1236	0.0271	0.2942	0.2845	0.1143	0.405	0	0.2025	2nd
6955	6955	9128	9128	4023297	0.1682	0.0134	0.4289	0.1607	0.2515	0.4731	0.2045	0.4411	FS
6955	6955	9562	9562	4023297	0.117	0.044	0.2672	0.4495	0.0458	0.0157	0	0.0079	UN
6955	6955	9841	9841	4023297	0.1124	0.0449	0.2605	0.4572	0.0253	0.0075	0	0.0037	UN
7091	7091	8653	8653	4023297	0.115	0.0135	0.3085	0.1233	0.1347	0.8225	0.0014	0.4127	2nd
7091	7091	8780	8780	4023297	0.1165	0.0417	0.2695	0.4469	0.0605	0.0253	0	0.0127	UN
7091	7091	8995	8995	4023297	0.1162	0.0459	0.263	0.4486	0.036	0.0041	0	0.002	UN
7091	7091	9045	9045	4023297	0.1116	0.0458	0.2623	0.461	0.0269	0.0111	0	0.0055	UN
7091	7091	9128	9128	4023297	0.1164	0.0432	0.2663	0.4461	0.0503	0.0156	0	0.0078	UN
7091	7091	9562	9562	4023297	0.1161	0.0428	0.2691	0.4424	0.0549	0.0146	0	0.0073	UN
7091	7091	9841	9841	4023297	0.111	0.045	0.2606	0.4641	0.0287	0	0	0	UN
7267	7267	8465	8465	4023297	0.1395	0.023	0.2661	0.245	0.0778	0.5165	0	0.2583	2nd
7267	7267	8780	8780	4023297	0.1163	0.0442	0.2641	0.4663	0.0435	0.0062	0	0.0031	UN
7267	7267	8995	8995	4023297	0.1244	0.0407	0.2817	0.399	0.0749	0.0047	0	0.0024	UN
7267	7267	9045	9045	4023297	0.1257	0.026	0.3	0.2739	0.1215	0.4501	0	0.225	2nd
7267	7267	9128	9128	4023297	0.1309	0.0274	0.304	0.2934	0.1335	0.4139	0	0.207	2nd
7267	7267	9562	9562	4023297	0.1192	0.0432	0.2731	0.4421	0.0519	0.0102	0	0.0051	UN
7267	7267	9841	9841	4023297	0.1133	0.0457	0.2625	0.4641	0.0228	0.0035	0	0.0017	UN
7311	7311	8465	8465	4023297	0.1304	0.0269	0.2532	0.28	0.0342	0.4747	0	0.2373	2nd
7311	7311	8780	8780	4023297	0.1115	0.0459	0.2612	0.4781	0.0279	0.0088	0	0.0044	UN
7311	7311	9045	9045	4023297	0.1651	0.0126	0.4565	0.1518	0.2649	0.4406	0.277	0.4973	FS
7311	7311	9128	9128	4023297	0.1262	0.0246	0.3027	0.2618	0.1319	0.4814	0	0.2407	2nd
7311	7311	9562	9562	4023297	0.1126	0.0428	0.2649	0.434	0.0425	0.0552	0	0.0276	UN
7311	7311	9841	9841	4023297	0.1088	0.0467	0.2603	0.4695	0.0278	0.0207	0	0.0104	UN
7625	7625	8465	8465	4023297	0.1425	0.0176	0.2751	0.1915	0.1006	0.6318	0.0013	0.3172	2nd
7625	7625	8780	8780	4023297	0.1169	0.0435	0.2675	0.4596	0.05	0.0073	0	0.0036	UN
7625	7625	8995	8995	4023297	0.1171	0.0474	0.2625	0.4574	0.0361	0.0141	0	0.007	UN
7625	7625	9045	9045	4023297	0.1279	0.0212	0.3089	0.2268	0.1468	0.5497	0	0.2749	2nd
7625	7625	9128	9128	4023297	0.1679	0.0126	0.4301	0.1519	0.2556	0.5131	0.2053	0.4619	FS
7625	7625	9562	9562	4023297	0.1167	0.0438	0.2677	0.447	0.0479	0.0148	0	0.0074	UN
7625	7625	9841	9841	4023297	0.1117	0.0453	0.2598	0.4603	0.0243	0.0032	0	0.0016	UN
7777	7777	8653	8653	4023297	0.0802	0.0222	0.278	0.1633	0.0226	0.3366	0.0663	0.2346	2nd
7777	7777	9514	9514	4023297	0.0737	0.0288	0.2975	0.2032	0.0356	0.0759	0.0597	0.0976	3rd
7937	7937	9562	9562	4023297	0.1112	0.0443	0.2659	0.4393	0.0273	0.0205	0	0.0103	UN
7937	7937	9841	9841	4023297	0.108	0.0443	0.2631	0.4402	0.0315	0.0115	0	0.0057	UN
8170	8170	8465	8465	4023297	0.1325	0.0279	0.2562	0.2964	0.0377	0.4251	0	0.2126	2nd
8170	8170	8581	8581	4023297	0.0992	0.0397	0.225	0.3327	0.0338	0.0131	0	0.0065	UN
8170	8170	8780	8780	4023297	0.1139	0.0435	0.2655	0.4665	0.0444	0.0178	0	0.0089	UN
8170	8170	8995	8995	4023297	0.1142	0.0473	0.2609	0.4631	0.0224	0.0211	0	0.0105	UN
8170	8170	9045	9045	4023297	0.1297	0.0239	0.3231	0.2619	0.1503	0.4329	0.0343	0.2508	2nd
8170	8170	9128	9128	4023297	0.1262	0.0263	0.2996	0.2869	0.1271	0.4069	0	0.2034	2nd
8170	8170	9562	9562	4023297	0.1147	0.0438	0.2685	0.4534	0.046	0.0281	0	0.0141	UN
8170	8170	9841	9841	4023297	0.1091	0.0463	0.2588	0.4766	0.0261	0.0068	0	0.0034	UN
8307	8307	8465	8465	4023297	0.1305	0.0251	0.2535	0.2627	0.0406	0.4999	0	0.2499	2nd
8307	8307	8780	8780	4023297	0.1116	0.0462	0.2617	0.4823	0.0264	0.0131	0	0.0065	UN
8307	8307	9045	9045	4023297	0.1552	0.0158	0.4181	0.1848	0.2342	0.4193	0.2246	0.4343	FS
8307	8307	9128	9128	4023297	0.1257	0.0242	0.3013	0.2586	0.1319	0.4832	0	0.2416	2nd
8307	8307	9562	9562	4023297	0.1148	0.0429	0.2717	0.4363	0.0458	0.0636	0	0.0318	UN
8307	8307	9841	9841	4023297	0.1079	0.0458	0.2579	0.4622	0.0299	0.0114	0	0.0057	UN
8344	8344	8465	8465	4023297	0.1684	0.0684	0.2713	0.4723	0.024	0.0171	0	0.0085	UN
8344	8344	9656	9656	4023297	0.1884	0.0709	0.2945	0.3075	0.0497	0.056	0	0.028	UN
8344	8344	9860	9860	4023297	0.1748	0.0654	0.2829	0.4386	0.0422	0.015	0	0.0075	UN
8395	8395	8465	8465	4023297	0.1424	0.0239	0.2746	0.2538	0.0785	0.5372	0.004	0.2726	2nd
8395	8395	8780	8780	4023297	0.1163	0.0436	0.2657	0.4591	0.048	0.0122	0	0.0061	UN
8395	8395	8995	8995	4023297	0.1177	0.0471	0.264	0.4538	0.0385	0.0062	0	0.0031	UN
8395	8395	9045	9045	4023297	0.1308	0.0176	0.3177	0.1895	0.1654	0.6267	0	0.3133	2nd
8395	8395	9128	9128	4023297	0.1812	0.0088	0.4799	0.1112	0.2926	0.5326	0.2648	0.5311	FS
8395	8395	9562	9562	4023297	0.1164	0.0439	0.2665	0.4457	0.0464	0.0191	0	0.0095	UN
8395	8395	9841	9841	4023297	0.1126	0.0452	0.2619	0.4588	0.0256	0.0065	0	0.0033	UN
8465	8465	9045	9045	4023297	0.1307	0.0243	0.2546	0.2526	0.0432	0.5258	0	0.2629	2nd
8465	8465	9128	9128	4023297	0.1388	0.0267	0.2661	0.2838	0.0612	0.4422	0.0013	0.2224	2nd
8465	8465	9860	9860	4023297	0.164	0.0471	0.2723	0.4423	0.0894	0.0047	0	0.0024	UN
8635	8635	8653	8653	4023297	0.0819	0.0254	0.2642	0.1919	0.039	0.1893	0.0355	0.1301	3rd
8780	8780	8995	8995	4023297	0.1165	0.0457	0.264	0.4578	0.0374	0.0107	0	0.0054	UN
8780	8780	9045	9045	4023297	0.1107	0.0461	0.2598	0.4765	0.0239	0.0045	0	0.0023	UN

FID1	ID1	FID2	ID2	N_SNP	HetHet	IBS0	HetConc	HomIBS0	Kinship	IBD1Seg	IBD2Seg	PropIBD	InfType
8780	8780	9128	9128	4023297	0.1162	0.0436	0.2659	0.4613	0.0488	0.0069	0	0.0035	UN
8780	8780	9562	9562	4023297	0.1156	0.0426	0.2675	0.453	0.0545	0.0097	0	0.0049	UN
8780	8780	9841	9841	4023297	0.1104	0.0448	0.2587	0.4732	0.0284	0.0018	0	0.0009	UN
8995	8995	9128	9128	4023297	0.1168	0.047	0.2619	0.455	0.0367	0.0088	0	0.0044	UN
8995	8995	9562	9562	4023297	0.1345	0.0248	0.3183	0.2568	0.1459	0.3778	0	0.1889	2nd
9045	9045	9128	9128	4023297	0.1314	0.0166	0.3202	0.1814	0.1711	0.6455	0	0.3227	2nd
9045	9045	9562	9562	4023297	0.1126	0.0441	0.2661	0.4432	0.0364	0.0369	0	0.0184	UN
9045	9045	9841	9841	4023297	0.1086	0.046	0.2606	0.4609	0.0316	0.0175	0	0.0088	UN
9128	9128	9562	9562	4023297	0.1149	0.0438	0.2625	0.4471	0.0447	0.0115	0	0.0058	UN
9128	9128	9841	9841	4023297	0.1122	0.0446	0.2614	0.4562	0.028	0.0061	0	0.003	UN
9562	9562	9841	9841	4023297	0.1129	0.0438	0.2671	0.4507	0.0381	0.0012	0	0.0006	UN

#KING on all 33 founders

FID1	ID1	FID2	ID2	N_SNP	HetHet	IBS0	HetConc	HomIBS0	Kinship	IBD1Seg	IBD2Seg	PropIBD	InfType
1X0576	1X0576	1X0580	1X0580	4023297	0.0979	0.043	0.2694	0.3506	0.0252	0.1153	0	0.0576	4th
1X0102	1X0102	1X0110	1X0110	4023297	0.1426	0.0656	0.273	0.1875	0.0134	0.1438	0.0023	0.0742	4th
1X0014	1X0014	1X0035	1X0035	4023297	0.1078	0.0383	0.2746	0.3128	0.0584	0.2714	0.0042	0.1399	3rd

#KING on samples that had more than 0.1 on minor ancestries on either PANE or ADMIXTURE

FID1	ID1	FID2	ID2	N_SNP	HetHet	IBS0	HetConc	HomIBS0	Kinship	IBD1Seg	IBD2Seg	PropIBD	InfType
1X0351	1X0351	1X3321	1X3321	4023297	0.1857	0.0138	0.3016	0.0648	0.1937	0.4379	0	0.2189	2nd
14951	14951	18019	18019	4023297	0.1754	0.0351	0.3396	0.2375	0.135	0.1266	0.0148	0.0781	4th

#KING on samples with more than 0.1 on minor ancestries plus samples with *P. hamadryas* mitochondrial genome

FID1	ID1	FID2	ID2	N_SNP	HetHet	IBS0	HetConc	HomIBS0	Kinship	IBD1Seg	IBD2Seg	PropIBD	InfType
1X0351	1X0351	1X3321	1X3321	4023297	0.1857	0.0138	0.3016	0.0599	0.1937	0.5029	0	0.2514	2nd
1X0351	1X0351	14182	14182	4023297	0.1678	0.0692	0.2679	0.463	0.0271	0	0	0	UN
1X0812	1X0812	1X2124	1X2124	4023297	0.1204	0.0003	0.31	0.0029	0.1974	0.9942	0	0.4971	PO
10173	10173	10998	10998	4023297	0.1243	0.0204	0.3683	0.2161	0.1062	0.3468	0	0.1734	3rd
10173	10173	19679	19679	4023297	0.1268	0.0022	0.4001	0.0214	0.2057	0.6432	0.0462	0.3678	2nd
10173	10173	1X2124	1X2124	4023297	0.1523	0.0001	0.3725	0.0018	0.2661	0.9979	0	0.499	PO
10998	10998	19679	19679	4023297	0.0837	0.0233	0.3076	0.188	0.0834	0.0227	0	0.0113	UN
10998	10998	1X2124	1X2124	4023297	0.1033	0.0168	0.2791	0.1661	0.0539	0.0072	0	0.0036	UN
14182	14182	14951	14951	4023297	0.1793	0.0479	0.3136	0.3092	0.0969	0.3489	0.0337	0.2081	2nd
14182	14182	17268	17268	4023297	0.1714	0.0311	0.3602	0.1889	0.0854	0.395	0.0164	0.2139	2nd
14182	14182	18019	18019	4023297	0.157	0.0538	0.2797	0.3412	0.0301	0.1026	0.0053	0.0566	4th
14951	14951	18019	18019	4023297	0.1754	0.0351	0.3396	0.2174	0.135	0.131	0.0192	0.0848	4th
1X0356	1X0356	7267	7267	4023297	0.1232	0.0003	0.3162	0.0034	0.2097	0.9845	0	0.4923	PO
10173	10173	7267	7267	4023297	0.1191	0.0419	0.272	0.4916	0.0576	0.0194	0	0.0097	UN
10173	10173	8995	8995	4023297	0.119	0.0434	0.2709	0.4554	0.0507	0.0124	0	0.0062	UN
11885	11885	7267	7267	4023297	0.1284	0.0001	0.3693	0.0007	0.2169	0.8346	0.1606	0.5779	PO
15652	15652	7267	7267	4023297	0.1105	0.01	0.2901	0.0949	0.1286	0.3817	0	0.1909	2nd
1X2124	1X2124	32040	32040	4023297	0.1153	0.049	0.251	0.5102	0.0285	0.0013	0	0.0006	UN
1X2124	1X2124	7267	7267	4023297	0.1281	0.0374	0.2907	0.4467	0.0908	0.0229	0	0.0115	UN
1X2124	1X2124	8995	8995	4023297	0.1281	0.0385	0.2899	0.4118	0.0878	0.0161	0	0.0081	UN
32040	32040	33106	33106	4023297	0.1124	0.0286	0.3055	0.2473	0.0178	0.2028	0	0.1014	3rd
32040	32040	7267	7267	4023297	0.134	0.0163	0.3067	0.1878	0.174	0.5351	0	0.2676	2nd
7267	7267	8995	8995	4023297	0.1244	0.0407	0.2817	0.4415	0.0749	0.0029	0	0.0014	UN

			Complete set		Set A		Set B			Notes
ID	Pop	Reported ancestries*	Admixture	Pane	Admixture	Pane	Admixture	Pane (3 PC)	RFMIX	
12869	SNPRC	*hamadryas*	0.129634	0.1280191477	0.171213	0.1672536671	NA	NA	NA	
12962	SNPRC	*ursinus*	0.157818	0.1534412298	0.243033	0.1958635582	NA	NA	NA	
13264	SNPRC	*hamadryas*	0.17345	0.2141784833	0.235944	0.2567583535	NA	NA	NA	
13589	SNPRC	*ursinus*	0.132118	0.1243284093	0.245841	0.1951585689	NA	NA	NA	
13597	SNPRC	*hamadryas*	0.006373	0	0.033894	0.006375597653	NA	NA	NA	*No unexpected ancestry (>0.90 P. anubis/P. cynocephalus)*
13694	SNPRC	*hamadryas*	0.005465	0	0.034011	0.007089053423	NA	NA	NA	*No unexpected ancestry (>0.90 P. anubis/P. cynocephalus)*
14867	SNPRC	*hamadryas*	0.005714	0	0.035925	0.01171356126	NA	NA	NA	*No unexpected ancestry (>0.90 P. anubis/P. cynocephalus)*
14951	SNPRC	*hamadryas*	0.091886	0.118177646	0.140291	0.1481651753	NA	NA	NA	
14994	SNPRC	*hamadryas*	0.003079	0	0.039231	0.02445490645	NA	NA	NA	*No unexpected ancestry (>0.90 P. anubis/P. cynocephalus)*
15581	SNPRC	*hamadryas*	0.000707	0.01830814832	0.010895	0.02797499526	NA	NA	NA	*No unexpected ancestry (>0.90 P. anubis/P. cynocephalus)*
16246	SNPRC	*hamadryas*	0.000192	0.009078184399	0.008155	0.0241110726	NA	NA	NA	*No unexpected ancestry (>0.90 P. anubis/P. cynocephalus)*
18019	SNPRC	*hamadryas*	0.077709	0.1009706764	0.116877	0.1214095227	NA	NA	NA	
18778	SNPRC	*hamadryas*	0.000559	0.01211678647	0.009093	0.02250335458	NA	NA	NA	*No unexpected ancestry (>0.90 P. anubis/P. cynocephalus)*
1X0351	SNPRC	*kindae*	0.103252	0.1218674932	0.160152	0.2262904228	NA	NA	NA	
1X0356	SNPRC	*hamadryas*	0.137754	0.1515272839	0.144207	0.1635900771	0.19041	0.2868564811	0.1130819658	
1X0812	SNPRC	*hamadryas*	0.193632	Founder	0.120355	0.1943981624	0.187399	0.327885602	0.1820941819	
1X1126	SNPRC	*hamadryas*	0.00001	Used as source	0.00001	Used as source	NA	NA	NA	*No unexpected ancestry (>0.90 P. anubis/P. cynocephalus)*
1X1152	SNPRC	*hamadryas*	0.00001	Used as source	0.00001	Used as source	NA	NA	NA	*No unexpected ancestry (>0.90 P. anubis/P. cynocephalus)*
1X1734	SNPRC	*hamadryas*	0.00001	Founder	0.00001	Used as source	NA	NA	NA	*No unexpected ancestry (>0.90 P. anubis/P. cynocephalus)*
1X1947	SNPRC	*hamadryas*	0.00001	Founder	0.00001	Used as source	NA	NA	NA	*No unexpected ancestry (>0.90 P. anubis/P. cynocephalus)*
1X2049	SNPRC	*hamadryas*	0.00001	Founder	0.00001	Used as source	NA	NA	NA	*No unexpected ancestry (>0.90 P. anubis/P. cynocephalus)*
1X2124	SNPRC	*hamadryas*	0.120599	0.1184749381	0.107181	0.1121814998	0.087557	0.2116324683	0.05688069822	
1X2304	SNPRC	*hamadryas*	0.00001	Used as source	0.00001	Used as source	NA	NA	NA	*No unexpected ancestry (>0.90 P. anubis/P. cynocephalus)*
1X3321	SNPRC	*kindae*	0.088425	0.131862411	0.153327	0.2318769994	NA	NA	NA	
1x0576	SNPRC	*hamadryas*	0.008638	Founder	0.00001	Used as source	NA	NA	NA	*No unexpected ancestry (>0.90 P. anubis/P. cynocephalus)*
1X3548	SNPRC	*hamadryas*	0.00001	Used as source	0.00001	Used as source	NA	NA	NA	*No unexpected ancestry (>0.90 P. anubis/P. cynocephalus)*
28003	SNPRC	*papio*	0.162093	0.2016352213	0.210779	0.2216514933	NA	NA	NA	
28368	SNPRC	*hamadryas*	0.17516	0.190246772	0.237613	0.2342376238	NA	NA	NA	
30008	SNPRC	*papio*	0.13999	0.1540746714	0.194641	0.1861084769	NA	NA	NA	
31025	SNPRC	*hamadryas*	0.099826	0.1233918992	0.137354	0.1367181353	NA	NA	NA	
31015	SNPRC	*hamadryas*	0.097365	0.116351996	0.153859	0.1569486783	NA	NA	NA	
31115	SNPRC	*hamadryas*	0.101941	0.1014643066	0.159131	0.1348839434	NA	NA	NA	
31602	SNPRC	*hamadryas*	0.088659	0.1064693975	0.133717	0.1328708519	NA	NA	NA	
32606	SNPRC	*hamadryas*	0.117243	0.1372024365	0.168284	0.1676018077	NA	NA	NA	
8995	SNPRC	*hamadryas*	0.109473	0.1084849112	0.132773	0.1112735128	NA	NA	NA	
9481	SNPRC	*hamadryas*	0.00043	0.00983714993	0.008817	0.02394689107	NA	NA	NA	*No unexpected ancestry (>0.90 P. anubis/P. cynocephalus)*
10488	SNPRC	*hamadryas*	0.001532	0.0128449434	NA	NA	0.00001	0.04483104938	0.004211550318	*No unexpected ancestry (>0.90 P. anubis/P. cynocephalus)*
14182	SNPRC	*hamadryas*	0.029725	0.03925744908	NA	NA	0.00001	0.09422866507	0.02068628087	*No unexpected ancestry (>0.90 P. anubis/P. cynocephalus)*
1X1958	SNPRC	*hamadryas*	0.005832	0.02408132161	NA	NA	0.00001	0.04296428871	0.004717284992	*No unexpected ancestry (>0.90 P. anubis/P. cynocephalus)*
1X3796	SNPRC	*hamadryas*	0.017008	0.03841085384	NA	NA	0.00001	0.06441443884	0.003353573906	*No unexpected ancestry (>0.90 P. anubis/P. cynocephalus)*
1X3837	SNPRC	*hamadryas*	0.00001	0.004009088442	NA	NA	0.00001	0.05853273664	0.004472264396	*No unexpected ancestry (>0.90 P. anubis/P. cynocephalus)*
1X4209	SNPRC	*hamadryas*	0.011432	0.03668097666	NA	NA	0.00001	0.0784272628	0.007632844455	*No unexpected ancestry (>0.90 P. anubis/P. cynocephalus)*
9860	SNPRC	*hamadryas*	0.014417	0.02256615064	NA	NA	0.00001	0.06463454654	0.003888681439	*No unexpected ancestry (>0.90 P. anubis/P. cynocephalus)*

***Reported ancestries relative to each sample's detected non *P. anubis/P. cynocephalus* ancestry.**

If sample had no unexpected ancestry over 0.1, we use these samples as a control and reported their P. hamadryas ancestry.

Supplementary Info References

- Chiou KL, Janiak MC, Schneider-Crease IA, Sen S, Ayele F, Chuma IS, Knauf S, Lemma A, Signore AV, D'Ippolito AM, Abebe B, Haile AA, Kebede F, Fashing PJ, Nguyen N, McCann C, Houck ML, Wall JD, Burrell AS, …Snyder-Mackler N. Genomic signatures of high-altitude adaptation and chromosomal polymorphism in geladas. Nat Ecol Evol. 2022 May;6(5):630-643. doi: 10.1038/s41559-022-01703-4.
- Finstermeier K, Zinner D, Brameier M, Meyer M, Kreuz E, Hofreiter M, Roos C. A mitogenomic phylogeny of living primates. PLoS One. 2013 Jul 16;8(7):e69504. doi: 10.1371/journal.pone.0069504.
- Hodgson JA, Sterner KN, Matthews LJ, Burrell AS, Jani RA, Raaum RL, Stewart CB, Disotell TR. Successive radiations, not stasis, in the South American primate fauna. Proc Natl Acad Sci U S A. 2009 Apr 7;106(14):5534-9. doi: 10.1073/pnas.0810346106.
- Kendall C, Robinson J, Debortoli G, Nooranikhojasteh A, Christian D, Newman D, Sayers K, Cole S, Parra E, Schillaci M, Viola B. Global and local ancestry estimation in a captive baboon colony. PLoS One. 2024 Jul 3;19(7):e0305157. doi: 10.1371/journal.pone.0305157.
- Rogers J, Raveendran M, Harris RA, Mailund T, Leppälä K, Athanasiadis G, Schierup MH, Cheng J, Munch K, Walker JA, Konkel MK, Jordan V, Steely CJ, Beckstrom TO, Bergey C, Burrell A, Schrempf D, Noll A, Kothe M, …Baboon Genome Analysis Consortium. The comparative genomics and complex population history of *Papio* baboons. Sci Adv. 2019 Jan 30;5(1):eaau6947. doi: 10.1126/sciadv.aau6947.
- Roos C, Knauf S, Chuma IS, Maille A, Callou C, Sabin R, Portela Miguez R, Zinner D. New mitogenomic lineages in Papio baboons and their phylogeographic implications. Am J Phys Anthropol. 2021 Mar;174(3):407-417. doi: 10.1002/ajpa.24186.
- Sørensen EF, Harris RA, Zhang L, Raveendran M, Kuderna LFK, Walker JA, Storer JM, Kuhlwilm M, Fontsere C, Seshadri L, Bergey CM, Burrell AS, Bergman J, Phillips-Conroy JE, Shiferaw F, Chiou KL, Chuma IS, Keyyu JD, Fischer J, …Rogers J. Genome-wide coancestry reveals details of ancient and recent male-driven reticulation in baboons. Science. 2023 Jun 2;380(6648):eabn8153. doi: 10.1126/science.abn8153.
- Wall JD, Schlebusch SA, Alberts SC, Cox LA, Snyder-Mackler N, Nevonen KA, Carbone L, Tung J. Genomewide ancestry and divergence patterns from low-coverage sequencing data reveal a complex history of admixture in wild baboons. Mol Ecol. 2016 Jul;25(14):3469-83. doi: 10.1111/mec.13684. Epub 2016 Jun 15.
- Wall JD, Schlebusch SA, Alberts SC, Cox LA, Snyder-Mackler N, Nevonen KA, Carbone L, Tung J. Genomewide ancestry and divergence patterns from

low-coverage sequencing data reveal a complex history of admixture in wild baboons. Mol Ecol. 2016 Jul;25(14):3469-83. doi: 10.1111/mec.13684. Epub 2016 Jun 15.

- Zinner D, Wertheimer J, Liedigk R, Groeneveld LF, Roos C. Baboon phylogeny as inferred from complete mitochondrial genomes. Am J Phys Anthropol. 2013 Jan;150(1):133-40. doi: 10.1002/ajpa.22185. Epub 2012 Nov 26. Erratum in: Am J Phys Anthropol. 2016 Jun;160(2):364. doi: 10.1002/ajpa.22973.
