# Supplementary figures and images for: A Reassessment of the Genomic Ancestry of the World's Largest Captive Baboon Colony
Source: Am J Primatol. 2025 Nov 25;87(11):e70096. doi: 10.1002/ajp.70096 (PMC12646039; doi:10.1002/ajp.70096)

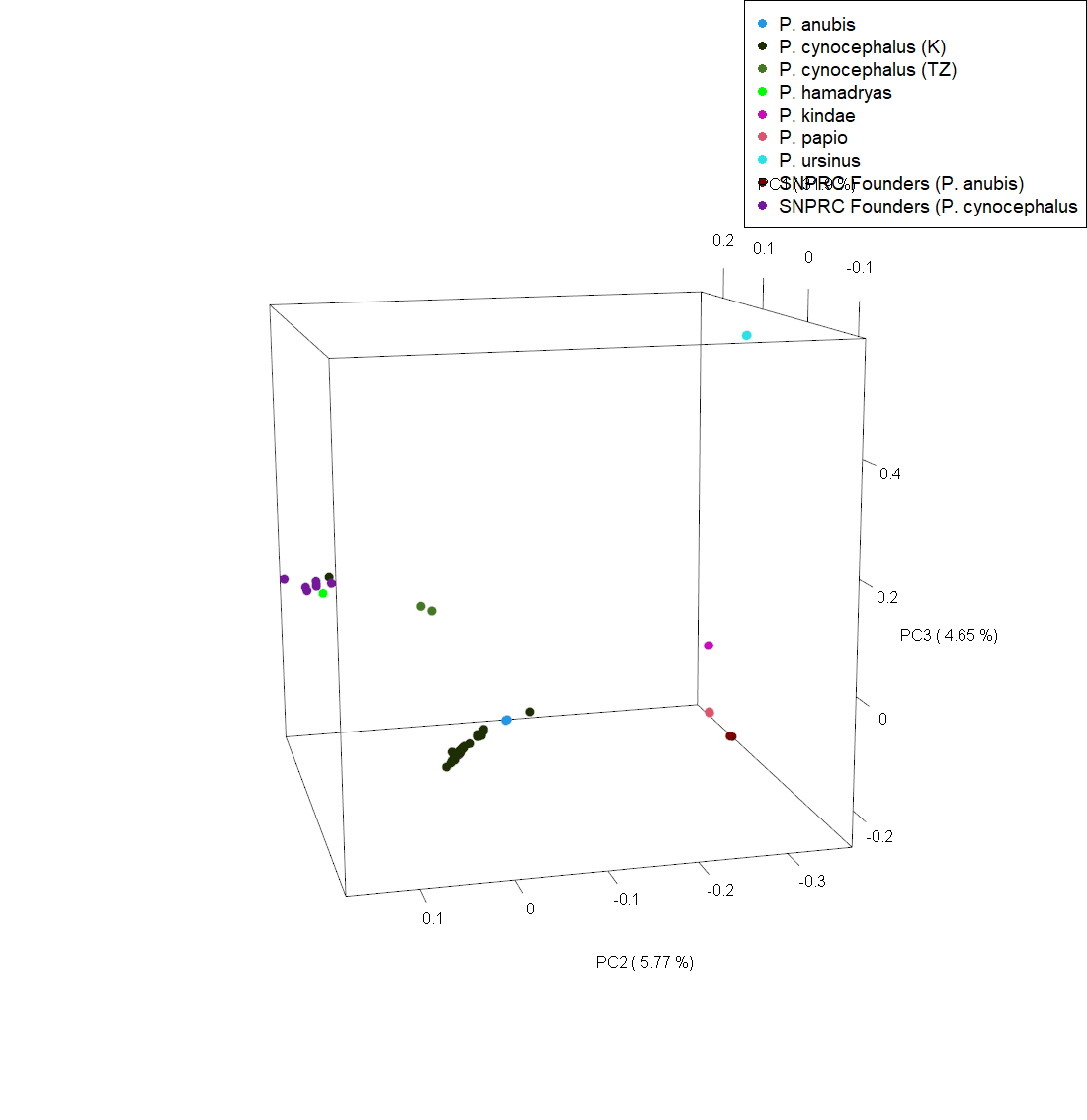

Supplement: Supplementary file 2 — Media_S1. [file AJP-87-e70096-s002.gif]

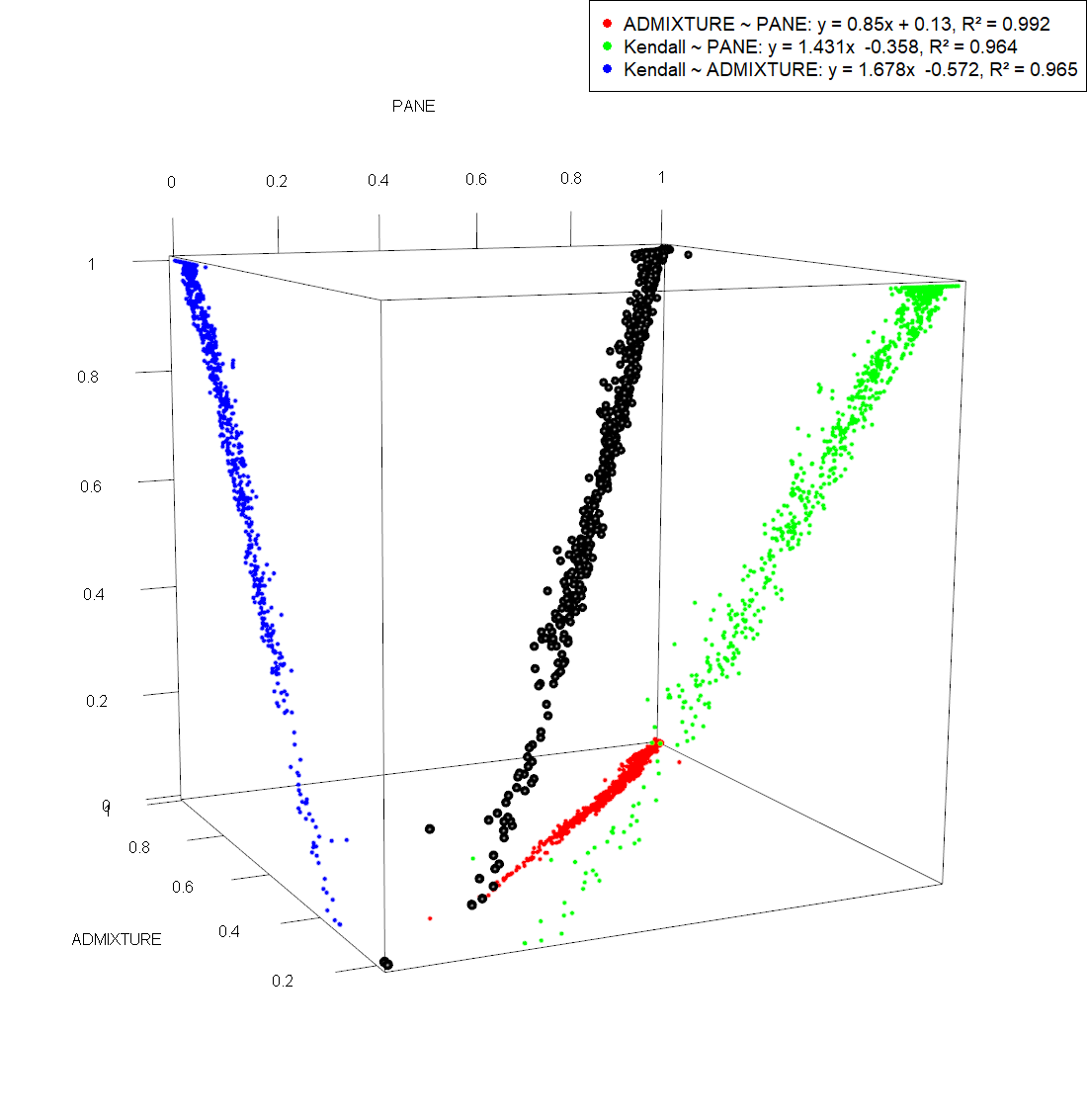

Supplement: Supplementary file 3 — Media_S2. [file AJP-87-e70096-s001.gif]

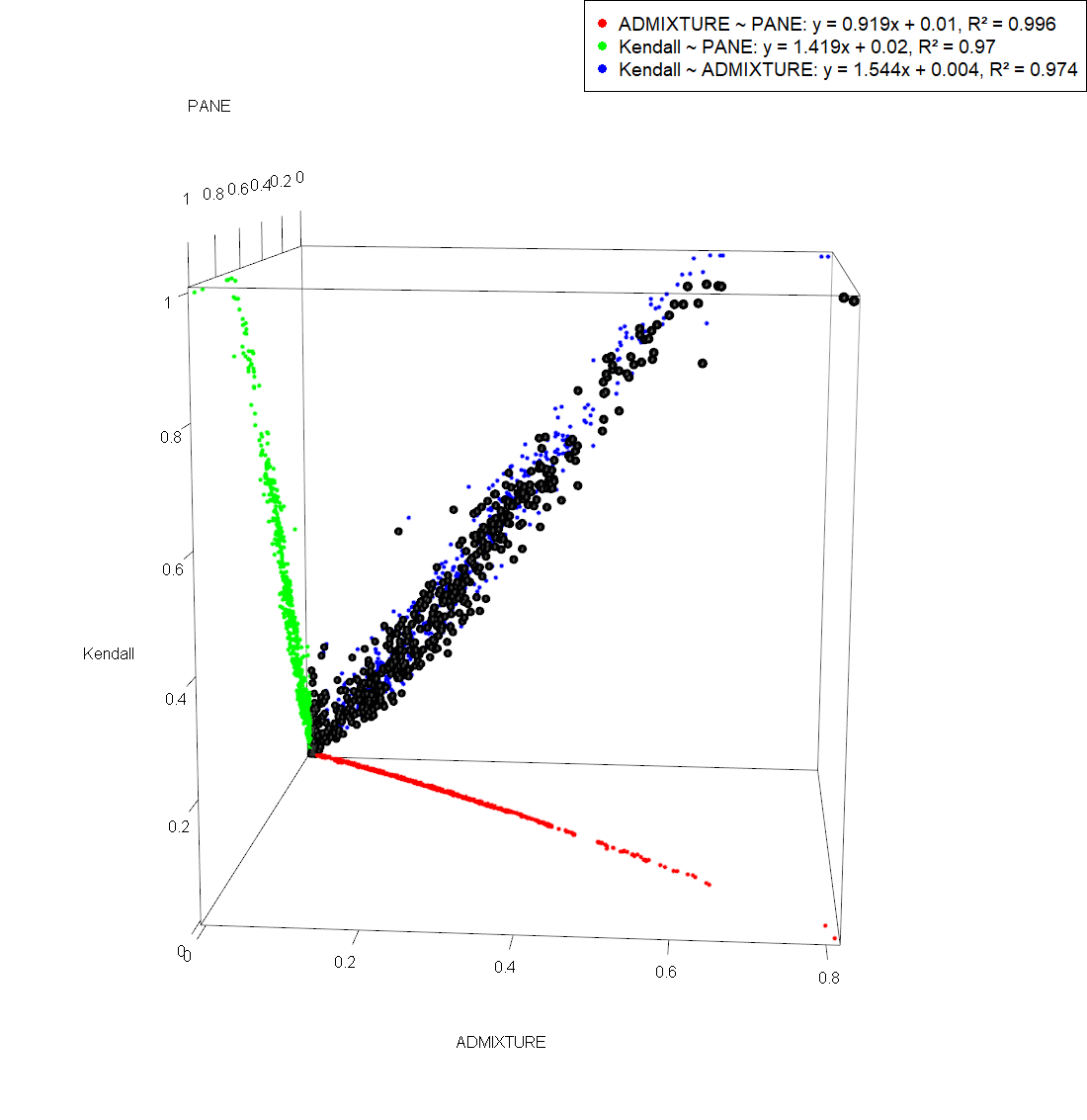

Supplement: Supplementary file 4 — Media_S3. [file AJP-87-e70096-s004.gif]
